# Copper-catalyzed carbonylative multi-component borylamidation of alkenes for synthesizing γ-boryl amides with CO as both methylene and carbonyl sources†

**DOI:** 10.1039/d4sc00156g

**Published:** 2024-02-05

**Authors:** Hui-Qing Geng, Yan-Hua Zhao, Peng Yang, Xiao-Feng Wu

**Affiliations:** a Leibniz-Institut für Katalyse e.V. Albert-Einstein-Straße 29a 18059 Rostock Germany xiao-feng.wu@catalysis.de; b Dalian National Laboratory for Clean Energy, Dalian Institute of Chemical Physics, Chinese Academy of Sciences 116023 Dalian Liaoning China xwu2020@dicp.ac.cn

## Abstract

A multi-component carbonylation reaction is an efficient strategy for the synthesis of valuable carbonyl compounds from simple and readily available substrates. However, there remain challenges in carbonylation reactions where two CO molecules are converted to different groups in the target product. Considering the merit of complex amides, we reported here a copper-catalyzed multi-component borylamidation for the synthesis of γ-boryl amides. This method provides access to a wide range of functional γ-boryl amides from alkenes, amines, B_2_pin_2_, and CO with good yields and excellent diastereomeric ratios. Notably, two CO molecules were converted to methylene and carbonyl groups in the target amides. A series of amines were successfully involved in the transformation, including arylamines, aliphatic amines, and hydrochloride salts of secondary aliphatic amines.

## Abstract 2

A copper-catalyzed multi-component borylamidation for the synthesis of γ-boryl amides has been developed.

## Graphical abstract

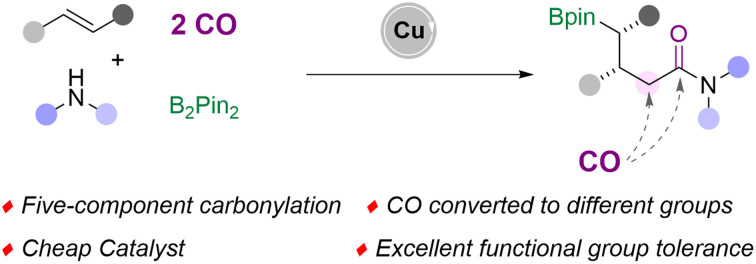

## Introduction

**Scheme 1:**
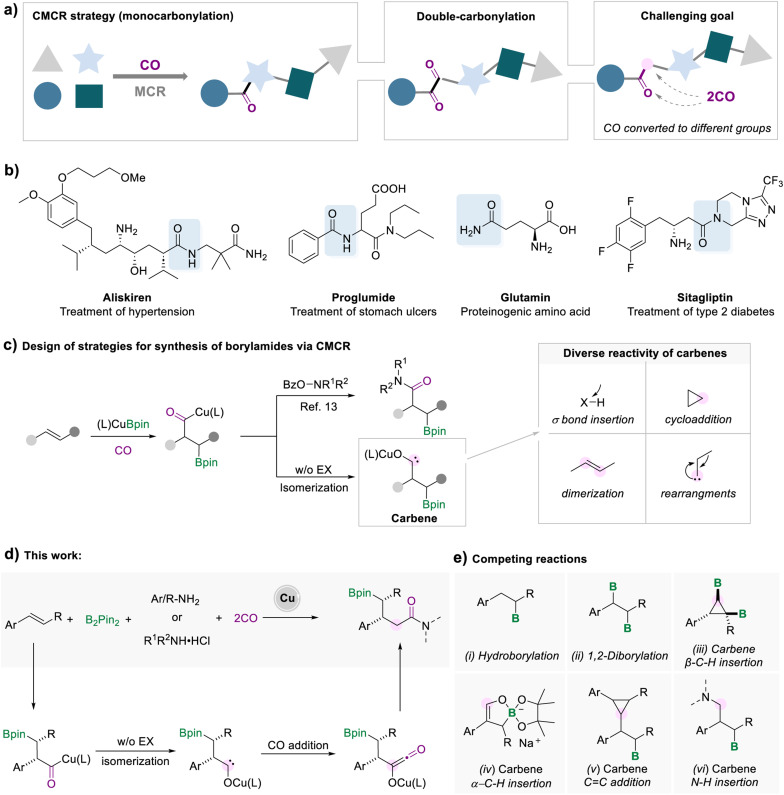
Strategies for synthesis of borylamides *via* a multicomponent carbonylation reaction (CMCR).

The synthesis of complex compounds *via* a more efficient and convenient process is a long-standing goal in organic synthesis. In the contemporary synthetic and pharmaceutical fields, a multi-component reaction (MCR) is considered as an ideal protocol for the one-pot preparation of complex compounds from readily available and inexpensive starting materials.^1^ A multicomponent carbonylation reaction (CMCR) is a straightforward process for the synthesis of valuable carbonyl compounds (Scheme 1a).^2^ The most common form of the CMCR is monocarbonylation, due to the susceptibility of acyl-metal species to nucleophilic attack or elimination. There are limited examples of carbonylation processes involving two or more carbon monoxide molecules. Representative examples include the Fischer–Tropsch process for the synthesis of liquid hydrocarbons^3^ and the double-carbonylation processes for the synthesis of 1,2-diketones.^4^ In these transformations, multiple CO molecules were converted to a single type of motif in the target compounds, either alkyl or carbonyl groups. Introducing two CO molecules into the target product and controlling the transformation of CO to different groups are enormously difficult tasks.^5^

Among various carbonyl-containing compounds, amide fragments are widely used in all facets of pharmaceuticals, proteins, agrochemicals, and versatile high-value products (Scheme 1b).^6^ The beneficial properties of amide groups, such as biological activity and stability, make them one of the most valuable functional groups. Despite the prevalence of amides, the existing techniques for the synthesis of amides are still associated with some drawbacks, such as poor atom efficiency, toxicity, high cost, harsh conditions, or limited substrate scope.^7^ Due to the breadth and importance of amide motifs, innovation in developing efficient methodologies is highly sought after, especially for amides with complex and diverse structures.

One efficient strategy for synthesizing complex amide structures is to form an amide bond, while simultaneously installing a reaction handle on the target product molecule *via* MCRs. Among the myriad of functionalizable reaction handles, organoboronates have attracted much attention, benefiting from the fact that carbon–boron bonds can be diversely converted to other carbon–carbon or carbon–heteroatom bonds.^8^ Currently, there are several strategies for the synthesis of boron-containing amides, for example, hydroboration of α, β-unsaturated amides,^9^ transition metal-catalyzed C–H borylation,^10^ and borylamidation of olefins with isocyanates.^11^ However, these strategies are limited to specific unsaturated substrates, or the pre-setting of the amide functional group, which increases the tediousness of the synthetic process, or toxic substrates.

In recent years, borocarbonylation catalyzed by copper has been explored with great interest.^12^ In 2020, our group reported a copper-catalyzed borylamidation for the synthesis of β-borylamides (Scheme 1c).^13^ The formed acyl-copper species could undergo oxidative addition to electrophiles and reductive elimination to afford the desired β-borylamides. Upon further investigation, our group found that the borocarbonylation of unsaturated alkenes in the absence of electrophilic reagents followed a completely different mechanism. Under a CO atmosphere, alkyl copper traps CO to form acyl copper, which can isomerize to carbene intermediates for subsequent conversion.^5*a*,14^ For example, we developed the cyclopropanation of two molecular alkenes, which involved the addition process of the carbene intermediates to the C=C double bonds.^14*f*^

As highly reactive intermediates, carbene can participate in various synthetic tasks, including σ bond insertion, cyclopropanation, dimerization, and rearrangement.^15^ Thus, a wide range of reactions of carbene species can be carried out to generate marvelous products, making this type of reaction extremely valuable to study (Scheme 1c). Here, we developed a copper-catalyzed five-component borylamidation for the synthesis of γ-borylamides, in which the two CO molecules were separately converted to the carbonyl group and a methylene group (Scheme 1d). We envisage that the borylamidation process involves the reaction of carbene intermediates with CO. After the subsequent reaction of the formed ketene species with amines, a range of γ-boryl amides were obtained. However, several competitive side reactions would occur due to the high reactivity of the carbene intermediates and increase the reaction complexity consequently (Scheme 1e).

## Results and discussion

For establishing the transformation, we selected *trans*-β-methylstyrene, aniline, and B_2_pin_2_ as the model substrates initially to optimize the reaction conditions (Table 1, for more details see the ESI†). Using a CuI/DPPE catalyst system with NaO^*t*^Bu as the base in DMAc solvent, γ-borylamide (1a) was obtained in 24% yield (Table 1, entry 1). A series of phosphine ligands were tested at the first stage (Table 1, entries 1–3 and Table S1 in the ESI†). We found that ligands play an important role in avoiding other side reactions such as boroaminomethylation^14*a*^ or cyclopropanation.^14*e*,*f*^ For example, when DPPP was used as the ligand, this conversion tended to generate cyclopropyl bis(boronates) *via* intramolecular carbene insertion (Scheme 1d(iii)). The cyclopropanation of two alkene molecules tended to occur when DPPB was used as the ligand (Scheme 1d(v)).^14*f*^ The borylamidation showed good selectivity to generate the desired amide 1a when DPPE was used (Table 1, entry 1). Polar solvents with good solubility, such as NMP, DMSO, or DMF, worked better than toluene for this reaction (Table 1, entries 4–7). The yield of 1a increased significantly using DMSO as the solvent (Table 1, entry 5). Notably, dimethyl sulfoxide is considered as a green solvent, which was classified by the FDA as the safest “Class 3” solvent, in the same class as ethanol.^16^ Subsequently, various copper pre-catalysts were investigated (Table 1, entries 8–12). From our obtained results, the copper salts applied have a significant effect on the reaction outcome and Cu(OAc)_2_ was found to be the best (Table 1, entry 11). Copper catalysis with a NHC ligand failed to yield the desired product (Table 1, entry 12). We sequentially optimized other conditions, including bases, reagent dosage, and temperature. Finally, we acquired the reaction conditions to give product 1a in 79% GC yield (Table 1, entry 13). Notably, most of the traditional double-carbonylation reactions require relatively high CO pressure to control the selectivity between monocarbonylation and double-carbonylation. Under the current optimized conditions, 10 bar CO is sufficient for a smooth transformation. Although we need an excess of amines to facilitate the reaction, a significant amount of amines could be recovered during the separation process. *cis*-β-Methylstyrene was also tested under the optimization conditions, and only 25% yield was detected.

**Table 1:** Studies on the reaction conditions

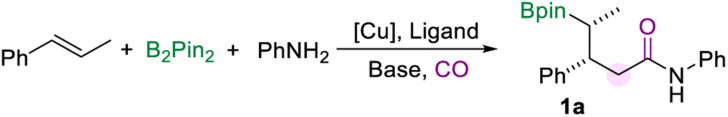				
Entry	Ligand	[Cu]	Solvent	Yield (%)^a^
1	DPPE	CuI	DMAc	24
2	DPPP	CuI	DMAc	11
3	DPPBz	CuI	DMAc	13
4	DPPE	CuI	DMF	21
5	DPPE	CuI	DMSO	68
6	DPPE	CuI	NMP	11
7	DPPE	CuI	Toluene	n.d.
8	DPPE	CuCl_2_	DMSO	54
9	DPPE	Cu(OTf)_2_	DMSO	13
10	DPPE	CuBr_2_	DMSO	10
11	DPPE	Cu(OAc)_2_	DMSO	69
12	DPPE	IPrCuCl	DMSO	Trace
13^b^	DPPE	Cu(OAc)_2_	DMSO	79

a Reaction conditions: *trans*-β-methylstyrene (0.2 mmol), [Cu] (5 mol%), ligand (5 mol%), NaO^*t*^Bu (2.5 equiv.), B_2_Pin_2_ (2.5 equiv.), PhNH_2_ (2.5 equiv.), solvent (1 mL), CO (10 bar), 60 °C, and 20 h. Yields are determined by GC with hexadecane as an internal standard.

b NaOEt (2.75 equiv.) and B_2_Pin_2_ (3.5 equiv.).

**Scheme 2:**
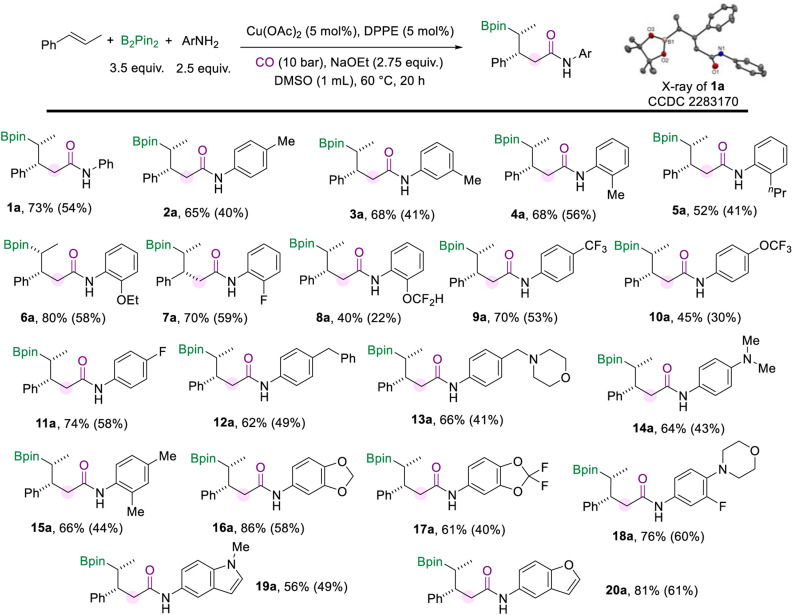
Reaction conditions for the scope of aryl amines. NMR yields are determined from the ^1^H NMR spectra of the crude reaction mixture using 1,3,5-trimethoxybenzene as the internal standard. Isolated yields are in parentheses (with 95% purify as the products are not stable and easy to decompose during the purification process and even in the NMR tube).

With the optimized protocol for the borylamidation in hand, we started to test the generality of the reaction by applying it to a range of arylamines (Scheme 2). Notably, the isolated yields of the γ-boryl amides were decreased after the purification process due to the instability of the alkyl boronate species. When the methyl group is substituted at the *para*-, *meta*-, or *ortho*-positions of the aniline, respectively, the corresponding amides were produced all in good yields and excellent stereoselectivity (2a–4a). The arylamines bearing electron-donating and electron-withdrawing groups at the *ortho*-position smoothly accessed amides in moderate to good yields, including *n*-propyl (5a), methoxy (6a), fluoride (7a), and difluoromethyl (8a) groups. Various aryl amines with a substituent at the *para* position were tested under the standard conditions. In the case of arylamines substituted with electron-withdrawing groups, the desired products (9a and 10a) were afforded in moderate to good yields. The *para*-fluorine substituted aniline produced the amide (11a) in 70% yield. In addition, substitutions such as benzyl, methyl morpholine, and dimethylamino at the *para* position of aniline can also produce the corresponding amides in good yields (12a–14a). This borylamidation protocol exhibits excellent compatibility for disubstituted anilines (16a–18a). For example, aniline with the piperonyl group could afford 16a in 86% yield. 3-Fluoro-4-morpholinoaniline which appeared as a pharmaceutical intermediate^17^ could be applied in this amidation reaction and afforded the relevant amide (18a) in 76% yield. Heterocyclic-based amines reacted well in the amidation process and afforded the desired amides that are modified with 1-methyl-1*H*-indole (19a) or benzofuran (20a) motifs in moderate to good yields. In all these amidation reactions of arylamines with *trans*-β-methylstyrene, the stereoselectivity of the amides was excellent (d.r. > 20 : 1).

**Scheme 3:**
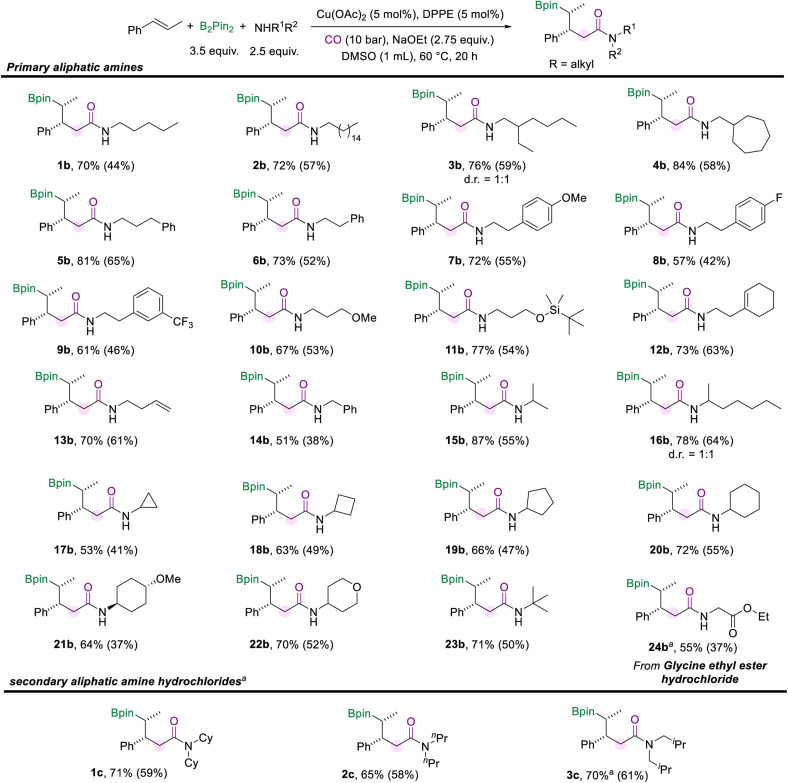
Reaction conditions for the scope of aliphatic amines. NMR yields are determined from the ^1^H NMR spectra of the crude reaction mixture using 1,3,5-trimethoxybenzene as the internal standard. Isolated yields are in parentheses (with 95% purify as the products are not stable and easy to decompose during the purification process and even in the NMR tube). [a] Cu(OAc)_2_ (10 mol%), DPPE (10 mol%), and the corresponding amine hydrochloride (1.5 equiv.).

Then we turned our attention to investigating the scope of aliphatic amines (Scheme 3). First, we used pentane-1-amine as the nucleophilic component to give the desired aliphatic amide (1b) in 70% NMR yield under the standard reaction conditions. Aliphatic amines for the amidation reaction can accommodate extended alkyl chains, and 2b was effectively obtained. When the branched carbon chain, cycloheptane, or benzene ring was present in the side chain of the aliphatic amines, the corresponding amides were obtained in good yields as well (3b–6b). Moreover, phenethylamine with different substituents (–OMe, –F, and –CF_3_) can also afford the desired products (7b–9b). Aliphatic amines with the methoxy or silyloxyl motifs installed on the carbon chain were also suitable substrates and produced the target amides (10b and 11b) in good yields. Unsaturated motifs, including the internal alkene and terminal alkene (12b and 13b), could be tolerated in this conversion rather than being involved in other competing reactions. The benzylamine was smoothly involved in the reaction and yielded the product 14b in 51% yield. Furthermore, when the amino group of the alkylamines was attached to a secondary carbon atom, the multi-component borylamidation reaction proceeded smoothly and generated the corresponding products in high yields (15b and 16b). Various cyclic amines were also suitable substrates in this amidation process. We obtained a series of amides (17b–22b) mounted with cyclopropane, cyclobutane, cyclopentane, cyclohexane, methoxycyclohexane, and tetrahydropyran structures in moderate to good yields (53–72%). In addition, when the amino group was attached to a tertiary carbon, 23b was still effectively obtained. Furthermore, the hydrochloride salt of glycine was successfully applied in this transformation and gave the desired amide 24b in 55% yield. However, only trace amounts of the desired amides were obtained when we attempted to employ secondary aliphatic amines in the reaction (For details, see the ESI†). Given the higher nucleophilicity of the secondary alkyl amines, the conduction of multi-component cascade borylamidation remains challenging. Interestingly, the yields of the corresponding amides were significantly improved when *sec*-alkylamine hydrochlorides were employed as nucleophiles. With a slight modification of the reaction conditions (for details, see the ESI†), several *sec*-alkylamine hydrochlorides were transformed successfully (Scheme 3). Experimental results showed that the steric hindrance of the substrate didn't affect the efficiency of the reaction. For example, the hindered hydrochloride salt of dicyclohexylamine delivered 1c in 71% NMR yield. Other hydrochloride salts of *sec*-alkylamines, such as dipropylamine and diisobutylamine, participated smoothly in this transformation and gave products (2c and 3c) with good yields as well. However, diphenylamine hydrochloride could only produce 26% of the desired product. Additionally, instead of amines, benzenethiol and cyclohexanethiol as sulfur nucleophiles were also tested under our standard conditions but no desired product was detected.

**Scheme 4:**
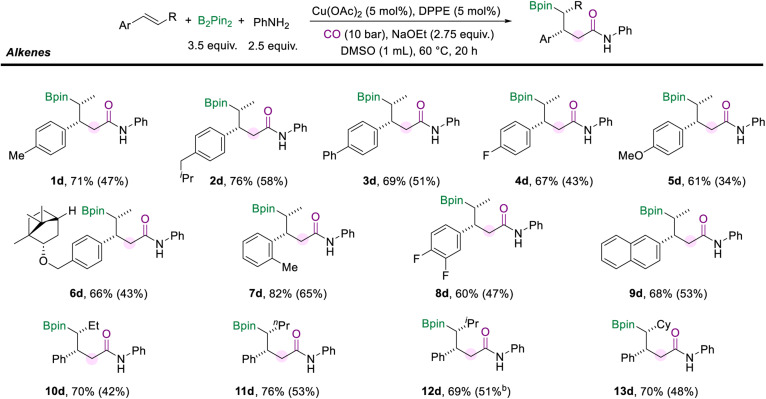
Reaction conditions for the scope of internal aryl alkenes. NMR yields are determined from the ^1^H NMR spectra of the crude reaction mixture using 1,3,5-trimethoxybenzene as the internal standard. Isolated yields are in parentheses (with 95% purify as the products are not stable and easy to decompose during the purification process and even in the NMR tube).

We also surveyed a range of alkenes to access the generality of this protocol (Scheme 4). Alkenes with a range of *para*-substituents were well-compatible under the standard conditions (1d–5d). Functional groups, including alkyl (methyl, isobutyl), phenyl, fluoro, and methoxy, were well tolerated. The multi-component cascade amidation worked exceptionally well for (−)-borneol-derived internal aryl olefin (6d, 66%). The position of the substituents on the aryl alkenes did not seem to affect the reaction performance. For example, *trans*-β-methylstyrene with *ortho*-methyl generated the desired amide (7d) in good yield and excellent diastereomeric ratios. And the reaction was found to proceed smoothly with the difluoro- and naphthalene-derived olefins as the substrates and delivered moderate yields of the corresponding products (8d and 9d). Importantly, the alkyl moiety of the internal olefins is not limited to the methyl group. When the alkenes were installed with ethyl or *n*-propyl fragments on the other side, the corresponding amides (10d and 11d) were successfully delivered with more than 70% yields. Moreover, the olefins modified with secondary alkyl substituents, such as isopropane and cyclohexane, did not hinder the reaction to give the desired products (12d, 69% and 13d, 70%). It is important to mention that the reaction failed when α-methylstyrene, stilbene, or aliphatic alkenes were tested.

**Scheme 5:**
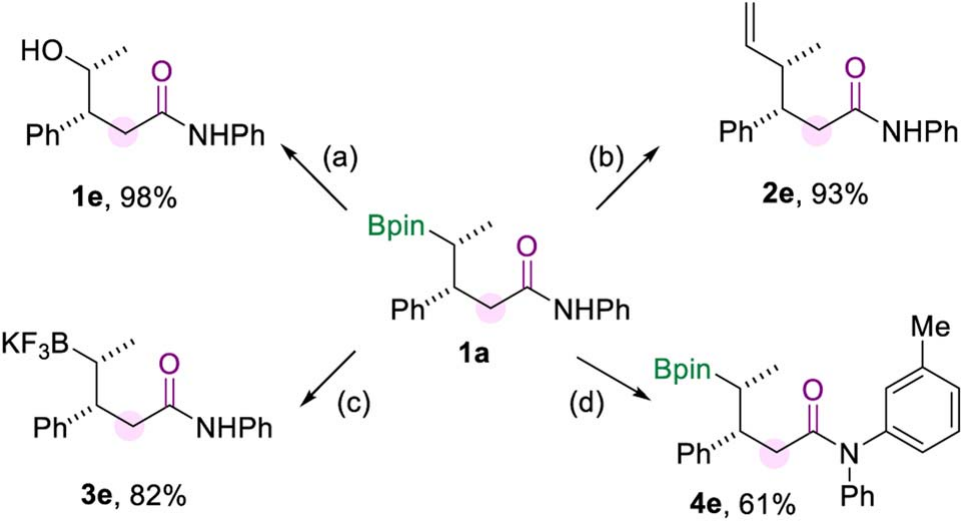
Synthetic transformations. [a] Oxidation of 1a. [b] Vinylation of 1a. [c] Preparation of the trifluoroboration salt of 1a. [d] *N*-arylation of 1a. The details for the reaction conditions are available in the ESI.†

To demonstrate the synthetic utility of this borylamidation, several transformations of model product 1a were carried out (Scheme 5), which prove the organoboron product to be a versatile synthetic reagent. Firstly, 1a could be oxidized to the corresponding alcohol 1e in excellent yield when using NaBO_3_·4H_2_O as the oxidizing agent. The vinylation of 1a was easily achieved through the reaction of the boryl group with the vinyl Grignard reagent to produce 2e in 93% yield. And the borylated amide 1a can be converted to its potassium trifluoroborate salt (3e). In addition, the amide group of 1a could be further modified by copper-catalyzed *N*-arylation with aryl iodide to produce 4e in 61% yield.

**Scheme 6:**
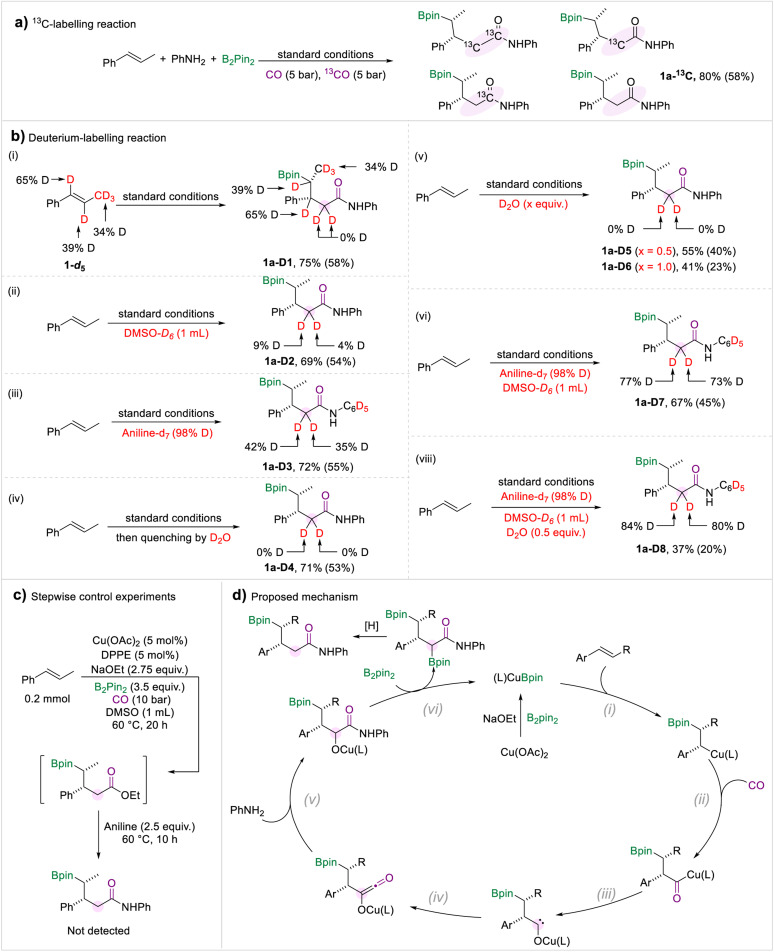
Mechanism studies and proposed mechanism. NMR yields are determined from the ^1^H NMR spectra of the crude reaction mixture using 1,3,5-trimethoxybenzene as the internal standard. Isolated yields are in parentheses.

A series of control experiments were performed to investigate the reaction mechanism (Scheme 6). The ^13^C-labelling experiment demonstrated that the methylene and carbonyl motifs converted from CO gas (Scheme 6a). Then we carried out a range of deuterium-labelling experiments to explore the provenance of the hydrogen atoms in the methylene group (Scheme 6b). Deuterated *trans*-β-methylstyrene was prepared and subjected to the borylamidation process. The amide 1a-D1 was obtained in which no hydrogen atom transfer was observed. When the solvent was changed to DMSO-*d*_6_, we only detected a trace substitution of deuterium in the target amide 1a-D2. In addition, when we submitted the aniline-*d*_7_ to the standard conditions, the two hydrogen atoms attached to the methylene group were deuterated by 42% and 35%, respectively (1a-D3), which implied that one proton derived from aniline. After completion of the borylamidation process, the reaction was quenched with D_2_O, and the methylene motif in 1a-D4 was not deuterated. Due to the hygroscopic nature of DMSO and NaOEt, traces of water in the reaction system may be a proton source. Thus, we carried out the reaction under standard conditions with additional D_2_O. We did not detect any significant deuterium substitution in the target amides, whether 0.5 equivalent of D_2_O was added or the amount was increased to 1.0 equivalent (Scheme 6b(v)). Afterwards, a borylamidation process involving multiple deuterated reagents was carried out to study the hydrogen source of the methylene. When aniline-*d*_7_ and DMSO-*d*_6_ were used under standard conditions, the deuteration ratio of 1a-D7 was significantly increased. Compared with the deuterium-labelling experiments of 1a-D2 and 1a-D3, we proposed that the H-D exchange process occurred between the aniline and DMSO. In addition, we performed the reaction using aniline-*d*_7_, DMSO-*d*_6_, and 0.5 equivalent of D_2_O. We obtained the desired amide 1a-D8 with a higher deuteration ratio compared with 1a-D7. Therefore, we conclude that the two protons came from the amine, DMSO, and trace amount of water. Under the standard conditions, there is a H/D exchange process between the amine, DMSO, and water. To investigate whether the amides were produced *via* the aminolysis process of the possible ester intermediates with amines, a stepwise experiment was carried out (Scheme 6c). We can't detect the amide product in the stepwise control experiment.

Based on the experiment results and our previous studies, a mechanism for this multi-component borylamidation reaction was proposed (Scheme 6d). Initially, the copper catalyst was converted to activated (L)CuBpin species in the presence of NaOEt and B_2_pin_2_. Then migratory insertion of (L)CuBpin and olefins occurred (step i). The formed alkyl-copper intermediate afforded the acyl-copper species under a CO atmosphere (step ii). Then the acyl-copper species will isomerize to a carbene intermediate (step iii). Under the optimized amidation conditions, the carbene motif was selectively reacted with a CO molecule to form the ketene intermediate (step iv). Subsequently, the reaction of the ketene with an amine generated the amide intermediate (step v). Through the subsequent reaction with B_2_pin_2_, the OBpin group was formed which was considered as a leaving group (step vi).^18^ The final desired amides were produced through a protonation process.

## Conclusions

In summary, we have developed a multi-competent borocarbonylation for the synthesis of γ-boryl amides utilizing readily available starting reagents. With Cu(OAc)_2_/DPPE as the catalyst system and DMSO as the solvent, a wide range of γ-boryl amides were obtained in good yields with excellent d.r. ratios under mild conditions. This transformation exhibits broad substrate tolerance. Both arylamines, aliphatic amines, and hydrochloride salts of secondary amines were involved in this transformation to produce the desired γ-boryl amides. Moreover, this method serves as an attractive method for the synthesis of complex amides. The γ-boryl amides are versatile synthetic materials, and several transformations were performed to show their synthetic values. The ^13^C-labelling experiment demonstrates that the methylene and carbonyl groups in the target amides were all derived from CO.

## Data availability

All data related with this research are provided in ESI.†

## Author contributions

XFW directed this project, revised the manuscript. HQG performed most of the experiments, prepared the ESI† and draft. YHZ and PY prepared some of the starting materials.

## Conflicts of interest

There are no conflicts to declare.

## Supplementary Material

SC-015-D4SC00156G-s001

SC-015-D4SC00156G-s002
