# Supplementary material for: Copper-catalyzed carbonylative multi-component borylamidation of alkenes for synthesizing γ-boryl amides with CO as both methylene and carbonyl sources
Source: Chem Sci. 2024 Feb 5;15(11):3996–4004. doi: 10.1039/d4sc00156g (PMC10935720; doi:10.1039/d4sc00156g)

Electronic Supplementary Material (ESI) for Chemical Science.
This journal is © The Royal Society of Chemistry 2024

Supporting Information

Copper-Catalyzed Carbonylative Multi-Component Borylamidation of Alkenes for Synthesizing γ-Boryl Amides with CO as both Methylene and Carbonyl Sources

Hui-Qing Geng,[a] Yan-Hua Zhao,[a] Peng Yang[a] and Xiao-Feng Wu* [a,b]

[a] Leibniz-Institut für Katalyse e.V., Albert-Einstein-Straße 29a, 18059 Rostock (Germany).

[b] Dalian National Laboratory for Clean Energy, Dalian Institute of Chemical Physics, Chinese Academy of Sciences, 116023, Dalian, Liaoning, China, E-mail: xwu2020@dicp.ac.cn

Table of Contents

1. Genernal information S2
2. Optimization of reaction conditions. S2
3. General procedure. S6
   3.1 General procedure for primary amines. S6
   3.2 General procedure for secondary amines-hydrochloride. S7
   3.3 Failed examples. S7
4. Characterization Data. S8
5. Derivatization of 1a. S31
   5.1 Oxidation of **1a**. S31
   5.2 Vinylation of **1a**. S32
   5.3 Preparation of trifluoroboration salt of **1a.** S32
   5.4 *N*-Arylation of **1a**. S33
6. Mechanism studies. S33
   6.1 ^{13}C labeling experiment. S33
   6.2 Deuterium labeling experiment. S36
      6.2.1 Deuterated *trans*-*β*-methylstyrene. S36
      6.2.2 DMSO-d_6. .... S39
      6.2.3 Aniline-d_7. .... S42
      6.2.4 Quenching with D_2O. S45
      6.2.5 Additional D_2O. S48
      6.2.6 Mixed deuterated reagents of aniline-d_7 and DMSO-d_6. .... S52
      6.2.7 Mixed deuterated reagents of aniline-d_7, DMSO-d_6, and D_2O. S55
7. X-ray crystal analysis of 1a. S57
8. References. S60
9. NMR spectra S61

1. General information

All experiments were carried out under a carbon monoxide or nitrogen atmosphere, unless otherwise stated. All chemical reagents were purchased from Sigma-Aldrich, TCI, abcr GmbH Germany, and BLD pharm of the highest purity grade and used without purification. In addition, B_2Pin_2 and NAOEt are stored in the glove box. Internal aryl olefins were prepared according to previous references. All solvents were dried by standard techniques. Silica gel column chromatography was carried out using silica gel (200–300 mesh). *n*-Pentane and ethyl acetate were used as eluent. Analytical thin layer chromatography (TLC) was performed on silica gel (silica gel 60 DC-Platten ALUGRAM® Xtra SIL G / UV_{254}). TLC plates were visualized with UV light, and/or submersion in $KMnO_4$ solution and/or phosphomolybdic acid. Bruker Avance III HD 300 NMR (^{1}H, 300 MHz; ^{13}C{^{1}H}, 75 MHz; ^{11}B, 96 MHz), and Bruker ARX 400 NMR spectrometers (^{1}H, 400 MHz; ^{13}C{^{1}H}, 101 MHz) recorded all NMR spectra. The chemical shifts are reported as δ values relative to internal chloroform (δ 7.26 for ^{1}H NMR) and TMS (δ 0.00 for ^{1}H NMR) chloroform (δ 77.16 for ^{13}C{^{1}H} NMR) in parts per million (ppm). The following abbreviations are used for the NMR spectra' multiplicities: s: singlet, d: doublet, dd: doublet of doublet, t: triplet, q: quartet, m: multiplet, and br: broad signal for proton spectra. All ^{13}C NMR spectra were broad-band ^{1}H decoupled. However, it is hard to observe the signals for the carbon attached to boron in the ^{13}C{^{1}H} NMR spectra. Gas chromatography (GC) analyses were performed on an Agilent HP-7890A instrument with an FID detector and HP-5 capillary column (polydimethylsiloxane with 5% phenyl groups, 30 m, 0.32 mm i.d. 0.25 μm film thickness) using argon as carrier gas. High-resolution mass spectra were recorded on an Agilent 6210 system. All the reactions which used CO were performed in an autoclave. The laboratory should be well-equipped with a CO detector and alarm system.

2. Optimization of reaction conditions.

***Table S1*. Screening of ligand**

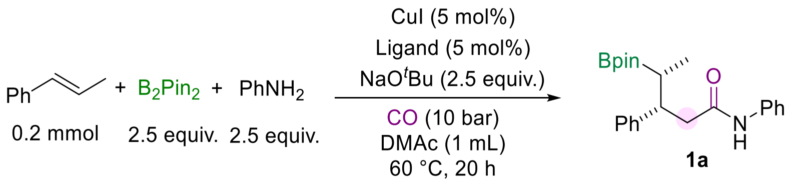

Entry	Base	**1a (%)**
1	DPPE	24
2	DPPB	8
3	DPPP	11
4	DPPPe	n.d.
5	DPPBz	13
6	Xantphos	n.d.
7	DPPM	n.d.

Reaction conditions: (*E*)-prop-1-en-1-ylbenzene (0.2 mmol), CuI (5 mol%), Ligand (5 mol%), NaO^{t}Bu (2.5 equiv.), B_2Pin_2 (2.5 equiv.), $PhNH_2$ (2.5 equiv.), DMAc (1 mL), CO (10 bar), 60 °C, 20 h; Yields are determined by GC with hexadecane as an internal standard; n.d.: not detected.

***Table S2*. Screening of solvent**

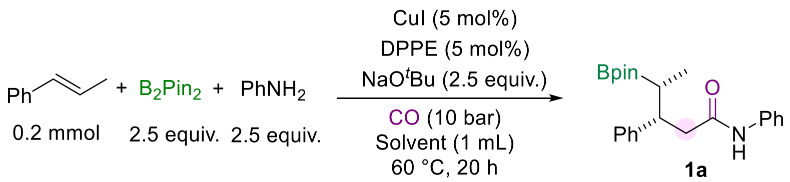

Entry	Solvent	**1a (%)**
1	DMF	21
2	DMSO	68
3	NMP	11
4	Toluene	n.d.
5	THF	33
6	CH_3CN	n.d.

Reaction conditions: (*E*)-prop-1-en-1-ylbenzene (0.2 mmol), CuI (5 mol%), DPPE (5 mol%), NaO^{t}Bu (2.5 equiv.), B_2Pin_2 (2.5 equiv.), $PhNH_2$ (2.5 equiv.), solvent (1 mL), CO (10 bar), 60 °C, 20 h; Yields are determined by GC with hexadecane as an internal standard; n.d.: not detected.

***Table S3*. Screening of copper catalyst**

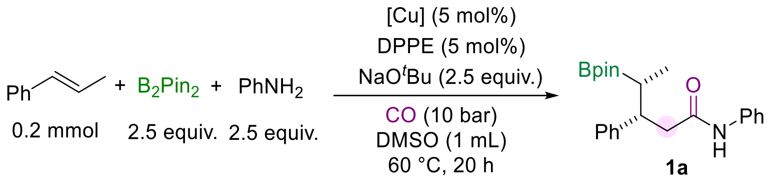

Entry	Solvent	**1a (%)**
1	$CuCl_2$	54
2	$Cu(OTf)_2$	13
3	CuCN	4
4	$CuSO_4$	n.d.
5	CuI	68
6	$CuBr_2$	10
7	$CuBr•Me_2S$	Trace
8	CuBr	Trace
9	$Cu(OAc)_2$	69
10	IPrCuCl	Trace
11	IPrCuCl (w/o DPPE)	Trace
12	IMesCuCl	6
13	IMesCuCl (w/o DPPE)	Trace

Reaction conditions: (*E*)-prop-1-en-1-ylbenzene (0.2 mmol), [Cu] (5 mol%), DPPE (5 mol%), NaO^{t}Bu (2.5 equiv.), B_2Pin_2 (2.5 equiv.), $PhNH_2$ (2.5 equiv.), DMSO (1 mL), CO (10 bar), 60 °C, 20 h; Yields are determined by GC with hexadecane as an internal standard; n.d.: not detected.

***Table S4*. Screening of the base**

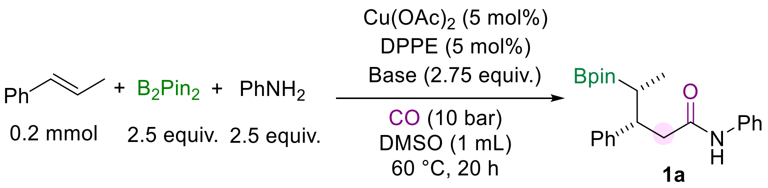

Entry	Base	**1a (%)**
1	NaO^{t}Bu	69
2	LiO^{t}Bu	41
3	KO^{t}Bu	Trace
4	NaOEt	74
5	Cs_2CO_3	11
6	LiOMe	Trace
7	KOMe	17

Reaction conditions: (*E*)-prop-1-en-1-ylbenzene (0.2 mmol), $Cu(OAc)_2$ (5 mol%), DPPE (5 mol%), base (2.5 equiv.), B_2Pin_2 (2.5 equiv.), $PhNH_2$ (2.5 equiv.), DMSO (1 mL), CO (10 bar), 60 °C, 20 h; Yields are determined by GC with hexadecane as an internal standard.

***Table S5*. Screening of the amount of catalytic system**

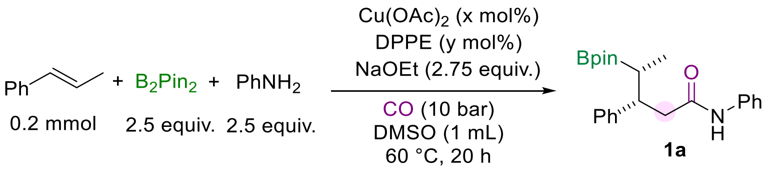

Entry	$Cu(OAc)_2$	DPPE	**1a (%)**
1	2.5 mol%	2.5 mol%	66
2	5 mol%	5 mol%	74
3	7.5 mol%	7.5 mol%	56
4	10 mol%	10 mol%	60
5	5 mol%	7.5 mol%	49
6	5 mol%	10 mol%	32

Reaction conditions: (*E*)-prop-1-en-1-ylbenzene (0.2 mmol), $Cu(OAc)_2$ (x mol%), DPPE (y mol%), NaOEt (2.5 equiv.), B_2Pin_2 (2.5 equiv.), $PhNH_2$ (2.5 equiv.), DMSO (1 mL), CO (10 bar), 60 °C, 20 h; Yields are determined by GC with hexadecane as an internal standard.

***Table S6*. Screening of the amount of B_2pin_2**

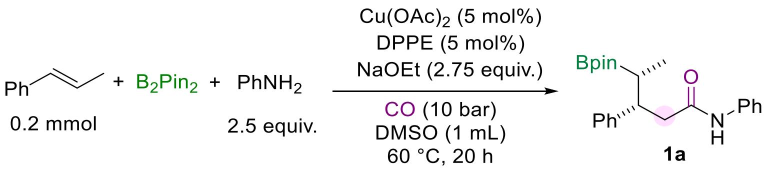

Entry	B_2pin_2	**1a (%)**
1	3.0 eq.	71
2	3.5 eq.	79
3	3.75 eq.	73
4	4.0 eq.	68

Reaction conditions: (*E*)-prop-1-en-1-ylbenzene (0.2 mmol), $Cu(OAc)_2$ (5 mol%), DPPE (5 mol%), NaOEt (2.5 equiv.), B_2Pin_2 (x equiv.), $PhNH_2$ (2.5 equiv.), DMSO (1 mL), CO (10 bar), 60 °C, 20 h; Yields are determined by GC with hexadecane as an internal standard.

***Table S7*. Screening of the amount of $PhNH_2$**

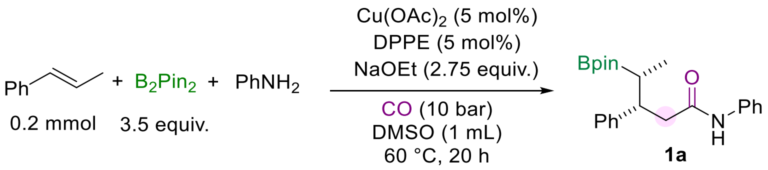

Entry	$PhNH_2$	**1a (%)**
1	1.5 eq.	64
2	1.75 eq	70
3	2.0 eq.	75
4	2.5 eq.	79
5	2.75 eq.	74

Reaction conditions: (*E*)-prop-1-en-1-ylbenzene (0.2 mmol), $Cu(OAc)_2$ (5 mol%), DPPE (5 mol%), NaOEt (2.5 equiv.), B_2Pin_2 (3.5 equiv.), $PhNH_2$ (x equiv.), DMSO (1 mL), CO (10 bar), 60 °C, 20 h; Yields are determined by GC with hexadecane as an internal standard.

***Table S8*. Screening of the temperature**

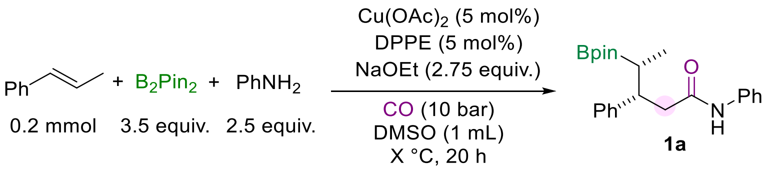

Entry	Temperature	**1a (%)**
1	r.t.	38
2	80 °C	44
3	100 °C	25

Reaction conditions: (*E*)-prop-1-en-1-ylbenzene (0.2 mmol), $Cu(OAc)_2$ (5 mol%), DPPE (5 mol%), NaOEt (2.75 equiv.), B_2Pin_2 (3.5 equiv.), $PhNH_2$ (2.5 equiv.), DMSO (1 mL), CO (10 bar), x °C, 20 h; Yields are determined by GC with hexadecane as an internal standard.

***Table S9*. Optimization the reaction conditions for secondary amines.**

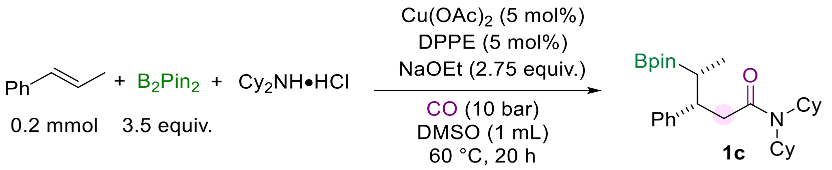

Entry	Deviation from the above conditions	**1a (%)**
1	Cy_2NH (2.5 equiv.)	19
2	Cy_2NH•HCl (2.5 equiv.)	46
3	Cy_2NH•HCl (2.0 equiv.)	59
4	Cy_2NH•HCl (1.5 equiv.)	65
5	Cy_2NH•HCl (1.0 equiv.)	47
6[b]	Cy_2NH•HCl (1.5 equiv.)	71

Reaction conditions: (*E*)-prop-1-en-1-ylbenzene (0.2 mmol), $Cu(OAc)_2$ (5 mol%), DPPE (5 mol%), NaOEt (2.75 equiv.), B_2Pin_2 (3.5 equiv.), amines (x equiv.), DMSO (1 mL), CO (10 bar), 60 °C, 20 h; yields are determined by ^1H NMR spectra of the crude reaction mixture. [b] $Cu(OAc)_2$ (10 mol%), DPPE (10 mol%)

3. General procedure.

3.1 General procedure **A** for primary amines.

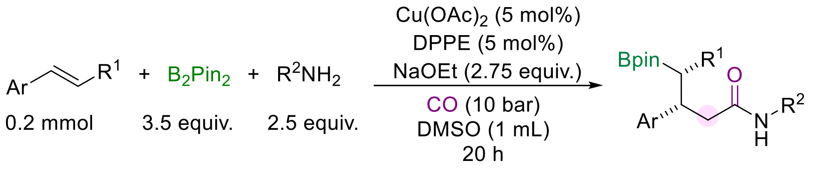

A dried vial (4 mL) was charged with $Cu(OAc)_2$ (5 mol%), DPPE (5 mol%), B_2pin_2 (3.5 equiv.), and a stirring bar. When the NaOEt (2.75 equiv.) is added, the vial was sealed by PTFE/white rubber septum (Wheaton 13 mm Septa) and phenolic cap immediately. The vial was evacuated under vacuum and recharged with argon for three times with a needle. Then, internal aryl olefins (0.2 mmol), aryl amines or alkyl amines (2.5 equiv.) and DMSO (1.0 mL) were added under argon by using a syringe. The vial (or several vials) was placed in an alloy plate and transferred into a 300 mL autoclave of the 4560 series from Parr Instruments. The autoclave was charged with CO (10 bar) after flushing two times with N_2 and two times with CO. The autoclave was then placed into an aluminum block on a magnetic stirrer. The reaction was performed for 20 h at 60 °C. After the reaction was complete, the autoclave was cooled down to room temperature and the pressure was released carefully. The reaction was quenched with water (2 mL) and extracted with EtOAc (2 mLx3). The organic layer was washed with water (10 mL) and dried over anhydrous Na_2SO_4 and concentrated under reduced pressure. After that the corresponding products was afforded by directly purified by fast column chromatography. (For NMR yield: The

organic layer was washed with water and 0.1 mmol of 1,3,5-trimethoxybenzene was added. After drying with anhydrous Na_2SO_4, an appropriate amount of organic layer was concentrated under reduced pressure to test the NMR yield.)

3.2 General procedure B for secondary amines-hydrochloride.

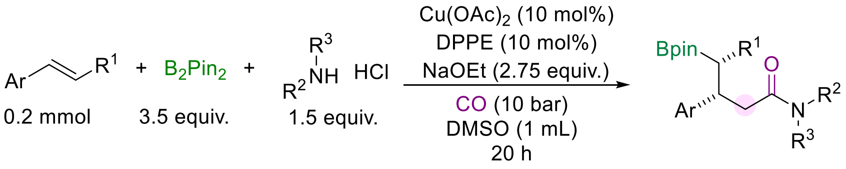

A dried vial (4 mL) was charged with $Cu(OAc)_2$ (10 mol%), DPPE (10 mol%), B_2pin_2 (3.5 equiv.), and a stirring bar. When the NaOEt (2.75 equiv.) is added, the vial was sealed by PTFE/white rubber septum (Wheaton 13 mm Septa) and phenolic cap immediately. The vial was evacuated under vacuum and recharged with argon for three times with a needle. Then, internal aryl olefins (0.2 mmol), secondary amines hydrochloride (1.5 equiv.) and DMSO (1.0 mL) were added under argon by using a syringe. The vial (or several vials) was placed in an alloy plate and transferred into a 300 mL autoclave of the 4560 series from Parr Instruments. The autoclave was charged with CO (10 bar) after flushing two times with N_2 and two times with CO. The autoclave was then placed into an aluminum block on a magnetic stirrer. The reaction was performed for 20 h at 60 °C. After the reaction was complete, the autoclave was cooled down to room temperature and the pressure was released carefully. The reaction was quenched with water (2 mL) and extracted with EtOAc (2 mLx3). The organic layer was washed with water (10 mL) and dried over anhydrous Na_2SO_4 and concentrated under reduced pressure. After that the corresponding products was afforded by directly purified by fast column chromatography. (For NMR yield: The organic layer was washed with water and 0.1 mmol of 1,3,5-trimethoxybenzene was added. After drying with anhydrous Na_2SO_4, an appropriate amount of organic layer was concentrated under reduced pressure to test the NMR yield.)

3.3 Failed examples.

Failed examples of amines

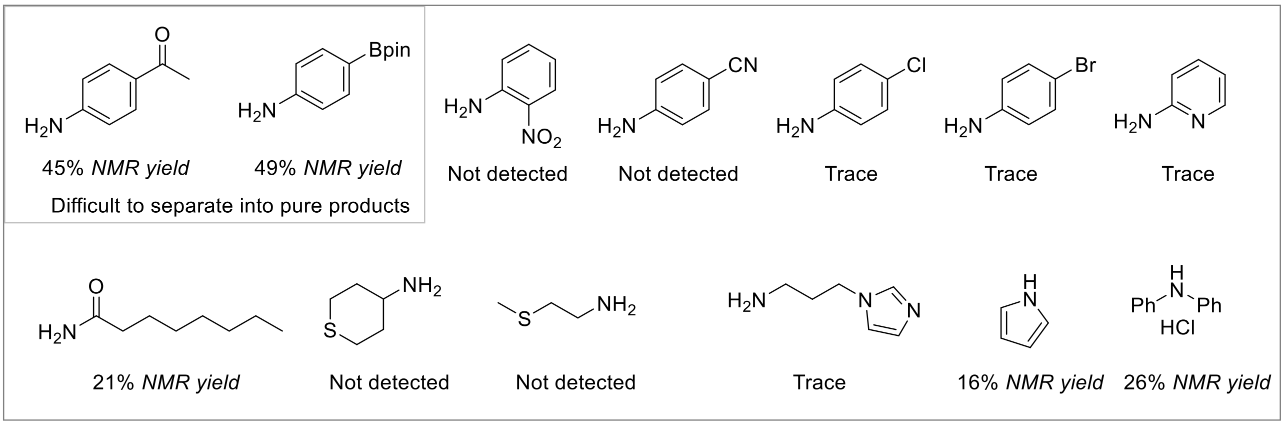

Failed examples of alkenes

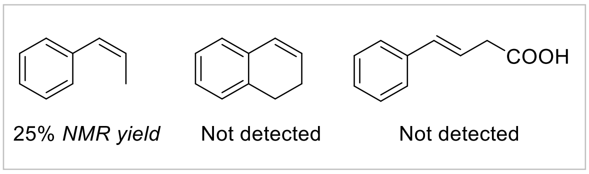

Failed examples of boronate ester

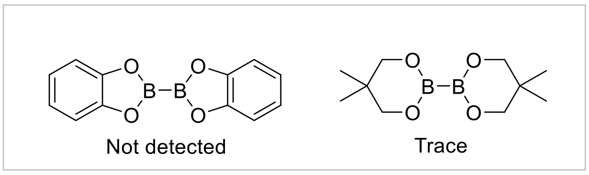

4. Characterization Data.

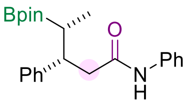

N,3-Diphenyl-4-(4,4,5,5-tetramethyl-1,3,2-dioxaborolan-2-yl)pentanamide (1a)

The title compound was prepared from *trans-β*-methylstyrene (0.2 mmol, 26 μL) and aniline (2.5 equiv., 45 μL), according to general procedure. The crude residue was purified by flash chromatography (*n*-pentane/EA = 10:1, Rf = 0.2) to give the product as a white solid (41 mg, 54%).

^{1}H NMR (300 MHz, $CDCl_3$) δ 7.33 – 7.27 (m, 2H), 7.26 – 7.16 (m, 7H), 7.10 – 6.91 (m, 2H), 3.16 (td, *J* = 10.2, 4.1 Hz, 1H), 2.80 (dd, *J* = 14.0, 4.1 Hz, 1H), 2.62 (dd, *J* = 14.0, 10.3 Hz, 1H), 1.53 – 1.40 (m, 1H), 1.29 (d, *J* = 1.6 Hz, 12H), 0.81 (d, *J* = 7.4 Hz, 3H).

^{13}C NMR (75 MHz, $CDCl_3$) δ 170.0, 143.7, 137.8, 128.7, 128.6, 127.8, 126.6, 123.9, 119.7, 83.4, 45.7, 45.3, 24.9, 24.6, 14.0.

^{11}B NMR (96 MHz, $CDCl_3$) δ 33.4.

HRMS (ESI-TOF): calcd for $[M+Na]^+$ $C_{23}H_{30}{}^{11}BNO_3$ 402.2215, found: 402.2221.

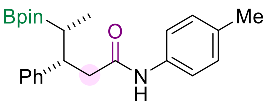

3-Phenyl-4-(4,4,5,5-tetramethyl-1,3,2-dioxaborolan-2-yl)-N-(p-tolyl)pentanamide (2a)

The title compound was prepared from *trans-β*-methylstyrene (0.2 mmol, 26 μL) and *p*-toluidine (2.5 equiv., 54 mg), according to general procedure. The crude residue was purified by flash chromatography (*n*-pentane/EA = 10:1, Rf = 0.2) to give the product as a white solid (31 mg, 40%).

^{1}H NMR (300 MHz, $CDCl_3$) δ 7.34 – 7.21 (m, 5H), 7.17 – 7.00 (m, 4H), 6.93 (s, 1H), 3.17 (td, *J* = 10.2, 4.1 Hz, 1H), 2.81 (dd, *J* = 14.0, 4.1 Hz, 1H), 2.62 (dd, *J* = 14.0, 10.3 Hz, 1H), 2.28 (s, 3H), 1.52 – 1.43 (m, 1H), 1.31 (d, *J* = 1.2 Hz, 12H), 0.83 (d, *J* = 7.4 Hz, 3H).

^{13}C NMR (75 MHz, $CDCl_3$) δ 169.9, 143.7, 135.2, 133.5, 129.2, 128.6, 127.9, 126.6, 119.8, 83.4, 45.8, 45.3, 24.9, 24.7, 20.8, 14.0.

^{11}B NMR (96 MHz, $CDCl_3$) δ 34.3

HRMS (ESI-TOF): calcd for $[M+Na]^+$ $C_{24}H_{32}{}^{10}BNO_3$ 415.2404, found: 415.2404.

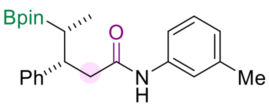

3-Phenyl-4-(4,4,5,5-tetramethyl-1,3,2-dioxaborolan-2-yl)-N-(m-tolyl)pentanamide (3a)

The title compound was prepared from *trans-β*-methylstyrene (0.2 mmol, 26 μL) and *m*-toluidine (2.5 equiv., 54 mg), according to general procedure. The crude residue was purified by flash chromatography (*n*-pentane/EA = 10:1, Rf = 0.2) to give the product as a white solid (32 mg, 41%).

^{1}H NMR (300 MHz, $CDCl_3$) δ 7.34 – 7.27 (m, 2H), 7.25 – 7.20 (m, 3H), 7.14 (s, 1H), 7.10 (t, *J* = 7.8 Hz, 1H), 6.95 (d, *J* = 12.5 Hz, 2H), 6.84 (d, *J* = 7.4 Hz, 1H), 3.15 (td, *J* = 10.2, 4.1 Hz, 1H), 2.79 (dd, *J* = 14.0, 4.1 Hz, 1H), 2.60 (dd, *J* = 14.0, 10.3 Hz, 1H), 2.27 (s, 3H), 1.50 – 1.40 (m, 1H), 1.29 (d, *J* = 1.8 Hz, 12H), 0.81 (d, *J* = 7.4 Hz, 3H).

^{13}C NMR (75 MHz, $CDCl_3$) δ 169.9, 143.7, 138.7, 137.7, 128.6, 128.5, 127.9, 126.6, 124.7, 120.4, 116.7, 83.4, 45.7, 45.4, 24.9, 24.6, 21.4, 14.0.

^{11}B NMR (96 MHz, $CDCl_3$) δ 32.9.

HRMS (ESI-TOF): calcd for $[M+Na]^+$ $C_{24}H_{32}{}^{10}BNO_3$ 415.2404, found: 415.2406.

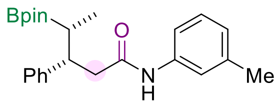

3-Phenyl-4-(4,4,5,5-tetramethyl-1,3,2-dioxaborolan-2-yl)-*N*-(*o*-tolyl)pentanamide (4a)

The title compound was prepared from *trans*-*β*-methylstyrene (0.2 mmol, 26 μL) and *o*-toluidine (2.5 equiv., 54 mg), according to general procedure. The crude residue was purified by flash chromatography (*n*-pentane/EA = 10:1, Rf = 0.2) to give the product as a white solid (44 mg, 56%).

^1H NMR (300 MHz, $CDCl_3$) δ 7.56 (d, *J* = 8.0 Hz, 1H), 7.34 – 7.26 (m, 3H), 7.25 – 7.17 (m, 2H), 7.13 – 6.93 (m, 3H), 6.67 (s, 1H), 3.18 (td, *J* = 10.6, 3.9 Hz, 1H), 2.85 (dd, *J* = 14.1, 3.7 Hz, 1H), 2.73 – 2.62 (m, 1H), 1.82 (s, 3H), 1.42 (t, *J* = 8.5 Hz, 1H), 1.28 (d, *J* = 1.8 Hz, 12H), 0.80 (d, *J* = 7.4 Hz, 3H).

^{13}C NMR (75 MHz, $CDCl_3$) δ 170.0, 143.5, 135.6, 130.2, 129.0, 128.7, 128.0, 126.7, 126.5, 124.9, 123.1, 83.4, 45.7, 45.0, 24.9, 24.6, 17.3, 13.9.

^{11}B NMR (96 MHz, $CDCl_3$) δ 33.8.

HRMS (ESI-TOF): calcd for $[M+Na]^+$ $C_{24}H_{32}{}^{10}BNO_3$ 415.2404, found: 415.2408.

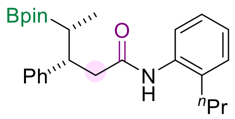

3-Phenyl-*N*-(2-propylphenyl)-4-(4,4,5,5-tetramethyl-1,3,2-dioxaborolan-2-yl)pentanamide (5a)

The title compound was prepared from *trans*-*β*-methylstyrene (0.2 mmol, 26 μL) and 2-propylaniline (2.5 equiv., 68 mg), according to general procedure. The crude residue was purified by flash chromatography (*n*-pentane/EA = 10:1, Rf = 0.2) to give the product as a white solid (35 mg, 41%).

^1H NMR (300 MHz, $CDCl_3$) δ 7.59 (d, *J* = 7.8 Hz, 1H), 7.35 – 7.22 (m, 5H), 7.18 – 7.03 (m, 3H), 6.74 (s, 1H), 3.22 (td, *J* = 10.6, 3.7 Hz, 1H), 2.88 (dd, *J* = 14.0, 3.7 Hz, 1H), 2.73 – 2.59 (m, 1H), 2.16 (dq, *J* = 15.8, 7.6 Hz, 2H), 1.43 (q, *J* = 7.4 Hz, 3H), 1.32 – 1.27 (m, 12H), 0.92 – 0.81 (m, 6H).

^{13}C NMR (75 MHz, $CDCl_3$) δ 170.1, 143.5, 135.0, 133.4, 129.2, 128.7, 127.9, 126.7, 126.4, 125.0, 123.8, 83.4, 45.6, 45.1, 32.8, 24.9, 24.6, 22.6, 13.9, 13.9.

^{11}B NMR (96 MHz, $CDCl_3$) δ 34.2.

HRMS (ESI-TOF): calcd for $[M+H]^+$ $C_{26}H_{36}{}^{11}BNO_3$ 422.2866, found: 422.2866.

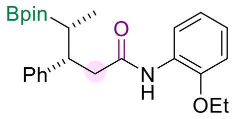

***N*-(2-Ethoxyphenyl)-3-phenyl-4-(4,4,5,5-tetramethyl-1,3,2-dioxaborolan-2-yl)pentanamide (6a)**

The title compound was prepared from *trans*-*β*-methylstyrene (0.2 mmol, 26 μL) and 2-ethoxyaniline (2.5 equiv., 69 mg), according to general procedure. The crude residue was purified by flash chromatography (*n*-pentane/EA = 5:1, Rf = 0.2) to give the product as a white solid (49 mg, 58%).

^{1}H NMR (300 MHz, $CDCl_3$) δ 8.24 (dd, *J* = 8.0, 1.4 Hz, 1H), 7.54 (s, 1H), 7.25 (d, *J* = 5.8 Hz, 4H), 7.18 – 7.11 (m, 1H), 6.89 (dtd, *J* = 24.2, 7.6, 1.6 Hz, 2H), 6.77 (dd, *J* = 8.0, 1.5 Hz, 1H), 4.00 (qd, *J* = 7.0, 4.1 Hz, 2H), 3.22 (td, *J* = 10.1, 4.4 Hz, 1H), 2.86 (dd, *J* = 14.3, 4.4 Hz, 1H), 2.66 (dd, *J* = 14.4, 10.4 Hz, 1H), 1.51 – 1.41 (m, 1H), 1.39 (t, *J* = 7.0 Hz, 3H), 1.26 (d, *J* = 2.0 Hz, 12H), 0.80 (d, *J* = 7.4 Hz, 3H).

^{13}C NMR (75 MHz, $CDCl_3$) δ 169.7, 146.9, 143.6, 137.0, 128.3, 127.9, 126.3, 123.2, 120.8, 119.8, 110.8, 83.3, 64.0, 45.2, 45.1, 25.0, 24.9, 24.6, 14.8, 13.8.

^{11}B NMR (96 MHz, $CDCl_3$) δ 30.1.

HRMS (ESI-TOF): calcd for $[M+Na]^+$ $C_{25}H_{34}{}^{10}BNO_3$ 445.2509, found: 445.2511.

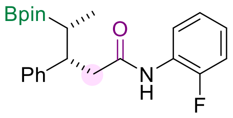

N-(2-Fluorophenyl)-3-phenyl-4-(4,4,5,5-tetramethyl-1,3,2-dioxaborolan-2-yl)pentanamide (7a)

The title compound was prepared from *trans-β*-methylstyrene (0.2 mmol, 26 μL) and 2-fluoroaniline (2.5 equiv., 55.5 mg), according to general procedure. The crude residue was purified by flash chromatography (*n*-pentane/EA = 10:1, Rf = 0.2) to give the product as a white solid (46 mg, 59%).

^{1}H NMR (400 MHz, $CDCl_3$) δ 8.20 (t, *J* = 7.8 Hz, 1H), 7.35 (s, 1H), 7.32 – 7.28 (m, 2H), 7.26 – 7.18 (m, 3H), 7.11 – 6.95 (m, 3H), 3.20 (td, *J* = 10.1, 4.1 Hz, 1H), 2.88 (dd, *J* = 14.2, 4.1 Hz, 1H), 2.70 (dd, *J* = 14.2, 10.2 Hz, 1H), 1.51 – 1.43 (m, 1H), 1.30 (d, *J* = 2.3 Hz, 12H), 0.83 (d, *J* = 7.5 Hz, 3H).

^{13}C NMR (101 MHz, $CDCl_3$) δ 170.1, 152.1 (d, *J* = 243.0 Hz), 143.4, 128.5, 127.7, 126.5, 126.4 (d, *J* = 10.2 Hz), 124.3 (d, *J* = 3.6 Hz), 123.8 (d, *J* = 7.5 Hz), 121.6, 114.5 (d, *J* = 19.2 Hz), 83.4, 45.6, 45.2, 24.8, 24.6, 14.0.

^{11}B NMR (128 MHz, $CDCl_3$) δ 35.3.

^{19}F NMR (282 MHz, $CDCl_3$) δ -131.2.

HRMS (ESI-TOF): calcd for $[M+H]^+$ $C_{23}H_{29}{}^{10}BFNO_3$ 397.2334, found: 397.2337.

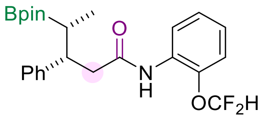

N-(2-(Difluoromethoxy)phenyl)-3-phenyl-4-(4,4,5,5-tetramethyl-1,3,2-dioxaborolan-2-yl)pentanamide (8a)

The title compound was prepared from *trans-β*-methylstyrene (0.2 mmol, 26 μL) and 2-(difluoromethoxy)aniline (2.5 equiv., 32 mg), according to general procedure. The crude residue was purified by flash chromatography (*n*-pentane/EA = 10:1, Rf = 0.2) to give the product as a white solid (20 mg, 22%).

^{1}H NMR (300 MHz, $CDCl_3$) δ 8.15 (d, *J* = 8.1 Hz, 1H), 7.28 (s, 1H), 7.22 – 7.16 (m, 3H), 7.15 – 6.99 (m, 3H), 6.96 – 6.88 (m, 2H), 6.24 (t, *J* = 73.6 Hz, 1H), 3.11 (td, *J* = 10.3, 4.2 Hz, 1H), 2.80 (dd, *J* = 14.2, 4.2 Hz, 1H), 2.60 (dd, *J* = 14.2, 10.5 Hz, 1H), 1.39 – 1.30 (m, 1H), 1.20 (d, *J* = 2.0 Hz, 12H), 0.73 (d, *J* = 7.4 Hz, 3H).

^{13}C NMR (75 MHz, $CDCl_3$) δ 170.1, 143.3, 139.9, 129.9, 128.5, 127.8, 126.5, 126.0, 123.8, 121.6, 118.4, 116.2 (d, *J* = 261.1 Hz), 83.3, 45.4, 45.1, 25.0, 24.8, 24.6, 13.9.

^{11}B NMR (96 MHz, $CDCl_3$) δ 33.2.

^{19}F NMR (282 MHz, $CDCl_3$) δ -79.75 (d, *J* = 6.4 Hz), -80.01 (d, *J* = 6.1 Hz).

HRMS (ESI-TOF): calcd for $[M+Na]^+$ $C_{24}H_{30}{}^{11}BF_2NO_4$ 468.2132, found: 468.2128.

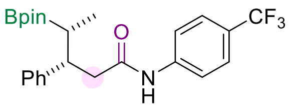

3-Phenyl-4-(4,4,5,5-tetramethyl-1,3,2-dioxaborolan-2-yl)-*N*-(4-(trifluoromethyl)phenyl)pentanamide (9a)

The title compound was prepared from *trans-β*-methylstyrene (0.2 mmol, 26 μL) and 4-(trifluoromethyl)aniline (2.5 equiv., 80.5 mg), according to general procedure. The crude residue was purified by flash chromatography (*n*-pentane/EA = 10:1, Rf = 0.2) to give the product as a white solid (47 mg, 53%).

^{1}H NMR (300 MHz, $CDCl_3$) δ 7.34 – 7.27 (m, 2H), 7.26 – 7.12 (m, 5H), 7.06 (d, J = 8.2 Hz, 3H), 3.13 (td, J = 10.2, 4.0 Hz, 1H), 2.80 (dd, J = 14.0, 4.1 Hz, 1H), 2.61 (dd, J = 14.0, 10.3 Hz, 1H), 1.52 – 1.39 (m, 1H), 1.29 (d, J = 1.6 Hz, 12H), 0.81 (d, J = 7.4 Hz, 3H).

^{13}C NMR (75 MHz, $CDCl_3$) δ 170.1, 143.6, 136.4, 128.7, 127.8, 126.7, 121.5, 120.8, 120.4 (q, J = 257.4 Hz), 83.5, 45.8, 45.3, 24.9, 24.6, 14.0.

^{11}B NMR (96 MHz, $CDCl_3$) δ 33.4.

^{19}F NMR (282 MHz, $CDCl_3$) δ -58.2.

HRMS (ESI-TOF): calcd for $[M+H]^+$ $C_{24}H_{29}{}^{10}BF_3NO_3$ 447.2302, found: 447.2293.

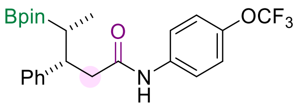

3-Phenyl-4-(4,4,5,5-tetramethyl-1,3,2-dioxaborolan-2-yl)-N-(4-(trifluoromethoxy)phenyl)pentanamide (10a)

The title compound was prepared from *trans-β*-methylstyrene (0.2 mmol, 26 μL) and 4-(trifluoromethoxy)aniline (2.5 equiv., 88.5 mg), according to general procedure. The crude residue was purified by flash chromatography (*n*-pentane/EA = 7:1, Rf = 0.2) to give the product as a white solid (28 mg, 30%).

^{1}H NMR (300 MHz, $CDCl_3$) δ 7.34 – 7.28 (m, 2H), 7.26 – 7.18 (m, 5H), 7.07 (d, J = 8.3 Hz, 2H), 7.00 (s, 1H), 3.13 (td, J = 10.3, 3.9 Hz, 1H), 2.80 (dd, J = 14.0, 4.0 Hz, 1H), 2.61 (dd, J = 13.9, 10.3 Hz, 1H), 1.50 – 1.41 (m, 1H), 1.29 (d, J = 1.5 Hz, 12H), 0.81 (d, J = 7.4 Hz, 3H).

^{13}C NMR (75 MHz, $CDCl_3$) δ 170.1, 143.6, 136.5, 128.8, 127.9, 126.8, 121.6, 120.8, 120.4 (q, J = 256.8 Hz), 83.5, 45.8, 45.4, 24.9, 24.7, 14.1.

^{11}B NMR (96 MHz, $CDCl_3$) δ 34.6.

^{19}F NMR (282 MHz, $CDCl_3$) δ -58.2.

HRMS (ESI-TOF): calcd for $[M+H]^+$ $C_{24}H_{29}{}^{10}BF3NO_4$ 463.2251, found: 463.2252.

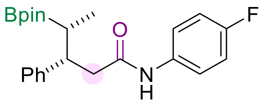

***N*-(4-Fluorophenyl)-3-phenyl-4-(4,4,5,5-tetramethyl-1,3,2-dioxaborolan-2-yl)pentanamide (11a)**

The title compound was prepared from *trans-β*-methylstyrene (0.2 mmol, 26 μL) and 4-fluoroaniline (2.5 equiv., 55.5 mg), according to general procedure. The crude residue was purified by flash chromatography (*n*-pentane/EA = 10:1, Rf = 0.2) to give the product as a white solid (46 mg, 58%).

^{1}H NMR (300 MHz, $CDCl_3$) δ 7.34 – 7.27 (m, 2H), 7.24 – 7.18 (m, 3H), 7.19 – 7.10 (m, 2H), 7.02 – 6.83 (m, 3H), 3.14 (td, *J* = 10.3, 4.1 Hz, 1H), 2.79 (dd, *J* = 13.9, 4.1 Hz, 1H), 2.59 (dd, *J* = 13.9, 10.4 Hz, 1H), 1.50 – 1.40 (m, 1H), 1.28 (d, *J* = 1.4 Hz, 12H), 0.81 (d, *J* = 7.4 Hz, 3H).

^{13}C NMR (75 MHz, $CDCl_3$) δ 170.0, 159.2 (d, *J* = 243.6 Hz), 143.6, 133.7 (d, *J* = 2.3 Hz) 128.7, 127.8, 126.7, 121.6 (d, *J* = 7.8 Hz), 115.4 (d, *J* = 22.4 Hz), 83.4, 45.8, 45.2, 24.9, 24.7, 14.0.

^{11}B NMR (96 MHz, $CDCl_3$) δ 33.6.

^{19}F NMR (282 MHz, $CDCl_3$) δ -118.48 (tt, *J* = 9.1, 5.1 Hz).

HRMS (ESI-TOF): calcd for $[M+H]^+$ $C_{23}H_{29}{}^{11}BFNO_3$ 398.2302, found: 398.2306.

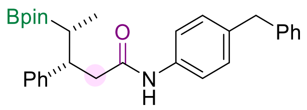

N-(4-Benzylphenyl)-3-phenyl-4-(4,4,5,5-tetramethyl-1,3,2-dioxaborolan-2-yl)pentanamide (12a)

The title compound was prepared from *trans-β*-methylstyrene (0.2 mmol, 26 μL) and 4-benzylaniline (2.5 equiv., 91.5 mg), according to general procedure. The crude residue was purified by flash chromatography (*n*-pentane/EA = 10:1, Rf = 0.2) to give the product as a white solid (50 mg, 49%).

^{1}H NMR (300 MHz, $CDCl_3$) δ 7.32 – 7.21 (m, 7H), 7.21 – 7.11 (m, 5H), 7.09 – 6.99 (m, 3H), 3.91 (s, 2H), 3.17 (td, *J* = 10.1, 4.1 Hz, 1H), 2.81 (dd, *J* = 14.0, 4.1 Hz, 1H), 2.62 (dd, *J* = 14.0, 10.3 Hz, 1H), 1.57 – 1.39 (m, 1H), 1.30 (d, *J* = 1.4 Hz, 12H), 0.83 (d, *J* = 7.4 Hz, 3H).

^{13}C NMR (75 MHz, $CDCl_3$) δ 170.0, 143.7, 141.1, 136.8, 135.9, 129.2, 128.9, 128.7, 128.4, 127.9, 126.7, 126.0, 120.0, 83.5, 45.8, 45.3, 41.3, 24.9, 24.6, 14.0.

^{11}B NMR (128 MHz, $CDCl_3$) δ 33.3.

HRMS (ESI-TOF): calcd for $[M+Na]^+$ $C_{30}H_{36}{}^{10}BNO_3$ 491.2717, found: 491.2720.

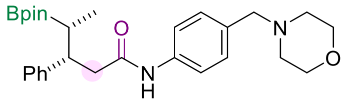

N-(4-(Morpholinomethyl)phenyl)-3-phenyl-4-(4,4,5,5-tetramethyl-1,3,2-dioxaborolan-2-yl)pentanamide (13a)

The title compound was prepared from *trans-β*-methylstyrene (0.2 mmol, 26 μL) and 4-(morpholinomethyl)aniline (2.5 equiv., 96 mg), according to general procedure. The crude residue was purified by flash chromatography (*n*-pentane/EA = 1:1, Rf = 0.2) to give the product as a white solid (39 mg, 41%).

^{1}H NMR (300 MHz, $CDCl_3$) δ 7.37 – 7.27 (m, 2H), 7.26 – 7.04 (m, 7H), 7.00 (s, 1H), 3.79 – 3.66 (m, 4H), 3.45 (s, 2H), 3.14 (td, *J* = 10.2, 4.1 Hz, 1H), 2.79 (dd, *J* = 14.0, 4.0 Hz, 1H), 2.61 (d, *J* = 3.7 Hz, 1H), 2.51 – 2.33 (m, 4H), 1.45 (dd, *J* = 10.1, 7.5 Hz, 1H), 1.28 (d, *J* = 1.7 Hz, 12H), 0.80 (d, *J* = 7.4 Hz, 3H).

^{13}C NMR (75 MHz, $CDCl_3$) δ 170.0, 143.7, 129.9, 128.7, 128.7, 127.9, 126.7, 119.6, 83.4, 66.6, 62.7, 53.3, 45.7, 45.3, 24.9, 24.7, 14.0.

^{11}B NMR (96 MHz, $CDCl_3$) δ 35.1.

HRMS (ESI-TOF): calcd for $[M+H]^+$ $C_{28}H_{39}{}^{10}BN_2O_4$ 478.3112, found: 478.3108.

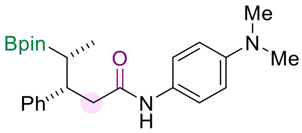

N-(4-(Dimethylamino)phenyl)-3-phenyl-4-(4,4,5,5-tetramethyl-1,3,2-dioxaborolan-2-yl)pentanamide (14a)

The title compound was prepared from *trans-β*-methylstyrene (0.2 mmol, 26 µL) and N^1,N^1-dimethylbenzene-1,4-diamine (2.5 equiv., 68 mg), according to general procedure. The crude residue was purified by flash chromatography (*n*-pentane/EA = 1:1, Rf = 0.2) to give the product as a white solid (36 mg, 43%).

^{1}H NMR (300 MHz, $CDCl_3$) δ 7.32 – 7.16 (m, 5H), 7.09 (d, *J* = 8.7 Hz, 2H), 7.00 – 6.40 (m, 3H), 3.15 (td, *J* = 10.2, 4.1 Hz, 1H), 2.85 (dd, *J* = 38.5, 12.4 Hz, 6H), 2.73 (s, 1H), 2.58 (dd, *J* = 13.8, 10.4 Hz, 1H), 1.45 (dd, *J* = 9.5, 6.9 Hz, 1H), 1.28 (d, *J* = 1.5 Hz, 12H), 0.81 (d, *J* = 7.4 Hz, 3H).

^{13}C NMR (75 MHz, $CDCl_3$) δ 169.8, 143.8, 128.6, 127.9, 126.6, 121.6, 83.4, 45.8, 45.1, 41.7, 24.9, 24.7, 14.0.

^{11}B NMR (96 MHz, $CDCl_3$) δ 34.0.

HRMS (ESI-TOF): calcd for $[M+H]^+$ $C_{25}H_{35}{}^{10}BN_2O_3$ 422.2850, found: 422.2847.

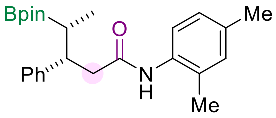

N-(2,4-Dimethylphenyl)-3-phenyl-4-(4,4,5,5-tetramethyl-1,3,2-dioxaborolan-2-yl)pentanamide (15a)

The title compound was prepared from *trans-β*-methylstyrene (0.2 mmol, 26 µL) and 2,4-dimethylaniline (2.5 equiv., 60.5 mg), according to general procedure. The crude residue was purified by flash chromatography (*n*-pentane/EA = 10:1, Rf = 0.2) to give the product as a white solid (36 mg, 44%).

^{1}H NMR (300 MHz, $CDCl_3$) δ 7.34 (s, 1H), 7.23 – 7.10 (m, 5H), 6.85 (d, *J* = 7.6 Hz, 1H), 6.72 (d, *J* = 7.2 Hz, 1H), 6.56 (s, 1H), 3.11 (td, *J* = 10.6, 3.9 Hz, 1H), 2.77 (dd, *J* = 13.8, 3.9 Hz, 1H), 2.59 (dd, *J* = 13.9, 11.0 Hz, 1H), 2.17 (s, 3H), 1.70 (s, 3H), 1.25 (d, *J* = 7.8 Hz, 1H), 1.21 (d, *J* = 2.2 Hz, 12H), 0.73 (d, *J* = 7.4 Hz, 3H).

^{13}C NMR (75 MHz, $CDCl_3$) δ 170.0, 155.7, 143.6, 136.2, 135.4, 129.9, 128.7, 128.0, 126.7, 125.8, 125.6, 123.6, 83.4, 45.7, 45.1, 24.9, 24.6, 21.0, 16.8, 13.9.

^{11}B NMR (96 MHz, $CDCl_3$) δ 34.1.

HRMS (ESI-TOF): calcd for $[M+H]^+$ $C_{25}H_{34}{}^{10}BNO_3$ 407.2741, found: 407.2741.

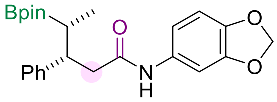

N-(Benzo[*d*][1,3]dioxol-5-yl)-3-phenyl-4-(4,4,5,5-tetramethyl-1,3,2-dioxaborolan-2-yl)pentanamide (16a)

The title compound was prepared from *trans-β*-methylstyrene (0.2 mmol, 26 µL) and benzo[*d*][1,3]dioxol-5-amine (2.5 equiv., 68.5 mg), according to general procedure. The crude residue was purified by flash chromatography (*n*-pentane/EA = 6:1, Rf = 0.2) to give the product as a white solid (49 mg, 58%).

^{1}H NMR (300 MHz, $CDCl_3$) δ 7.34 – 7.27 (m, 2H), 7.22 (d, *J* = 6.9 Hz, 3H), 6.92 (d, *J* = 2.1 Hz, 1H), 6.87 (s, 1H), 6.63 (d, *J* = 8.3 Hz, 1H), 6.44 (dd, *J* = 8.3, 2.1 Hz, 1H), 5.88 (s, 2H), 3.13 (td, *J* = 10.3, 4.1 Hz, 1H), 2.77 (dd, *J* = 13.9, 4.1 Hz, 1H), 2.56 (dd, *J* = 13.9, 10.4 Hz, 1H), 1.52 – 1.42 (m, 1H), 1.29 – 1.27 (m, 12H), 0.80 (d, *J* = 7.4 Hz, 3H).

^{13}C NMR (75 MHz, $CDCl_3$) δ 169.9, 147.5, 144.0, 143.7, 132.0, 128.6, 127.8, 126.7, 113.0, 107.8, 102.9, 101.1, 83.4, 45.8, 45.2, 24.9, 24.7, 14.0.

^{11}B NMR (96 MHz, $CDCl_3$) δ 34.6.

HRMS (ESI-TOF): calcd for $[M+H]^+$ $C_{24}H_{30}{}^{11}BNO_5$ 424.2294, found: 424.2294.

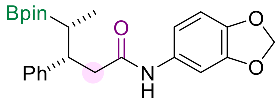

N-(2,2-Difluorobenzo[*d*][1,3]dioxol-5-yl)-3-phenyl-4-(4,4,5,5-tetramethyl-1,3,2-dioxaborolan-2-yl)pentanamide (17a)

The title compound was prepared from *trans-β*-methylstyrene (0.2 mmol, 26 μL) and 2,2-difluorobenzo[*d*][1,3]dioxol-5-amine (2.5 equiv., 86.5 mg), according to general procedure. The crude residue was purified by flash chromatography (*n*-pentane/EA = 7:1, Rf = 0.2) to give the product as a white solid (36 mg, 40%).

^1H NMR (400 MHz, $CDCl_3$) δ 7.38 – 7.27 (m, 3H), 7.22 (td, *J* = 6.4, 1.7 Hz, 3H), 6.98 (s, 1H), 6.85 (d, *J* = 8.6 Hz, 1H), 6.59 (dd, *J* = 8.6, 2.1 Hz, 1H), 3.12 (td, *J* = 10.3, 4.0 Hz, 1H), 2.79 (dd, *J* = 13.9, 4.0 Hz, 1H), 2.59 (dd, *J* = 13.9, 10.4 Hz, 1H), 1.45 (dd, *J* = 10.2, 7.4 Hz, 1H), 1.29 (d, *J* = 2.3 Hz, 12H), 0.81 (d, *J* = 7.4 Hz, 3H).

^{13}C NMR (101 MHz, $CDCl_3$) δ 170.1, 143.7, 143.5, 139.9, 133.9, 131.7 (t, *J* = 231.5 Hz), 128.7, 127.8, 126.8, 114.4, 109.0, 103.1, 83.5, 45.9, 45.3, 24.9, 24.7, 14.0.

^{11}B NMR (128 MHz, $CDCl_3$) δ 35.4.

^{19}F NMR (282 MHz, $CDCl_3$) δ -50.0.

HRMS (ESI-TOF): calcd for $[M+Na]^+$ $C_{24}H_{28}{}^{10}BF_2NO_5$ 481.1957, found: 481.1956.

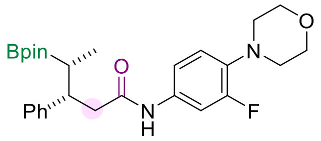

N-(3-Fluoro-4-morpholinophenyl)-3-phenyl-4-(4,4,5,5-tetramethyl-1,3,2-dioxaborolan-2-yl)pentanamide (18a)

The title compound was prepared from *trans-β*-methylstyrene (0.2 mmol, 26 μL) and 3-fluoro-4-morpholinoaniline (2.5 equiv., 98 mg), according to general procedure. The crude residue was purified by flash chromatography (*n*-pentane/EA = 5:1, Rf = 0.3) to give the product as a white solid (58 mg, 60%).

^1H NMR (300 MHz, $CDCl_3$) δ 7.31 – 7.26 (m, 2H), 7.25 – 7.11 (m, 4H), 7.02 (s, 1H), 6.88 – 6.62 (m, 2H), 3.89 – 3.80 (m, 4H), 3.13 (dt, *J* = 10.2, 5.1 Hz, 1H), 3.05 – 2.92 (m, 4H), 2.77 (dd, *J* = 14.0, 4.1 Hz, 1H), 2.58 (dd, *J* = 14.0, 10.3 Hz, 1H), 1.56 – 1.37 (m, 1H), 1.27 (d, *J* = 1.7 Hz, 12H), 0.80 (d, *J* = 7.4 Hz, 3H).

^{13}C NMR (75 MHz, $CDCl_3$) δ 170.0, 155.3 (d, *J* = 245.7 Hz), 143.6, 135.7 (d, *J* = 16.6 Hz), 133.3 (d, *J* = 12.6 Hz), 128.6, 127.8, 126.7, 118.7 (d, *J* = 3.6 Hz), 115.5 (d, *J* = 3.3 Hz), 108.9 (d, *J* = 25.4 Hz), 83.4, 66.8, 51.1, 45.7, 45.2, 24.9, 24.6, 14.0.

^{11}B NMR (96 MHz, $CDCl_3$) δ 33.9.

^{19}F NMR (282 MHz, $CDCl_3$) δ -121.2.

HRMS (ESI-TOF): calcd for $[M+Na]^+$ $C_{27}H_{36}{}^{10}BFN_2O_4$ 504.2680, found: 504.2679.

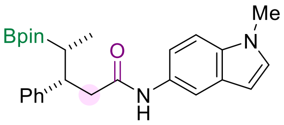

N-(1-Methyl-1H-indol-5-yl)-3-phenyl-4-(4,4,5,5-tetramethyl-1,3,2-dioxaborolan-2-yl)pentanamide (19a)

The title compound was prepared from *trans-β*-methylstyrene (0.2 mmol, 26 μL) and 1-methyl-1*H*-indol-5-amine (2.5 equiv., 73 mg), according to general procedure. The crude residue was purified by flash chromatography (*n*-pentane/EA = 3:1, Rf = 0.2) to give the product as a white solid (42 mg, 49%).

^{1}H NMR (300 MHz, $CDCl_3$) δ 7.54 (d, *J* = 1.9 Hz, 1H), 7.34 – 7.22 (m, 5H), 7.13 (d, *J* = 8.7 Hz, 1H), 7.01 – 6.90 (m, 3H), 6.37 (dd, *J* = 3.1, 0.7 Hz, 1H), 3.71 (s, 3H), 3.21 (td, *J* = 10.2, 4.2 Hz, 1H), 2.83 (dd, *J* = 13.9, 4.2 Hz, 1H), 2.62 (dd, *J* = 13.9, 10.4 Hz, 1H), 1.52 – 1.43 (m, 1H), 1.30 (d, *J* = 1.3 Hz, 12H), 0.84 (d, *J* = 7.4 Hz, 3H).

^{13}C NMR (75 MHz, $CDCl_3$) δ 169.9, 143.9, 134.0, 130.1, 129.4, 128.5, 128.3, 127.9, 126.5, 115.8, 112.6, 109.0, 100.9, 83.3, 45.8, 45.2, 32.8, 24.9, 24.6, 14.0.

^{11}B NMR (96 MHz, $CDCl_3$) δ 32.9.

HRMS (ESI-TOF): calcd for $[M+H]^+$ $C_{26}H_{33}{}^{11}BN_2O_3$ 433.2662, found: 433.2670.

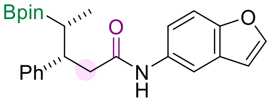

N-(Benzofuran-5-yl)-3-phenyl-4-(4,4,5,5-tetramethyl-1,3,2-dioxaborolan-2-yl)pentanamide (20a)

The title compound was prepared from *trans-β*-methylstyrene (0.2 mmol, 26 μL) and benzo[*d*][1,3]dioxol-5-amine (2.5 equiv., 68.5 mg), according to general procedure. The crude residue was purified by flash chromatography (*n*-pentane/EA = 6:1, Rf = 0.2) to give the product as a white solid (51 mg, 61%).

^{1}H NMR (300 MHz, $CDCl_3$) δ 7.66 (d, *J* = 2.0 Hz, 1H), 7.55 (d, *J* = 2.2 Hz, 1H), 7.34 – 7.27 (m, 3H), 7.26 – 7.20 (m, 3H), 7.10 (s, 1H), 6.89 (dd, *J* = 8.8, 2.1 Hz, 1H), 6.66 (dd, *J* = 2.1, 0.8 Hz, 1H), 3.18 (td, *J* = 10.2, 4.2 Hz, 1H), 2.83 (dd, *J* = 13.9, 4.2 Hz, 1H), 2.63 (dd, *J* = 13.9, 10.3 Hz, 1H), 1.50 – 1.42 (m, 1H), 1.30 – 1.27 (m, 12H), 0.82 (d, *J* = 7.5 Hz, 3H).

^{13}C NMR (75 MHz, $CDCl_3$) δ 170.1, 151.8, 145.6, 143.7, 132.9, 128.6, 127.9, 127.6, 126.6, 117.5, 113.0, 111.1, 106.7, 83.4, 45.8, 45.1, 24.9, 24.7, 14.0.

^{11}B NMR (96 MHz, $CDCl_3$) δ 33.0.

HRMS (ESI-TOF): calcd for $[M+H]^+$ $C_{25}H_{30}{}^{11}BNO_3$ 420.2345, found: 420.2343.

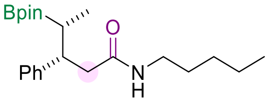

N-Pentyl-3-phenyl-4-(4,4,5,5-tetramethyl-1,3,2-dioxaborolan-2-yl)pentanamide (1b)

The title compound was prepared from *trans-β*-methylstyrene (0.2 mmol, 26 μL) and pentan-1-amine (2.5 equiv., 39 mg), according to general procedure. The crude residue was purified by flash chromatography (*n*-pentane/EA = 5:1, Rf = 0.2) to give the product as a white solid (33 mg, 44%).

^{1}H NMR (300 MHz, $CDCl_3$) δ 7.31 – 7.26 (m, 1H), 7.24 (d, *J* = 2.0 Hz, 1H), 7.20 – 7.13 (m, 3H), 5.12 (s, 1H), 3.12 – 2.89 (m, 3H), 2.64 (dd, *J* = 13.9, 4.2 Hz, 1H), 2.39 (dd, *J* = 13.9, 11.0 Hz, 1H), 1.41 – 1.33 (m, 1H), 1.26

(d, J = 2.0 Hz, 12H), 1.17 (ddd, J = 9.4, 6.3, 2.6 Hz, 4H), 1.01 (ddd, J = 12.6, 7.5, 2.3 Hz, 2H), 0.84 – 0.73 (m, 6H).

^{13}C NMR (75 MHz, $CDCl_3$) δ 171.6, 143.7, 128.4, 127.8, 126.4, 83.3, 45.6, 44.1, 39.2, 36.3, 29.0, 28.8, 24.8, 24.6, 22.2, 13.9.

^{11}B NMR (96 MHz, $CDCl_3$) δ 34.2.

HRMS (ESI-TOF): calcd for $[M+Na]^+$ $C_{22}H_{36}{}^{10}BNO_3$ 395.2717, found: 395.2721.

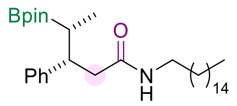

N-Hexadecyl-3-phenyl-4-(4,4,5,5-tetramethyl-1,3,2-dioxaborolan-2-yl)pentanamide (2b)

The title compound was prepared from *trans-β*-methylstyrene (0.2 mmol, 26 µL) and hexadecan-1-amine (2.5 equiv., 120 mg), according to general procedure. The crude residue was purified by flash chromatography (*n*-pentane/EA = 5:1, Rf = 0.2) to give the product as a white solid (60 mg, 57%).

^{1}H NMR (300 MHz, $CDCl_3$) δ 7.29 – 7.24 (m, 2H), 7.21 – 7.14 (m, 3H), 5.44 (s, 1H), 3.09 – 2.97 (m, 3H), 2.66 (dd, J = 14.0, 4.6 Hz, 1H), 2.44 (dd, J = 14.0, 10.6 Hz, 1H), 1.34 (d, J = 7.5 Hz, 1H), 1.29 – 1.18 (m, 40H), 0.90 – 0.85 (m, 3H), 0.77 (d, J = 7.4 Hz, 3H).

^{13}C NMR (75 MHz, $CDCl_3$) δ 172.0, 143.7, 128.4, 127.8, 126.4, 83.3, 45.5, 43.6, 39.5, 31.9, 29.7, 29.7, 29.6, 29.6, 29.5, 29.3, 29.2, 26.7, 24.8, 24.7, 22.8, 22.7, 14.5, 14.1, 13.9.

^{11}B NMR (96 MHz, $CDCl_3$) δ 32.8.

HRMS (ESI-TOF): calcd for $[M+H]^+$ $C_{33}H_{58}{}^{10}BNO_3$ 527.4619, found: 527.4622.

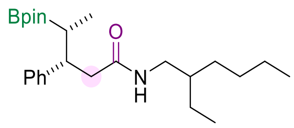

N-(2-Ethylhexyl)-3-phenyl-4-(4,4,5,5-tetramethyl-1,3,2-dioxaborolan-2-yl)pentanamide (3b)

The title compound was prepared from *trans-β*-methylstyrene (0.2 mmol, 26 µL) and 2-ethylhexan-1-amine (2.5 equiv., 129 mg), according to general procedure. The crude residue was purified by flash chromatography (*n*-pentane/EA = 5:1, Rf = 0.2) to give the product as a white solid (49 mg, 59%).

^{1}H NMR (400 MHz, $CDCl_3$) δ 7.28 – 7.24 (m, 2H), 7.20 – 7.12 (m, 3H), 5.07 (s, 1H), 3.06 (td, J = 10.5, 3.8 Hz, 1H), 3.02 – 2.84 (m, 2H), 2.64 (dd, J = 14.0, 4.0 Hz, 1H), 2.44 (dd, J = 14.0, 11.1 Hz, 1H), 1.36 – 1.31 (m, 1H), 1.25 (d, J = 3.3 Hz, 12H), 1.19 (dd, J = 8.1, 5.7 Hz, 2H), 1.14 – 1.06 (m, 3H), 1.02 – 0.89 (m, 4H), 0.85 (t, J = 7.2 Hz, 3H), 0.76 (d, J = 7.4 Hz, 3H), 0.71 (td, J = 7.4, 2.1 Hz, 3H).

^{13}C NMR (101 MHz, $CDCl_3$) δ 171.7, 143.7, 128.5, 127.9, 126.5, 83.3, 45.5, 44.1, 41.9, 39.1, 30.7, 28.8, 24.9, 24.7, 23.9, 22.9, 14.1, 13.9, 10.8.

^{11}B NMR (128 MHz, $CDCl_3$) δ 33.3.

HRMS (ESI-TOF): calcd for $[M+H]^+$ $C_{25}H_{42}{}^{10}BNO_3$ 415.3367, found: 415.3372.

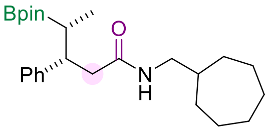

***N*-(Cycloheptylmethyl)-3-phenyl-4-(4,4,5,5-tetramethyl-1,3,2-dioxaborolan-2-yl)pentanamide (4b)**

The title compound was prepared from *trans*-*β*-methylstyrene (0.2 mmol, 26 μL) and cycloheptylmethanamine (2.5 equiv., 63.5 mg), according to general procedure. The crude residue was purified by flash chromatography (*n*-pentane/EA = 5:1, Rf = 0.2) to give the product as a white solid (48 mg, 58%).

^{1}H NMR (300 MHz, $CDCl_3$) δ 7.28 – 7.23 (m, 2H), 7.20 – 7.11 (m, 3H), 5.31 (s, 1H), 3.06 (td, *J* = 10.6, 4.1 Hz, 1H), 2.91 (dt, *J* = 12.8, 6.3 Hz, 1H), 2.79 (dt, *J* = 13.1, 5.9 Hz, 1H), 2.65 (dd, *J* = 14.0, 4.1 Hz, 1H), 2.43 (dd, *J* = 13.9, 11.1 Hz, 2H), 1.60 – 1.41 (m, 5H), 1.35 (ddt, *J* = 19.0, 6.3, 2.8 Hz, 6H), 1.26 – 1.24 (m, 12H), 0.92 – 0.82 (m, 2H), 0.76 (d, *J* = 7.4 Hz, 3H).

^{13}C NMR (75 MHz, $CDCl_3$) δ 171.8, 143.6, 128.4, 127.9, 126.4, 83.3, 45.9, 45.5, 44.0, 39.3, 31.9, 31.8, 28.2, 28.1, 26.3, 26.2, 24.9, 24.7, 13.9.

^{11}B NMR (96 MHz, $CDCl_3$) δ 33.1.

HRMS (ESI-TOF): calcd for $[M+H]^+$ $C_{25}H_{40}{}^{10}BNO_3$ 413.3210, found: 413.3210.

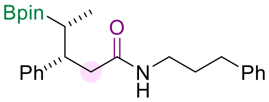

3-Phenyl-*N*-(3-phenylpropyl)-4-(4,4,5,5-tetramethyl-1,3,2-dioxaborolan-2-yl)pentanamide (5b)

The title compound was prepared from *trans*-*β*-methylstyrene (0.2 mmol, 26 μL) and 3-phenylpropan-1-amine (2.5 equiv., 67.5 mg), according to general procedure. The crude residue was purified by flash chromatography (*n*-pentane/EA = 5:1, Rf = 0.2) to give the product as a white solid (55 mg, 65%).

^{1}H NMR (300 MHz, $CDCl_3$) δ 7.29 – 7.22 (m, 4H), 7.21 – 7.12 (m, 4H), 7.10 – 6.96 (m, 2H), 5.13 (t, *J* = 5.1 Hz, 1H), 3.21 – 2.89 (m, 3H), 2.64 (dd, *J* = 13.9, 4.1 Hz, 1H), 2.54 – 2.19 (m, 3H), 1.51 (p, *J* = 7.2 Hz, 2H), 1.40 – 1.36 (m, 1H), 1.26 (d, *J* = 2.2 Hz, 12H), 0.77 (d, *J* = 7.4 Hz, 3H).

^{13}C NMR (75 MHz, $CDCl_3$) δ 171.6, 143.7, 141.4, 128.4, 128.3, 127.9, 126.4, 125.8, 83.2, 45.5, 44.1, 38.7, 32.8, 30.9, 24.8, 24.6, 13.9.

^{11}B NMR (96 MHz, $CDCl_3$) δ 33.0.

HRMS (ESI-TOF): calcd for $[M+H]^+$ $C_{26}H_{36}{}^{11}BNO_3$ 422.2866, found: 422.2870.

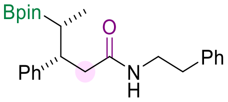

***N*-Phenethyl-3-phenyl-4-(4,4,5,5-tetramethyl-1,3,2-dioxaborolan-2-yl)pentanamide (6b)**

The title compound was prepared from *trans*-*β*-methylstyrene (0.2 mmol, 26 μL) and 2-phenylethan-1-amine (2.5 equiv., 60.5 mg), according to general procedure. The crude residue was purified by flash chromatography (*n*-pentane/EA = 5:1, Rf = 0.2) to give the product as a white solid (42 mg, 52%).

^{1}H NMR (300 MHz, $CDCl_3$) δ 7.29 – 7.19 (m, 5H), 7.16 (ddd, *J* = 7.8, 4.2, 1.7 Hz, 3H), 7.06 – 6.84 (m, 2H), 5.24 (s, 1H), 3.37 (dq, *J* = 13.5, 6.4 Hz, 1H), 3.28 – 2.96 (m, 2H), 2.63 (dd, *J* = 13.9, 4.2 Hz, 1H), 2.58 – 2.30 (m, 3H), 1.38 – 1.32 (m, 1H), 1.25 (d, *J* = 2.2 Hz, 12H), 0.76 (d, *J* = 7.4 Hz, 3H).

^{13}C NMR (75 MHz, $CDCl_3$) δ 171.7, 143.7, 138.9, 128.6, 128.5, 128.4, 127.9, 126.4, 126.3, 83.2, 45.4, 44.0, 40.4, 35.5, 24.8, 24.6, 13.8.
^{11}B NMR (96 MHz, $CDCl_3$) δ 33.3.
HRMS (ESI-TOF): calcd for $[M+Na]^+$ $C_{25}H_{34}{}^{11}BNO_3$ 430.2528, found: 430.2529.

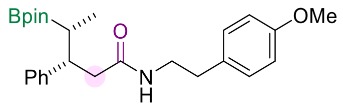

N-(4-Methoxyphenethyl)-3-phenyl-4-(4,4,5,5-tetramethyl-1,3,2-dioxaborolan-2-yl)pentanamide (7b)
The title compound was prepared from *trans-β*-methylstyrene (0.2 mmol, 26 μL) and 2-(4-methoxyphenyl)ethan-1-amine (2.5 equiv., 75.5 mg), according to general procedure. The crude residue was purified by flash chromatography (*n*-pentane/EA = 4:1, Rf = 0.2) to give the product as a white solid (48 mg, 55%).
^1H NMR (300 MHz, $CDCl_3$) δ 7.31 – 7.25 (m, 2H), 7.23 – 7.15 (m, 3H), 6.92 – 6.85 (m, 2H), 6.83 – 6.74 (m, 2H), 5.29 (s, 1H), 3.80 (s, 3H), 3.35 (dq, *J* = 13.4, 6.4 Hz, 1H), 3.23 – 3.05 (m, 2H), 2.64 (dd, *J* = 13.9, 4.2 Hz, 1H), 2.54 – 2.35 (m, 3H), 1.40 – 1.32 (m, 1H), 1.27 (d, *J* = 2.3 Hz, 12H), 0.78 (d, *J* = 7.4 Hz, 3H).
^{13}C NMR (75 MHz, $CDCl_3$) δ 171.6, 158.1, 143.7, 130.9, 129.5, 128.3, 127.8, 126.3, 113.8, 83.2, 55.2, 45.3, 43.9, 40.5, 34.5, 24.8, 24.6, 13.8.
^{11}B NMR (96 MHz, $CDCl_3$) δ 32.7.
HRMS (ESI-TOF): calcd for $[M+Na]^+$ $C_{26}H_{36}{}^{11}BNO_4$ 460.2634, found: 460.2635.

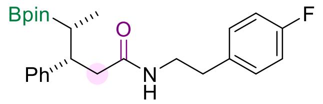

N-(4-Fluorophenethyl)-3-phenyl-4-(4,4,5,5-tetramethyl-1,3,2-dioxaborolan-2-yl)pentanamide (8b)
The title compound was prepared from *trans-β*-methylstyrene (0.2 mmol, 26 μL) and 2-(4-fluorophenyl)ethan-1-amine (2.5 equiv., 69.5 mg), according to general procedure. The crude residue was purified by flash chromatography (*n*-pentane/EA = 5:1, Rf = 0.2) to give the product as a white solid (36 mg, 42%).
^1H NMR (300 MHz, $CDCl_3$) δ 7.30 – 7.23 (m, 2H), 7.21 – 7.13 (m, 3H), 6.97 – 6.85 (m, 4H), 5.34 (s, 1H), 3.35 (dq, *J* = 13.4, 6.4 Hz, 1H), 3.22 – 3.02 (m, 2H), 2.66 – 2.30 (m, 4H), 1.34 (d, *J* = 4.1 Hz, 1H), 1.25 (d, *J* = 2.2 Hz, 12H), 0.75 (d, *J* = 7.4 Hz, 3H).
^{13}C NMR (75 MHz, $CDCl_3$) δ 171.9, 161.5 (d, *J* = 244.4 Hz), 143.7, 134.5 (d, *J* = 3.4 Hz), 130.1 (d, *J* = 7.8 Hz), 128.4, 127.9, 126.5, 115.27 (d, *J* = 21.1 Hz), 83.3, 45.4, 43.8, 40.6, 34.7, 24.9, 24.7, 13.9.
^{11}B NMR (96 MHz, $CDCl_3$) δ 32.8.
^{19}F NMR (282 MHz, $CDCl_3$) δ -116.89 (p, *J* = 7.0 Hz).
HRMS (ESI-TOF): calcd for $[M+Na]^+$ $C_{25}H_{33}{}^{11}BFNO_3$ 448.2434, found: 448.2434.

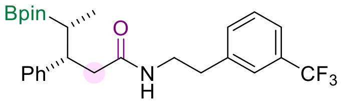

3-Phenyl-4-(4,4,5,5-tetramethyl-1,3,2-dioxaborolan-2-yl)-*N*-(3-(trifluoromethyl)phenethyl)pentanamide (9b)

The title compound was prepared from *trans*-*β*-methylstyrene (0.2 mmol, 26 μL) and 2-(3-(trifluoromethyl)phenyl)ethan-1-amine (2.5 equiv., 94.5 mg), according to general procedure. The crude residue was purified by flash chromatography (*n*-pentane/EA = 5:1, Rf = 0.2) to give the product as a white solid (44 mg, 46%).

^{1}H NMR (300 MHz, $CDCl_3$) δ 7.45 (d, *J* = 7.8 Hz, 1H), 7.35 (t, *J* = 7.7 Hz, 1H), 7.30 – 7.22 (m, 3H), 7.20 – 7.10 (m, 4H), 5.36 (s, 1H), 3.36 (dt, *J* = 13.7, 6.9 Hz, 1H), 3.20 (td, *J* = 13.0, 7.2 Hz, 1H), 3.05 (td, *J* = 10.5, 4.2 Hz, 1H), 2.66 – 2.57 (m, 2H), 2.49 (dt, *J* = 14.1, 7.4 Hz, 1H), 2.38 (dd, *J* = 14.0, 10.8 Hz, 1H), 1.35 – 1.32 (m, 1H), 1.25 (d, *J* = 1.9 Hz, 12H), 0.76 (d, *J* = 7.4 Hz, 3H).

^{13}C NMR (75 MHz, $CDCl_3$) δ 171.9, 143.6, 139.9, 132.1, 130.8 (q, *J* = 31.5 Hz), 128.9, 128.4, 127.8, 126.4, 125.3 (q, *J* = 3.7 Hz), 123.2 (q, *J* = 3.7 Hz), 119.8 (q, *J* = 251.0 Hz), 83.3, 45.5, 43.8, 40.2, 35.3, 24.8, 24.6, 13.9.

^{11}B NMR (96 MHz, $CDCl_3$) δ 32.9.

^{19}F NMR (282 MHz, $CDCl_3$) δ -62.5.

HRMS (ESI-TOF): calcd for $[M+Na]^+$ $C_{26}H_{33}{}^{11}BF_3NO_3$ 498.2402, found: 498.2405.

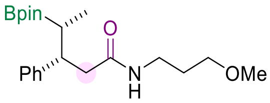

***N*-(3-Methoxypropyl)-3-phenyl-4-(4,4,5,5-tetramethyl-1,3,2-dioxaborolan-2-yl)pentanamide (10b)**

The title compound was prepared from *trans*-*β*-methylstyrene (0.2 mmol, 26 μL) and 3-methoxypropan-1-amine (2.5 equiv., 44.5 mg), according to general procedure. The crude residue was purified by flash chromatography (*n*-pentane/EA = 1:1, Rf = 0.2) to give the product as a white solid (40 mg, 53%).

^{1}H NMR (300 MHz, $CDCl_3$) δ 7.23 – 7.16 (m, 2H), 7.15 – 7.02 (m, 3H), 5.48 (s, 1H), 3.17 (s, 3H), 3.15 – 2.94 (m, 5H), 2.57 (dd, *J* = 13.8, 4.3 Hz, 1H), 2.31 (dd, *J* = 13.6, 10.7 Hz, 1H), 1.43 – 1.25 (m, 3H), 1.19 (d, *J* = 2.7 Hz, 12H), 0.70 (d, *J* = 7.4 Hz, 3H).

^{13}C NMR (75 MHz, $CDCl_3$) δ 171.5, 143.7, 128.3, 127.8, 126.3, 83.2, 70.9, 58.5, 45.4, 43.9, 37.2, 28.9, 24.8, 24.6, 24.5, 13.8.

^{11}B NMR (96 MHz, $CDCl_3$) δ 33.5.

HRMS (ESI-TOF): calcd for $[M+Na]^+$ $C_{21}H_{34}{}^{11}BNO_4$ 398.2477, found: 398.2478.

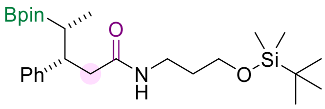

***N*-(3-((*tert*-Butyldimethylsilyl)oxy)propyl)-3-phenyl-4-(4,4,5,5-tetramethyl-1,3,2-dioxaborolan-2-yl)pentanamide (11b)**

The title compound was prepared from *trans*-*β*-methylstyrene (0.2 mmol, 26 μL) and 3-((*tert*-butyldimethylsilyl)oxy)propan-1-amine (2.5 equiv., 94.5 mg), according to general procedure. The crude residue

was purified by flash chromatography (*n*-pentane/EA = 4:1, Rf = 0.2) to give the product as a white solid (52 mg, 54%).

^{1}H NMR (300 MHz, $CDCl_3$) δ 7.24 (d, *J* = 2.3 Hz, 2H), 7.20 – 7.14 (m, 3H), 5.59 (s, 1H), 3.53 – 3.38 (m, 2H), 3.18 – 3.01 (m, 3H), 2.64 (dd, *J* = 13.8, 4.4 Hz, 1H), 2.34 (dd, *J* = 13.8, 10.8 Hz, 1H), 1.48 – 1.33 (m, 3H), 1.26 (d, *J* = 1.9 Hz, 12H), 0.88 (s, 9H), 0.78 (d, *J* = 7.4 Hz, 3H), 0.02 (s, 6H).

^{13}C NMR (75 MHz, $CDCl_3$) δ 171.4, 143.8, 128.3, 127.9, 126.2, 83.2, 61.6, 45.4, 44.0, 37.3, 31.6, 25.9, 24.8, 24.6, 18.2, 13.9, -5.4.

^{11}B NMR (96 MHz, $CDCl_3$) δ 33.7.

HRMS (ESI-TOF): calcd for $[M+H]^+$ $C_{26}H_{46}{}^{11}BNO_4Si$ 476.3367, found: 476.3370.

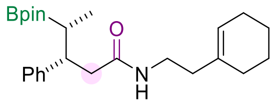

N-(2-(Cyclohex-1-en-1-yl)ethyl)-3-phenyl-4-(4,4,5,5-tetramethyl-1,3,2-dioxaborolan-2-yl)pentanamide (12b)

The title compound was prepared from *trans-β*-methylstyrene (0.2 mmol, 26 μL) and 2-(cyclohex-1-en-1-yl)ethan-1-amine (2.5 equiv., 62.5 mg), according to general procedure. The crude residue was purified by flash chromatography (*n*-pentane/EA = 5:1, Rf = 0.2) to give the product as a white solid (52 mg, 63%).

^{1}H NMR (300 MHz, $CDCl_3$) δ 7.29 – 7.24 (m, 2H), 7.16 (ddd, *J* = 8.2, 4.8, 1.7 Hz, 3H), 5.39 – 4.86 (m, 2H), 3.07 (dq, *J* = 10.0, 5.3 Hz, 3H), 2.64 (dd, *J* = 13.9, 4.4 Hz, 1H), 2.39 (dd, *J* = 13.9, 10.7 Hz, 1H), 1.93 (d, *J* = 1.8 Hz, 2H), 1.88 – 1.67 (m, 4H), 1.52 (tdd, *J* = 7.8, 6.2, 3.3 Hz, 4H), 1.40 – 1.33 (m, 1H), 1.25 (d, *J* = 2.2 Hz, 12H), 0.76 (d, *J* = 7.4 Hz, 3H).

^{13}C NMR (75 MHz, $CDCl_3$) δ 171.6, 143.7, 134.5, 128.3, 127.8, 126.4, 123.1, 83.2, 45.4, 43.9, 37.2, 36.9, 27.7, 25.1, 24.8, 24.7, 22.7, 22.3, 13.8.

^{11}B NMR (96 MHz, $CDCl_3$) δ 33.3.

HRMS (ESI-TOF): calcd for $[M+Na]^+$ $C_{25}H_{38}{}^{11}BNO_3$ 434.2841, found: 434.2838.

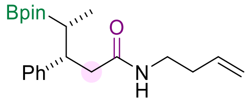

N-(But-3-en-1-yl)-3-phenyl-4-(4,4,5,5-tetramethyl-1,3,2-dioxaborolan-2-yl)pentanamide (13b)

The title compound was prepared from *trans-β*-methylstyrene (0.2 mmol, 26 μL) and but-3-en-1-amine (2.5 equiv., 30.5 mg), according to general procedure. The crude residue was purified by flash chromatography (*n*-pentane/EA = 5:1, Rf = 0.2) to give the product as a white solid (44 mg, 61%).

^{1}H NMR (300 MHz, $CDCl_3$) δ 7.29 – 7.24 (m, 2H), 7.17 (ddq, *J* = 6.8, 3.1, 1.8 Hz, 3H), 5.52 (ddt, *J* = 17.1, 10.3, 6.8 Hz, 1H), 5.30 (s, 1H), 5.01 – 4.77 (m, 2H), 3.17 – 3.00 (m, 3H), 2.65 (dd, *J* = 13.9, 4.4 Hz, 1H), 2.40 (dd, *J* = 13.9, 10.7 Hz, 1H), 2.05 – 1.85 (m, 2H), 1.39 – 1.32 (m, 1H), 1.26 (d, *J* = 1.9 Hz, 12H), 0.77 (d, *J* = 7.4 Hz, 3H).

^{13}C NMR (75 MHz, $CDCl_3$) δ 171.8, 143.7, 135.1, 128.4, 127.9, 126.4, 116.9, 83.3, 45.5, 43.8, 38.3, 33.4, 24.8, 24.6, 13.9.

^{11}B NMR (96 MHz, $CDCl_3$) δ 33.3.

HRMS (ESI-TOF): calcd for $[M+H]^+$ $C_{21}H_{32}{}^{10}BNO_3$ 357.2585, found: 357.2593.

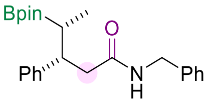

N-Benzyl-3-phenyl-4-(4,4,5,5-tetramethyl-1,3,2-dioxaborolan-2-yl)pentanamide (14b)

The title compound was prepared from *trans-β*-methylstyrene (0.2 mmol, 26 μL) and phenylmethanamine (2.5 equiv., 53.5 mg), according to general procedure. The crude residue was purified by flash chromatography (*n*-pentane/EA = 5:1, Rf = 0.2) to give the product as a white solid (30 mg, 38%).

^{1}H NMR (300 MHz, $CDCl_3$) δ 7.22 – 7.14 (m, 3H), 7.11 (ddt, *J* = 4.4, 2.2, 1.1 Hz, 5H), 6.93 – 6.57 (m, 2H), 5.42 (d, *J* = 5.1 Hz, 1H), 4.24 (dd, *J* = 14.9, 6.2 Hz, 1H), 4.08 – 4.00 (m, 1H), 3.06 (td, *J* = 10.8, 4.1 Hz, 1H), 2.65 (dd, *J* = 13.8, 4.1 Hz, 1H), 2.37 (dd, *J* = 13.9, 11.1 Hz, 1H), 1.32 – 1.26 (m, 1H), 1.18 (d, *J* = 1.7 Hz, 12H), 0.71 (d, *J* = 7.4 Hz, 3H).

^{13}C NMR (75 MHz, $CDCl_3$) δ 171.5, 143.6, 138.1, 128.4, 128.4, 127.9, 127.4, 127.1, 126.4, 83.2, 45.5, 44.0, 43.2, 24.8, 24.6, 13.9.

^{11}B NMR (96 MHz, $CDCl_3$) δ 33.2.

HRMS (ESI-TOF): calcd for $[M+H]^+$ $C_{24}H_{32}{}^{10}BNO_3$ 393.2585, found: 393.2588.

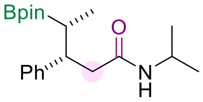

N-Isopropyl-3-phenyl-4-(4,4,5,5-tetramethyl-1,3,2-dioxaborolan-2-yl)pentanamide (15b)

The title compound was prepared from *trans-β*-methylstyrene (0.2 mmol, 26 μL) and propan-2-amine (2.5 equiv., 29.5 mg), according to general procedure. The crude residue was purified by flash chromatography (*n*-pentane/EA = 5:1, Rf = 0.2) to give the product as a white solid (38 mg, 55%).

^{1}H NMR (300 MHz, $CDCl_3$) δ 7.30 – 7.26 (m, 1H), 7.24 (s, 1H), 7.21 – 7.14 (m, 3H), 5.04 (d, *J* = 7.0 Hz, 1H), 3.92 – 3.71 (m, 1H), 3.04 (td, *J* = 10.6, 4.4 Hz, 1H), 2.62 (dd, *J* = 13.6, 4.4 Hz, 1H), 2.36 – 2.32 (m, 1H), 1.42 – 1.33 (m, 1H), 1.27 (d, *J* = 1.3 Hz, 12H), 0.92 (d, *J* = 6.6 Hz, 3H), 0.80 – 0.73 (m, 6H).

^{13}C NMR (75 MHz, $CDCl_3$) δ 171.0, 143.7, 128.4, 127.9, 126.4, 83.3, 45.8, 44.2, 41.0, 24.8, 24.6, 22.4, 22.3, 13.9.

^{11}B NMR (96 MHz, $CDCl_3$) δ 33.6.

HRMS (ESI-TOF): calcd for $[M+H]^+$ $C_{20}H_{32}{}^{11}BNO_3$ 346.2552, found: 346.2555.

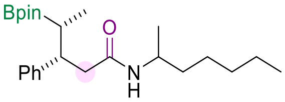

N-(Heptan-2-yl)-3-phenyl-4-(4,4,5,5-tetramethyl-1,3,2-dioxaborolan-2-yl)pentanamide (16b)

The title compound was prepared from *trans-β*-methylstyrene (0.2 mmol, 26 μL) and heptan-2-amine (2.5 equiv., 57.5 mg), according to general procedure. The crude residue was purified by flash chromatography (*n*-pentane/EA = 5:1, Rf = 0.2) to give the product as a white solid (51 mg, 64%).

^{1}H NMR (300 MHz, $CDCl_3$) δ 7.23 – 7.17 (m, 2H), 7.14 – 7.08 (m, 3H), 4.94 (d, *J* = 8.8 Hz, 1H), 3.72 – 3.57 (m, 1H), 2.97 (td, *J* = 10.8, 4.2 Hz, 1H), 2.58 (dd, *J* = 13.8, 4.2 Hz, 1H), 2.32 (dd, *J* = 13.8, 11.1 Hz, 1H), 1.37 – 1.27 (m, 1H), 1.20 (d, *J* = 1.6 Hz, 12H), 1.16 – 0.96 (m, 8H), 0.77 (t, *J* = 7.0 Hz, 3H), 0.71 (d, *J* = 7.4 Hz, 3H), 0.65 (d, *J* = 6.6 Hz, 3H).

^{13}C NMR (75 MHz, $CDCl_3$) δ 171.1, 143.6, 128.4, 127.9, 126.4, 83.3, 45.7, 44.9, 44.1, 36.6, 31.6, 25.4, 24.8, 24.7, 22.5, 20.5, 14.0, 13.9.

^{11}B NMR (96 MHz, $CDCl_3$) δ 33.1.

HRMS (ESI-TOF): calcd for $[M+H]^+$ $C_{24}H_{40}{}^{10}BNO_3$ 401.3210, found: 401.3219.

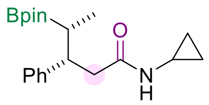

***N*-Cyclopropyl-3-phenyl-4-(4,4,5,5-tetramethyl-1,3,2-dioxaborolan-2-yl)pentanamide (17b)**

The title compound was prepared from *trans-β*-methylstyrene (0.2 mmol, 26 μL) and cyclopropanamine (2.5 equiv., 28.5 mg), according to general procedure. The crude residue was purified by flash chromatography (*n*-pentane/EA = 5:1, Rf = 0.2) to give the product as a white solid (28 mg, 41%).

^{1}H NMR (300 MHz, $CDCl_3$) δ 7.28 – 7.26 (m, 1H), 7.23 (q, *J* = 1.4 Hz, 1H), 7.20 – 7.12 (m, 3H), 5.21 (s, 1H), 3.03 (td, *J* = 10.6, 4.2 Hz, 1H), 2.60 (dd, *J* = 13.7, 4.3 Hz, 1H), 2.43 (tq, *J* = 7.0, 3.8 Hz, 1H), 2.31 (dd, *J* = 13.7, 11.0 Hz, 1H), 1.47 – 1.34 (m, 1H), 1.25 (d, *J* = 1.6 Hz, 12H), 0.76 (d, *J* = 7.4 Hz, 3H), 0.62 – 0.49 (m, 2H), 0.19 – -0.09 (m, 2H).

^{13}C NMR (75 MHz, $CDCl_3$) δ 173.1, 143.7, 128.3, 127.8, 126.4, 83.3, 45.7, 44.0, 24.8, 24.6, 22.1, 13.9, 6.5, 6.3.

^{11}B NMR (96 MHz, $CDCl_3$) δ 33.3.

HRMS (ESI-TOF): calcd for $[M+Na]^+$ $C_{20}H_{30}{}^{10}BNO_3$ 365.2247, found: 365.2255.

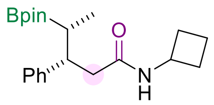

***N*-Cyclobutyl-3-phenyl-4-(4,4,5,5-tetramethyl-1,3,2-dioxaborolan-2-yl)pentanamide (18b)**

The title compound was prepared from *trans-β*-methylstyrene (0.2 mmol, 26 μL) and cyclobutanamine (2.5 equiv., 35.5 mg), according to general procedure. The crude residue was purified by flash chromatography (*n*-pentane/EA = 5:1, Rf = 0.2) to give the product as a white solid (35 mg, 49%).

^{1}H NMR (300 MHz, $CDCl_3$) δ 7.28 (dt, *J* = 7.2, 1.2 Hz, 1H), 7.25 (d, *J* = 0.7 Hz, 1H), 7.21 – 7.09 (m, 3H), 5.23 (d, *J* = 6.9 Hz, 1H), 4.15 (h, *J* = 7.9 Hz, 1H), 3.03 (td, *J* = 10.5, 4.2 Hz, 1H), 2.59 (dd, *J* = 13.7, 4.3 Hz, 1H), 2.33 (dd, *J* = 13.7, 10.8 Hz, 1H), 2.09 (dddd, *J* = 17.3, 6.2, 4.6, 2.8 Hz, 2H), 1.53 (dt, *J* = 5.0, 3.2 Hz, 2H), 1.46 – 1.28 (m, 3H), 1.26 (d, *J* = 1.6 Hz, 12H), 0.77 (d, *J* = 7.4 Hz, 3H).

^{13}C NMR (75 MHz, $CDCl_3$) δ 170.8, 143.8, 128.4, 127.9, 126.4, 83.3, 45.7, 44.3, 44.1, 31.0, 30.8, 24.8, 24.6, 14.9, 13.9.

^{11}B NMR (96 MHz, $CDCl_3$) δ 33.2.

HRMS (ESI-TOF): calcd for $[M+H]^+$ $C_{21}H_{32}{}^{10}BNO_3$ 357.2585, found: 357.2585.

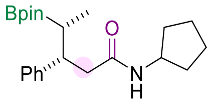

***N*-Cyclopentyl-3-phenyl-4-(4,4,5,5-tetramethyl-1,3,2-dioxaborolan-2-yl)pentanamide (19b)**

The title compound was prepared from *trans-β*-methylstyrene (0.2 mmol, 26 μL) and cyclopentanamine (2.5 equiv., 42.5 mg), according to general procedure. The crude residue was purified by flash chromatography (*n*-pentane/EA = 5:1, Rf = 0.2) to give the product as a white solid (34 mg, 47%).

^{1}H NMR (300 MHz, $CDCl_3$) δ 7.28 (dt, *J* = 7.0, 1.1 Hz, 1H), 7.26 – 7.21 (m, 1H), 7.21 – 7.12 (m, 3H), 5.05 (d, *J* = 7.0 Hz, 1H), 3.97 (h, *J* = 7.1 Hz, 1H), 3.03 (td, *J* = 10.7, 4.2 Hz, 1H), 2.61 (dd, *J* = 13.7, 4.2 Hz, 1H), 2.35 (dd, *J* = 13.7, 11.0 Hz, 1H), 1.80 – 1.59 (m, 2H), 1.41 (dtdd, *J* = 11.9, 8.2, 4.1, 2.2 Hz, 5H), 1.26 (d, *J* = 1.6 Hz, 12H), 1.06 (dd, *J* = 13.3, 7.2 Hz, 1H), 0.92 – 0.85 (m, 1H), 0.77 (d, *J* = 7.4 Hz, 3H).

^{13}C NMR (75 MHz, $CDCl_3$) δ 171.2, 143.7, 128.4, 127.9, 126.4, 83.3, 50.7, 45.8, 44.2, 32.8, 32.7, 24.8, 24.6, 23.5, 23.4, 13.9.

^{11}B NMR (96 MHz, $CDCl_3$) δ 33.5.

HRMS (ESI-TOF): calcd for $[M+H]^+$ $C_{22}H_{34}{}^{10}BNO_3$ 371.2741, found: 371.2750.

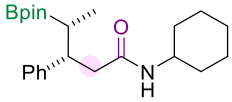

***N*-Cyclohexyl-3-phenyl-4-(4,4,5,5-tetramethyl-1,3,2-dioxaborolan-2-yl)pentanamide (20b)**

The title compound was prepared from *trans-β*-methylstyrene (0.2 mmol, 26 μL) and cyclohexanamine (2.5 equiv., 50.5 mg), according to general procedure. The crude residue was purified by flash chromatography (*n*-pentane/EA = 5:1, Rf = 0.2) to give the product as a white solid (42 mg, 55%).

^{1}H NMR (300 MHz, $CDCl_3$) δ 7.29 – 7.23 (m, 2H), 7.19 – 7.13 (m, 3H), 4.99 (d, *J* = 8.1 Hz, 1H), 3.64 – 3.45 (m, 1H), 3.03 (td, *J* = 10.7, 4.2 Hz, 1H), 2.60 (dd, *J* = 13.7, 4.2 Hz, 1H), 2.34 (dd, *J* = 13.7, 11.0 Hz, 1H), 1.71 – 1.61 (m, 1H), 1.48 (dtt, *J* = 9.6, 7.3, 3.5 Hz, 4H), 1.26 (d, *J* = 1.8 Hz, 12H), 1.18 – 0.81 (m, 4H), 0.77 (d, *J* = 7.4 Hz, 3H), 0.72 – 0.60 (m, 1H).

^{13}C NMR (75 MHz, $CDCl_3$) δ 170.6, 143.7, 128.3, 127.9, 126.3, 83.2, 47.5, 45.7, 44.3, 32.8, 32.7, 25.4, 24.8, 24.6, 13.9.

^{11}B NMR (96 MHz, $CDCl_3$) δ 34.4.

HRMS (ESI-TOF): calcd for $[M+H]^+$ $C_{23}H_{36}{}^{11}BNO_3$ 386.2865, found: 386.2870.

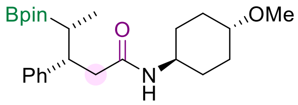

***N*-((1*r*,4*r*)-4-Methoxycyclohexyl)-3-phenyl-4-(4,4,5,5-tetramethyl-1,3,2-dioxaborolan-2-yl)pentanamide (21b)**

The title compound was prepared from *trans-β*-methylstyrene (0.2 mmol, 26 μL) and (1*r*,4*r*)-4-methoxycyclohexan-1-amine (2.5 equiv., 64.5 mg), according to general procedure. The crude residue was purified by flash chromatography (*n*-pentane/EA = 2:1, Rf = 0.2) to give the product as a white solid (31 mg, 37%).

^{1}H NMR (400 MHz, $CDCl_3$) δ 7.28 – 7.24 (m, 2H), 7.17 (td, *J* = 6.7, 1.4 Hz, 3H), 4.94 (d, *J* = 8.1 Hz, 1H), 3.53 (dtt, *J* = 15.1, 8.0, 4.0 Hz, 1H), 3.27 (s, 3H), 3.08 – 2.95 (m, 2H), 2.61 (dd, *J* = 13.7, 4.1 Hz, 1H), 2.34 (dd, *J* = 13.7, 11.1 Hz, 1H), 1.91 – 1.73 (m, 3H), 1.59 – 1.51 (m, 1H), 1.35 (ddd, *J* = 12.1, 7.4, 3.7 Hz, 1H), 1.26 (d, *J* = 2.9 Hz, 12H), 1.21 – 1.07 (m, 2H), 0.94 – 0.86 (m, 1H), 0.77 (d, *J* = 7.4 Hz, 3H), 0.74 – 0.64 (m, 1H).

^{13}C NMR (101 MHz, $CDCl_3$) δ 171.0, 143.6, 128.4, 127.9, 126.4, 83.3, 78.1, 55.7, 47.2, 45.8, 44.3, 30.3, 30.1, 29.9, 29.8, 24.8, 24.6, 13.9.

^{11}B NMR (128 MHz, $CDCl_3$) δ 35.7.

HRMS (ESI-TOF): calcd for $[M+Na]^+$ $C_{24}H_{38}{}^{11}BNO_4$ 438.2790, found: 438.2794.

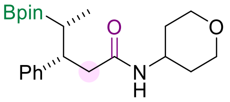

3-Phenyl-*N*-(tetrahydro-2*H*-pyran-4-yl)-4-(4,4,5,5-tetramethyl-1,3,2-dioxaborolan-2-yl)pentanamide (22b)

The title compound was prepared from *trans*-*β*-methylstyrene (0.2 mmol, 26 μL) and tetrahydro-2*H*-pyran-4-amine (2.5 equiv., 50.5 mg), according to general procedure. The crude residue was purified by flash chromatography (*n*-pentane/EA = 1:1, Rf = 0.3) to give the product as a white solid (40 mg, 52%).

^{1}H NMR (300 MHz, $CDCl_3$) δ 7.42 – 7.00 (m, 5H), 5.06 (d, *J* = 8.0 Hz, 1H), 3.74 (ddq, *J* = 16.9, 11.2, 3.5 Hz, 3H), 3.32 (tdd, *J* = 11.8, 9.5, 2.4 Hz, 2H), 3.03 (td, *J* = 10.7, 4.1 Hz, 1H), 2.63 (dd, *J* = 13.6, 4.2 Hz, 1H), 2.36 (dd, *J* = 13.6, 11.1 Hz, 1H), 1.64 (ddq, *J* = 11.6, 4.6, 2.5 Hz, 1H), 1.40 (dddd, *J* = 21.0, 10.8, 5.2, 2.8 Hz, 2H), 1.26 (d, *J* = 1.8 Hz, 12H), 1.20 – 1.10 (m, 1H), 1.04 – 0.93 (m, 1H), 0.77 (d, *J* = 7.4 Hz, 3H).

^{13}C NMR (75 MHz, $CDCl_3$) δ 171.0, 143.5, 128.4, 127.9, 126.5, 83.3, 66.5, 45.8, 45.0, 44.2, 32.7, 32.6, 24.8, 24.6, 13.9.

^{11}B NMR (96 MHz, $CDCl_3$) δ 33.5.

HRMS (ESI-TOF): calcd for $[M+H]^+$ $C_{22}H_{34}{}^{10}BNO_4$ 387.2690, found: 387.2693.

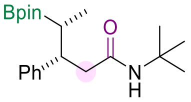

***N*-(*tert*-Butyl)-3-phenyl-4-(4,4,5,5-tetramethyl-1,3,2-dioxaborolan-2-yl)pentanamide (23b)**

The title compound was prepared from *trans*-*β*-methylstyrene (0.2 mmol, 26 μL) and 2-methylpropan-2-amine (2.5 equiv., 36.5 mg), according to general procedure. The crude residue was purified by flash chromatography (*n*-pentane/EA = 5:1, Rf = 0.3) to give the product as a white solid (36 mg, 50%).

^{1}H NMR (300 MHz, $CDCl_3$) δ 7.24 – 7.18 (m, 2H), 7.15 – 7.07 (m, 3H), 4.77 (s, 1H), 2.94 (td, *J* = 10.9, 4.1 Hz, 1H), 2.49 (dd, *J* = 13.4, 4.1 Hz, 1H), 2.21 (dd, *J* = 13.4, 11.3 Hz, 1H), 1.33 – 1.24 (m, 1H), 1.20 (s, 12H), 0.97 (s, 9H), 0.70 (d, *J* = 7.4 Hz, 3H).

^{13}C NMR (75 MHz, $CDCl_3$) δ 171.1, 143.8, 128.3, 128.0, 126.3, 83.2, 50.6, 46.1, 45.3, 28.4, 24.8, 24.6, 13.9.

^{11}B NMR (96 MHz, $CDCl_3$) δ 33.7.

HRMS (ESI-TOF): calcd for $[M+H]^+$ $C_{21}H_{34}{}^{11}BNO_3$ 360.2708, found: 360.2709.

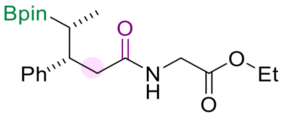

Ethyl-3-phenyl-4-(4,4,5,5-tetramethyl-1,3,2-dioxaborolan-2-yl)pentanoyl)glycinate (24b)

The title compound was prepared from *trans-β*-methylstyrene (0.2 mmol, 26 μL) and ethyl glycinate hydrogen chloride (1.5 equiv., 41.7 mg), according to general procedure B. The crude residue was purified by flash chromatography (*n*-pentane/EA = 4:1, Rf = 0.3) to give the product as a white solid (29 mg, 37%).

^{1}H NMR (300 MHz, $CDCl_3$) δ 7.28 – 7.24 (m, 2H), 7.22 – 7.13 (m, 3H), 5.75 (s, 1H), 4.14 (q, *J* = 7.1 Hz, 2H), 3.97 – 3.67 (m, 2H), 3.12 (td, *J* = 10.2, 4.2 Hz, 1H), 2.71 (dd, *J* = 14.2, 4.2 Hz, 1H), 2.52 (dd, *J* = 14.2, 10.5 Hz, 1H), 1.42 – 1.33 (m, 1H), 1.26 (d, *J* = 2.3 Hz, 12H), 1.24 (t, *J* = 2.1 Hz, 3H), 0.78 (d, *J* = 7.4 Hz, 3H).

^{13}C NMR (75 MHz, $CDCl_3$) δ 171.7, 169.9, 143.7, 128.3, 127.8, 126.4, 83.3, 61.3, 45.2, 43.4, 41.3, 24.9, 24.6, 14.1, 13.9.

^{11}B NMR (96 MHz, $CDCl_3$) δ 33.9.

HRMS (ESI-TOF): calcd for $[M+H]^+$ $C_{21}H_{32}{}^{11}BNO_5$ 390.2450, found: 390.2451.

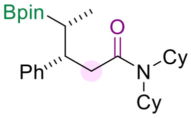

***N,N*-Dicyclohexyl-3-phenyl-4-(4,4,5,5-tetramethyl-1,3,2-dioxaborolan-2-yl)pentanamide (1c)**

The title compound was prepared from *trans-β*-methylstyrene (0.2 mmol, 26 μL) and dicyclohexylamine hydrochloride (1.5 equiv., 65 mg), according to general procedure B. The crude residue was purified by flash chromatography (*n*-pentane/EA = 7:1, Rf = 0.3) to give the product as a white solid (55 mg, 59%).

^{1}H NMR (300 MHz, $CDCl_3$) δ 7.28 – 7.15 (m, 5H), 3.39 (t, *J* = 10.5 Hz, 1H), 3.15 (td, *J* = 9.8, 5.4 Hz, 1H), 2.80 – 2.46 (m, 3H), 2.31 – 2.24 (m, 1H), 1.90 – 1.30 (m, 12H), 1.28 (s, 12H), 1.22 – 1.00 (m, 8H), 0.79 (d, *J* = 7.4 Hz, 3H).

^{13}C NMR (75 MHz, $CDCl_3$) δ 171.1, 144.0, 128.2, 128.0, 125.9, 82.9, 58.0, 56.0, 45.6, 41.3, 31.5, 30.9, 29.9, 29.8, 26.6, 26.6, 25.9, 25.2, 24.9, 24.7, 14.1.

^{11}B NMR (96 MHz, $CDCl_3$) δ 32.5.

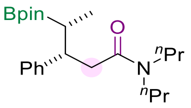

3-Phenyl-*N,N*-dipropyl-4-(4,4,5,5-tetramethyl-1,3,2-dioxaborolan-2-yl)pentanamide (2c)

The title compound was prepared from *trans-β*-methylstyrene (0.2 mmol, 26 μL) and dipropylamine hydrochloride (1.5 equiv., 41 mg), according to general procedure. The crude residue was purified by flash chromatography (*n*-pentane/EA = 7:1, Rf = 0.3) to give the product as a white solid (45 mg, 58%).

^{1}H NMR (300 MHz, $CDCl_3$) δ 7.31 – 7.20 (m, 1H), 7.18 – 7.11 (m, 4H), 3.28 – 3.05 (m, 2H), 2.94 (qt, *J* = 14.9, 8.5 Hz, 3H), 2.74 – 2.48 (m, 2H), 1.44 – 1.21 (m, 5H), 1.20 (s, 12H), 0.78 (t, *J* = 7.4 Hz, 3H), 0.69 (dd, *J* = 13.2, 7.4 Hz, 6H).

^{13}C NMR (75 MHz, $CDCl_3$) δ 171.7, 144.3, 128.1, 128.1, 126.1, 83.0, 49.7, 47.8, 45.1, 39.2, 24.9, 24.8, 22.1, 20.7, 14.1, 11.2.

^{11}B NMR (96 MHz, $CDCl_3$) δ 31.4.

HRMS (ESI-TOF): calcd for $[M+H]^+$ $C_{23}H_{38}{}^{11}BNO_3$ 388.3022, found: 388.3028.

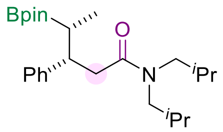

_N,N_-Diisobutyl-3-phenyl-4-(4,4,5,5-tetramethyl-1,3,2-dioxaborolan-2-yl)pentanamide (3c)

The title compound was prepared from *trans-β*-methylstyrene (0.2 mmol, 26 μL) and diisobutylamine hydrochloride (1.5 equiv., 49.5 mg), according to general procedure. The crude residue was purified by flash chromatography (*n*-pentane/EA = 7:1, Rf = 0.3) to give the product as a white solid (51 mg, 61%).

^1H NMR (300 MHz, $CDCl_3$) δ 7.24 – 7.16 (m, 4H), 7.11 (ddd, *J* = 8.5, 5.3, 2.1 Hz, 1H), 3.39 – 3.14 (m, 2H), 3.04 (dd, *J* = 14.6, 7.3 Hz, 1H), 2.87 – 2.68 (m, 3H), 2.58 (dd, *J* = 14.6, 4.6 Hz, 1H), 1.76 (dhept, *J* = 27.5, 6.9 Hz, 2H), 1.36 (d, *J* = 3.7 Hz, 1H), 1.24 (s, 12H), 0.84 (dd, *J* = 6.7, 1.7 Hz, 6H), 0.77 (d, *J* = 7.4 Hz, 3H), 0.60 (dd, *J* = 6.7, 1.4 Hz, 6H).

^{13}C NMR (75 MHz, $CDCl_3$) δ 172.0, 144.3, 128.0, 128.0, 125.9, 82.9, 55.8, 53.5, 45.2, 39.3, 28.0, 26.4, 24.9, 24.7, 20.0, 19.9, 14.1.

^{11}B NMR (96 MHz, $CDCl_3$) δ 32.6.

HRMS (ESI-TOF): calcd for $[M+H]^+$ $C_{25}H_{42}{}^{11}BNO_3$ 416.3335, found: 416.3335.

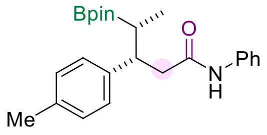

_N_-Phenyl-4-(4,4,5,5-tetramethyl-1,3,2-dioxaborolan-2-yl)-3-(_p_-tolyl)pentanamide (1d)

The title compound was prepared from *(E)*-1-methyl-4-(prop-1-en-1-yl)benzene (0.2 mmol, 26.4 mg) and aniline (2.5 equiv., 45 μL), according to general procedure. The crude residue was purified by flash chromatography (*n*-pentane/EA = 10:1, Rf = 0.2) to give the product as a white solid (37 mg, 47%).

^1H NMR (400 MHz, $CDCl_3$) δ 7.27 – 7.20 (m, 4H), 7.11 (s, 5H), 7.05 – 7.00 (m, 1H), 3.11 (td, *J* = 10.2, 4.1 Hz, 1H), 2.77 (dd, *J* = 14.1, 4.1 Hz, 1H), 2.61 (dd, *J* = 14.1, 10.2 Hz, 1H), 2.31 (s, 3H), 1.46 – 1.39 (m, 1H), 1.29 (d, *J* = 2.3 Hz, 12H), 0.80 (d, *J* = 7.4 Hz, 3H).

^{13}C NMR (101 MHz, $CDCl_3$) δ 170.2, 140.5, 137.9, 136.1, 129.3, 128.7, 127.7, 123.9, 119.7, 83.4, 45.3, 45.3, 24.9, 24.6, 21.0, 14.0.

^{11}B NMR (128 MHz, $CDCl_3$) δ 33.4.

HRMS (ESI-TOF): calcd for $[M+H]^+$ $C_{24}H_{32}{}^{11}BNO_3$ 394.2552, found: 394.2560.

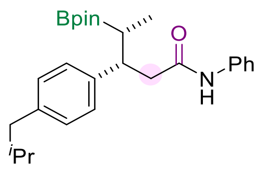

3-(4-Isobutylphenyl)-*N*-phenyl-4-(4,4,5,5-tetramethyl-1,3,2-dioxaborolan-2-yl)pentanamide (2d)

The title compound was prepared from *(E)*-1-isobutyl-4-(prop-1-en-1-yl)benzene (0.2 mmol, 34.8 mg) and aniline (2.5 equiv., 45 μL), according to general procedure. The crude residue was purified by flash chromatography (*n*-pentane/EA = 10:1, Rf = 0.2) to give the product as a white solid (51 mg, 58%).

^{1}H NMR (400 MHz, $CDCl_3$) δ 7.20 (d, *J* = 4.3 Hz, 4H), 7.14 (d, *J* = 8.0 Hz, 2H), 7.08 (d, *J* = 8.1 Hz, 2H), 7.01 (td, *J* = 8.4, 4.1 Hz, 2H), 3.12 (td, *J* = 10.2, 4.1 Hz, 1H), 2.78 (dd, *J* = 14.0, 4.1 Hz, 1H), 2.62 (dd, *J* = 14.0, 10.4 Hz, 1H), 2.43 (d, *J* = 7.2 Hz, 2H), 1.83 (dq, *J* = 13.5, 6.8 Hz, 1H), 1.43 (dt, *J* = 9.9, 7.5 Hz, 1H), 1.28 (d, *J* = 3.0 Hz, 12H), 0.87 (d, *J* = 1.6 Hz, 3H), 0.86 (d, *J* = 1.6 Hz, 3H), 0.81 (d, *J* = 7.4 Hz, 3H).

^{13}C NMR (101 MHz, $CDCl_3$) δ 170.2, 140.8, 140.0, 137.8, 129.4, 128.7, 127.6, 123.9, 119.7, 83.4, 45.3, 45.3, 45.0, 30.2, 24.9, 24.6, 22.3, 22.3, 13.9.

^{11}B NMR (96 MHz, $CDCl_3$) δ 30.2.

HRMS (ESI-TOF): calcd for $[M+Na]^+$ $C_{27}H_{38}{}^{10}BNO_3$ 457.2873, found: 457.2878.

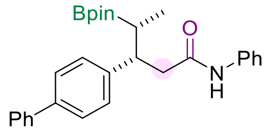

3-([1,1'-Biphenyl]-4-yl)-*N*-phenyl-4-(4,4,5,5-tetramethyl-1,3,2-dioxaborolan-2-yl)pentanamide (3d)

The title compound was prepared from *(E)*-4-(prop-1-en-1-yl)-1,1'-biphenyl (0.2 mmol, 38.8 mg) and aniline (2.5 equiv., 45 μL), according to general procedure. The crude residue was purified by flash chromatography (*n*-pentane/EA = 10:1, Rf = 0.2) to give the product as a white solid (46 mg, 51%).

^{1}H NMR (300 MHz, $CDCl_3$) δ 7.56 (ddd, *J* = 10.8, 7.7, 1.6 Hz, 4H), 7.46 – 7.39 (m, 2H), 7.32 (tt, *J* = 8.2, 1.7 Hz, 3H), 7.27 – 7.16 (m, 4H), 7.13 – 6.96 (m, 2H), 3.22 (td, *J* = 10.1, 4.1 Hz, 1H), 2.84 (dd, *J* = 14.0, 4.1 Hz, 1H), 2.65 (dd, *J* = 14.0, 10.2 Hz, 1H), 1.50 (dd, *J* = 9.9, 7.5 Hz, 1H), 1.30 (d, *J* = 1.4 Hz, 12H), 0.87 (d, *J* = 7.4 Hz, 3H).

^{13}C NMR (75 MHz, $CDCl_3$) δ 170.0, 142.8, 140.9, 139.4, 137.8, 128.8, 128.7, 128.3, 127.3, 127.1, 127.0, 123.9, 119.7, 83.4, 45.4, 45.3, 24.9, 24.7, 14.1.

^{11}B NMR (96 MHz, $CDCl_3$) δ 33.7.

HRMS (ESI-TOF): calcd for $[M+H]^+$ $C_9H_{34}{}^{11}BNO_3$ 456.2710, found: 456.2711.

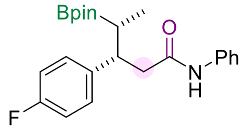

3-(4-Fluorophenyl)-*N*-phenyl-4-(4,4,5,5-tetramethyl-1,3,2-dioxaborolan-2-yl)pentanamide (4d)

The title compound was prepared from *(E)*-1-fluoro-4-(prop-1-en-1-yl)benzene (0.2 mmol, 27.2 mg) and aniline (2.5 equiv., 45 μL), according to general procedure. The crude residue was purified by flash chromatography (*n*-pentane/EA = 10:1, Rf = 0.2) to give the product as a white solid (34 mg, 43%).

^{1}H NMR (300 MHz, $CDCl_3$) δ 7.31 – 7.15 (m, 6H), 7.15 – 7.01 (m, 2H), 7.00 – 6.90 (m, 2H), 3.17 (td, *J* = 10.1, 4.2 Hz, 1H), 2.79 (dd, *J* = 14.0, 4.2 Hz, 1H), 2.55 (dd, *J* = 14.0, 10.1 Hz, 1H), 1.42 (dd, *J* = 10.3, 7.2 Hz, 1H), 1.28 (d, *J* = 1.1 Hz, 12H), 0.80 (d, *J* = 7.4 Hz, 3H).

^{13}C NMR (75 MHz, $CDCl_3$) δ 169.8, 161.5 (d, *J* = 244.4 Hz), 139.3 (d, *J* = 3.2 Hz), 137.7, 129.21 (d, *J* = 7.8 Hz), 128.9, 124.1, 119.7, 115.3 (d, *J* = 21.1 Hz), 83.5, 45.2, 44.9, 24.9, 24.7, 13.9.

^{11}B NMR (96 MHz, $CDCl_3$) δ 33.8.

^{19}F NMR (282 MHz, $CDCl_3$) δ -116.5.

HRMS (ESI-TOF): calcd for $[M+Na]^+$ $C_{23}H_{29}{}^{11}BFNO_3$ 420.2121, found: 420.2124.

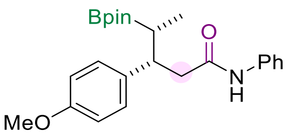

3-(4-Methoxyphenyl)-*N*-phenyl-4-(4,4,5,5-tetramethyl-1,3,2-dioxaborolan-2-yl)pentanamide (5d)

The title compound was prepared from *(E)*-1-methoxy-4-(prop-1-en-1-yl)benzene (0.2 mmol, 29.6 mg) and aniline (2.5 equiv., 45 µL), according to general procedure. The crude residue was purified by flash chromatography (*n*-pentane/EA = 5:1, Rf = 0.3) to give the product as a white solid (28 mg, 34%).

^{1}H NMR (300 MHz, $CDCl_3$) δ 7.28 – 7.19 (m, 4H), 7.15 (d, *J* = 8.6 Hz, 2H), 7.02 (t, *J* = 6.6 Hz, 2H), 6.88 – 6.75 (m, 2H), 3.77 (s, 3H), 3.10 (td, *J* = 10.2, 4.0 Hz, 1H), 2.77 (dd, *J* = 14.0, 4.0 Hz, 1H), 2.58 (dd, *J* = 14.0, 10.3 Hz, 1H), 1.44 – 1.36 (m, 1H), 1.28 (d, *J* = 1.5 Hz, 12H), 0.80 (d, *J* = 7.4 Hz, 3H).

^{13}C NMR (75 MHz, $CDCl_3$) δ 170.1, 158.2, 137.9, 135.6, 128.8, 123.9, 119.7, 114.0, 83.4, 55.2, 45.5, 44.9, 24.9, 24.7, 13.9.

^{11}B NMR (96 MHz, $CDCl_3$) δ 33.9.

HRMS (ESI-TOF): calcd for $[M+Na]^+$ $C_{24}H_{32}{}^{10}BNO_4$ 431.2353, found: 431.2363.

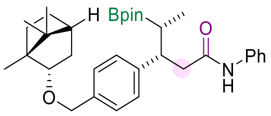

***N*-Phenyl-4-(4,4,5,5-tetramethyl-1,3,2-dioxaborolan-2-yl)-3-(4-((((1*S*,2*R*,4*R*)-1,7,7-trimethylbicyclo[2.2.1]heptan-2-yl)oxy)methyl)phenyl)pentanamide (6d)**

The title compound was prepared from (1*S*,2*R*,4*R*)-1,7,7-trimethyl-2-((4-((*E*)-prop-1-en-1-yl)benzyl)oxy)bicyclo[2.2.1]heptane (0.2 mmol, 56 mg) and aniline (2.5 equiv., 45 µL), according to general procedure. The crude residue was purified by flash chromatography (*n*-pentane/EA = 5:1, Rf = 0.2) to give the product as a white solid (47 mg, 43%).

^{1}H NMR (300 MHz, $CDCl_3$) δ 7.29 – 7.18 (m, 8H), 7.07 – 6.95 (m, 2H), 4.55 – 4.38 (m, 2H), 3.67 (ddd, *J* = 9.4, 3.3, 1.8 Hz, 1H), 3.15 (td, *J* = 10.1, 4.0 Hz, 1H), 2.79 (dd, *J* = 14.0, 4.0 Hz, 1H), 2.61 (dd, *J* = 14.0, 10.2 Hz, 1H), 2.14 – 2.03 (m, 2H), 1.67 (ddd, *J* = 22.0, 8.5, 4.5 Hz, 3H), 1.49 – 1.42 (m, 1H), 1.29 – 1.28 (m, 12H), 1.07 (dd, *J* = 12.8, 3.1 Hz, 2H), 0.88 – 0.80 (m, 12H).

^{13}C NMR (75 MHz, $CDCl_3$) δ 170.0, 142.6, 137.9, 137.9, 128.8, 127.7, 127.5, 123.9, 119.7, 84.4, 83.4, 71.4, 49.3, 47.8, 45.5, 45.0, 36.1, 31.5, 30.1, 29.7, 28.2, 26.7, 24.9, 24.7, 19.8, 18.9, 14.0.

^{11}B NMR (96 MHz, $CDCl_3$) δ 32.8.

HRMS (ESI-TOF): calcd for $[M+Na]^+$ $C_{34}H_{48}{}^{11}BNO_4$ 568.3574, found: 568.3581.

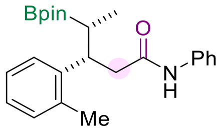

N-Phenyl-4-(4,4,5,5-tetramethyl-1,3,2-dioxaborolan-2-yl)-3-(o-tolyl)pentanamide (7d)

The title compound was prepared from *(E)*-1-methyl-2-(prop-1-en-1-yl)benzene (0.2 mmol, 26.4 mg) and aniline (2.5 equiv., 45 µL), according to general procedure. The crude residue was purified by flash chromatography (*n*-pentane/EA = 10:1, Rf = 0.2) to give the product as a white solid (51 mg, 65%).

^{1}H NMR (300 MHz, $CDCl_3$) δ 7.26 – 7.18 (m, 6H), 7.11 (dd, *J* = 5.5, 1.9 Hz, 2H), 7.01 (ddd, *J* = 8.6, 5.9, 2.7 Hz, 1H), 6.89 (s, 1H), 3.48 (td, *J* = 10.4, 3.9 Hz, 1H), 2.80 (dd, *J* = 13.7, 3.9 Hz, 1H), 2.59 (dd, *J* = 13.7, 10.3 Hz, 1H), 2.33 (s, 3H), 1.48 – 1.40 (m, 1H), 1.30 (s, 12H), 0.81 (d, *J* = 7.4 Hz, 3H).

^{13}C NMR (75 MHz, $CDCl_3$) δ 170.2, 142.6, 137.9, 137.2, 130.5, 128.8, 126.5, 126.1, 125.3, 123.8, 119.6, 83.4, 45.4, 40.1, 24.9, 24.6, 20.1, 14.0.

^{11}B NMR (96 MHz, $CDCl_3$) δ 33.9.

HRMS (ESI-TOF): calcd for $[M+H]^+$ $C_{24}H_{32}{}^{11}BNO_3$ 394.2552, found: 394.2556.

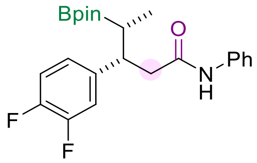

3-(3,4-Difluorophenyl)-N-phenyl-4-(4,4,5,5-tetramethyl-1,3,2-dioxaborolan-2-yl)pentanamide (8d)

The title compound was prepared from *(E)*-1,2-difluoro-4-(prop-1-en-1-yl)benzene (0.2 mmol, 30 mg) and aniline (2.5 equiv., 45 µL), according to general procedure. The crude residue was purified by flash chromatography (*n*-pentane/EA = 10:1, Rf = 0.2) to give the product as a white solid (39 mg, 47%).

^{1}H NMR (300 MHz, $CDCl_3$) δ 7.36 – 7.23 (m, 5H), 7.05 (qd, *J* = 8.3, 3.6 Hz, 3H), 6.94 (d, *J* = 2.0 Hz, 1H), 3.19 (td, *J* = 9.8, 4.3 Hz, 1H), 2.80 (dd, *J* = 14.2, 4.3 Hz, 1H), 2.53 (dd, *J* = 14.2, 9.9 Hz, 1H), 1.42 – 1.34 (m, 1H), 1.28 (s, 12H), 0.81 (d, *J* = 7.4 Hz, 3H).

^{13}C NMR (75 MHz, $CDCl_3$) δ 169.5, 148.9 (d, *J* = 259.2 Hz), 145.9, 140.9 (t, *J* = 4.3 Hz), 139.3 (d, *J* = 242.7 Hz), 128.9, 124.2, 123.9, (dd, *J* = 6.1, 3.3 Hz), 119.8, 117.0 (d, *J* = 16.5 Hz), 116.3 (d, *J* = 16.9 Hz), 83.6, 44.8, 44.6, 24.9, 24.7, 13.9.

^{11}B NMR (96 MHz, $CDCl_3$) δ 33.2.

^{19}F NMR (282 MHz, $CDCl_3$) δ -136.48 – -138.67 (m), -139.95 – -141.22 (m).

HRMS (ESI-TOF): calcd for $[M+Na]^+$ $C_{23}H_{28}{}^{10}BF_2NO_3$ 437.2059, found: 437.2069.

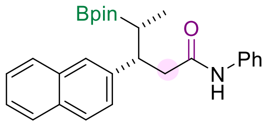

3-(Naphthalen-2-yl)-N-phenyl-4-(4,4,5,5-tetramethyl-1,3,2-dioxaborolan-2-yl)pentanamide (9d)

The title compound was prepared from *(E)*-2-(prop-1-en-1-yl)naphthalene (0.2 mmol, 33.6 mg) and aniline (2.5 equiv., 45 µL), according to general procedure. The crude residue was purified by flash chromatography (*n*-pentane/EA = 10:1, Rf = 0.2) to give the product as a white solid (45 mg, 53%).

^{1}H NMR (300 MHz, $CDCl_3$) δ 7.83 – 7.73 (m, 3H), 7.68 (s, 1H), 7.47 – 7.36 (m, 3H), 7.25 – 7.10 (m, 4H), 7.07 (s, 1H), 6.99 (dq, J = 8.6, 4.2 Hz, 1H), 3.35 (td, J = 9.9, 4.2 Hz, 1H), 2.88 (dd, J = 14.2, 4.2 Hz, 1H), 2.73 (dd, J = 14.1, 9.9 Hz, 1H), 1.60 – 1.51 (m, 1H), 1.30 (d, J = 2.0 Hz, 12H), 0.83 (d, J = 7.4 Hz, 3H).

^{13}C NMR (75 MHz, $CDCl_3$) δ 170.0, 141.2, 137.8, 133.5, 132.4, 128.7, 128.4, 127.7, 127.6, 126.9, 126.0, 125.6, 125.4, 123.9, 119.7, 83.5, 45.8, 45.2, 24.9, 24.7, 14.1.

^{11}B NMR (96 MHz, $CDCl_3$) δ 33.6.

HRMS (ESI-TOF): calcd for $[M+Na]^+$ $C_{27}H_{32}{}^{10}BNO_3$ 451.2404, found: 451.2409.

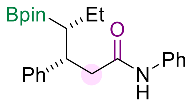

N,3-Diphenyl-4-(4,4,5,5-tetramethyl-1,3,2-dioxaborolan-2-yl)hexanamide (10d)

The title compound was prepared from *(E)*-but-1-en-1-ylbenzene (0.2 mmol, 26.4 mg) and aniline (2.5 equiv., 45 μL), according to general procedure. The crude residue was purified by flash chromatography (*n*-pentane/EA = 10:1, Rf = 0.2) to give the product as a white solid (33 mg, 42%).

^{1}H NMR (300 MHz, $CDCl_3$) δ 7.34 – 7.27 (m, 2H), 7.25 – 7.15 (m, 7H), 7.01 (dt, J = 12.5, 6.2 Hz, 2H), 3.22 (td, J = 10.3, 3.9 Hz, 1H), 2.77 (dd, J = 13.9, 3.9 Hz, 1H), 2.58 (dd, J = 13.9, 10.5 Hz, 1H), 1.54 – 1.35 (m, 1H), 1.32 (s, 12H), 1.23 (d, J = 6.3 Hz, 2H), 0.83 (t, J = 7.3 Hz, 3H).

^{13}C NMR (75 MHz, $CDCl_3$) δ 170.0, 144.1, 137.8, 128.8, 128.7, 127.8, 126.7, 124.0, 119.8, 83.5, 45.6, 44.5, 25.0, 24.9, 22.7, 13.4.

^{11}B NMR (96 MHz, $CDCl_3$) δ 33.3.

HRMS (ESI-TOF): calcd for $[M+H]^+$ $C_{24}H_{32}{}^{10}BNO_3$ 393.2585, found: 393.2596.

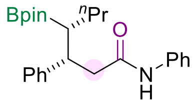

N,3-diphenyl-4-(4,4,5,5-tetramethyl-1,3,2-dioxaborolan-2-yl)heptanamide (11d)

The title compound was prepared from *(E)*-pent-1-en-1-ylbenzene (0.2 mmol, 29.2 mg) and aniline (2.5 equiv., 45 μL), according to general procedure. The crude residue was purified by flash chromatography (*n*-pentane/EA = 10:1, Rf = 0.2) to give the product as a white solid (43 mg, 53%).

^{1}H NMR (300 MHz, $CDCl_3$) δ 7.33 – 7.27 (m, 2H), 7.25 – 7.16 (m, 7H), 7.01 (dt, J = 12.0, 5.9 Hz, 2H), 3.20 (td, J = 10.3, 3.9 Hz, 1H), 2.76 (dd, J = 13.9, 3.9 Hz, 1H), 2.59 (dd, J = 13.9, 10.5 Hz, 1H), 1.49 – 1.39 (m, 1H), 1.31 (s, 12H), 1.23 (d, J = 2.8 Hz, 2H), 1.14 (ddd, J = 17.6, 8.4, 3.6 Hz, 2H), 0.77 (t, J = 6.9 Hz, 3H).

^{13}C NMR (75 MHz, $CDCl_3$) δ 169.9, 144.1, 137.8, 128.7, 128.7, 127.7, 126.6, 123.9, 119.7, 83.4, 45.5, 44.7, 32.0, 25.0, 24.8, 22.1, 14.3.

^{11}B NMR (96 MHz, $CDCl_3$) δ 33.0.

HRMS (ESI-TOF): calcd for $[M+Na]^+$ $C_{25}H_{34}{}^{10}BNO_3$ 429.2560, found: 429.2566.

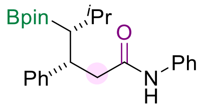

5-Methyl-*N*,3-diphenyl-4-(4,4,5,5-tetramethyl-1,3,2-dioxaborolan-2-yl)hexanamide (12d)

The title compound was prepared from *(E)*-(3-methylbut-1-en-1-yl)benzene (0.2 mmol, 29.2 mg) and aniline (2.5 equiv., 45 μL), according to general procedure. The crude residue was purified by flash chromatography (*n*-pentane/EA = 10:1, Rf = 0.2) to give the product as a white solid (42 mg, 51%).

^{1}H NMR (300 MHz, $CDCl_3$) δ 7.35 – 7.26 (m, 2H), 7.26 – 7.16 (m, 7H), 7.07 – 6.95 (m, 1H), 6.86 (s, 1H), 3.33 (td, *J* = 10.9, 3.5 Hz, 1H), 2.72 (dd, *J* = 13.7, 3.5 Hz, 1H), 2.51 (dd, *J* = 13.7, 10.7 Hz, 1H), 1.50 – 1.37 (m, 2H), 1.33 (d, *J* = 2.9 Hz, 12H), 0.88 (dd, *J* = 15.8, 6.8 Hz, 6H).

^{13}C NMR (75 MHz, $CDCl_3$) δ 169.9, 143.9, 137.8, 128.8, 128.7, 127.7, 126.6, 123.9, 119.7, 83.5, 46.2, 43.0, 27.2, 25.3, 25.0, 23.4, 19.1.

^{11}B NMR (96 MHz, $CDCl_3$) δ 33.7.

HRMS (ESI-TOF): calcd for $[M+Na]^+$ $C_{25}H_{34}{}^{11}BNO_3$ 430.2528, found: 430.2533.

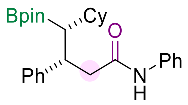

4-Cyclohexyl-*N*,3-diphenyl-4-(4,4,5,5-tetramethyl-1,3,2-dioxaborolan-2-yl)butanamide (13d)

The title compound was prepared from *(E)*-(2-cyclohexylvinyl)benzene (0.2 mmol, 37.2 mg) and aniline (2.5 equiv., 45 μL), according to general procedure. The crude residue was purified by flash chromatography (*n*-pentane/EA = 10:1, Rf = 0.2) to give the product as a white solid (43 mg, 48%).

^{1}H NMR (300 MHz, $CDCl_3$) δ 7.32 – 7.26 (m, 2H), 7.26 – 7.10 (m, 7H), 7.01 (dt, *J* = 8.6, 4.2 Hz, 1H), 6.94 (s, 1H), 3.39 (td, *J* = 10.8, 3.6 Hz, 1H), 2.71 (dd, *J* = 13.8, 3.6 Hz, 1H), 2.53 (dd, *J* = 13.8, 10.7 Hz, 1H), 1.64 – 1.51 (m, 4H), 1.33 (d, *J* = 4.5 Hz, 12H), 1.17 – 0.80 (m, 8H).

^{13}C NMR (75 MHz, $CDCl_3$) δ 170.0, 144.0, 137.8, 128.7, 128.7, 127.6, 126.5, 123.9, 119.7, 83.5, 45.8, 42.0, 37.3, 33.8, 30.3, 26.7, 26.7, 26.4, 25.2, 25.0.

^{11}B NMR (96 MHz, $CDCl_3$) δ 31.0.

HRMS (ESI-TOF): calcd for $[M+H]^+$ $C_{28}H_{38}{}^{11}BNO_3$ 448.3023, found: 448.3025.

5. Derivatization of 1a.

5.1 Oxidation of **1a**.

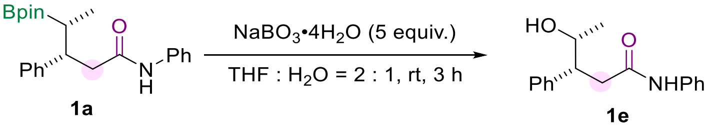

The title compound **1e** was synthesized according to the following literature[1]: A 25 mL flask charged with **1a** (0.1 mmol) and THF/H_2O (1.0 mL/0.5 mL). Then the $NaBO_3$•$4H_2O$ (5 equiv.) was added to a solution. The resulting mixture was stirred vigorously for 3 h at room temperature, then quenched with water and extracted with ethyl acetate (5 mL). The combined organic layers were washed with brine (15 mL), dried over Na_2SO_4 and concentrated. Purification by column chromatography on silica gel gave the corresponding product **1e** as a colorless oil (98% yield).

4-Hydroxy-*N*,3-diphenylpentanamide (1e)

^{1}H NMR (300 MHz, $CDCl_3$) δ 7.39 – 7.34 (m, 2H), 7.32 – 7.24 (m, 5H), 7.23 – 7.19 (m, 2H), 7.09 (td, *J* = 6.2, 2.8 Hz, 1H), 4.01 (s, 1H), 3.12 (dt, *J* = 8.4, 6.3 Hz, 1H), 2.98 (dd, *J* = 14.6, 5.9 Hz, 1H), 2.75 (dd, *J* = 14.6, 6.6 Hz, 1H), 2.64 (d, *J* = 5.7 Hz, 1H), 1.10 (d, *J* = 6.2 Hz, 3H).

^{13}C NMR (75 MHz, $CDCl_3$) δ 171.4, 142.3, 137.7, 128.8, 128.7, 127.9, 126.9, 124.3, 120.2, 71.7, 50.8, 41.4, 22.1.

5.2 Vinylation of **1a**.

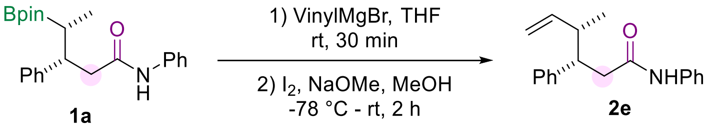

The title compound **2e** was synthesized according to the modified following literature[2]: an oven-dried round bottom flask was charged with a stirring bar, **1a** (0.1 mmol) in THF (1 mL). Vinylmagnesium bromide (1 M, 0.4 mL, 4.0 equiv.) was added dropwise to the mixture at room temperature. The resulting mixture was stirred for 0.5 h and then cooled to -78 °C. A solution of I_2 (4.0 equiv.) in methanol (1.0 mL) was added dropwise and the mixture was allowed to stir 0.5 h at the same temperature. A solution of NaOMe (8.0 equiv.) in methanol (1.5 mL) was added dropwise. Then the reaction mixture was warmed to room temperature and stirred for another 1.5 h. The reaction mixture was quenched by saturated $Na_2S_2O_3$ (2 mL) and diluted with EtOAc (10 mL) and water 10 mL. The combined organic layer was dried over Na_2SO_4 and concentrated under reduced pressure. The crude product was purified by column chromatography on silica gel to afford the corresponding product **2e** as a colorless oil (93%).

4-Methyl-*N*,3-diphenylhex-5-enamide (2e)

^{1}H NMR (300 MHz, $CDCl_3$) δ 7.36 – 7.28 (m, 2H), 7.27 – 7.10 (m, 7H), 7.06 – 6.90 (m, 1H), 6.79 (s, 1H), 5.75 (ddd, *J* = 17.1, 10.1, 8.9 Hz, 1H), 5.19 – 4.98 (m, 2H), 2.99 (td, *J* = 9.9, 4.3 Hz, 1H), 2.89 (dd, *J* = 14.3, 4.3 Hz, 1H), 2.57 – 2.32 (m, 2H), 0.84 (d, *J* = 6.7 Hz, 3H).

^{13}C NMR (75 MHz, $CDCl_3$) δ 170.2, 142.8, 142.7, 137.6, 128.8, 128.7, 128.1, 126.8, 124.1, 119.9, 115.1, 48.1, 44.3, 43.5, 19.1.

HRMS (ESI-TOF): calcd for $[M+Na]^+$ $C_{19}H_{21}NO$ 302.1515, found: 302.1518.

5.3 Preparation of trifluoroboration salt of **1a.**

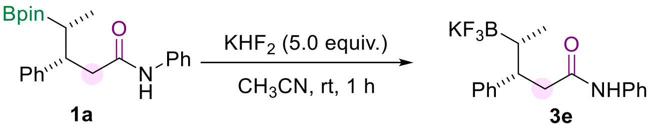

The title compound **3e** was prepared according to the following literature[4]: an oven-dried 25 mL Schlenk tube was charged with **1a** (0.1 mmol) and MeCN (0.2 mL). The reaction mixture was stirred vigorously for 1 h at room temperature after KHF_2 (4.0 equiv.) was added. The resulting slurry was stirred concentrated, then placed under high vacuum for 1 h. The dried solids were triturated with hot acetone and filtered to remove inorganic salts. The combined organic layers were concentrated and Et_2O (5 mL) was added, then the mixture was sonicated for 15 minutes, and the organic layer was removed, and this step was repeated. The trifluoroboration salt **4e** was obtained as a white solid (82% yield).

N,3-Diphenyl-4-(trifluoro-λ4-boraneyl)pentanamide, potassium salt (3e)

^{1}H NMR (300 MHz, DMSO) δ 9.38 (s, 1H), 7.38 (d, J = 7.6 Hz, 2H), 7.22 – 7.05 (m, 6H), 6.98 (tt, J = 6.3, 2.9 Hz, 1H), 6.90 (t, J = 7.3 Hz, 1H), 3.09 (dd, J = 14.9, 3.9 Hz, 1H), 2.85 (td, J = 10.9, 3.7 Hz, 1H), 2.50 – 2.42 (m, 1H), 0.40 (s, 3H).

^{13}C NMR (75 MHz, DMSO) δ 171.7, 148.0, 139.5, 128.3, 128.2, 127.2, 124.5, 122.4, 118.9, 45.8, 43.4, 15.9.

^{11}B NMR (96 MHz, DMSO) δ 6.6.

5.4 *N*-Arylation of **1a**.

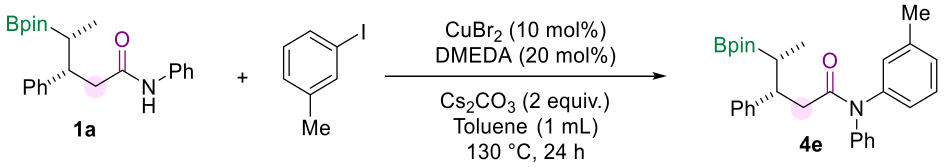

The title compound **4e** was prepared according to the following literature[3]: an oven-dried 25 mL Schlenk tube was charged with **1a** (0.1 mmol), $CuBr_2$ (10 mol%), and Cs_2CO_3 (2.0 equiv.). The tube was evacuated under vacuum and recharged with argon for three times. Then 1-iodo-3-methylbenzene (2.0 equiv.), 1,2-dimethylethylenediamine (DMEDA, 20 mol%) , and toluene (1 mL) was injected into the tube. The resulted mixture was allowed to stir at 130 °C for 24 h. Then the reaction mixture was cooled to room temperature. Then the solution was extracted by EtOAc (5 mLx2) and dried over Na_2SO_4. The combined organic layer was concentrated under reduced pressure and purified by column chromatography to give the corresponding **3e** in 61% yield.

N,3-Diphenyl-4-(4,4,5,5-tetramethyl-1,3,2-dioxaborolan-2-yl)-N-(m-tolyl)pentanamide (4e)

^{1}H NMR (300 MHz, $CDCl_3$) δ 7.29 – 7.09 (m, 6H), 7.11 – 6.95 (m, 4H), 6.80 (d, J = 7.3 Hz, 2H), 6.55 (s, 2H), 3.20 (dt, J = 9.9, 7.6 Hz, 1H), 2.56 (d, J = 8.0 Hz, 2H), 2.19 (s, 3H), 1.19 (d, J = 6.1 Hz, 1H), 1.14 (s, 12H), 0.67 (d, J = 7.4 Hz, 3H).

^{13}C NMR (75 MHz, $CDCl_3$) δ 172.0, 144.0, 143.0, 142.8, 129.0, 128.9, 128.8, 128.7, 128.7, 128.6, 128.4, 128.0, 126.1, 83.0, 45.8, 41.1, 24.8, 24.6, 21.2, 14.1.

^{11}B NMR (96 MHz, $CDCl_3$) δ 33.3.

HRMS (ESI-TOF): calcd for $[M+H]^+$ $C_{30}H_{36}{}^{11}BNO_3$ 470.2867, found: 470.2872.

6. Mechanism studies.

6.1 ^{13}C labeling experiment.

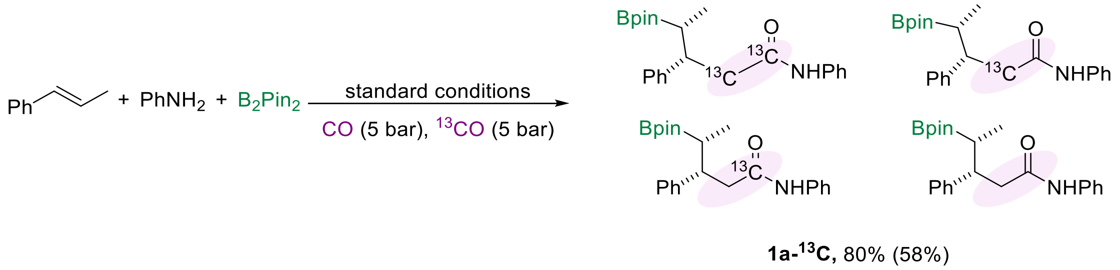

A dried vial (4 mL) was charged with $Cu(OAc)_2$ (5 mol%), DPPE (5 mol%), B_2pin_2 (3.5 equiv.), and a stirring bar. When the NaOEt (2.75 equiv.) is added, the vial was sealed by PTFE/white rubber septum (Wheaton 13 mm Septa) and phenolic cap immediately. The vial was evacuated under vacuum and recharged with argon for three

times with a needle. Then, *trans-β*-methylstyrene (0.2 mmol), aniline (2.5 equiv.) and DMSO (1.0 mL) were added under argon by using a syringe. The vial (or several vials) was placed in an alloy plate and transferred into a 300 mL autoclave of the 4560 series from Parr Instruments. The autoclave was charged with ^{13}CO (5 bar) and CO (5 bar) after flushing two times with N_2 and two times with CO. The autoclave was then placed into an aluminum block on a magnetic stirrer. The reaction was performed for 20 h at 60 °C. After the reaction was complete, the autoclave was cooled down to room temperature and the pressure was released carefully. The reaction was quenched with water (2 mL) and extracted with EtOAc (2 mLx3). The organic layer was washed with water (10 mL) and dried over anhydrous Na_2SO_4 and concentrated under reduced pressure. After that the corresponding products **1a-^{13}C** was afforded as a white solid in 80% NMR yield and 58% isolated yield by directly purified by fast column chromatography. (For NMR yield: The organic layer was washed with water and 0.1 mmol of 1,3,5-trimethoxybenzene was added. After drying with anhydrous Na_2SO_4, an appropriate amount of organic layer was concentrated under reduced pressure to test the NMR yield.)

^{1}H NMR (300 MHz, $CDCl_3$) δ 7.32 – 7.16 (m, 10H), 7.05 – 6.94 (m, 1H), 3.18 (ddd, *J* = 10.0, 6.0, 2.0 Hz, 1H), 3.07 – 2.72 (m, 1H), 2.68 – 2.33 (m, 1H), 1.46 (dd, *J* = 15.5, 7.3 Hz, 1H), 1.27 (d, *J* = 2.1 Hz, 12H), 0.81 (d, *J* = 7.4 Hz, 3H).

^{13}C NMR (75 MHz, $CDCl_3$) δ 170.0 (d, *J* = 51.3 Hz), 170.0, 169.7, 143.6, 137.8, 128.6, 128.5, 127.8, 126.5, 123.8, 119.7, 83.3, 45.0 (d, *J* = 50.6 Hz), 45.0, 24.8, 24.6, 13.8.

^{11}B NMR (96 MHz, $CDCl_3$) δ 33.4.

(Note: Through the NMR spectra, we can clearly see that the carbonyl and methylene carbon atoms are labelled by ^{13}C).

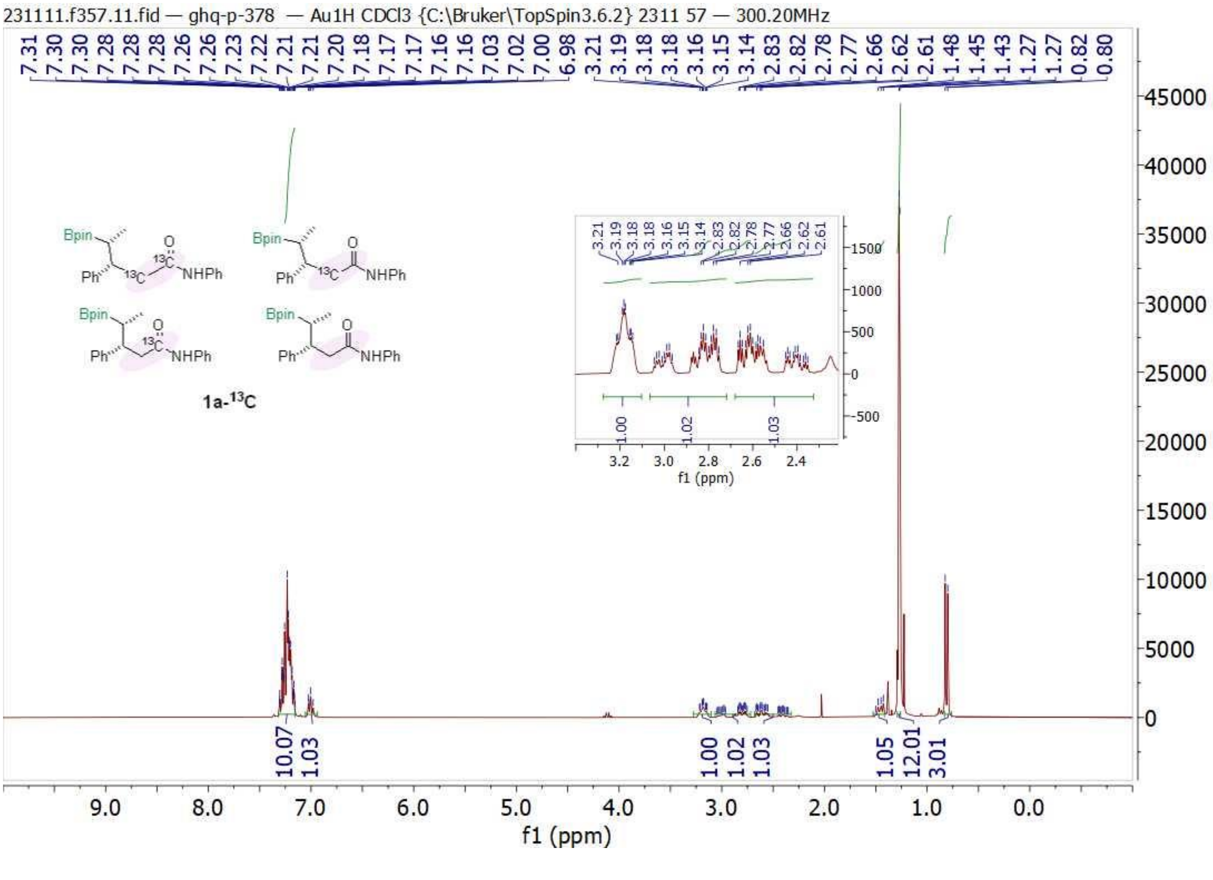

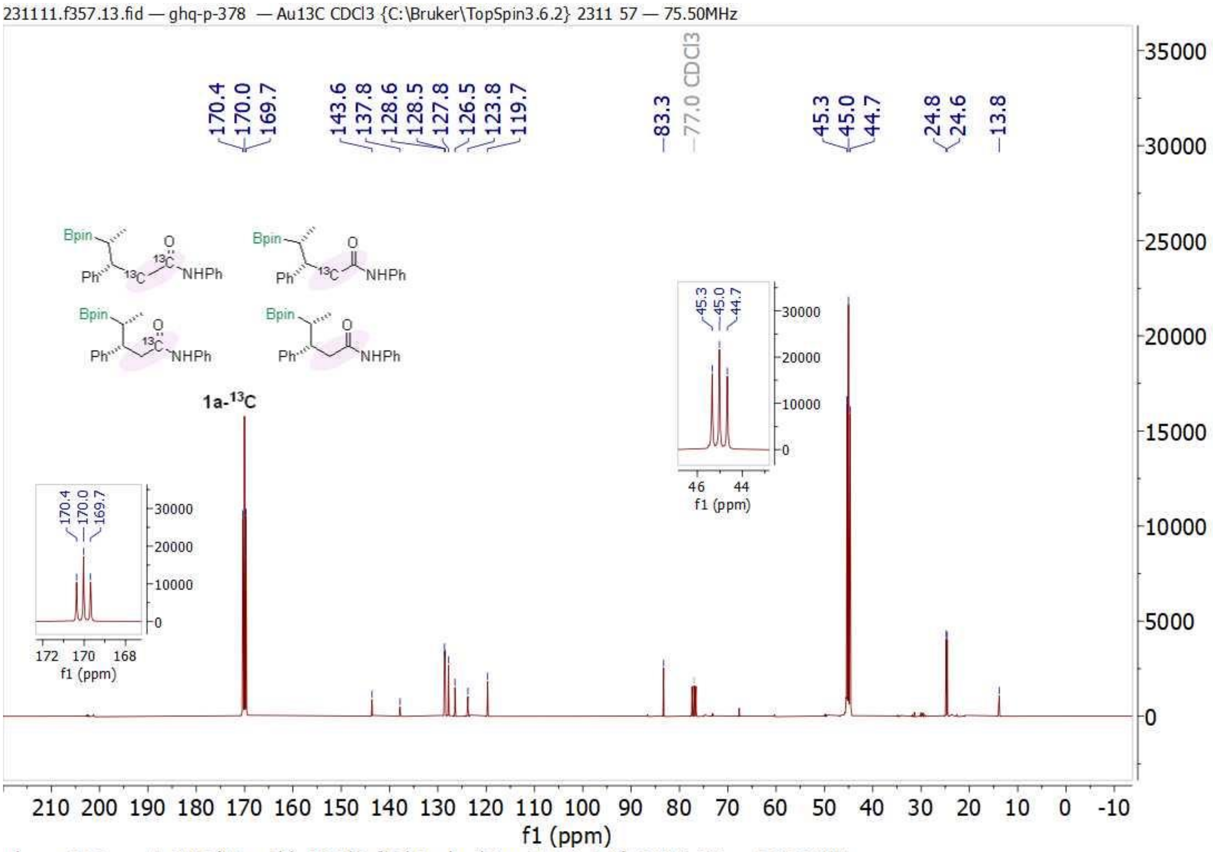
231111.f357.13.fid — ghq-p-378 — Au13C CDCl3 {C:\Bruker\TopSpin3.6.2} 2311 57 — 75.50MHz
170.4
170.0
169.7
143.6
137.8
128.6
128.5
127.8
126.5
123.8
119.7
83.3
77.0 CDCl3
45.3
45.0
44.7
24.8
24.6
13.8
Bpin
NHPh
Ph
1a-13C
f1 (ppm)

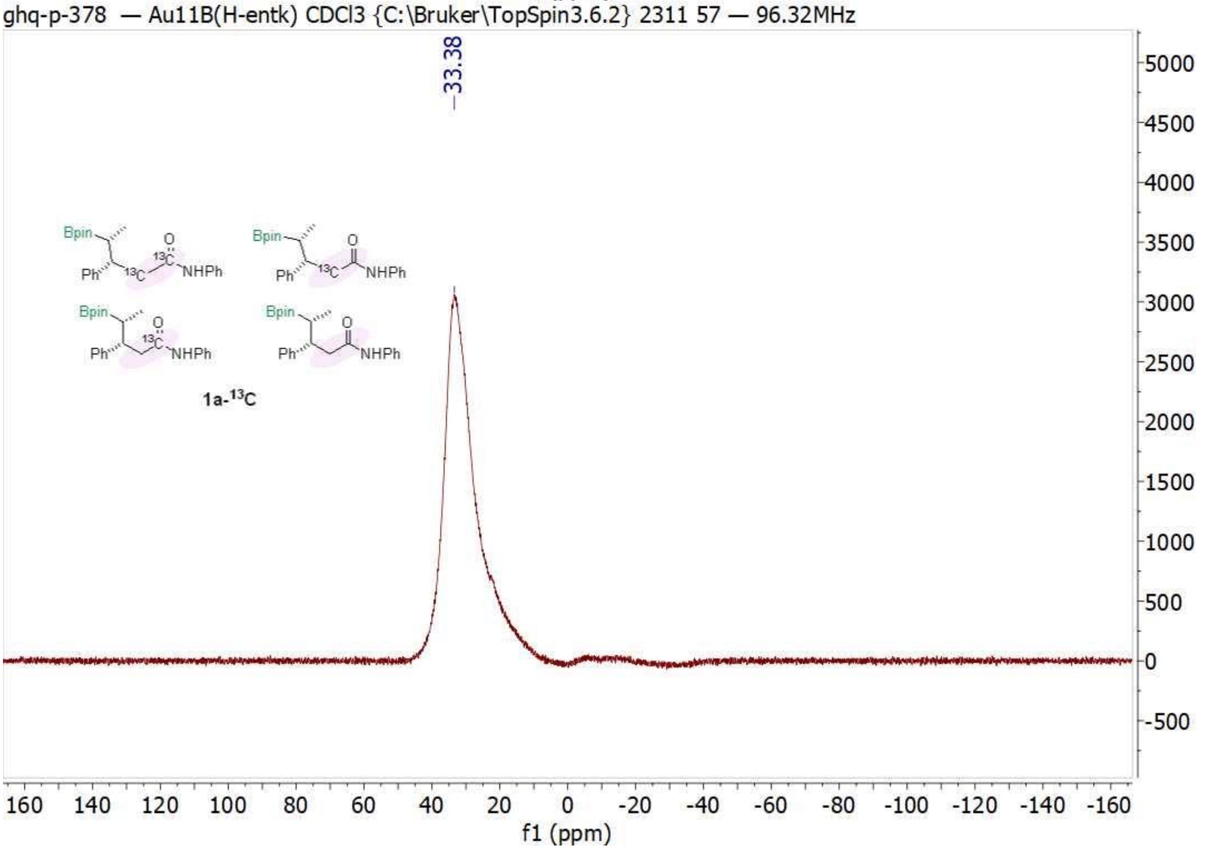
ghq-p-378 — Au11B(H-entk) CDCl3 {C:\Bruker\TopSpin3.6.2} 2311 57 — 96.32MHz
33.38
Bpin
NHPh
Ph
1a-13C
f1 (ppm)

6.2 Deuterium labeling experiment.

6.2.1 Deuterated *trans-β*-methylstyrene.

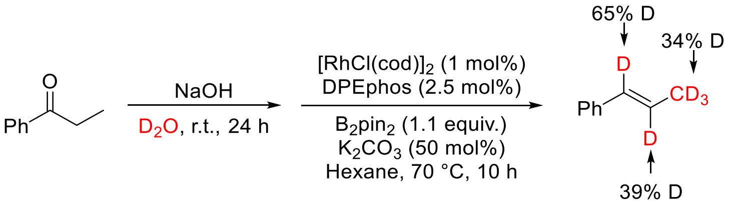

1-*d*$_5$

(*E*)-(prop-1-en-1-yl-*d*$_5$)benzene (**1-*d*$_5$**)was synthesized according to the existed literature.[5]

^{1}H NMR (400 MHz, $CDCl_3$) δ 7.46 – 7.33 (m, 4H), 7.29 – 7.24 (m, 1H), 6.47 (dd, *J* = 4.5, 1.8 Hz, 0.35H), 6.31 (dddd, *J* = 10.1, 5.6, 2.5, 1.1 Hz, 0.61H), 1.96 – 1.93 (m, 1.97H).

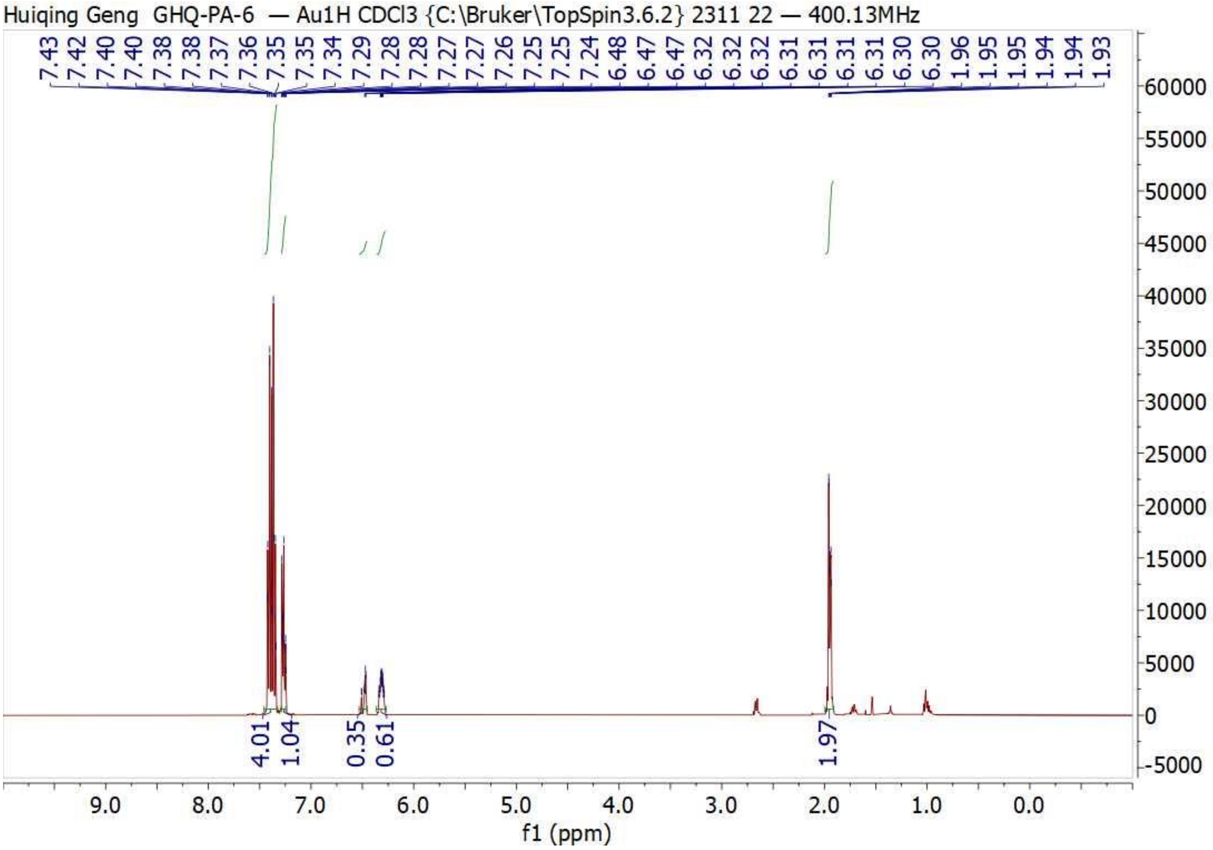

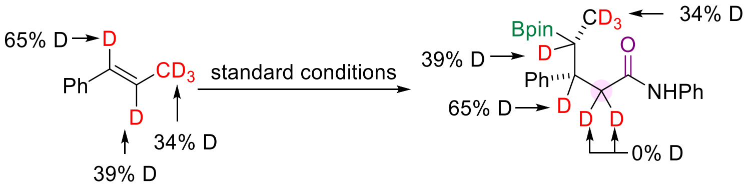

1-*d*$_5$ **1a-D1**, 75% (58%)

A dried vial (4 mL) was charged with $Cu(OAc)_2$ (5 mol%), DPPE (5 mol%), B_2pin_2 (3.5 equiv.), and a stirring bar. When the NaOEt (2.75 equiv.) is added, the vial was sealed by PTFE/white rubber septum (Wheaton 13 mm Septa) and phenolic cap immediately. The vial was evacuated under vacuum and recharged with argon for three times with a needle. Then, (*E*)-(prop-1-en-1-yl-*d*$_5$)benzene (**1-*d*$_5$**) (0.2 mmol), aniline (2.5 equiv.) and DMSO (1.0 mL) were added under argon by using a syringe. The vial (or several vials) was placed in an alloy plate and transferred into a 300 mL autoclave of the 4560 series from Parr Instruments. The autoclave was charged with

CO (10 bar) after flushing two times with N_2 and two times with CO. The autoclave was then placed into an aluminum block on a magnetic stirrer. The reaction was performed for 20 h at 60 °C. After the reaction was complete, the autoclave was cooled down to room temperature and the pressure was released carefully. The reaction was quenched with water (2 mL) and extracted with EtOAc (2 mLx3). The organic layer was washed with water (10 mL) and dried over anhydrous Na_2SO_4 and concentrated under reduced pressure. After that the corresponding product (**1a-D1**) was afforded as white solid by directly purified by fast column chromatography. (For NMR yield: The organic layer was washed with water and 0.1 mmol of 1,3,5-trimethoxybenzene was added. After drying with anhydrous Na_2SO_4, an appropriate amount of organic layer was concentrated under reduced pressure to test the NMR yield.)

^{1}H NMR (300 MHz, $CDCl_3$) δ 7.33 – 7.22 (m, 9H), 7.04 (ddd, *J* = 8.5, 6.3, 2.4 Hz, 2H), 3.19 (ddt, *J* = 10.0, 6.8, 4.0 Hz, 1H), 2.82 (dd, *J* = 14.0, 3.2 Hz, 1H), 2.70 – 2.53 (m, 1H), 1.50 – 1.44 (m, 0.61H), 1.31 (d, *J* = 1.8 Hz, 13H), 0.82 (d, *J* = 6.3 Hz, 1.97H).

^{13}C NMR (75 MHz, $CDCl_3$) δ 170.0, 143.6, 137.8, 128.7, 128.6, 127.8, 126.6, 123.9, 119.7, 83.4, 45.6, 45.2, 24.9, 24.6, 13.9 – 13.4 (m).

^{11}B NMR (96 MHz, $CDCl_3$) δ 33.6.

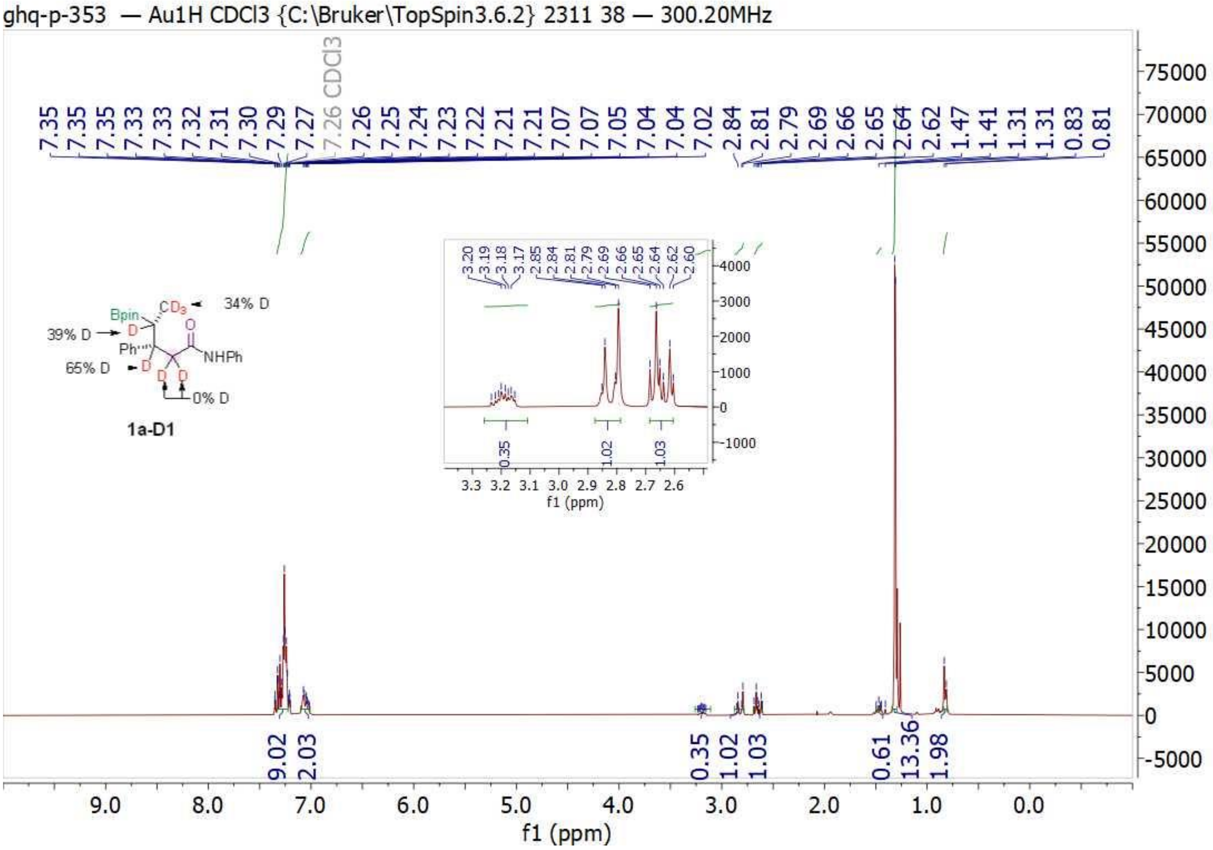

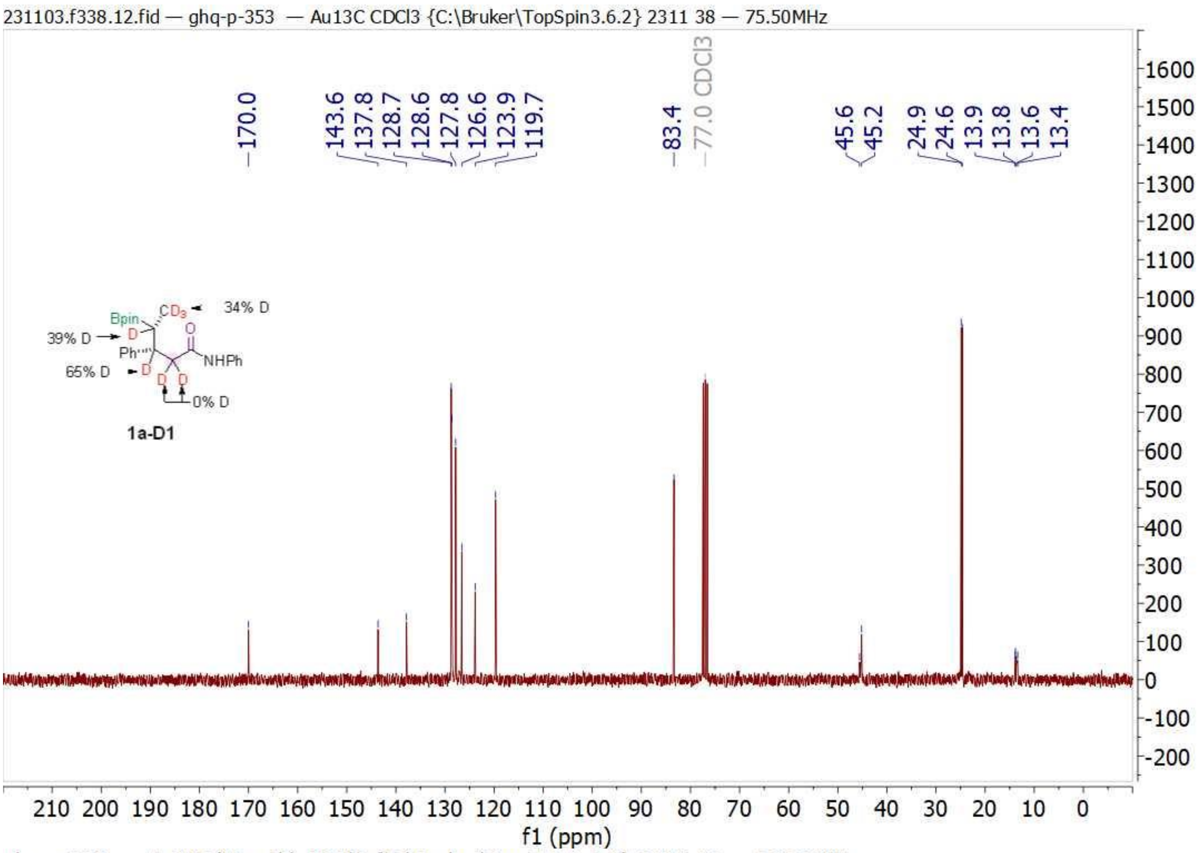
231103.f338.12.fid — ghq-p-353 — Au13C CDCl3 {C:\Bruker\TopSpin3.6.2} 2311 38 — 75.50MHz
170.0
143.6
137.8
128.7
128.6
127.8
126.6
123.9
119.7
83.4
77.0 CDCl3
45.6
45.2
24.9
24.6
13.9
13.8
13.6
13.4
34% D
39% D
65% D
0% D
1a-D1
f1 (ppm)

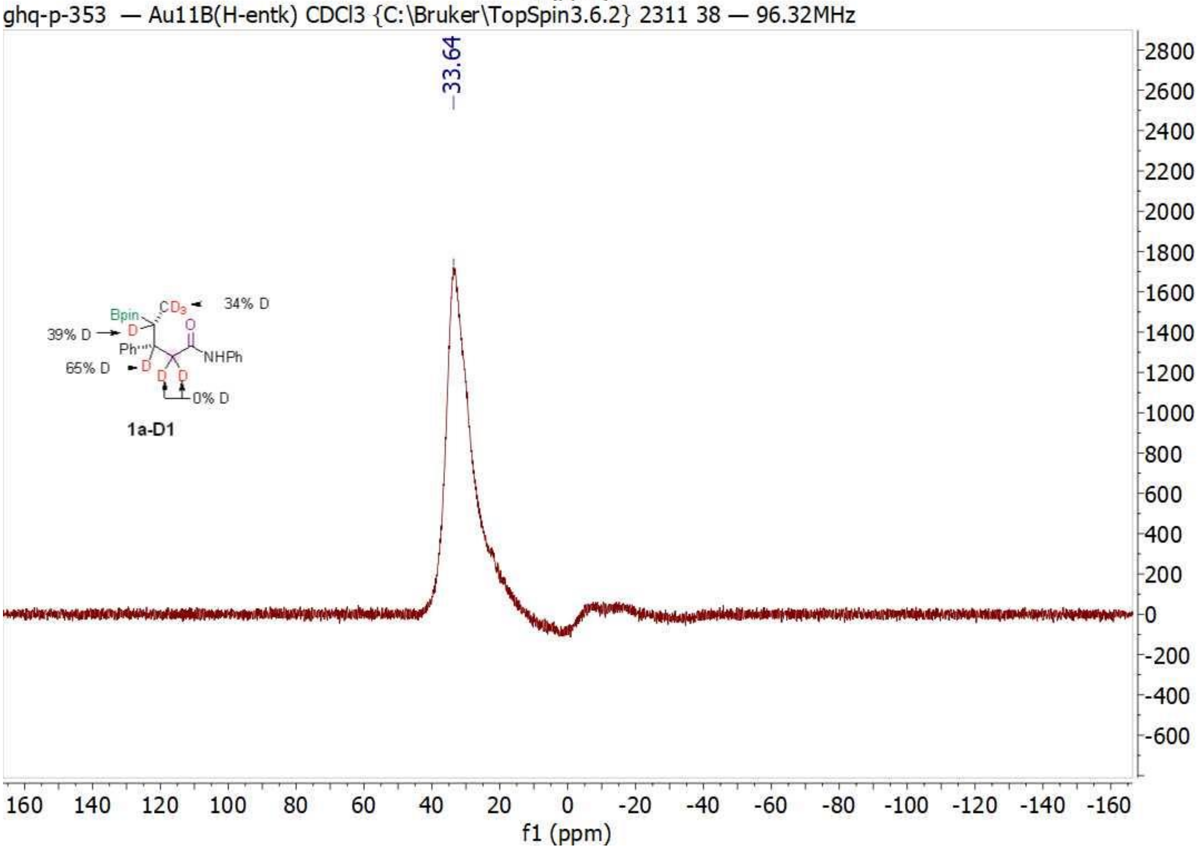
ghq-p-353 — Au11B(H-entk) CDCl3 {C:\Bruker\TopSpin3.6.2} 2311 38 — 96.32MHz
33.64
34% D
39% D
65% D
0% D
1a-D1
f1 (ppm)

6.2.2 DMSO-d_6.

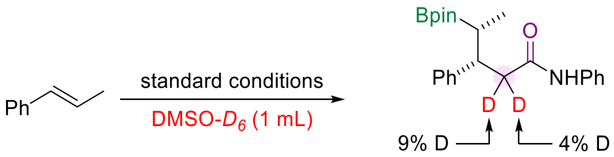

1a-D2, 69% (54%)

A dried vial (4 mL) was charged with $Cu(OAc)_2$ (5 mol%), DPPE (5 mol%), B_2pin_2 (3.5 equiv.), and a stirring bar. When the NaOEt (2.75 equiv.) is added, the vial was sealed by PTFE/white rubber septum (Wheaton 13 mm Septa) and phenolic cap immediately. The vial was evacuated under vacuum and recharged with argon for three times with a needle. Then, *trans*-*β*-methylstyrene (0.2 mmol), aniline (2.5 equiv.) and DMSO-d_6 (1.0 mL) were added under argon by using a syringe. The vial (or several vials) was placed in an alloy plate and transferred into a 300 mL autoclave of the 4560 series from Parr Instruments. The autoclave was charged with CO (10 bar) after flushing two times with N_2 and two times with CO. The autoclave was then placed into an aluminum block on a magnetic stirrer. The reaction was performed for 20 h at 60 °C. After the reaction was complete, the autoclave was cooled down to room temperature and the pressure was released carefully. The reaction was quenched with water (2 mL) and extracted with EtOAc (2 mLx3). The organic layer was washed with water (10 mL) and dried over anhydrous Na_2SO_4 and concentrated under reduced pressure. After that the corresponding product (**1a-D2**) was afforded as white solid by directly purified by fast column chromatography. (For NMR yield: The organic layer was washed with water and 0.1 mmol of 1,3,5-trimethoxybenzene was added. After drying with anhydrous Na_2SO_4, an appropriate amount of organic layer was concentrated under reduced pressure to test the NMR yield.)

^{1}H NMR (400 MHz, $CDCl_3$) δ 7.33 – 7.28 (m, 2H), 7.25 – 7.18 (m, 7H), 7.07 – 6.96 (m, 2H), 3.15 (td, J = 10.2, 4.1 Hz, 1H), 2.80 (dd, J = 14.0, 4.1 Hz, 1H), 2.62 (dd, J = 14.0, 10.3 Hz, 1H), 1.49 – 1.42 (m, 1H), 1.29 (d, J = 2.4 Hz, 12H), 0.81 (d, J = 7.4 Hz, 3H).

^{13}C NMR (101 MHz, $CDCl_3$) δ 170.0, 143.7, 137.8, 128.8, 128.6, 127.8, 126.6, 123.9, 119.7, 83.4, 45.7, 45.3, 24.9, 24.6, 14.0.

^{11}B NMR (96 MHz, $CDCl_3$) δ 33.3.

HRMS (ESI-TOF): calcd for $[M+Na]^+$ $C_{27}H_{30}{}^{10}BNO_3$ 401.2247, found: 401.2251.

Huiqing Geng GHQ-P-347 — Au1H CDCl3 {C:\Bruker\TopSpin3.6.2} 2311 16 — 400.13MHz

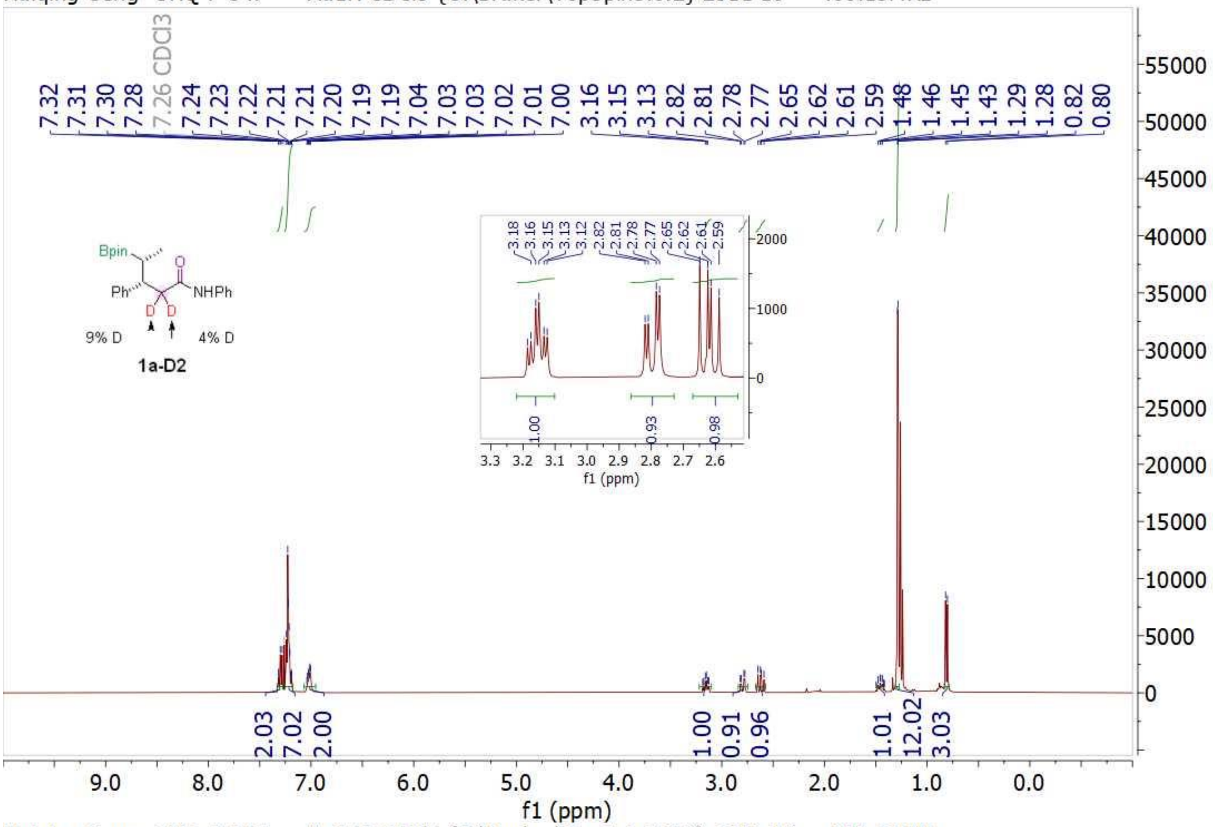

Huiqing Geng GHQ-P-347 — Au13C CDCl3 {C:\Bruker\TopSpin3.6.2} 2311 16 — 100.63MHz

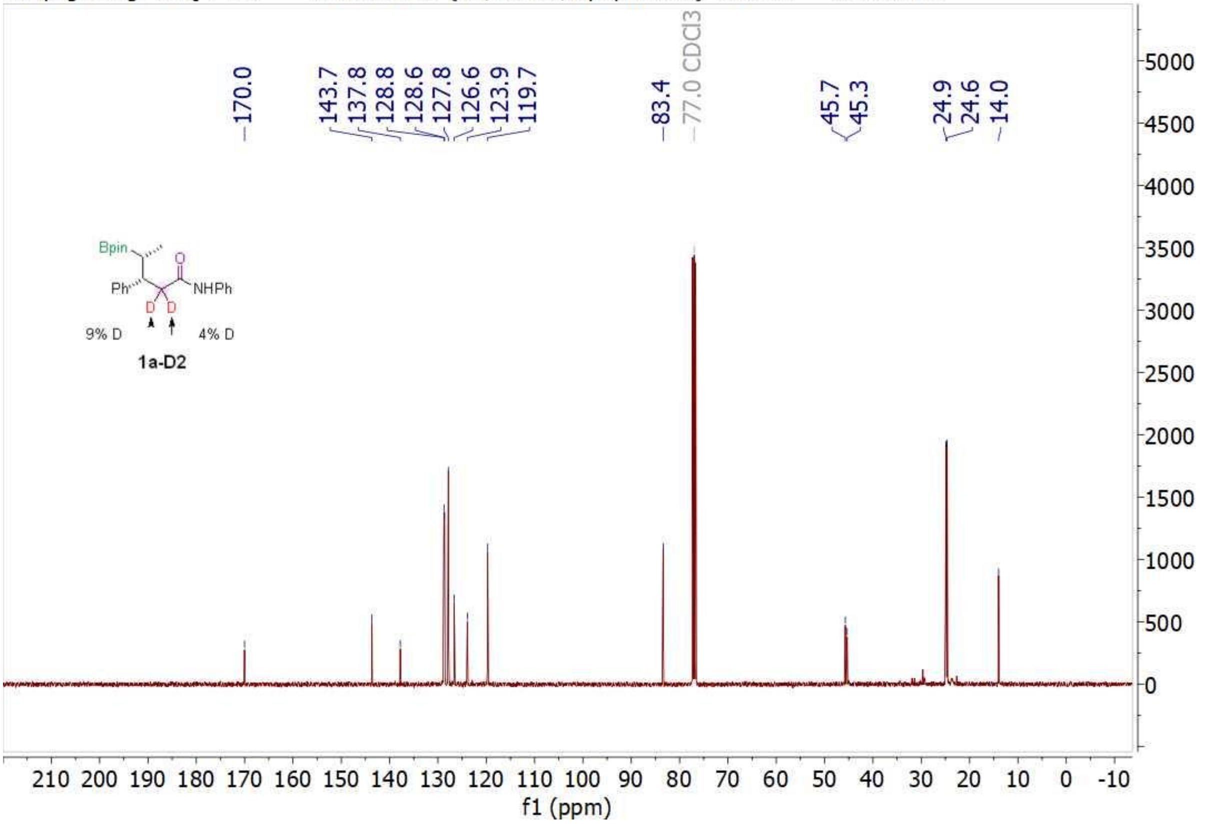

Huiqing Geng GHQ-P-347 — Au11B(H-entk) CDCl3 {C:\Bruker\TopSpin3.6.2} 2311 22 — 96.32MHz

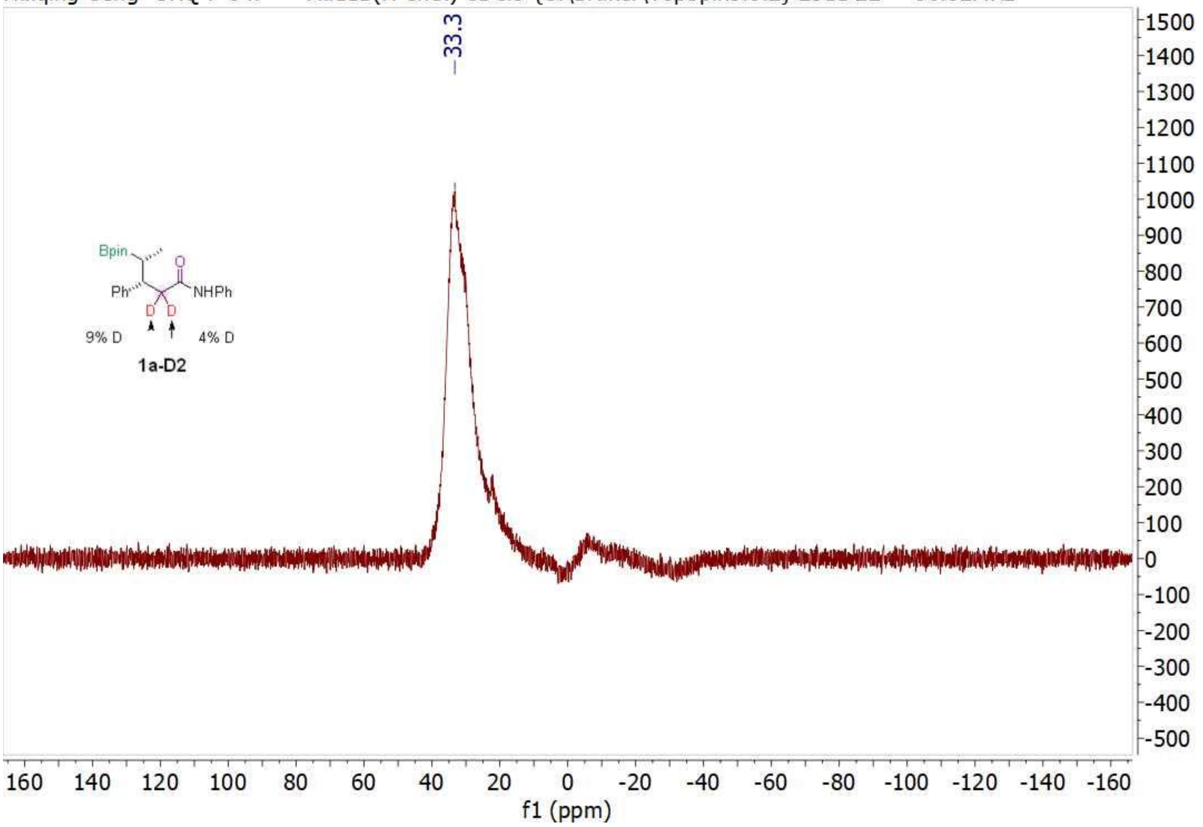

6.2.3 Aniline-d_7.

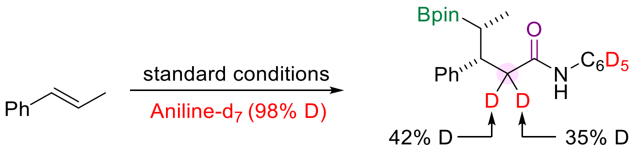

1a-D3, 72% (55%)

A dried vial (4 mL) was charged with $Cu(OAc)_2$ (5 mol%), DPPE (5 mol%), B_2pin_2 (3.5 equiv.), and a stirring bar. When the NaOEt (2.75 equiv.) is added, the vial was sealed by PTFE/white rubber septum (Wheaton 13 mm Septa) and phenolic cap immediately. The vial was evacuated under vacuum and recharged with argon for three times with a needle. Then, *trans*-*β*-methylstyrene (0.2 mmol), aniline-d_7 (2.5 equiv.) and DMSO (1.0 mL) were added under argon by using a syringe. The vial (or several vials) was placed in an alloy plate and transferred into a 300 mL autoclave of the 4560 series from Parr Instruments. The autoclave was charged with CO (10 bar) after flushing two times with N_2 and two times with CO. The autoclave was then placed into an aluminum block on a magnetic stirrer. The reaction was performed for 20 h at 60 °C. After the reaction was complete, the autoclave was cooled down to room temperature and the pressure was released carefully. The reaction was quenched with water (2 mL) and extracted with EtOAc (2 mLx3). The organic layer was washed with water (10 mL) and dried over anhydrous Na_2SO_4 and concentrated under reduced pressure. After that the corresponding product (**1a-D3**) was afforded as white solid by directly purified by fast column chromatography. (For NMR yield: The organic layer was washed with water and 0.1 mmol of 1,3,5-trimethoxybenzene was added. After drying with anhydrous Na_2SO_4, an appropriate amount of organic layer was concentrated under reduced pressure to test the NMR yield.)

^{1}H NMR (300 MHz, $CDCl_3$) δ 7.33 – 7.17 (m, 5H), 7.08 (s, 1H), 3.33 – 3.05 (m, 1H), 2.80 (dd, J = 14.0, 4.1 Hz, 1H), 2.67 – 2.58 (m, 1H), 1.46 (dd, J = 10.0, 7.5 Hz, 1H), 1.28 (d, J = 1.8 Hz, 12H), 0.82 (d, J = 7.4 Hz, 3H).

^{13}C NMR (75 MHz, $CDCl_3$) δ 170.0, 143.7, 128.6, 127.8, 126.6, 83.3, 45.6, 45.2, 24.9, 24.6, 13.9.

^{11}B NMR (96 MHz, $CDCl_3$) δ 33.3.

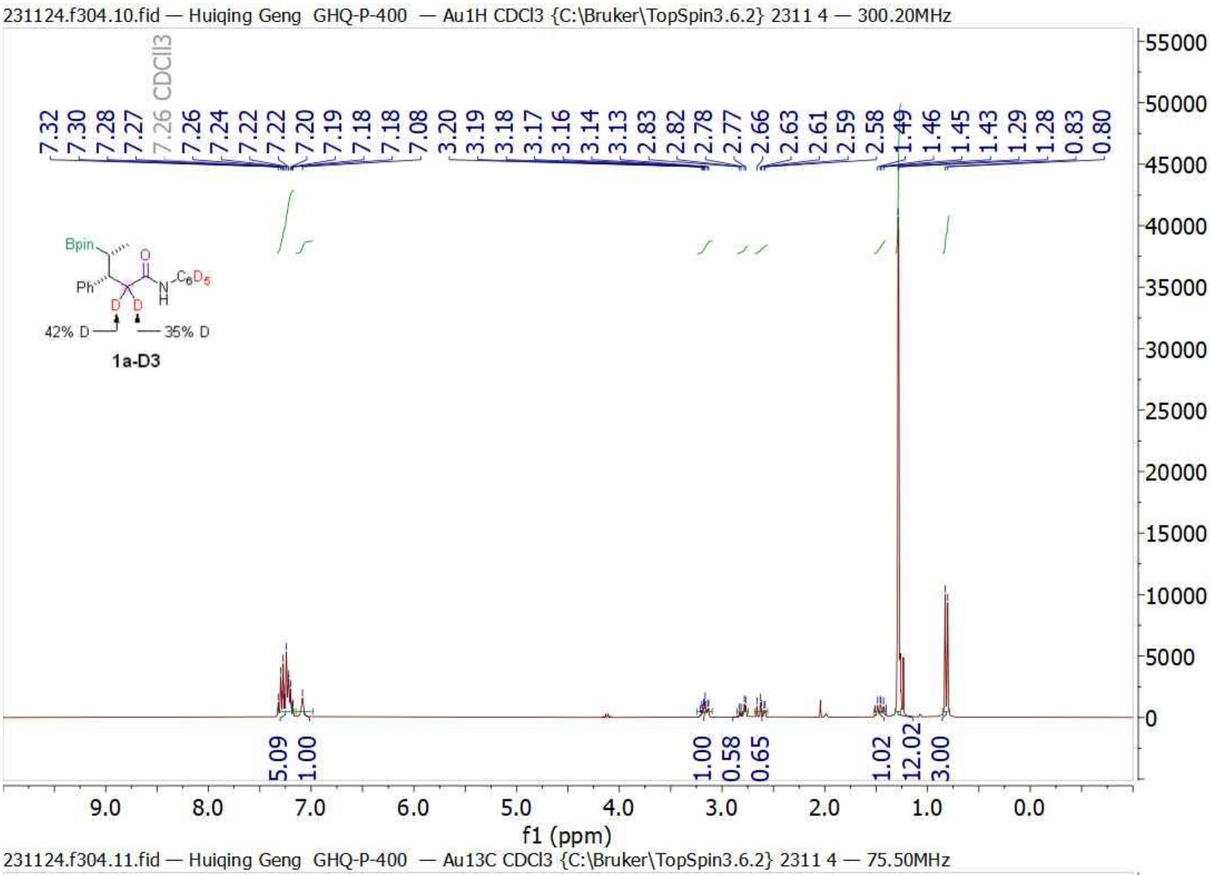
231124.f304.10.fid — Huiqing Geng GHQ-P-400 — Au1H CDCl3 {C:\Bruker\TopSpin3.6.2} 2311 4 — 300.20MHz
7.32 7.30 7.28 7.27 7.26 CDCl3 7.26 7.24 7.22 7.22 7.20 7.19 7.18 7.18 7.08 3.20 3.19 3.18 3.17 3.16 3.14 3.13 2.83 2.82 2.78 2.77 2.66 2.63 2.61 2.59 2.58 1.49 1.46 1.45 1.43 1.29 1.28 0.83 0.80
Bpin
Ph
D D
N
H
C_6D_5
42% D
35% D
1a-D3
5.09 1.00 1.00 0.58 0.65 1.02 12.02 3.00
f1 (ppm)

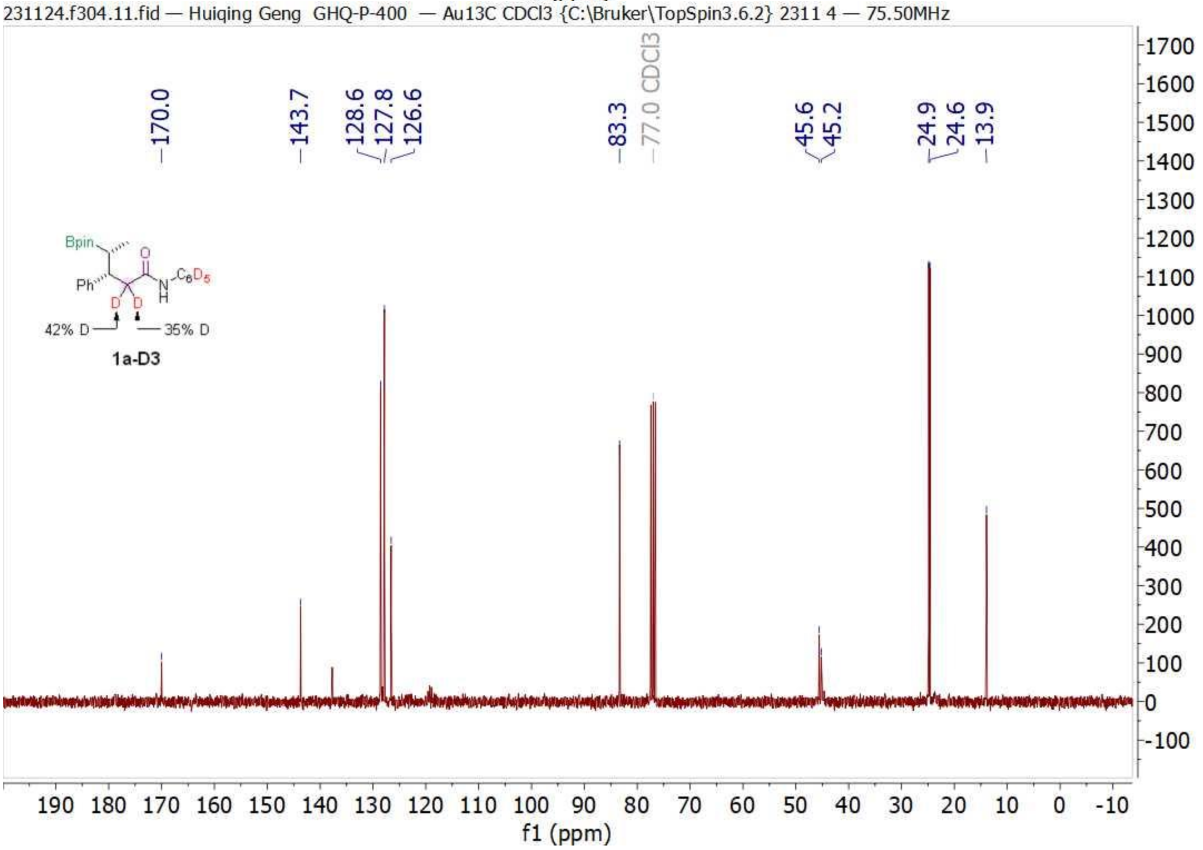
231124.f304.11.fid — Huiqing Geng GHQ-P-400 — Au13C CDCl3 {C:\Bruker\TopSpin3.6.2} 2311 4 — 75.50MHz
170.0 143.7 128.6 127.8 126.6 83.3 77.0 CDCl3 45.6 45.2 24.9 24.6 13.9
Bpin
Ph
D D
N
H
C_6D_5
42% D
35% D
1a-D3
f1 (ppm)

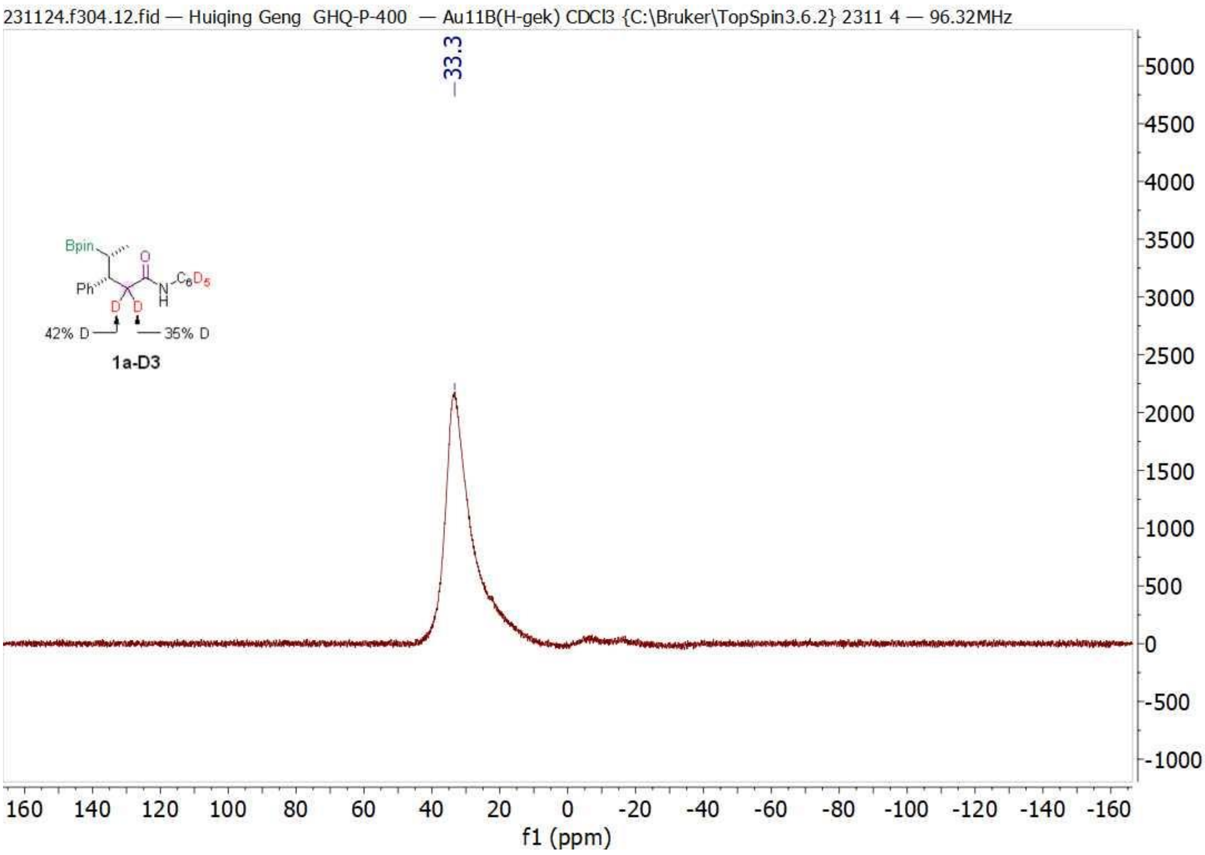
231124.f304.12.fid — Huiqing Geng GHQ-P-400 — Au11B(H-gek) CDCl3 {C:\Bruker\TopSpin3.6.2} 2311 4 — 96.32MHz
33.3
Bpin
Ph
D
D
N
H
C6D5
O
42% D
35% D
1a-D3
f1 (ppm)

6.2.4 Quenching with D_2O.

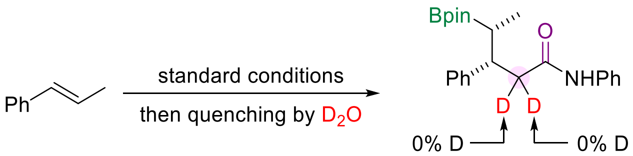

1a-D4, 71% (53%)

A dried vial (4 mL) was charged with $Cu(OAc)_2$ (5 mol%), DPPE (5 mol%), B_2pin_2 (3.5 equiv.), and a stirring bar. When the NaOEt (2.75 equiv.) is added, the vial was sealed by PTFE/white rubber septum (Wheaton 13 mm Septa) and phenolic cap immediately. The vial was evacuated under vacuum and recharged with argon for three times with a needle. Then, *trans-β*-methylstyrene (0.2 mmol), aniline (2.5 equiv.) and DMSO (1.0 mL) were added under argon by using a syringe. The vial (or several vials) was placed in an alloy plate and transferred into a 300 mL autoclave of the 4560 series from Parr Instruments. The autoclave was charged with CO (10 bar) after flushing two times with N_2 and two times with CO. The autoclave was then placed into an aluminum block on a magnetic stirrer. The reaction was performed for 20 h at 60 °C. After the reaction was complete, the autoclave was cooled down to room temperature and the pressure was released carefully. The reaction was quenched with D_2O (2 mL) and stirring for 5 minutes, then extracted with EtOAc (2 mLx3). The organic layer was washed with water (10 mL) and dried over anhydrous Na_2SO_4 and concentrated under reduced pressure. After that the corresponding product (**1a-D4**) was afforded as white solid by directly purified by fast column chromatography. (For NMR yield: The organic layer was washed with water and 0.1 mmol of 1,3,5-trimethoxybenzene was added. After drying with anhydrous Na_2SO_4, an appropriate amount of organic layer was concentrated under reduced pressure to test the NMR yield.)

^{1}H NMR (300 MHz, $CDCl_3$) δ 7.24 – 7.09 (m, 9H), 7.01 (dd, *J* = 8.4, 2.5 Hz, 1H), 6.97 – 6.88 (m, 1H), 3.09 (td, *J* = 10.1, 4.1 Hz, 1H), 2.72 (dd, *J* = 14.0, 4.2 Hz, 1H), 2.54 (dd, *J* = 14.0, 10.2 Hz, 1H), 1.37 (dd, *J* = 9.8, 7.3 Hz, 1H), 1.20 (d, *J* = 1.9 Hz, 12H), 0.73 (d, *J* = 7.4 Hz, 3H).

^{13}C NMR (75 MHz, $CDCl_3$) δ 170.0, 143.6, 137.8, 128.7, 128.6, 127.8, 126.6, 123.8, 119.7, 83.3, 45.6, 45.2, 24.9, 24.6, 13.9.

^{11}B NMR (96 MHz, $CDCl_3$) δ 33.1.

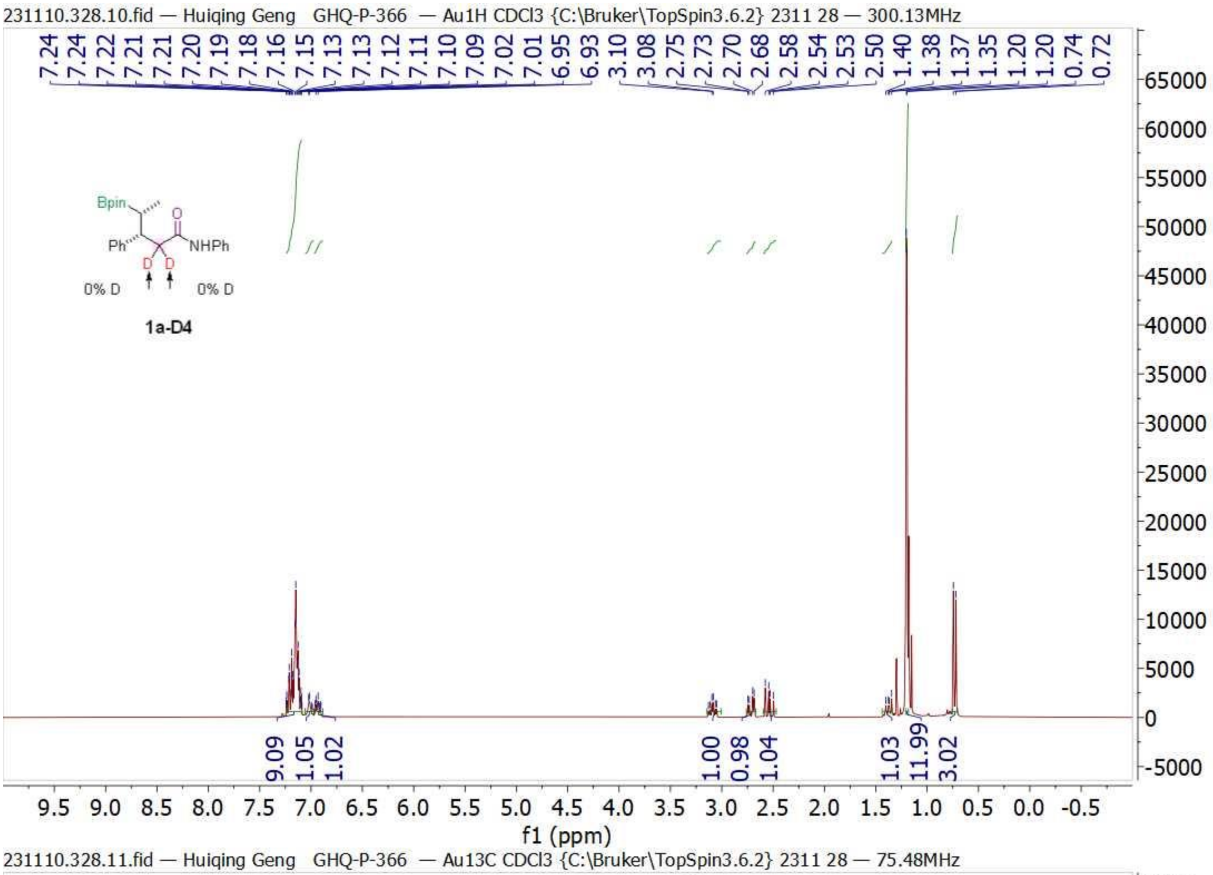
231110.328.10.fid — Huiqing Geng GHQ-P-366 — Au1H CDCl3 {C:\Bruker\TopSpin3.6.2} 2311 28 — 300.13MHz
7.24 7.24 7.22 7.21 7.21 7.20 7.19 7.18 7.16 7.15 7.13 7.13 7.12 7.11 7.10 7.09 7.02 7.01 6.95 6.93 3.10 3.08 2.75 2.73 2.70 2.68 2.58 2.54 2.53 2.50 1.40 1.38 1.37 1.35 1.20 1.20 0.74 0.72
Bpin
O
Ph
NHPh
D D
0% D
0% D
1a-D4
9.09 1.05 1.02 1.00 0.98 1.04 1.03 11.99 3.02
f1 (ppm)

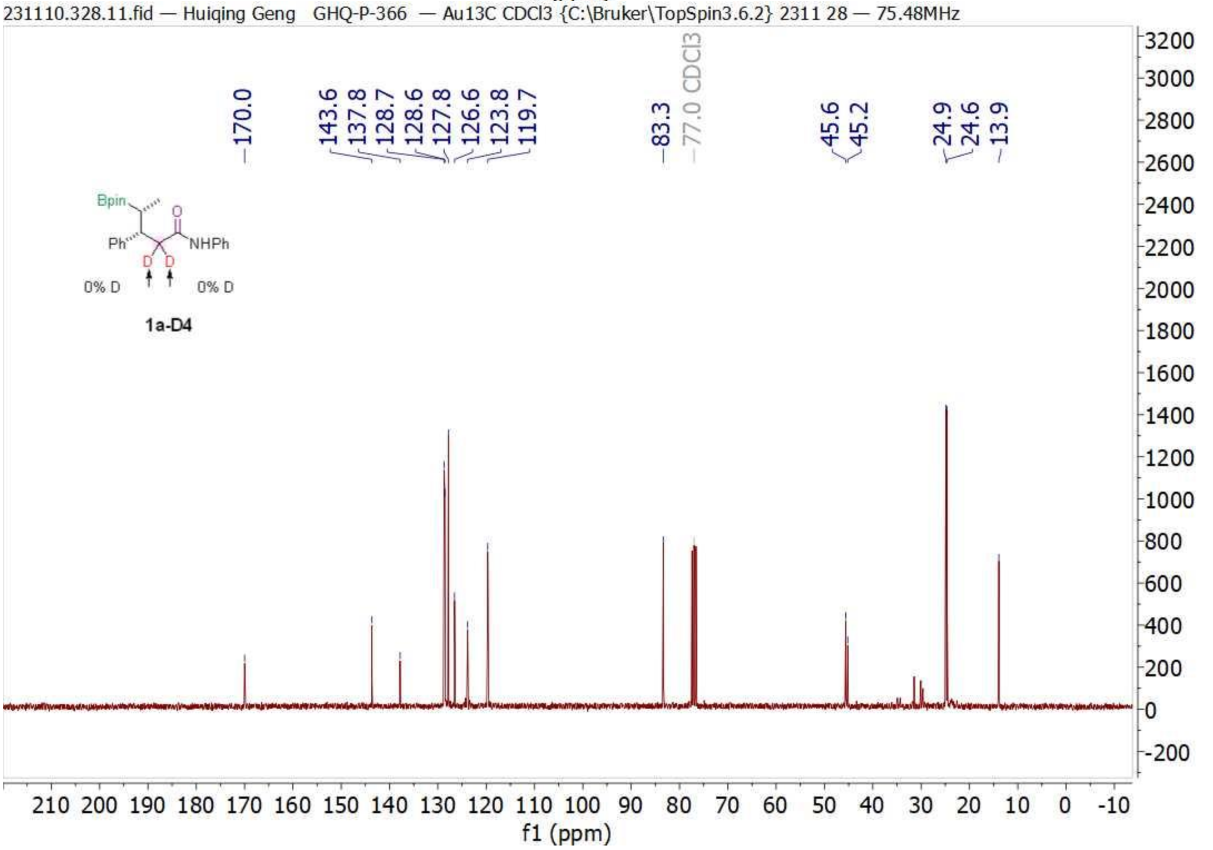
231110.328.11.fid — Huiqing Geng GHQ-P-366 — Au13C CDCl3 {C:\Bruker\TopSpin3.6.2} 2311 28 — 75.48MHz
170.0 143.6 137.8 128.7 128.6 127.8 126.6 123.8 119.7 83.3 77.0 CDCl3 45.6 45.2 24.9 24.6 13.9
Bpin
O
Ph
NHPh
D D
0% D
0% D
1a-D4
f1 (ppm)

231110.328.12.fid — Huiqing Geng GHQ-P-366 — Au11B CDCl3 {C:\Bruker\TopSpin3.6.2} 2311 28 — 96.29MHz

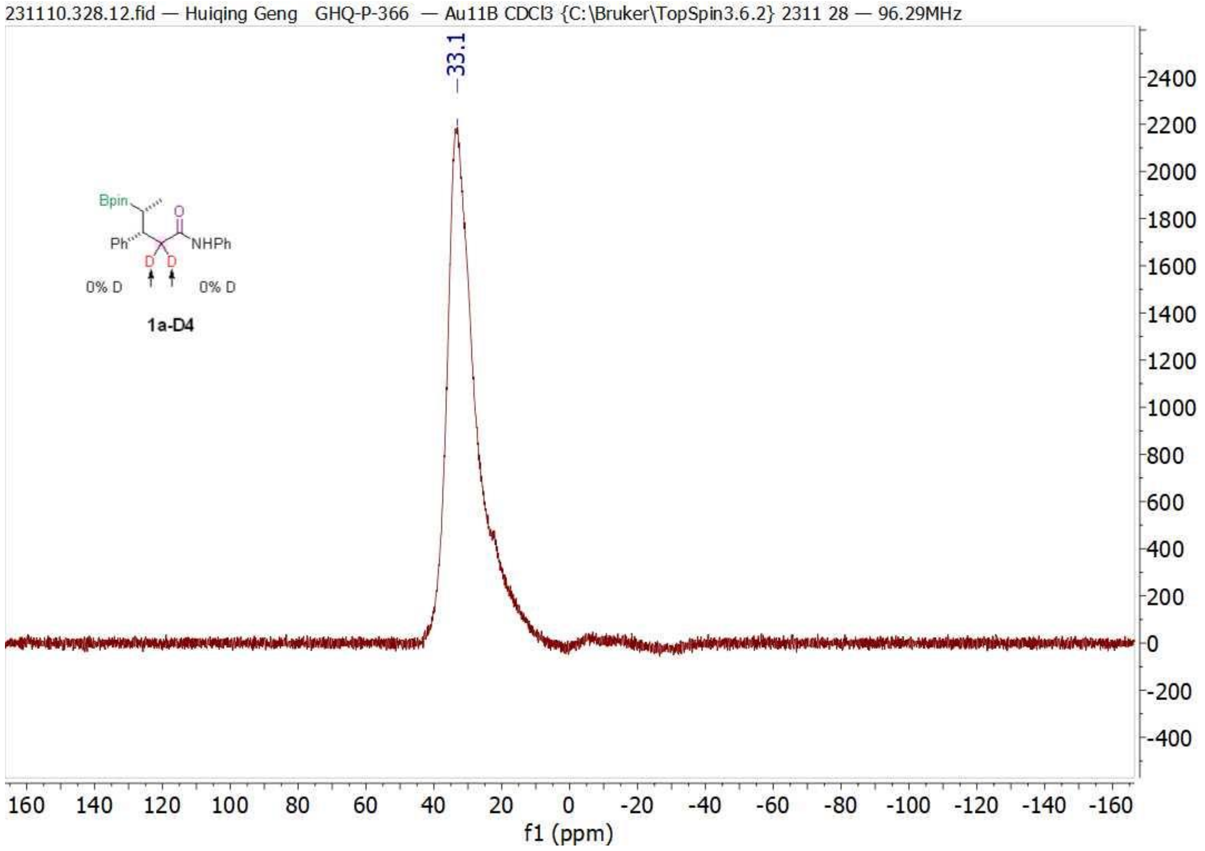

6.2.5 Additional D_2O.

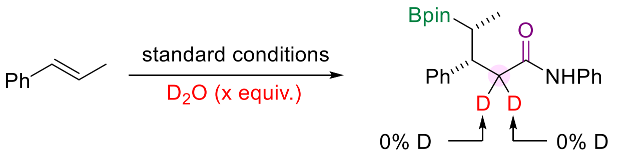

1a-D5 (x = 0.5), 55% (40%)
1a-D6 (x = 1.0), 41% (23%)

A dried vial (4 mL) was charged with $Cu(OAc)_2$ (5 mol%), DPPE (5 mol%), B_2pin_2 (3.5 equiv.), and a stirring bar. When the NaOEt (2.75 equiv.) is added, the vial was sealed by PTFE/white rubber septum (Wheaton 13 mm Septa) and phenolic cap immediately. The vial was evacuated under vacuum and recharged with argon for three times with a needle. Then, *trans-β*-methylstyrene (0.2 mmol), aniline (2.5 equiv.), D_2O (0.5 equiv.) and DMSO (1.0 mL) were added under argon by using a syringe. The vial (or several vials) was placed in an alloy plate and transferred into a 300 mL autoclave of the 4560 series from Parr Instruments. The autoclave was charged with CO (10 bar) after flushing two times with N_2 and two times with CO. The autoclave was then placed into an aluminum block on a magnetic stirrer. The reaction was performed for 20 h at 60 °C. After the reaction was complete, the autoclave was cooled down to room temperature and the pressure was released carefully. The reaction was quenched with water (2 mL) and extracted with EtOAc (2 mLx3). The organic layer was washed with water (10 mL) and dried over anhydrous Na_2SO_4 and concentrated under reduced pressure. After that the corresponding product (**1a-D5**) was afforded as white solid by directly purified by fast column chromatography. (For NMR yield: The organic layer was washed with water and 0.1 mmol of 1,3,5-trimethoxybenzene was added. After drying with anhydrous Na_2SO_4, an appropriate amount of organic layer was concentrated under reduced pressure to test the NMR yield.). The same procedure, but with 1.0 eq. of water, gives the product **1a-D6**.

1a-D5

^{1}H NMR (400 MHz, $CDCl_3$) δ 7.33 – 7.28 (m, 2H), 7.25 – 7.14 (m, 7H), 7.06 – 6.90 (m, 2H), 3.16 (td, *J* = 10.2, 4.0 Hz, 1H), 2.80 (dd, *J* = 14.0, 4.1 Hz, 1H), 2.62 (dd, *J* = 14.0, 10.3 Hz, 1H), 1.49 – 1.42 (m, 1H), 1.29 (d, *J* = 2.4 Hz, 12H), 0.81 (d, *J* = 7.4 Hz, 3H).

^{13}C NMR (101 MHz, $CDCl_3$) δ 170.0, 143.7, 137.8, 128.8, 128.6, 127.9, 126.6, 123.9, 119.7, 83.4, 45.7, 45.3, 24.9, 24.7, 14.0.

1a-D6

^{1}H NMR (400 MHz, $CDCl_3$) δ 7.33 – 7.29 (m, 2H), 7.23 (t, *J* = 5.2 Hz, 7H), 7.02 (dt, *J* = 8.6, 4.2 Hz, 1H), 6.94 (s, 1H), 3.15 (td, *J* = 10.2, 4.0 Hz, 1H), 2.80 (dd, *J* = 14.1, 4.0 Hz, 1H), 2.62 (dd, *J* = 14.0, 10.3 Hz, 1H), 1.46 (dd, *J* = 10.1, 7.6 Hz, 1H), 1.29 (d, *J* = 2.3 Hz, 12H), 0.81 (d, *J* = 7.4 Hz, 3H).

^{13}C NMR (101 MHz, $CDCl_3$) δ 170.0, 143.7, 137.9, 128.8, 128.7, 127.9, 126.7, 124.0, 119.7, 83.5, 45.8, 45.4, 24.9, 24.7, 14.1.

^{11}B NMR (128 MHz, $CDCl_3$) δ 36.3.

Huiqing Geng GHQ-P-370-1 — Au1H CDCl3 {C:\Bruker\TopSpin3.6.2} 2311 1 — 400.13MHz

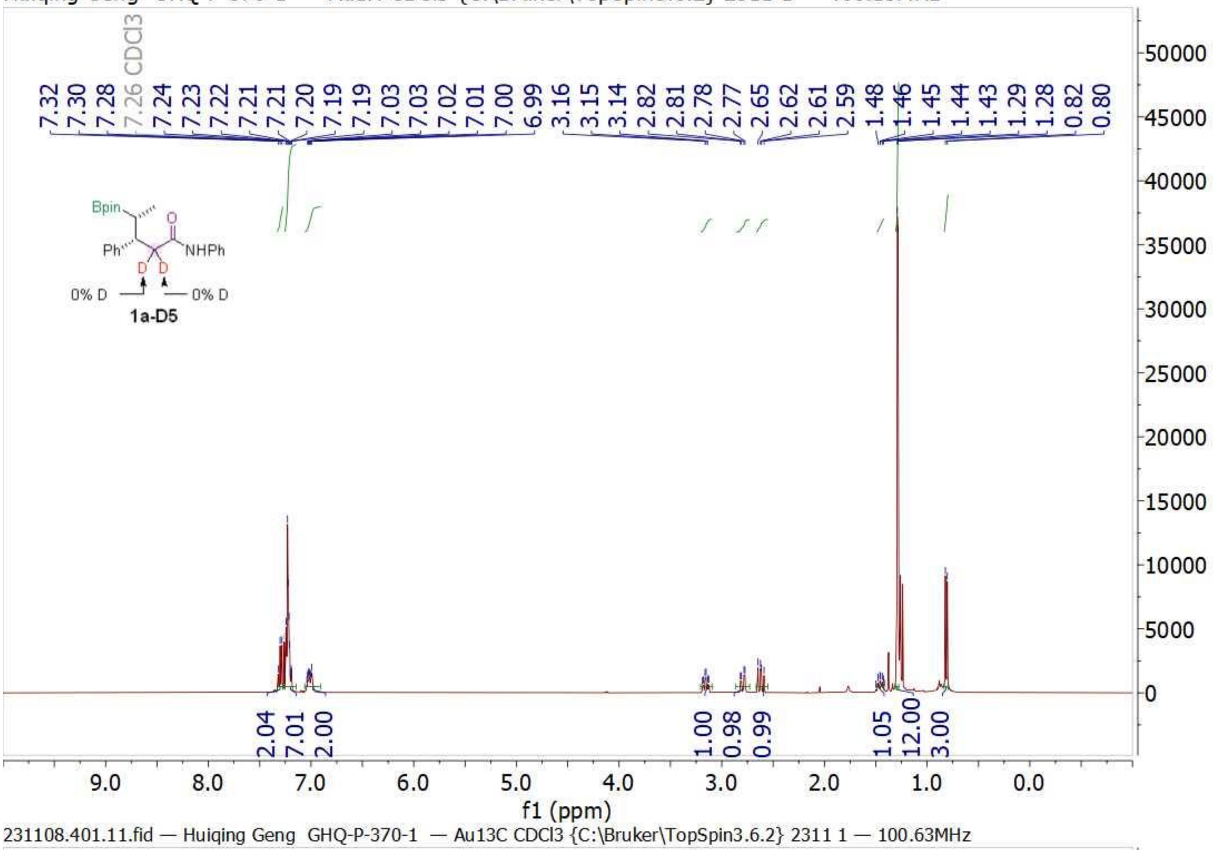

231108.401.11.fid — Huiqing Geng GHQ-P-370-1 — Au13C CDCl3 {C:\Bruker\TopSpin3.6.2} 2311 1 — 100.63MHz

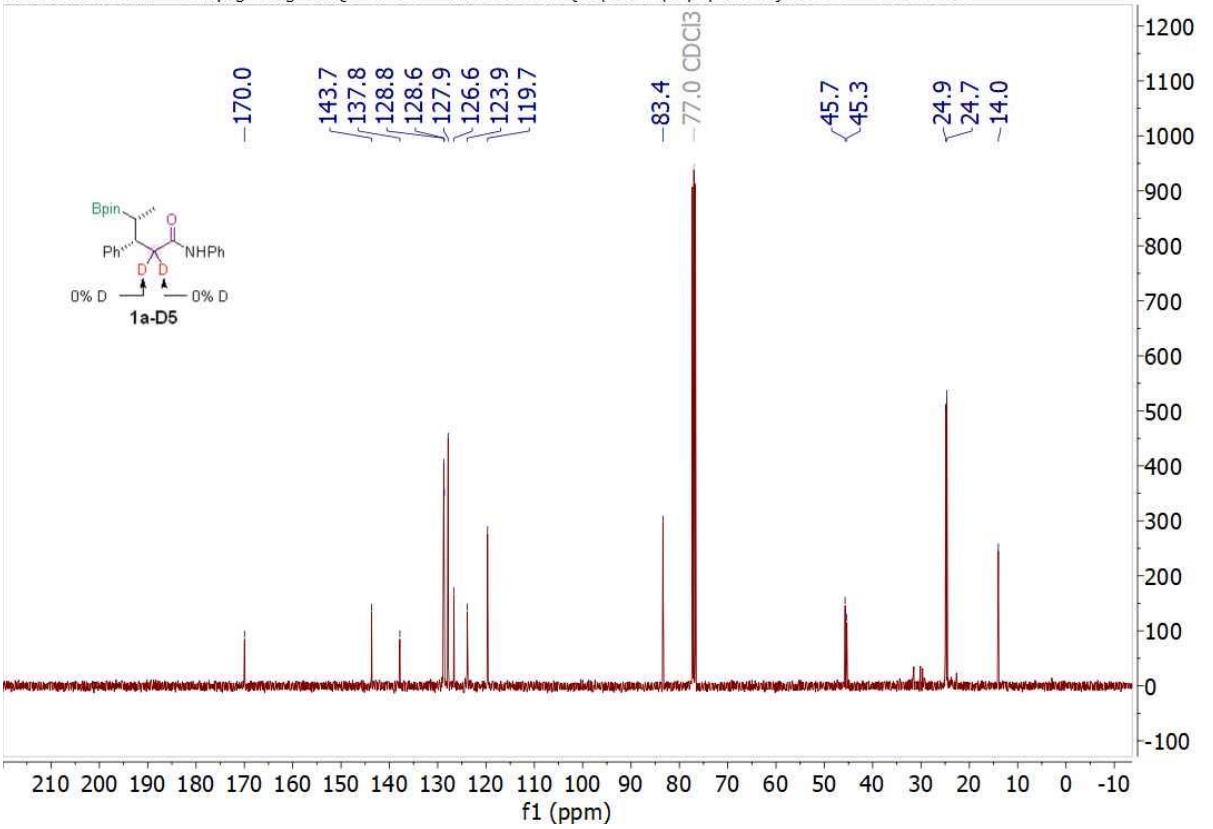

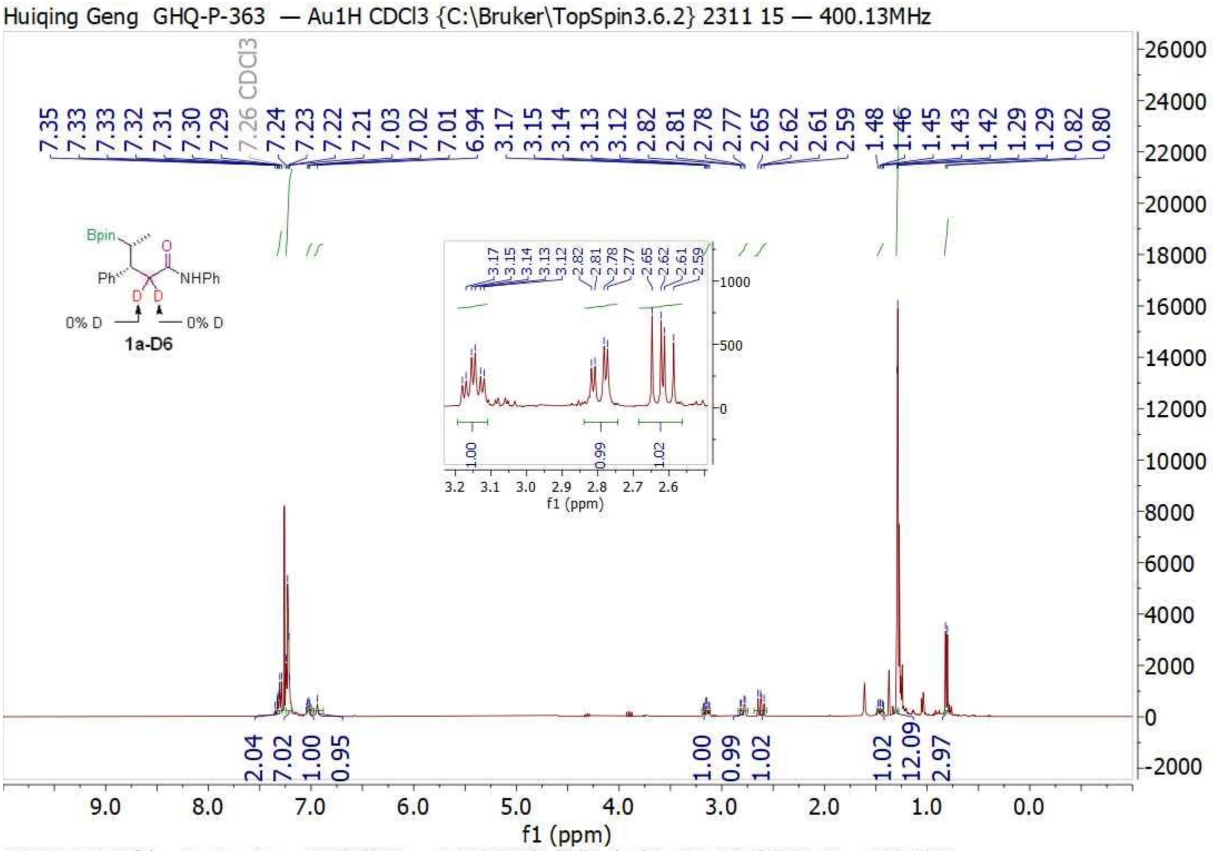
Huiqing Geng GHQ-P-363 — Au1H CDCl3 {C:\Bruker\TopSpin3.6.2} 2311 15 — 400.13MHz
7.35 7.33 7.33 7.32 7.31 7.30 7.29 7.26 CDCl3 7.24 7.23 7.22 7.21 7.03 7.02 7.01 6.94 3.17 3.15 3.14 3.13 3.12 2.82 2.81 2.78 2.77 2.65 2.62 2.61 2.59 1.48 1.46 1.45 1.43 1.42 1.29 1.29 0.82 0.80
Bpin
Ph
NHPh
0% D
0% D
1a-D6
2.04 7.02 1.00 0.95 1.00 0.99 1.02 1.02 12.09 2.97
f1 (ppm)

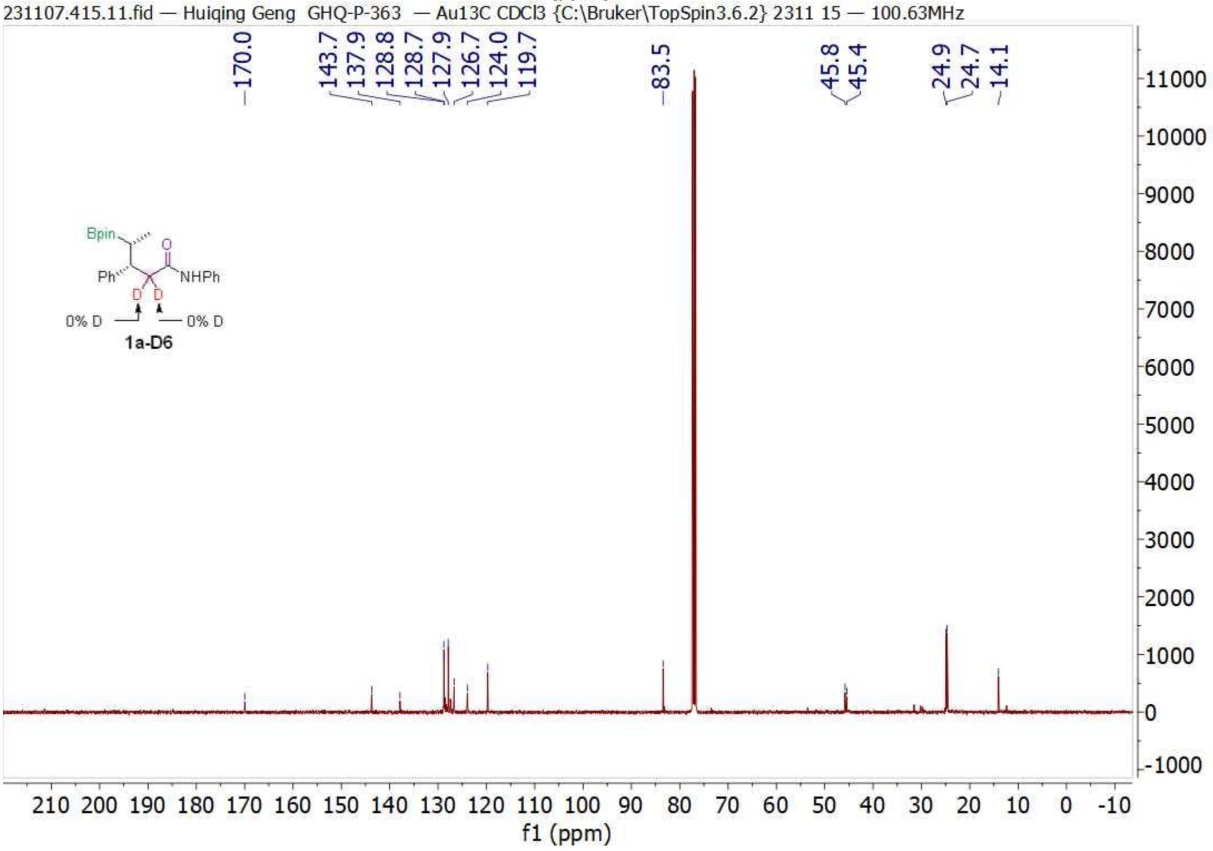
231107.415.11.fid — Huiqing Geng GHQ-P-363 — Au13C CDCl3 {C:\Bruker\TopSpin3.6.2} 2311 15 — 100.63MHz
170.0 143.7 137.9 128.8 128.7 127.9 126.7 124.0 119.7 83.5 45.8 45.4 24.9 24.7 14.1
Bpin
Ph
NHPh
0% D
0% D
1a-D6
f1 (ppm)

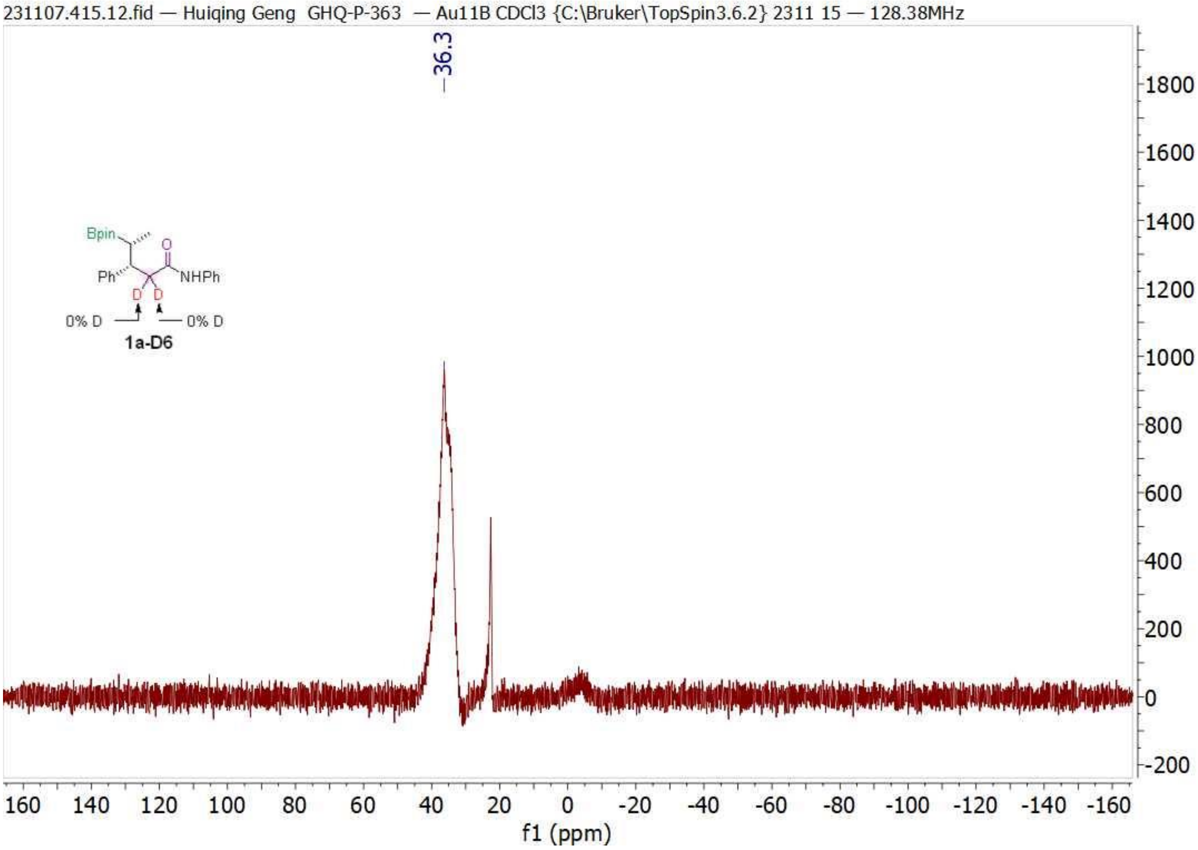

231107.415.12.fid — Huiqing Geng GHQ-P-363 — Au11B CDCl3 {C:\Bruker\TopSpin3.6.2} 2311 15 — 128.38MHz
36.3
Bpin
O
Ph
NHPh
D
D
0% D
0% D
1a-D6
f1 (ppm)

6.2.6 Mixed deuterated reagents of aniline-d_7 and DMSO-d_6.

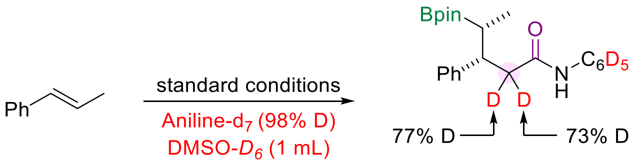

1a-D7, 67% (45%)

A dried vial (4 mL) was charged with $Cu(OAc)_2$ (5 mol%), DPPE (5 mol%), B_2pin_2 (3.5 equiv.), and a stirring bar. When the NaOEt (2.75 equiv.) is added, the vial was sealed by PTFE/white rubber septum (Wheaton 13 mm Septa) and phenolic cap immediately. The vial was evacuated under vacuum and recharged with argon for three times with a needle. Then, *trans*-*β*-methylstyrene (0.2 mmol), aniline-d_7 (2.5 equiv.), and DMSO-d_6 (1.0 mL) were added under argon by using a syringe. The vial (or several vials) was placed in an alloy plate and transferred into a 300 mL autoclave of the 4560 series from Parr Instruments. The autoclave was charged with CO (10 bar) after flushing two times with N_2 and two times with CO. The autoclave was then placed into an aluminum block on a magnetic stirrer. The reaction was performed for 20 h at 60 °C. After the reaction was complete, the autoclave was cooled down to room temperature and the pressure was released carefully. The reaction was quenched with water (2 mL) and extracted with EtOAc (2 mLx3). The organic layer was washed with water (10 mL) and dried over anhydrous Na_2SO_4 and concentrated under reduced pressure. After that the corresponding product (**1a-D7**) was afforded as white solid by directly purified by fast column chromatography. (For NMR yield: The organic layer was washed with water and 0.1 mmol of 1,3,5-trimethoxybenzene was added. After drying with anhydrous Na_2SO_4, an appropriate amount of organic layer was concentrated under reduced pressure to test the NMR yield.).

^{1}H NMR (300 MHz, $CDCl_3$) δ 7.25 (ddt, *J* = 15.1, 11.1, 7.1 Hz, 5H), 6.96 (s, 1H), 3.15 (dd, *J* = 10.0, 7.4 Hz, 1H), 1.51 – 1.40 (m, 1H), 1.29 (d, *J* = 1.5 Hz, 12H), 0.81 (d, *J* = 7.4 Hz, 3H).

^{13}C NMR (75 MHz, $CDCl_3$) δ 170.0, 143.7, 137.7, 128.6, 127.9, 126.6, 83.4, 45.6, 24.9, 24.7, 14.0.

^{11}B NMR (96 MHz, $CDCl_3$) δ 33.5.

231201.f329.10.fid — ghq-p-403 — Au1H CDCl3 {C:\Bruker\TopSpin3.6.2} 2312 29 — 300.20MHz

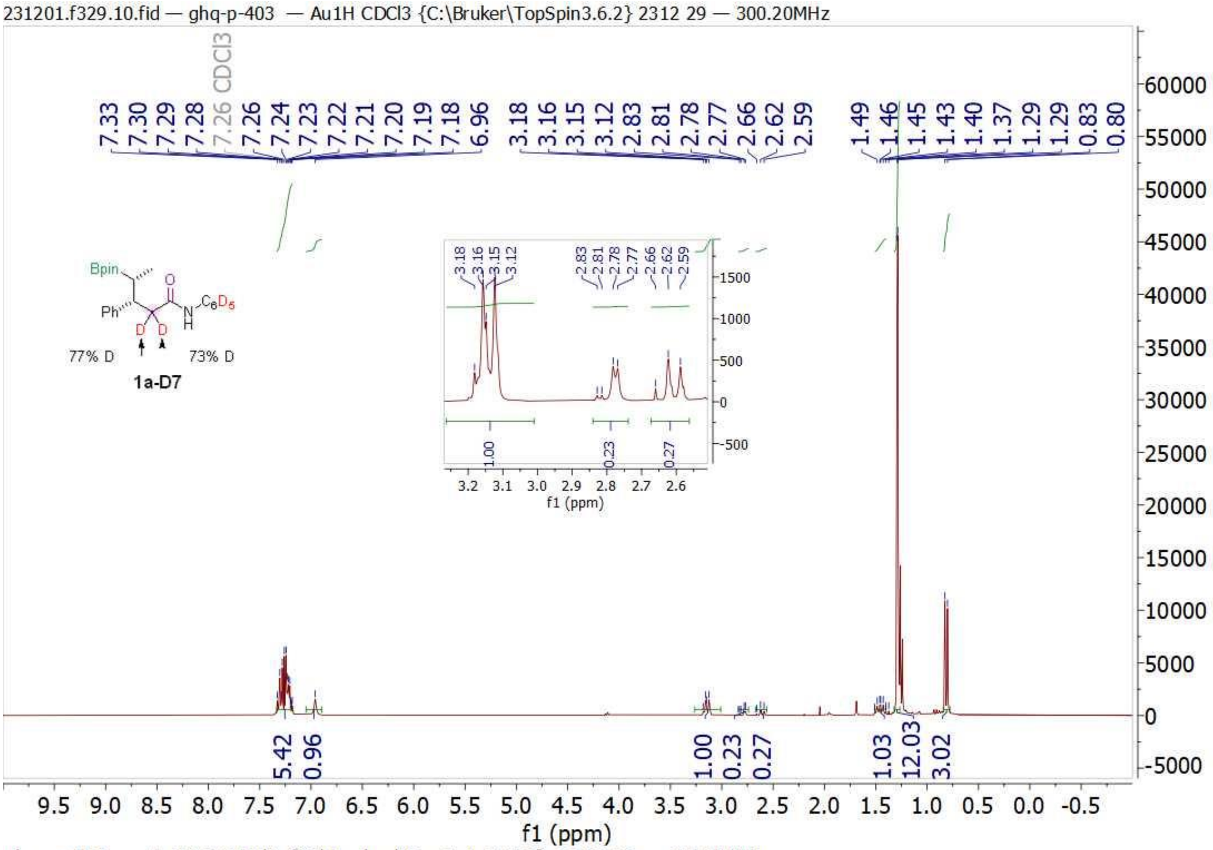

ghq-p-403 — Au13C CDCl3 {C:\Bruker\TopSpin3.6.2} 2312 29 — 75.50MHz

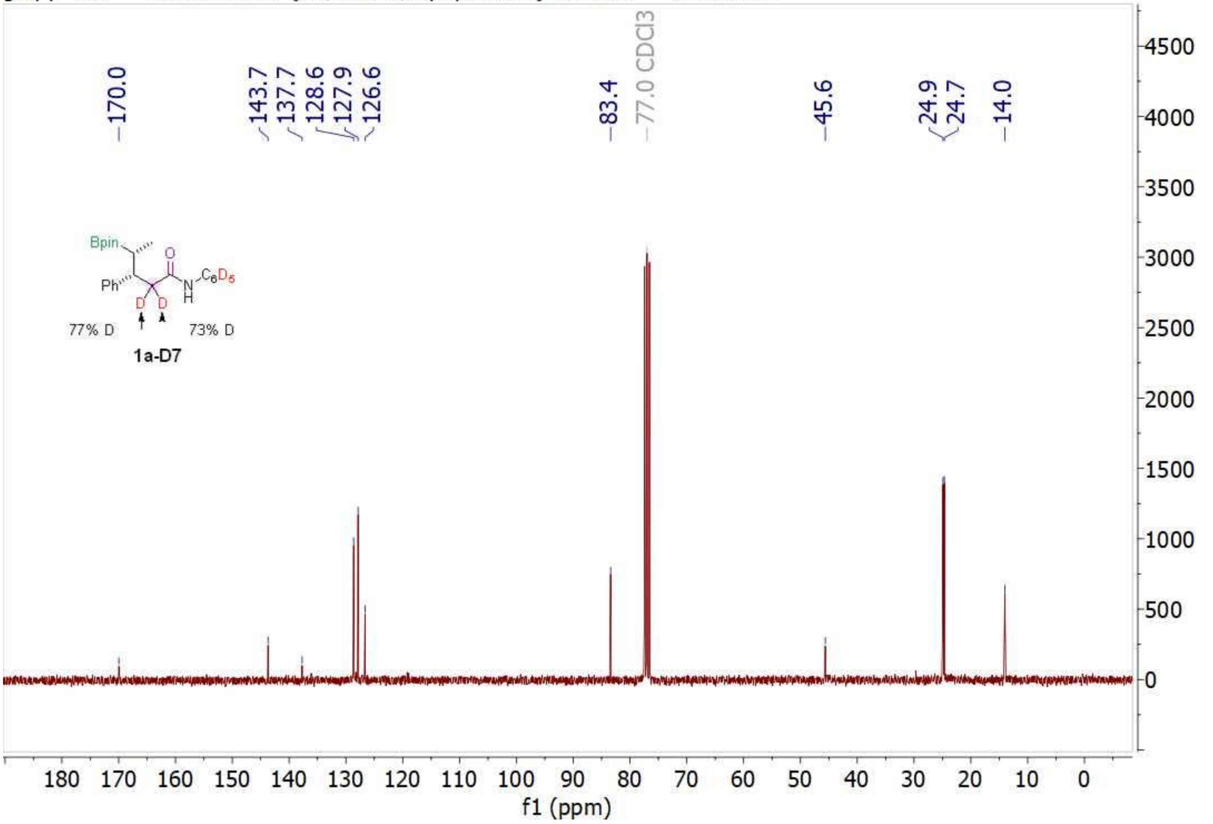

ghq-p-403 — Au11B(H-entk) CDCl3 {C:\Bruker\TopSpin3.6.2} 2312 29 — 96.32MHz

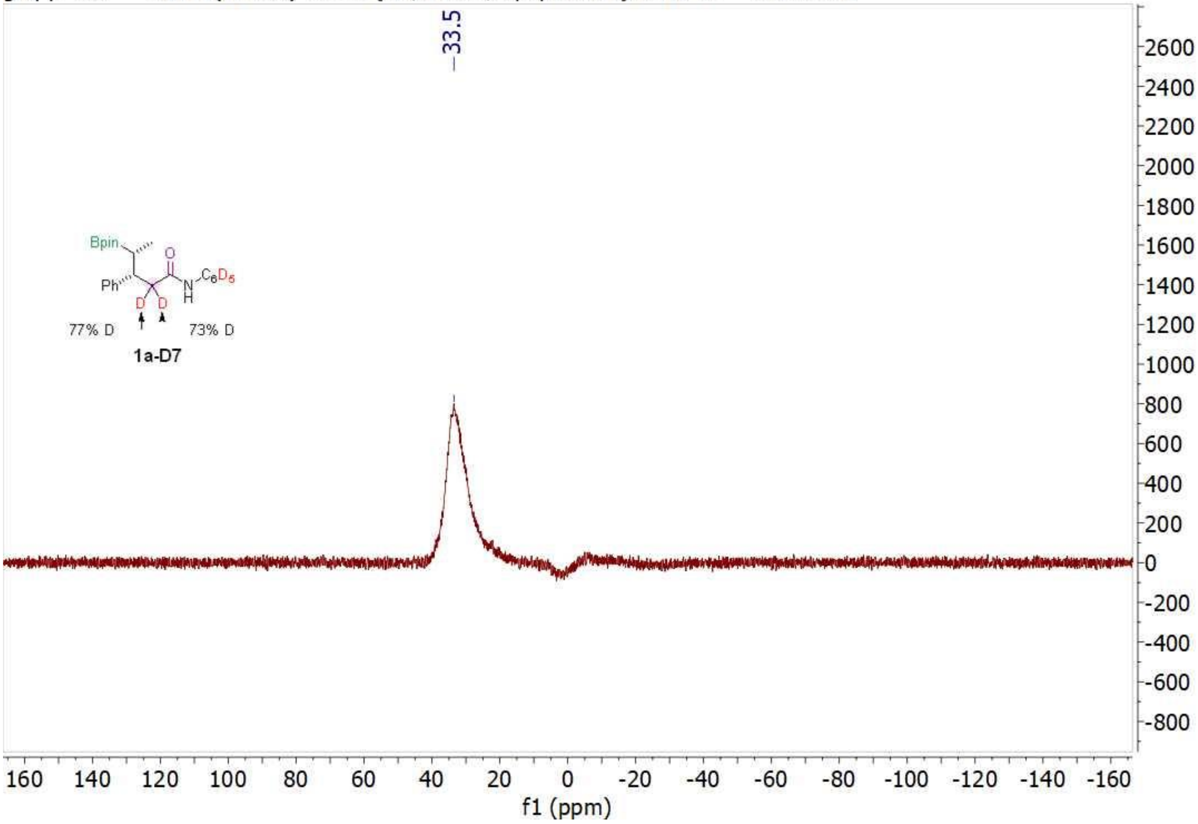

6.2.7 Mixed deuterated reagents of aniline-*d*$_7$, DMSO-*d*$_6$, and D_2O.

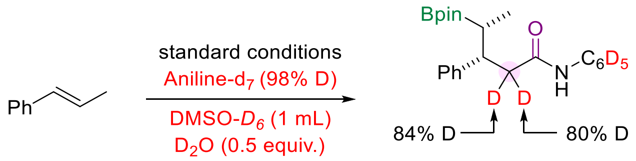

1a-D8, 37% (20%)

A dried vial (4 mL) was charged with $Cu(OAc)_2$ (5 mol%), DPPE (5 mol%), B_2pin_2 (3.5 equiv.), and a stirring bar. When the NaOEt (2.75 equiv.) is added, the vial was sealed by PTFE/white rubber septum (Wheaton 13 mm Septa) and phenolic cap immediately. The vial was evacuated under vacuum and recharged with argon for three times with a needle. Then, *trans-β*-methylstyrene (0.2 mmol), aniline-*d*$_7$ (2.5 equiv.), DMSO-*d*$_6$ (1.0 mL) and D_2O (0.5 equiv.) were added under argon by using a syringe. The vial (or several vials) was placed in an alloy plate and transferred into a 300 mL autoclave of the 4560 series from Parr Instruments. The autoclave was charged with CO (10 bar) after flushing two times with N_2 and two times with CO. The autoclave was then placed into an aluminum block on a magnetic stirrer. The reaction was performed for 20 h at 60 °C. After the reaction was complete, the autoclave was cooled down to room temperature and the pressure was released carefully. The reaction was quenched with water (2 mL) and extracted with EtOAc (2 mLx3). The organic layer was washed with water (10 mL) and dried over anhydrous Na_2SO_4 and concentrated under reduced pressure. After that the corresponding product (**1a-D8**) was afforded as white solid by directly purified by fast column chromatography. (For NMR yield: The organic layer was washed with water and 0.1 mmol of 1,3,5-trimethoxybenzene was added. After drying with anhydrous Na_2SO_4, an appropriate amount of organic layer was concentrated under reduced pressure to test the NMR yield.).

^{1}H NMR (300 MHz, $CDCl_3$) δ 7.34 – 7.27 (m, 2H), 7.25 – 7.16 (m, 3H), 6.93 (s, 1H), 3.13 (d, *J* = 10.1 Hz, 1H), 1.46 (dd, *J* = 10.1, 7.5 Hz, 1H), 1.29 (d, *J* = 1.5 Hz, 12H), 0.81 (d, *J* = 7.4 Hz, 3H).

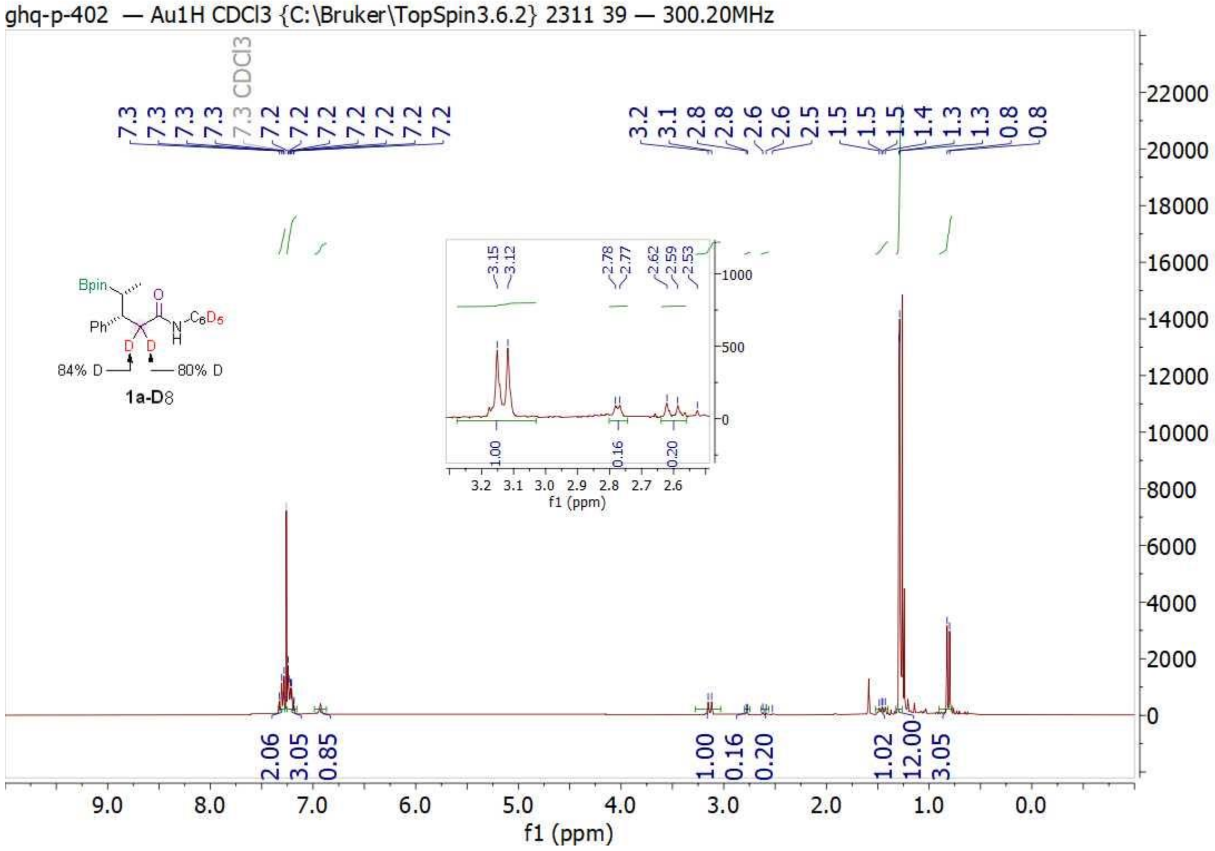
ghq-p-402 — Au1H CDCl3 {C:\Bruker\TopSpin3.6.2} 2311 39 — 300.20MHz
Bpin
Ph
C6D5
84% D
80% D
1a-D8
f1 (ppm)

7. X-ray crystal analysis of 1a.

1a

Data were collected on a Bruker Kappa APEX II Duo diffractometer. The structure was solved by direct methods (SHELXS-97: Sheldrick, G. M. *Acta Cryst.* **2008**, *A64*, 112.) and refined by full-matrix least-squares procedures on F^2 (SHELXL-2019: Sheldrick, G. M. *Acta Cryst.* **2015**, *C71*, 3.). XP (Bruker AXS) was used for graphical representations. CCDC 2283170 contains the supplementary crystallographic data for this paper. These data are provided free of charge by the joint Cambridge Crystallographic Data Centre and Fachinformationszentrum Karlsruhe Access Structures service www.ccdc.cam.ac.uk/structures.

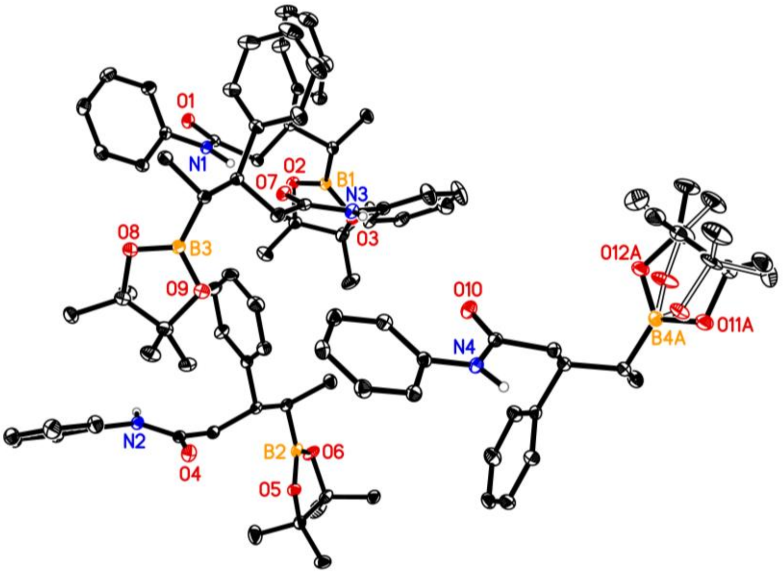

Molecular structure of **1a**. Displacement ellipsoids correspond to 30% probability. The pinacol boronic esters (tetramethyl-1,3,2-dioxaborolanes or “Bpin esters” for short) unit of one molecule is disordered over two sites with occupancies of 0.675(3):0.325(3). The lower occupied unit is shown with unfilled bonds. C-bound hydrogen atoms are omitted for clarity.

Table 1. Crystallographic Details

chemical formula	$C_{23}H_{30}BNO_3$
formula weight	379.29
crystal system	triclinic
unit cell dimensions	
a [Å]	13.6717(12)
b [Å]	16.5727(14)
c [Å]	19.6345(17)
a [deg]	85.336(2)
b [deg]	85.800(2)
g [deg]	78.852(2)
V [Å^3]	4342.8(7)
T [K]	110(2)
space group	$P\bar{1}$
Z	8
m [mm^{-1}]	0.075
density [g/cm^3]	1.160
no. of reflections measured	140929
no. of independent reflections	20961 (R_{int} = 0.0378)
no. of observed reflections ($I > 2\sigma(I)$)	16120
no. of parameters	1105
R_1 (I>2*s*(I))	0.0561
wR_2 (all data)	0.1572
Goodness of fit on F^2	1.025
largest diff. peak and hole [e/Å^3]	1.425 and -0.373

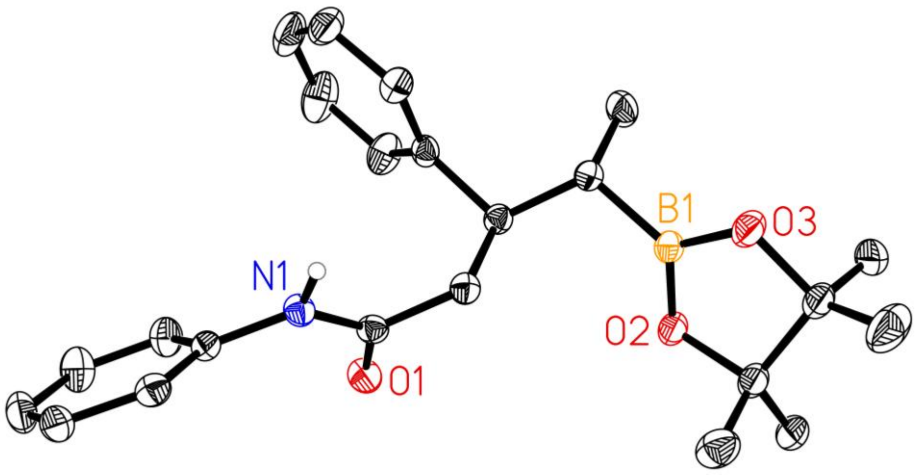

Molecular structure of one molecule of the asymmetric unit of **1a**. Displacement ellipsoids correspond to 50% probability. C-bound hydrogen atoms are omitted for clarity.

8. References.

(1) C. N. Farthing, S. P. Marsden, *Tetrahedron Lett.* **2000**, *41*, 4235-4238.

(2) R. P. Sonawane, V. Jheengut, C. Rabalakos, R. Larouche-Gauthier, H. K. Scott, V. K. Aggarwal, *Angew. Chem., Int. Ed.* **2011**, *50*, 3760-3763.

(3) M. Wang, H. Yu, X. You, J. Wu, Z. Shang, *Chin. J. Chem.* **2012**, *30*, 2356-2362.

(4) D. L. Sandrock, L. Jean-Gérard, C.-Y. Chen, S. D. Dreher, G. A. Molander, *J. Am. Chem. Soc.* **2010**, *132*, 17108-17110.

9. NMR spectra

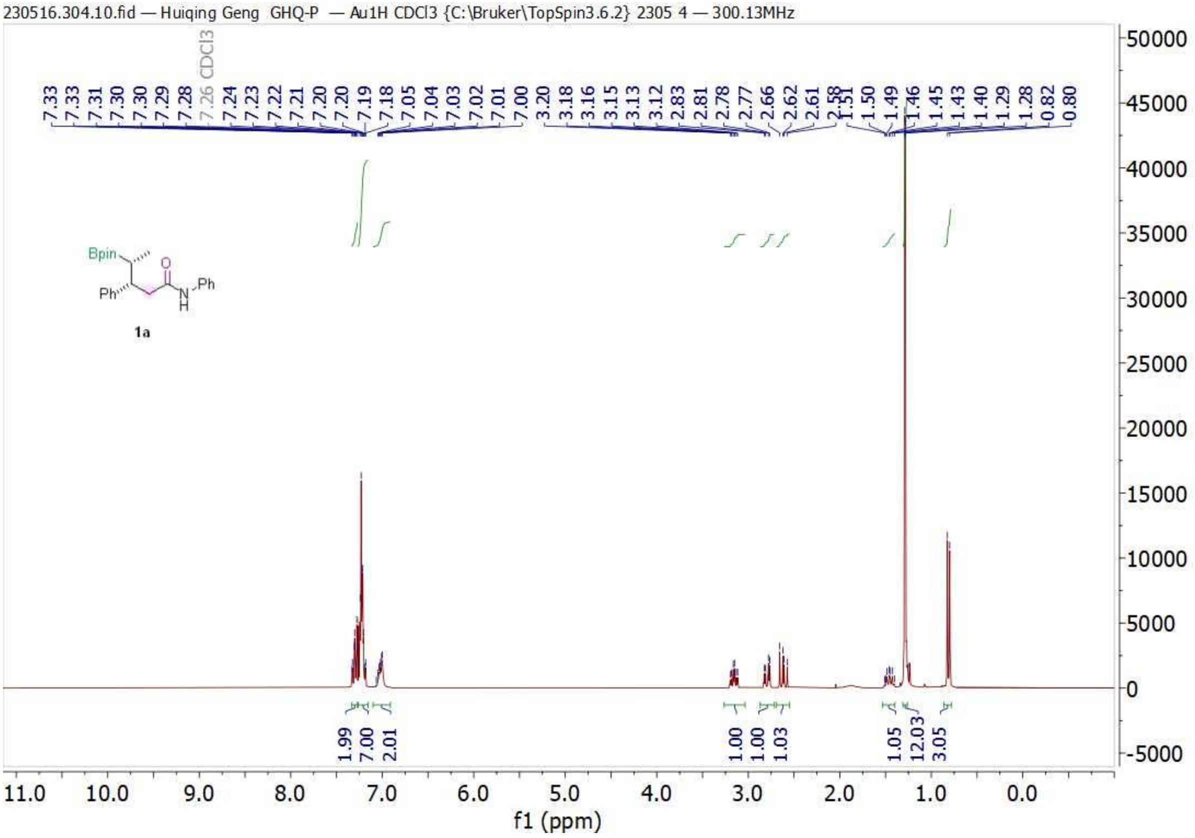

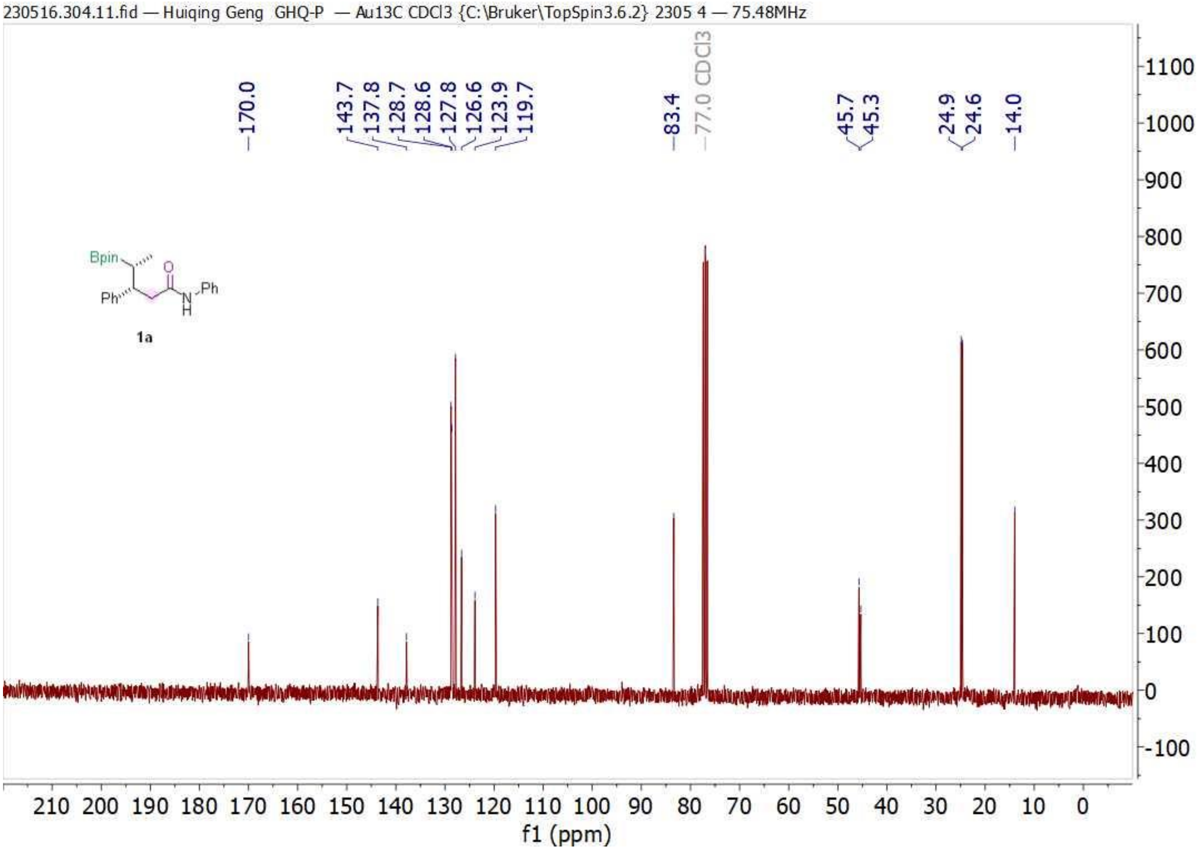

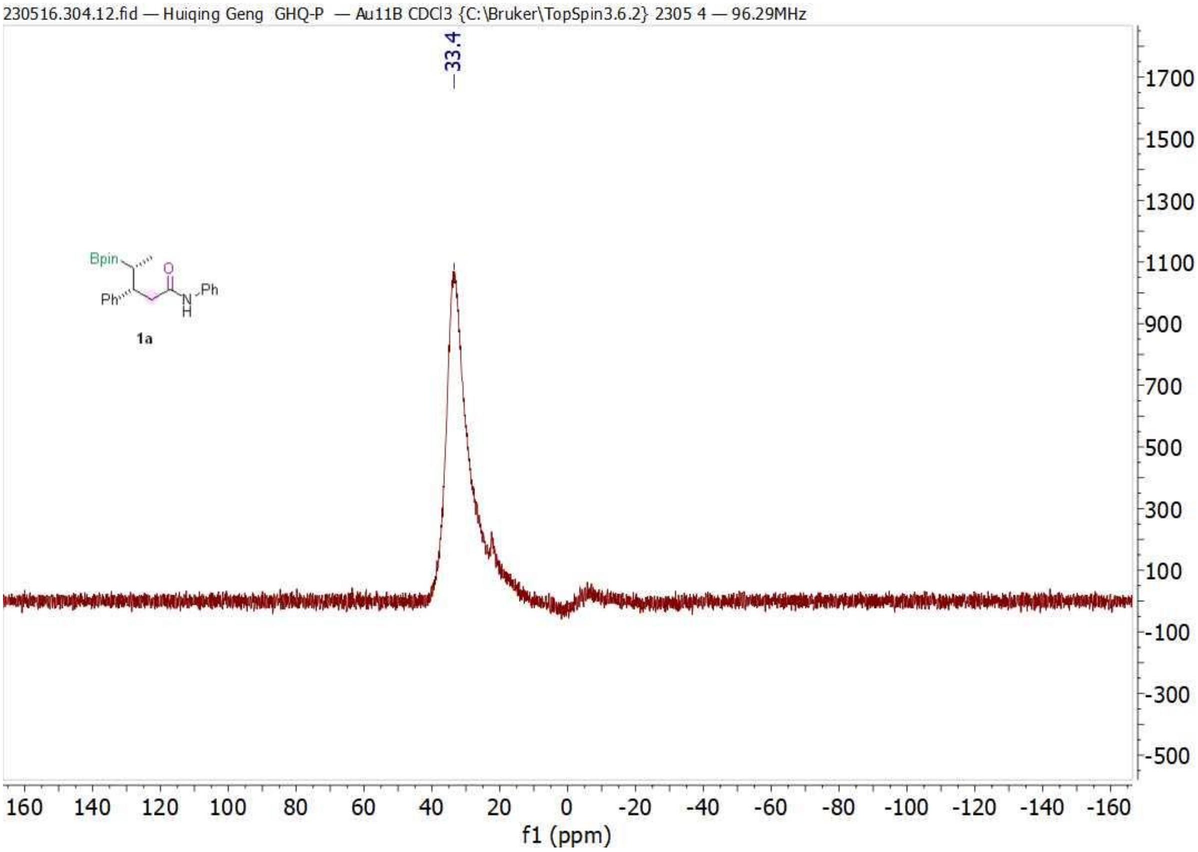
230516.304.12.fid — Huiqing Geng GHQ-P — Au11B CDCl3 {C:\Bruker\TopSpin3.6.2} 2305 4 — 96.29MHz
—33.4
Bpin
Ph
O
N
H
Ph
1a
1700
1500
1300
1100
900
700
500
300
100
-100
-300
-500
160 140 120 100 80 60 40 20 0 -20 -40 -60 -80 -100 -120 -140 -160
f1 (ppm)

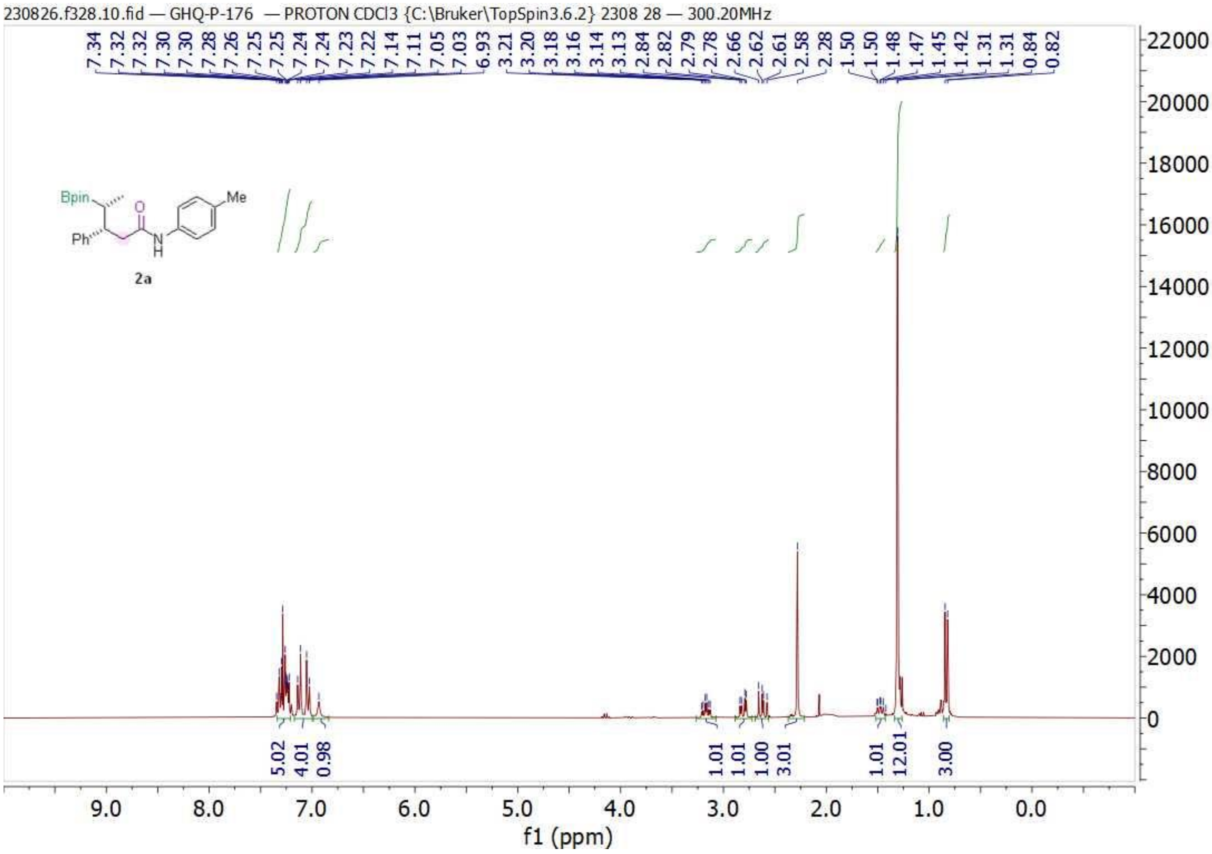
230826.f328.10.fid — GHQ-P-176 — PROTON CDCl3 {C:\Bruker\TopSpin3.6.2} 2308 28 — 300.20MHz
7.34 7.32 7.32 7.30 7.30 7.28 7.26 7.25 7.25 7.24 7.24 7.24 7.23 7.22 7.14 7.11 7.05 7.03 6.93 3.21 3.20 3.18 3.16 3.14 3.13 2.84 2.82 2.79 2.78 2.66 2.62 2.61 2.58 2.28 1.50 1.50 1.48 1.47 1.45 1.42 1.31 1.31 0.84 0.82
Bpin
Ph
O
N
H
Me
2a
5.02 4.01 0.98 1.01 1.01 1.00 3.01 1.01 12.01 3.00
f1 (ppm)

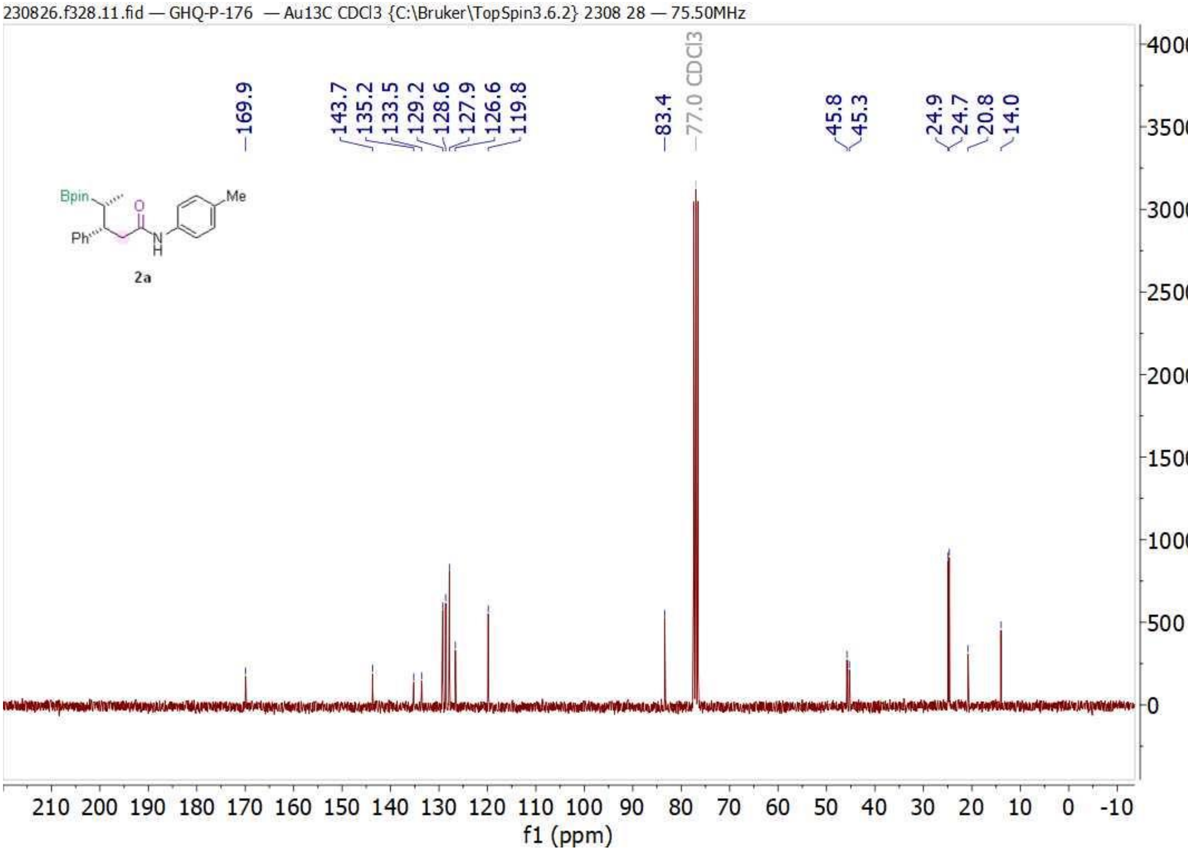
230826.f328.11.fid — GHQ-P-176 — Au13C CDCl3 {C:\Bruker\TopSpin3.6.2} 2308 28 — 75.50MHz
169.9
143.7 135.2 133.5 129.2 128.6 127.9 126.6 119.8
83.4
77.0 CDCl3
45.8 45.3
24.9 24.7 20.8 14.0
Bpin
Ph
O
N
H
Me
2a
f1 (ppm)

231116.f304.12.fid — Huiqing Geng GHQ-P-179 — Au11B(H-gek) CDCl3 {C:\Bruker\TopSpin3.6.2} 2311 4 — 96.32MHz

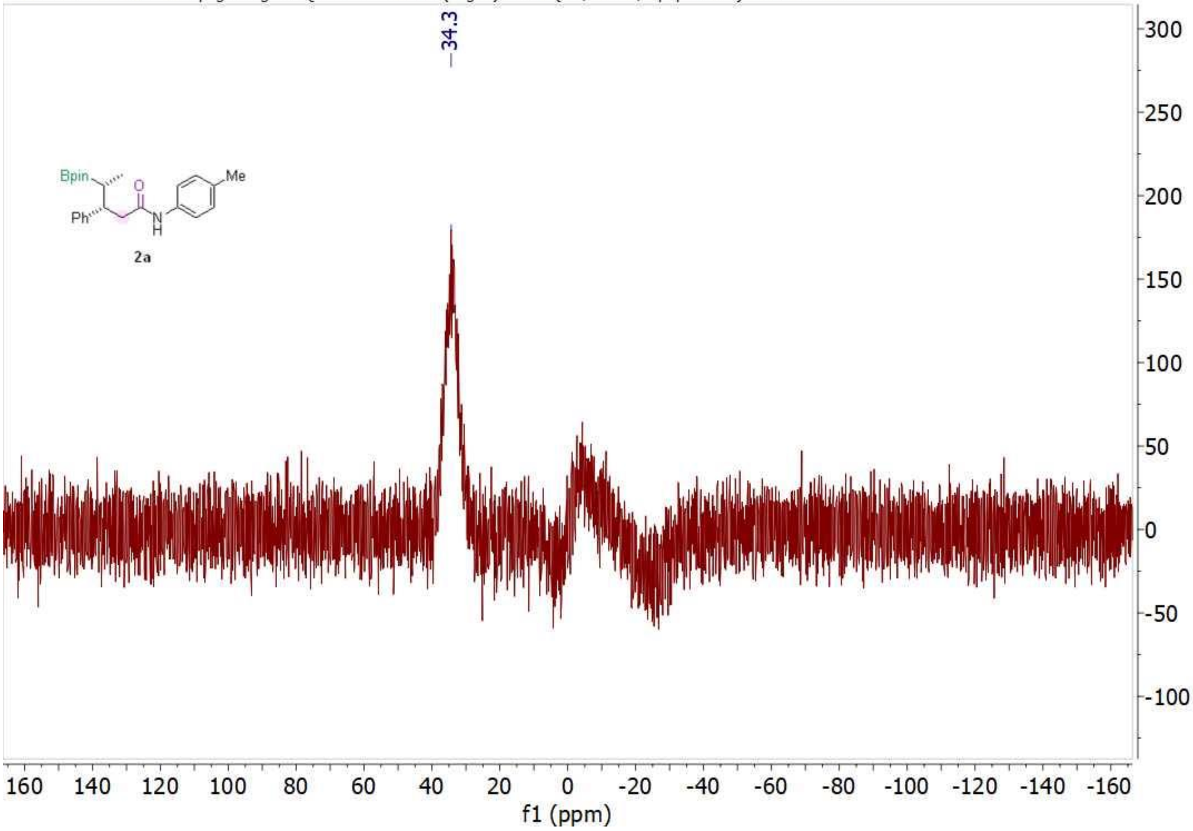

230713.308.10.fid — Huiqing Geng GHQ-P-158 — Au1H CDCl3 {C:\Bruker\TopSpin3.6.2} 2307 8 — 300.13MHz

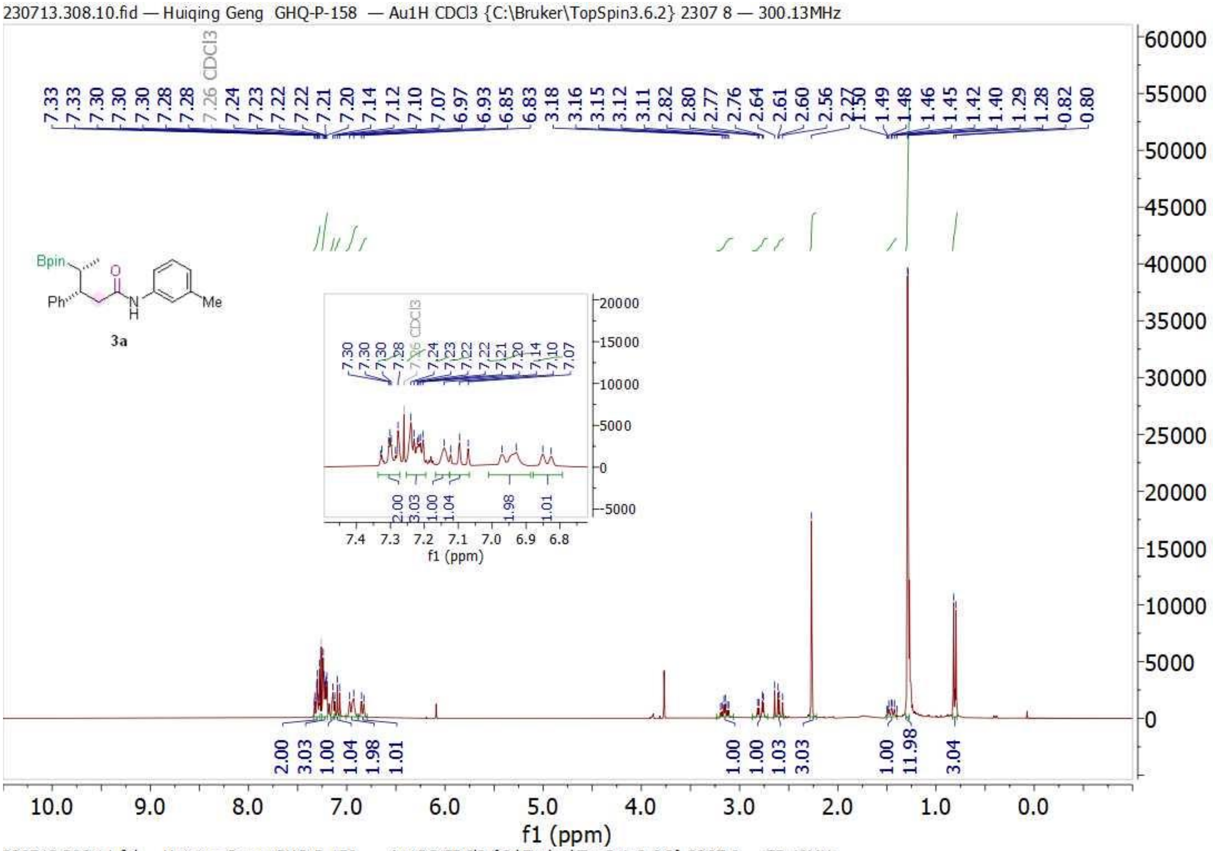

230713.308.11.fid — Huiqing Geng GHQ-P-158 — Au13C CDCl3 {C:\Bruker\TopSpin3.6.2} 2307 8 — 75.48MHz

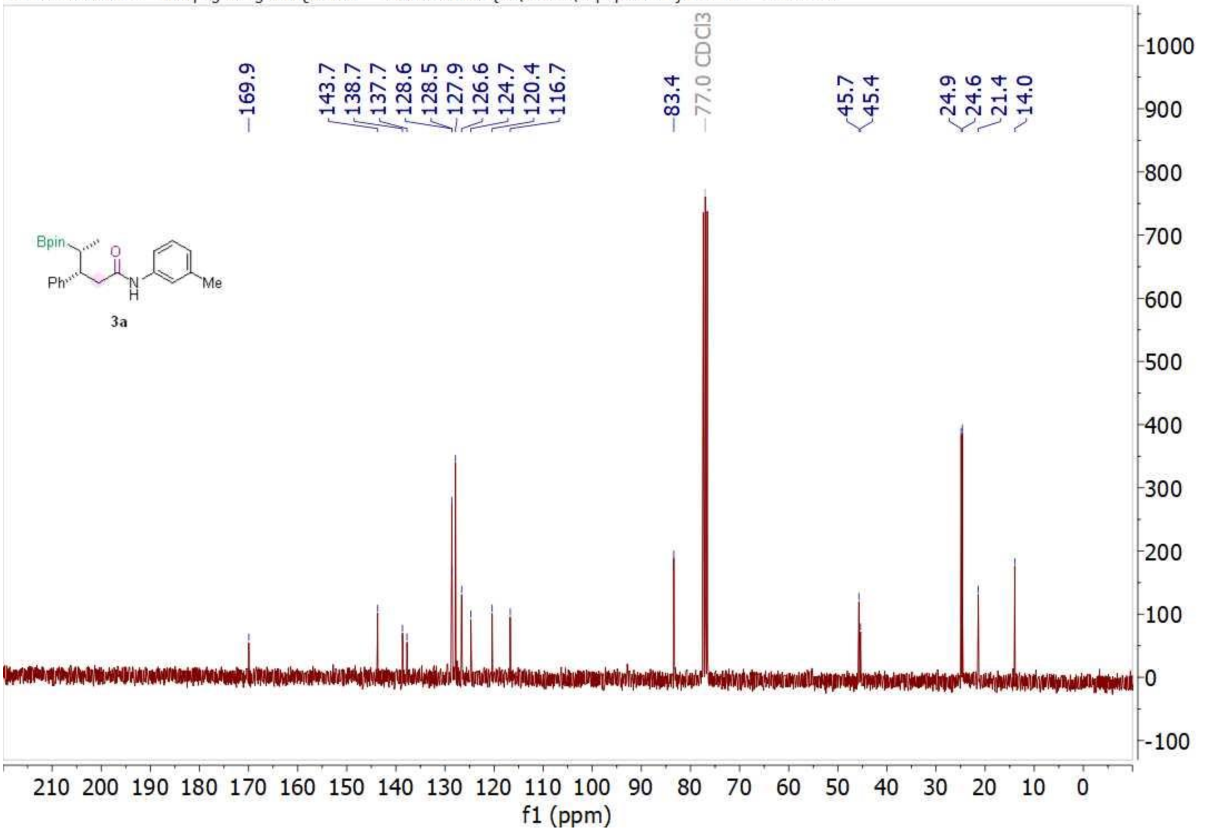

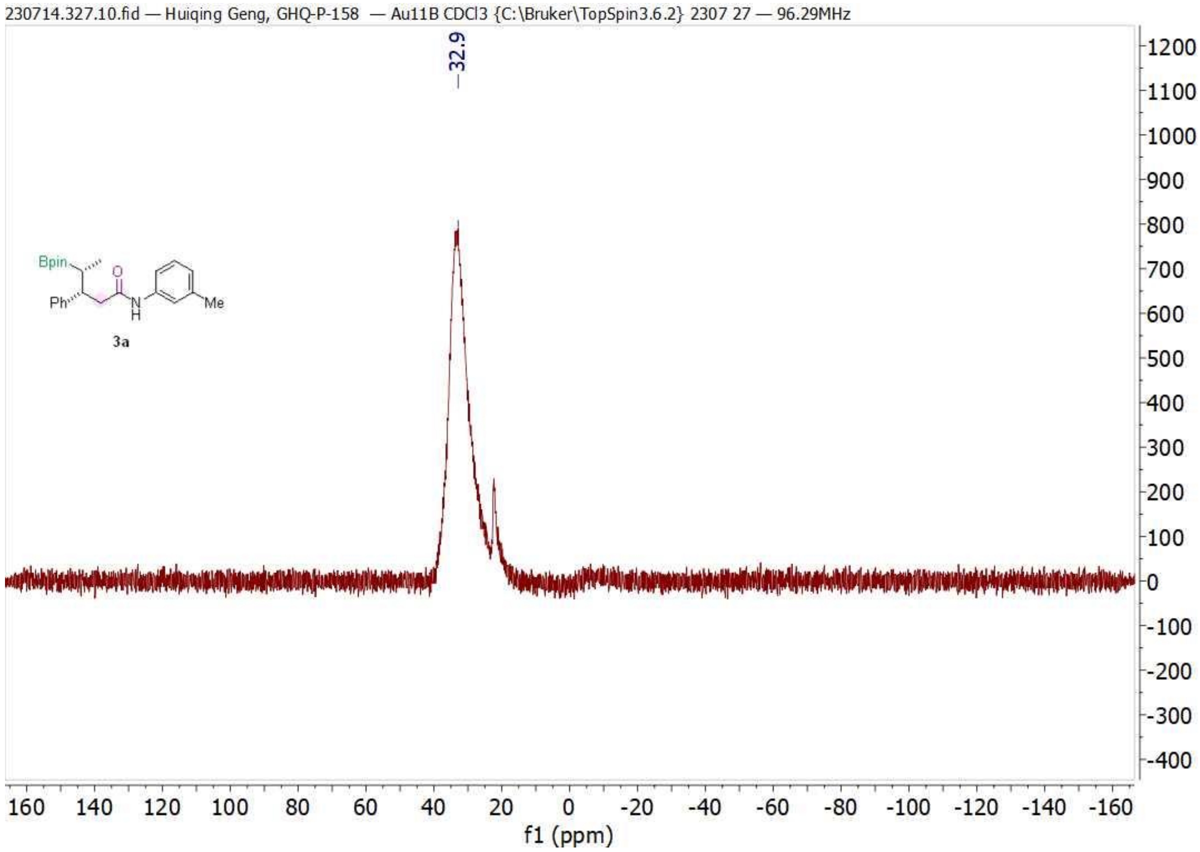
230714.327.10.fid — Huiqing Geng, GHQ-P-158 — Au11B CDCl3 {C:\Bruker\TopSpin3.6.2} 2307 27 — 96.29MHz
32.9
Bpin
Ph
O
N
H
Me
3a
f1 (ppm)

230815.303.10.fid — Huiqing Geng GHQ-P-219 — Au1H CDCl3 {C:\Bruker\TopSpin3.6.2} 2308 3 — 300.13MHz

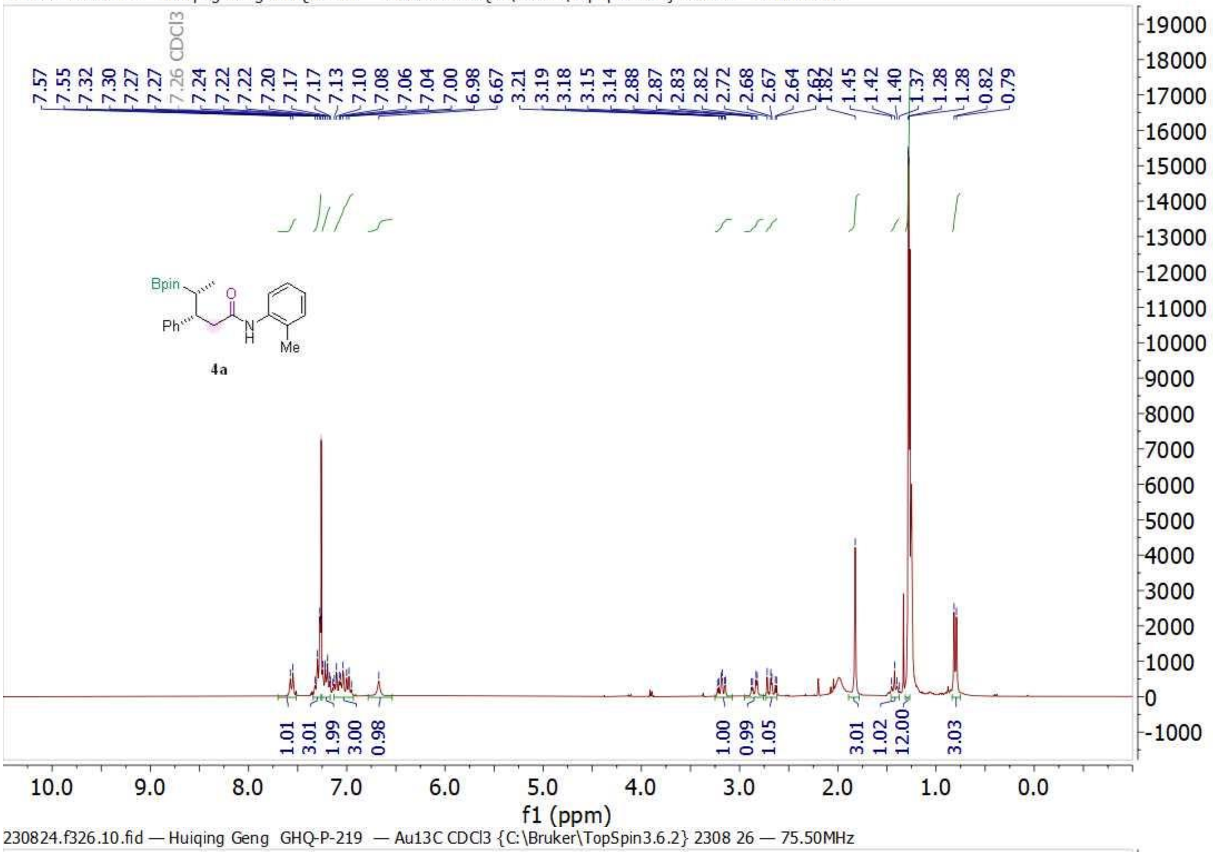

230824.f326.10.fid — Huiqing Geng GHQ-P-219 — Au13C CDCl3 {C:\Bruker\TopSpin3.6.2} 2308 26 — 75.50MHz

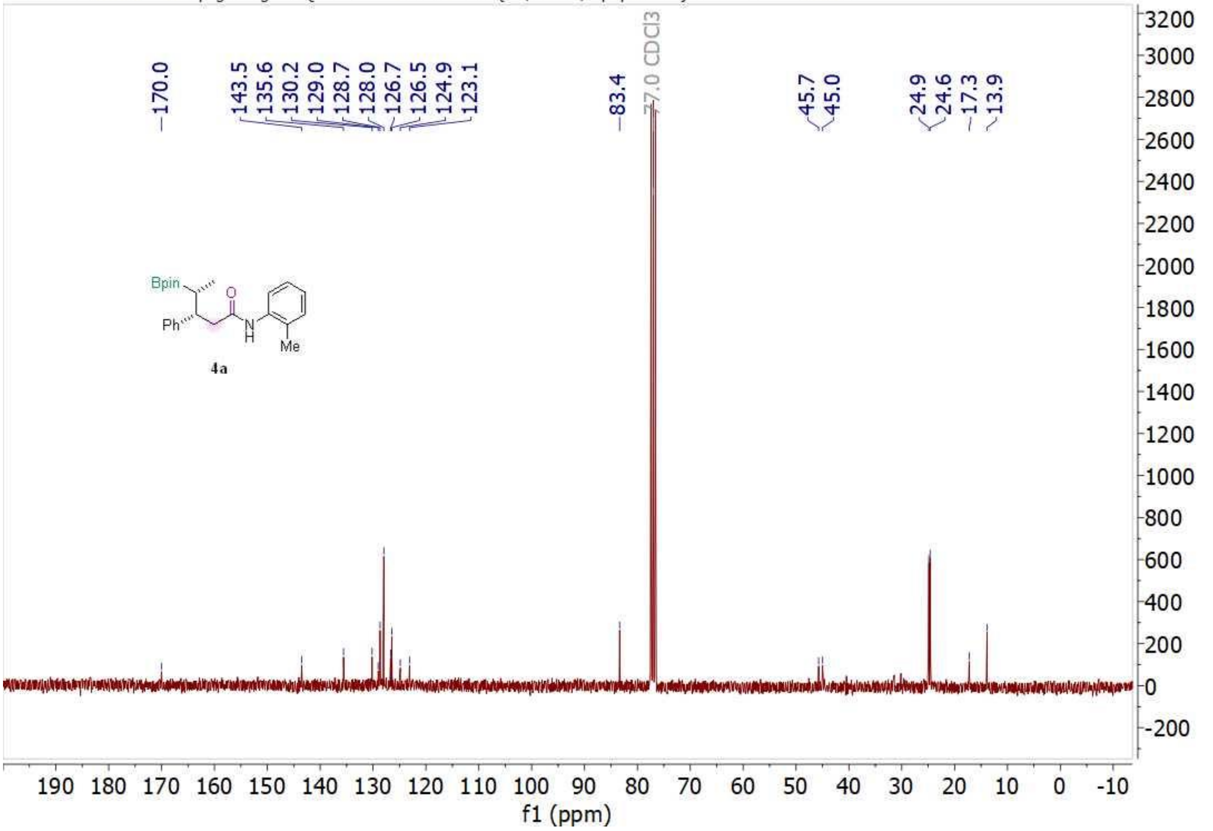

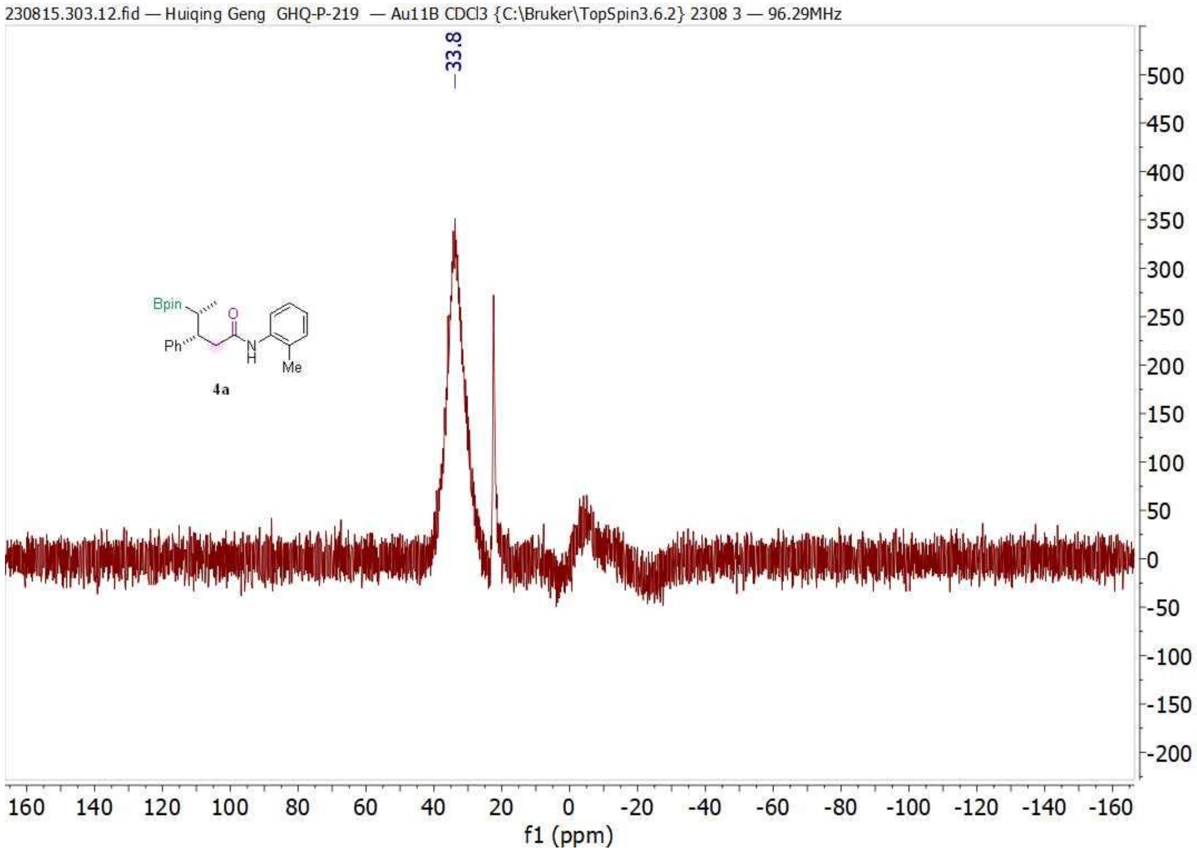
230815.303.12.fid — Huiqing Geng GHQ-P-219 — Au11B CDCl3 {C:\Bruker\TopSpin3.6.2} 2308 3 — 96.29MHz
33.8
Bpin
Ph
O
N
H
Me
4a
f1 (ppm)

230901.307.10.fid — Huiqing Geng GHQ-p-207 — Au1H CDCl3 {C:\Bruker\TopSpin3.6.2} 2309 7 — 300.13MHz

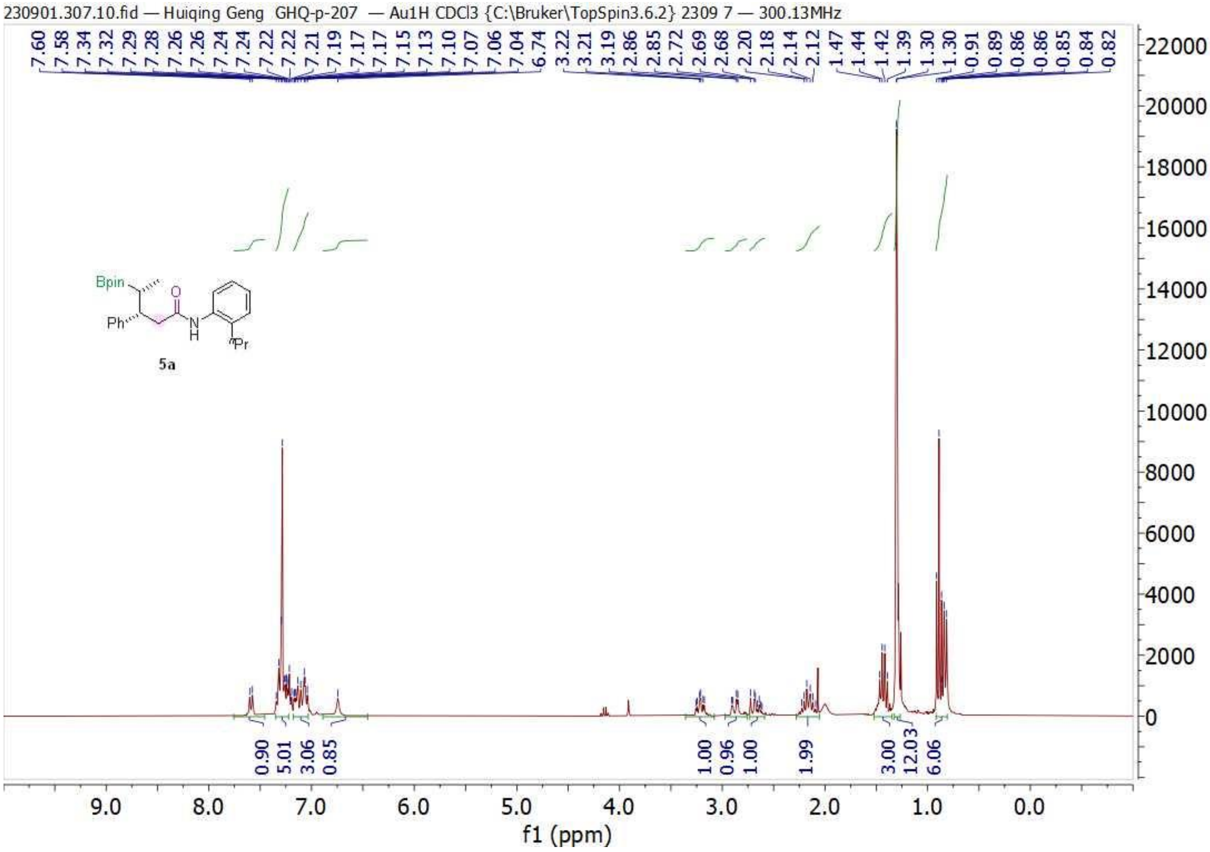

230901.307.11.fid — Huiqing Geng GHQ-p-207 — Au13C CDCl3 {C:\Bruker\TopSpin3.6.2} 2309 7 — 75.48MHz

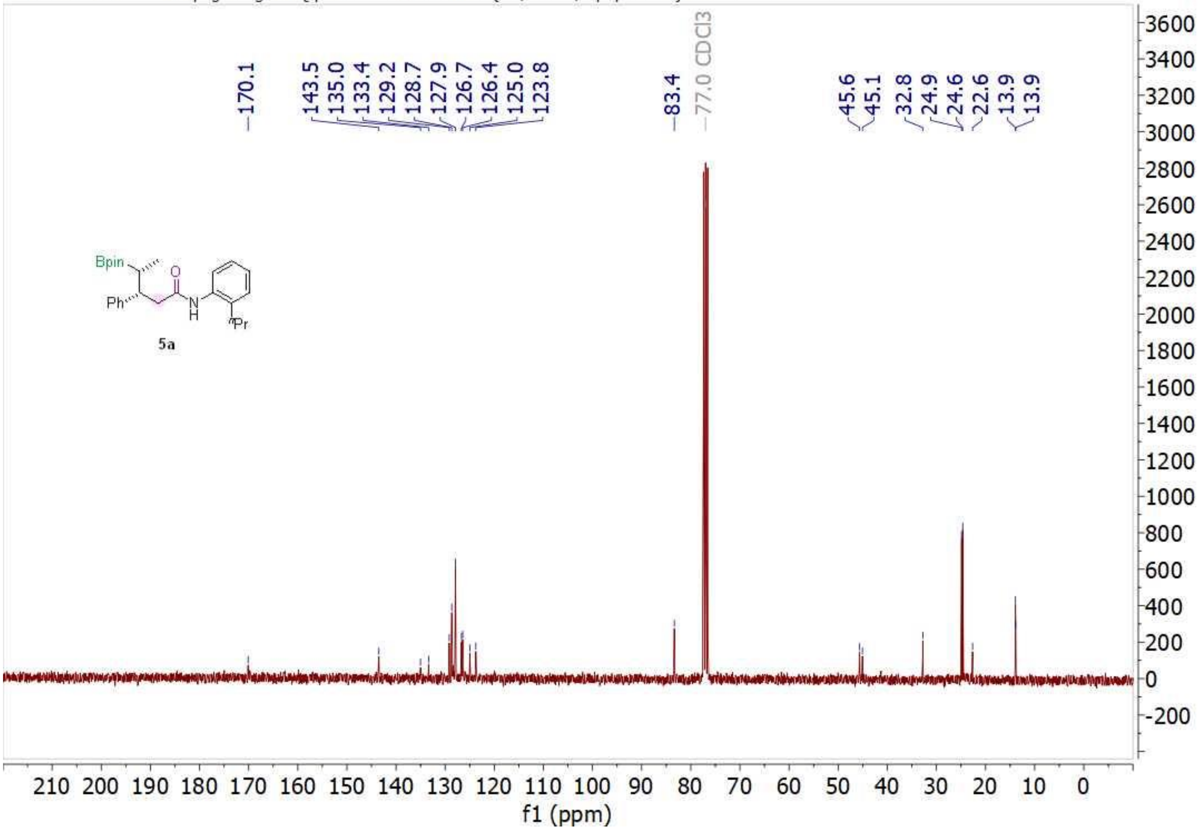

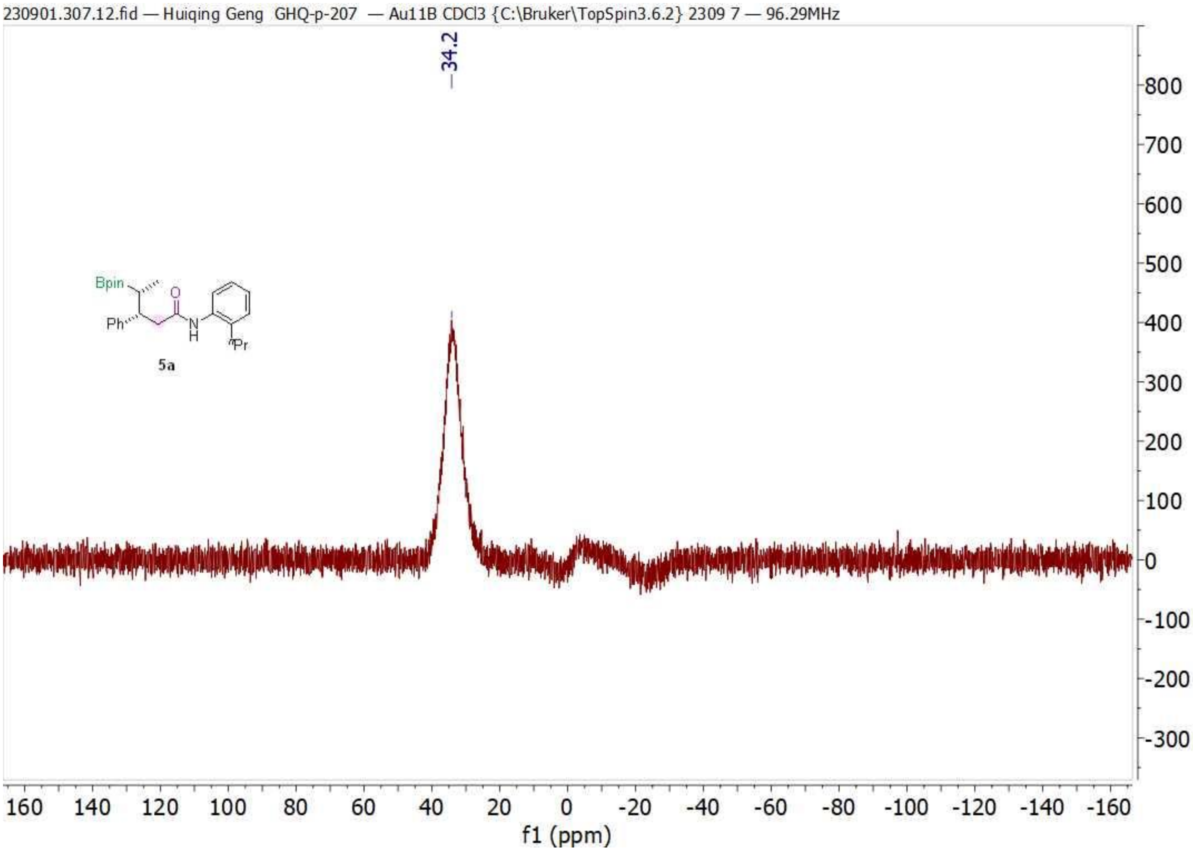

230901.307.12.fid — Huiqing Geng GHQ-p-207 — Au11B CDCl3 {C:\Bruker\TopSpin3.6.2} 2309 7 — 96.29MHz
34.2
Bpin
Ph
O
N
H
iPr
5a
800
700
600
500
400
300
200
100
0
-100
-200
-300
160 140 120 100 80 60 40 20 0 -20 -40 -60 -80 -100 -120 -140 -160
f1 (ppm)

230815.304.10.fid — Huiqing Geng GHQ-P-220 — Au1H CDCl3 {C:\Bruker\TopSpin3.6.2} 2308 4 — 300.13MHz

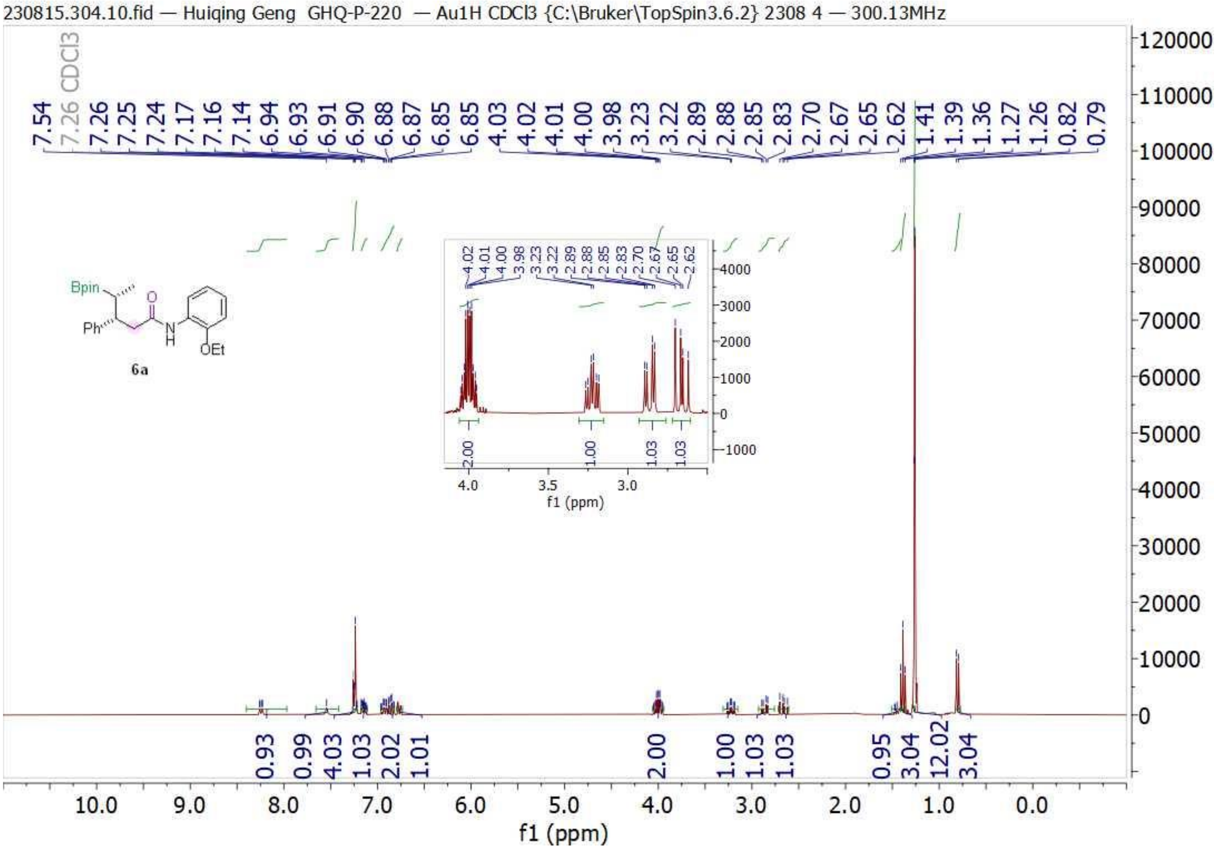

230815.304.11.fid — Huiqing Geng GHQ-P-220 — Au13C CDCl3 {C:\Bruker\TopSpin3.6.2} 2308 4 — 75.48MHz

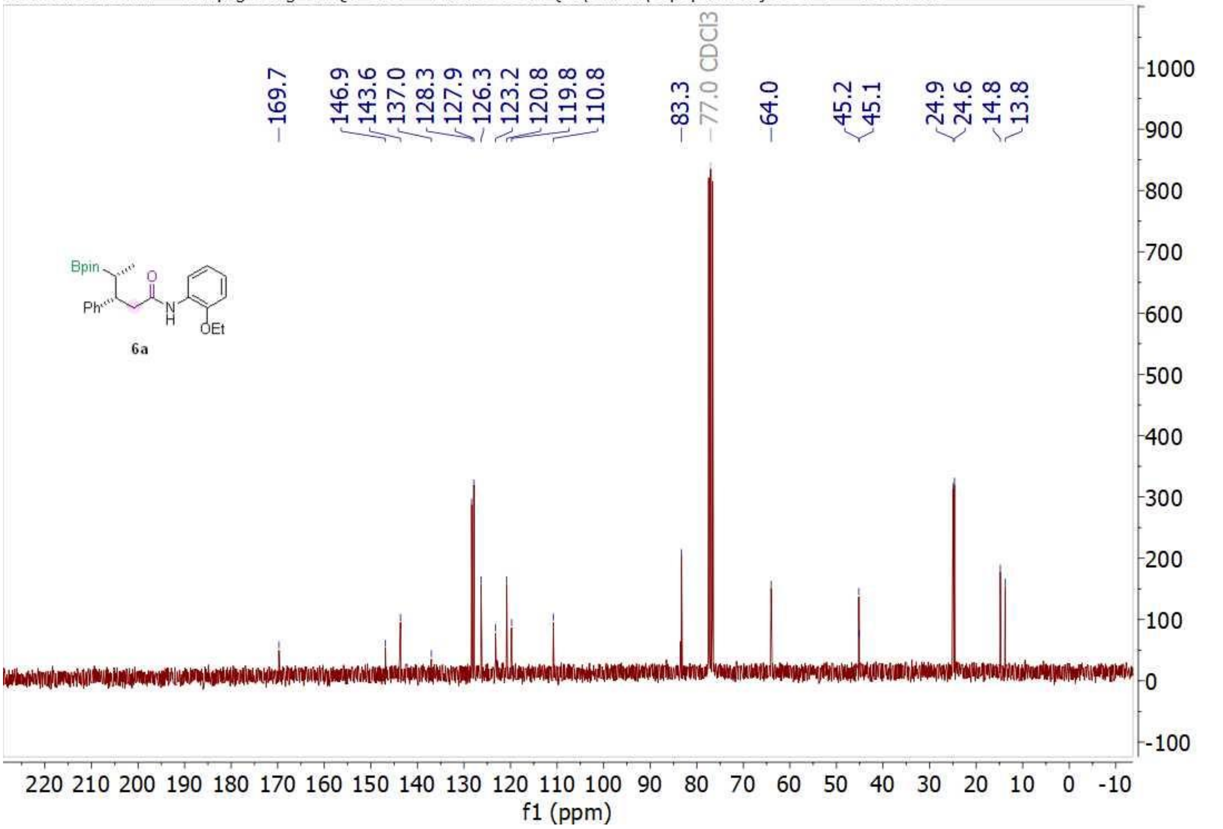

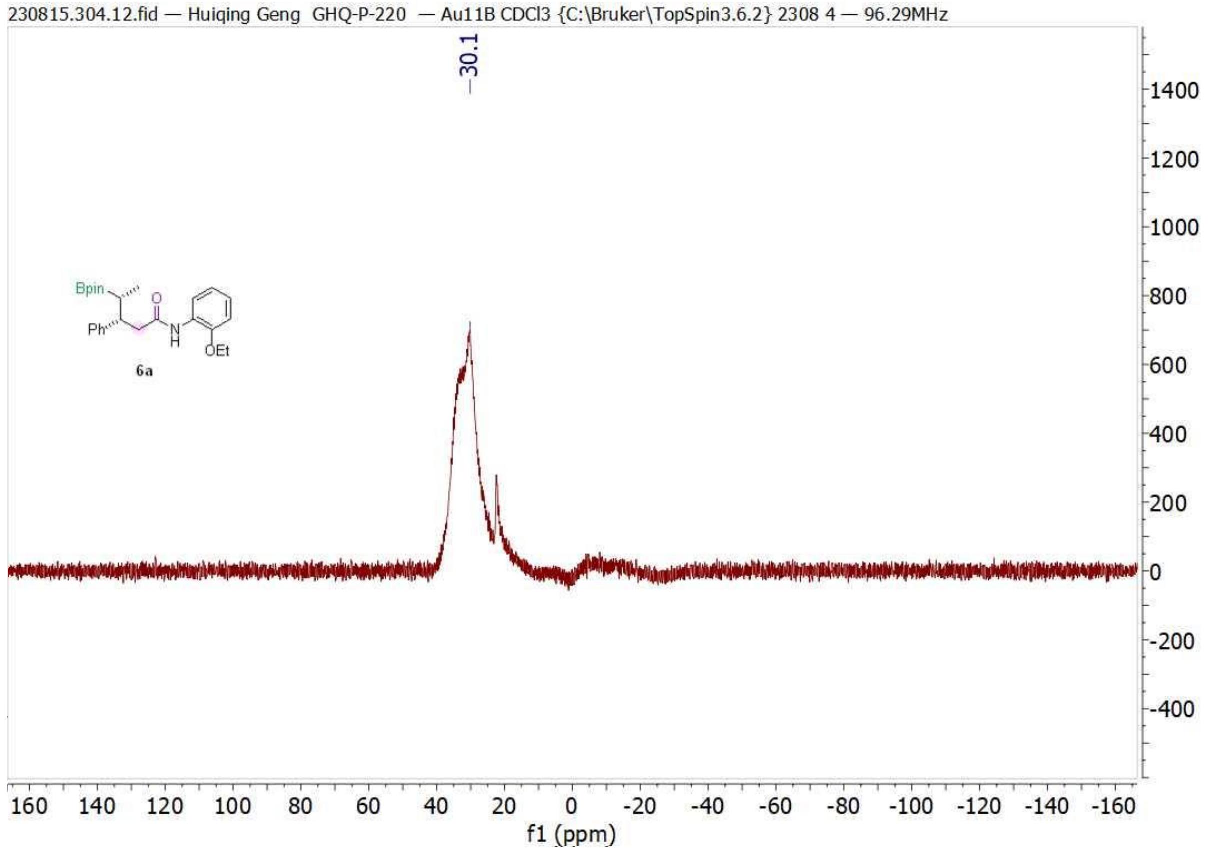
230815.304.12.fid — Huiqing Geng GHQ-P-220 — Au11B CDCl3 {C:\Bruker\TopSpin3.6.2} 2308 4 — 96.29MHz
30.1
Bpin
Ph
O
N
H
OEt
6a
f1 (ppm)

230817.421.10.fid — Huiqing Geng GHQ-P-223 — Au1H CDCl3 {C:\Bruker\TopSpin3.6.2} 2308 21 — 400.13MHz

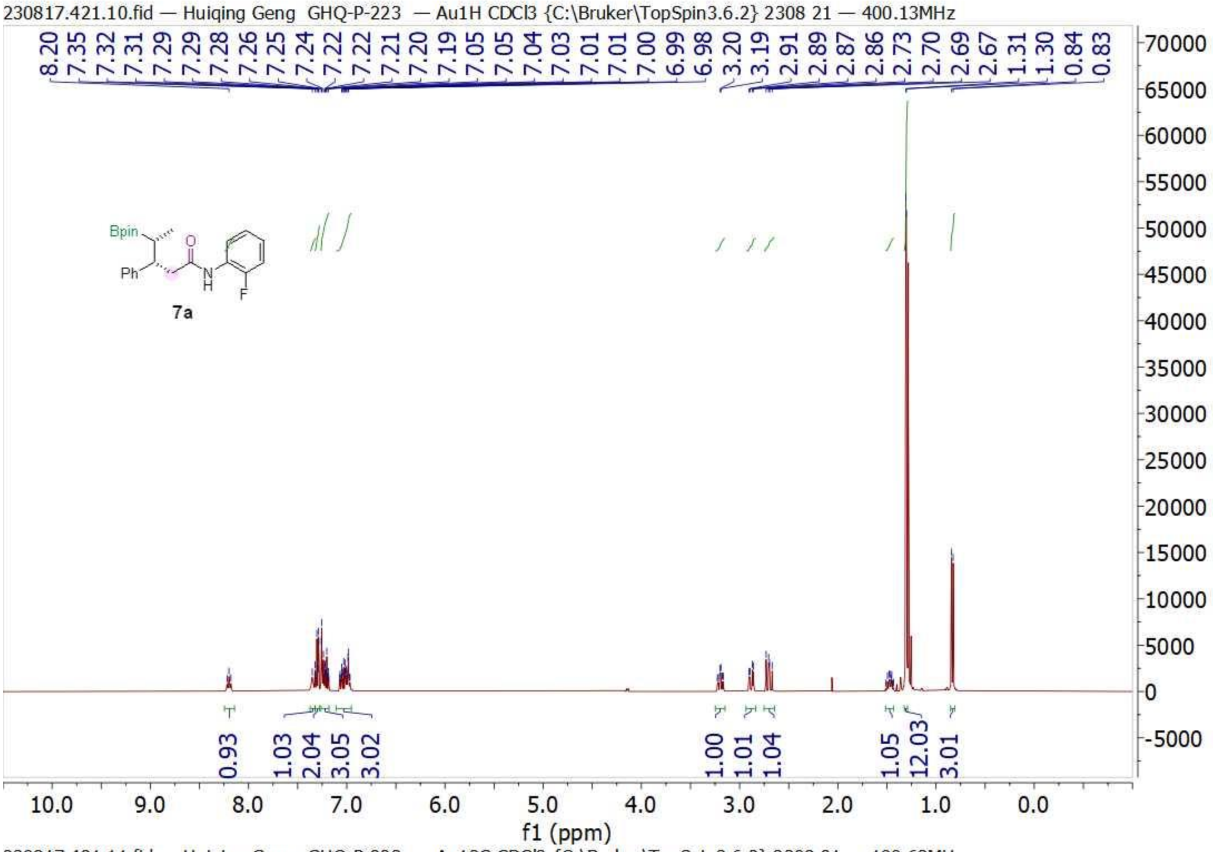

230817.421.11.fid — Huiqing Geng GHQ-P-223 — Au13C CDCl3 {C:\Bruker\TopSpin3.6.2} 2308 21 — 100.63MHz

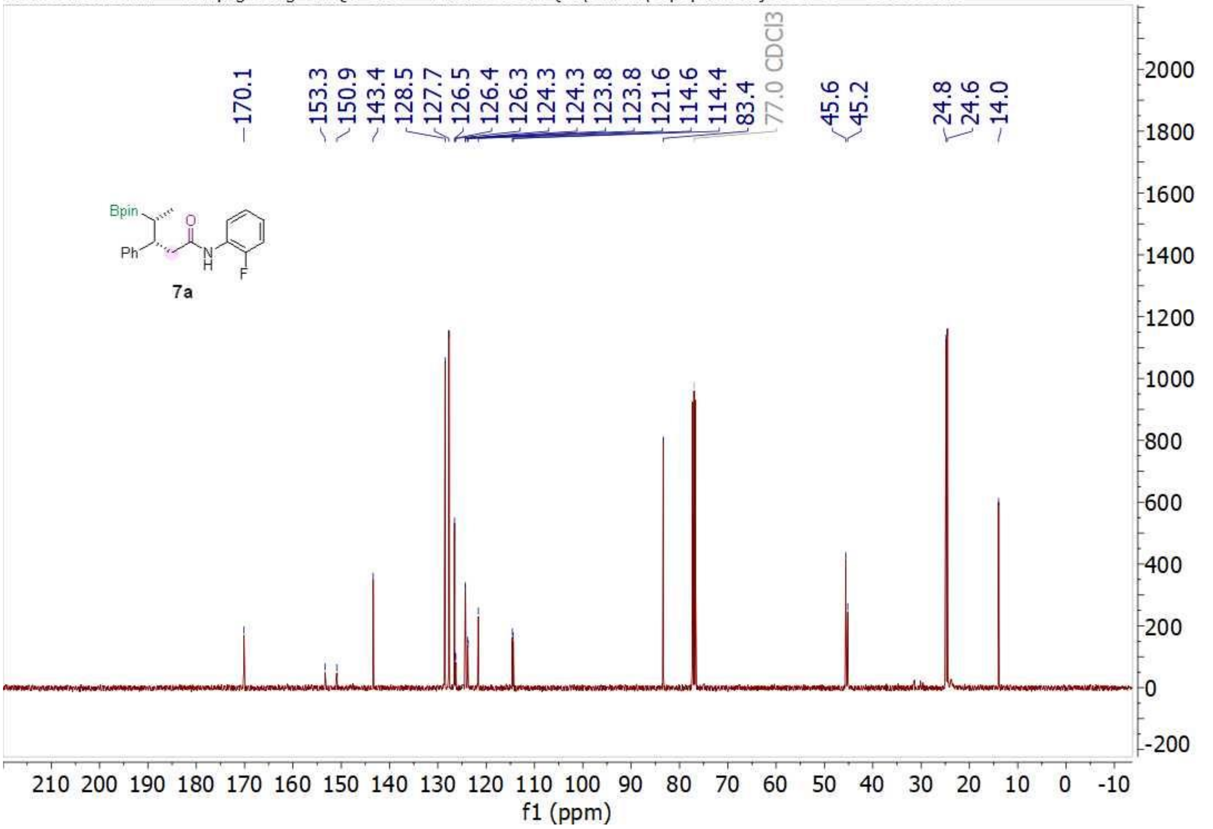

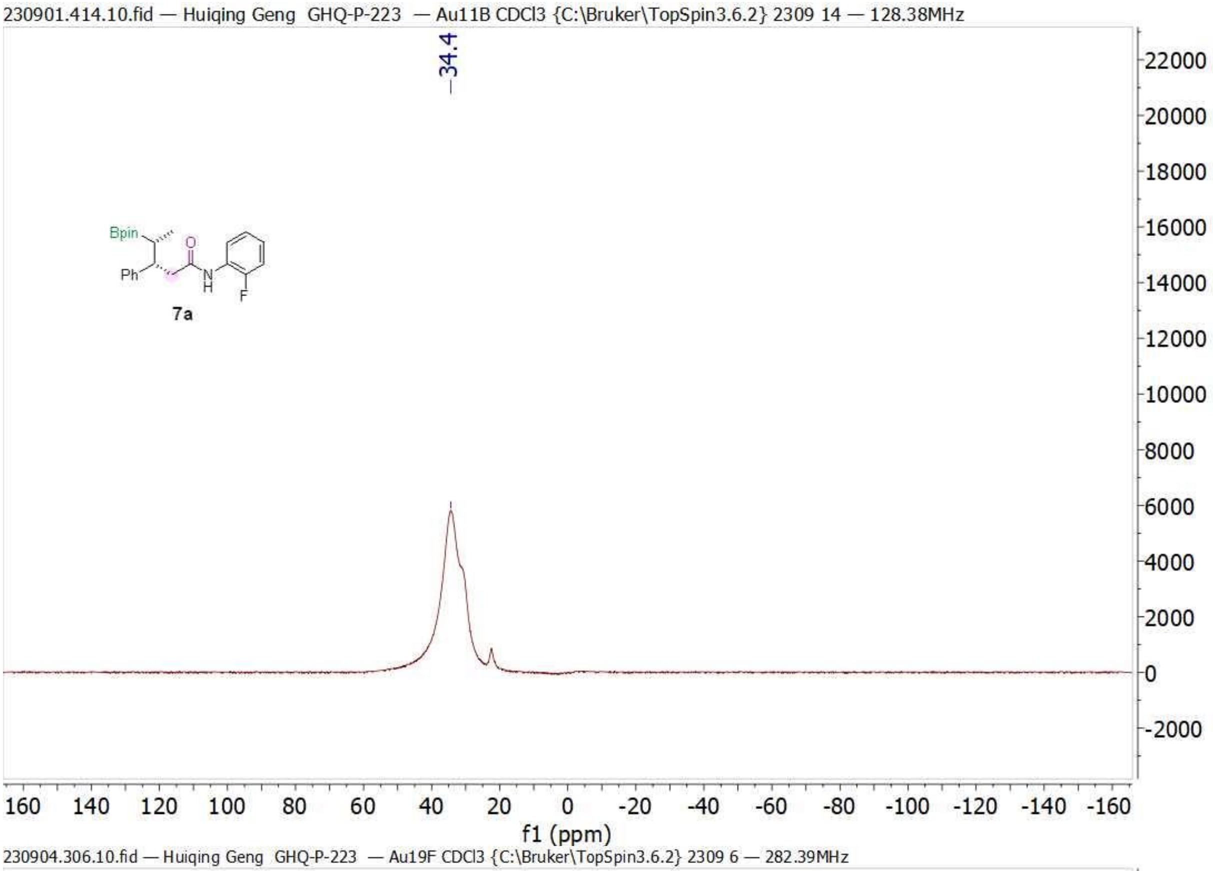
230901.414.10.fid — Huiqing Geng GHQ-P-223 — Au11B CDCl3 {C:\Bruker\TopSpin3.6.2} 2309 14 — 128.38MHz
—34.4
Bpin
O
Ph
N
H
F
7a
f1 (ppm)

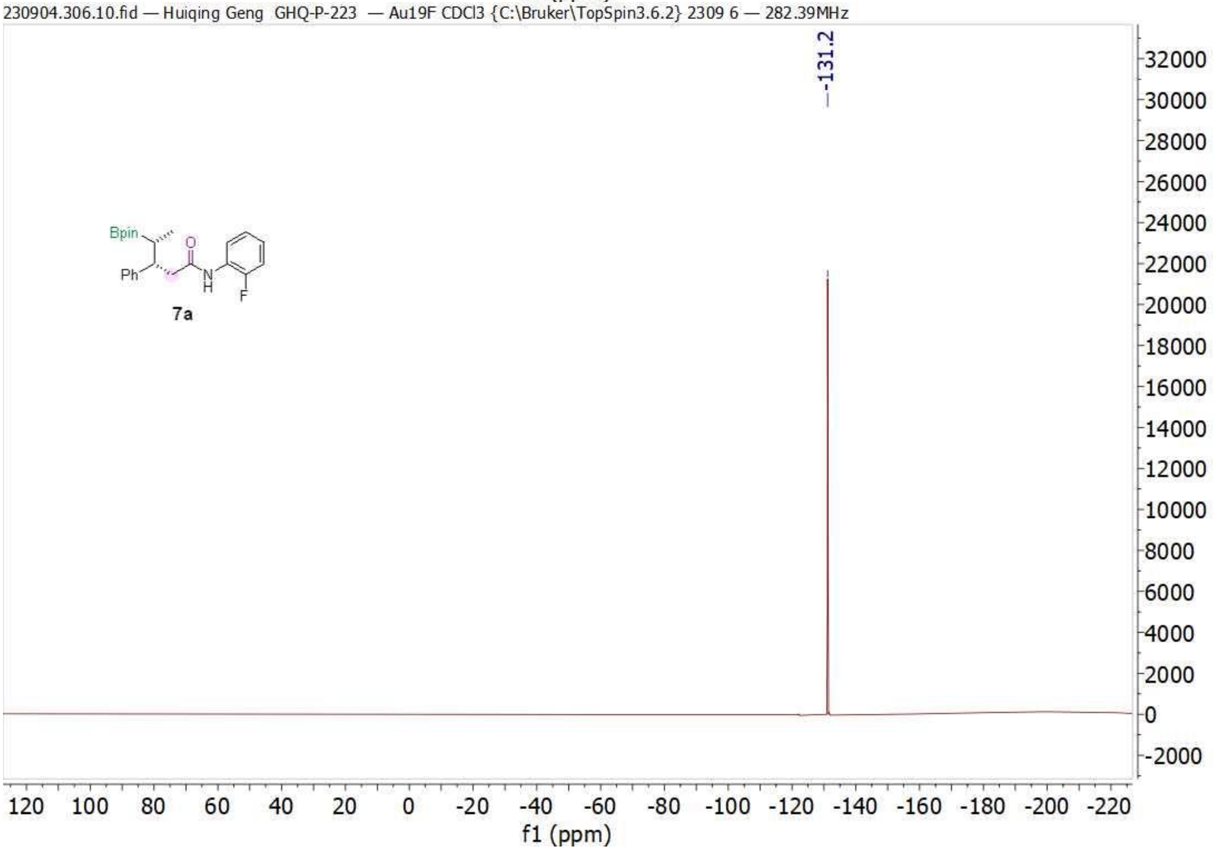
230904.306.10.fid — Huiqing Geng GHQ-P-223 — Au19F CDCl3 {C:\Bruker\TopSpin3.6.2} 2309 6 — 282.39MHz
—-131.2
Bpin
O
Ph
N
H
F
7a
f1 (ppm)

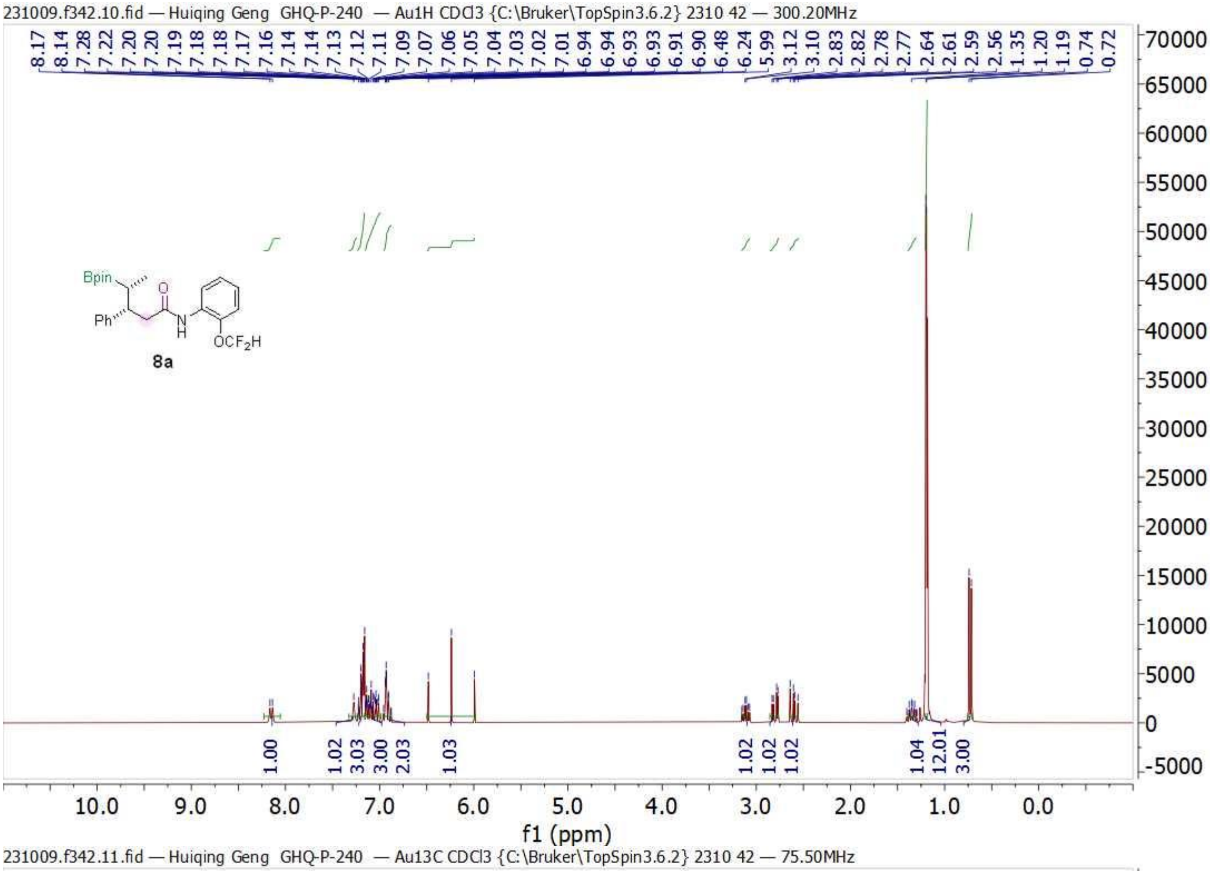
231009.f342.10.fid — Huiqing Geng GHQ-P-240 — Au1H CDCl3 {C:\Bruker\TopSpin3.6.2} 2310 42 — 300.20MHz
8.17 8.14 7.28 7.22 7.20 7.20 7.19 7.18 7.18 7.17 7.16 7.14 7.14 7.13 7.12 7.11 7.09 7.07 7.06 7.05 7.04 7.03 7.02 7.01 6.94 6.94 6.93 6.93 6.91 6.90 6.48 6.24 5.99 3.12 3.10 2.83 2.82 2.78 2.77 2.64 2.61 2.59 2.56 1.35 1.20 1.19 0.74 0.72
Bpin
Ph
O
N
H
OCF2H
8a
1.00 1.02 3.03 3.00 2.03 1.03 1.02 1.02 1.02 1.04 12.01 3.00
f1 (ppm)
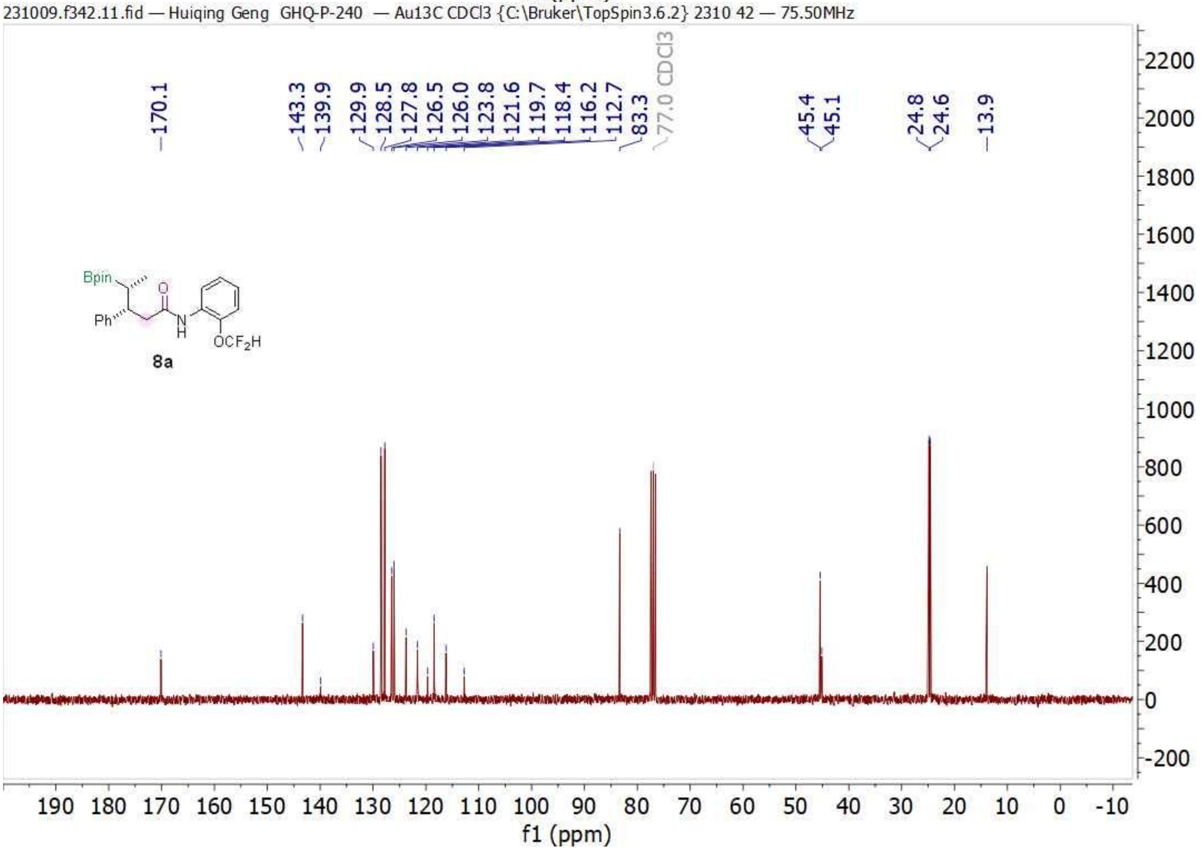
231009.f342.11.fid — Huiqing Geng GHQ-P-240 — Au13C CDCl3 {C:\Bruker\TopSpin3.6.2} 2310 42 — 75.50MHz
170.1 143.3 139.9 129.9 128.5 127.8 126.5 126.0 123.8 121.6 119.7 118.4 116.2 112.7 83.3 77.0 CDCl3 45.4 45.1 24.8 24.6 13.9
Bpin
Ph
O
N
H
OCF2H
8a
f1 (ppm)

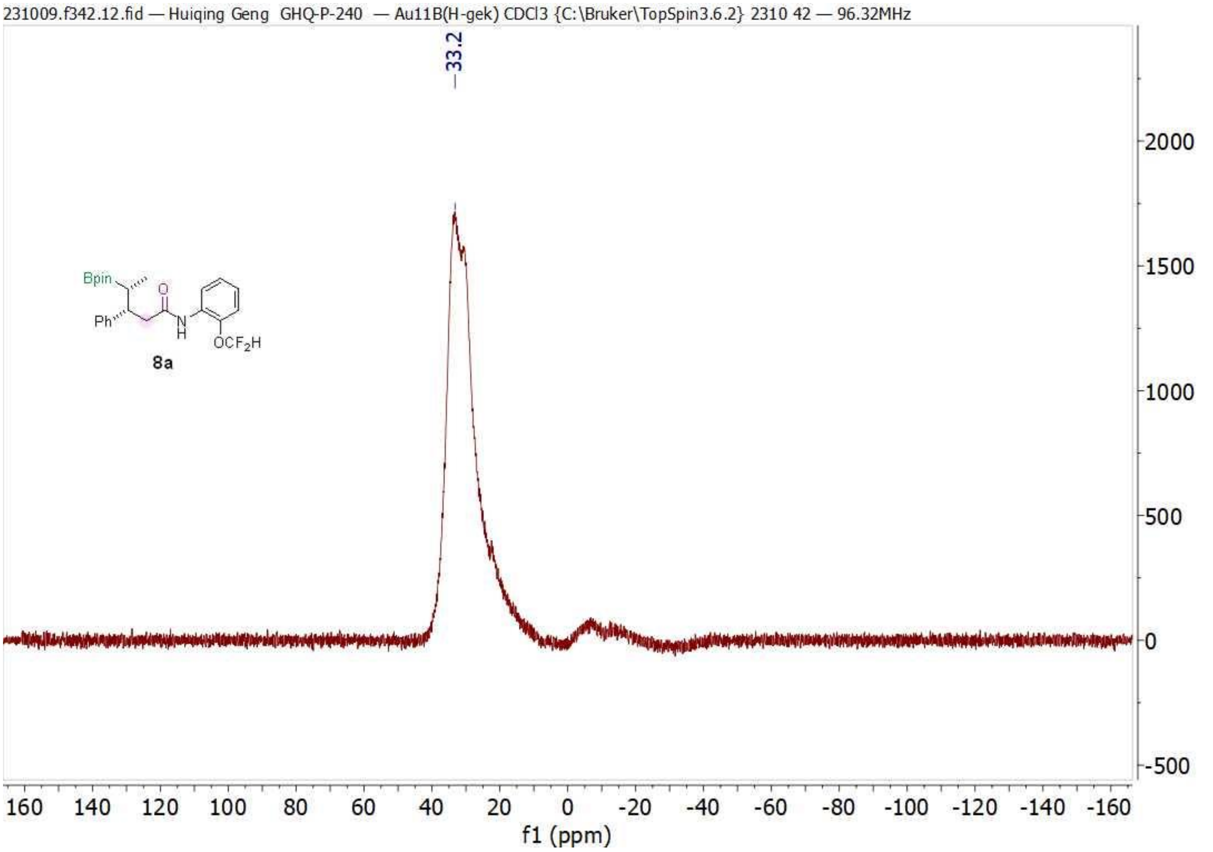
231009.f342.12.fid — Huiqing Geng GHQ-P-240 — Au11B(H-gek) CDCl3 {C:\Bruker\TopSpin3.6.2} 2310 42 — 96.32MHz
33.2
Bpin
Ph
O
N
H
OCF2H
8a
2000
1500
1000
500
0
-500
160 140 120 100 80 60 40 20 0 -20 -40 -60 -80 -100 -120 -140 -160
f1 (ppm)

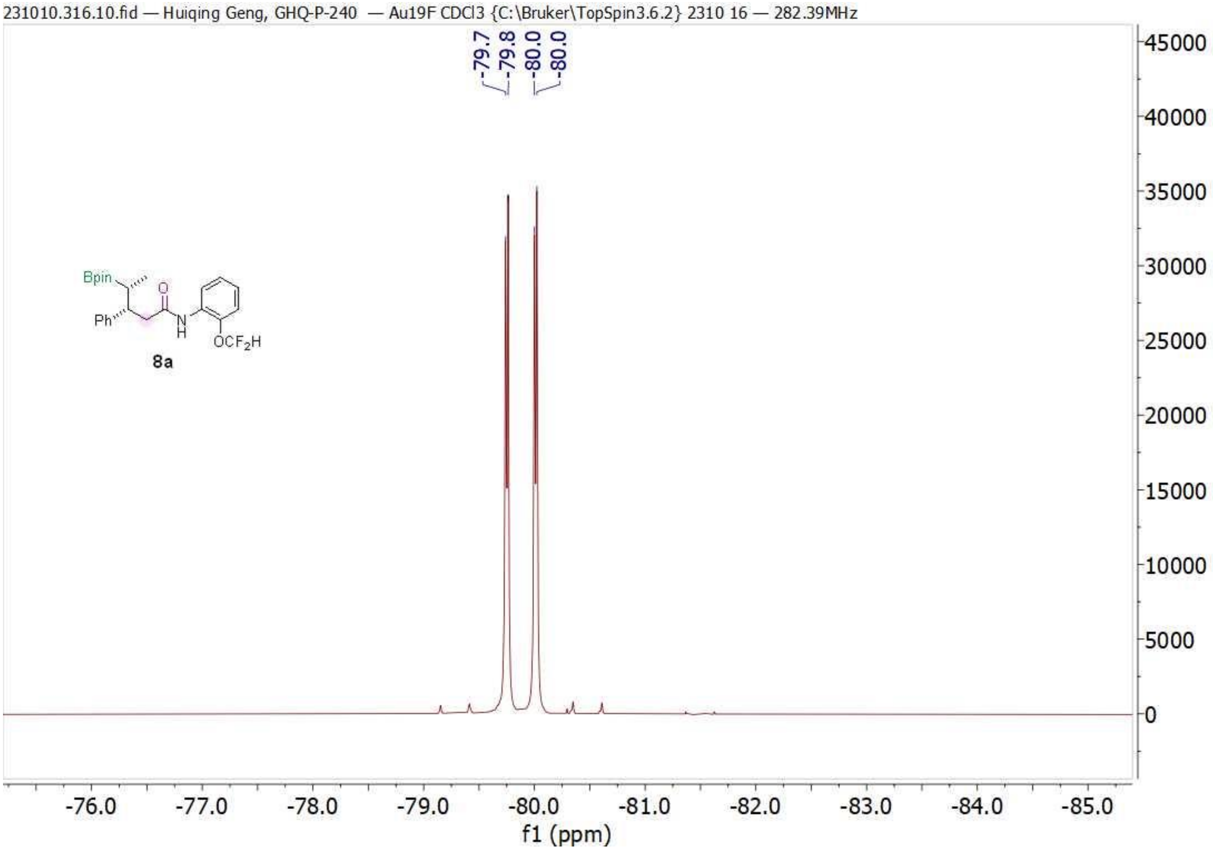
231010.316.10.fid — Huiqing Geng, GHQ-P-240 — Au19F CDCl3 {C:\Bruker\TopSpin3.6.2} 2310 16 — 282.39MHz
-79.7
-79.8
-80.0
-80.0
Bpin
Ph
O
N
H
OCF2H
8a
45000
40000
35000
30000
25000
20000
15000
10000
5000
0
-76.0 -77.0 -78.0 -79.0 -80.0 -81.0 -82.0 -83.0 -84.0 -85.0
f1 (ppm)

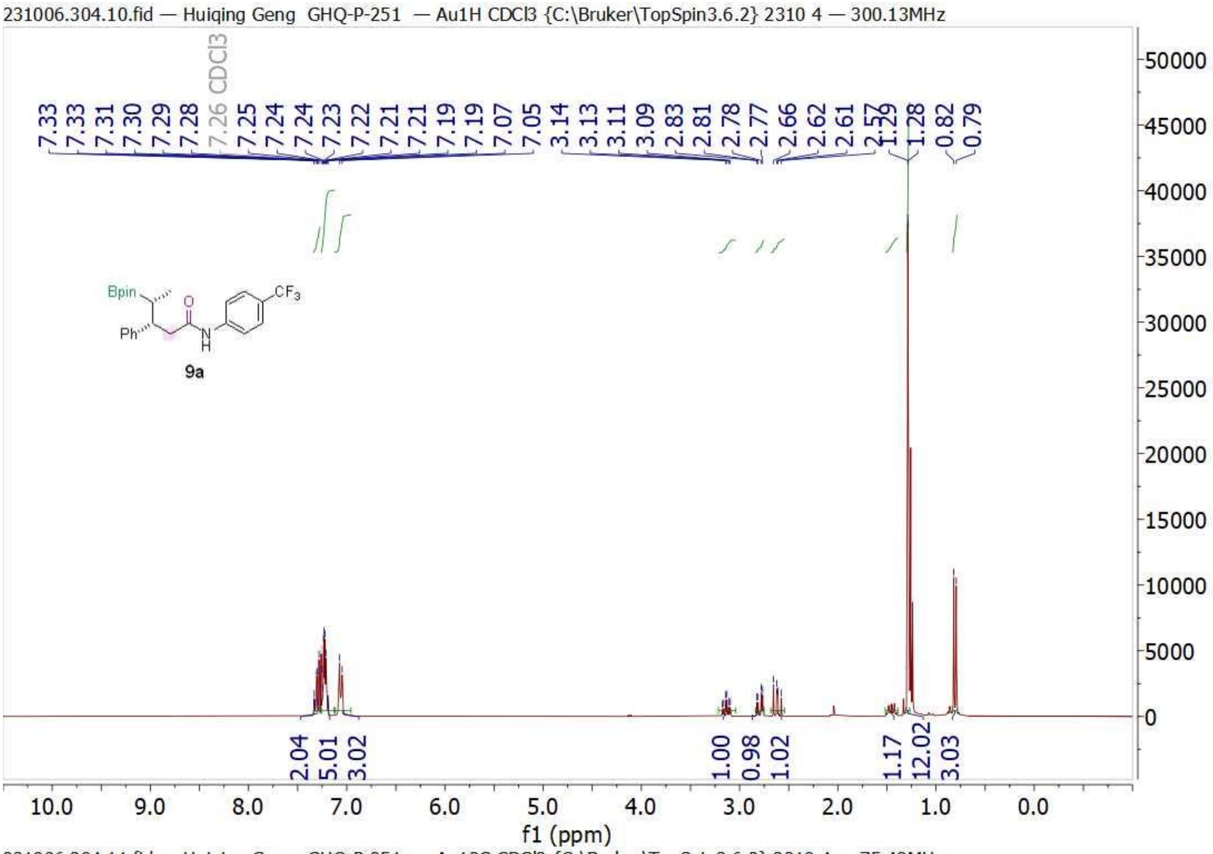
231006.304.10.fid — Huiqing Geng GHQ-P-251 — Au1H CDCl3 {C:\Bruker\TopSpin3.6.2} 2310 4 — 300.13MHz
9a
f1 (ppm)

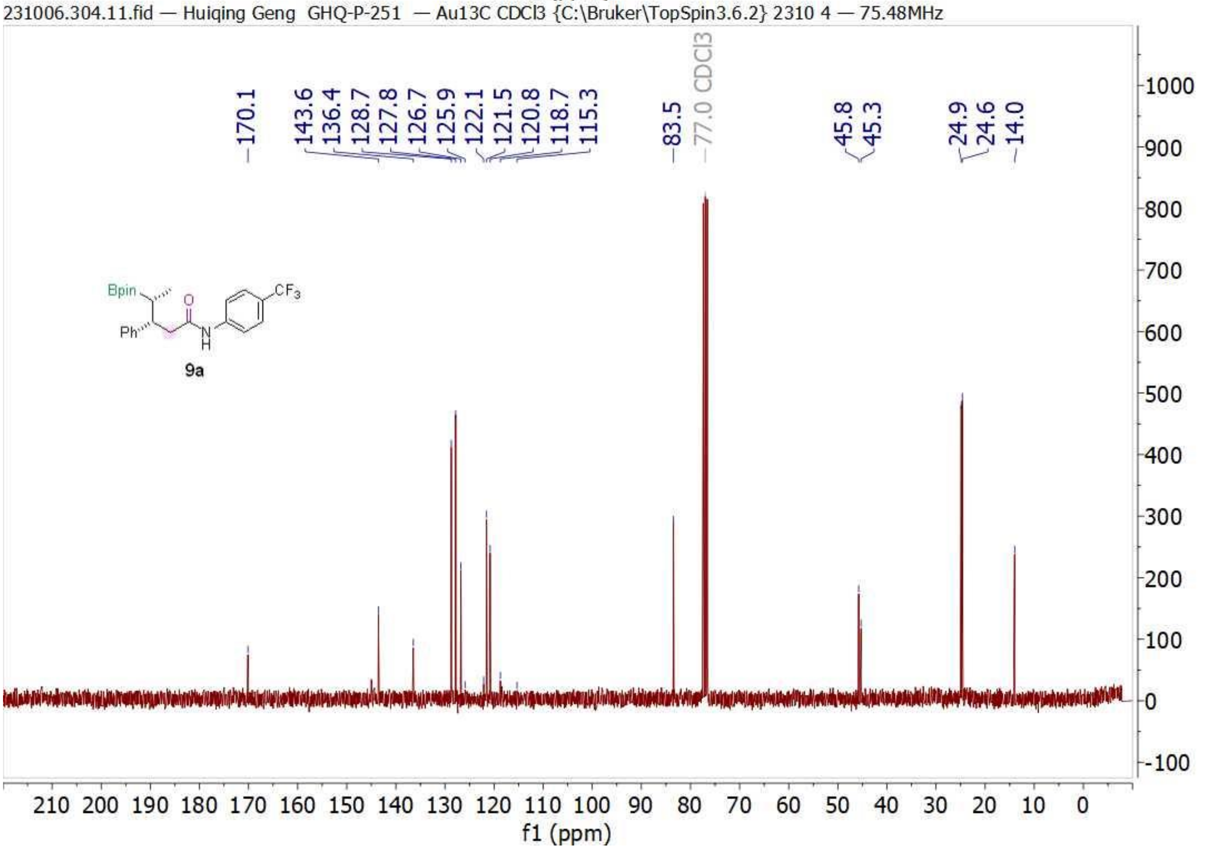
231006.304.11.fid — Huiqing Geng GHQ-P-251 — Au13C CDCl3 {C:\Bruker\TopSpin3.6.2} 2310 4 — 75.48MHz
9a
f1 (ppm)

231006.304.12.fid — Huiqing Geng GHQ-P-251 — Au11B CDCl3 {C:\Bruker\TopSpin3.6.2} 2310 4 — 96.29MHz

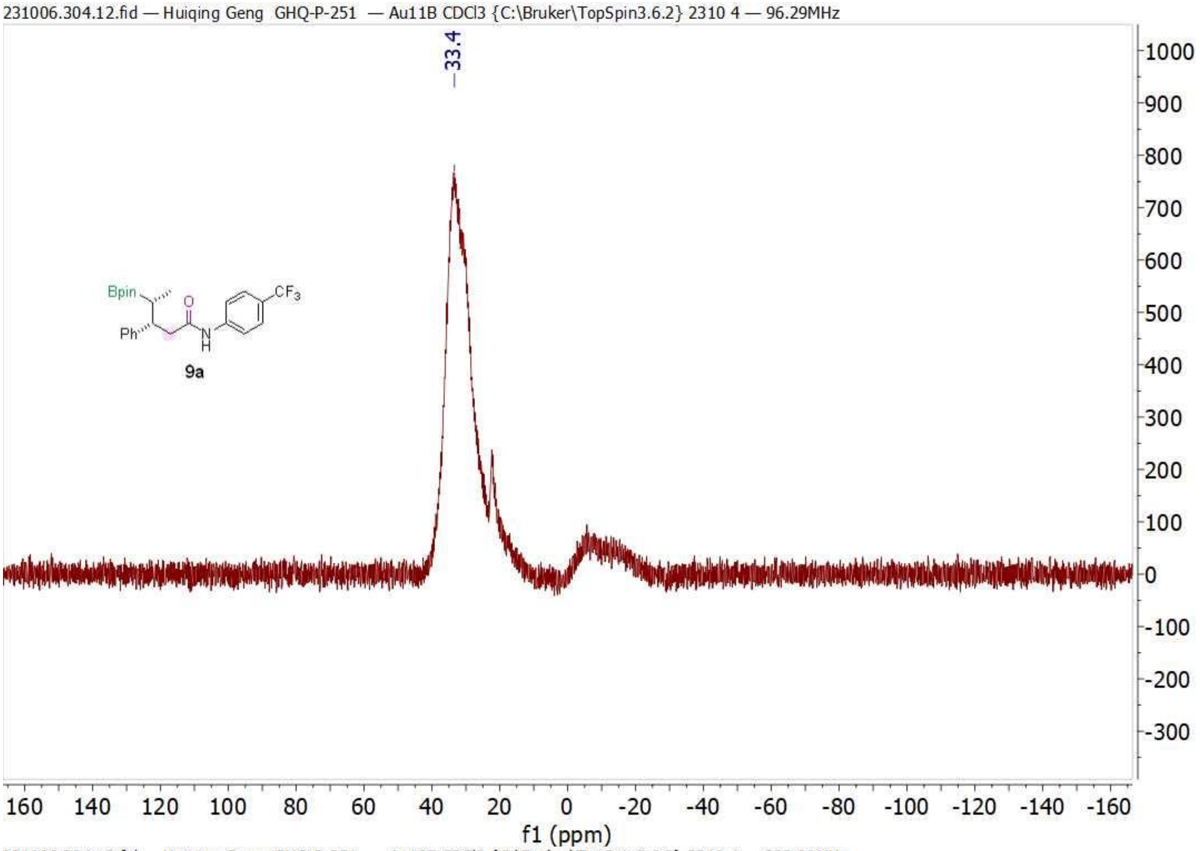

231006.304.13.fid — Huiqing Geng GHQ-P-251 — Au19F CDCl3 {C:\Bruker\TopSpin3.6.2} 2310 4 — 282.39MHz

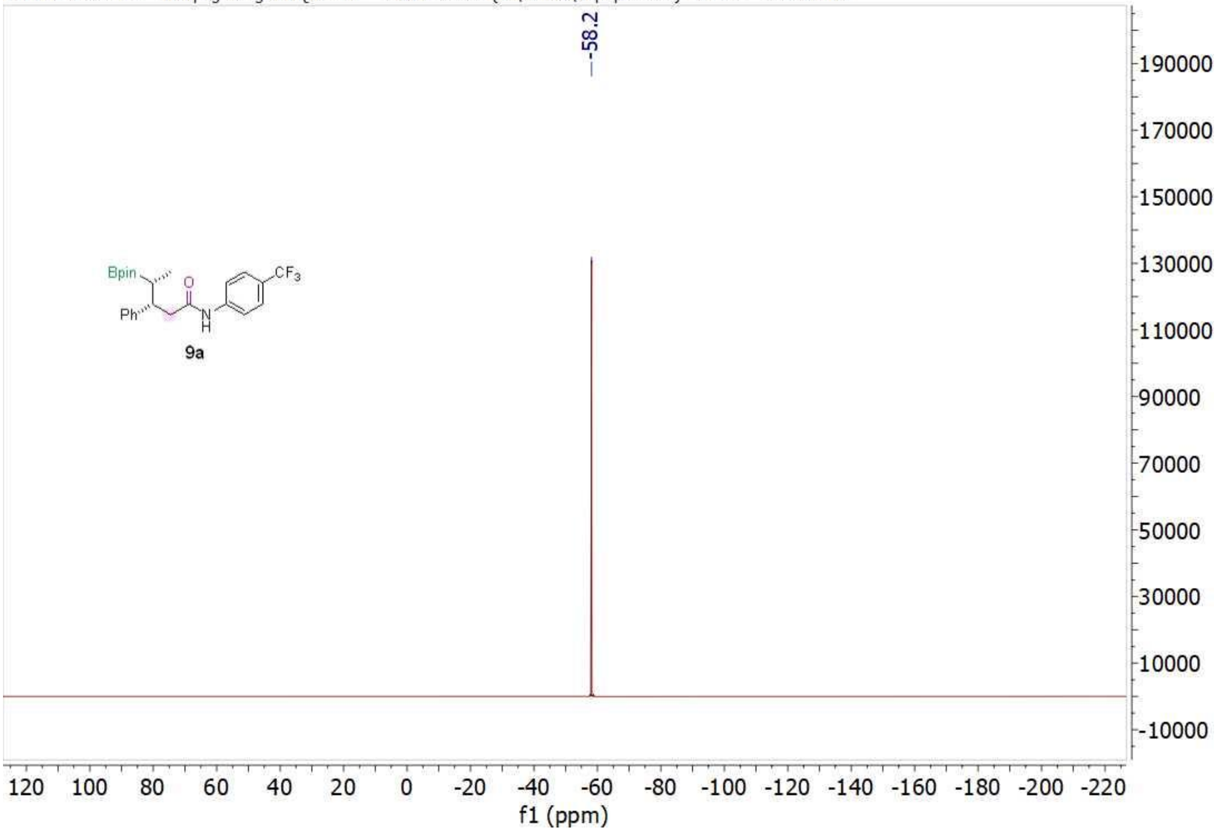

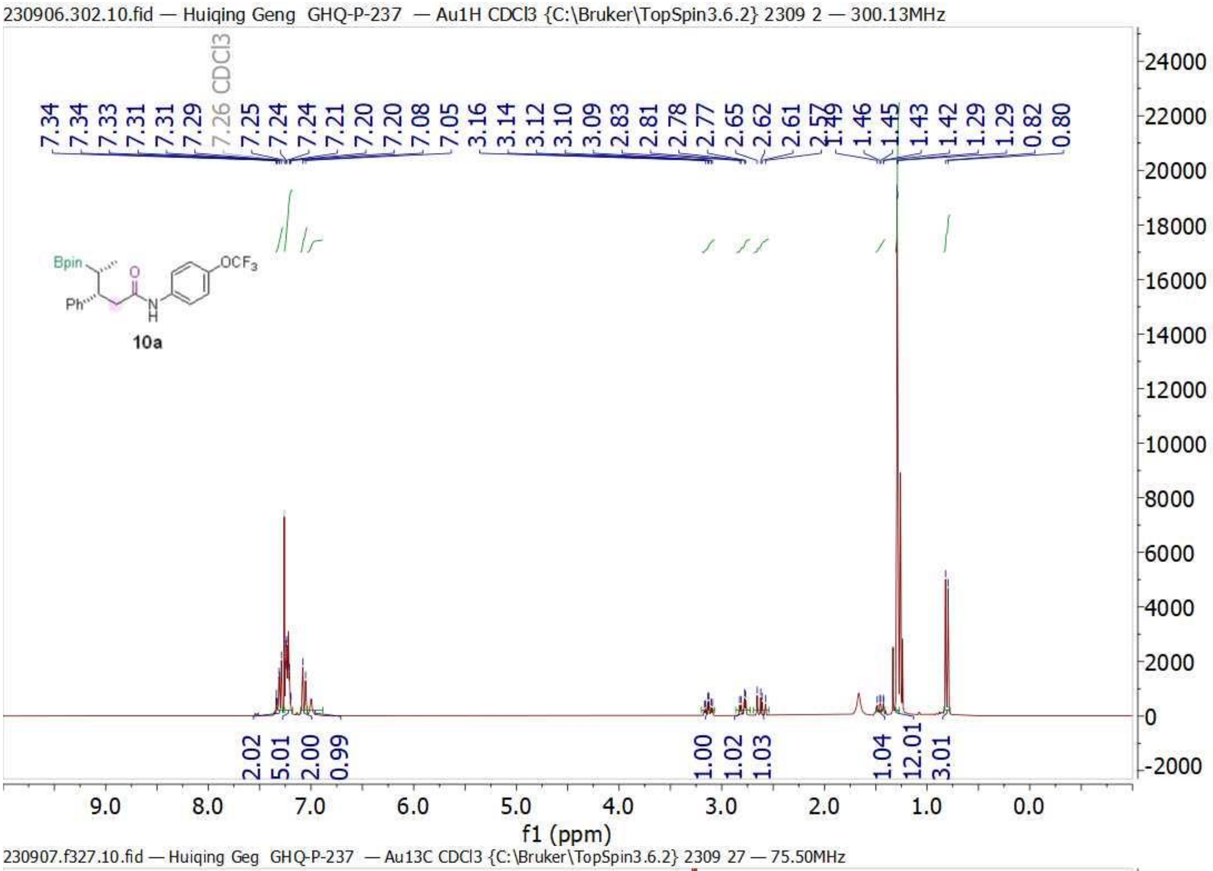
230906.302.10.fid — Huiqing Geng GHQ-P-237 — Au1H CDCl3 {C:\Bruker\TopSpin3.6.2} 2309 2 — 300.13MHz
10a
f1 (ppm)

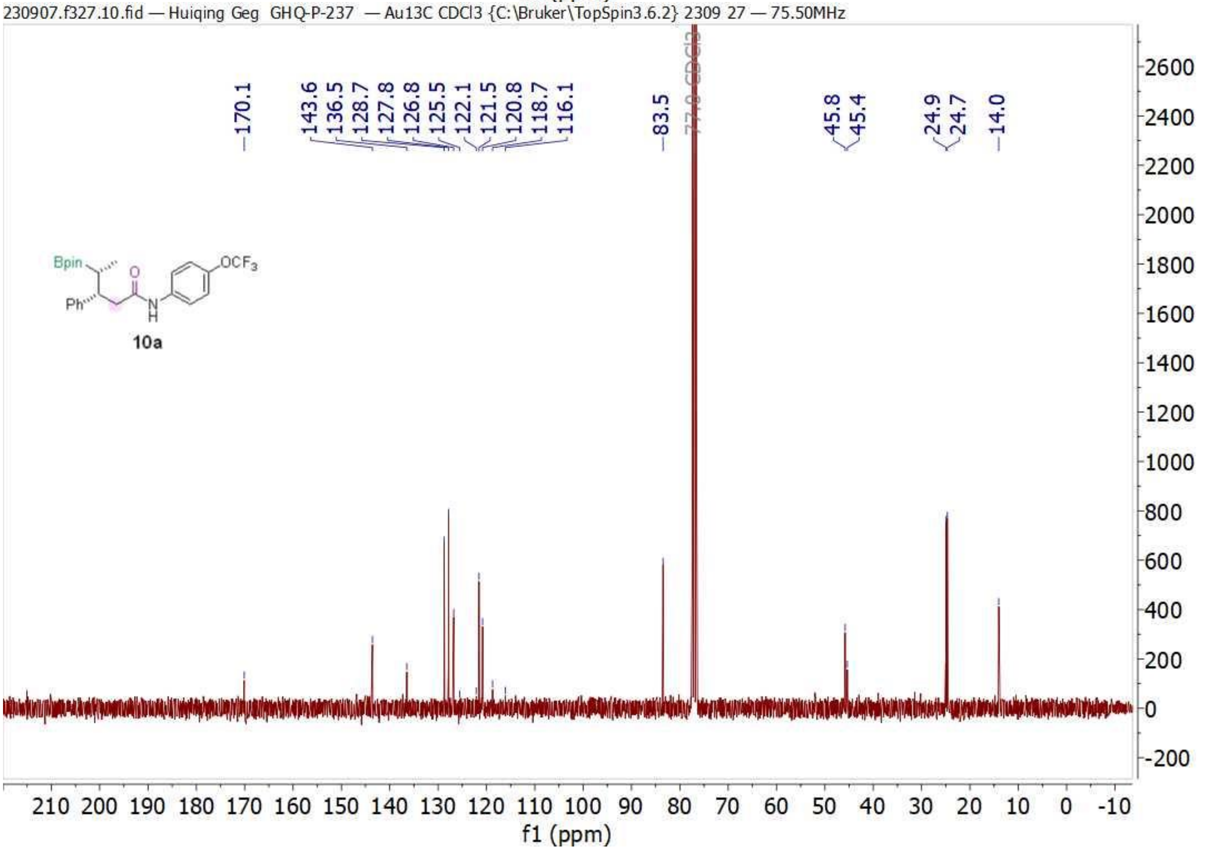
230907.f327.10.fid — Huiqing Geg GHQ-P-237 — Au13C CDCl3 {C:\Bruker\TopSpin3.6.2} 2309 27 — 75.50MHz
10a
f1 (ppm)

230906.302.12.fid — Huiqing Geng GHQ-P-237 — Au11B CDCl3 {C:\Bruker\TopSpin3.6.2} 2309 2 — 96.29MHz

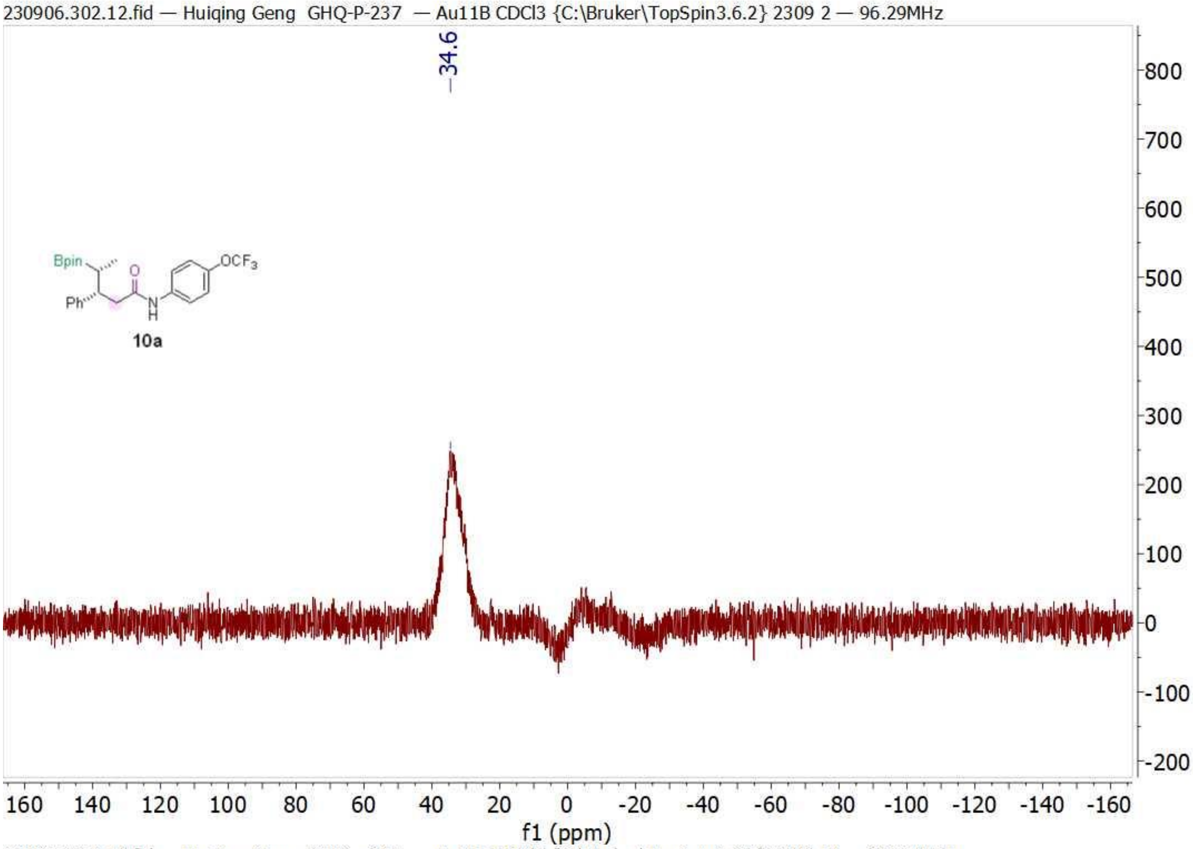

230921.313.10.fid — Huiqing Geng GHQ-p-237 — Au19F CDCl3 {C:\Bruker\TopSpin3.6.2} 2309 13 — 282.39MHz

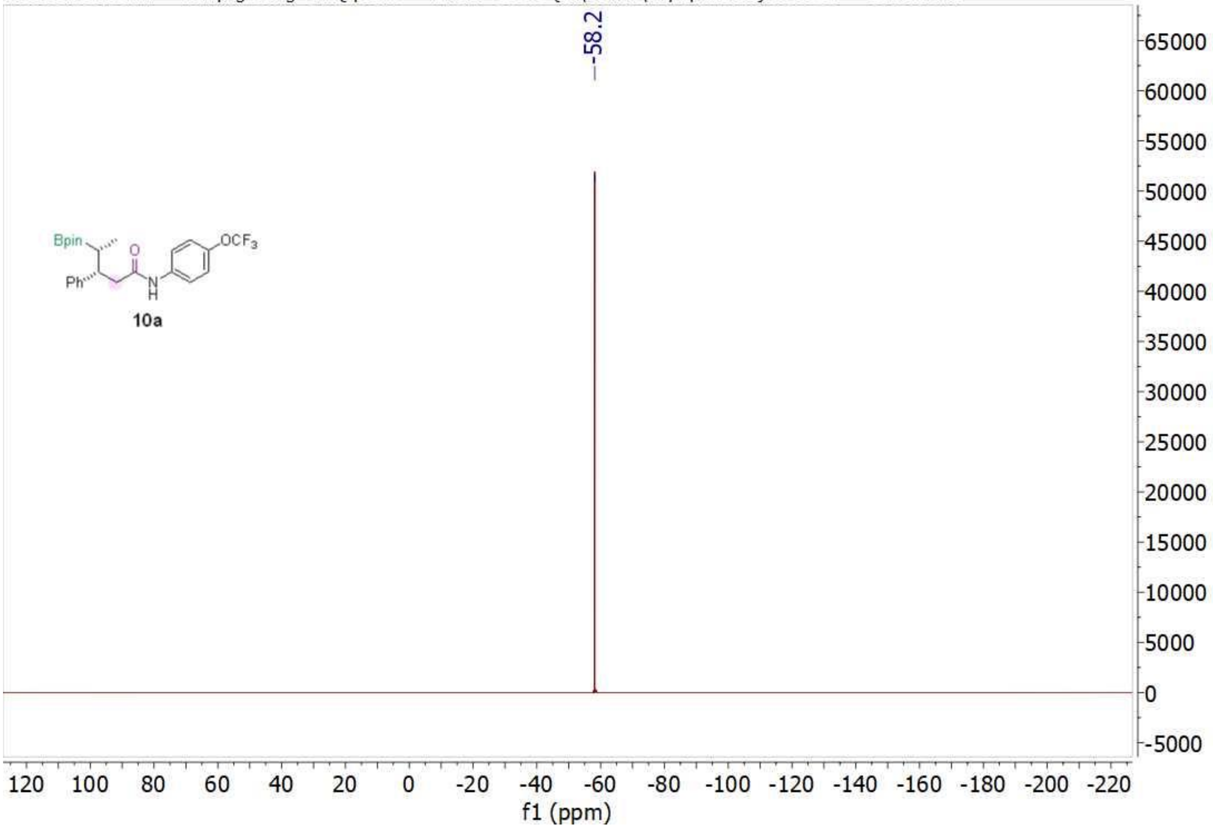

230904.303.10.fid — Huiqing Geng GHQ-P-205 — Au1H CDCl3 {C:\Bruker\TopSpin3.6.2} 2309 3 — 300.13MHz

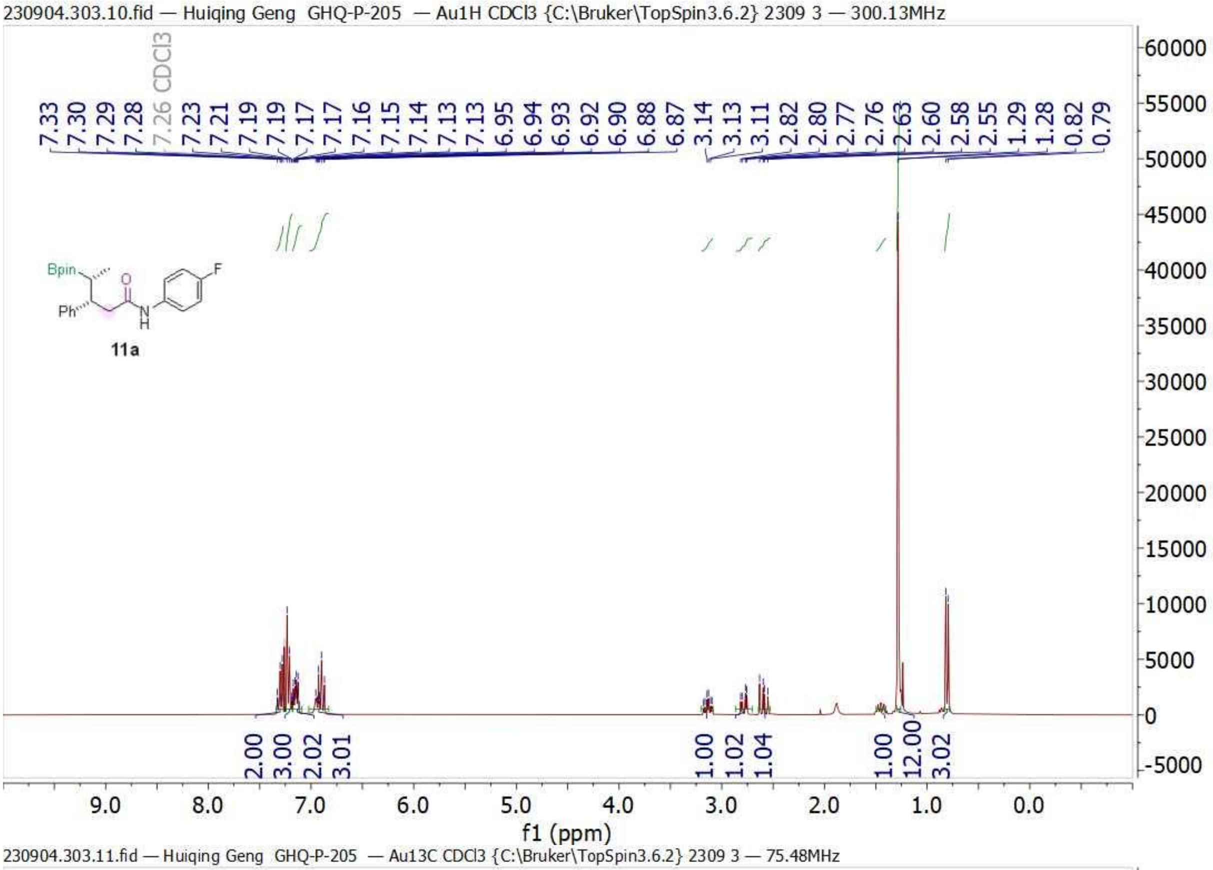

230904.303.11.fid — Huiqing Geng GHQ-P-205 — Au13C CDCl3 {C:\Bruker\TopSpin3.6.2} 2309 3 — 75.48MHz

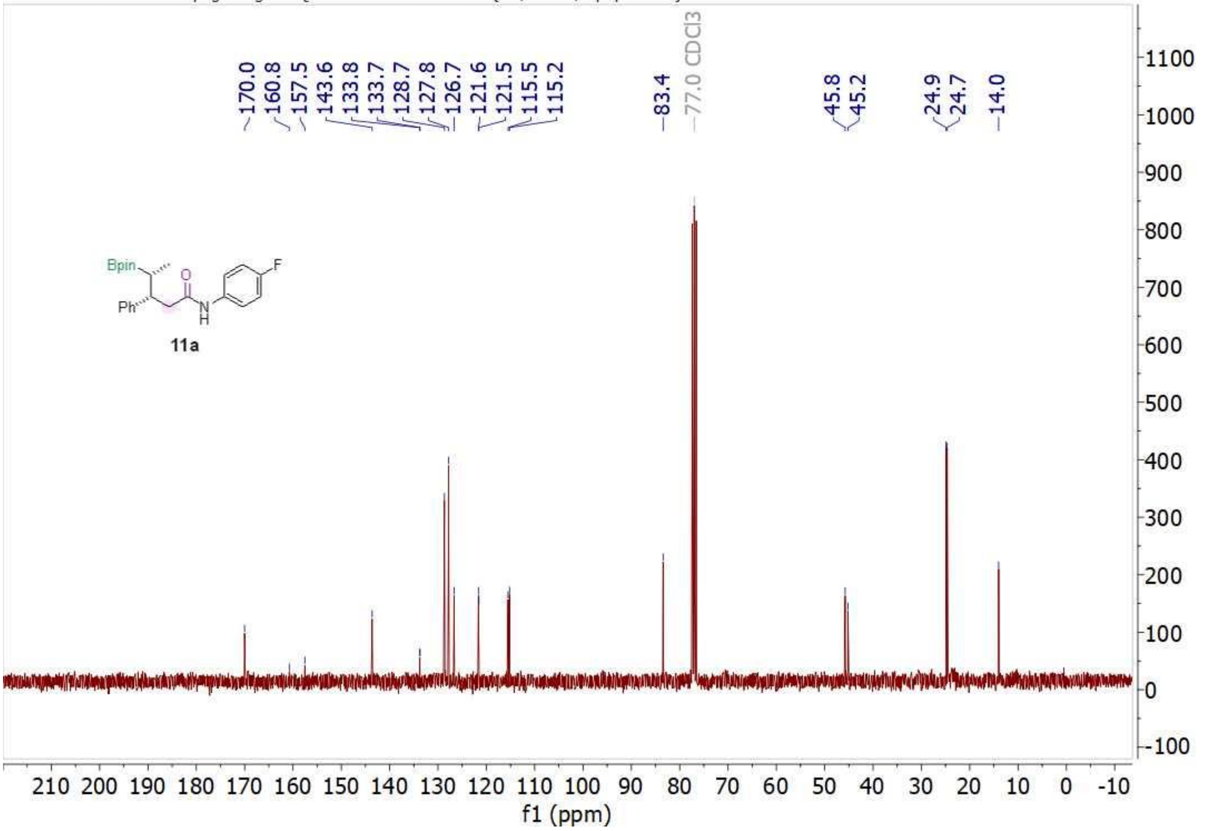

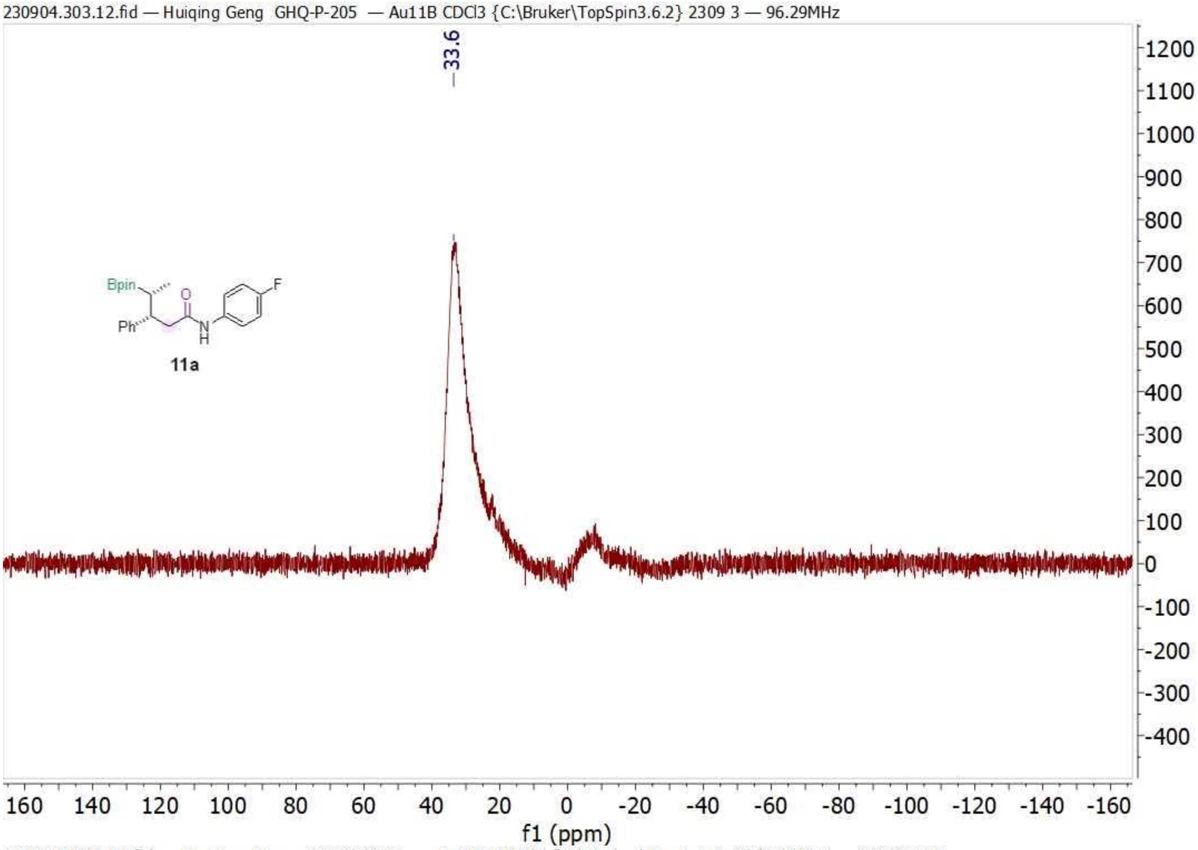
230904.303.12.fid — Huiqing Geng GHQ-P-205 — Au11B CDCl3 {C:\Bruker\TopSpin3.6.2} 2309 3 — 96.29MHz
33.6
Bpin
Ph
O
N
H
F
11a
f1 (ppm)

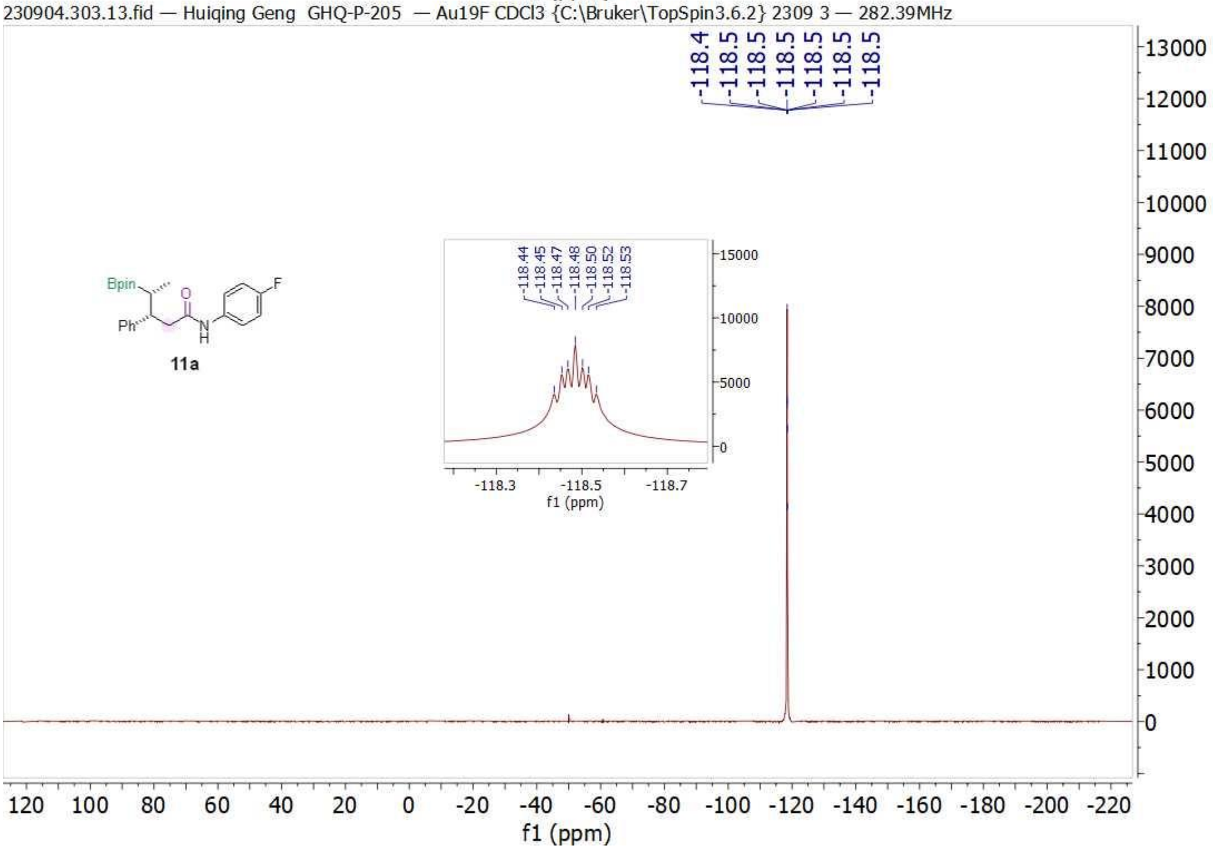
230904.303.13.fid — Huiqing Geng GHQ-P-205 — Au19F CDCl3 {C:\Bruker\TopSpin3.6.2} 2309 3 — 282.39MHz
-118.4
-118.5
-118.5
-118.5
-118.5
-118.5
-118.5
Bpin
Ph
O
N
H
F
11a
-118.44
-118.45
-118.47
-118.48
-118.50
-118.52
-118.53
f1 (ppm)

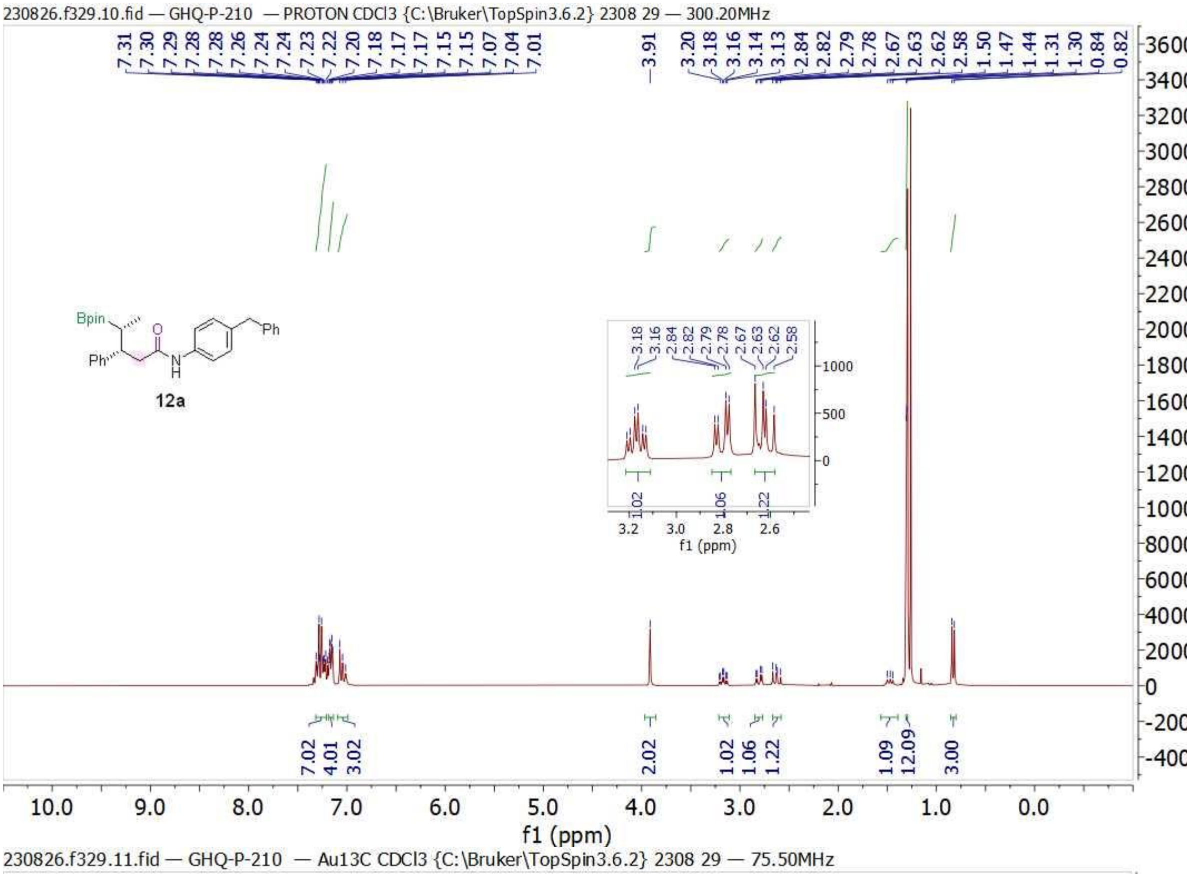
230826.f329.10.fid — GHQ-P-210 — PROTON CDCl3 {C:\Bruker\TopSpin3.6.2} 2308 29 — 300.20MHz
7.31 7.30 7.29 7.28 7.28 7.26 7.24 7.24 7.23 7.22 7.20 7.18 7.17 7.17 7.15 7.15 7.07 7.04 7.01
3.91
3.20 3.18 3.16 3.14 3.13 2.84 2.82 2.79 2.78 2.67 2.63 2.62 2.58 1.50 1.47 1.44 1.31 1.30 0.84 0.82
Bpin
Ph
Ph
H
N
O
12a
7.02 4.01 3.02 2.02 1.02 1.06 1.22 1.09 12.09 3.00
f1 (ppm)

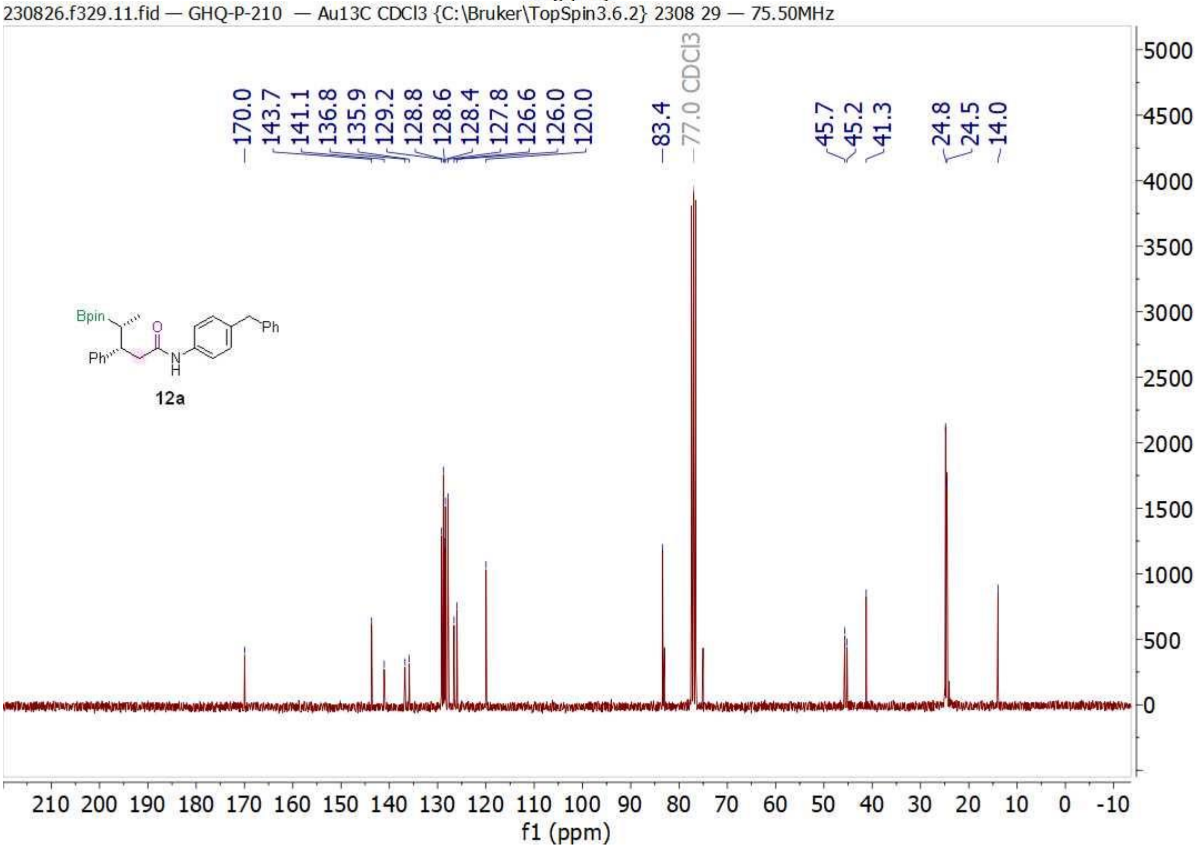
230826.f329.11.fid — GHQ-P-210 — Au13C CDCl3 {C:\Bruker\TopSpin3.6.2} 2308 29 — 75.50MHz
170.0 143.7 141.1 136.8 135.9 129.2 128.8 128.6 128.4 127.8 126.6 126.0 120.0
83.4
77.0 CDCl3
45.7 45.2 41.3
24.8 24.5 14.0
Bpin
Ph
Ph
H
N
O
12a
f1 (ppm)

230901.410.12.fid — Huiqing Geng GHQ-P-210 — Au11B CDCl3 {C:\Bruker\TopSpin3.6.2} 2309 10 — 128.38MHz

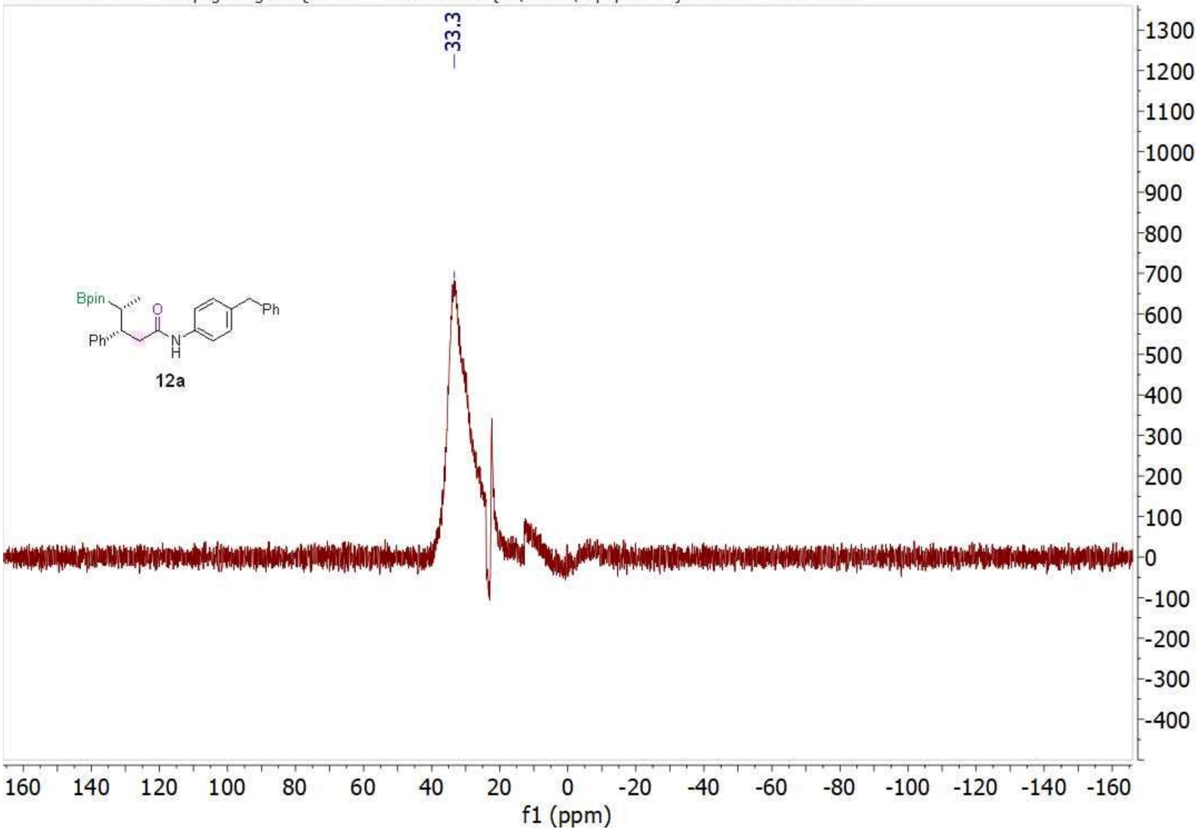

230908.302.10.fid — Huiqing Geng GHQ-P-241 — Au1H CDCl3 {C:\Bruker\TopSpin3.6.2} 2309 2 — 300.13MHz

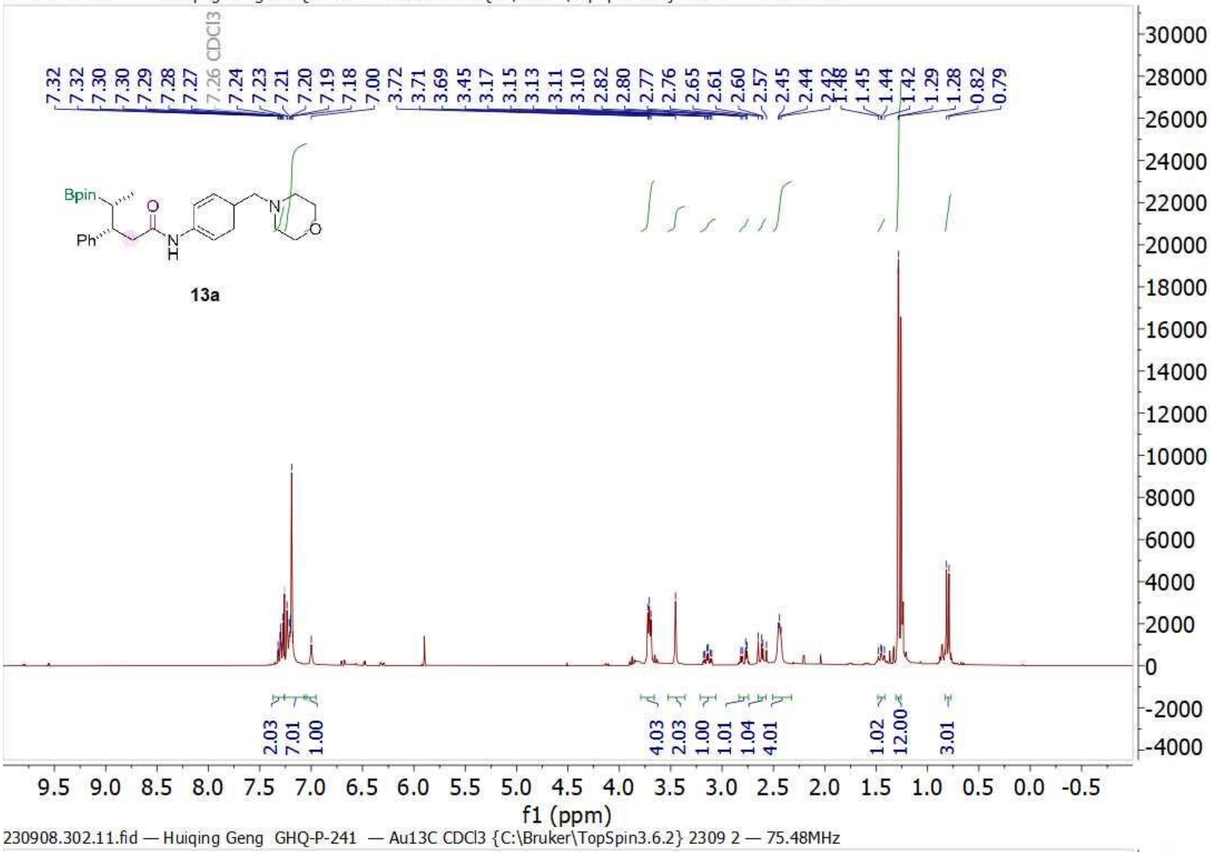

13a

230908.302.11.fid — Huiqing Geng GHQ-P-241 — Au13C CDCl3 {C:\Bruker\TopSpin3.6.2} 2309 2 — 75.48MHz

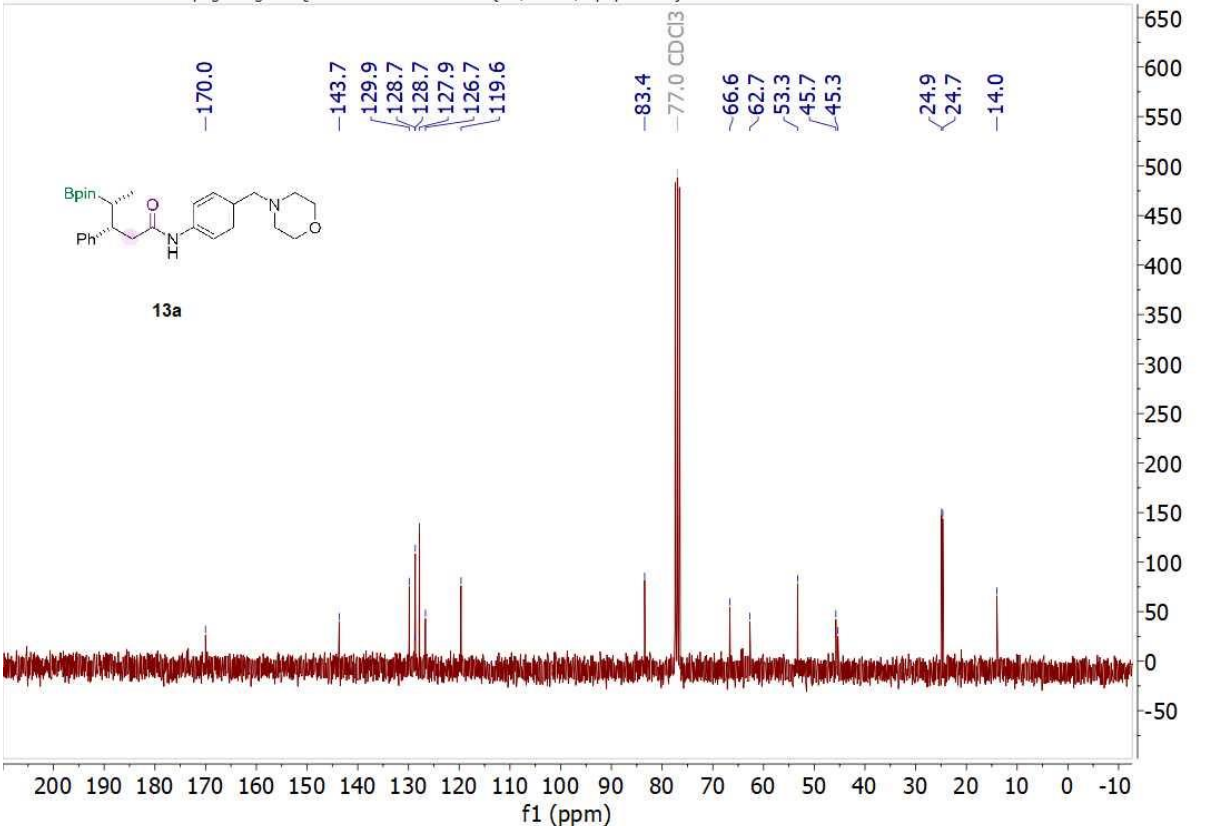

13a

230908.302.12.fid — Huiqing Geng GHQ-P-241 — Au11B CDCl3 {C:\Bruker\TopSpin3.6.2} 2309 2 — 96.29MHz

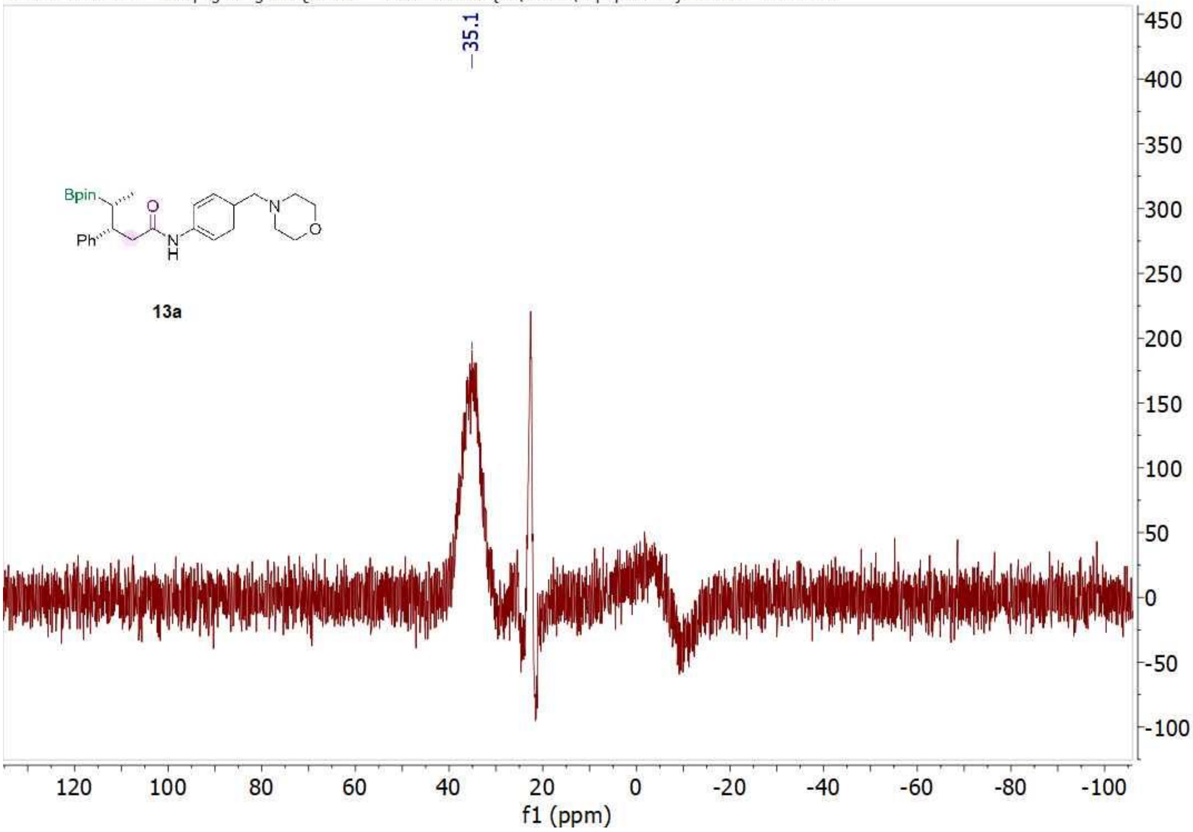

231026.f308.10.fid — Huiqing Geng GHQ-P-278 — Au1H CDCl3 {C:\Bruker\TopSpin3.6.2} 2310 8 — 300.20MHz

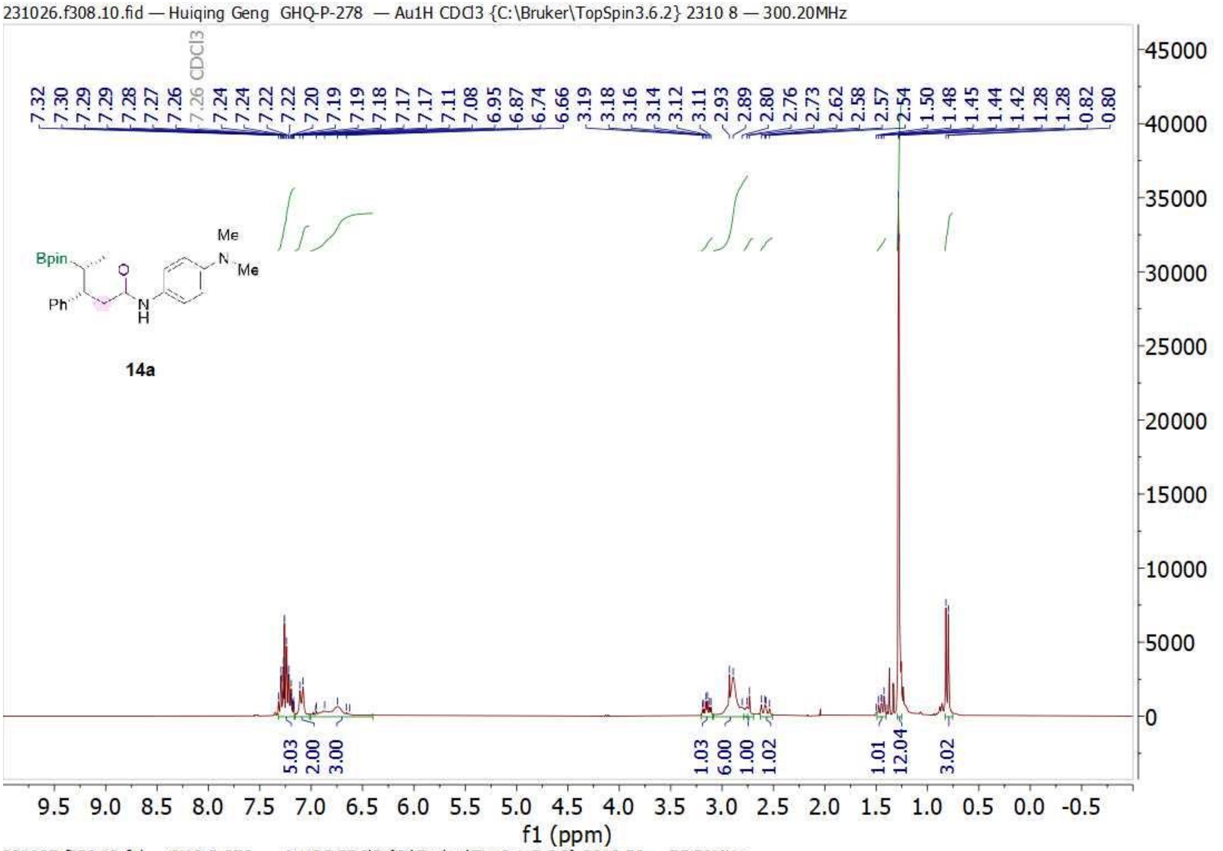

231027.f358.12.fid — GHQ-P-278 — Au13C CDCl3 {C:\Bruker\TopSpin3.6.2} 2310 58 — 75.50MHz

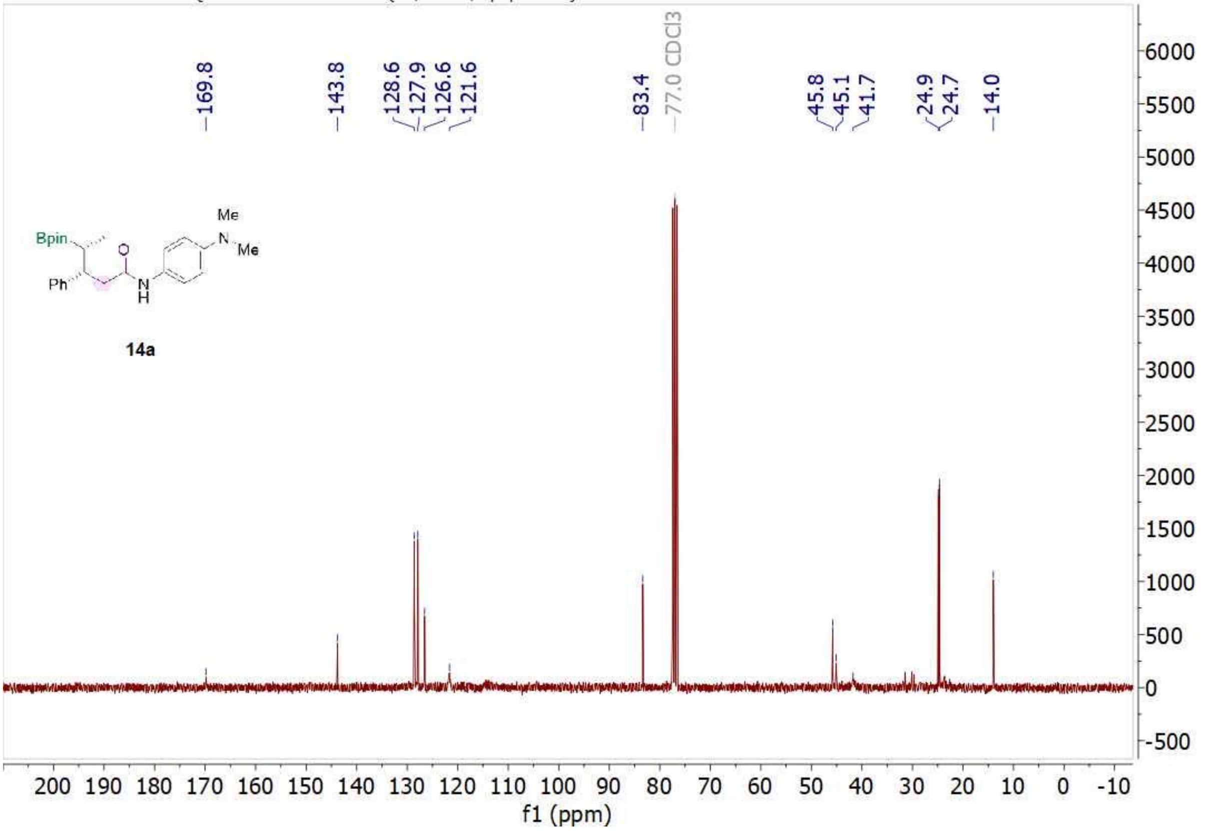

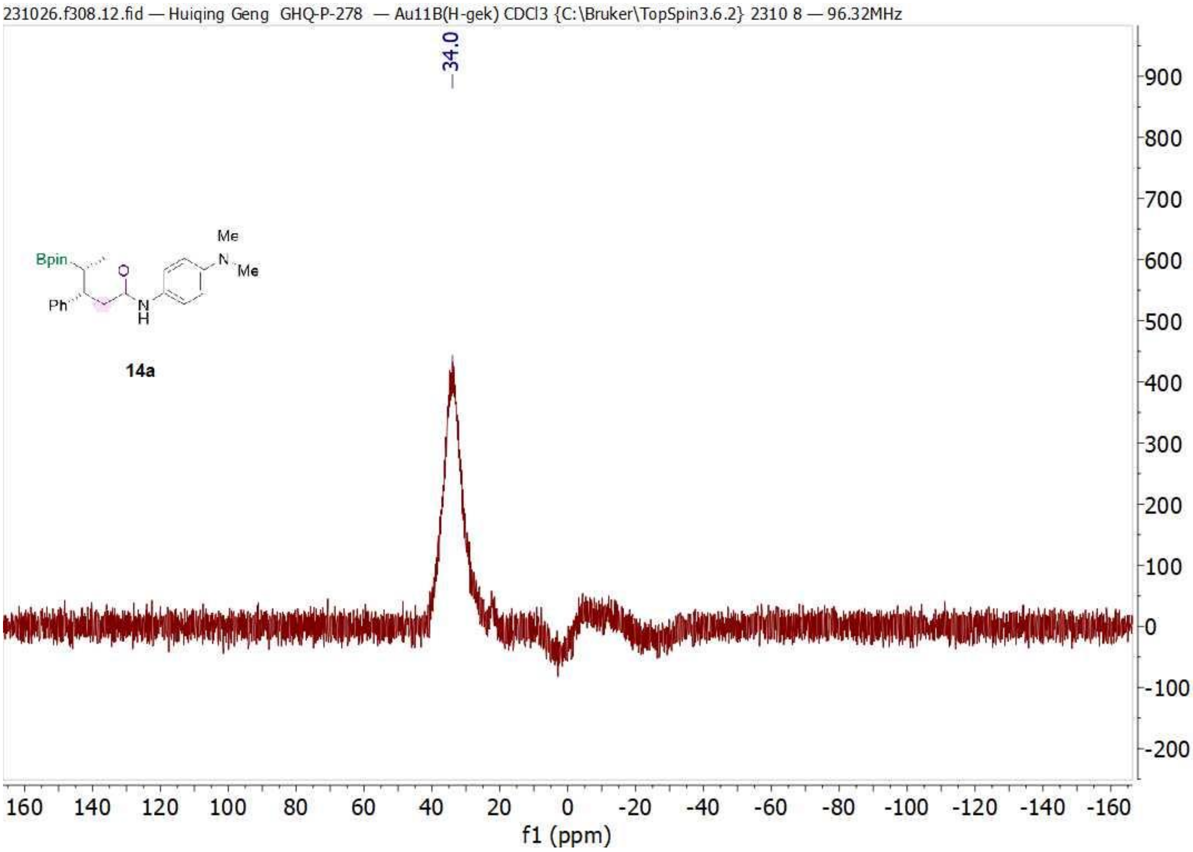

231026.f308.12.fid — Huiqing Geng GHQ-P-278 — Au11B(H-gek) CDCl3 {C:\Bruker\TopSpin3.6.2} 2310 8 — 96.32MHz
34.0
Bpin
Ph
O
N
H
N
Me
Me
14a
f1 (ppm)

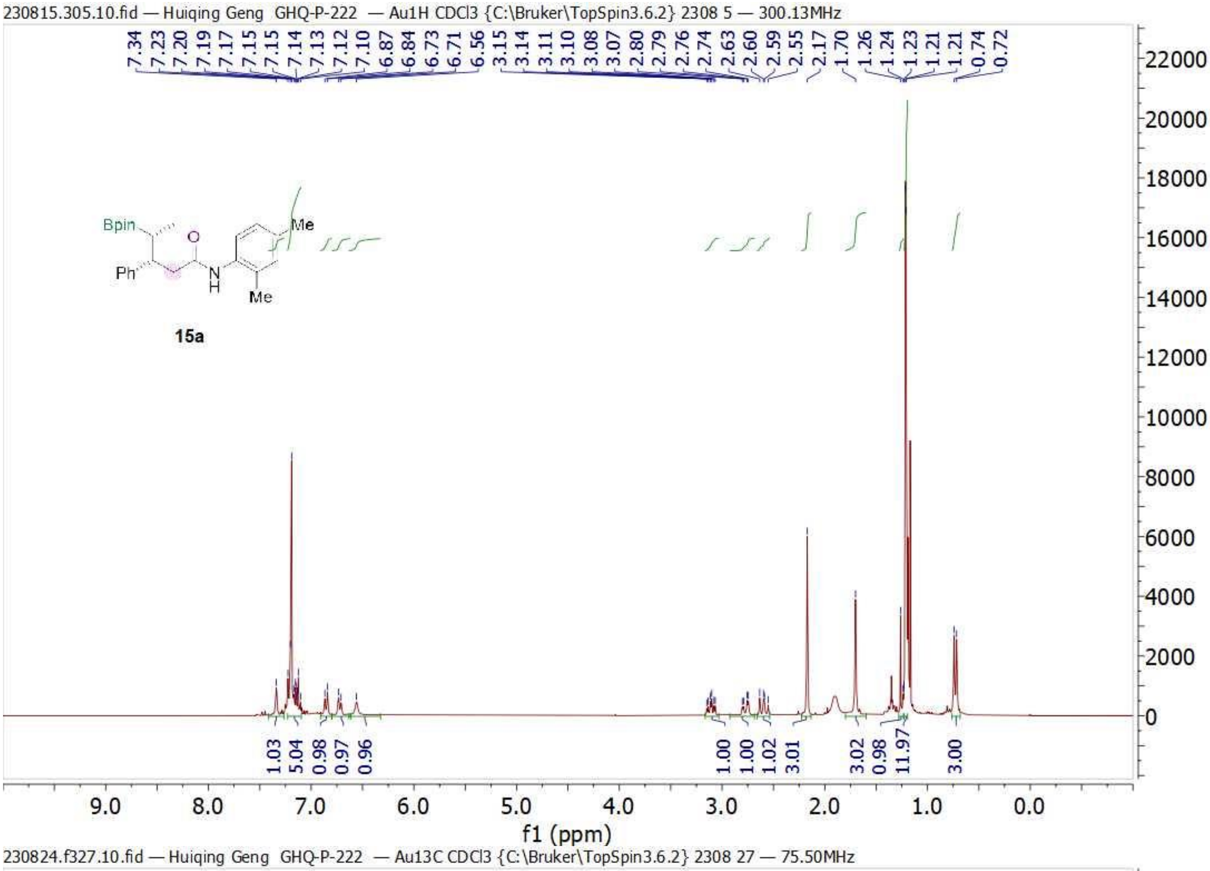
230815.305.10.fid — Huiqing Geng GHQ-P-222 — Au1H CDCl3 {C:\Bruker\TopSpin3.6.2} 2308 5 — 300.13MHz
7.34 7.23 7.20 7.19 7.17 7.15 7.14 7.13 7.12 7.10 6.87 6.84 6.73 6.71 6.56 3.15 3.14 3.11 3.10 3.08 3.07 2.80 2.79 2.76 2.74 2.63 2.60 2.59 2.55 2.17 1.70 1.26 1.24 1.23 1.21 1.21 0.74 0.72
Bpin
O
Ph
N
H
Me
Me
15a
1.03 5.04 0.98 0.97 0.96
1.00 1.00 1.02 3.01 3.02 0.98 11.97 3.00
f1 (ppm)

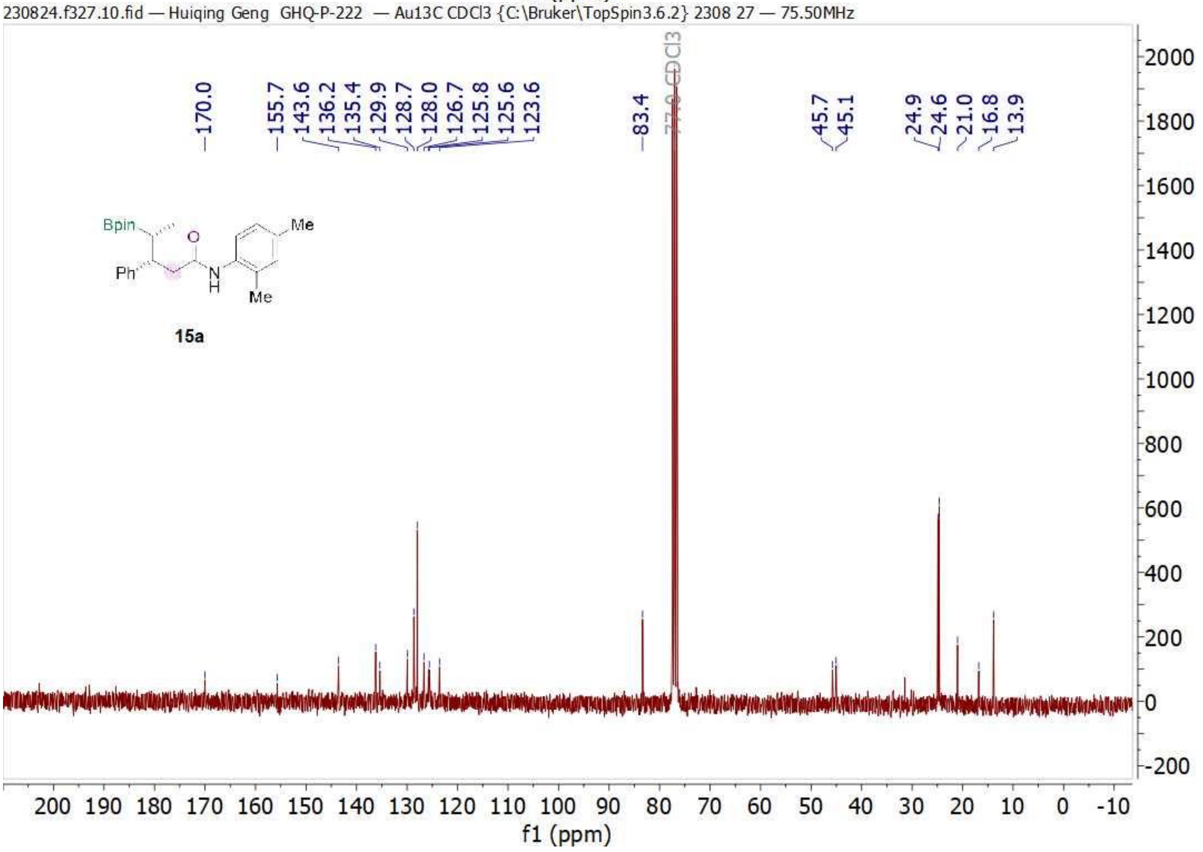
230824.f327.10.fid — Huiqing Geng GHQ-P-222 — Au13C CDCl3 {C:\Bruker\TopSpin3.6.2} 2308 27 — 75.50MHz
170.0 155.7 143.6 136.2 135.4 129.9 128.7 128.0 126.7 125.8 125.6 123.6 83.4 77.0 CDCl3 45.7 45.1 24.9 24.6 21.0 16.8 13.9
Bpin
O
Ph
N
H
Me
Me
15a
f1 (ppm)

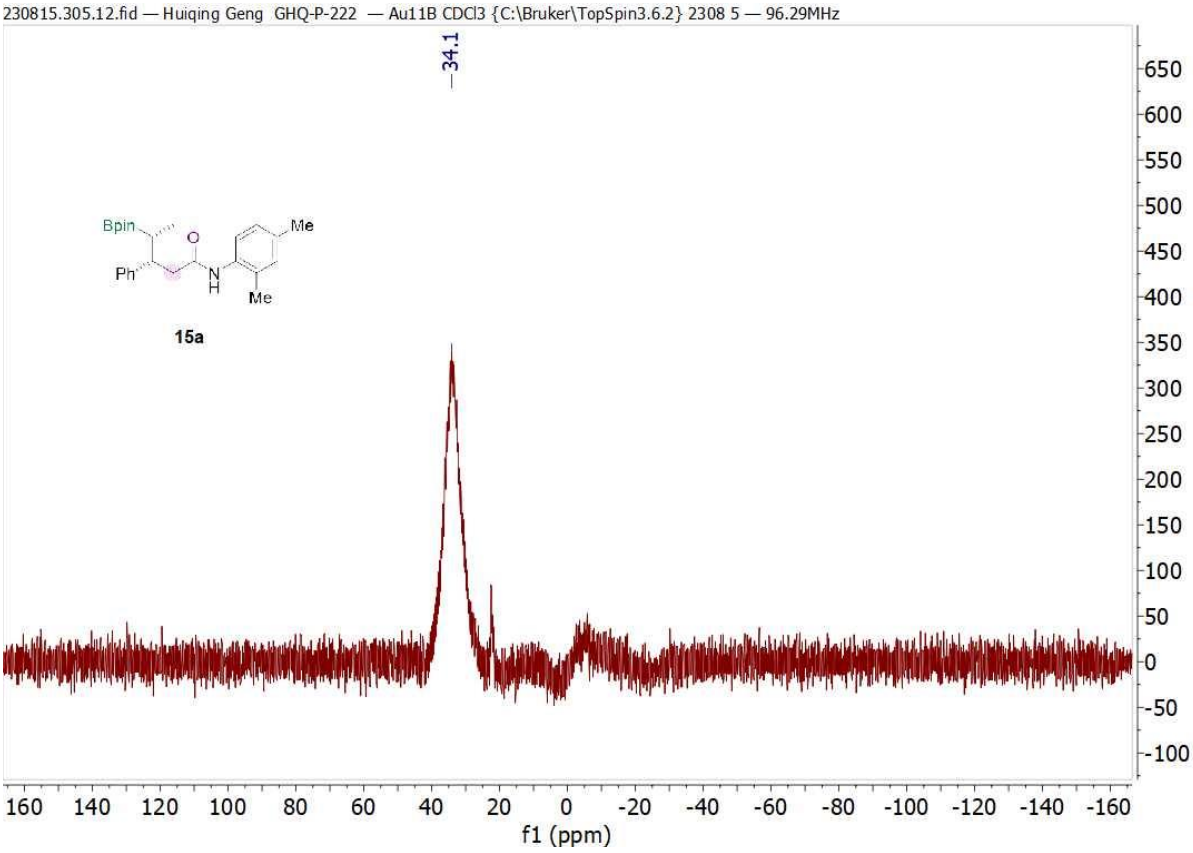
230815.305.12.fid — Huiqing Geng GHQ-P-222 — Au11B CDCl3 {C:\Bruker\TopSpin3.6.2} 2308 5 — 96.29MHz
34.1
Bpin
O
Me
Ph
N
H
Me
15a
f1 (ppm)

230925.305.10.fid — Huiqing Geng GHQ-P-234 — Au1H CDCl3 {C:\Bruker\TopSpin3.6.2} 2309 5 — 300.13MHz

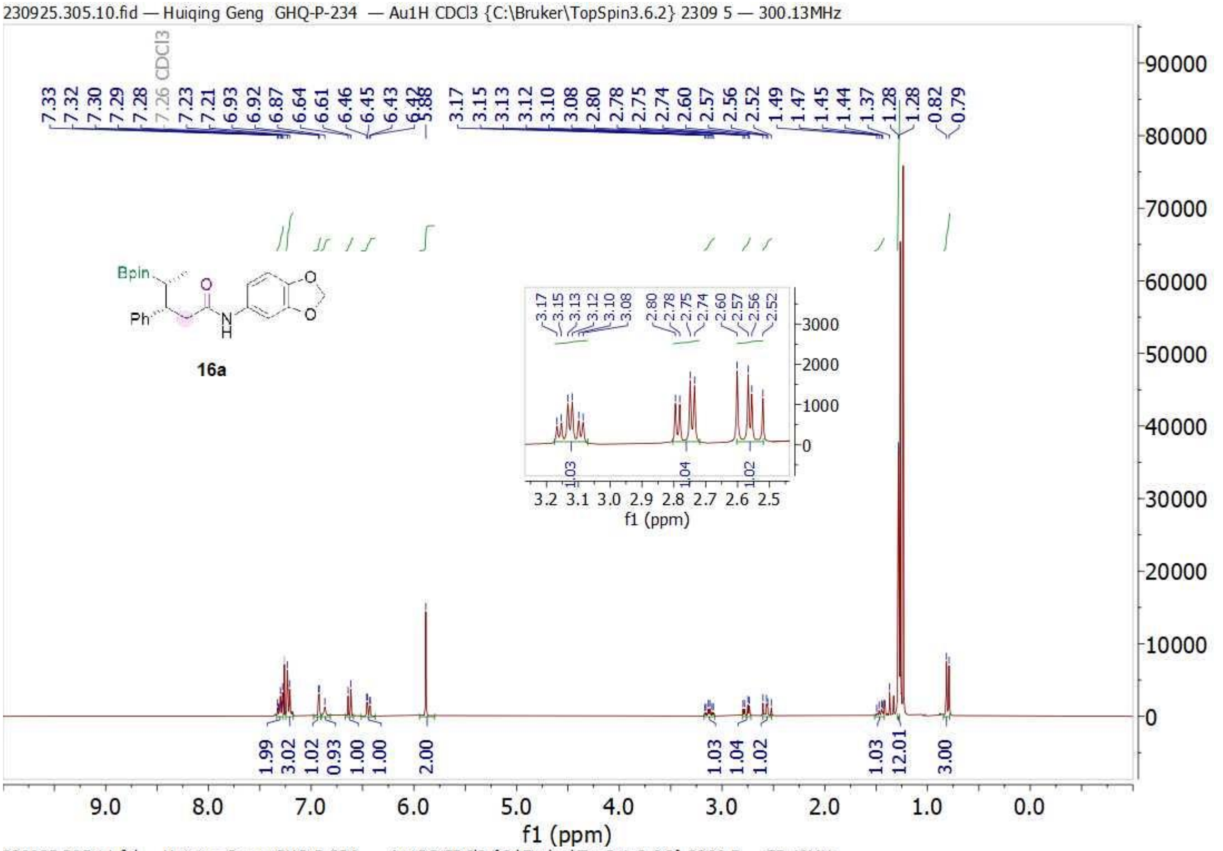

230925.305.11.fid — Huiqing Geng GHQ-P-234 — Au13C CDCl3 {C:\Bruker\TopSpin3.6.2} 2309 5 — 75.48MHz

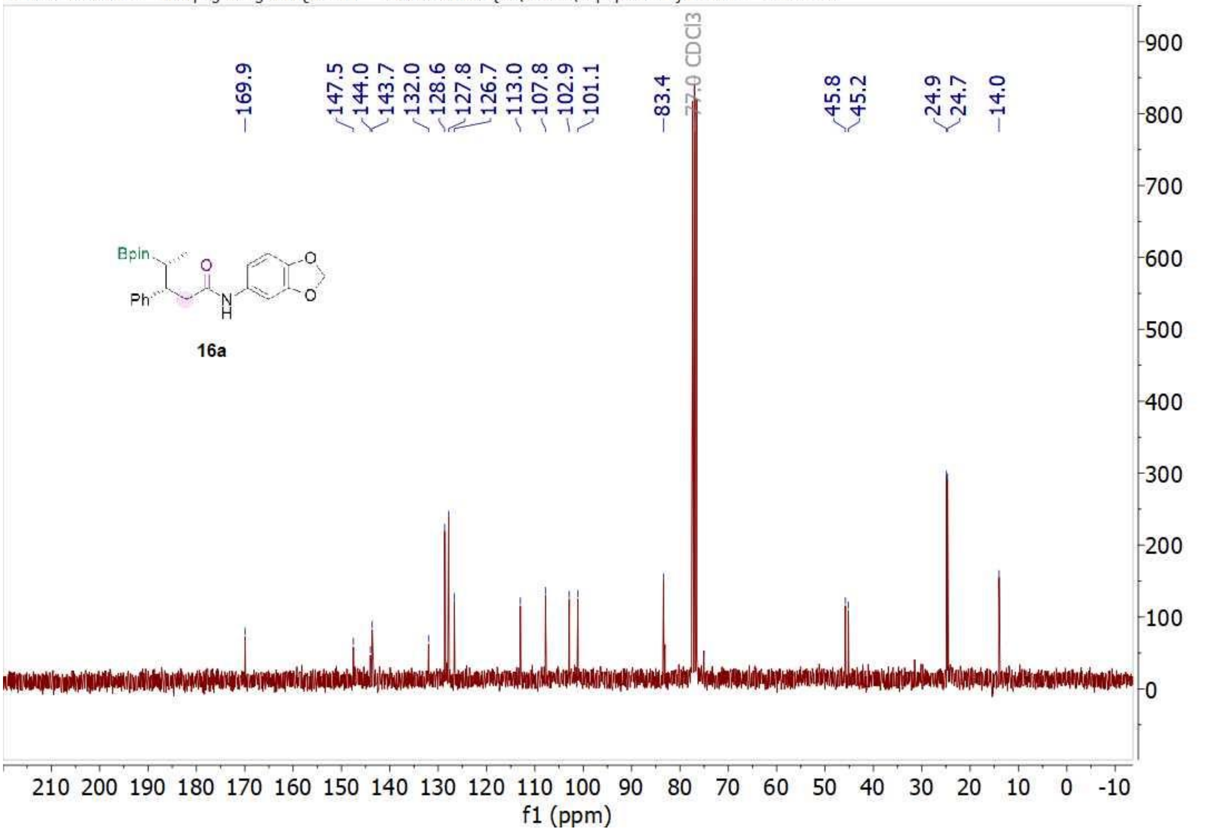

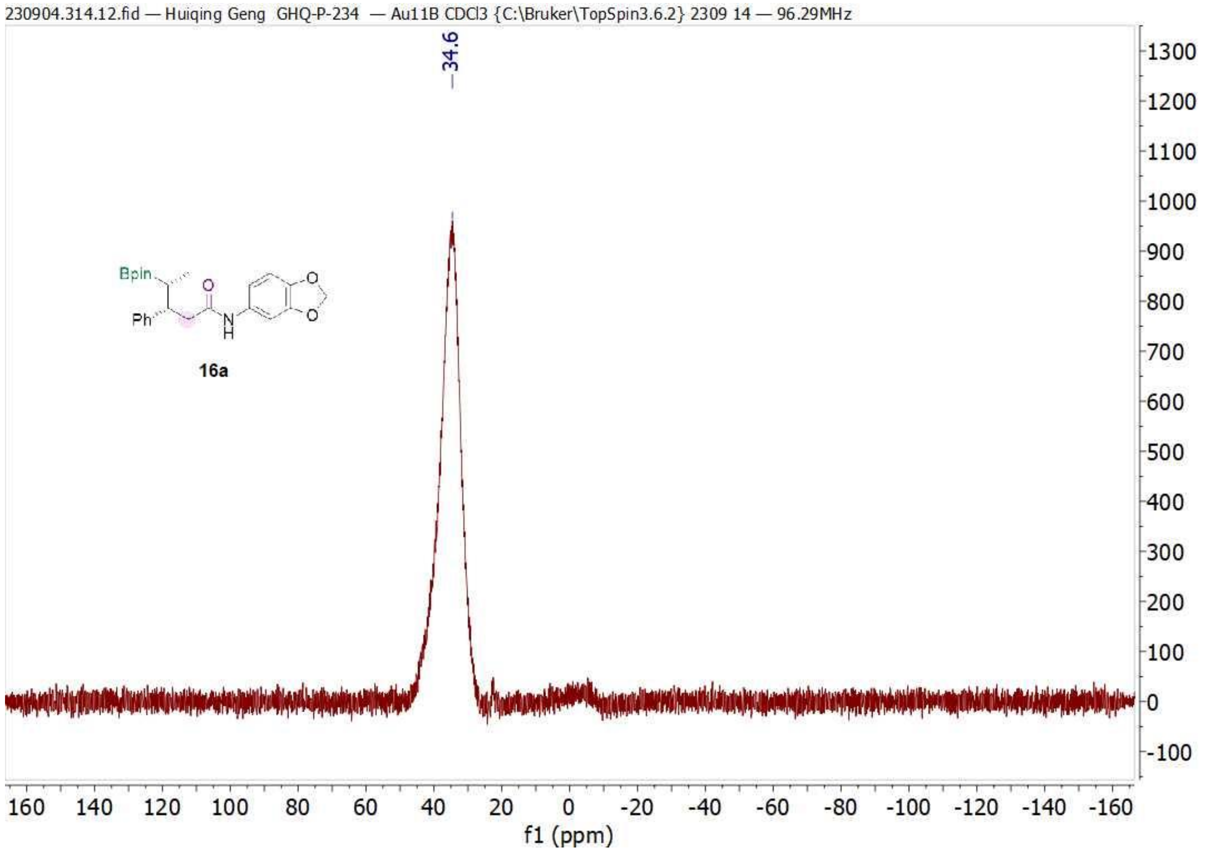
230904.314.12.fid — Huiqing Geng GHQ-P-234 — Au11B CDCl3 {C:\Bruker\TopSpin3.6.2} 2309 14 — 96.29MHz
—34.6
Bpin
Ph
O
N
H
O
O
16a
f1 (ppm)

230901.409.10.fid — Huiqing Geng GHQ-P-209 — Au1H CDCl3 {C:\Bruker\TopSpin3.6.2} 2309 9 — 400.13MHz

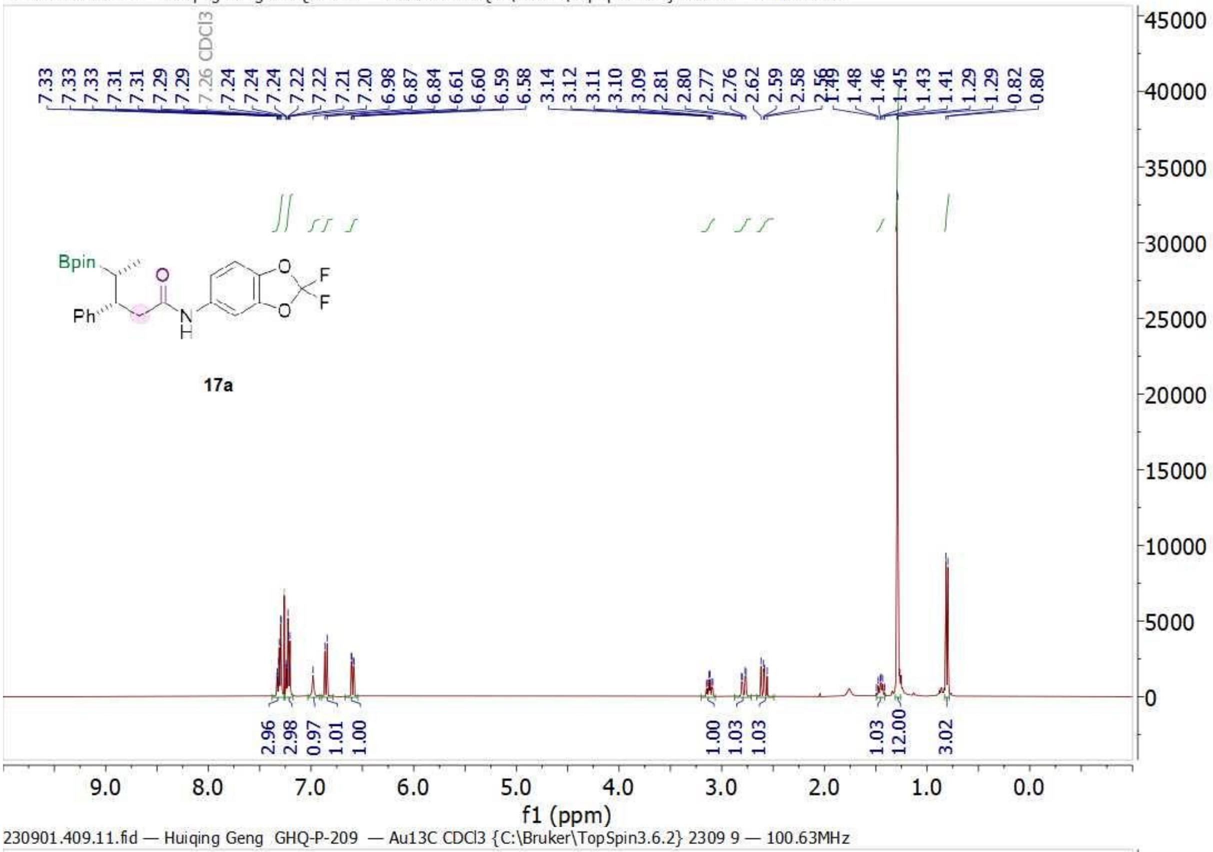

230901.409.11.fid — Huiqing Geng GHQ-P-209 — Au13C CDCl3 {C:\Bruker\TopSpin3.6.2} 2309 9 — 100.63MHz

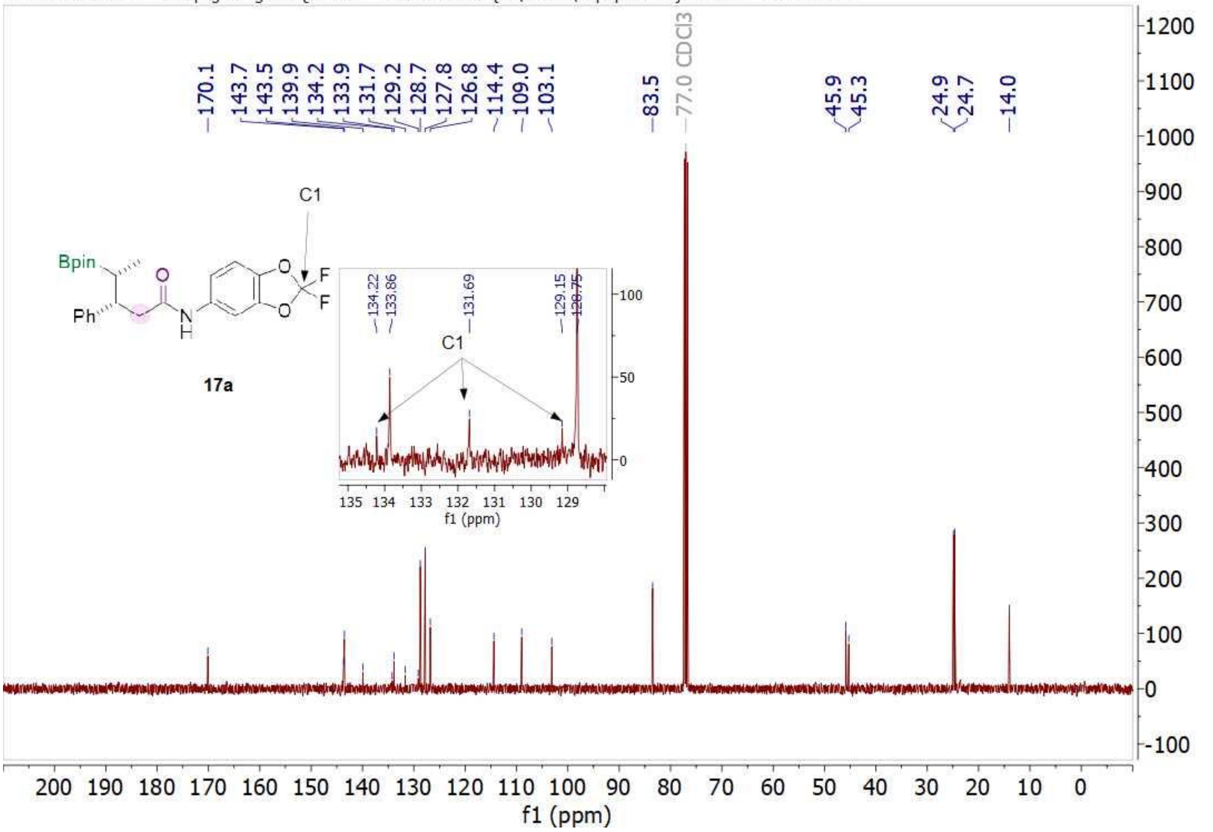

230901.409.12.fid — Huiqing Geng GHQ-P-209 — Au11B CDCl3 {C:\Bruker\TopSpin3.6.2} 2309 9 — 128.38MHz

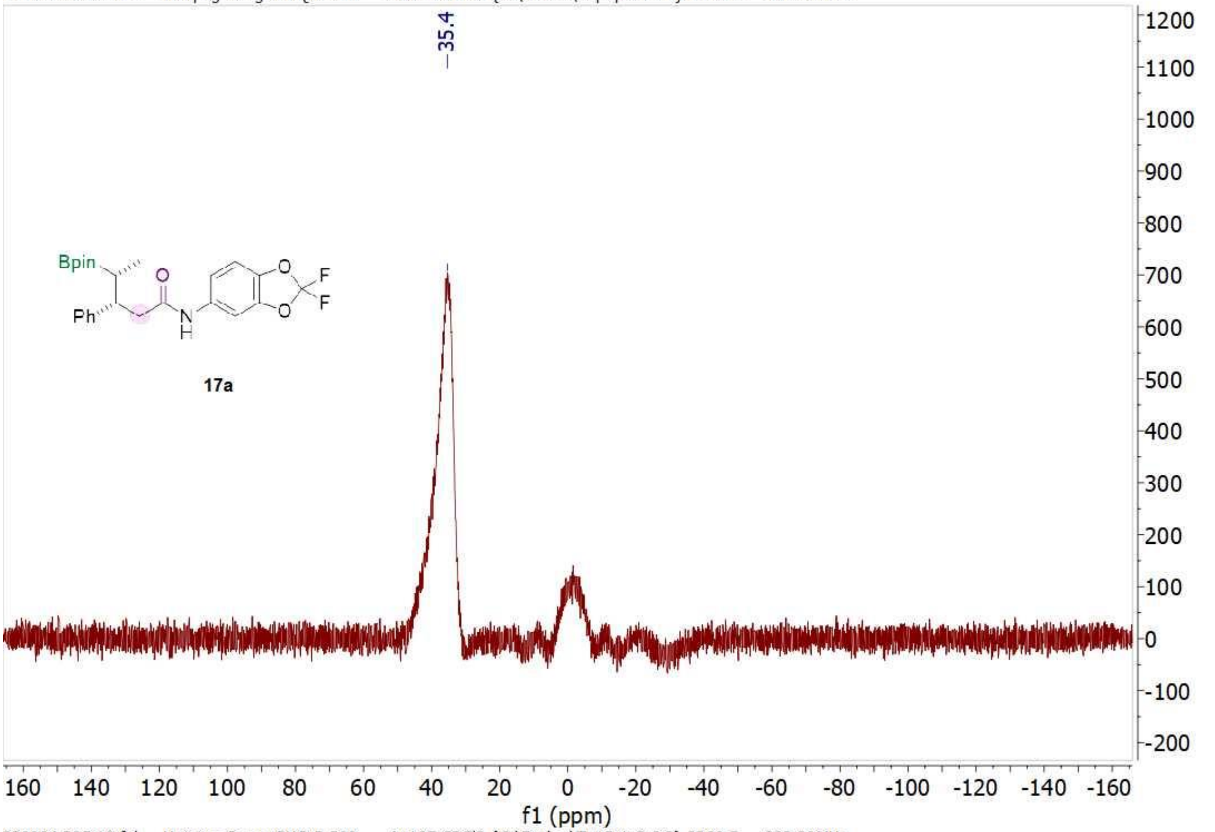

230904.305.10.fid — Huiqing Geng GHQ-P-209 — Au19F CDCl3 {C:\Bruker\TopSpin3.6.2} 2309 5 — 282.39MHz

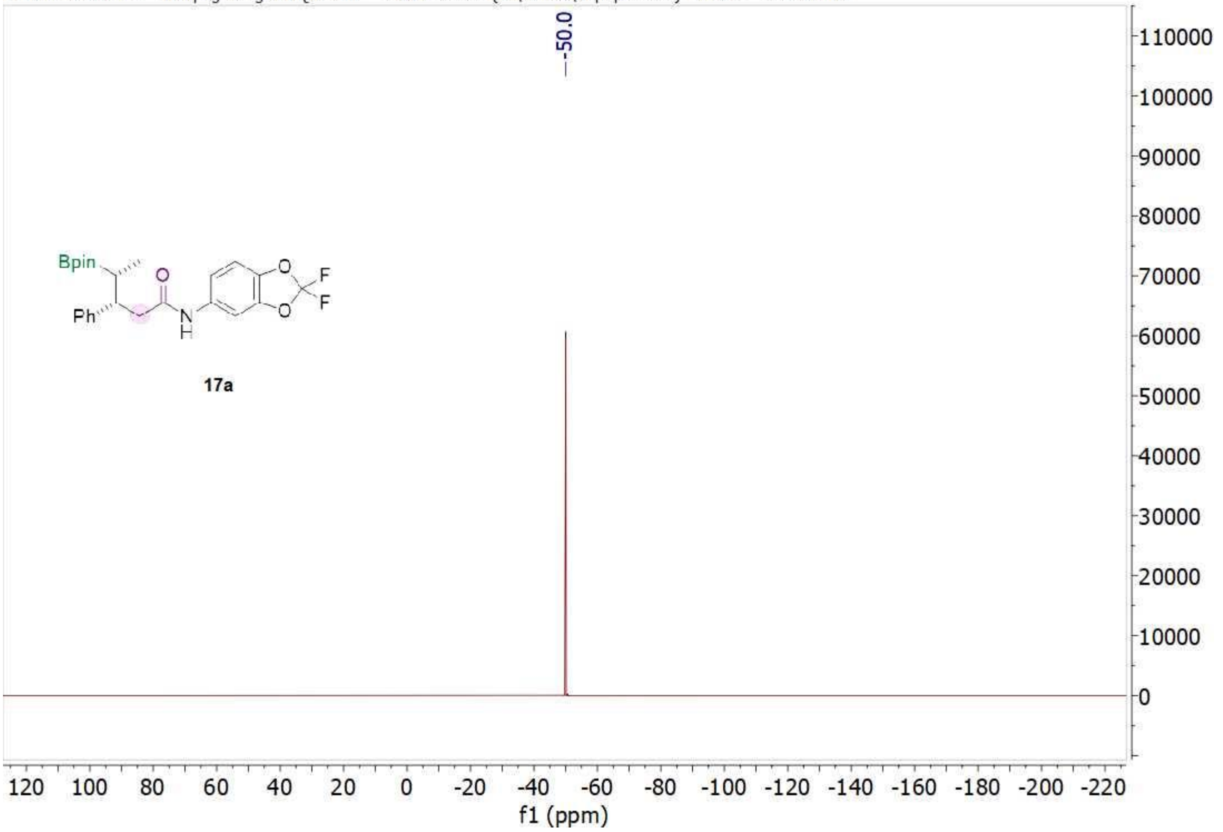

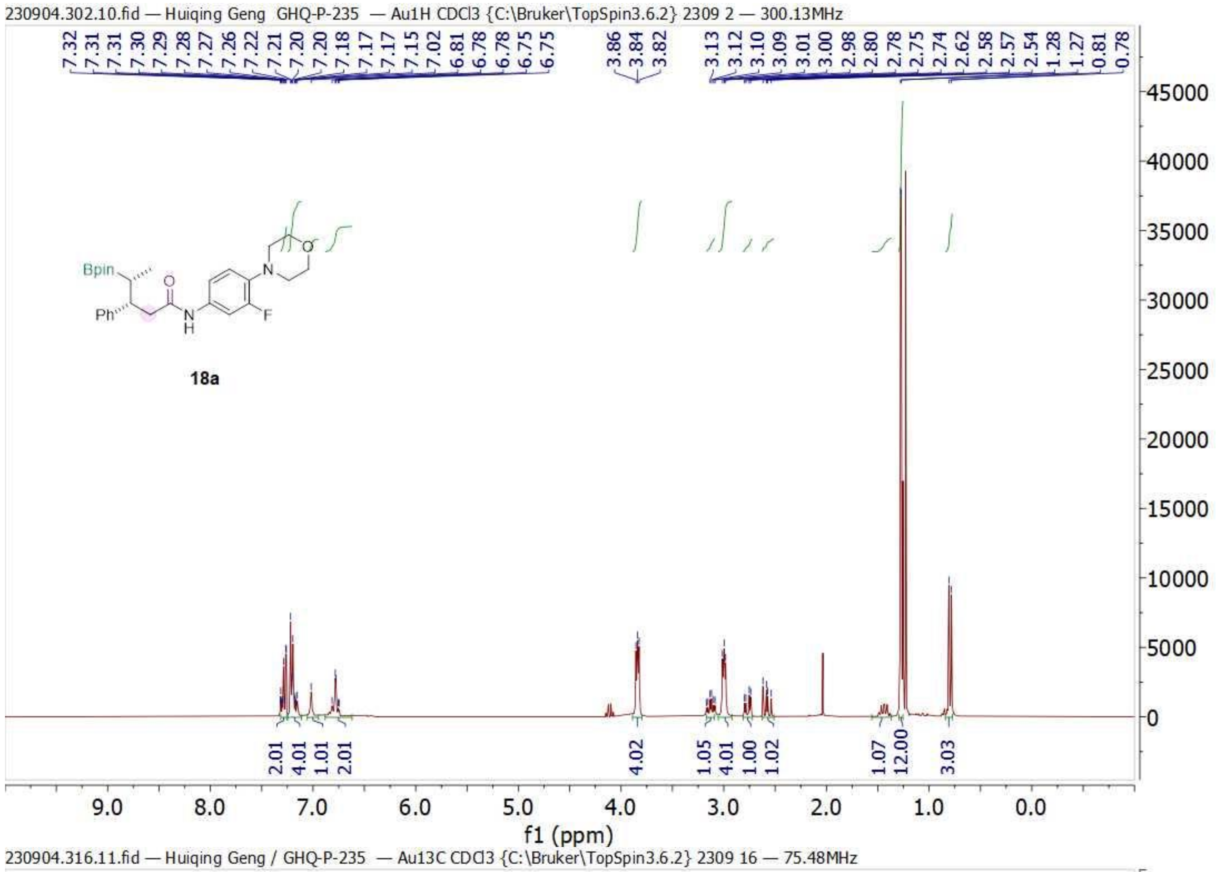
230904.302.10.fid — Huiqing Geng GHQ-P-235 — Au1H CDCl3 {C:\Bruker\TopSpin3.6.2} 2309 2 — 300.13MHz
7.32 7.31 7.30 7.29 7.28 7.27 7.26 7.22 7.21 7.20 7.20 7.18 7.17 7.15 7.02 6.81 6.78 6.78 6.75 6.75
3.86 3.84 3.82
3.13 3.12 3.10 3.09 3.01 3.00 2.98 2.80 2.78 2.75 2.74 2.62 2.58 2.57 2.54 1.28 1.27 0.81 0.78
Bpin
Ph
N
H
O
F
18a
2.01 4.01 1.01 2.01 4.02 1.05 4.01 1.00 1.02 1.07 12.00 3.03
f1 (ppm)

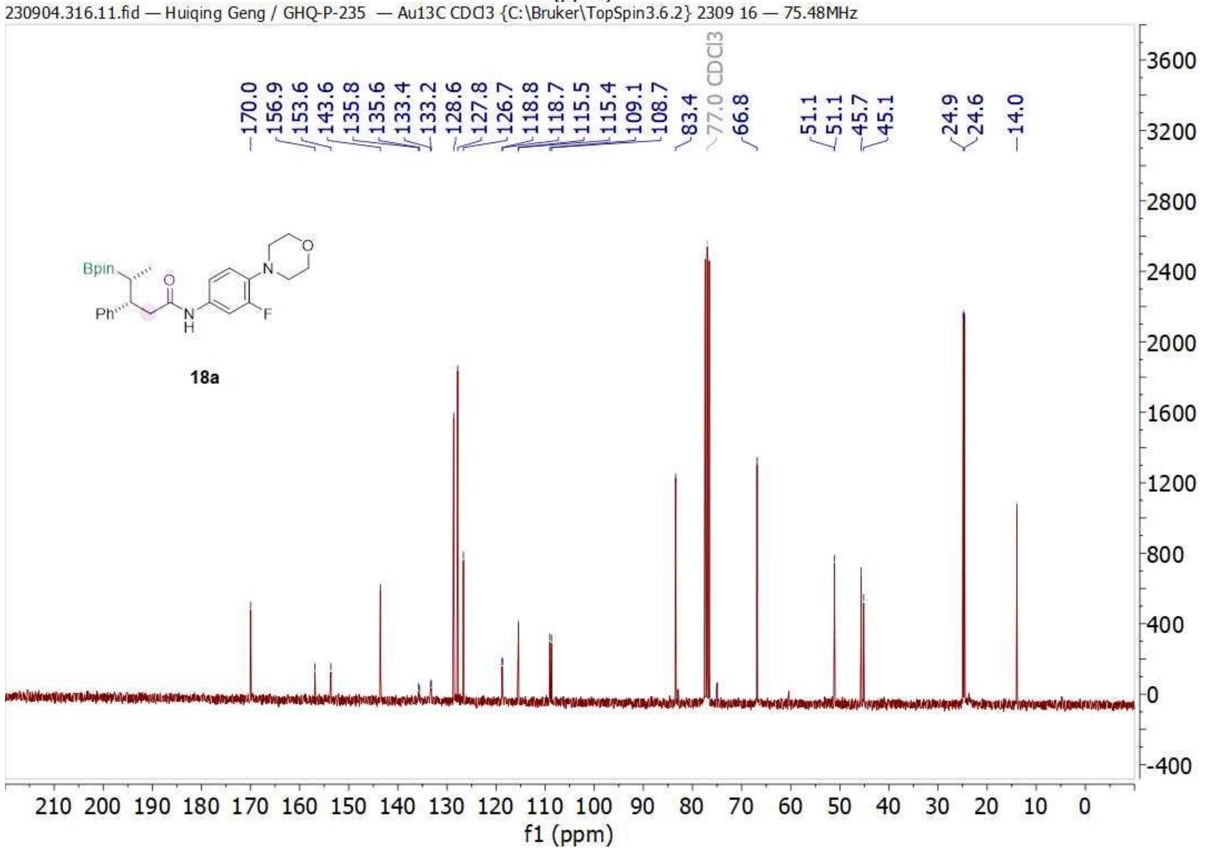
230904.316.11.fid — Huiqing Geng / GHQ-P-235 — Au13C CDCl3 {C:\Bruker\TopSpin3.6.2} 2309 16 — 75.48MHz
170.0 156.9 153.6 143.6 135.8 135.6 133.4 133.2 128.6 127.8 126.7 118.8 118.7 115.5 115.4 109.1 108.7 83.4 77.0 CDCl3 66.8 51.1 51.1 45.7 45.1 24.9 24.6 14.0
Bpin
Ph
N
H
O
F
18a
f1 (ppm)

230904.302.12.fid — Huiqing Geng GHQ-P-235 — Au11B CDCl3 {C:\Bruker\TopSpin3.6.2} 2309 2 — 96.29MHz

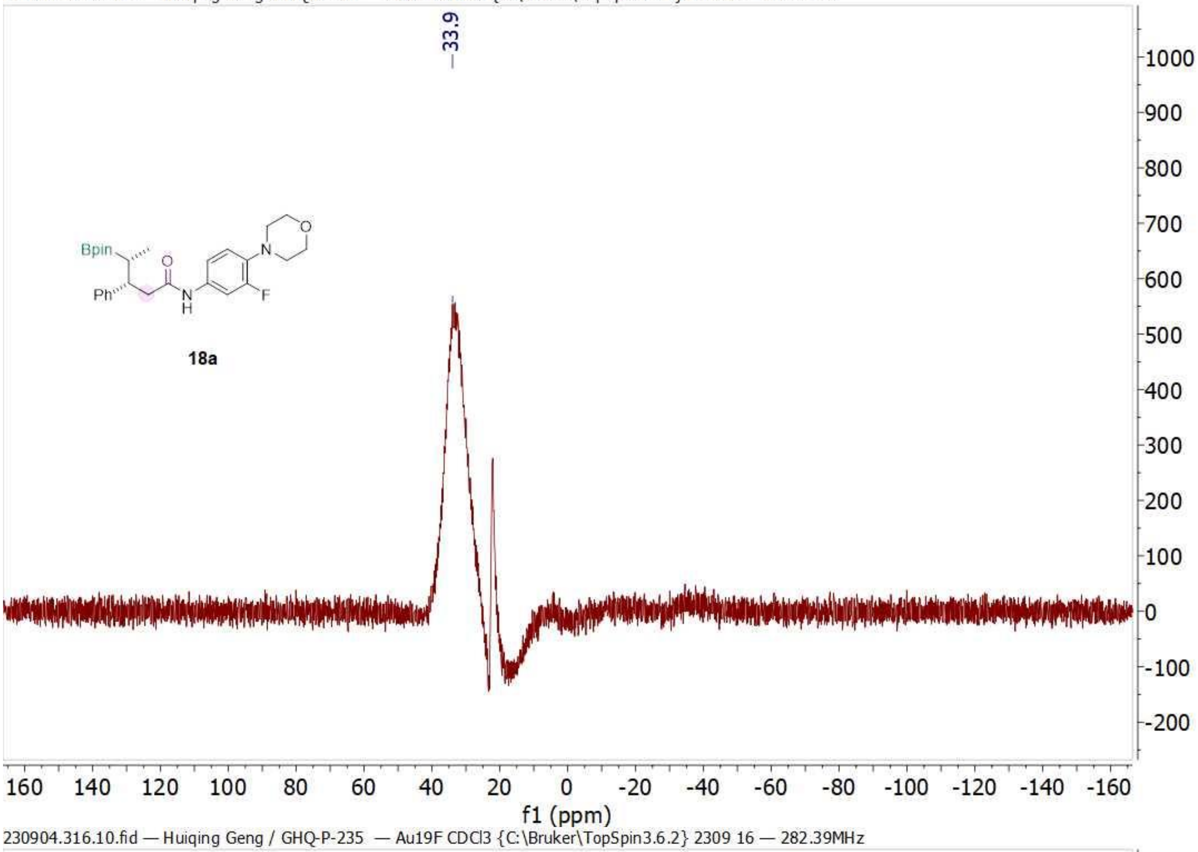

230904.316.10.fid — Huiqing Geng / GHQ-P-235 — Au19F CDCl3 {C:\Bruker\TopSpin3.6.2} 2309 16 — 282.39MHz

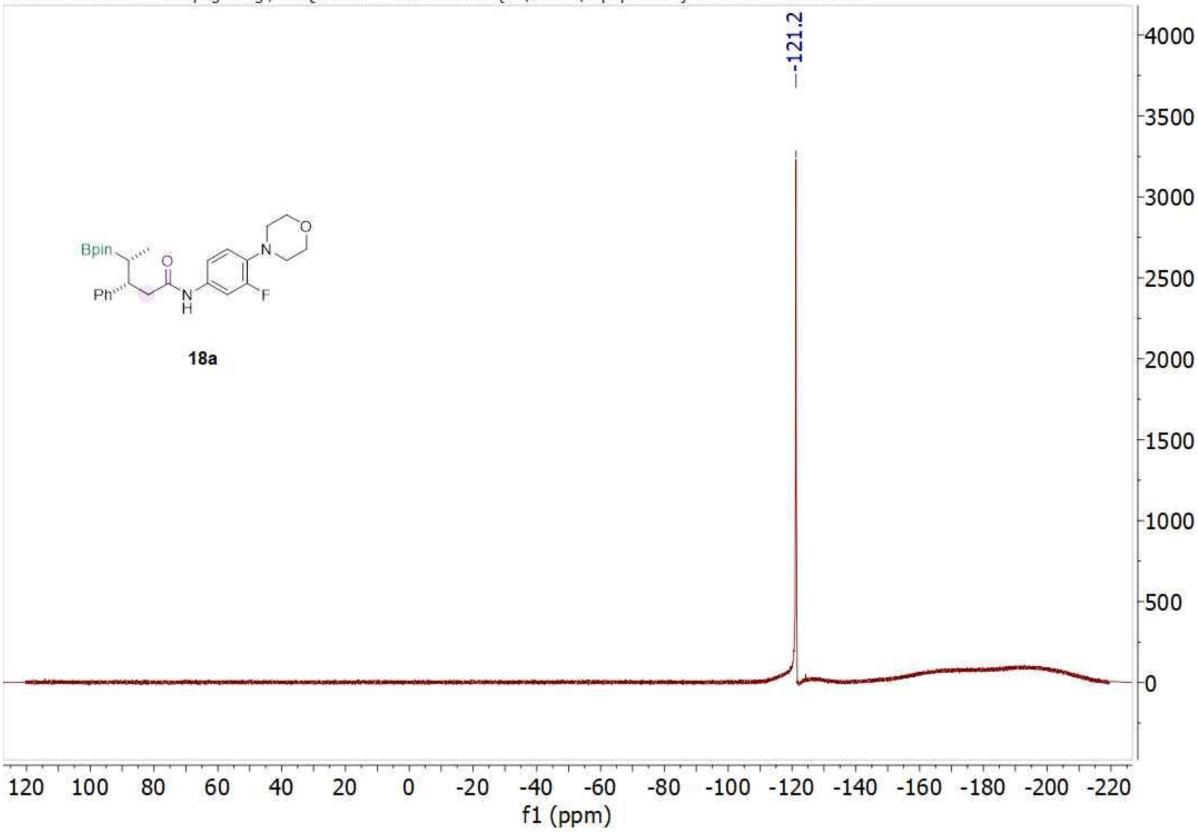

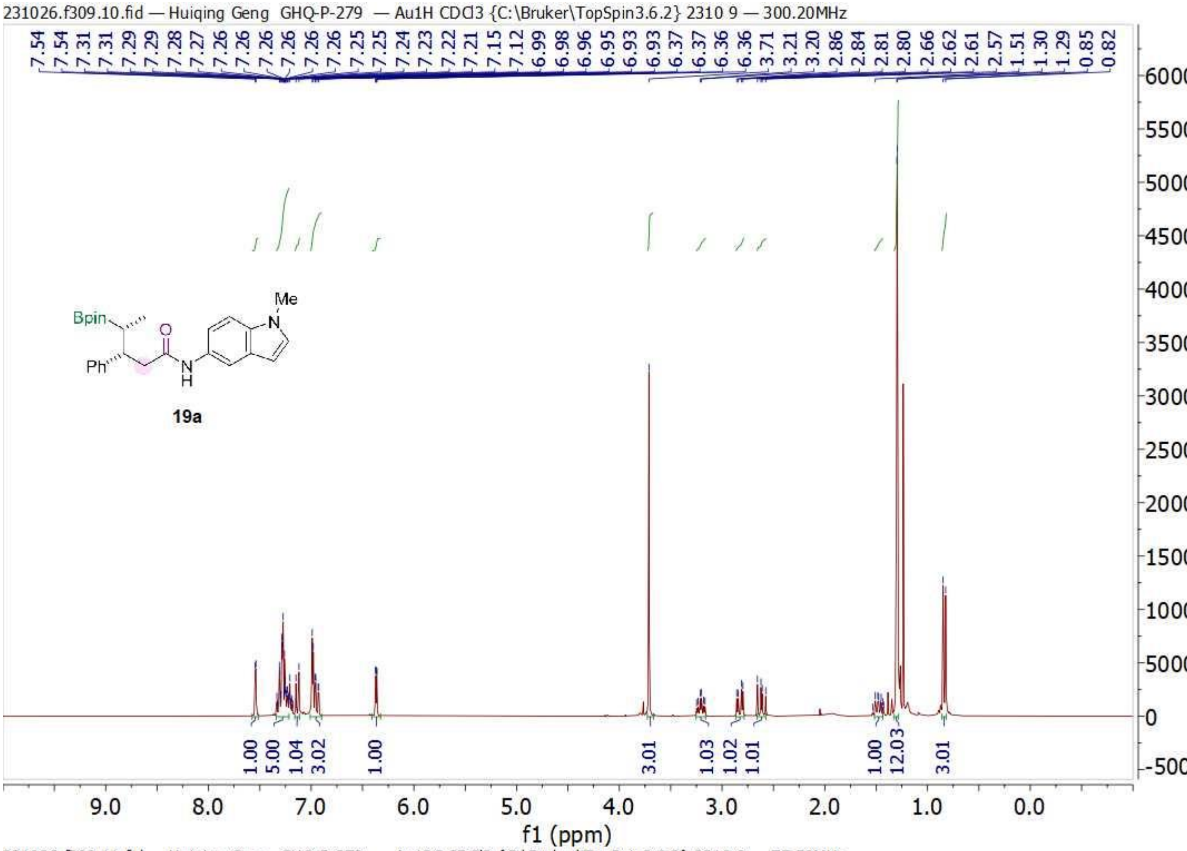
231026.f309.10.fid — Huiqing Geng GHQ-P-279 — Au1H CDCl3 {C:\Bruker\TopSpin3.6.2} 2310 9 — 300.20MHz
7.54 7.54 7.31 7.29 7.29 7.28 7.27 7.26 7.26 7.26 7.26 7.26 7.25 7.25 7.24 7.23 7.22 7.21 7.15 7.12 6.99 6.98 6.96 6.95 6.93 6.93 6.37 6.37 6.36 6.36 3.71 3.21 3.20 2.86 2.84 2.81 2.80 2.66 2.62 2.61 2.57 1.51 1.30 1.29 0.85 0.82
Bpin
Me
N
Ph
H
19a
1.00 5.00 1.04 3.02 1.00 3.01 1.03 1.02 1.01 1.00 12.03 3.01
f1 (ppm)

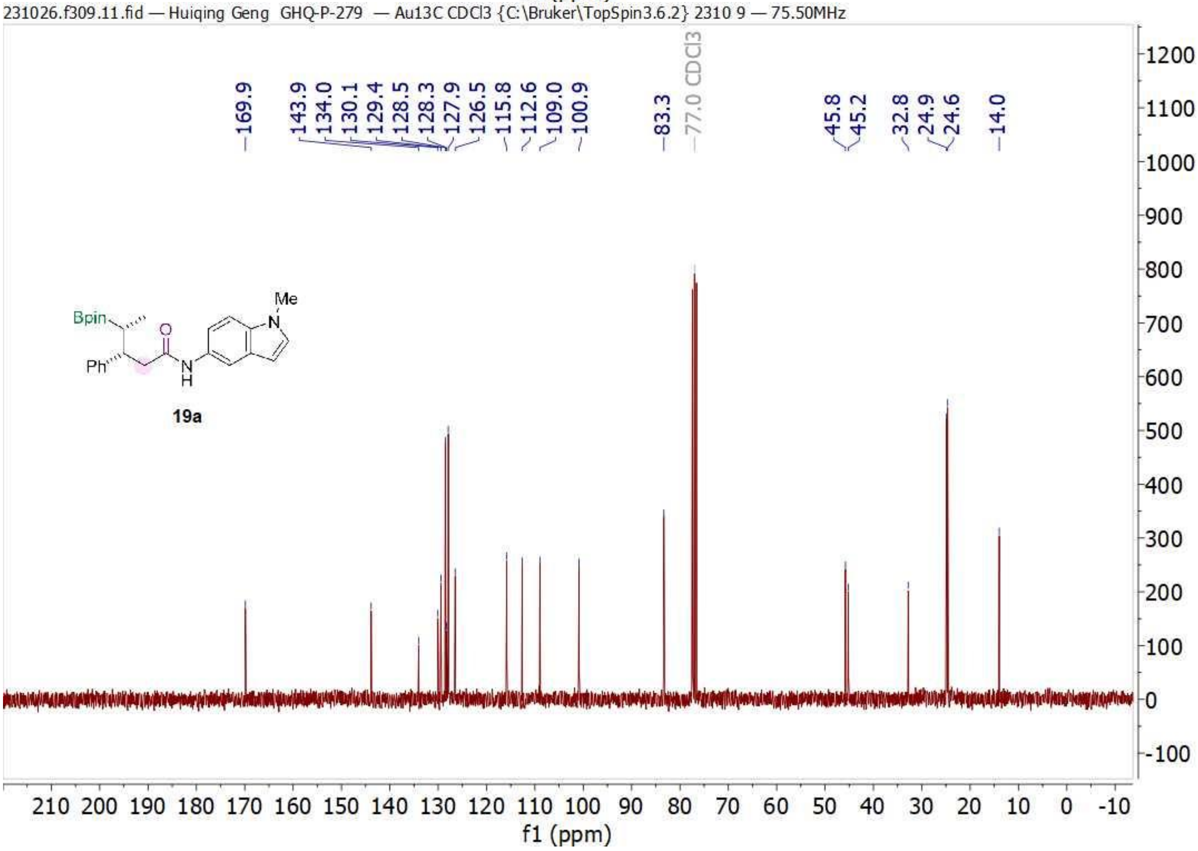
231026.f309.11.fid — Huiqing Geng GHQ-P-279 — Au13C CDCl3 {C:\Bruker\TopSpin3.6.2} 2310 9 — 75.50MHz
169.9 143.9 134.0 130.1 129.4 128.5 128.3 127.9 126.5 115.8 112.6 109.0 100.9 83.3 77.0 CDCl3 45.8 45.2 32.8 24.9 24.6 14.0
Bpin
Me
N
Ph
H
19a
f1 (ppm)

231026.f309.12.fid — Huiqing Geng GHQ-P-279 — Au11B(H-gek) CDCl3 {C:\Bruker\TopSpin3.6.2} 2310 9 — 96.32MHz

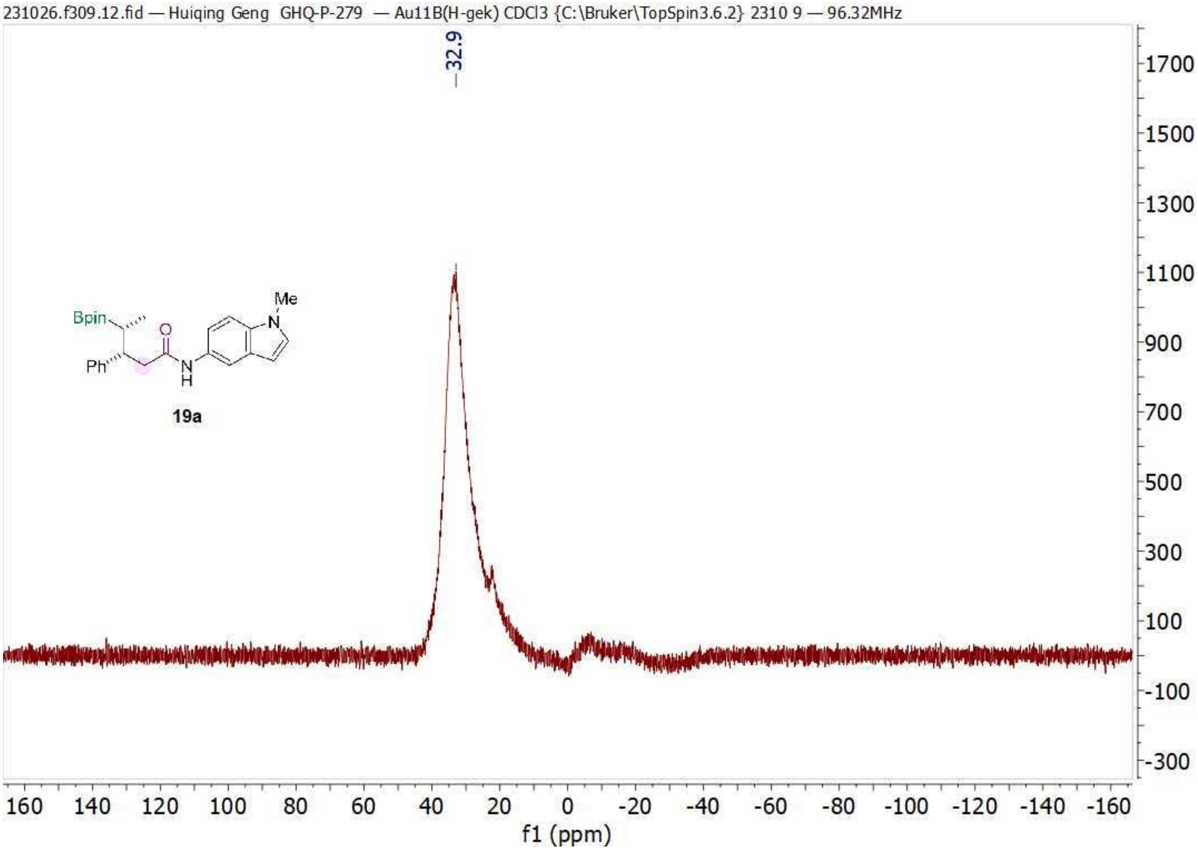

231024.f312.10.fid — Huiqing Geng GHQ-P-319 — Au1H CDCl3 {C:\Bruker\TopSpin3.6.2} 2310 12 — 300.20MHz

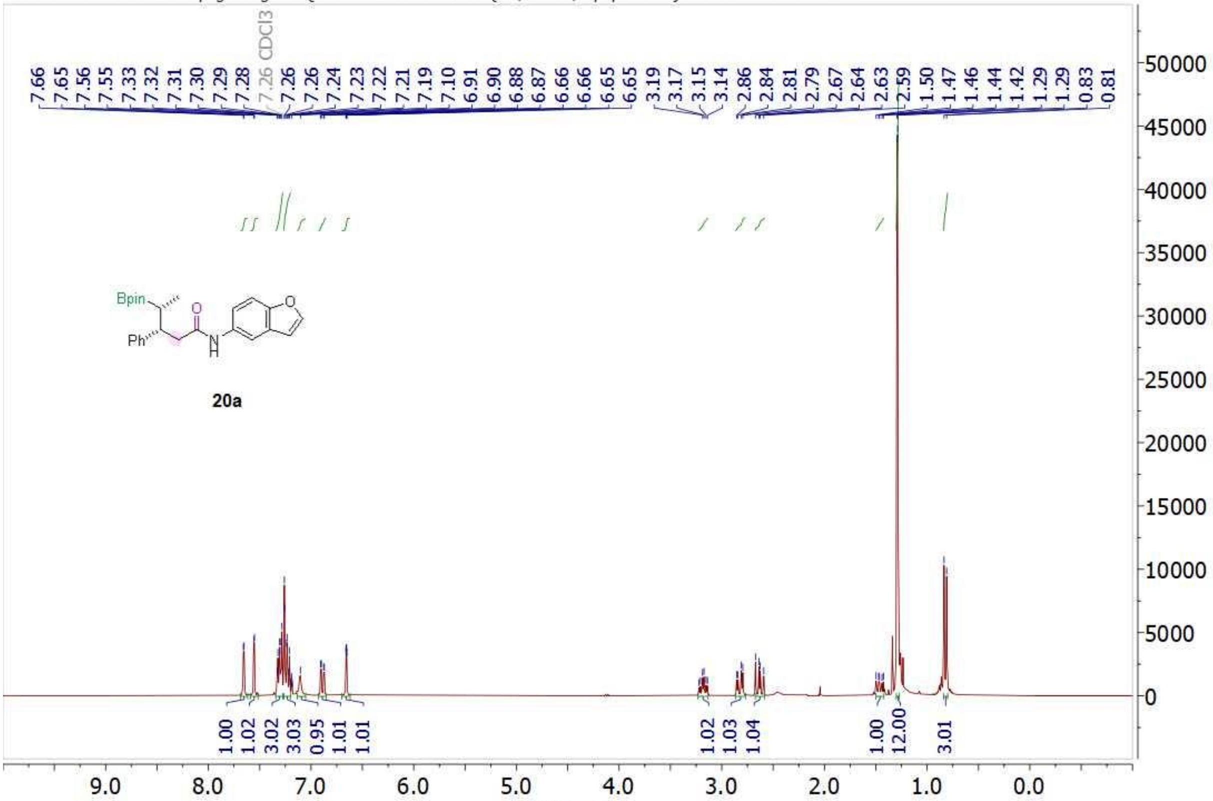

20a

231024.f312.11.fid — Huiqing Geng GHQ-P-319 — Au13C CDCl3 {C:\Bruker\TopSpin3.6.2} 2310 12 — 75.50MHz

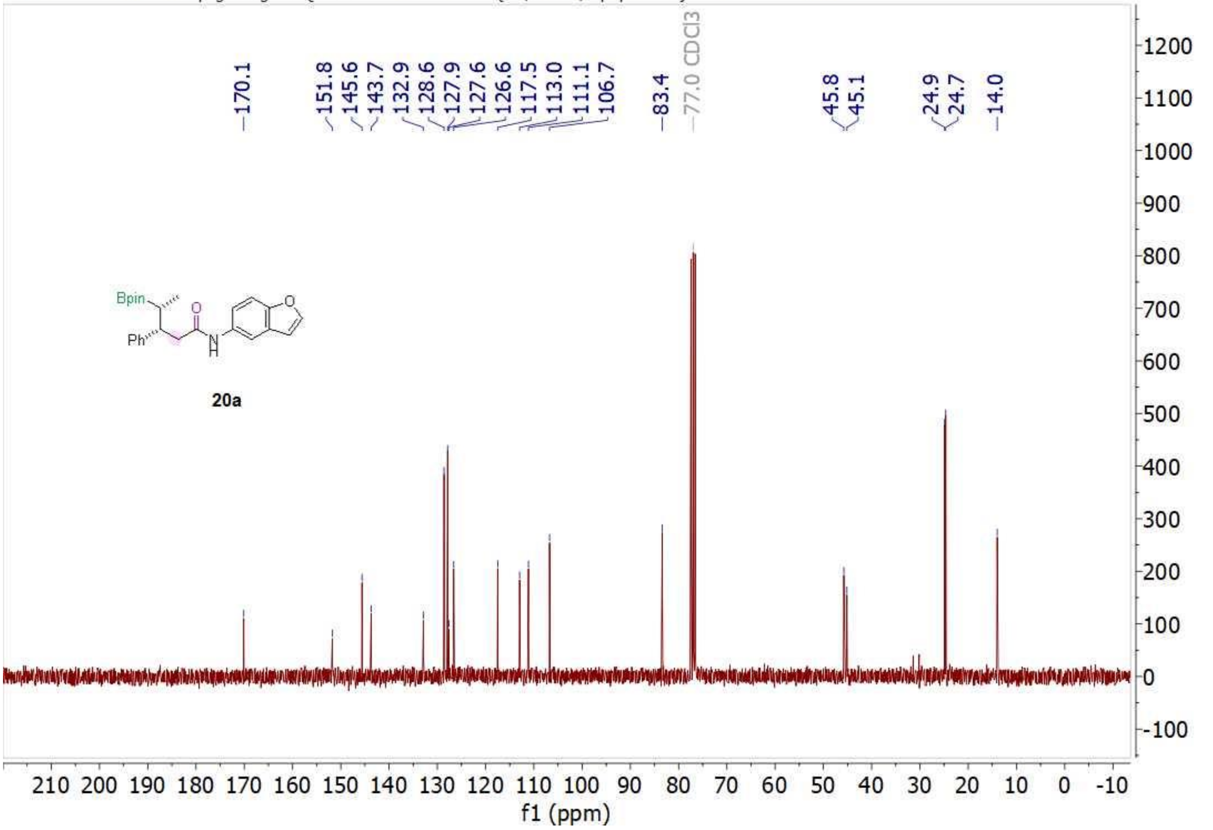

20a

231024.f312.12.fid — Huiqing Geng GHQ-P-319 — Au11B(H-gek) CDCl3 {C:\Bruker\TopSpin3.6.2} 2310 12 — 96.32MHz

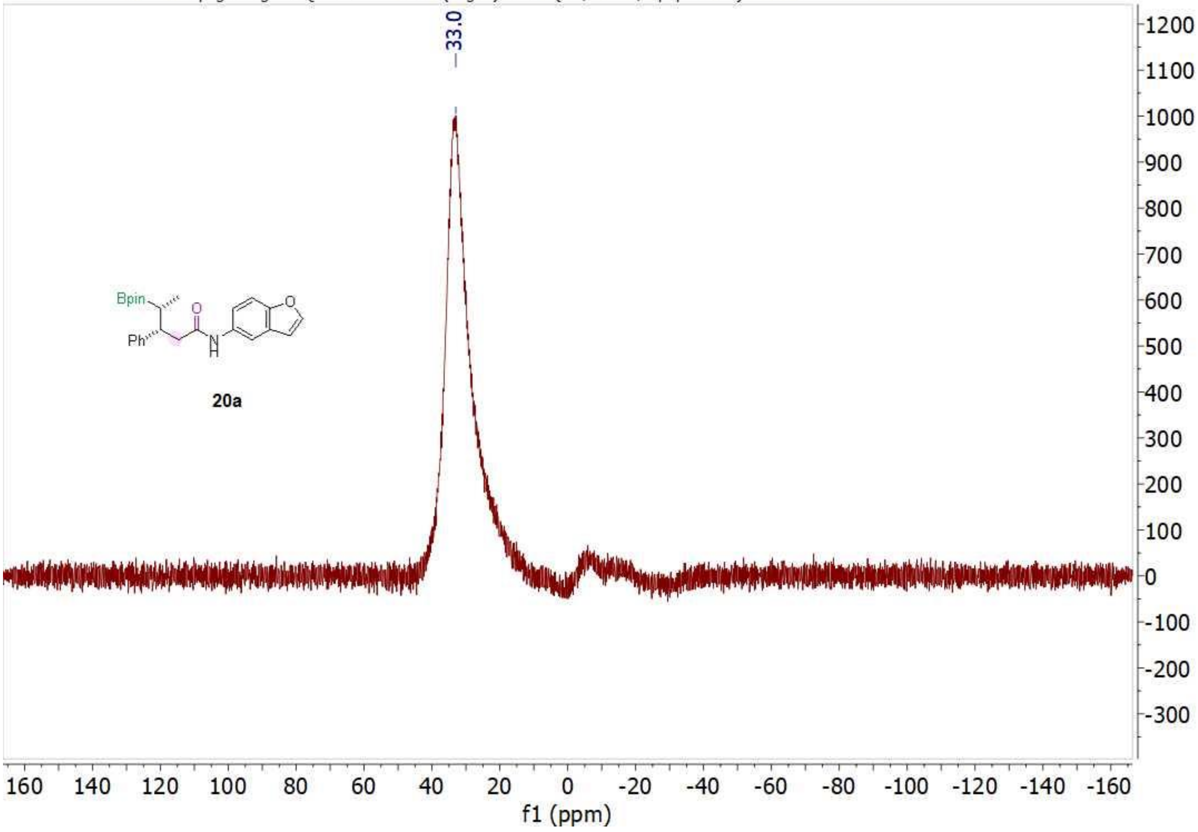

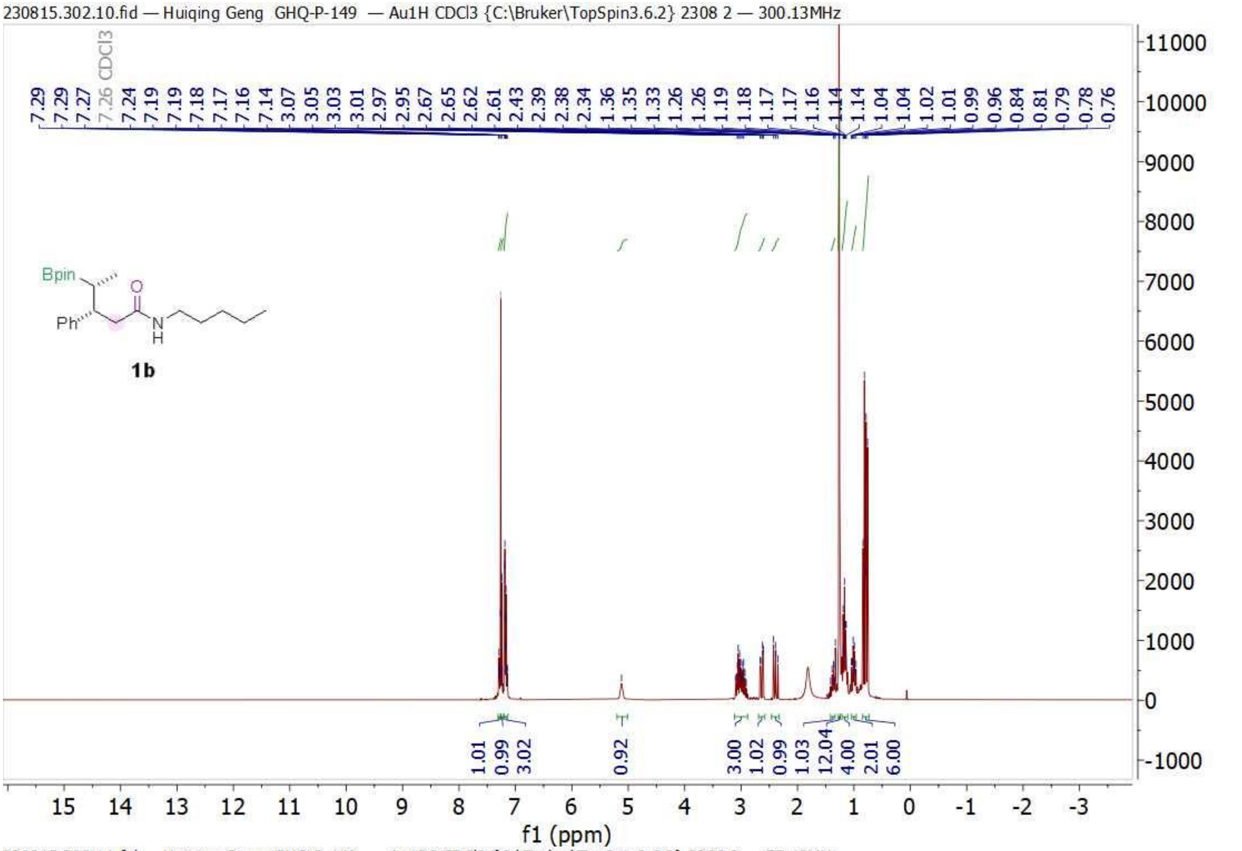
230815.302.10.fid — Huiqing Geng GHQ-P-149 — Au1H CDCl3 {C:\Bruker\TopSpin3.6.2} 2308 2 — 300.13MHz
7.29 7.29 7.27 7.26 CDCl3 7.24 7.19 7.19 7.18 7.17 7.16 7.14 3.07 3.05 3.03 3.01 2.97 2.95 2.67 2.65 2.62 2.61 2.43 2.39 2.38 2.34 1.36 1.35 1.33 1.26 1.26 1.19 1.18 1.17 1.17 1.16 1.14 1.14 1.04 1.04 1.02 1.01 0.99 0.96 0.84 0.81 0.79 0.78 0.76
Bpin
O
Ph
N
H
1b
1.01 0.99 3.02 0.92 3.00 1.02 0.99 1.03 12.04 4.00 2.01 6.00
f1 (ppm)

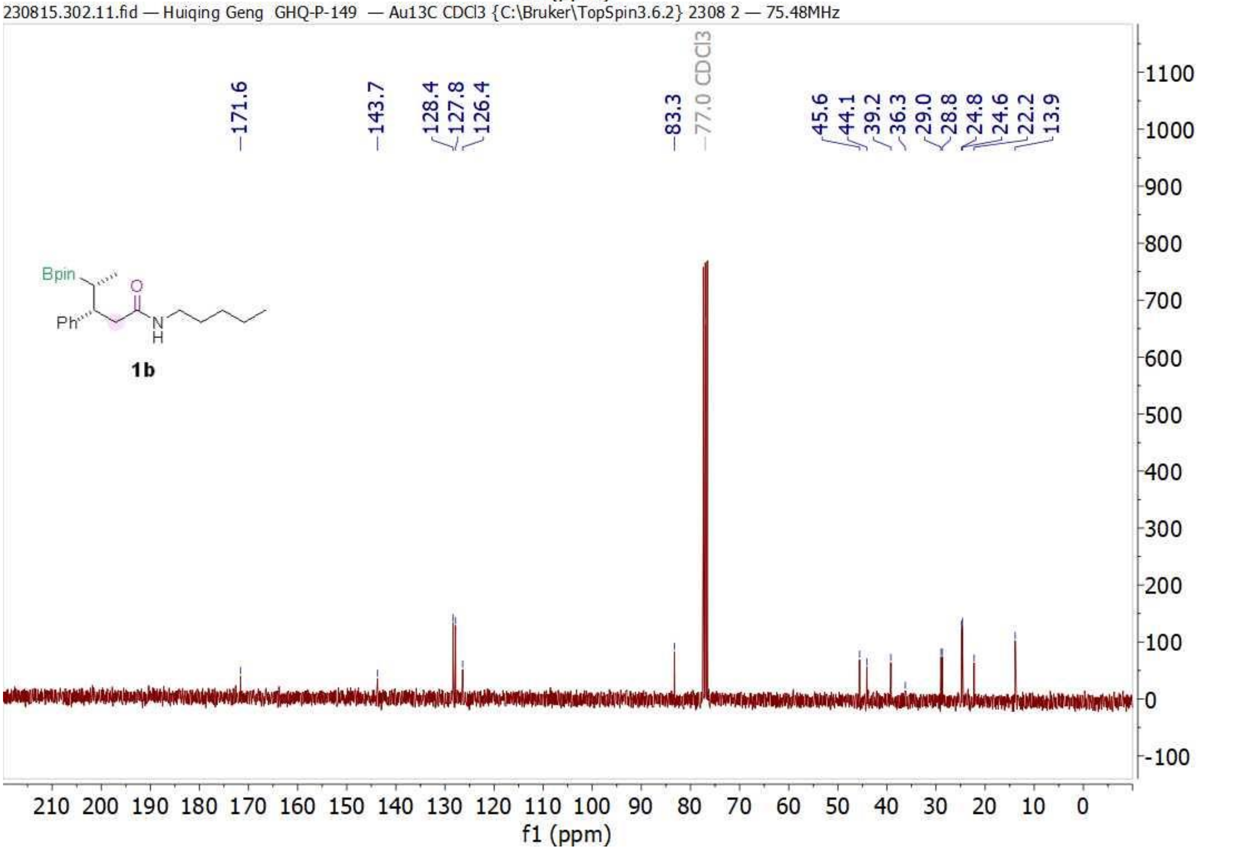
230815.302.11.fid — Huiqing Geng GHQ-P-149 — Au13C CDCl3 {C:\Bruker\TopSpin3.6.2} 2308 2 — 75.48MHz
171.6 143.7 128.4 127.8 126.4 83.3 77.0 CDCl3 45.6 44.1 39.2 36.3 29.0 28.8 24.8 24.6 22.2 13.9
Bpin
O
Ph
N
H
1b
f1 (ppm)

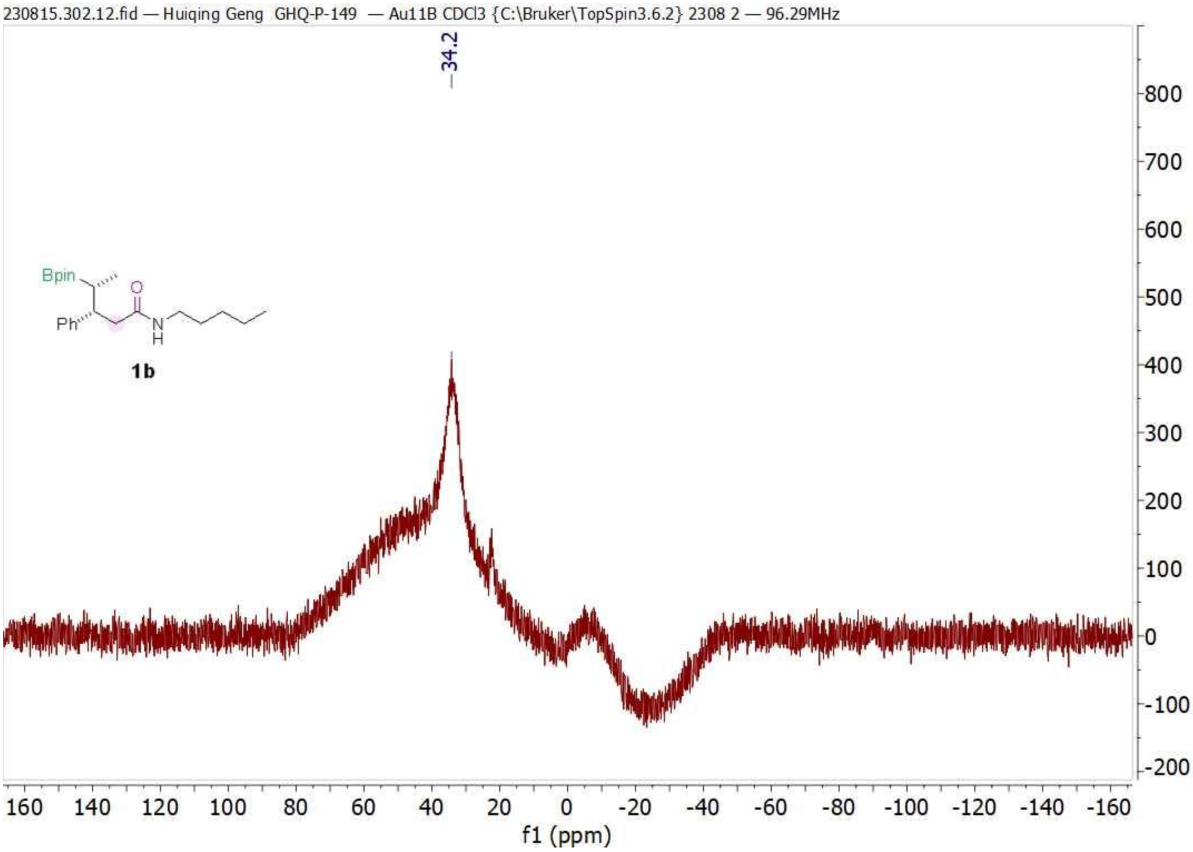
230815.302.12.fid — Huiqing Geng GHQ-P-149 — Au11B CDCl3 {C:\Bruker\TopSpin3.6.2} 2308 2 — 96.29MHz
34.2
Bpin
O
Ph
N
H
1b
800
700
600
500
400
300
200
100
0
-100
-200
160 140 120 100 80 60 40 20 0 -20 -40 -60 -80 -100 -120 -140 -160
f1 (ppm)

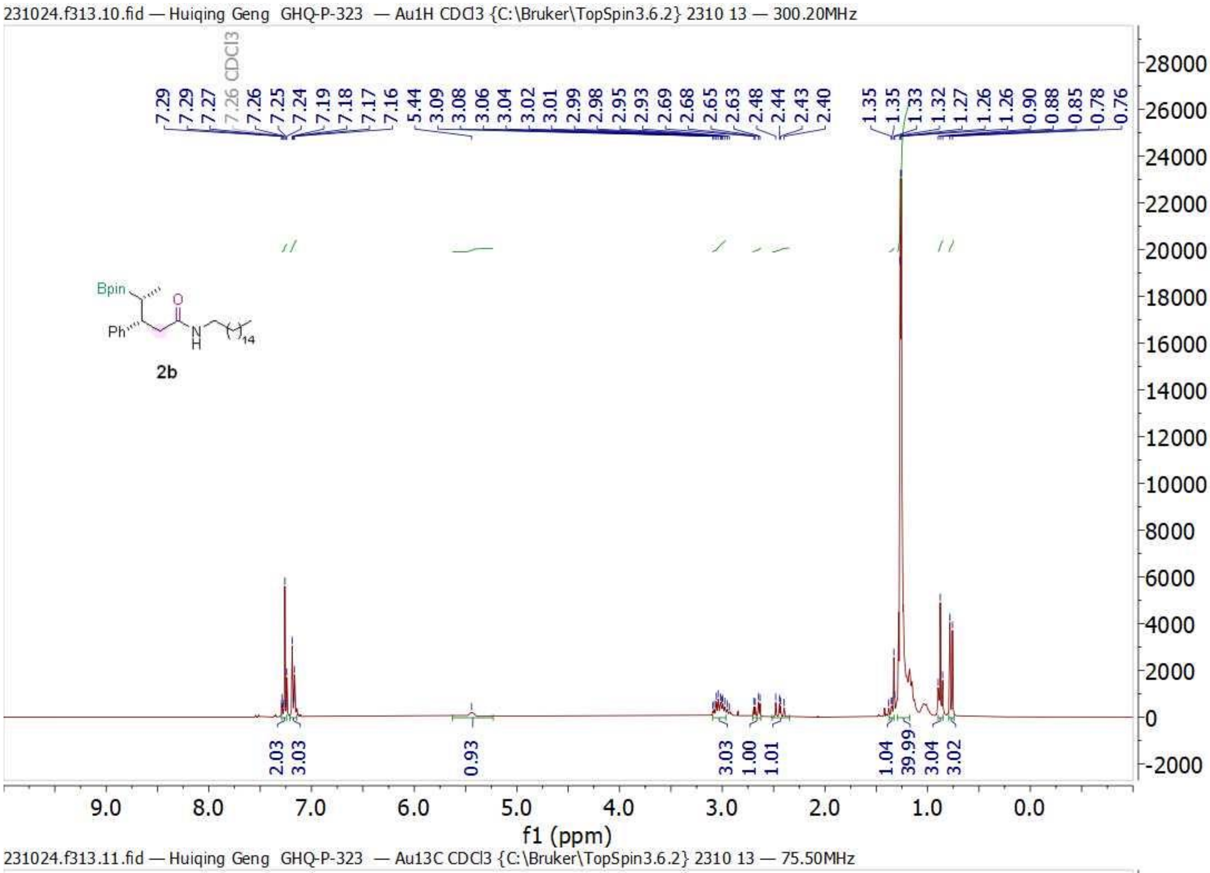
231024.f313.10.fid — Huiqing Geng GHQ-P-323 — Au1H CDCl3 {C:\Bruker\TopSpin3.6.2} 2310 13 — 300.20MHz
7.29 7.29 7.27 7.26 CDCl3 7.26 7.25 7.24 7.19 7.18 7.17 7.16 5.44 3.09 3.08 3.06 3.04 3.02 3.01 2.99 2.98 2.95 2.93 2.69 2.68 2.65 2.63 2.48 2.44 2.43 2.40 1.35 1.35 1.33 1.32 1.27 1.26 1.26 0.90 0.88 0.85 0.78 0.76
Bpin
Ph
N
H
14
2b
2.03 3.03 0.93 3.03 1.00 1.01 1.04 39.99 3.04 3.02
f1 (ppm)
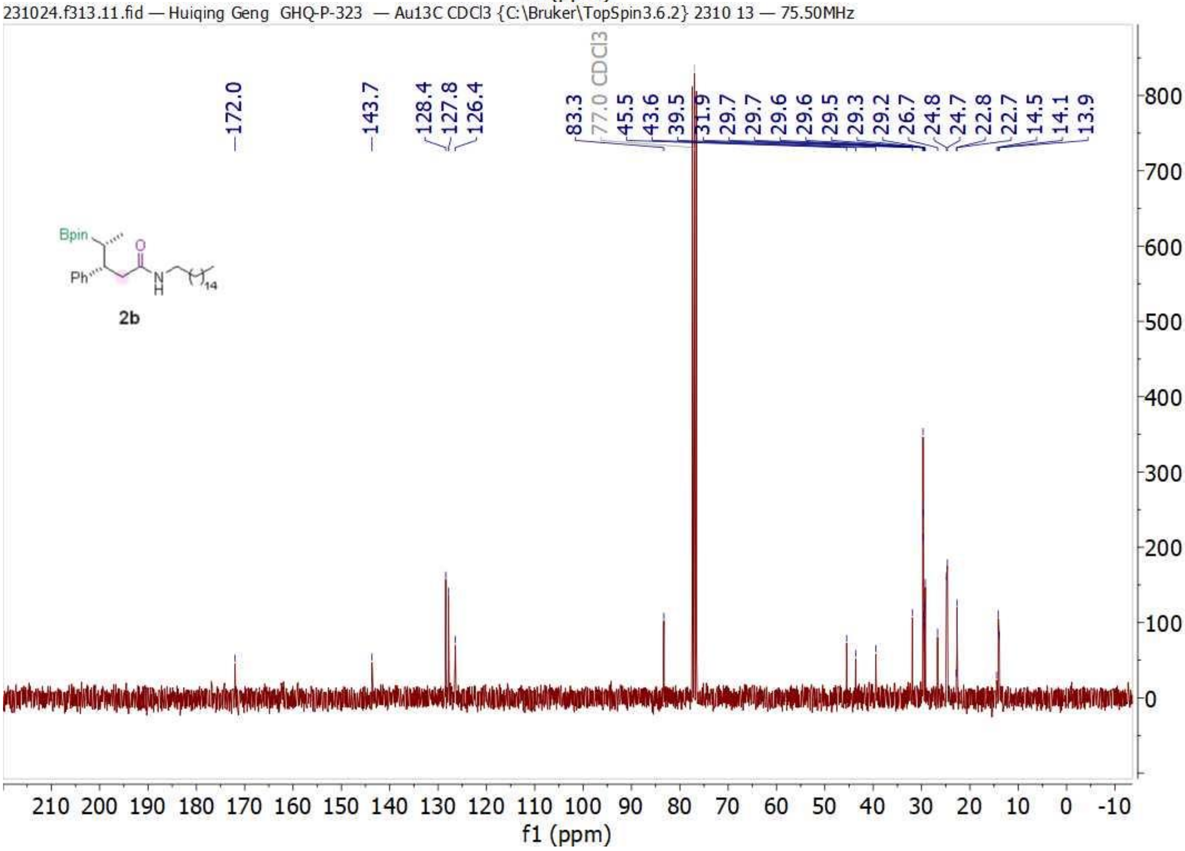
231024.f313.11.fid — Huiqing Geng GHQ-P-323 — Au13C CDCl3 {C:\Bruker\TopSpin3.6.2} 2310 13 — 75.50MHz
172.0 143.7 128.4 127.8 126.4 83.3 77.0 CDCl3 45.5 43.6 39.5 31.9 29.7 29.7 29.6 29.6 29.5 29.3 29.2 26.7 24.8 24.7 22.8 22.7 14.5 14.1 13.9
Bpin
Ph
N
H
14
2b
f1 (ppm)

231024.f313.12.fid — Huiqing Geng GHQ-P-323 — Au11B(H-gek) CDCl3 {C:\Bruker\TopSpin3.6.2} 2310 13 — 96.32MHz

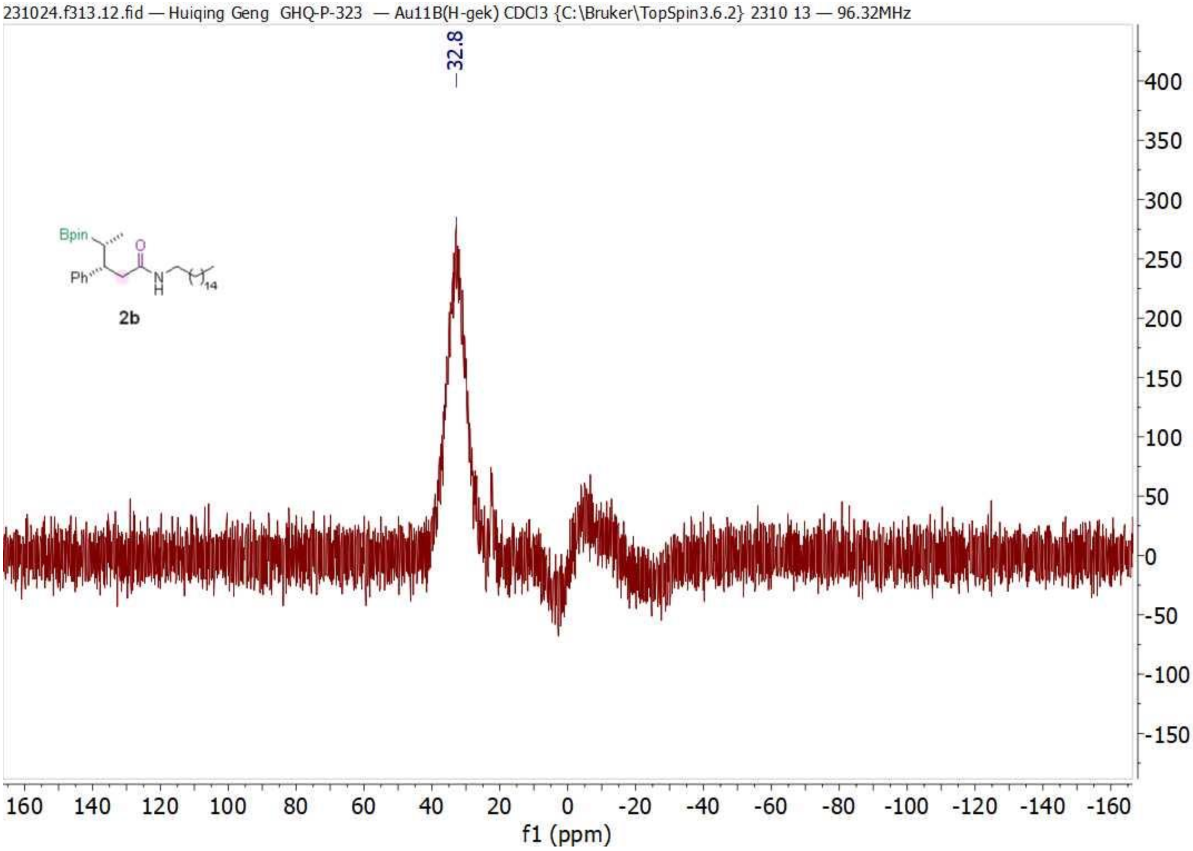

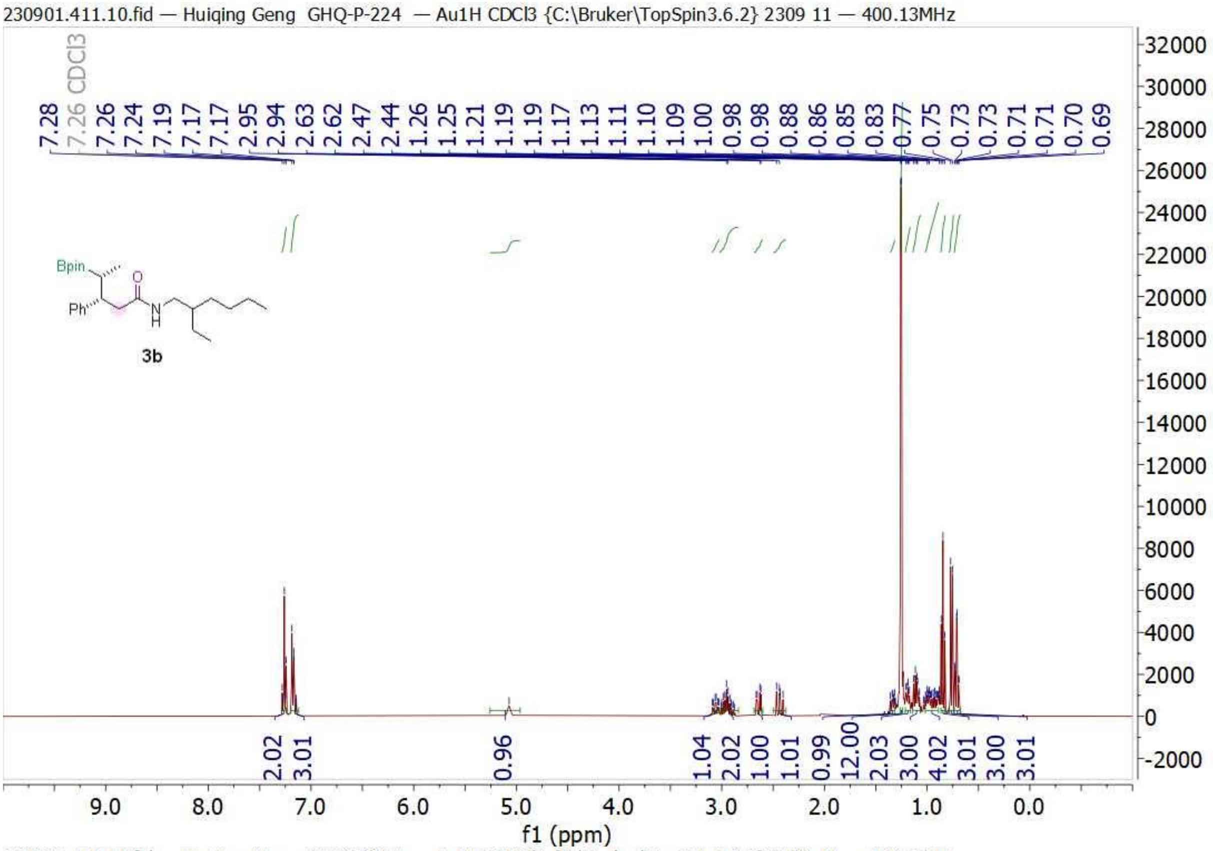
230901.411.10.fid — Huiqing Geng GHQ-P-224 — Au1H CDCl3 {C:\Bruker\TopSpin3.6.2} 2309 11 — 400.13MHz
7.28
7.26 CDCl3
7.26 7.24 7.19 7.17 7.17 2.95 2.94 2.63 2.62 2.47 2.44 1.26 1.25 1.21 1.19 1.19 1.17 1.13 1.11 1.10 1.09 1.00 0.98 0.98 0.88 0.86 0.85 0.83 0.77 0.75 0.75 0.73 0.73 0.71 0.71 0.70 0.69
Bpin
Ph
O
N
H
3b
2.02 3.01 0.96 1.04 2.02 1.00 1.01 0.99 12.00 2.03 3.00 3.00 4.02 3.01 3.00 3.01
f1 (ppm)

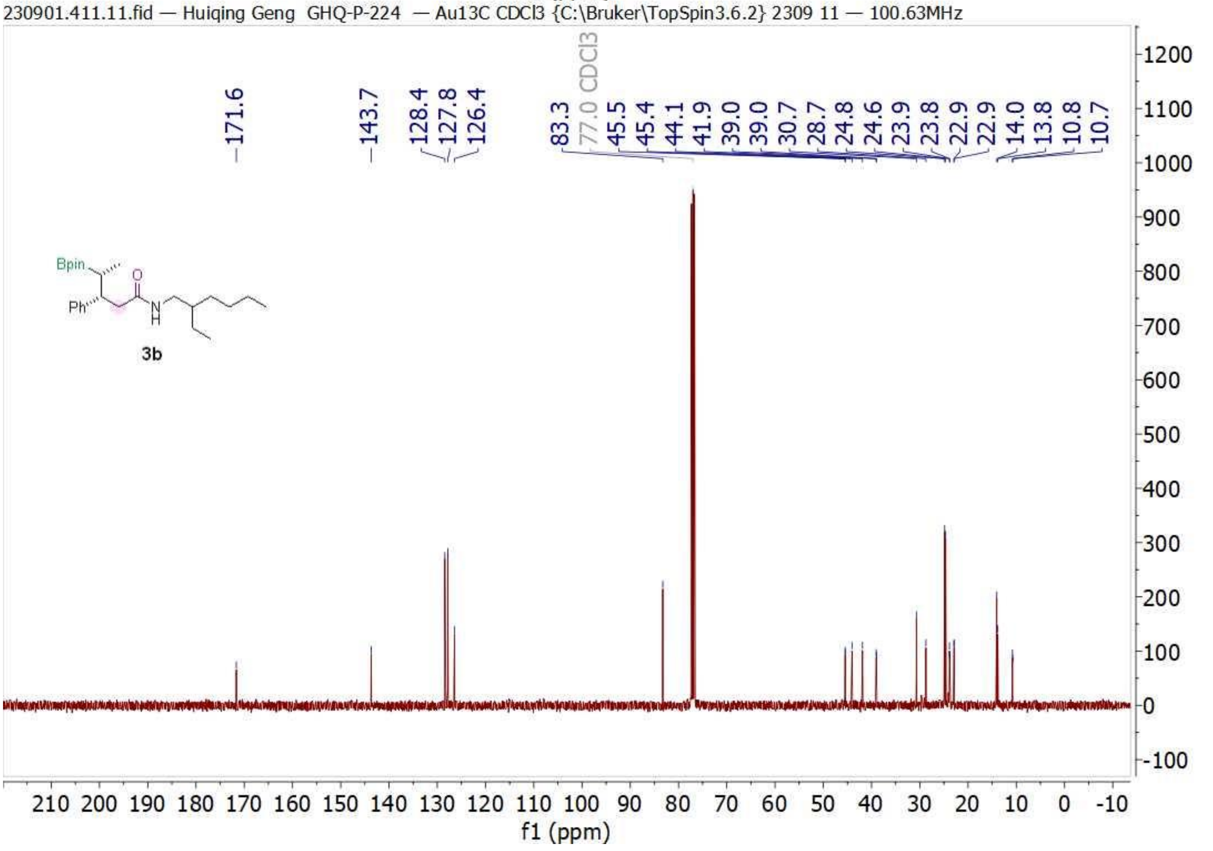
230901.411.11.fid — Huiqing Geng GHQ-P-224 — Au13C CDCl3 {C:\Bruker\TopSpin3.6.2} 2309 11 — 100.63MHz
171.6
143.7
128.4 127.8 126.4
83.3
77.0 CDCl3
45.5 45.4 44.1 41.9 39.0 39.0 30.7 28.7 24.8 24.6 23.9 23.8 22.9 22.9 14.0 13.8 10.8 10.7
Bpin
Ph
O
N
H
3b
f1 (ppm)

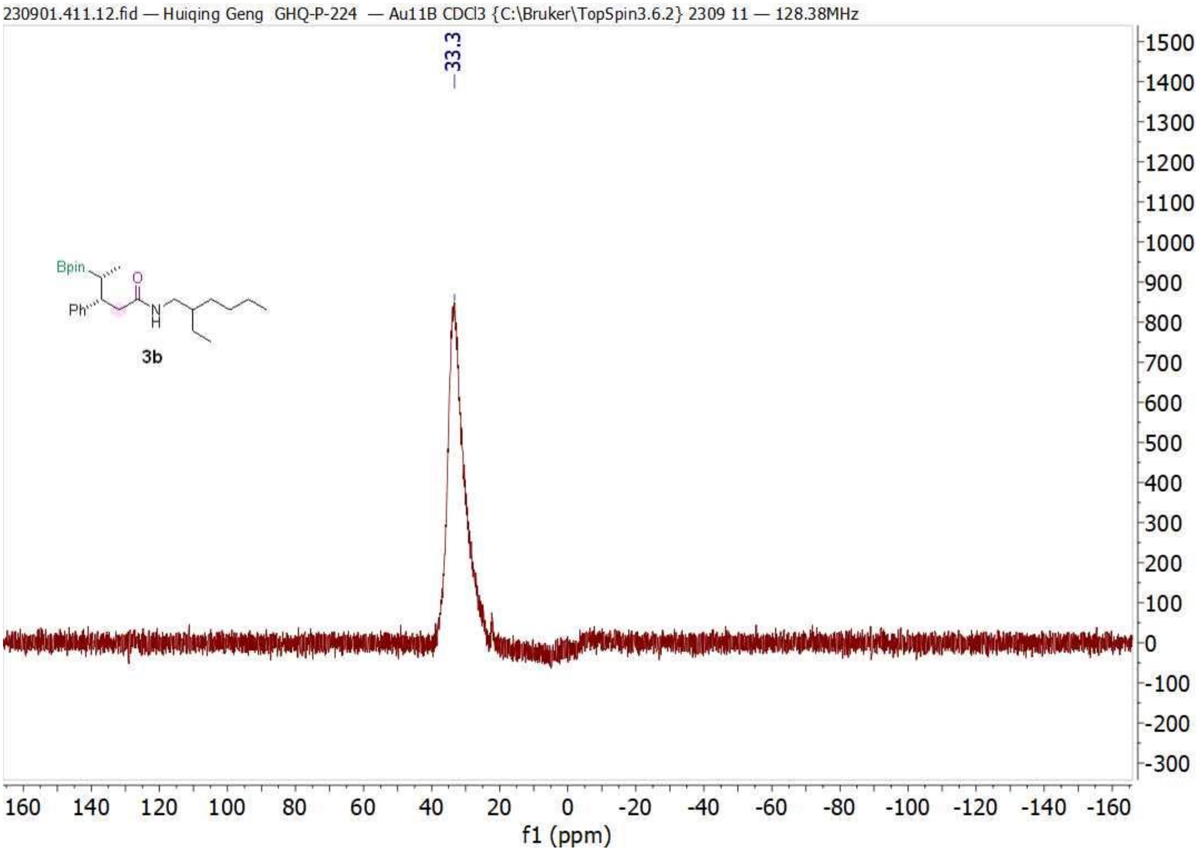
230901.411.12.fid — Huiqing Geng GHQ-P-224 — Au11B CDCl3 {C:\Bruker\TopSpin3.6.2} 2309 11 — 128.38MHz
—33.3
Bpin
Ph
O
N
H
3b
f1 (ppm)

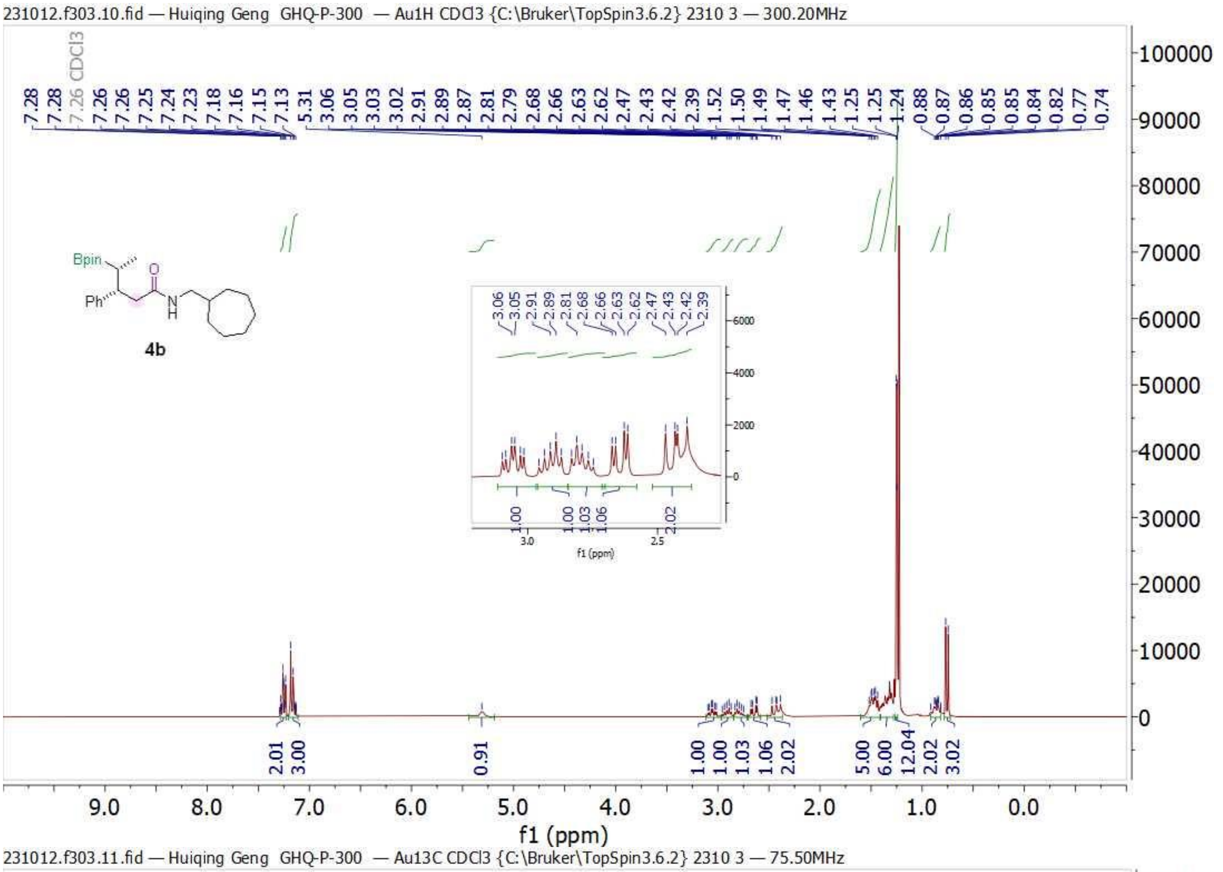
231012.f303.10.fid — Huiqing Geng GHQ-P-300 — Au1H CDCl3 {C:\Bruker\TopSpin3.6.2} 2310 3 — 300.20MHz
Bpin
Ph
O
N
H
4b
f1 (ppm)

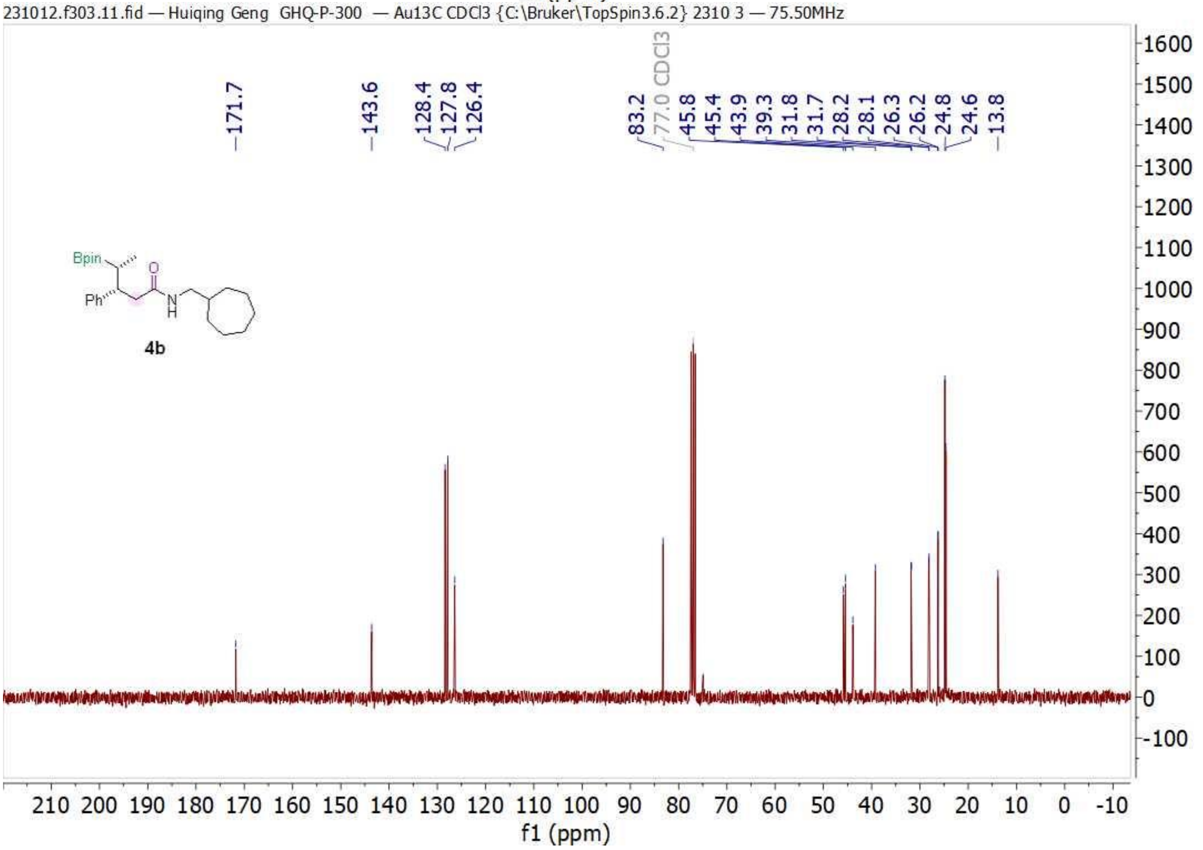
231012.f303.11.fid — Huiqing Geng GHQ-P-300 — Au13C CDCl3 {C:\Bruker\TopSpin3.6.2} 2310 3 — 75.50MHz
171.7
143.6
128.4
127.8
126.4
83.2
77.0 CDCl3
45.8
45.4
43.9
39.3
31.8
31.7
28.2
28.1
26.3
26.2
24.8
24.6
13.8
Bpin
Ph
O
N
H
4b
f1 (ppm)

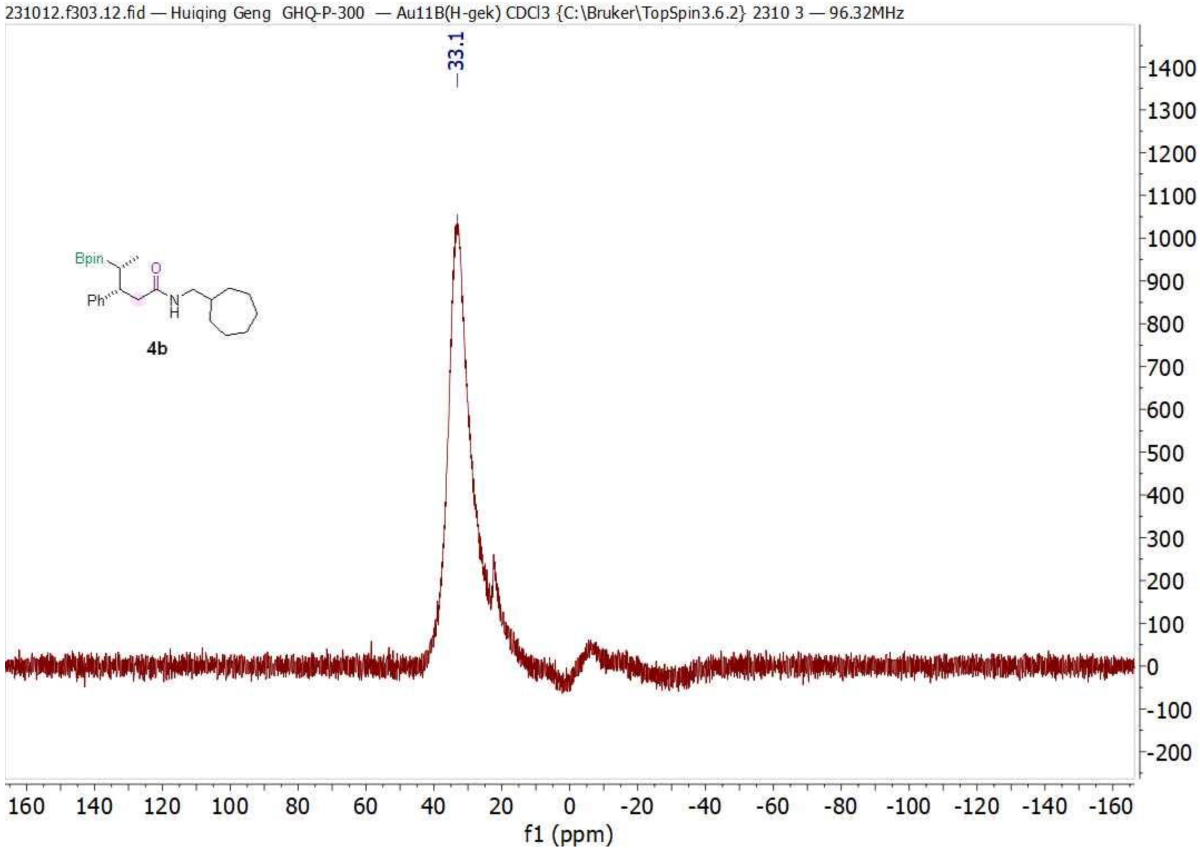

231012.f303.12.fid — Huiqing Geng GHQ-P-300 — Au11B(H-gek) CDCl3 {C:\Bruker\TopSpin3.6.2} 2310 3 — 96.32MHz
—33.1
Bpin
O
Ph
N
H
4b
160 140 120 100 80 60 40 20 0 -20 -40 -60 -80 -100 -120 -140 -160
f1 (ppm)
1400
1300
1200
1100
1000
900
800
700
600
500
400
300
200
100
0
-100
-200

231004.309.10.fid — Huiqing Geng GHQ-P-275 — Au1H CDCl3 {C:\Bruker\TopSpin3.6.2} 2310 9 — 300.13MHz

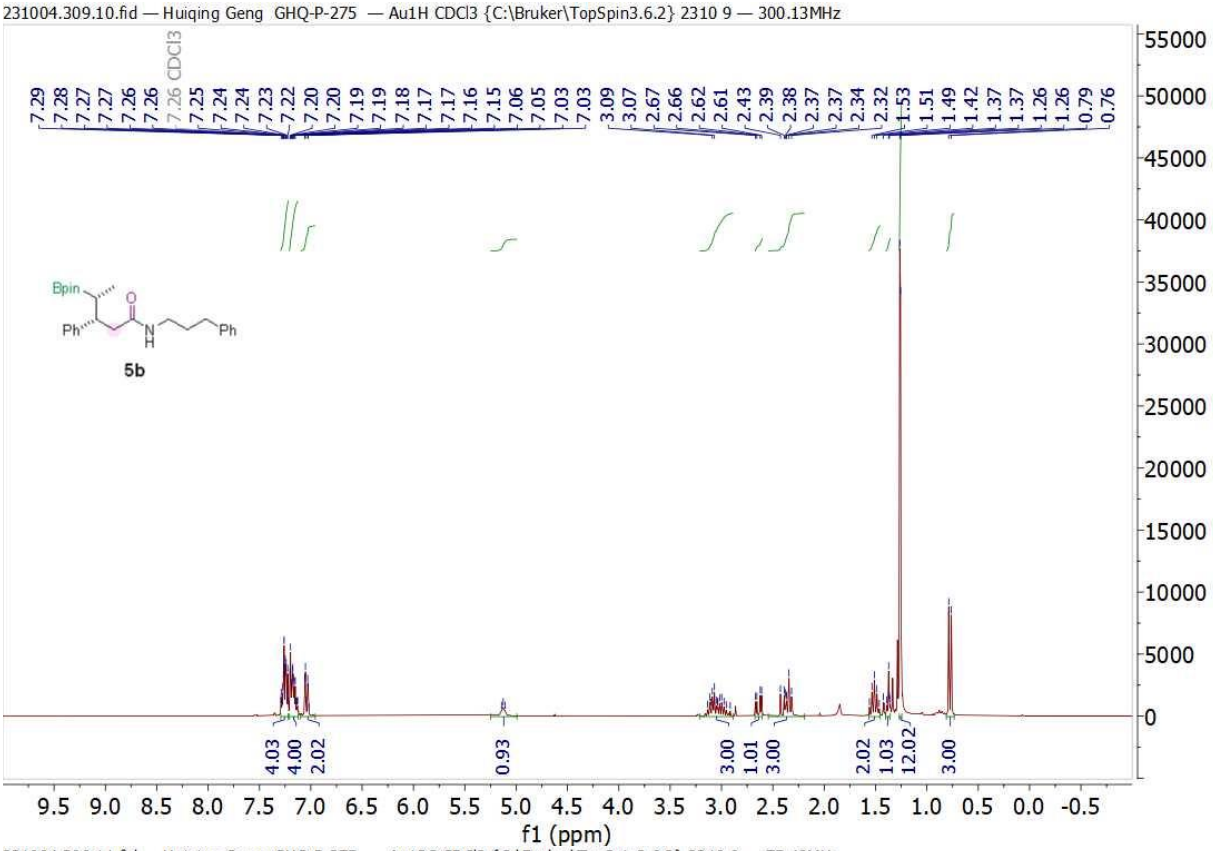

231004.309.11.fid — Huiqing Geng GHQ-P-275 — Au13C CDCl3 {C:\Bruker\TopSpin3.6.2} 2310 9 — 75.48MHz

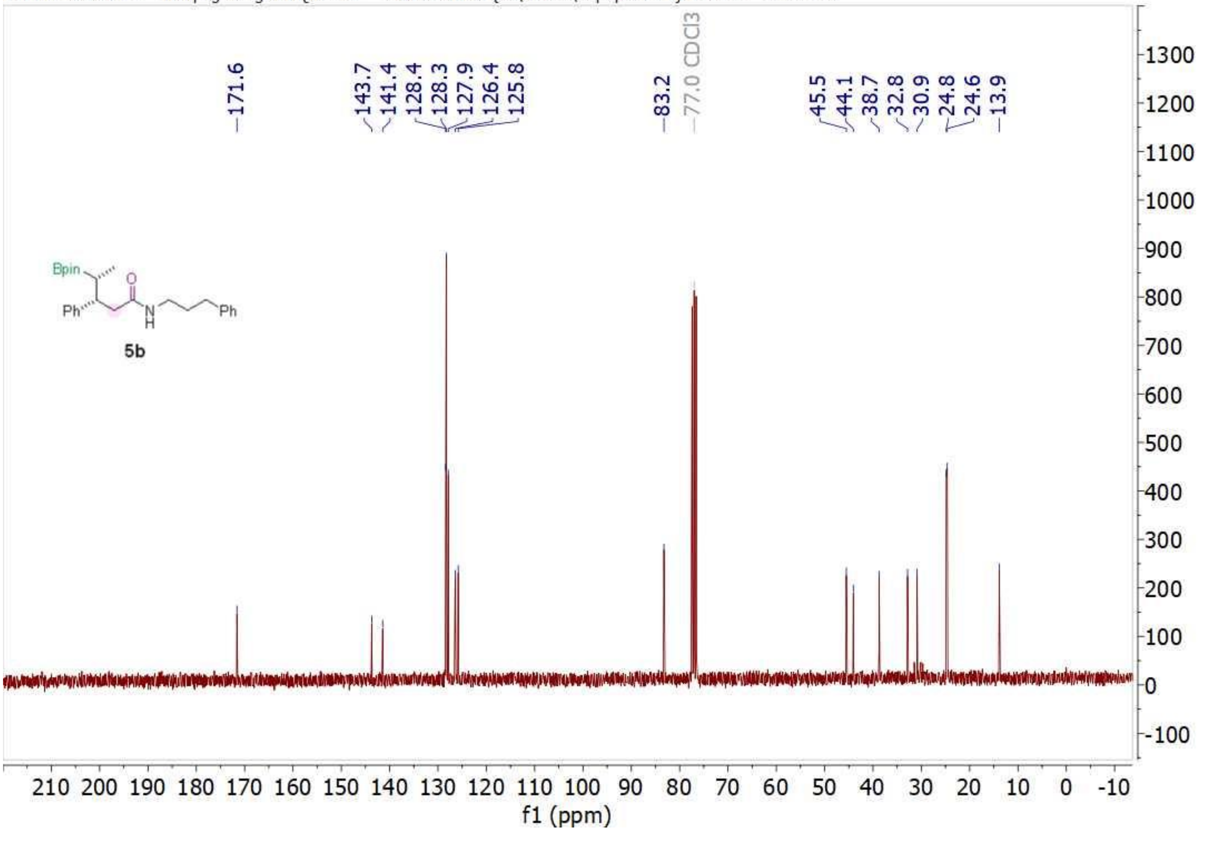

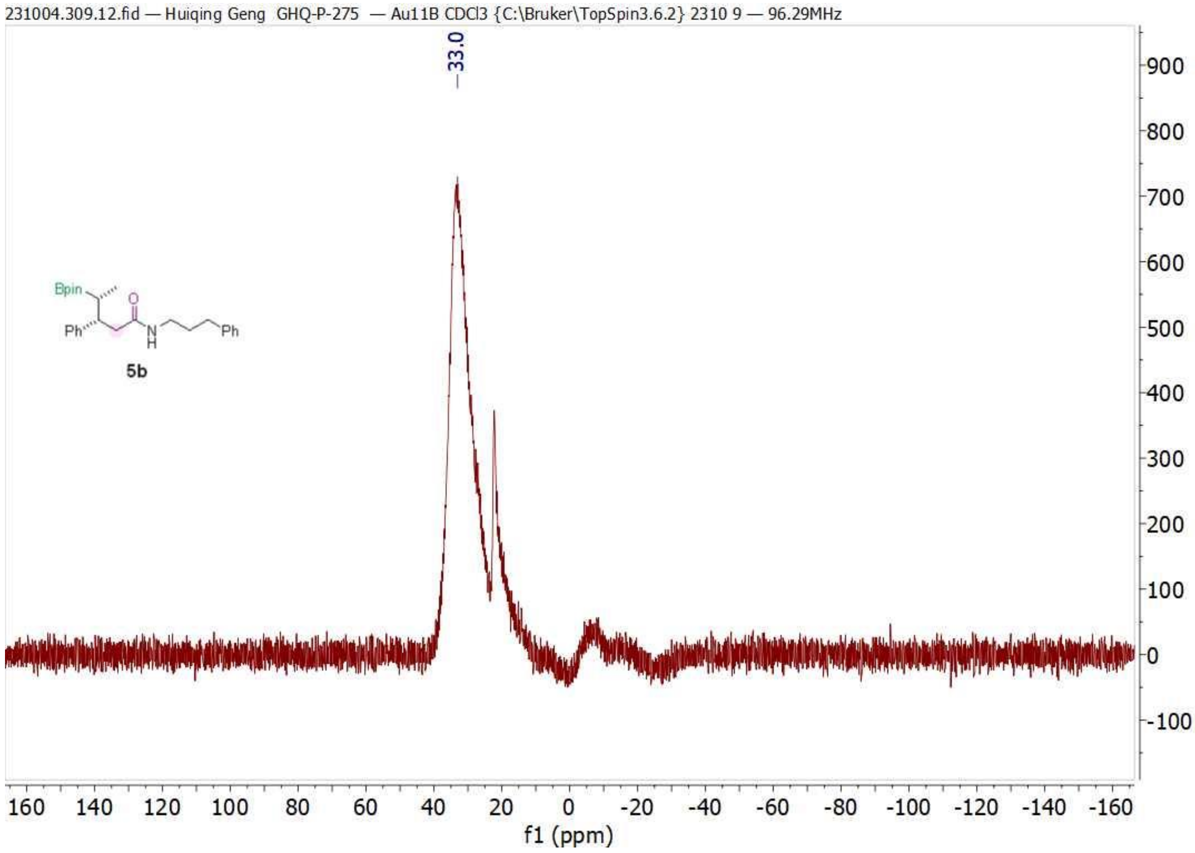

231004.309.12.fid — Huiqing Geng GHQ-P-275 — Au11B CDCl3 {C:\Bruker\TopSpin3.6.2} 2310 9 — 96.29MHz
—33.0
Bpin
O
Ph
N
H
Ph
5b
f1 (ppm)

231007.f359.10.fid — GHQ-P-288 — Au1H CDCl3 {C:\Bruker\TopSpin3.6.2} 2310 59 — 300.20MHz

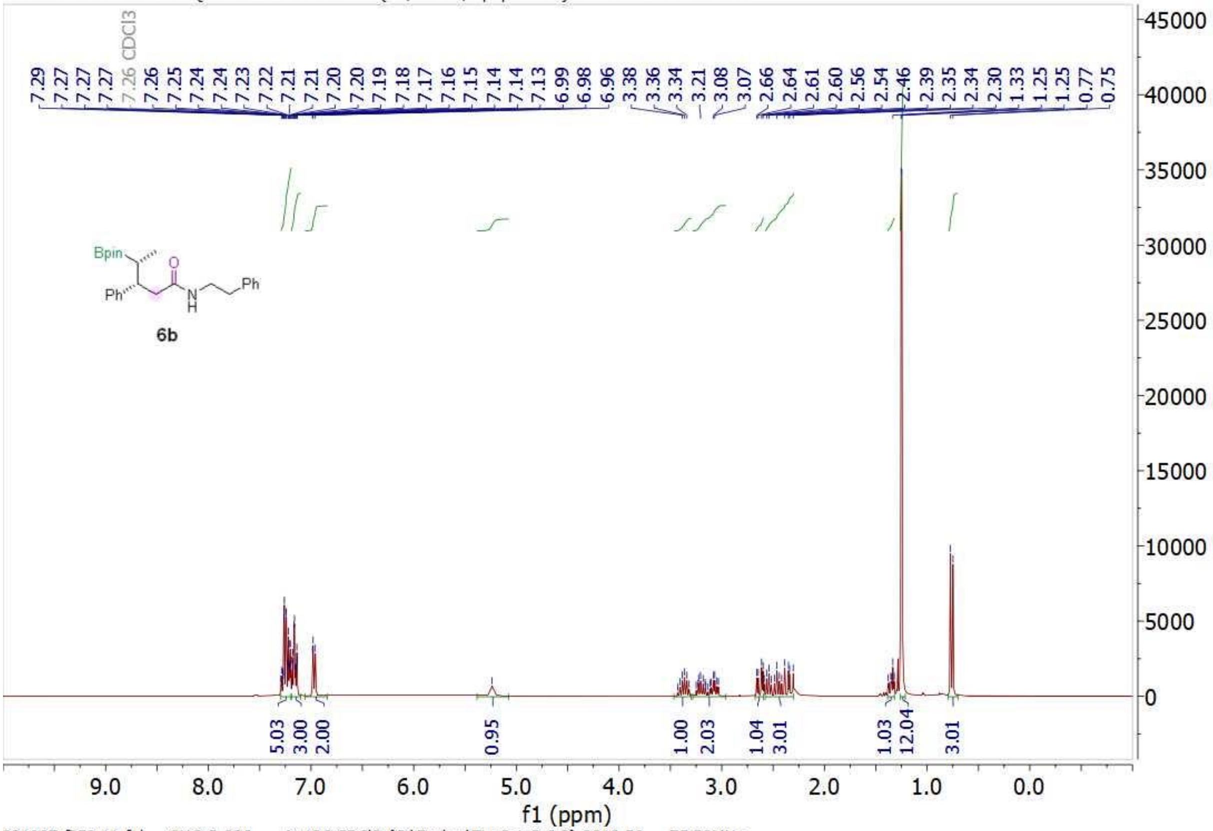

231007.f359.11.fid — GHQ-P-288 — Au13C CDCl3 {C:\Bruker\TopSpin3.6.2} 2310 59 — 75.50MHz

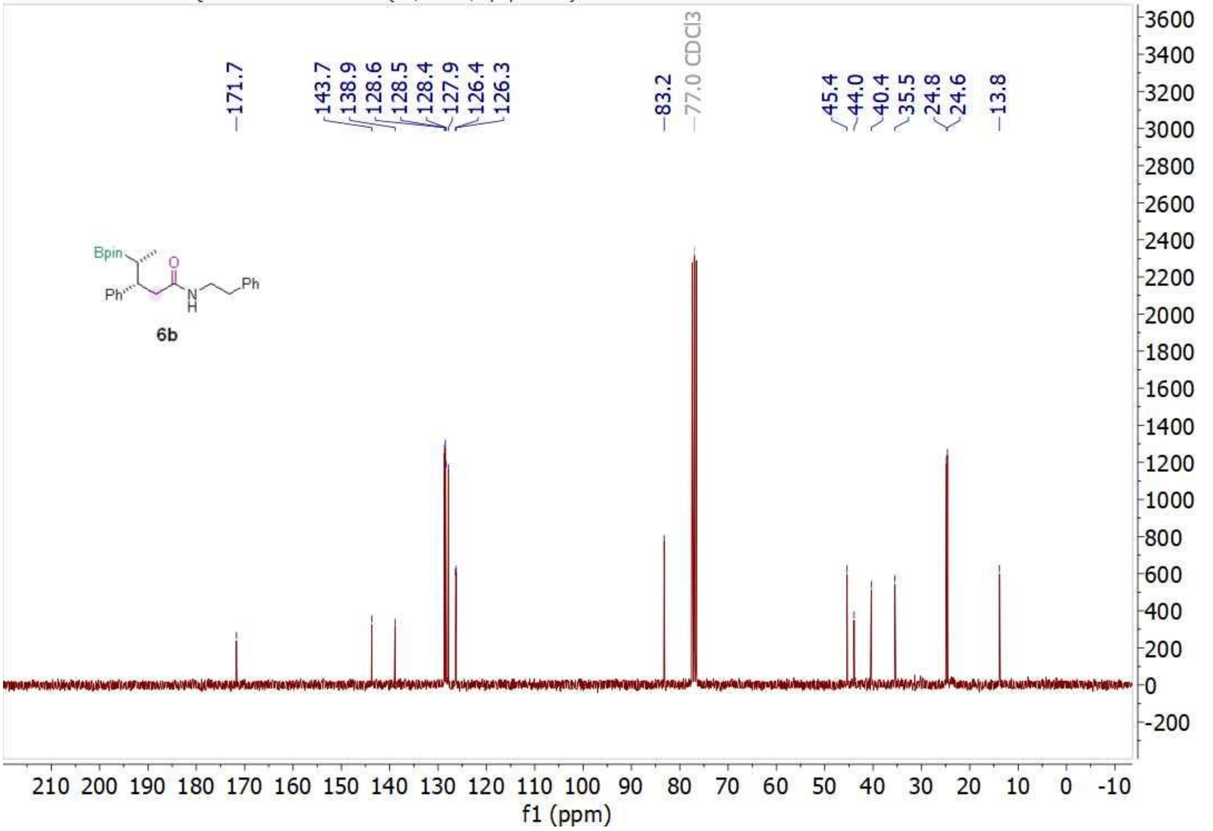

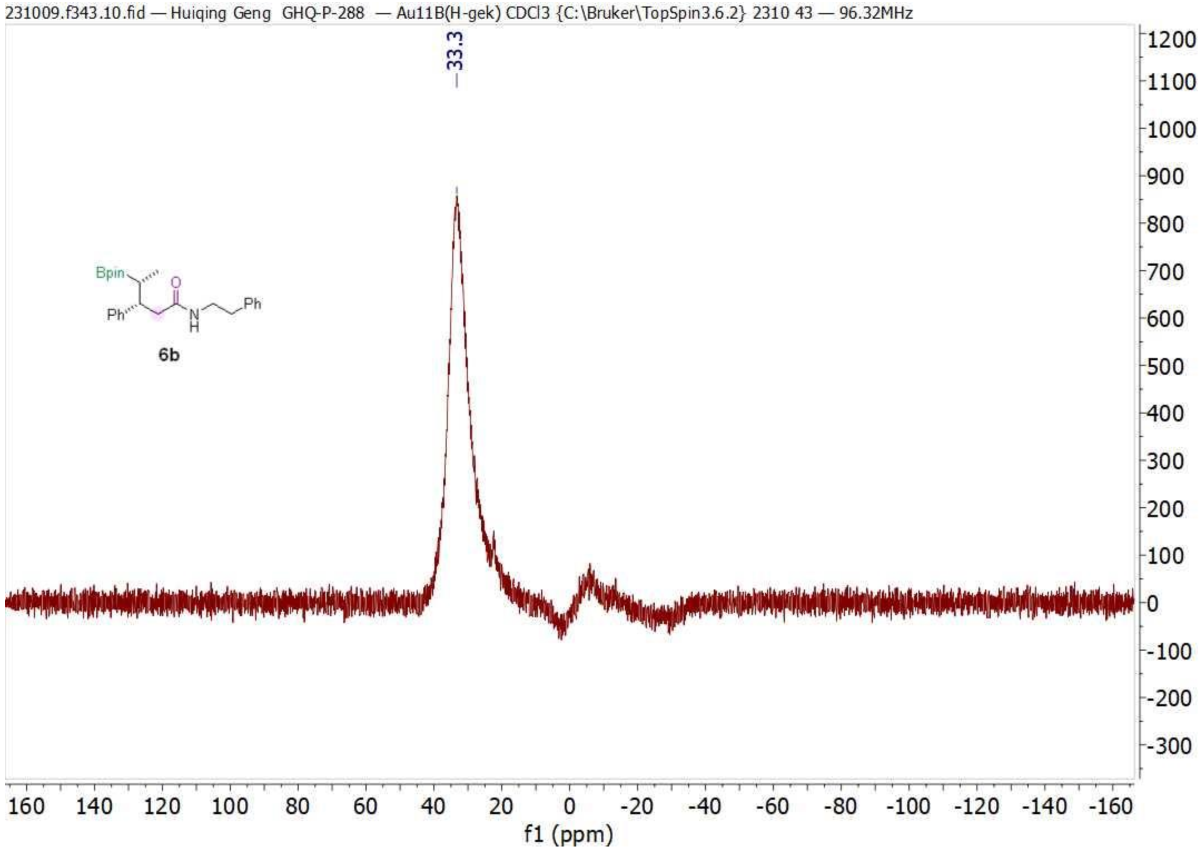
231009.f343.10.fid — Huiqing Geng GHQ-P-288 — Au11B(H-gek) CDCl3 {C:\Bruker\TopSpin3.6.2} 2310 43 — 96.32MHz
—33.3
Bpin
O
Ph
N
H
Ph
6b
f1 (ppm)

231012.f302.10.fid — Huiqing Geng GHQ-P-297 — Au1H CDCl3 {C:\Bruker\TopSpin3.6.2} 2310 2 — 300.20MHz

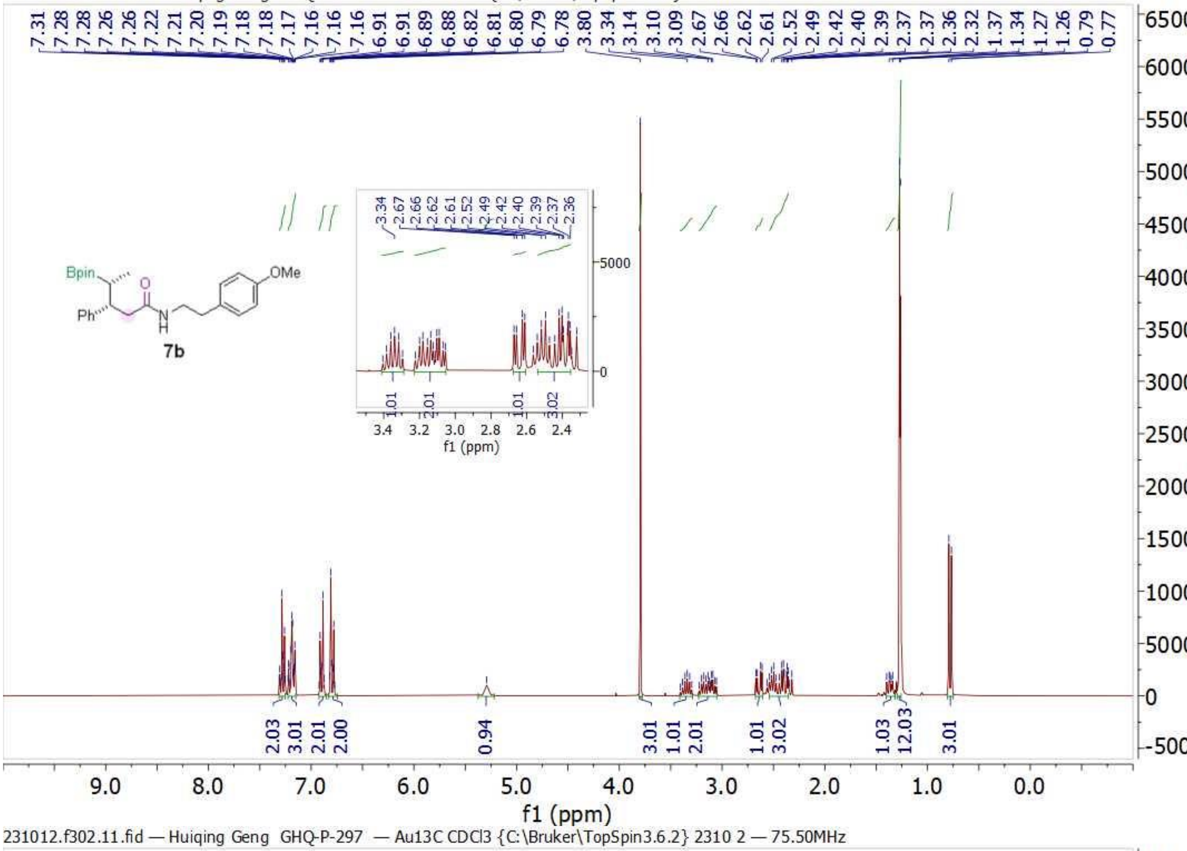

231012.f302.11.fid — Huiqing Geng GHQ-P-297 — Au13C CDCl3 {C:\Bruker\TopSpin3.6.2} 2310 2 — 75.50MHz

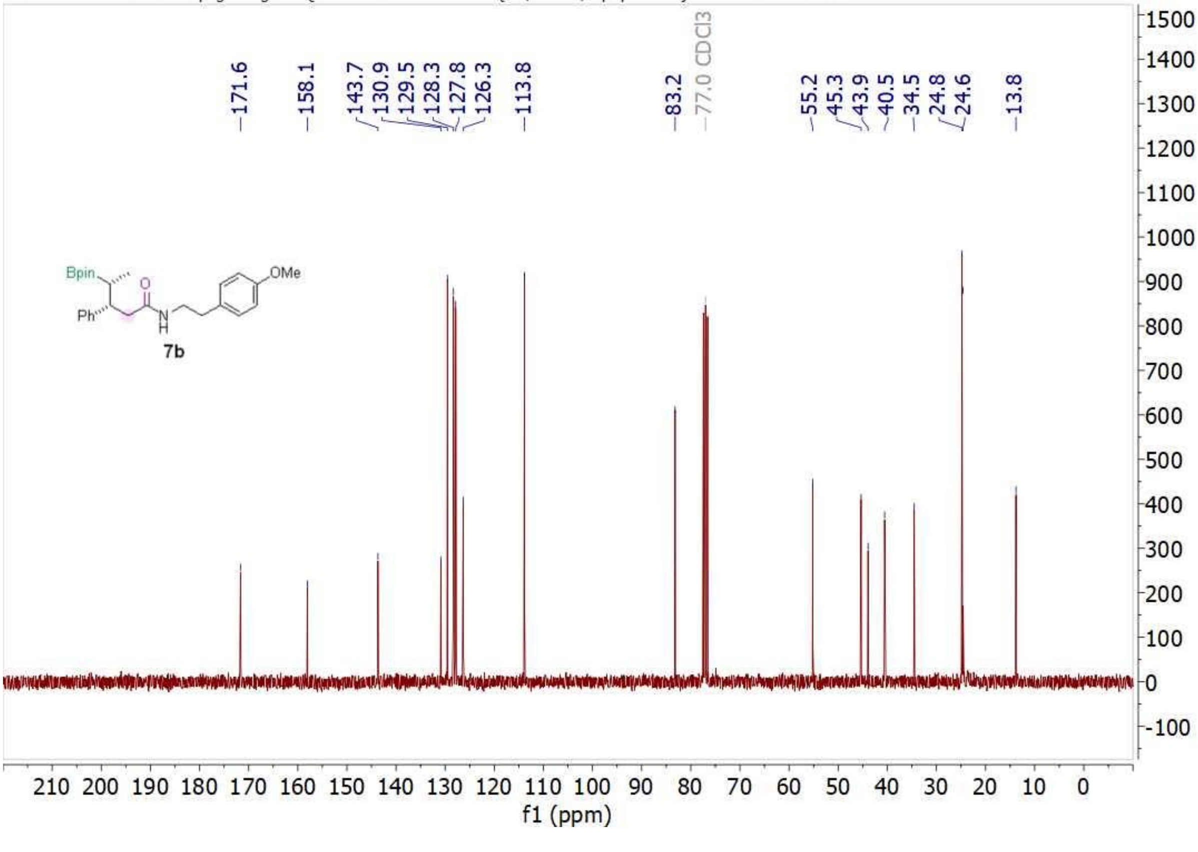

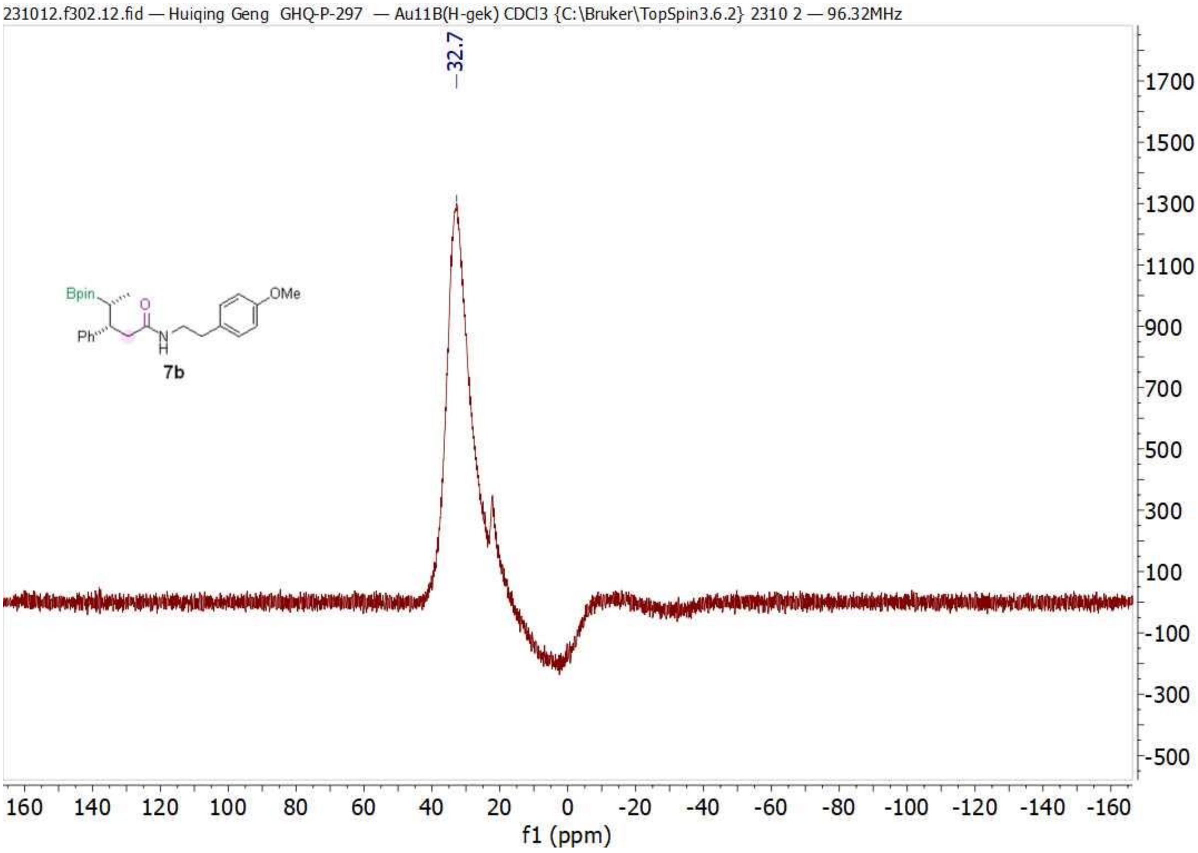
231012.f302.12.fid — Huiqing Geng GHQ-P-297 — Au11B(H-gek) CDCl3 {C:\Bruker\TopSpin3.6.2} 2310 2 — 96.32MHz
32.7
Bpin
Ph
OMe
N
H
O
7b
f1 (ppm)

231024.f314.10.fid — Huiqing Geng GHQ-P-320 — Au1H CDCl3 {C:\Bruker\TopSpin3.6.2} 2310 14 — 300.20MHz

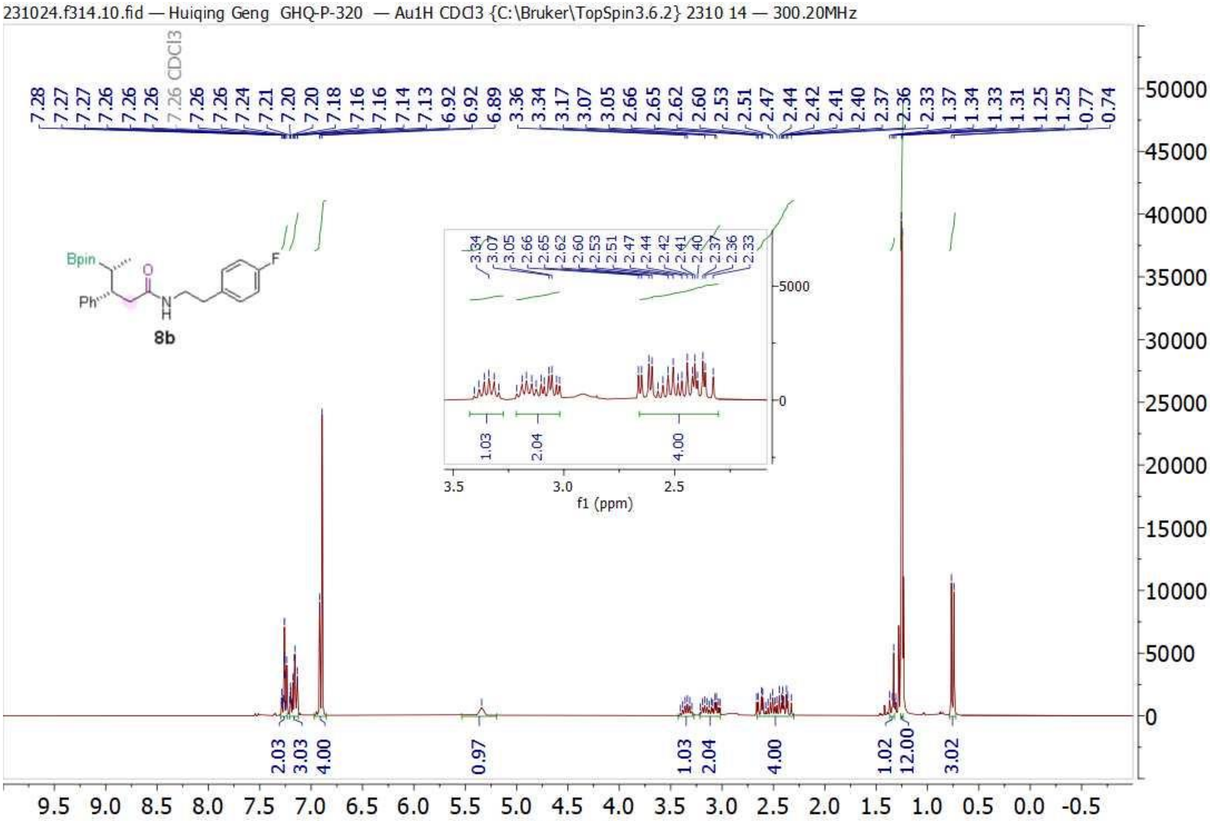

231024.f314.11.fid — Huiqing Geng GHQ-P-320 — Au13C CDCl3 {C:\Bruker\TopSpin3.6.2} 2310 14 — 75.50MHz

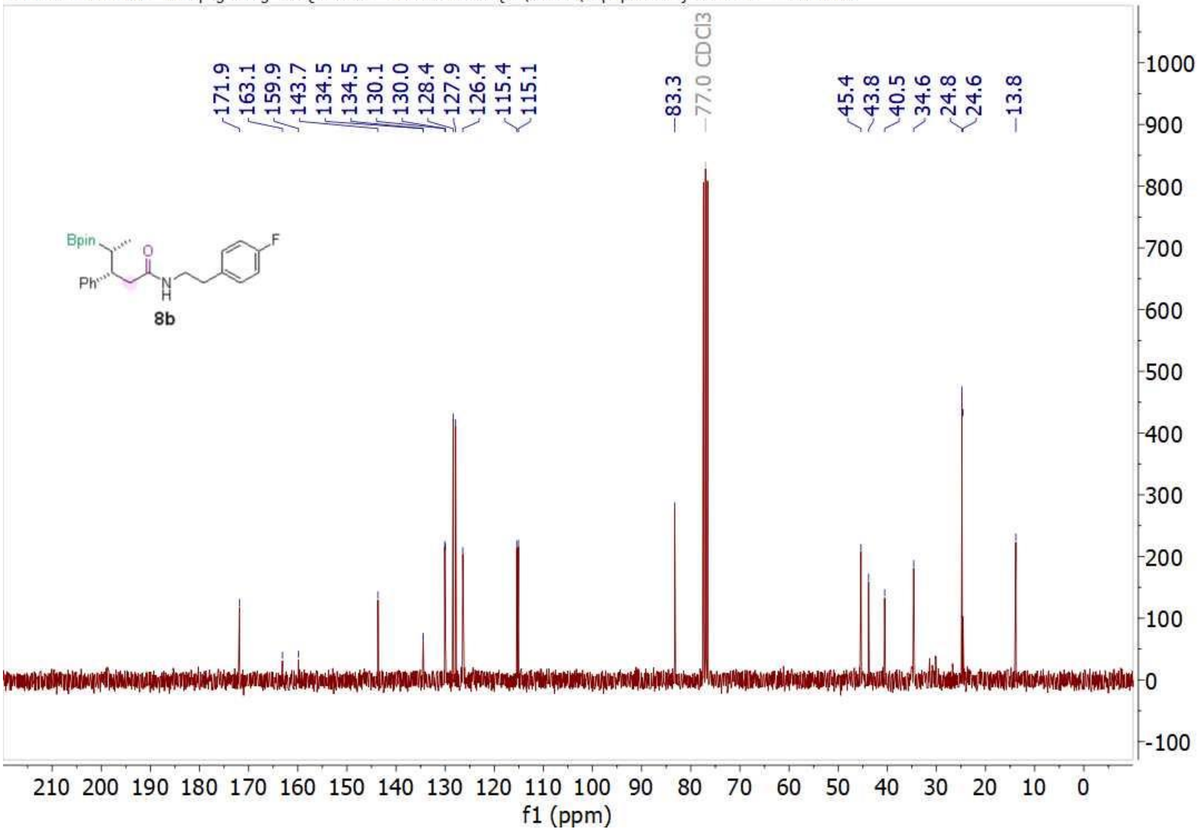

231024.f314.12.fid — Huiqing Geng GHQ-P-320 — Au11B(H-gek) CDCl3 {C:\Bruker\TopSpin3.6.2} 2310 14 — 96.32MHz

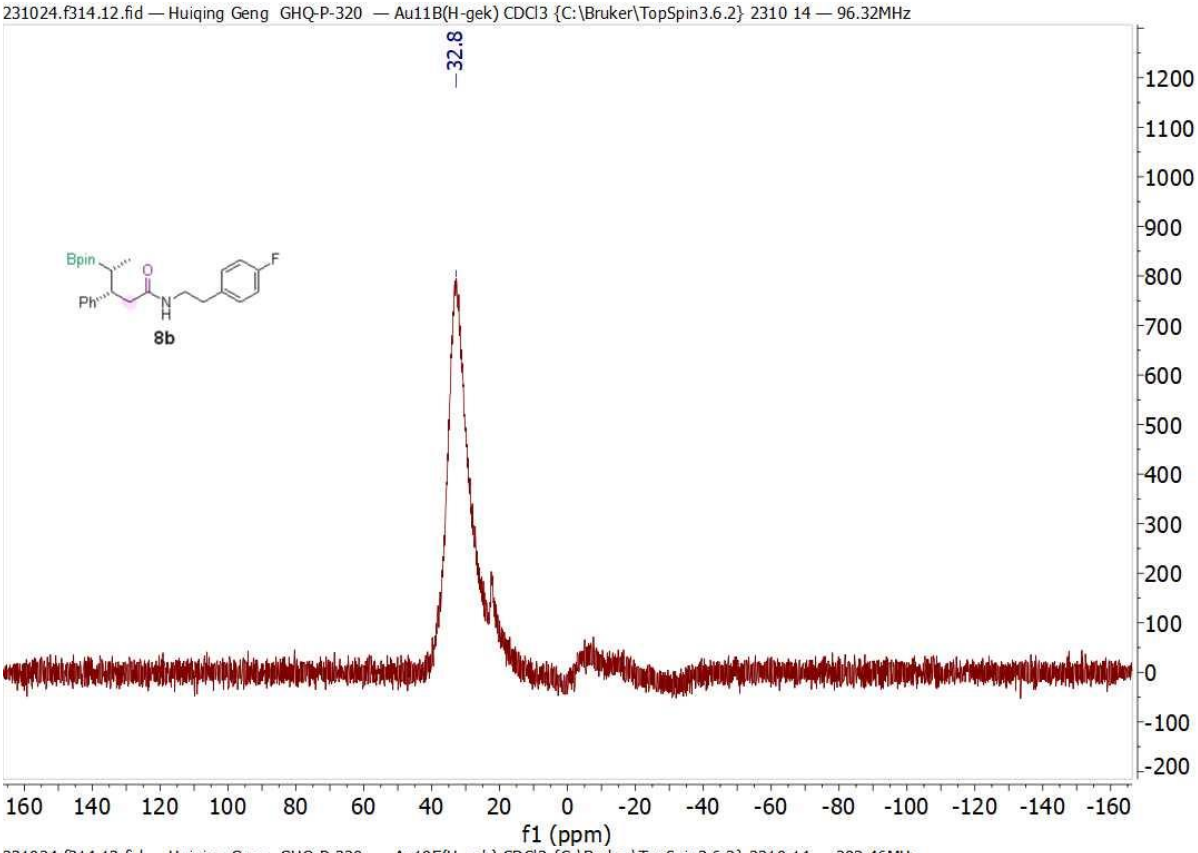

231024.f314.13.fid — Huiqing Geng GHQ-P-320 — Au19F(H-gek) CDCl3 {C:\Bruker\TopSpin3.6.2} 2310 14 — 282.46MHz

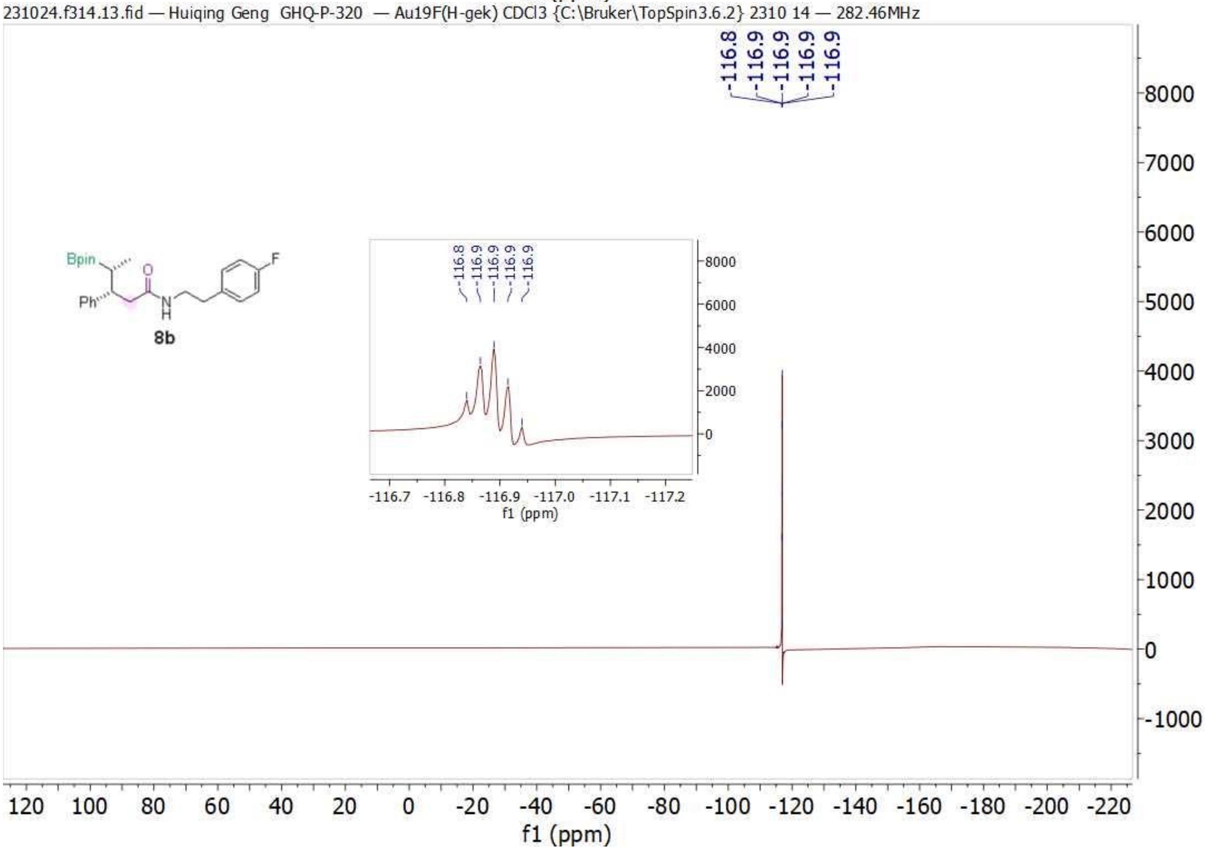

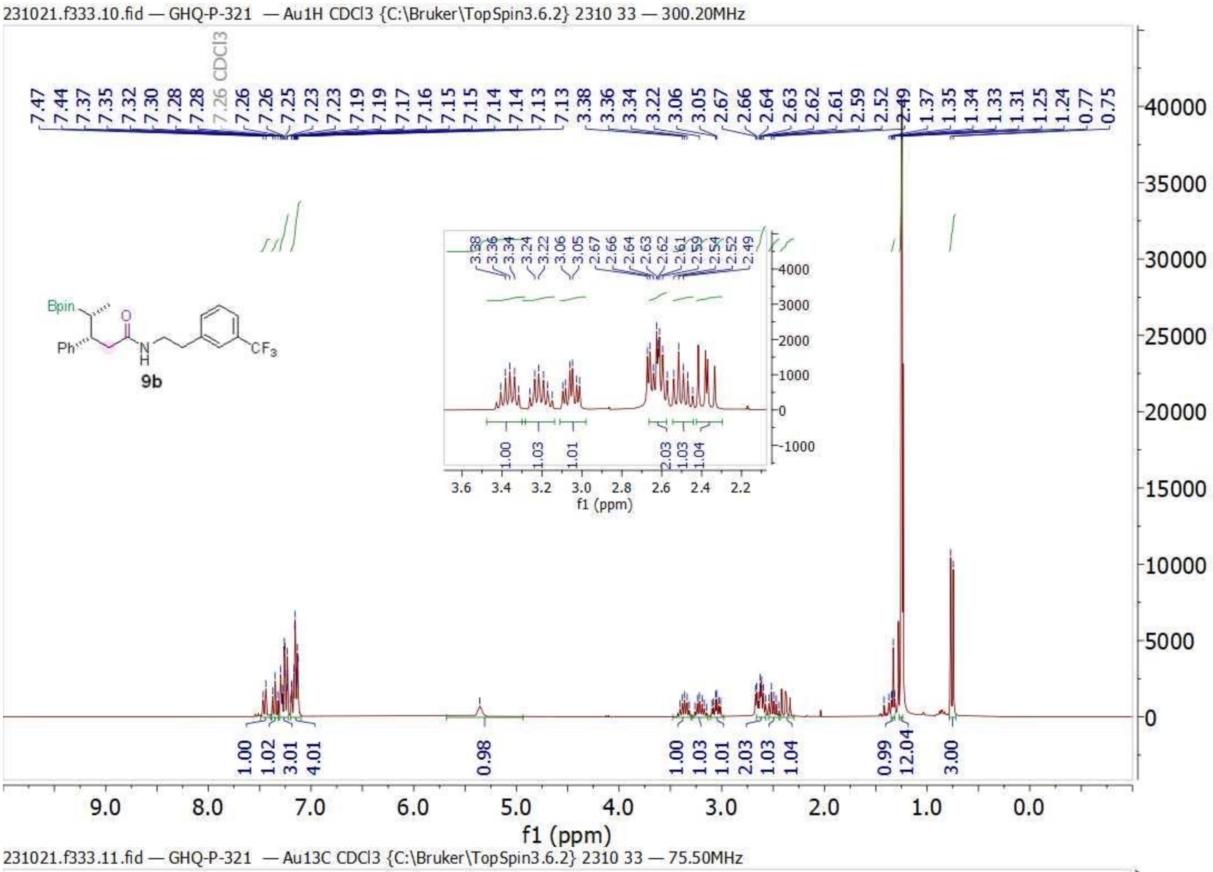
231021.f333.10.fid — GHQ-P-321 — Au1H CDCl3 {C:\Bruker\TopSpin3.6.2} 2310 33 — 300.20MHz
Bpin
Ph
CF3
9b
f1 (ppm)

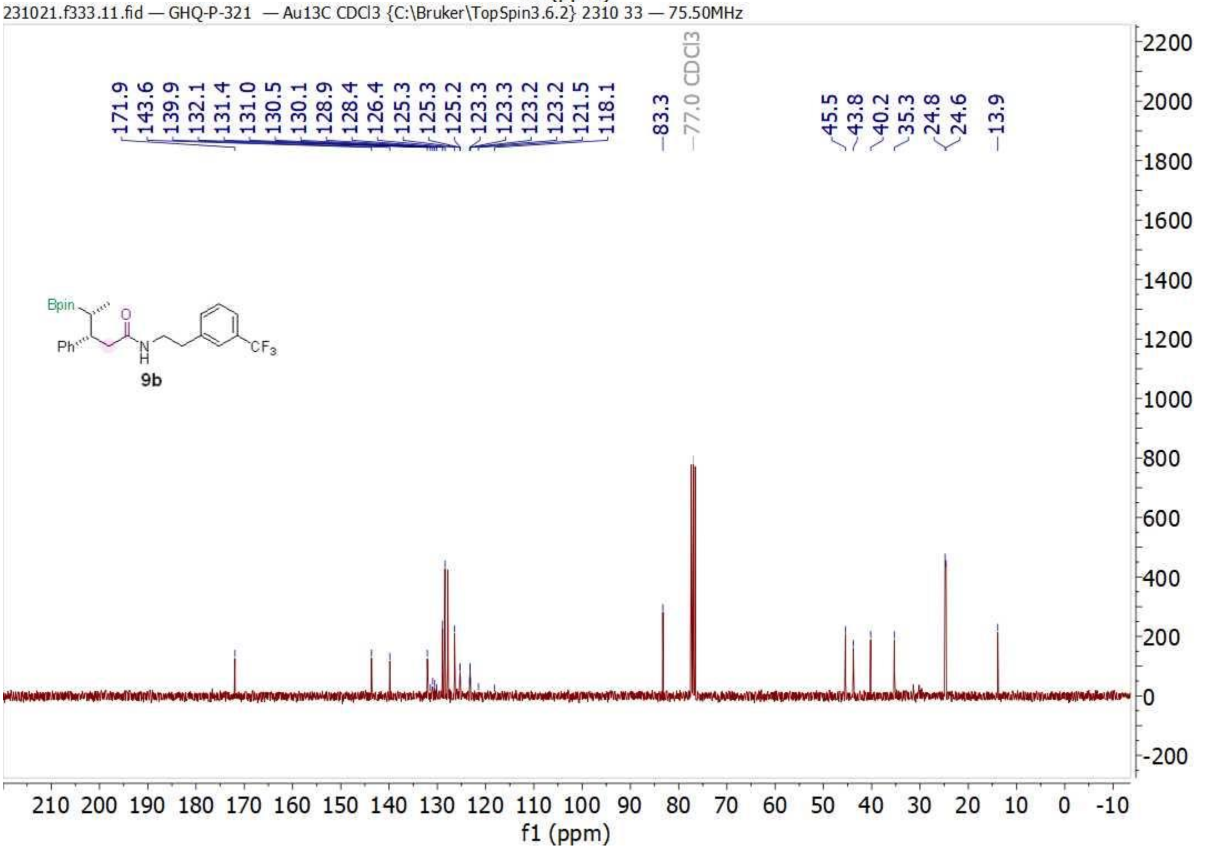
231021.f333.11.fid — GHQ-P-321 — Au13C CDCl3 {C:\Bruker\TopSpin3.6.2} 2310 33 — 75.50MHz
Bpin
Ph
CF3
9b
f1 (ppm)

231023.308.10.fid — Huiqing Geng GHQ-P-321 — Au11B CDCl3 {C:\Bruker\TopSpin3.6.2} 2310 8 — 96.29MHz

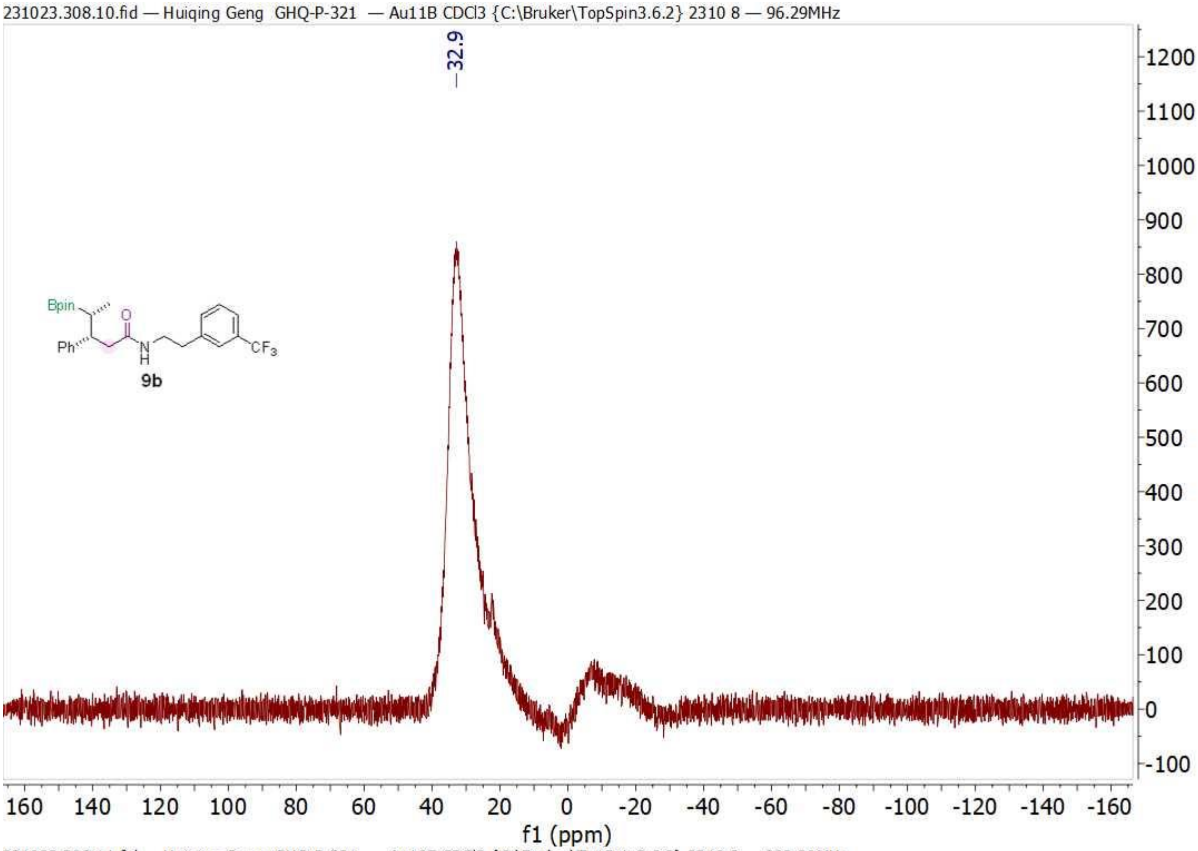

231023.308.11.fid — Huiqing Geng GHQ-P-321 — Au19F CDCl3 {C:\Bruker\TopSpin3.6.2} 2310 8 — 282.39MHz

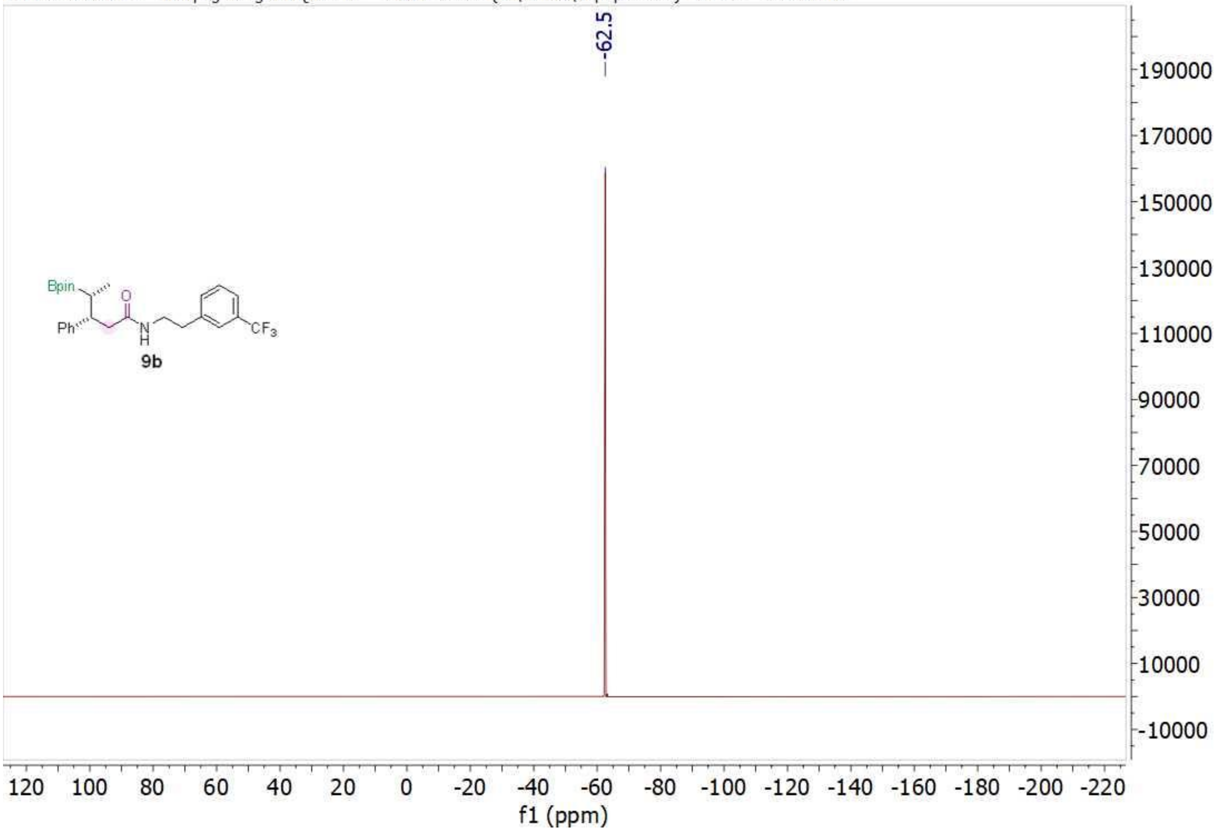

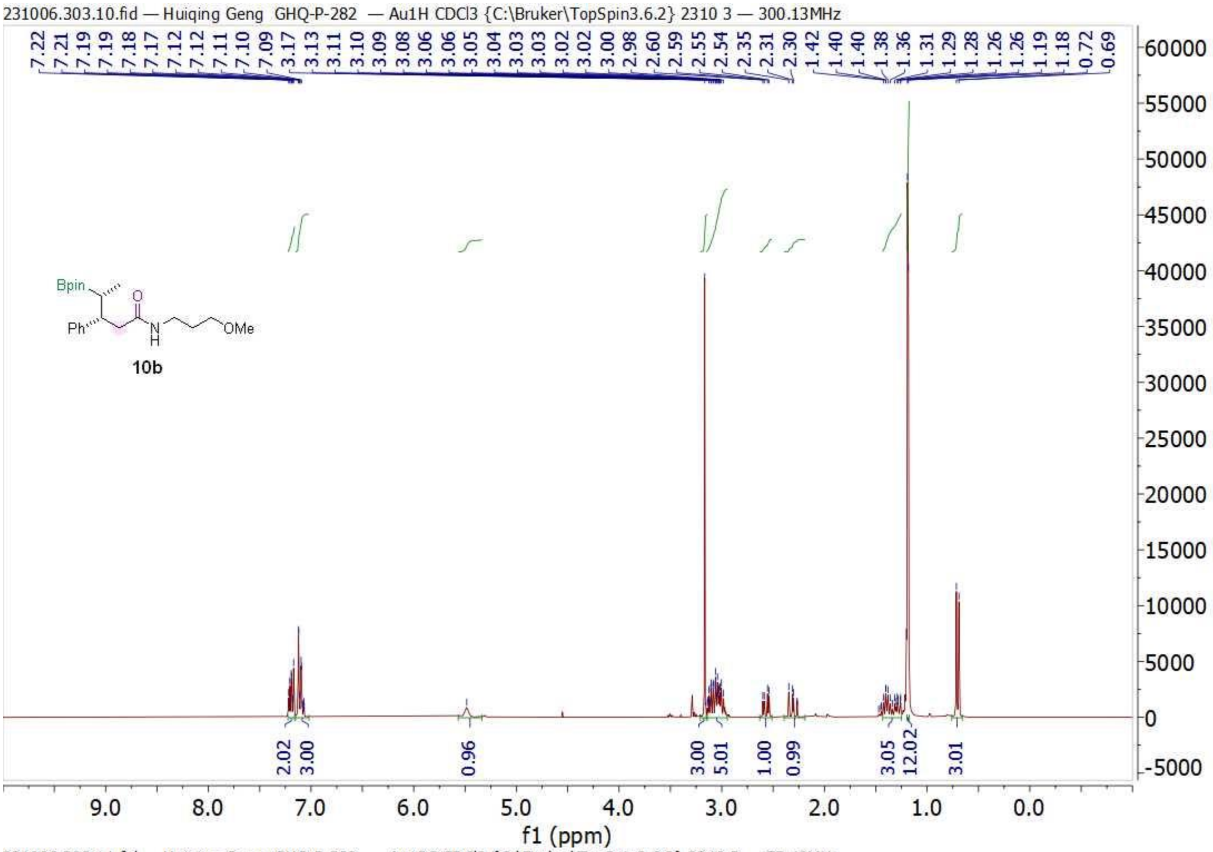
231006.303.10.fid — Huiqing Geng GHQ-P-282 — Au1H CDCl3 {C:\Bruker\TopSpin3.6.2} 2310 3 — 300.13MHz
7.22 7.21 7.19 7.19 7.18 7.17 7.12 7.12 7.11 7.10 7.09 3.17 3.13 3.11 3.10 3.09 3.08 3.06 3.06 3.05 3.04 3.03 3.03 3.02 3.00 2.98 2.60 2.59 2.55 2.54 2.35 2.31 2.30 1.42 1.40 1.40 1.38 1.36 1.31 1.29 1.28 1.26 1.26 1.19 1.18 0.72 0.69
2.02 3.00 0.96 3.00 5.01 1.00 0.99 3.05 12.02 3.01
Bpin
O
Ph
N
H
OMe
10b
f1 (ppm)

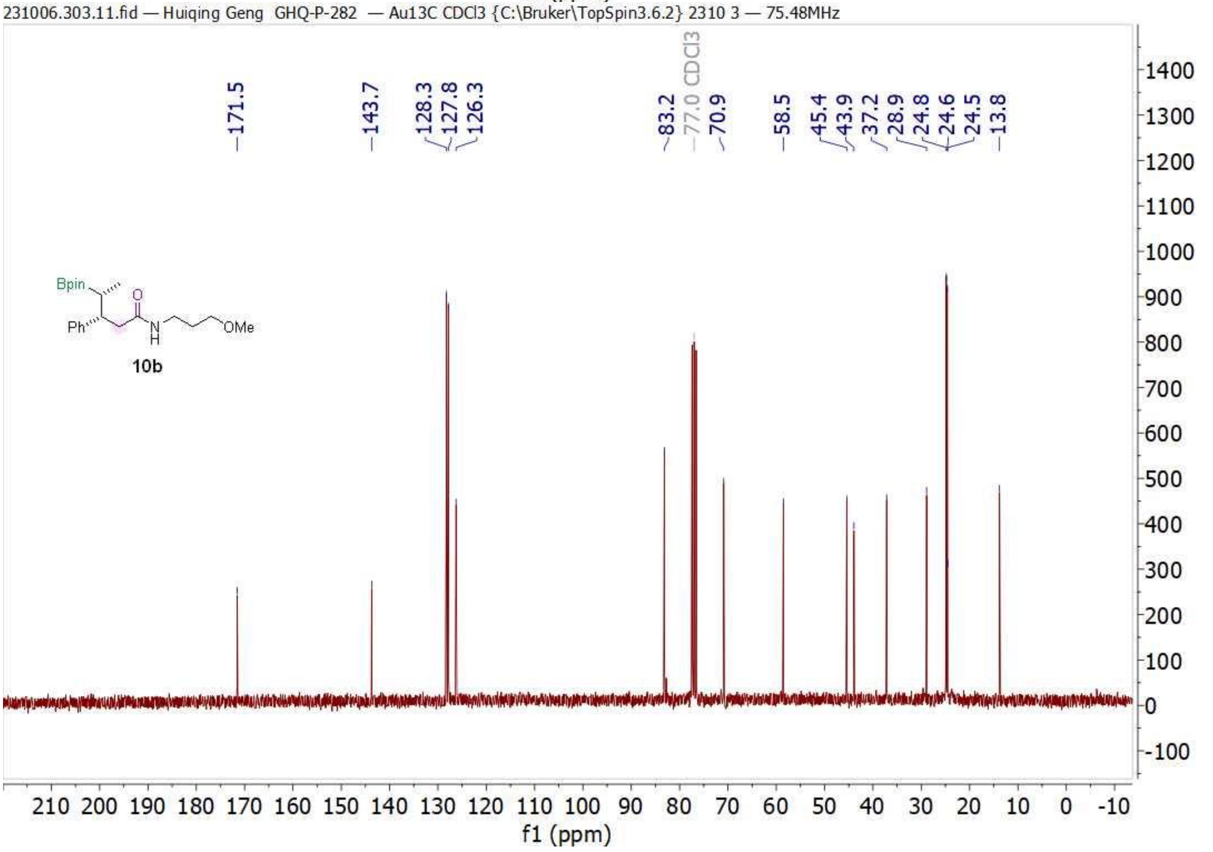
231006.303.11.fid — Huiqing Geng GHQ-P-282 — Au13C CDCl3 {C:\Bruker\TopSpin3.6.2} 2310 3 — 75.48MHz
171.5 143.7 128.3 127.8 126.3 83.2 77.0 CDCl3 70.9 58.5 45.4 43.9 37.2 28.9 24.8 24.6 24.5 13.8
Bpin
O
Ph
N
H
OMe
10b
f1 (ppm)

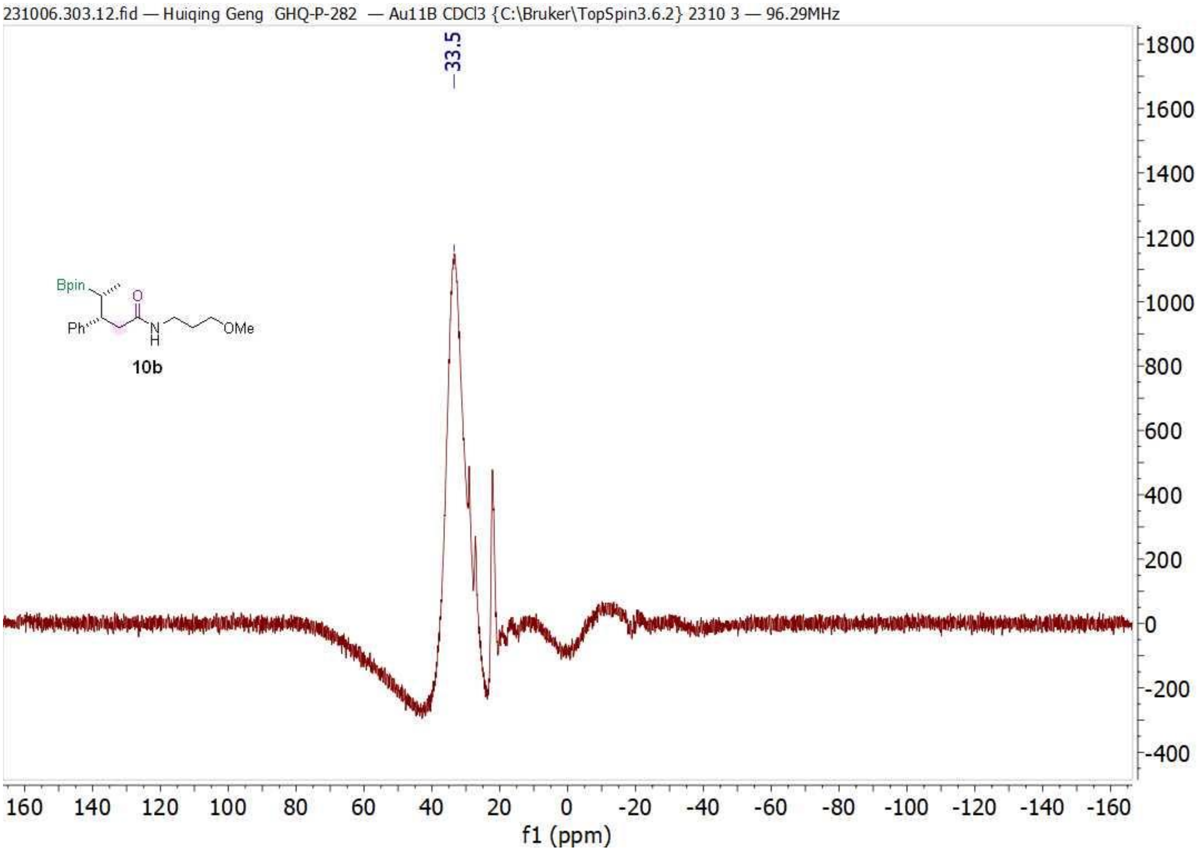
231006.303.12.fid — Huiqing Geng GHQ-P-282 — Au11B CDCl3 {C:\Bruker\TopSpin3.6.2} 2310 3 — 96.29MHz
—33.5
Bpin
Ph
OMe
10b
f1 (ppm)

231007.f360.10.fid — GHQ-P-291 — Au1H CDCl3 {C:\Bruker\TopSpin3.6.2} 2310 60 — 300.20MHz

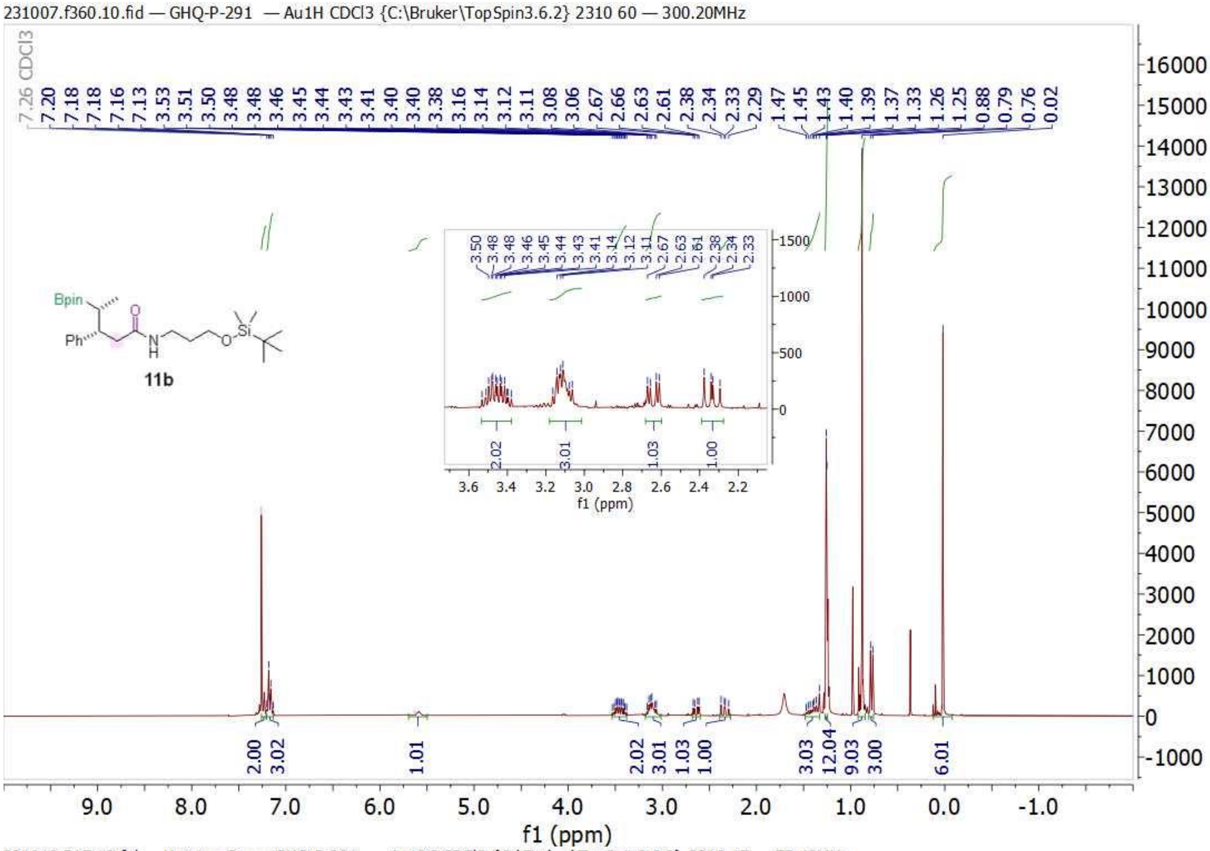

231010.317.12.fid — Huiqing Geng, GHQ-P-291 — Au13C CDCl3 {C:\Bruker\TopSpin3.6.2} 2310 17 — 75.48MHz

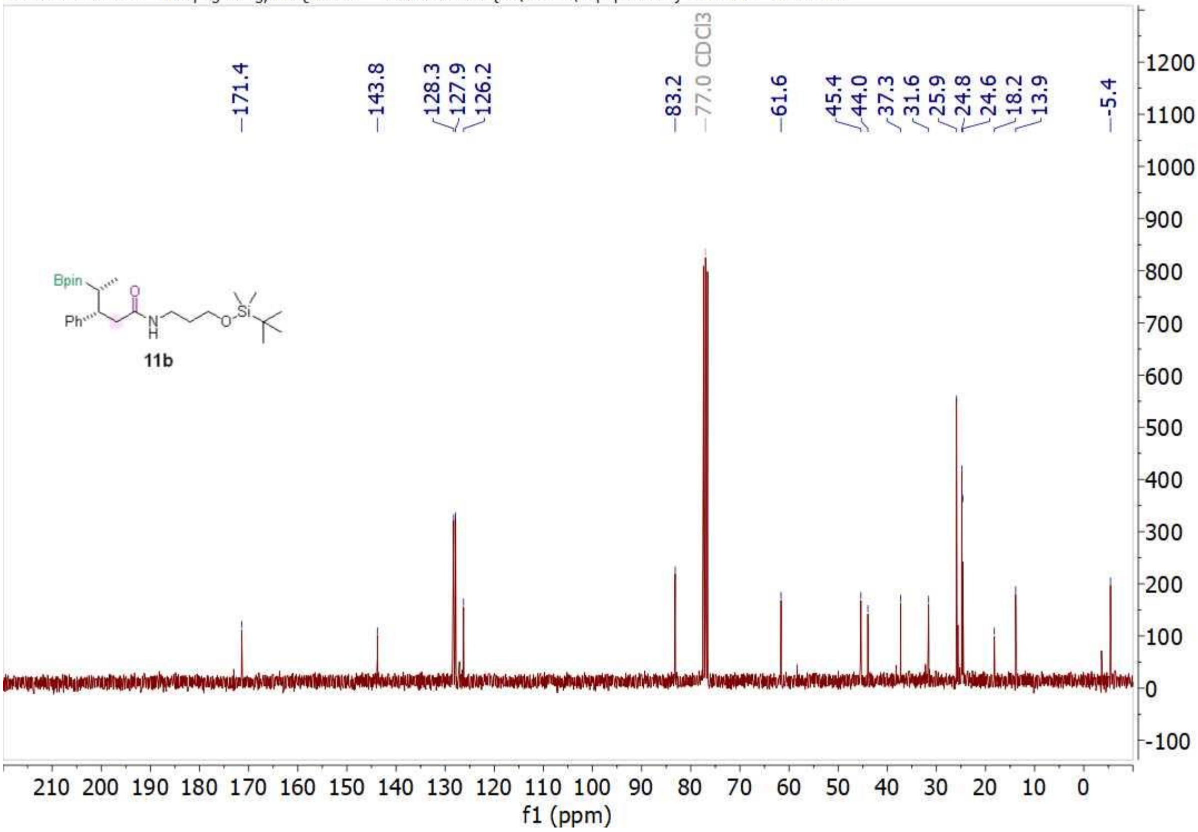

231010.317.11.fid — Huiqing Geng, GHQ-P-291 — Au11B CDCl3 {C:\Bruker\TopSpin3.6.2} 2310 17 — 96.29MHz

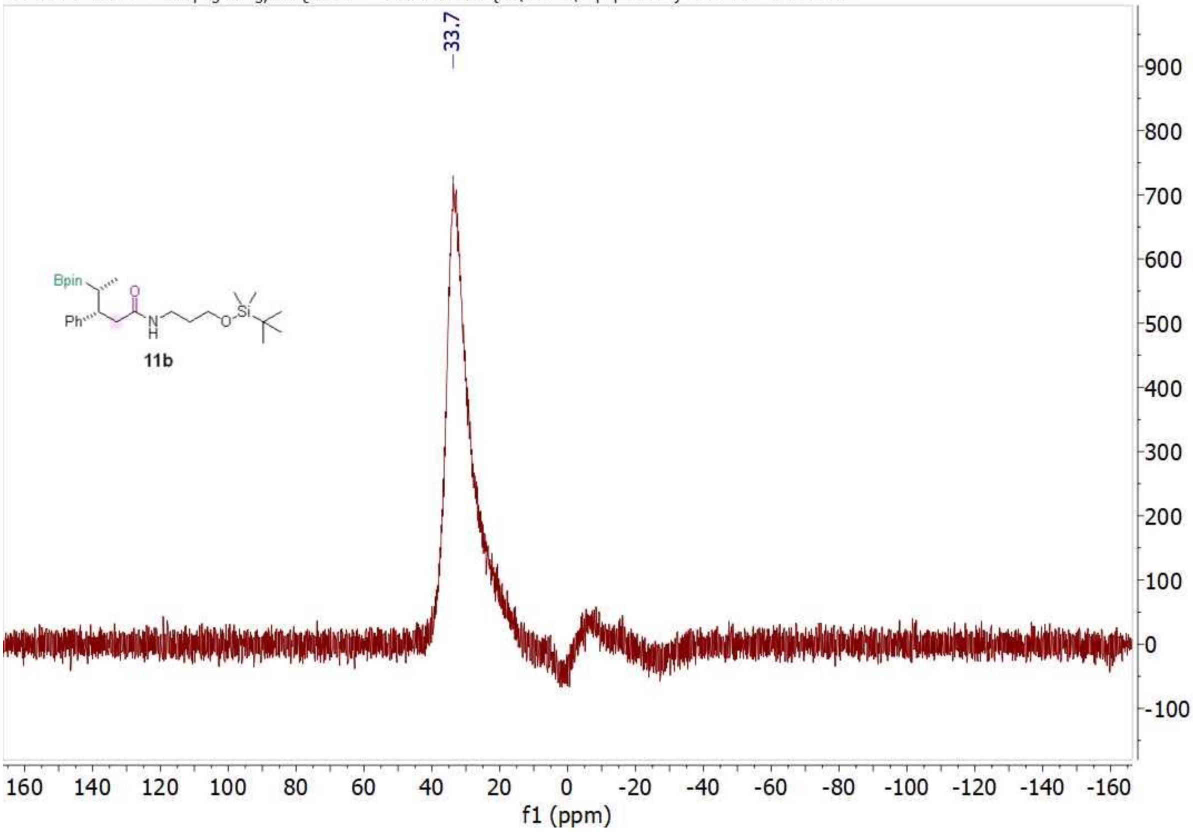

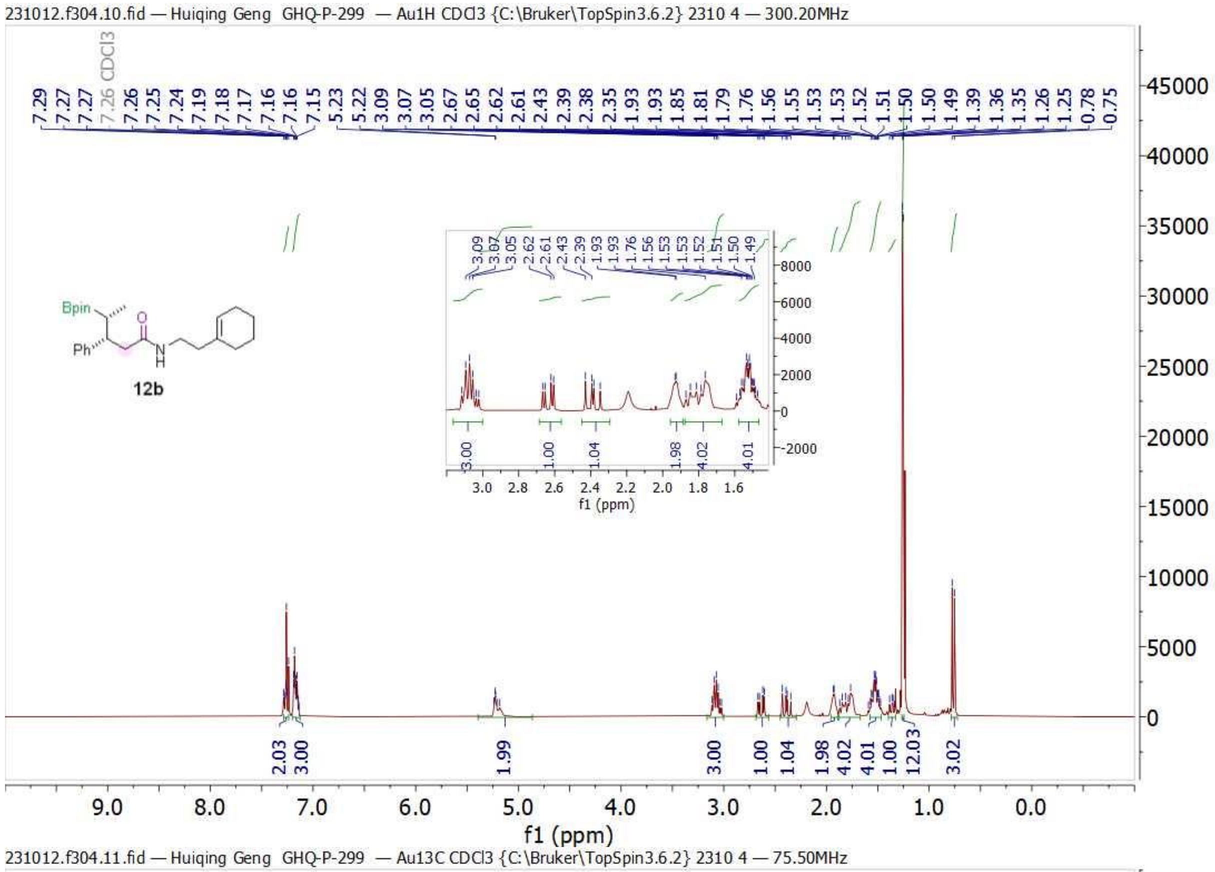
231012.f304.10.fid — Huiqing Geng GHQ-P-299 — Au1H CDCl3 {C:\Bruker\TopSpin3.6.2} 2310 4 — 300.20MHz
7.29 7.27 7.27 7.26 CDCl3 7.26 7.25 7.24 7.19 7.18 7.17 7.16 7.16 7.15 5.23 5.22 3.09 3.07 3.05 2.67 2.65 2.62 2.61 2.43 2.39 2.38 2.35 1.93 1.93 1.85 1.81 1.79 1.76 1.56 1.55 1.53 1.53 1.52 1.51 1.50 1.50 1.49 1.39 1.36 1.35 1.26 1.25 0.78 0.75
Bpin
Ph
12b
2.03 3.00 1.99 3.00 1.00 1.04 1.98 4.02 4.01 1.00 12.03 3.02
f1 (ppm)

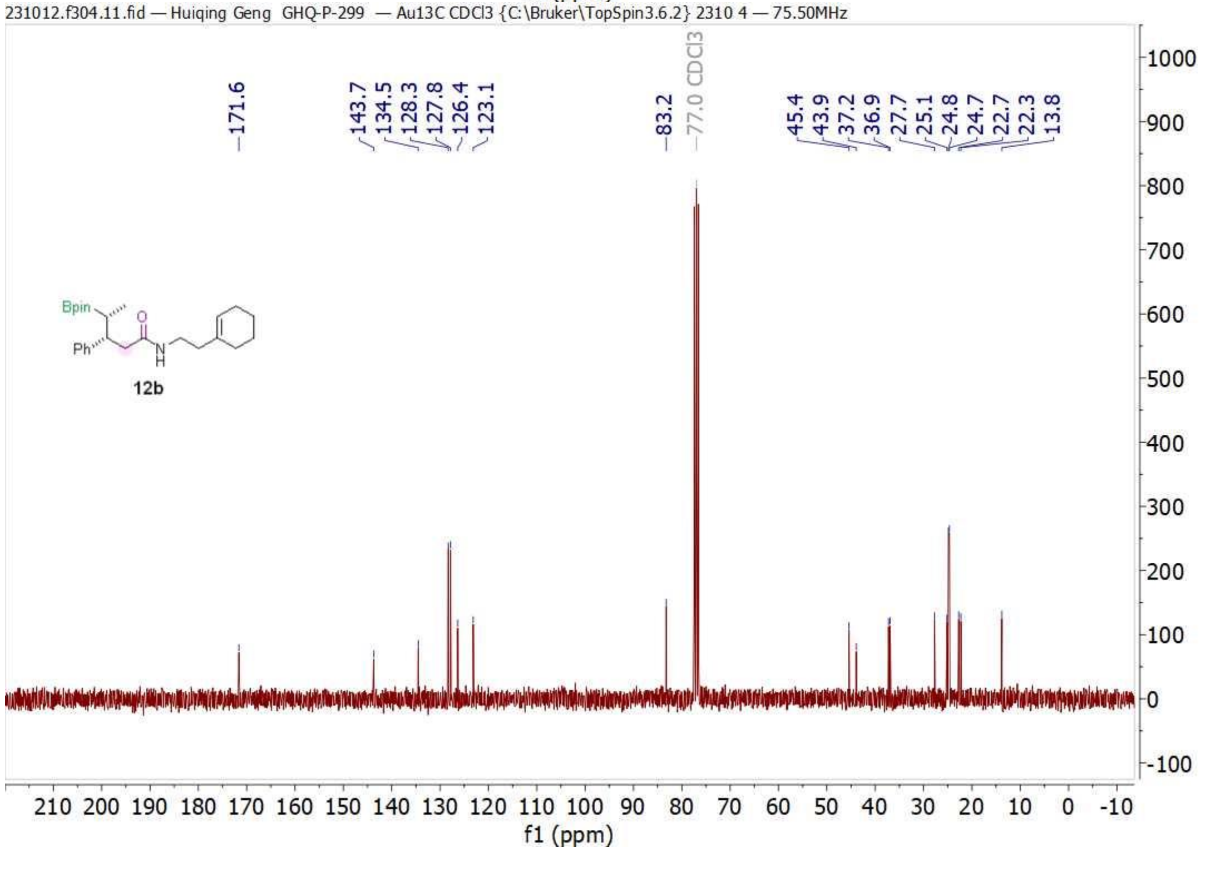
231012.f304.11.fid — Huiqing Geng GHQ-P-299 — Au13C CDCl3 {C:\Bruker\TopSpin3.6.2} 2310 4 — 75.50MHz
171.6 143.7 134.5 128.3 127.8 126.4 123.1 83.2 77.0 CDCl3 45.4 43.9 37.2 36.9 27.7 25.1 24.8 24.7 22.7 22.3 13.8
Bpin
Ph
12b
f1 (ppm)

231012.f304.12.fid — Huiqing Geng GHQ-P-299 — Au11B(H-gek) CDCl3 {C:\Bruker\TopSpin3.6.2} 2310 4 — 96.32MHz

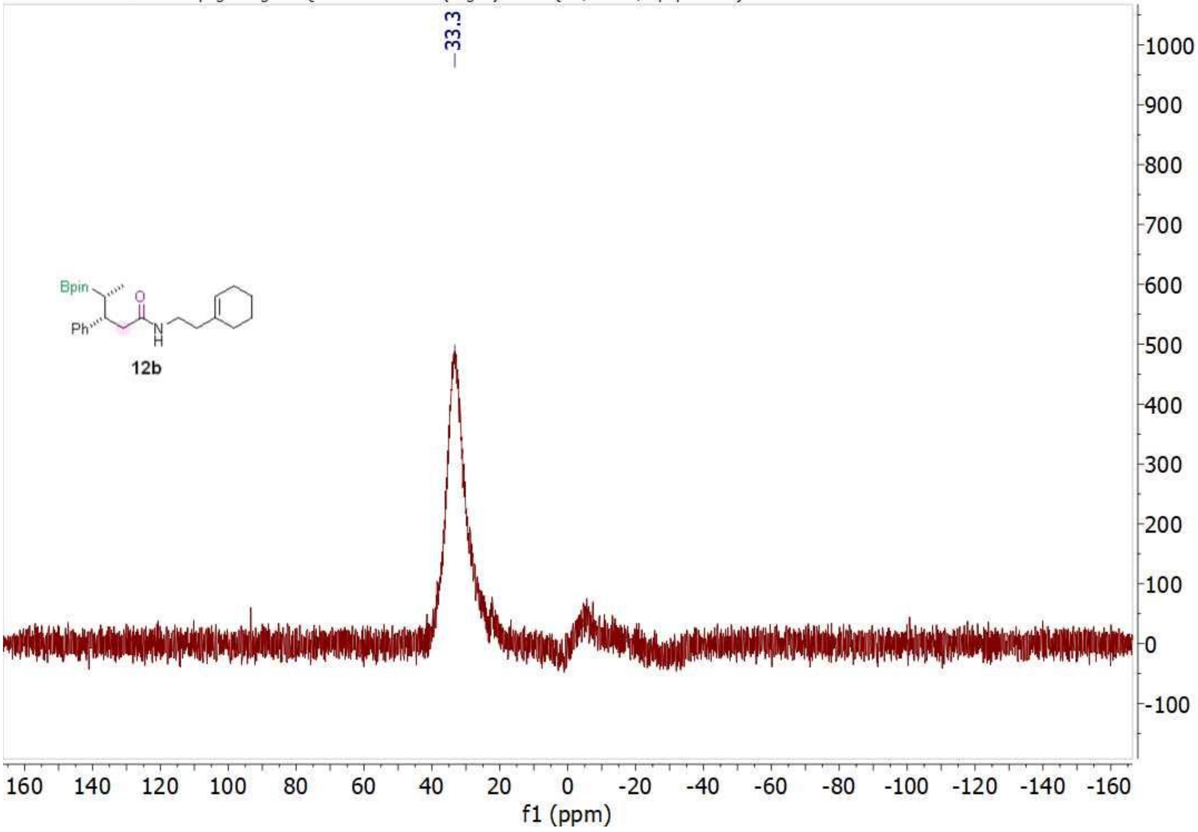

231103.f323.11.fid — Huiqing Geng GHQ-P-344 — Au1H CDCl3 {C:\Bruker\TopSpin3.6.2} 2311 23 — 300.20MHz

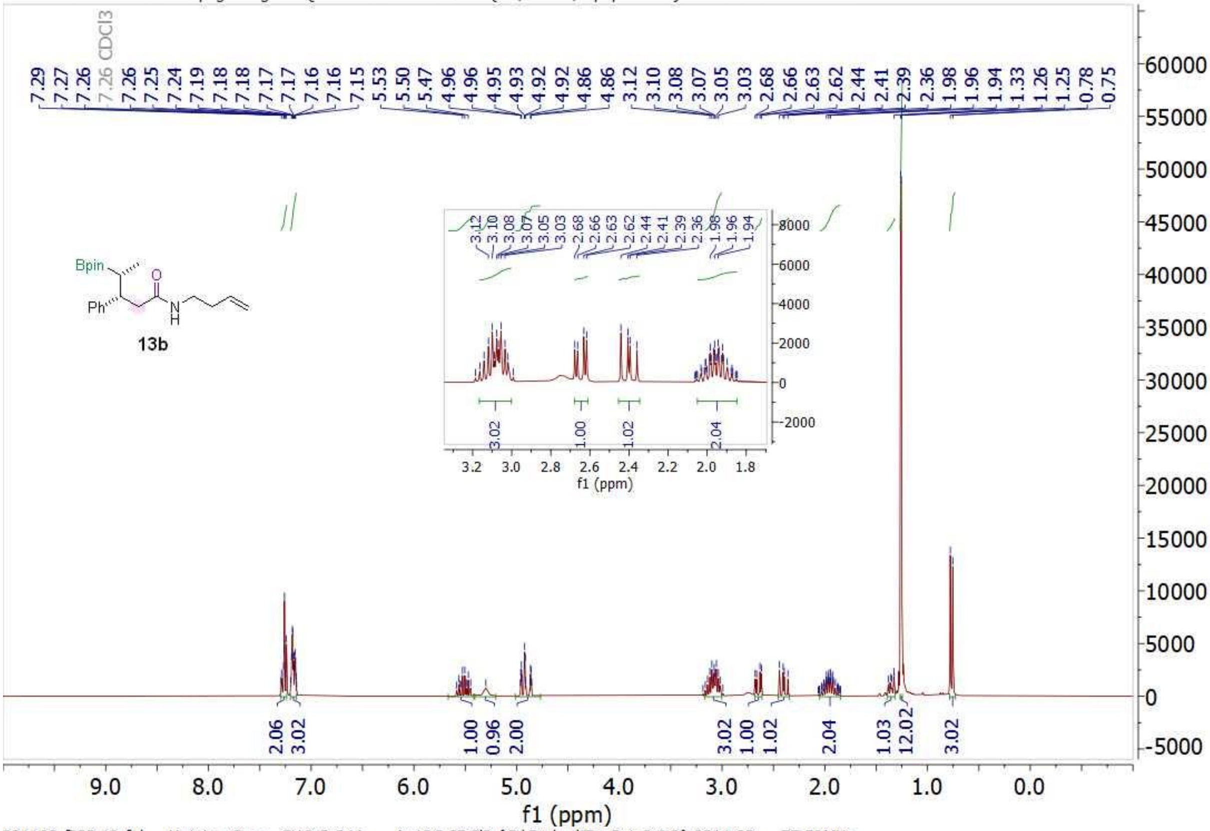

231103.f323.12.fid — Huiqing Geng GHQ-P-344 — Au13C CDCl3 {C:\Bruker\TopSpin3.6.2} 2311 23 — 75.50MHz

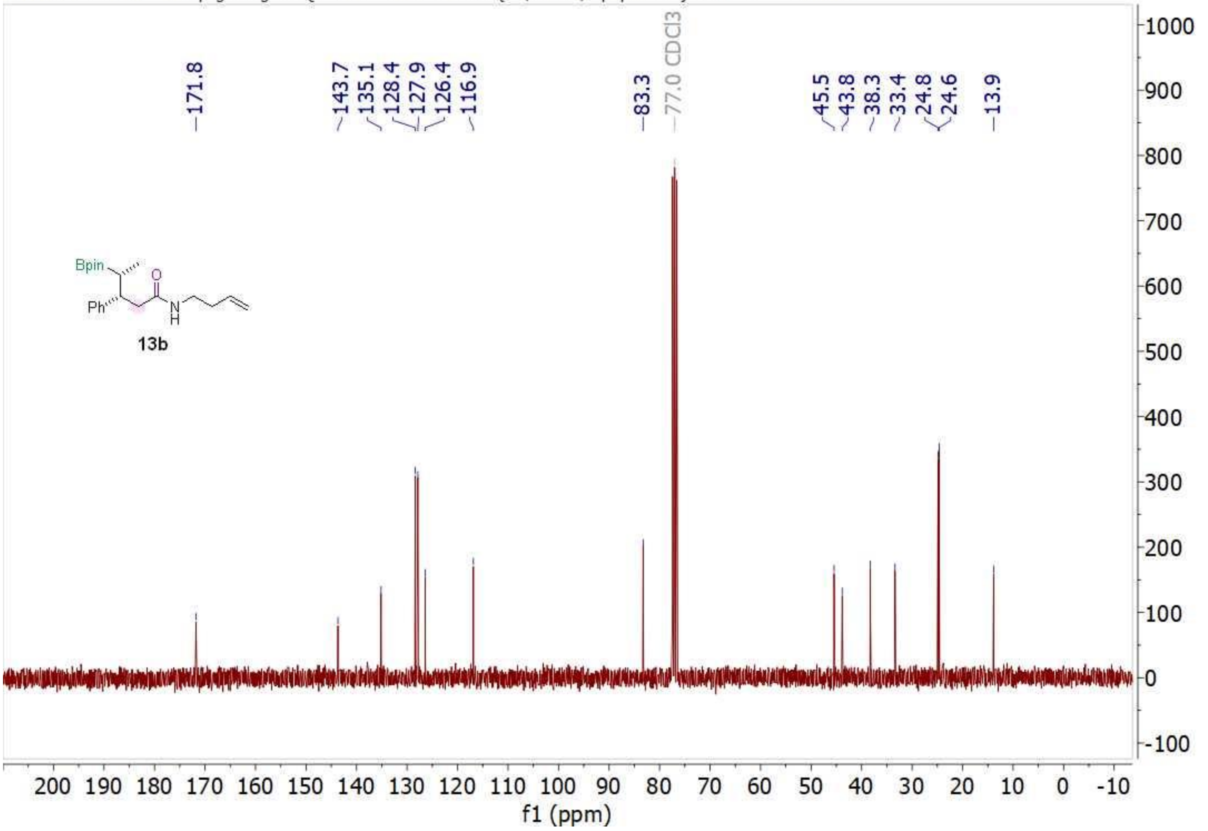

231103.f323.10.fid — Huiqing Geng GHQ-P-344 — Au11B(H-entk) CDCl3 {C:\Bruker\TopSpin3.6.2} 2311 23 — 96.32MHz

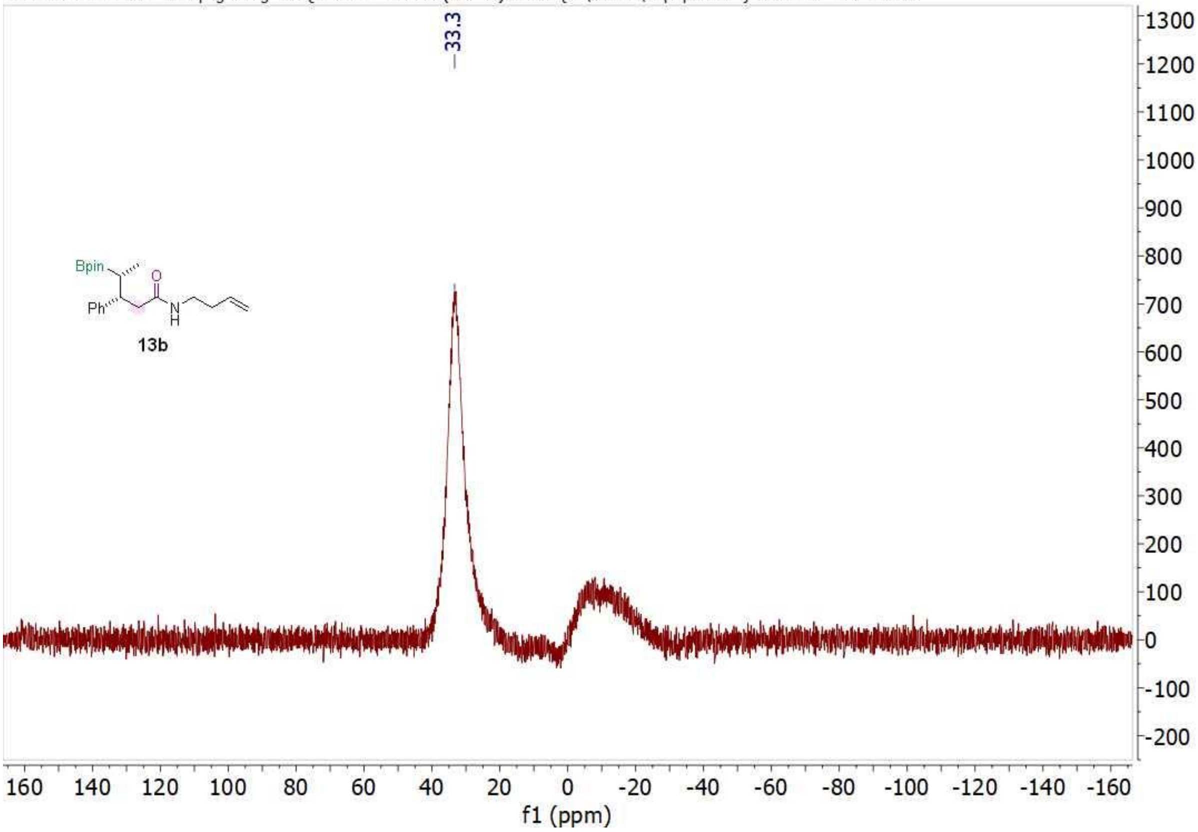

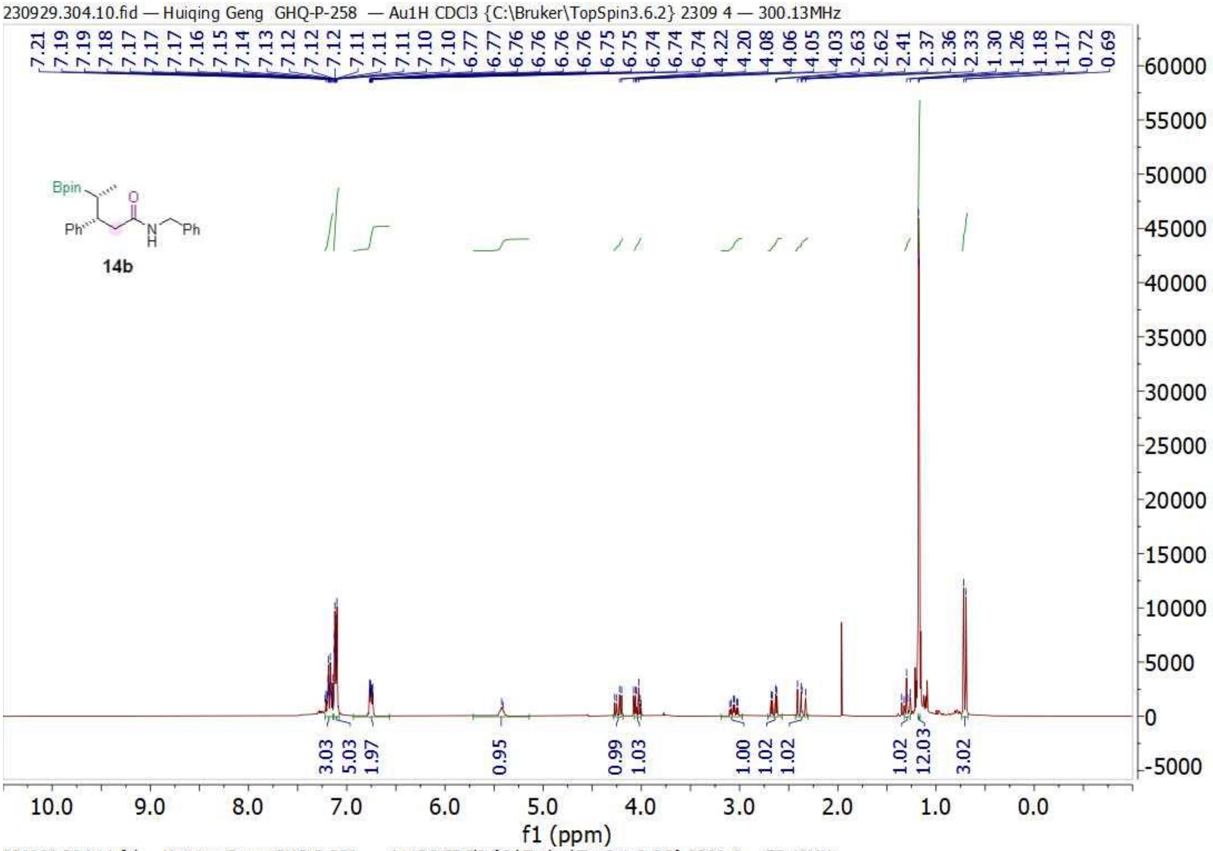
230929.304.10.fid — Huiqing Geng GHQ-P-258 — Au1H CDCl3 {C:\Bruker\TopSpin3.6.2} 2309 4 — 300.13MHz
7.21 7.19 7.18 7.17 7.17 7.17 7.16 7.15 7.14 7.13 7.12 7.12 7.12 7.11 7.11 7.11 7.10 7.10 6.77 6.76 6.76 6.76 6.76 6.75 6.75 6.74 6.74 6.74 4.22 4.20 4.08 4.06 4.05 4.03 2.63 2.62 2.41 2.37 2.36 2.33 1.30 1.26 1.18 1.17 0.72 0.69
Bpin
Ph
N
H
Ph
14b
3.03 5.03 1.97 0.95 0.99 1.03 1.00 1.02 1.02 1.02 12.03 3.02
f1 (ppm)

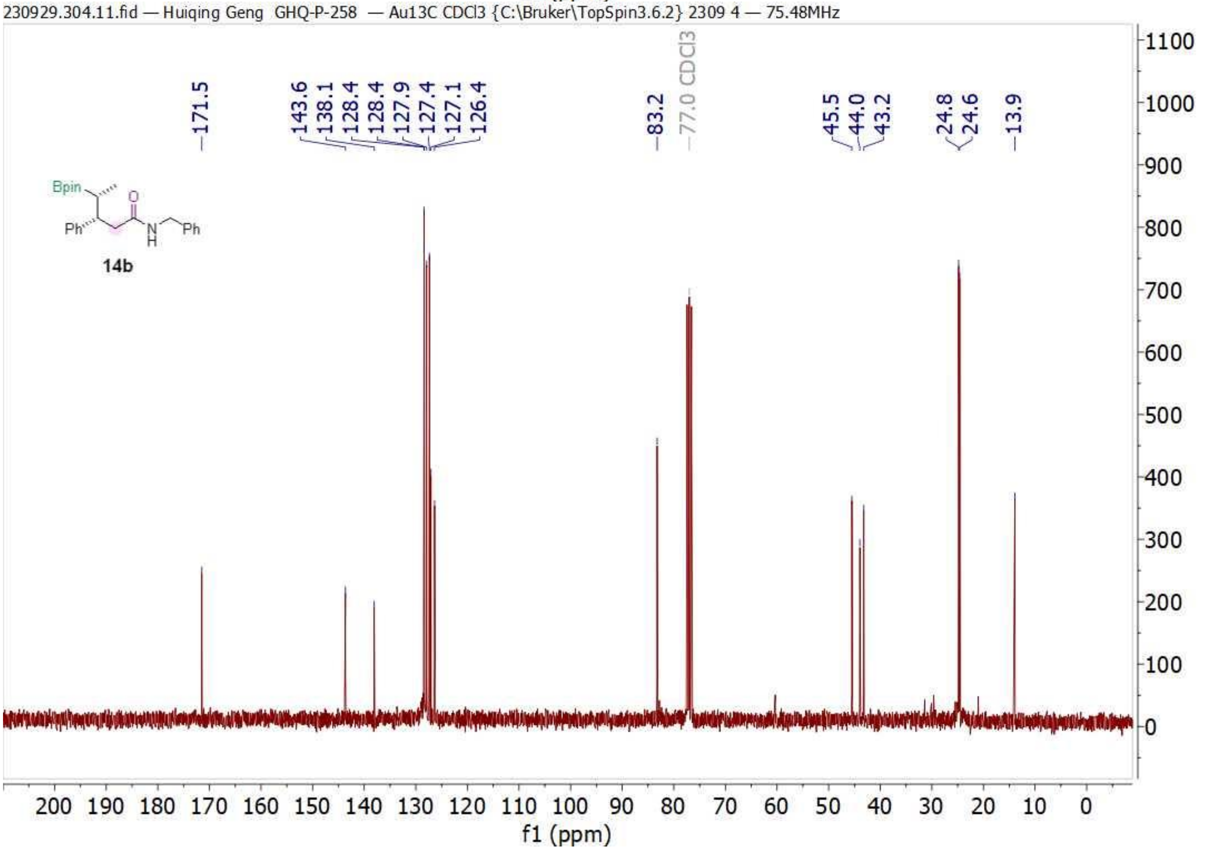
230929.304.11.fid — Huiqing Geng GHQ-P-258 — Au13C CDCl3 {C:\Bruker\TopSpin3.6.2} 2309 4 — 75.48MHz
171.5 143.6 138.1 128.4 128.4 127.9 127.4 127.1 126.4 83.2 77.0 CDCl3 45.5 44.0 43.2 24.8 24.6 13.9
Bpin
Ph
N
H
Ph
14b
f1 (ppm)

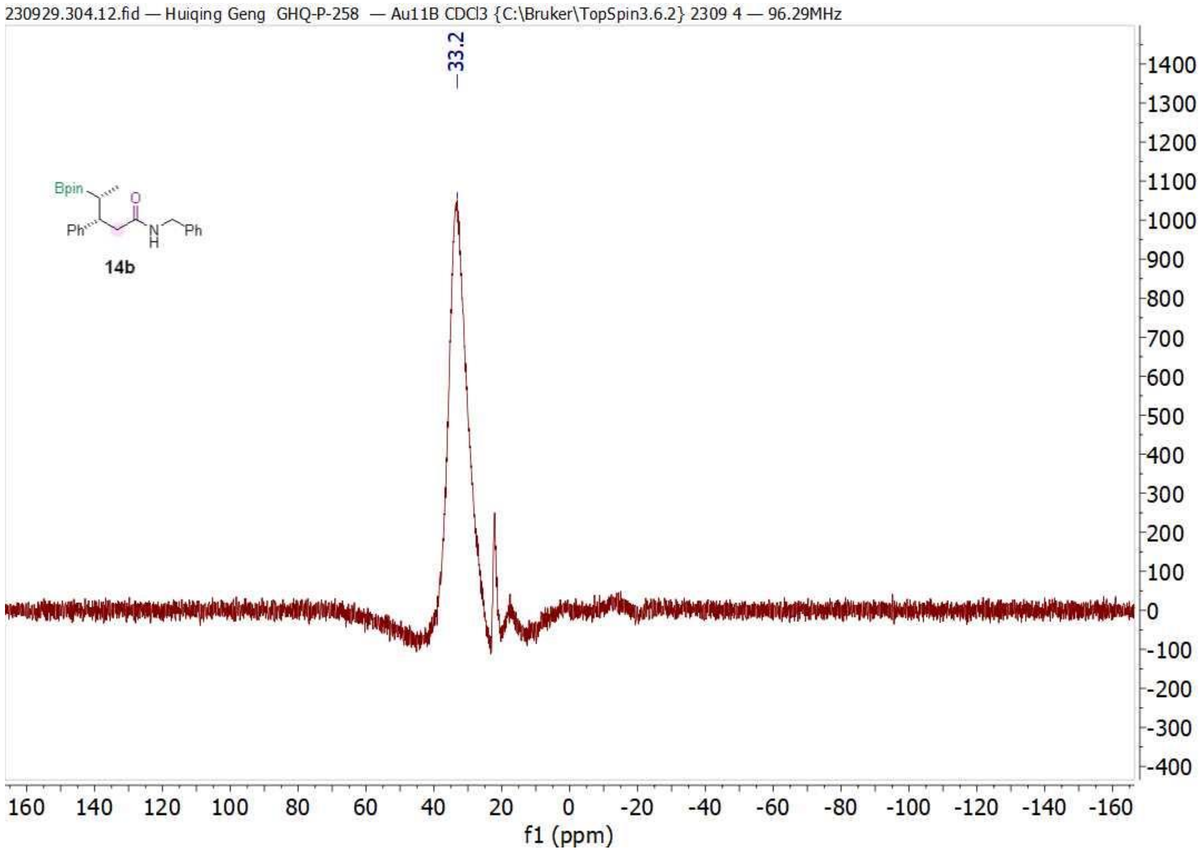
230929.304.12.fid — Huiqing Geng GHQ-P-258 — Au11B CDCl3 {C:\Bruker\TopSpin3.6.2} 2309 4 — 96.29MHz
33.2
Bpin
Ph
O
N
H
Ph
14b
f1 (ppm)

231001.307.10.fid — Huiqing Geng, GHQ-P-255 — Au1H CDCl3 {C:\Bruker\TopSpin3.6.2} 2310 7 — 300.13MHz

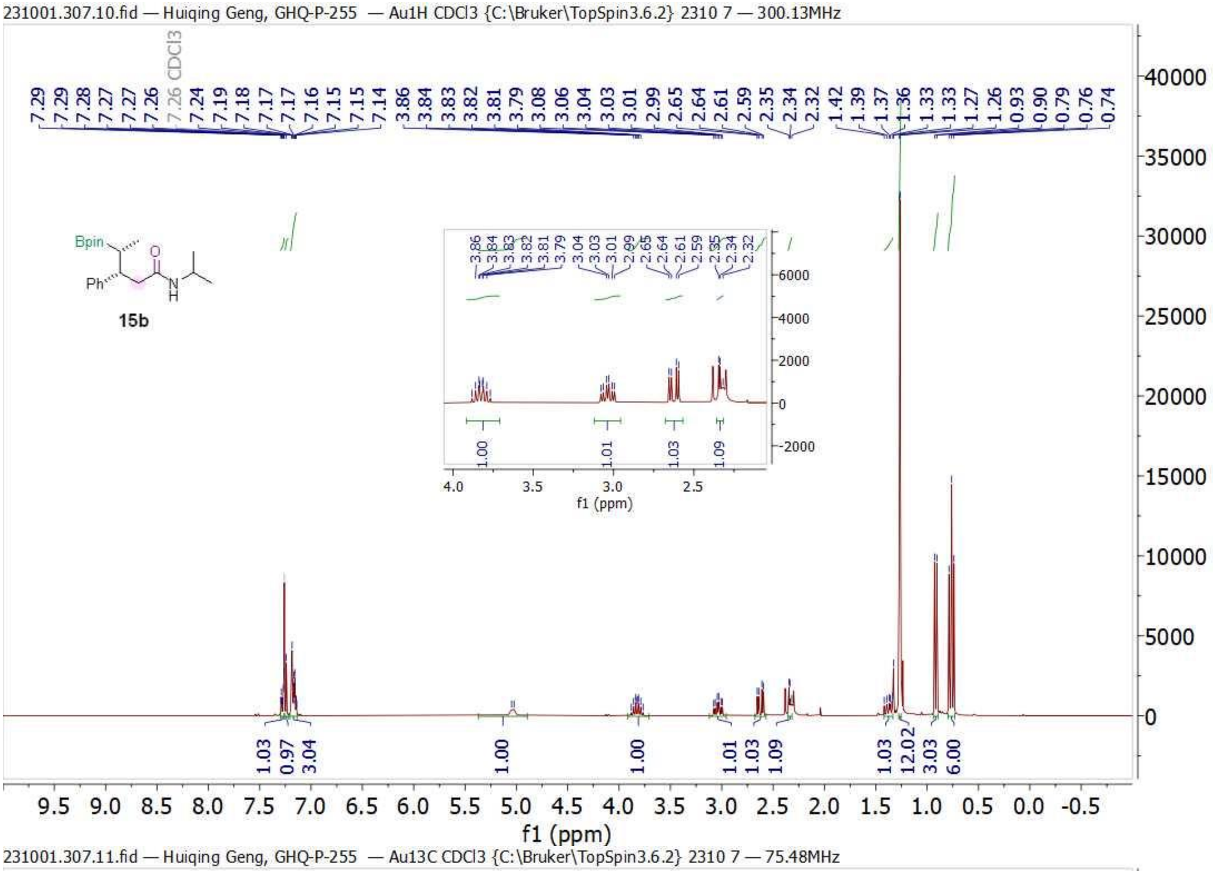

231001.307.11.fid — Huiqing Geng, GHQ-P-255 — Au13C CDCl3 {C:\Bruker\TopSpin3.6.2} 2310 7 — 75.48MHz

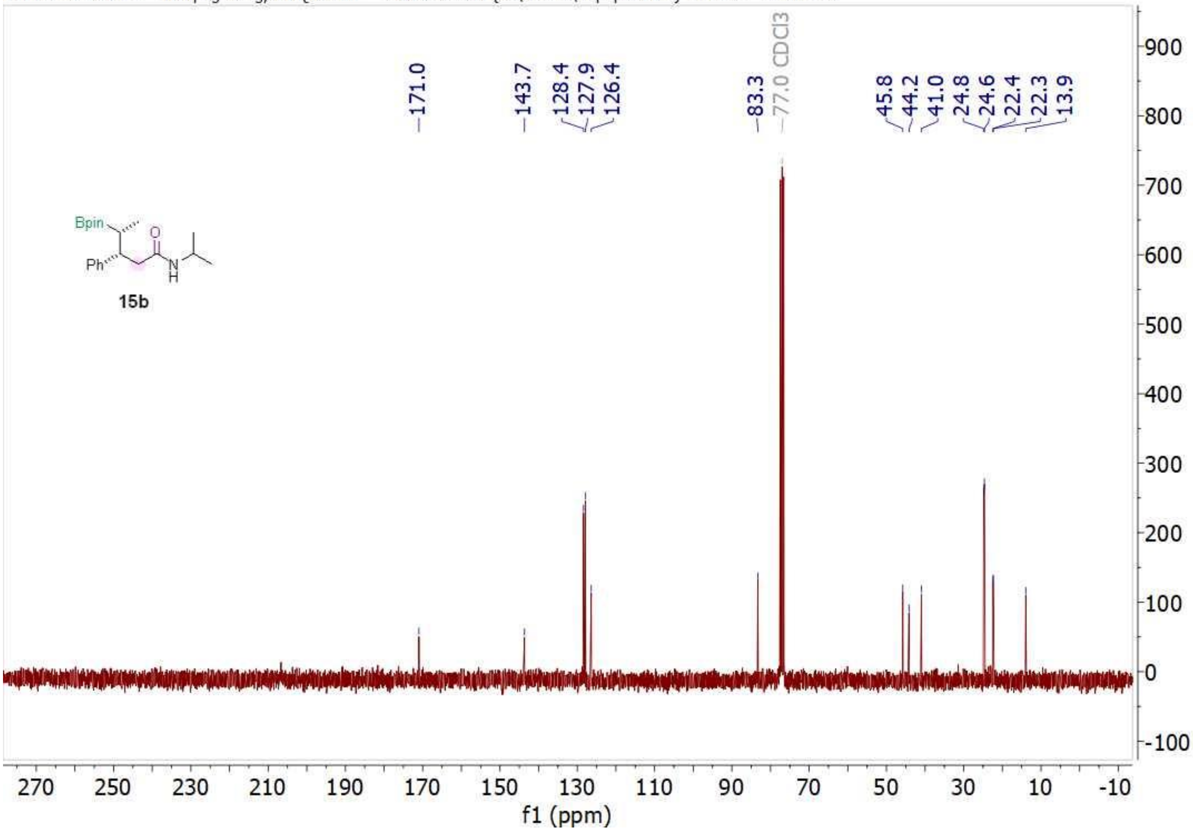

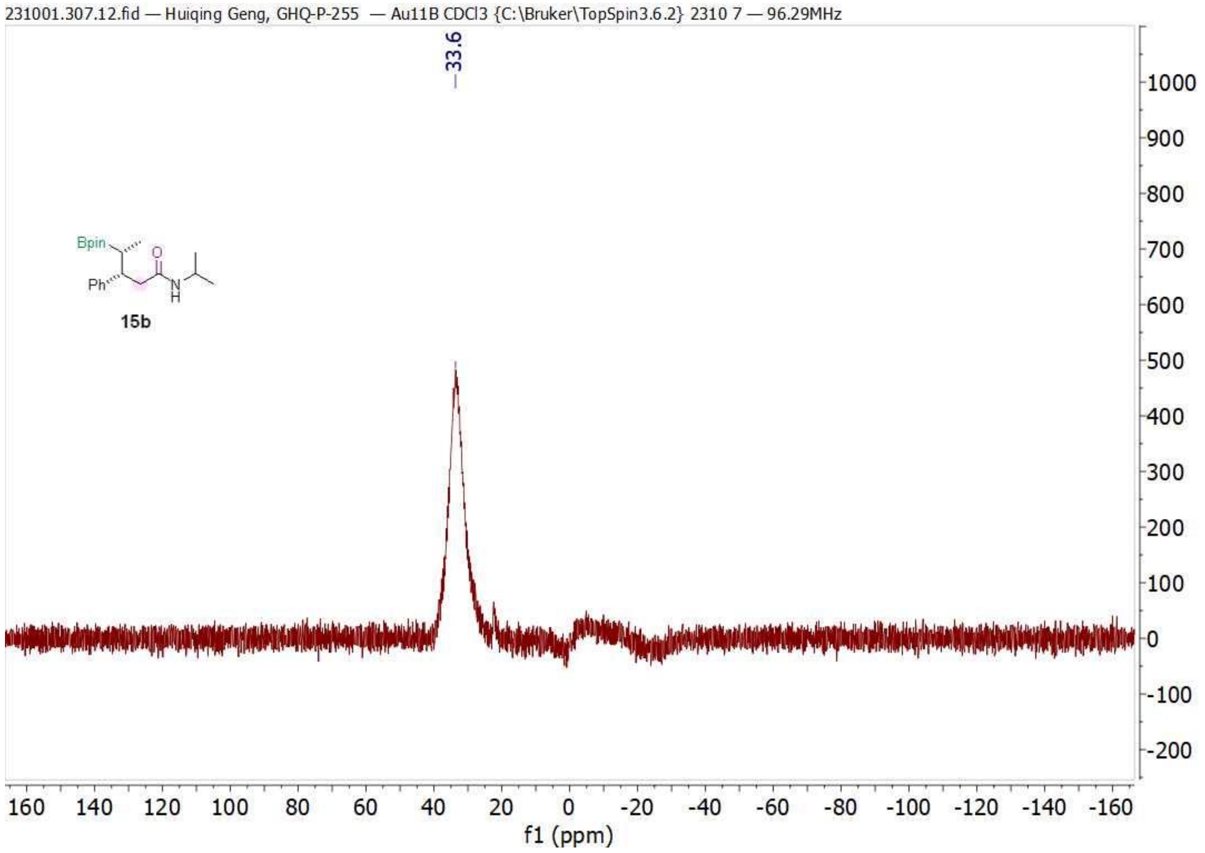
231001.307.12.fid — Huiqing Geng, GHQ-P-255 — Au11B CDCl3 {C:\Bruker\TopSpin3.6.2} 2310 7 — 96.29MHz
—33.6
Bpin
O
Ph
N
H
15b
f1 (ppm)

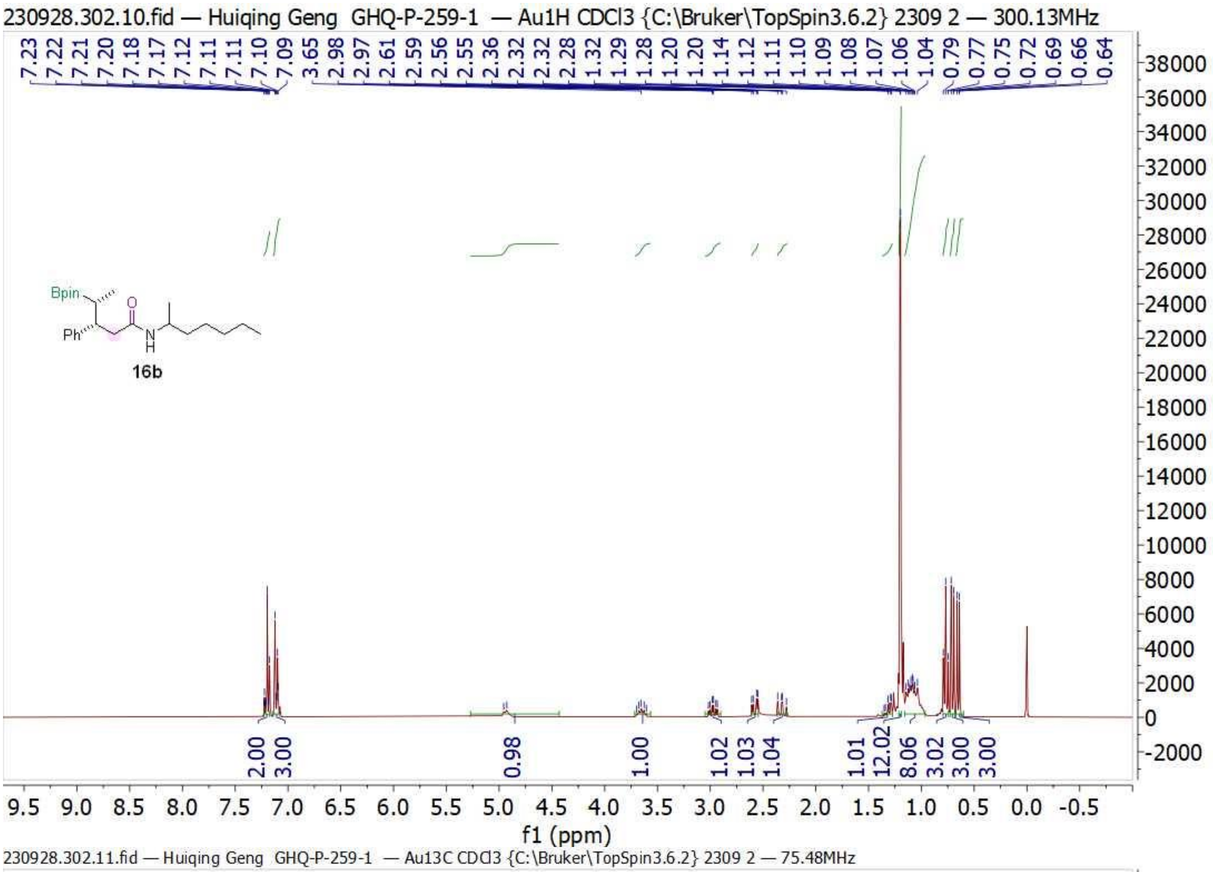
230928.302.10.fid — Huiqing Geng GHQ-P-259-1 — Au1H CDCl3 {C:\Bruker\TopSpin3.6.2} 2309 2 — 300.13MHz
7.23
7.22
7.21
7.20
7.18
7.17
7.12
7.11
7.11
7.10
7.09
3.65
2.98
2.97
2.61
2.59
2.56
2.55
2.36
2.32
2.32
2.28
1.32
1.29
1.28
1.20
1.20
1.14
1.12
1.11
1.10
1.09
1.08
1.07
1.06
1.04
0.79
0.77
0.75
0.72
0.69
0.66
0.64
Bpin
Ph
16b
2.00
3.00
0.98
1.00
1.02
1.03
1.04
1.01
12.02
8.06
3.02
3.00
3.00
f1 (ppm)

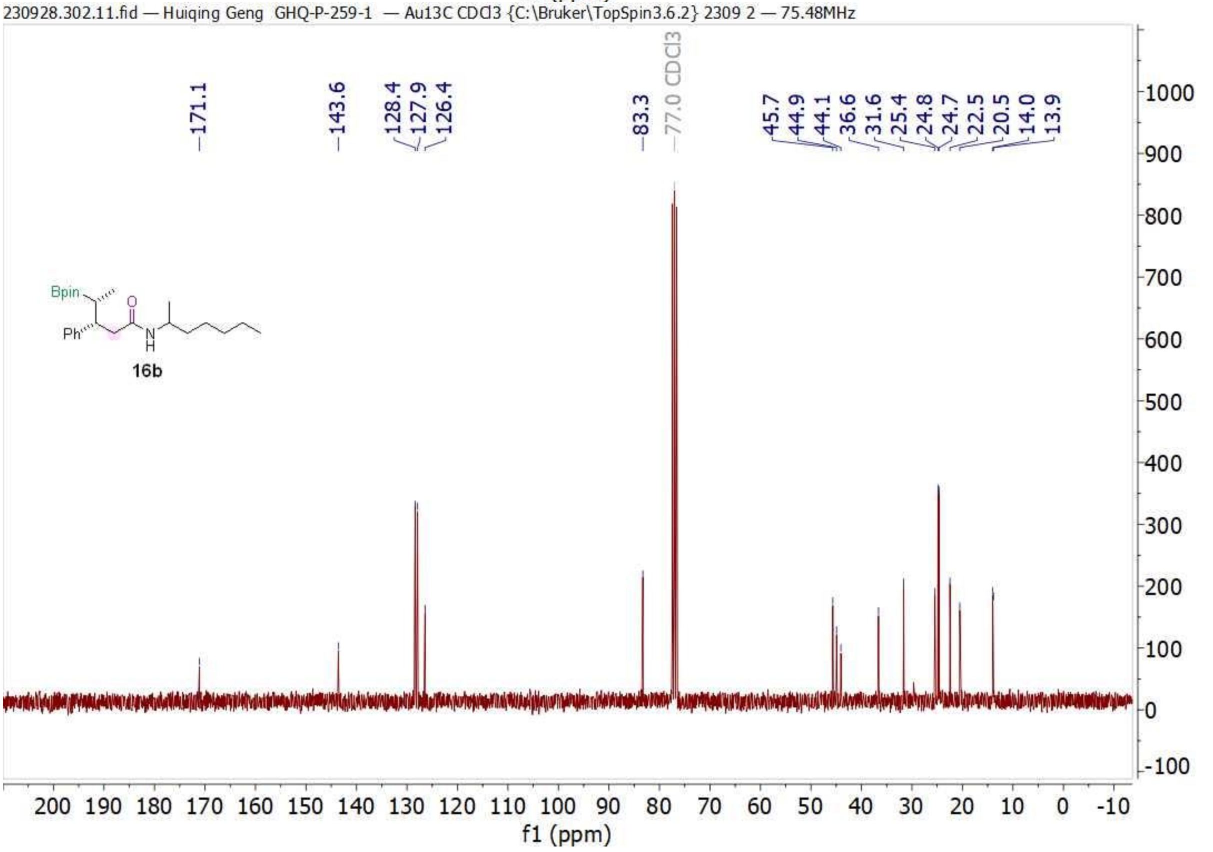
230928.302.11.fid — Huiqing Geng GHQ-P-259-1 — Au13C CDCl3 {C:\Bruker\TopSpin3.6.2} 2309 2 — 75.48MHz
171.1
143.6
128.4
127.9
126.4
83.3
77.0 CDCl3
45.7
44.9
44.1
36.6
31.6
25.4
24.8
24.7
22.5
20.5
14.0
13.9
Bpin
Ph
16b
f1 (ppm)

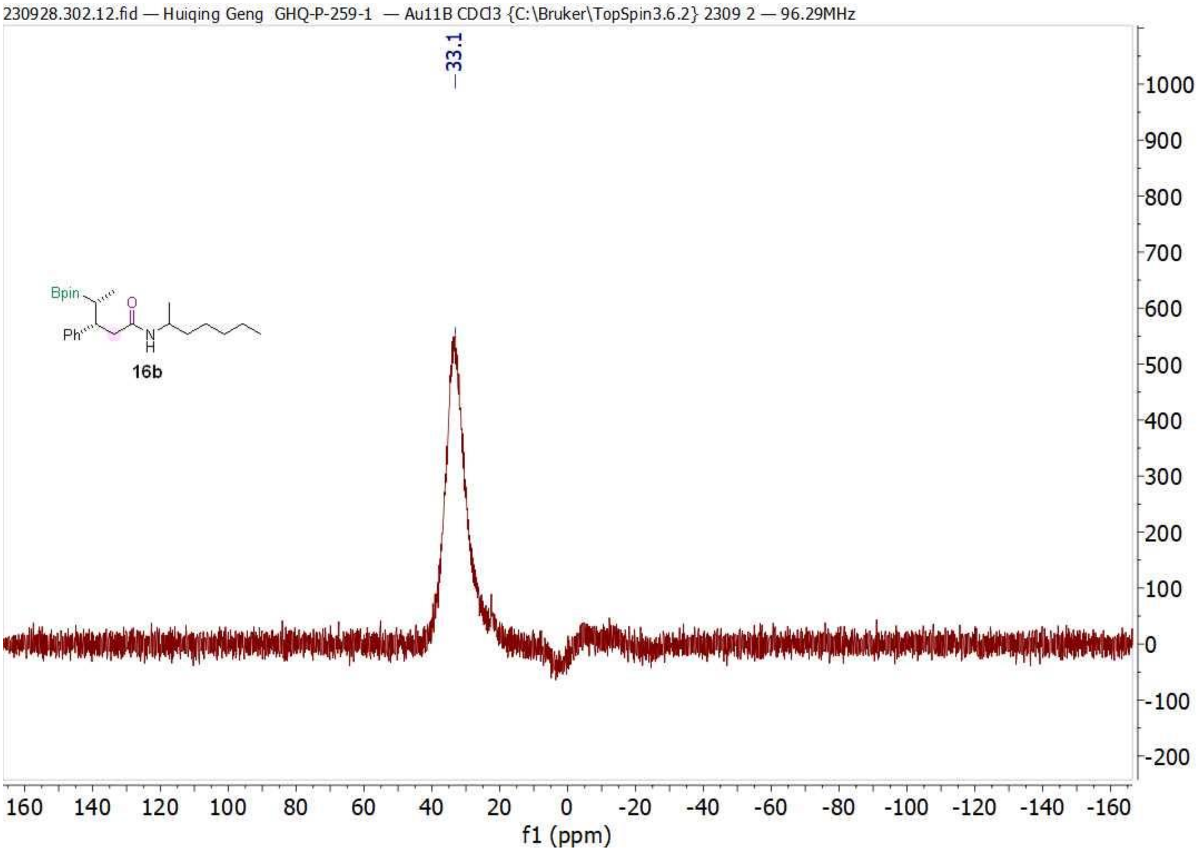
230928.302.12.fid — Huiqing Geng GHQ-P-259-1 — Au11B CDCl3 {C:\Bruker\TopSpin3.6.2} 2309 2 — 96.29MHz
—33.1
Bpin
Ph
O
N
H
16b
1000
900
800
700
600
500
400
300
200
100
0
-100
-200
160 140 120 100 80 60 40 20 0 -20 -40 -60 -80 -100 -120 -140 -160
f1 (ppm)

231002.319.10.fid — Huiqing Geng GHQ-P-245 — Au1H CDCl3 {C:\Bruker\TopSpin3.6.2} 2310 19 — 300.13MHz

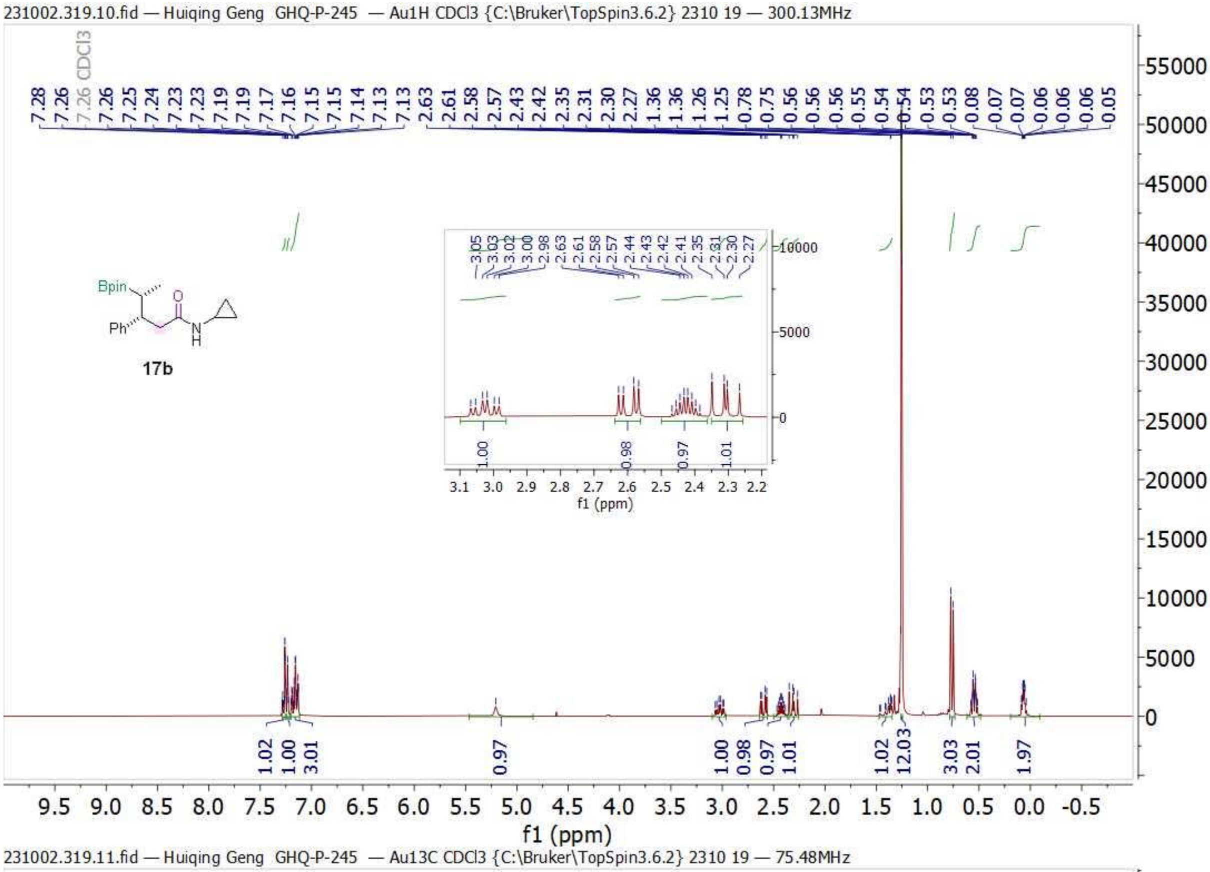

231002.319.11.fid — Huiqing Geng GHQ-P-245 — Au13C CDCl3 {C:\Bruker\TopSpin3.6.2} 2310 19 — 75.48MHz

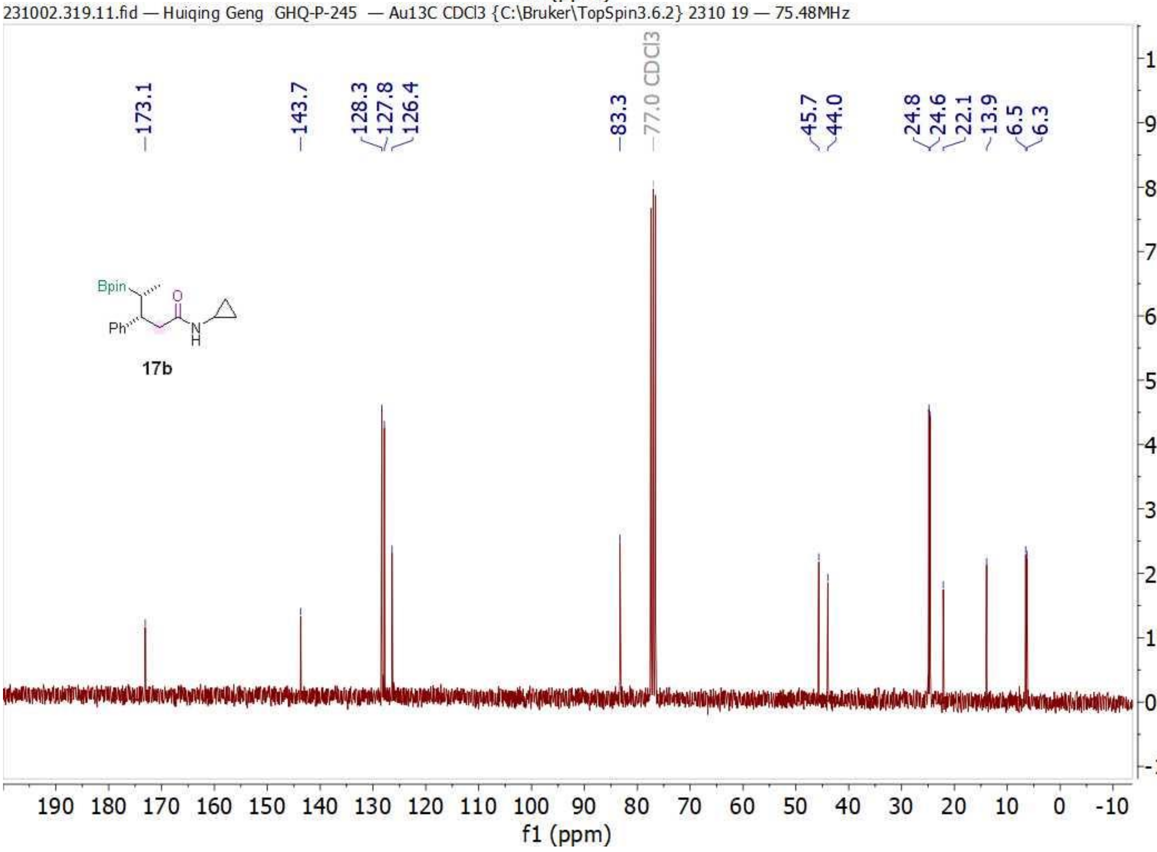

231002.319.12.fid — Huiqing Geng GHQ-P-245 — Au11B CDCl3 {C:\Bruker\TopSpin3.6.2} 2310 19 — 96.29MHz

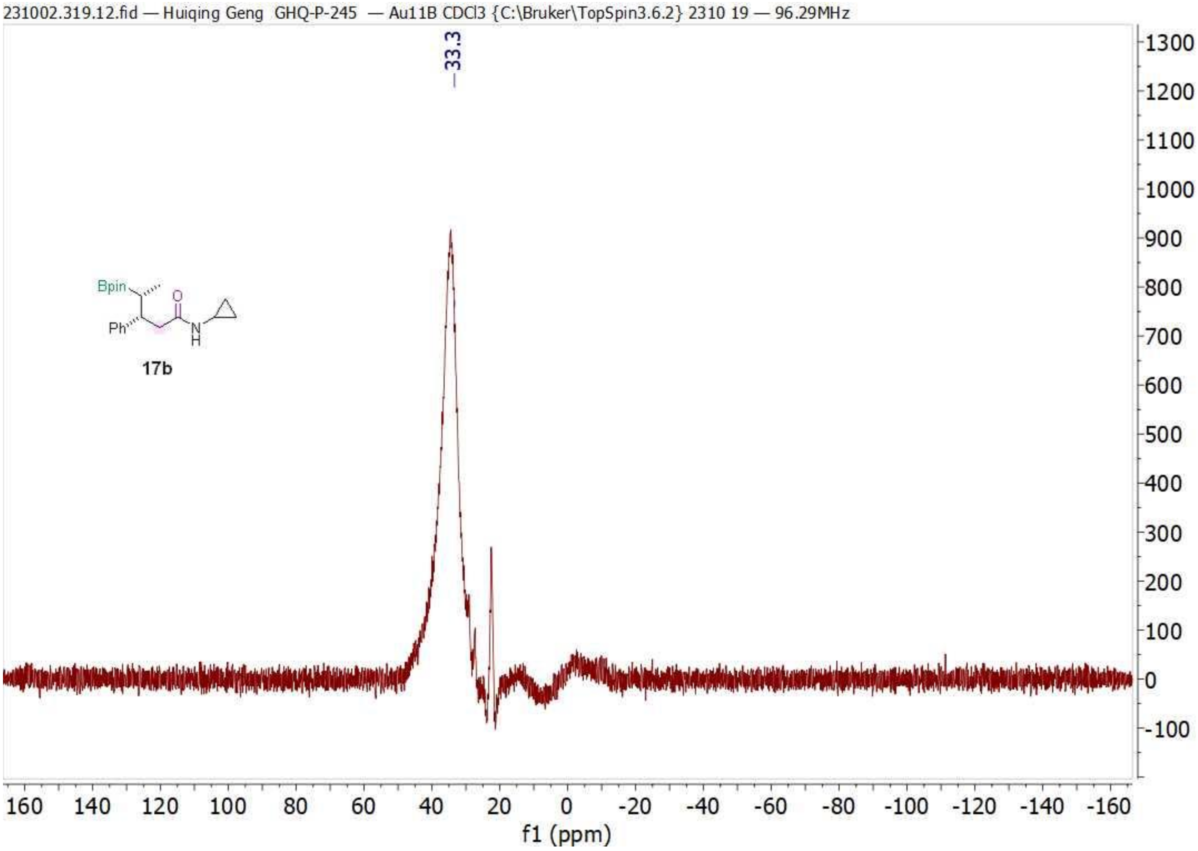

17b

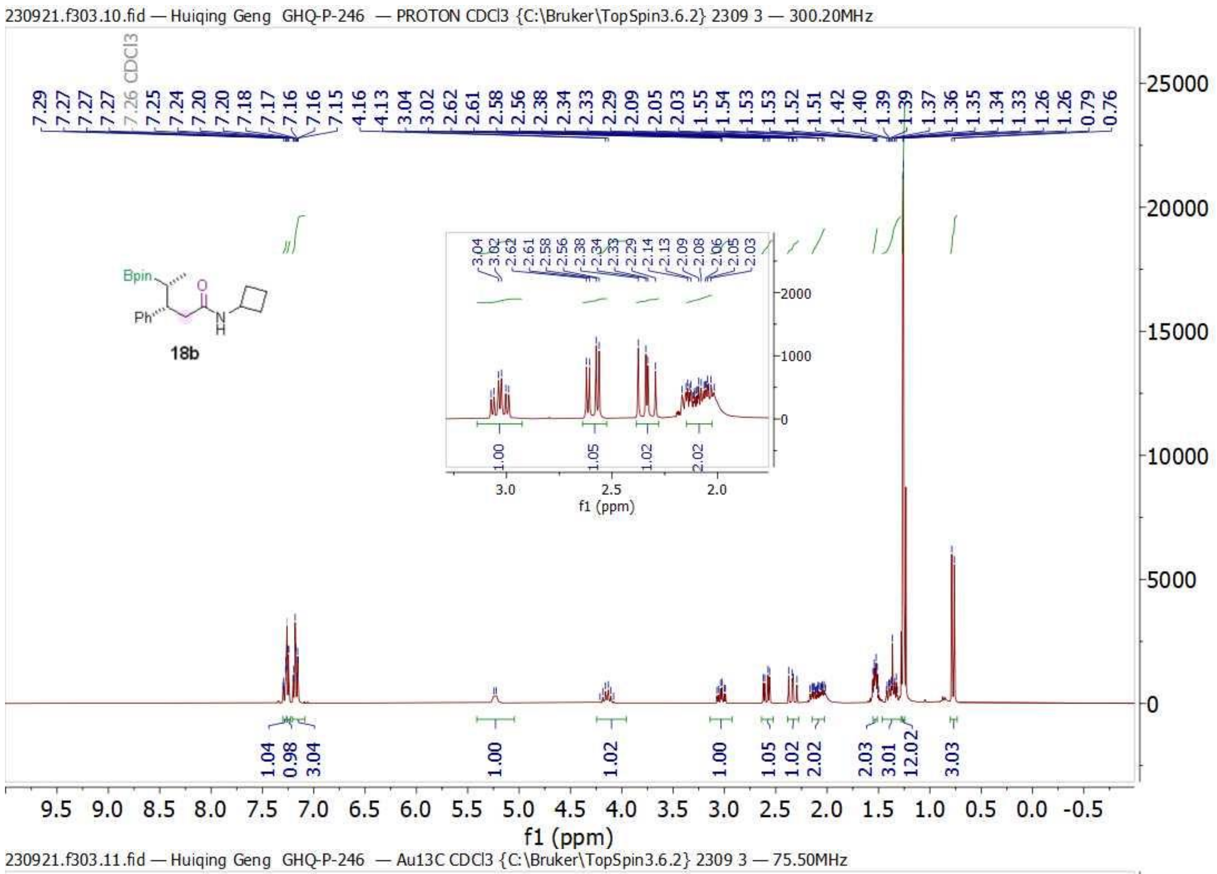
230921.f303.10.fid — Huiqing Geng GHQ-P-246 — PROTON CDCl3 {C:\Bruker\TopSpin3.6.2} 2309 3 — 300.20MHz
7.29 7.27 7.27 7.27 7.26 CDCl3 7.25 7.24 7.20 7.20 7.18 7.17 7.16 7.16 7.15 4.16 4.13 3.04 3.02 2.62 2.61 2.58 2.56 2.38 2.34 2.33 2.29 2.09 2.05 2.03 1.55 1.54 1.53 1.52 1.51 1.42 1.40 1.39 1.39 1.37 1.36 1.35 1.34 1.33 1.26 0.79 0.76
Bpin
Ph
N
H
O
18b
1.04 0.98 3.04 1.00 1.02 1.00 1.05 1.02 2.02 2.03 3.01 12.02 3.03
f1 (ppm)
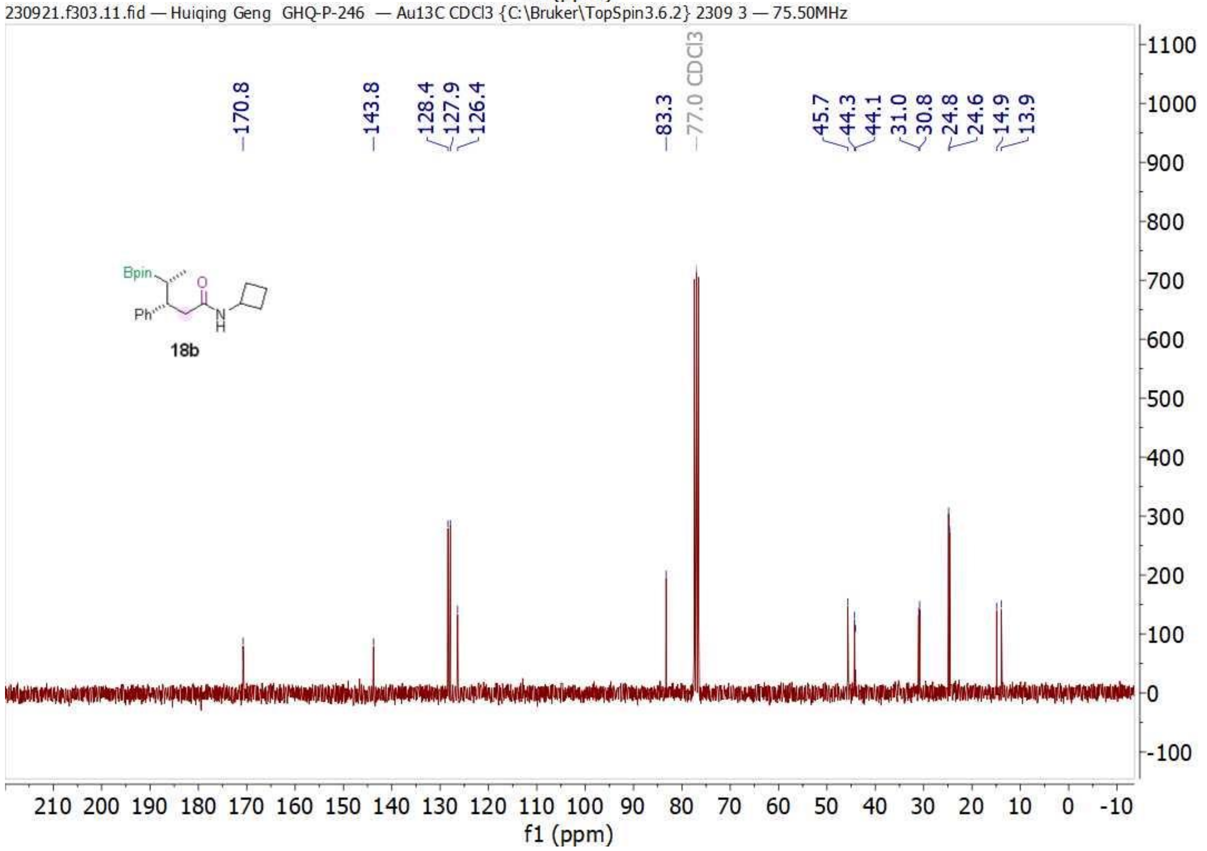
230921.f303.11.fid — Huiqing Geng GHQ-P-246 — Au13C CDCl3 {C:\Bruker\TopSpin3.6.2} 2309 3 — 75.50MHz
170.8 143.8 128.4 127.9 126.4 83.3 77.0 CDCl3 45.7 44.3 44.1 31.0 30.8 24.8 24.6 14.9 13.9
Bpin
Ph
N
H
O
18b
f1 (ppm)

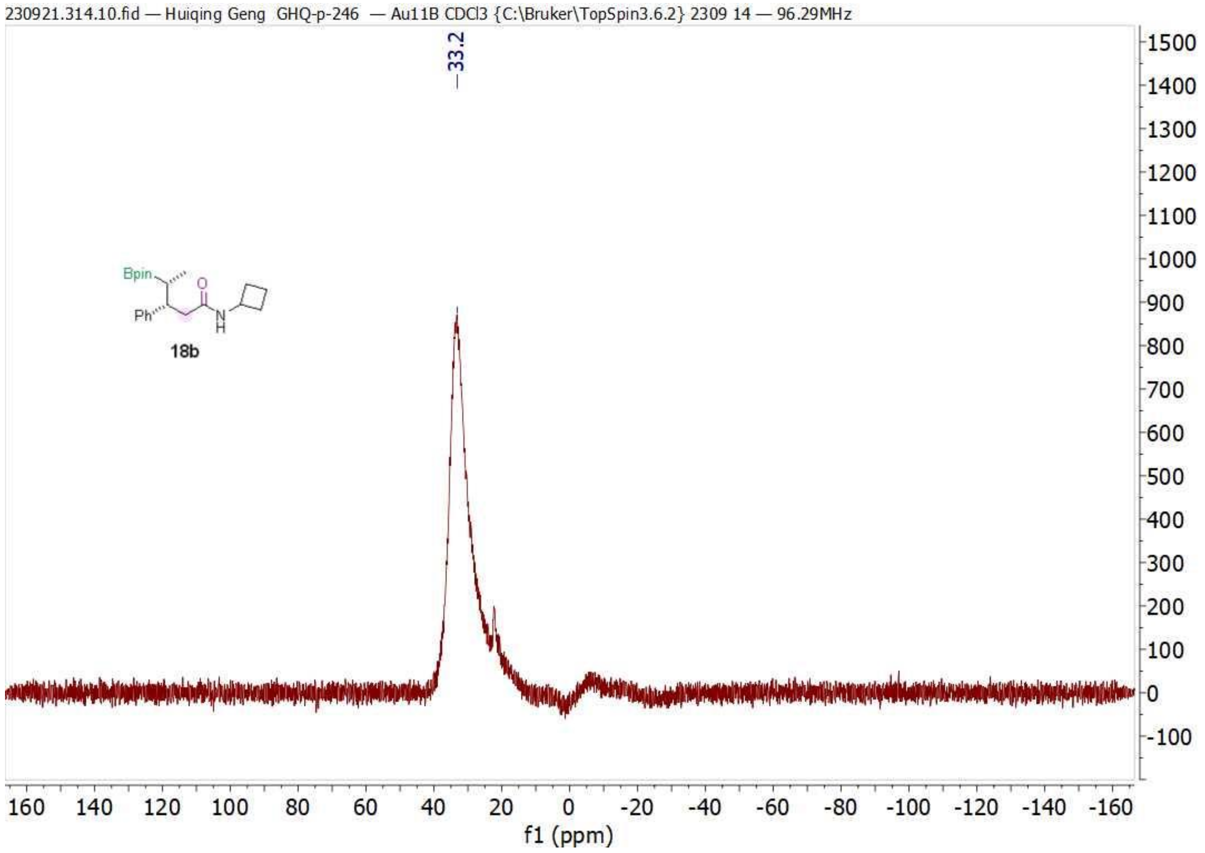

230921.314.10.fid — Huiqing Geng GHQ-p-246 — Au11B CDCl3 {C:\Bruker\TopSpin3.6.2} 2309 14 — 96.29MHz
33.2
Bpin
Ph
O
N
H
18b
f1 (ppm)

230922.f302.10.fid — Huiqing Geng GHQ-P-247-2 — PROTON CDCl3 {C:\Bruker\TopSpin3.6.2} 2309 2 — 300.20MHz

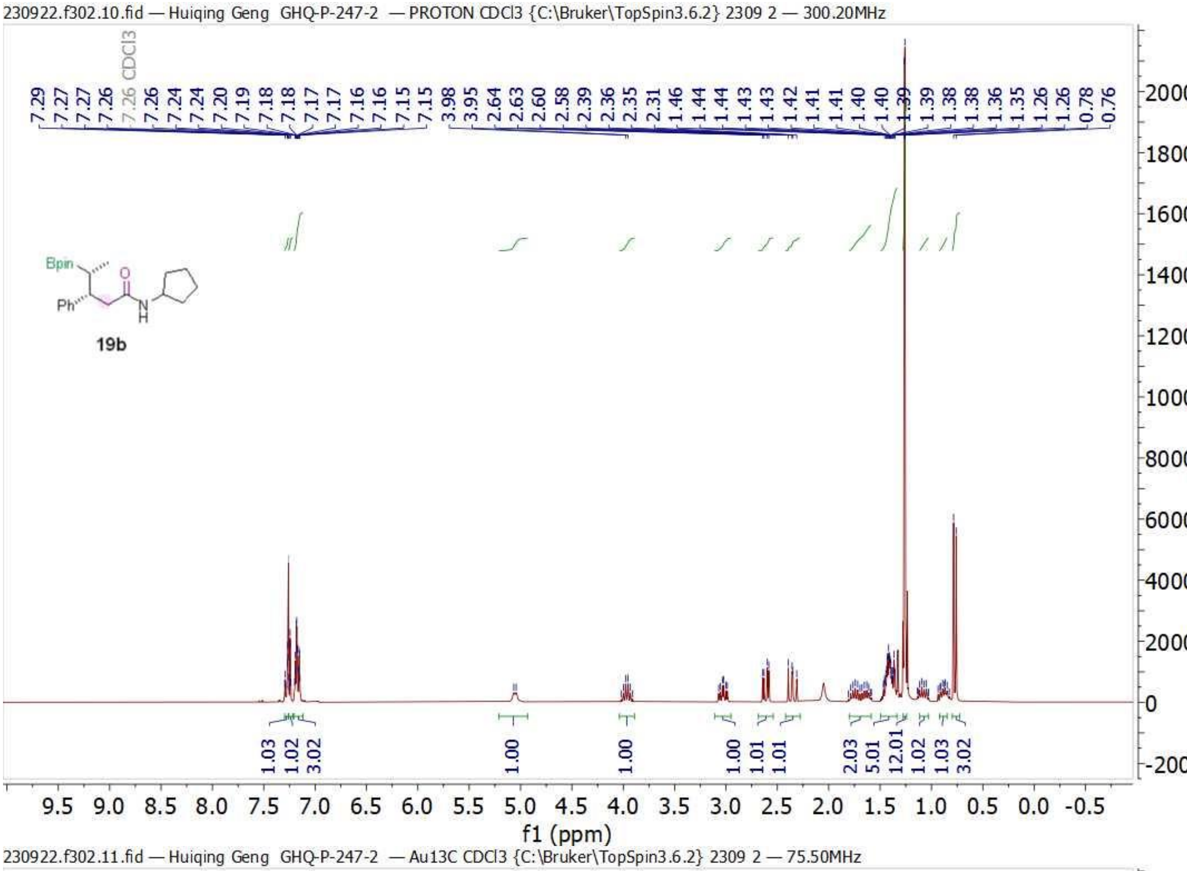

230922.f302.11.fid — Huiqing Geng GHQ-P-247-2 — Au13C CDCl3 {C:\Bruker\TopSpin3.6.2} 2309 2 — 75.50MHz

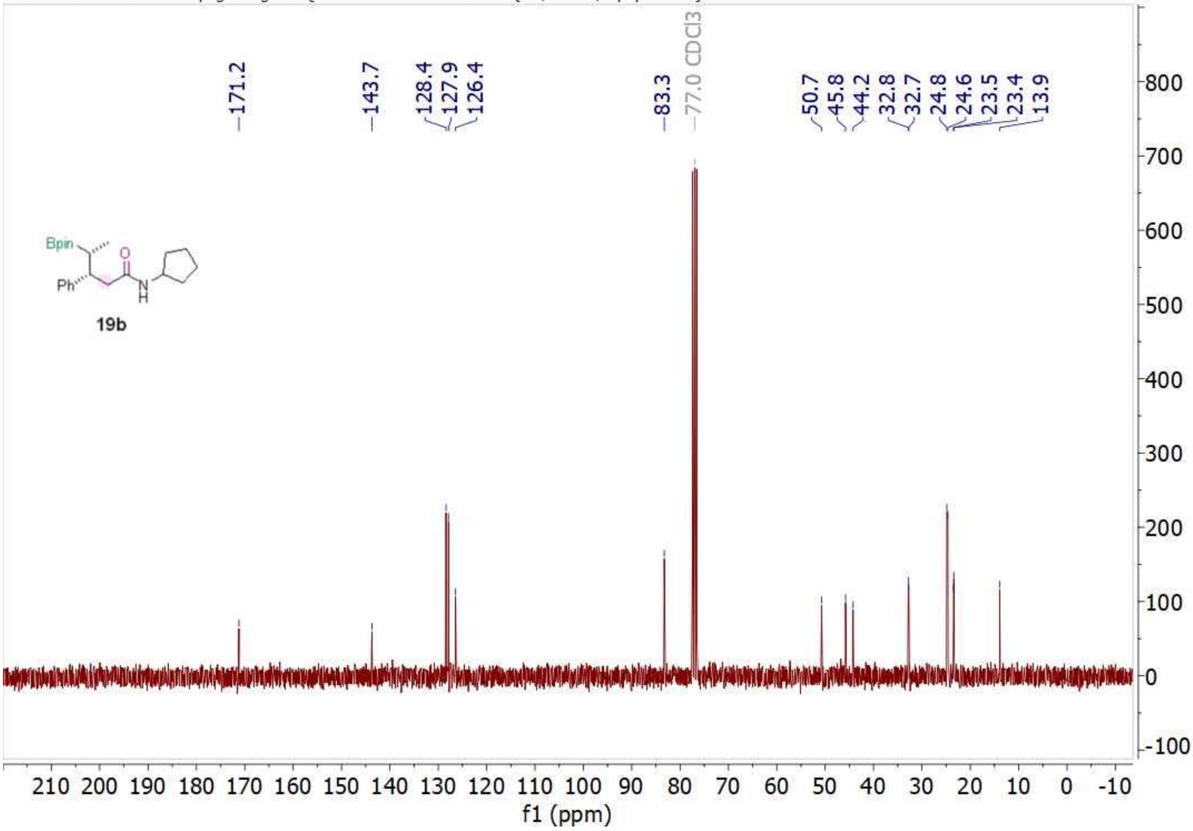

230925.301.10.fid — Huiqing Geng GHQ-P-247 — Au11B CDCl3 {C:\Bruker\TopSpin3.6.2} 2309 1 — 96.29MHz

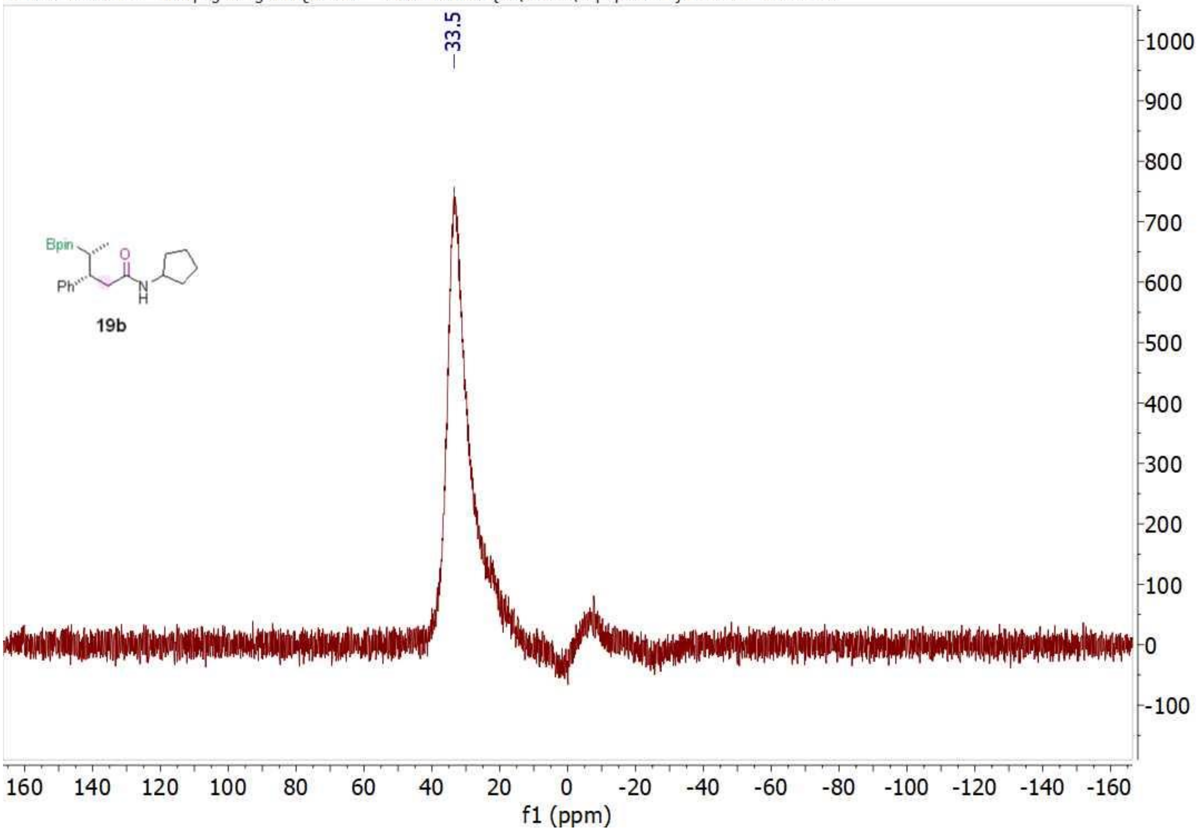

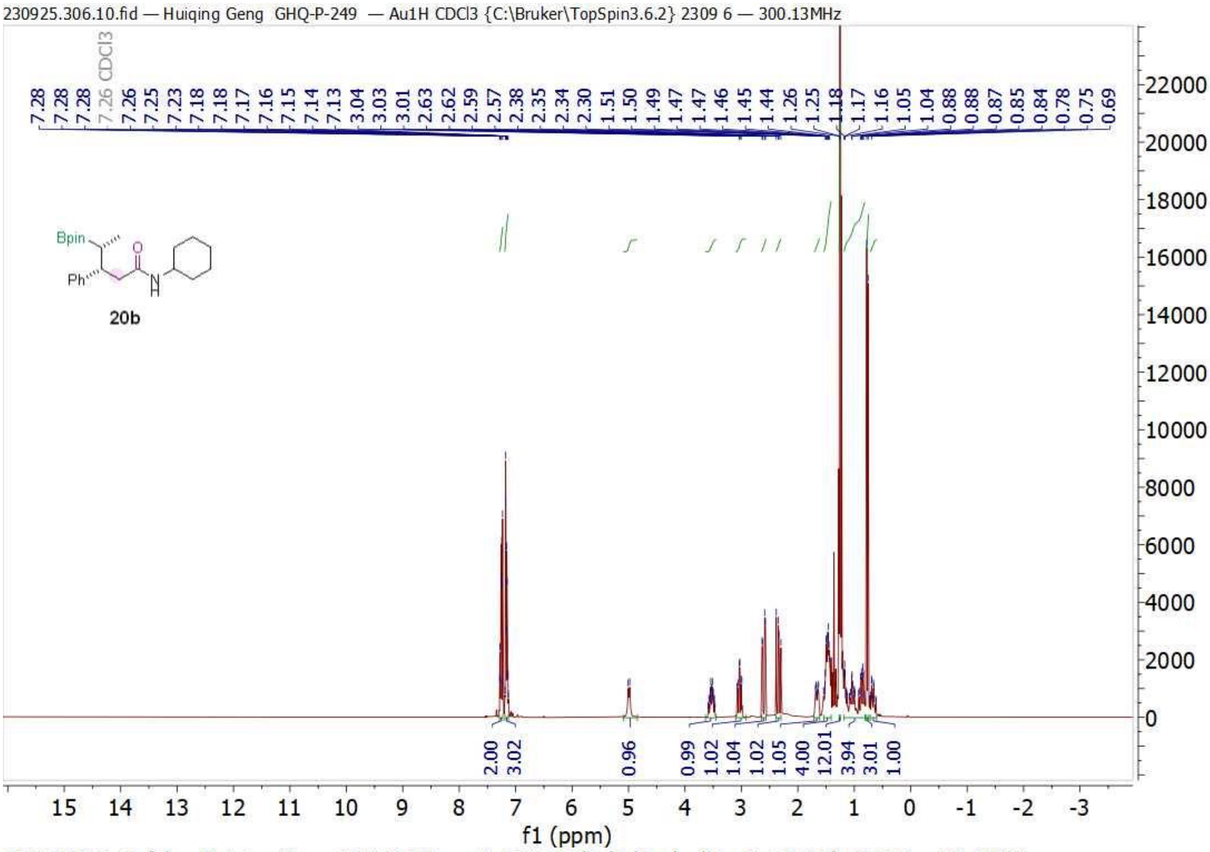
230925.306.10.fid — Huiqing Geng GHQ-P-249 — Au1H CDCl3 {C:\Bruker\TopSpin3.6.2} 2309 6 — 300.13MHz
7.28 7.28 7.28 7.26 CDCl3 7.26 7.25 7.23 7.18 7.18 7.17 7.16 7.15 7.14 7.13 3.04 3.03 3.01 2.63 2.62 2.59 2.57 2.38 2.35 2.34 2.30 1.51 1.50 1.49 1.47 1.47 1.46 1.45 1.44 1.26 1.25 1.18 1.17 1.16 1.05 1.04 0.88 0.88 0.87 0.85 0.84 0.78 0.75 0.69
Bpin
Ph
20b
2.00 3.02 0.96 0.99 1.02 1.04 1.02 1.05 4.00 12.01 3.94 3.01 1.00
f1 (ppm)

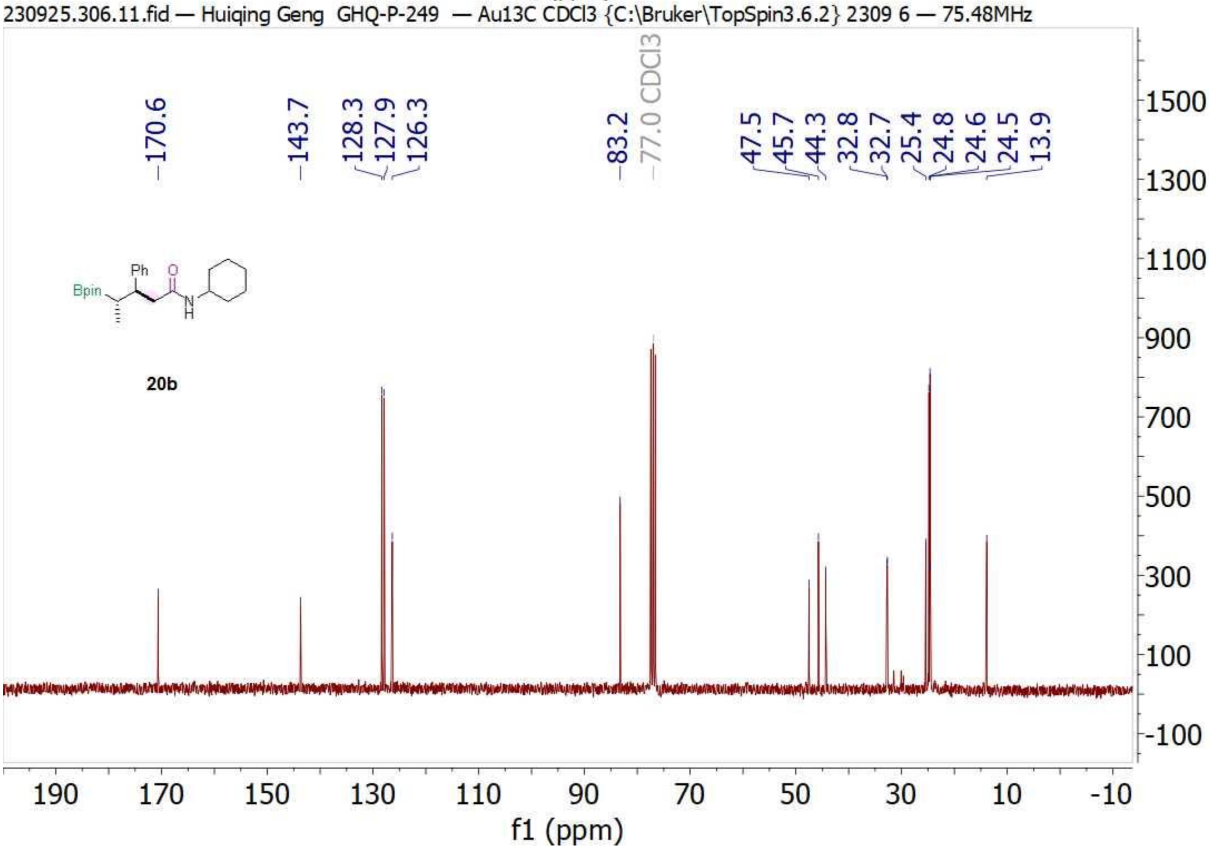
230925.306.11.fid — Huiqing Geng GHQ-P-249 — Au13C CDCl3 {C:\Bruker\TopSpin3.6.2} 2309 6 — 75.48MHz
170.6 143.7 128.3 127.9 126.3 83.2 77.0 CDCl3 47.5 45.7 44.3 32.8 32.7 25.4 24.8 24.6 24.5 13.9
Bpin
Ph
20b
f1 (ppm)

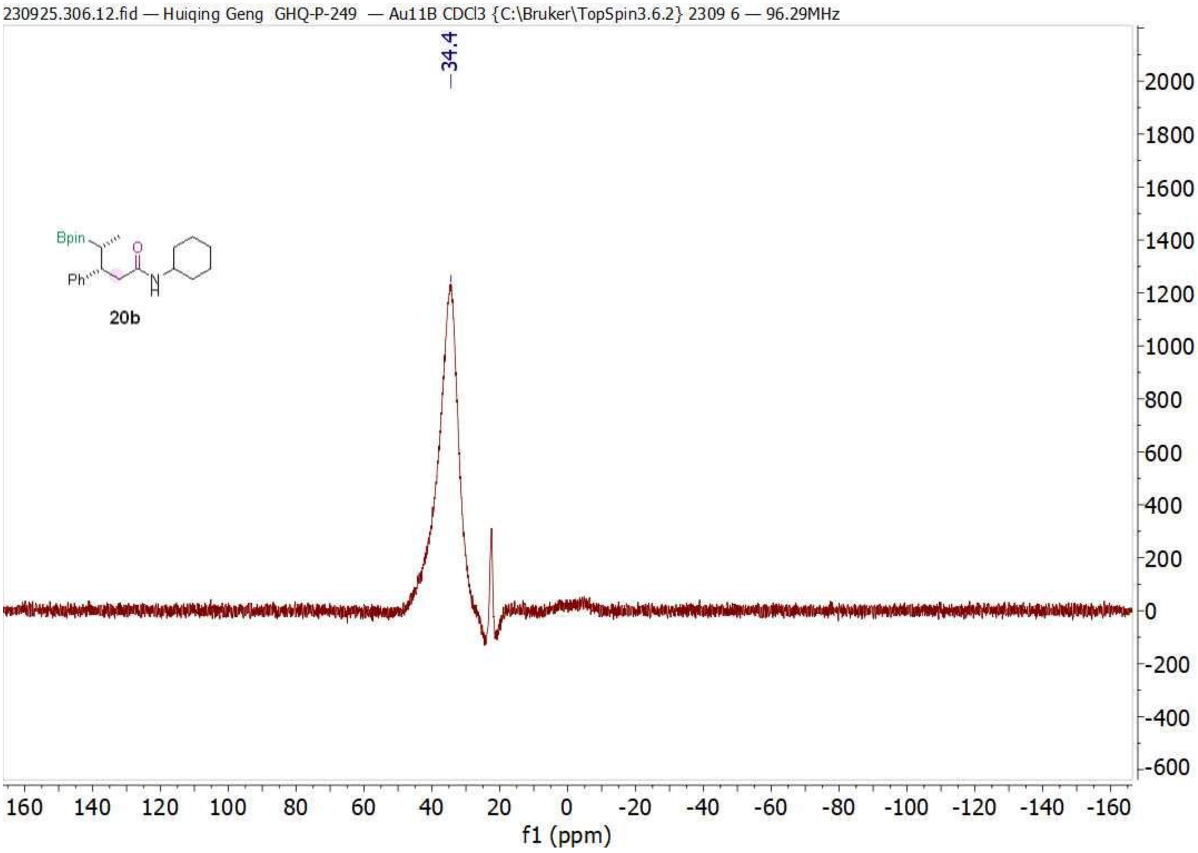
230925.306.12.fid — Huiqing Geng GHQ-P-249 — Au11B CDCl3 {C:\Bruker\TopSpin3.6.2} 2309 6 — 96.29MHz
34.4
Bpin
Ph
O
N
H
20b
f1 (ppm)

231005.428.10.fid — Huiqing Geng GHQ-P-276 — Au1H CDCl3 {C:\Bruker\TopSpin3.6.2} 2310 28 — 400.13MHz

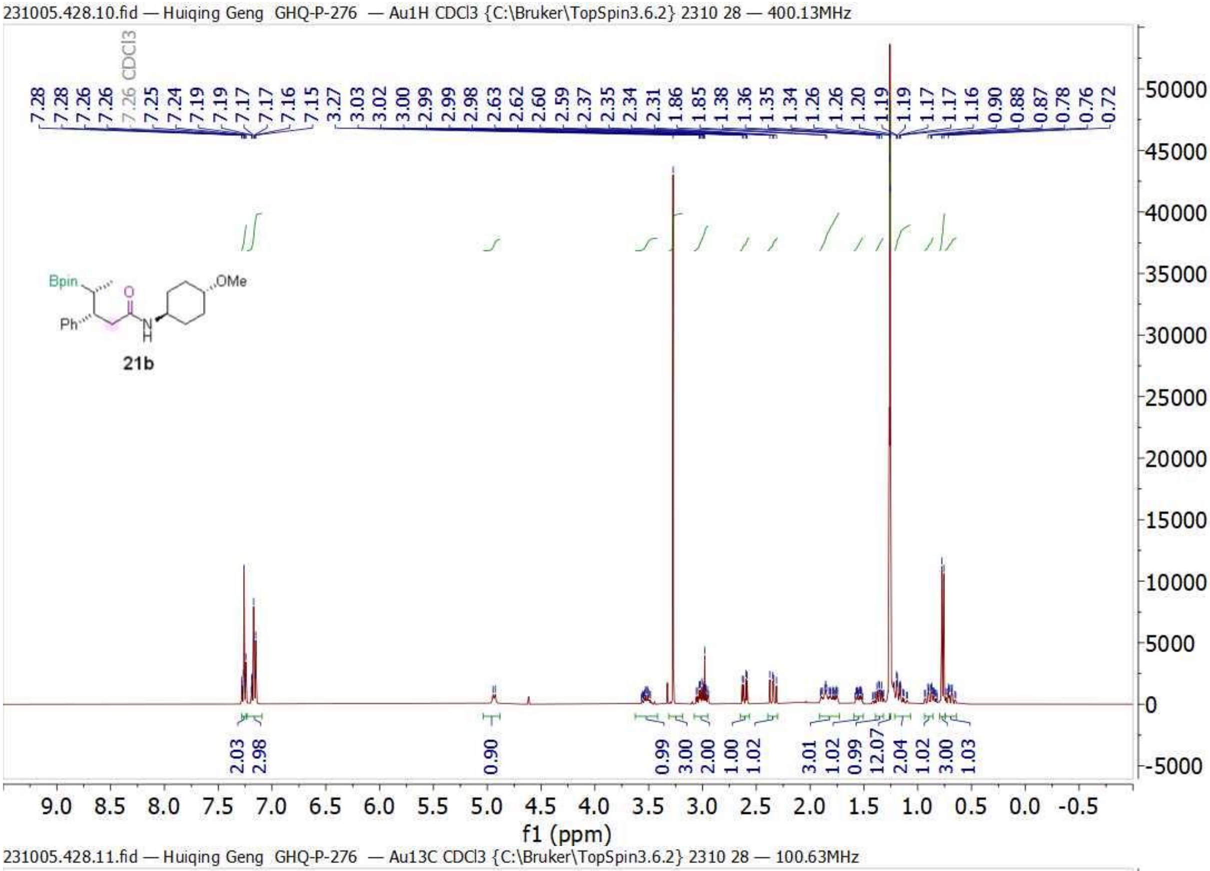

231005.428.11.fid — Huiqing Geng GHQ-P-276 — Au13C CDCl3 {C:\Bruker\TopSpin3.6.2} 2310 28 — 100.63MHz

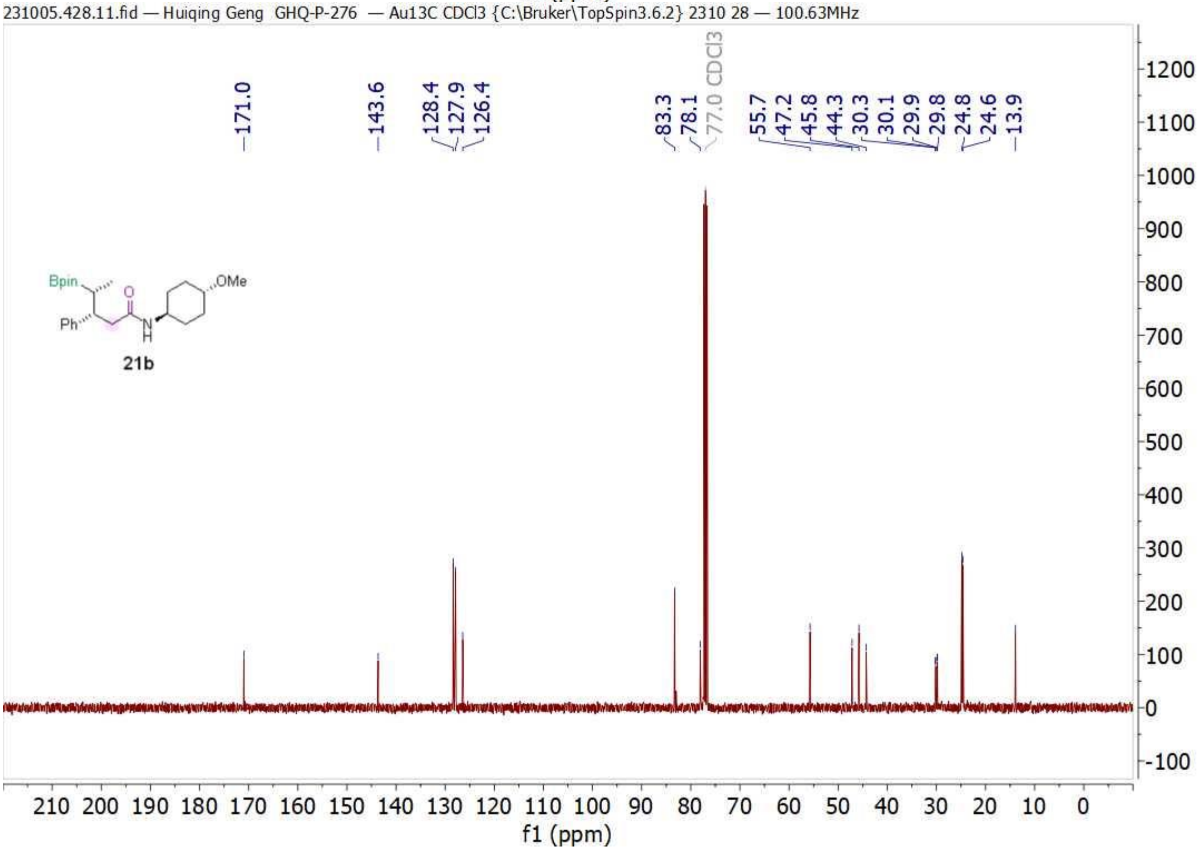

231005.428.12.fid — Huiqing Geng GHQ-P-276 — Au11B CDCl3 {C:\Bruker\TopSpin3.6.2} 2310 28 — 128.38MHz

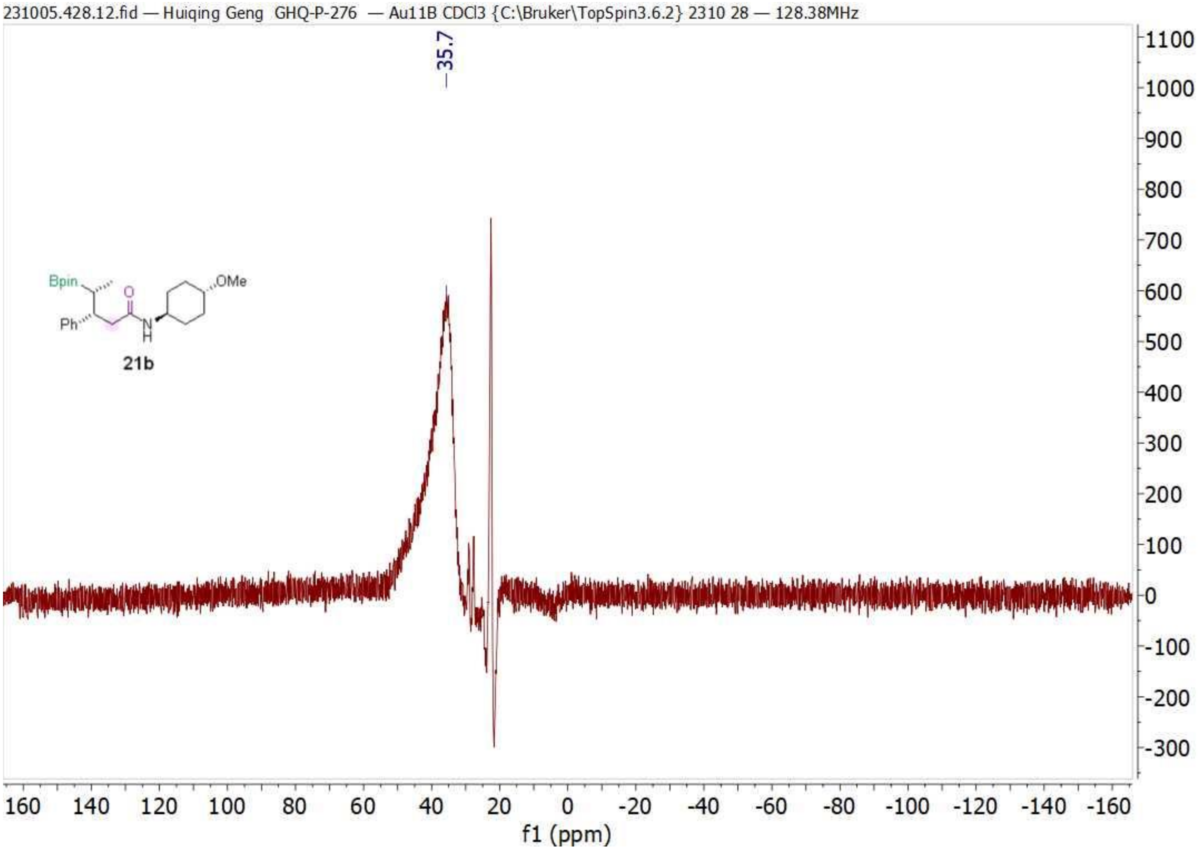

231004.307.10.fid — Huiqing Geng GHQ-P-281 — Au1H CDCl3 {C:\Bruker\TopSpin3.6.2} 2310 7 — 300.13MHz

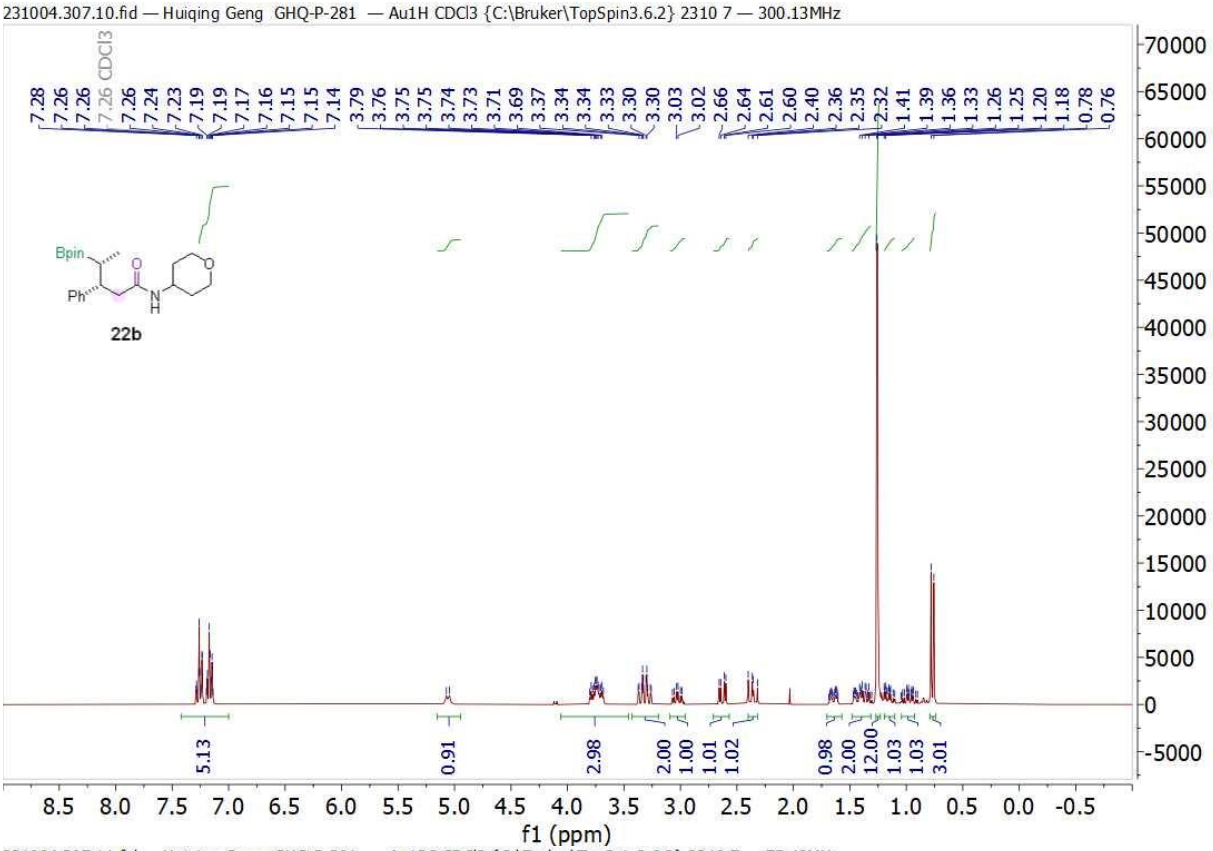

231004.307.11.fid — Huiqing Geng GHQ-P-281 — Au13C CDCl3 {C:\Bruker\TopSpin3.6.2} 2310 7 — 75.48MHz

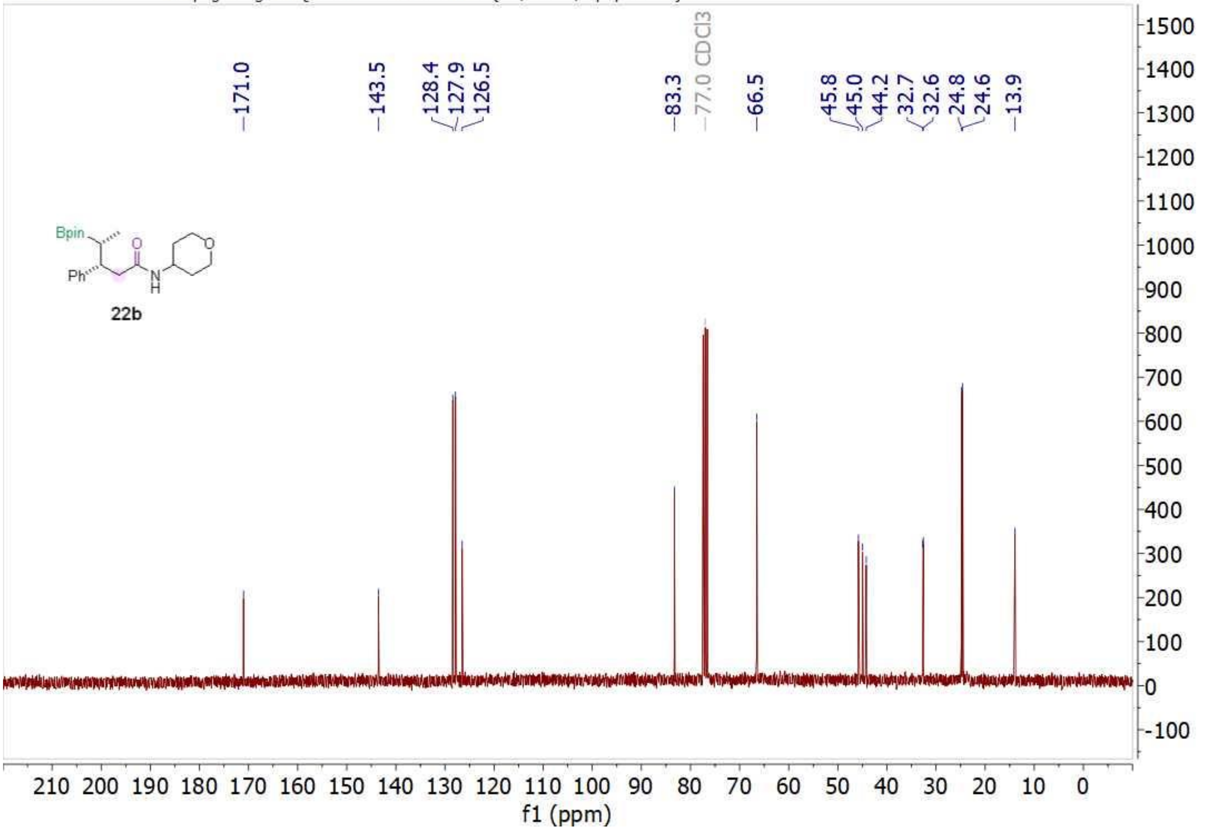

231004.307.12.fid — Huiqing Geng GHQ-P-281 — Au11B CDCl3 {C:\Bruker\TopSpin3.6.2} 2310 7 — 96.29MHz

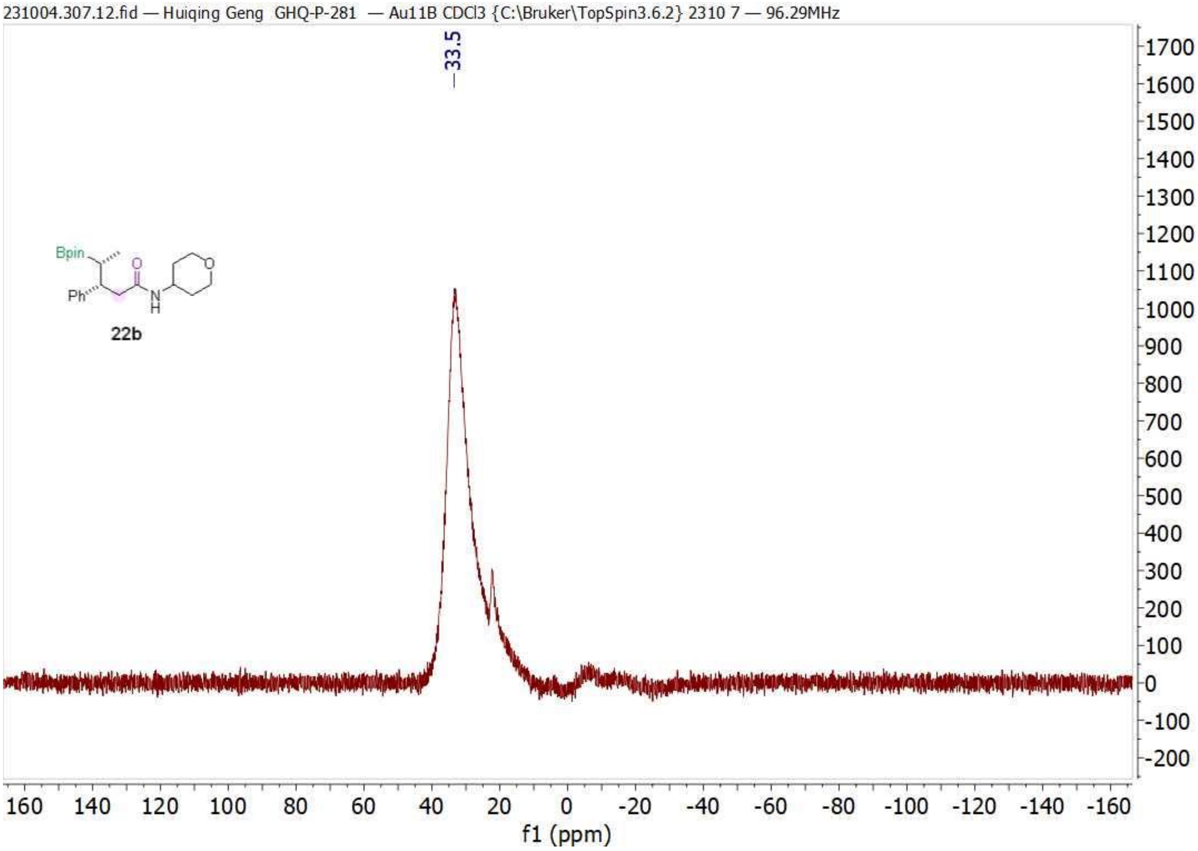

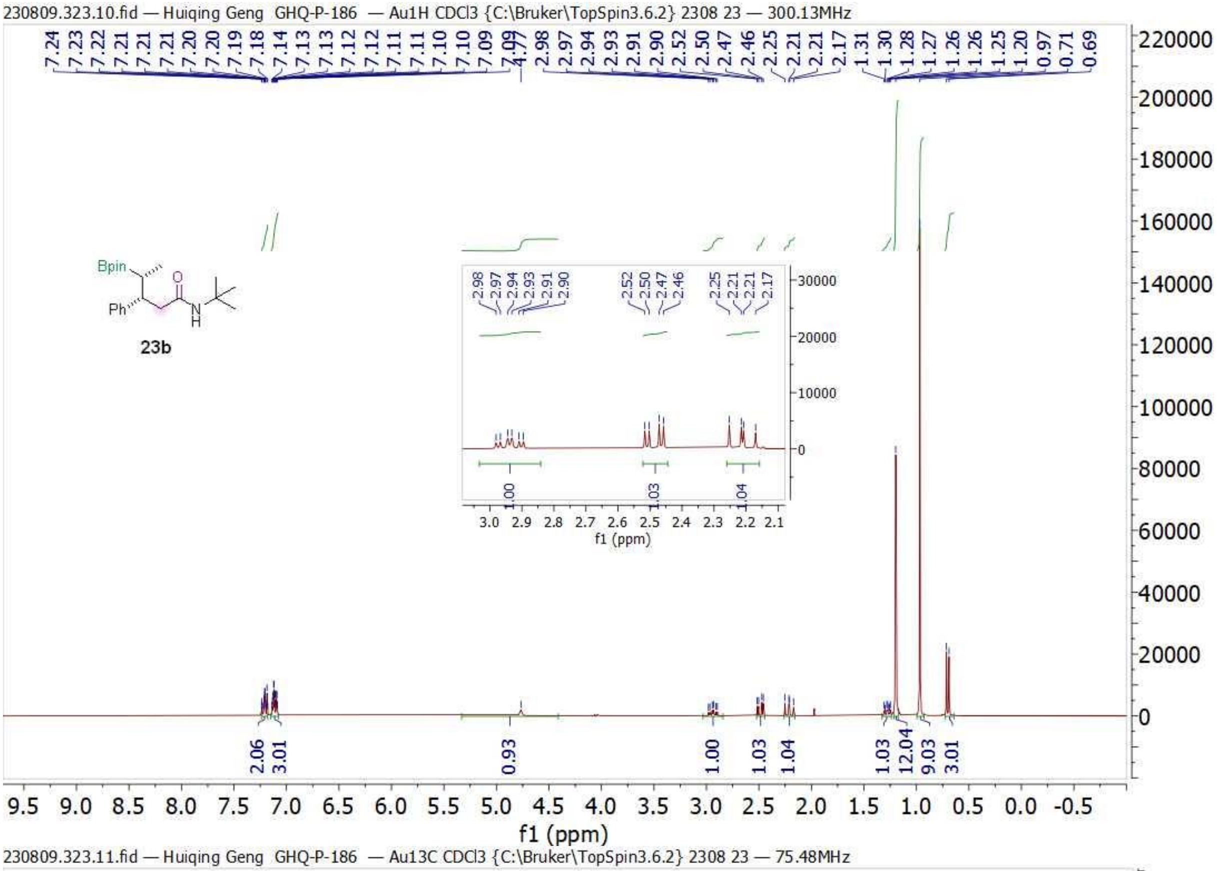
230809.323.10.fid — Huiqing Geng GHQ-P-186 — Au1H CDCl3 {C:\Bruker\TopSpin3.6.2} 2308 23 — 300.13MHz
23b
f1 (ppm)

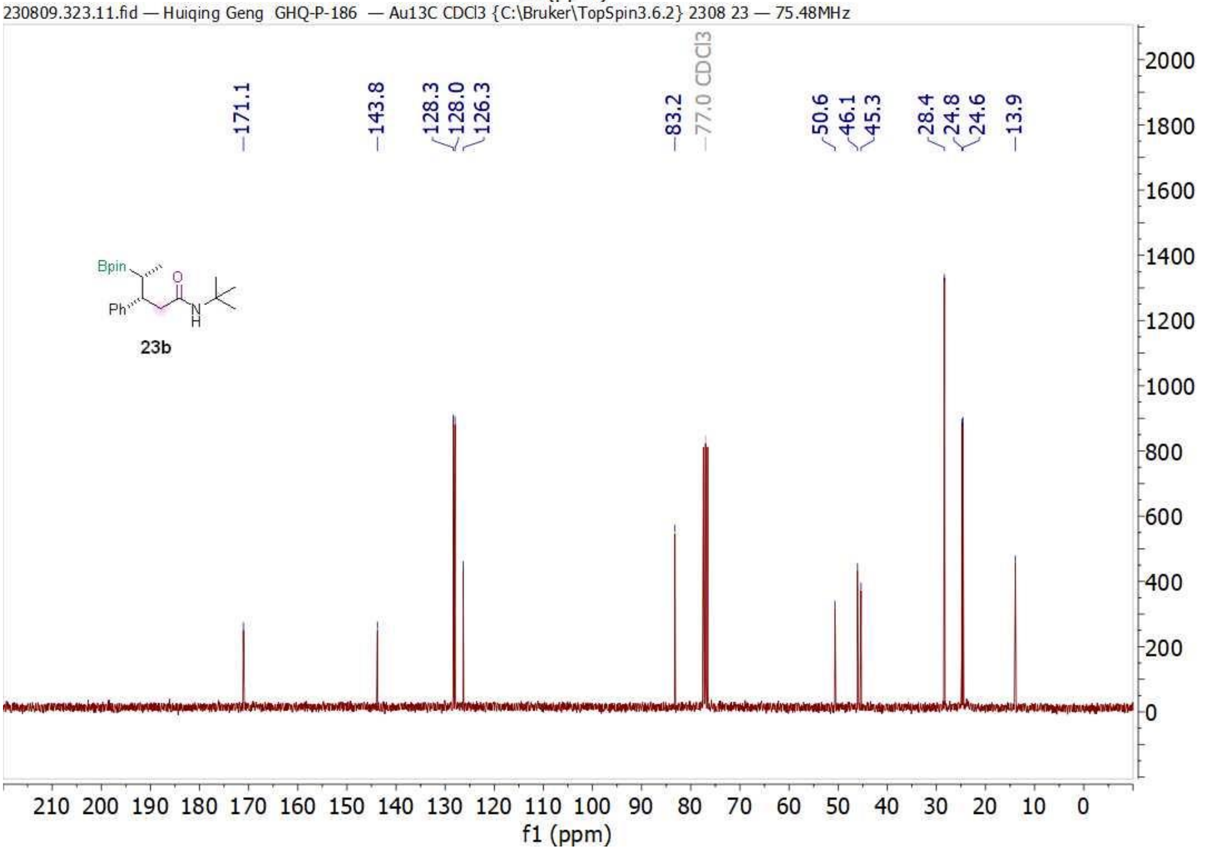
230809.323.11.fid — Huiqing Geng GHQ-P-186 — Au13C CDCl3 {C:\Bruker\TopSpin3.6.2} 2308 23 — 75.48MHz
171.1
143.8
128.3
128.0
126.3
83.2
77.0 CDCl3
50.6
46.1
45.3
28.4
24.8
24.6
13.9
23b
f1 (ppm)

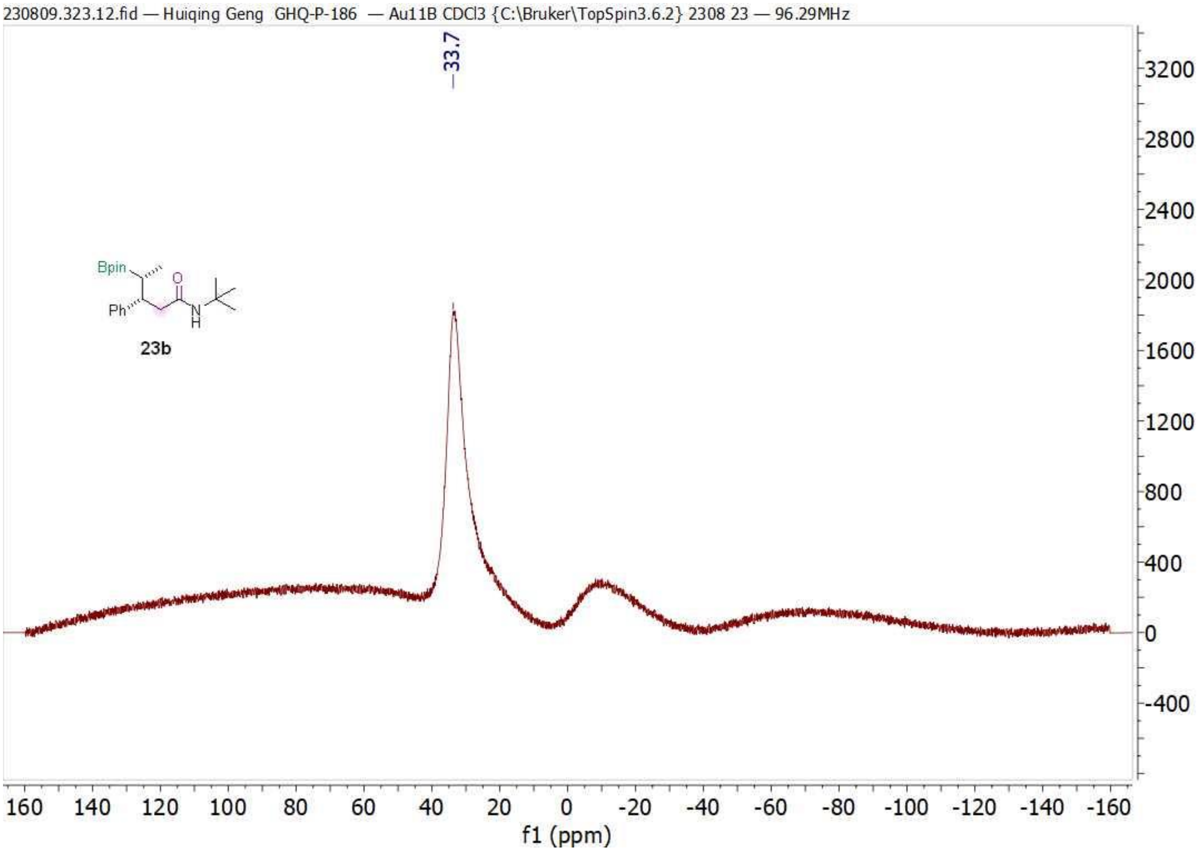
230809.323.12.fid — Huiqing Geng GHQ-P-186 — Au11B CDCl3 {C:\Bruker\TopSpin3.6.2} 2308 23 — 96.29MHz
33.7
Bpin
O
Ph
N
H
23b
3200
2800
2400
2000
1600
1200
800
400
0
-400
160 140 120 100 80 60 40 20 0 -20 -40 -60 -80 -100 -120 -140 -160
f1 (ppm)

231124.f337.10.fid — ghq-p-398 — Au1H CDCl3 {C:\Bruker\TopSpin3.6.2} 2311 37 — 300.20MHz

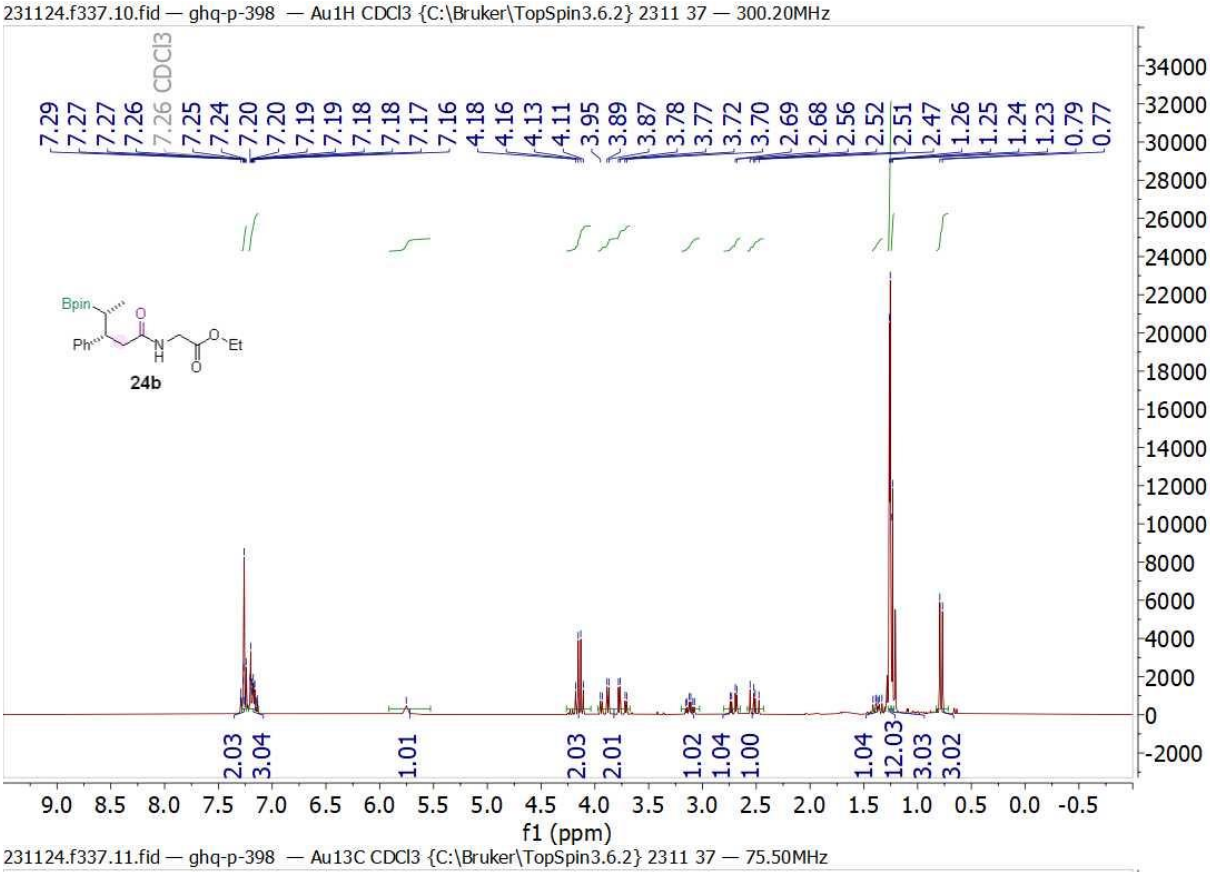

231124.f337.11.fid — ghq-p-398 — Au13C CDCl3 {C:\Bruker\TopSpin3.6.2} 2311 37 — 75.50MHz

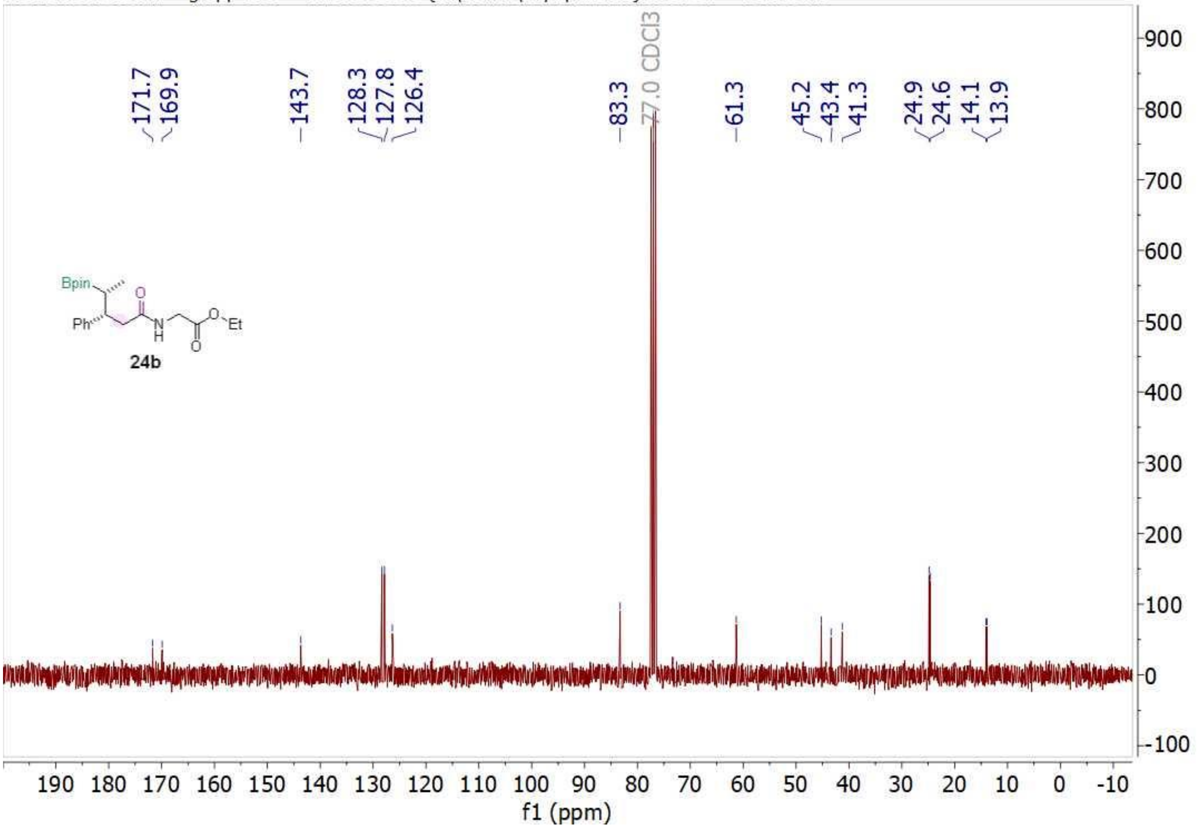

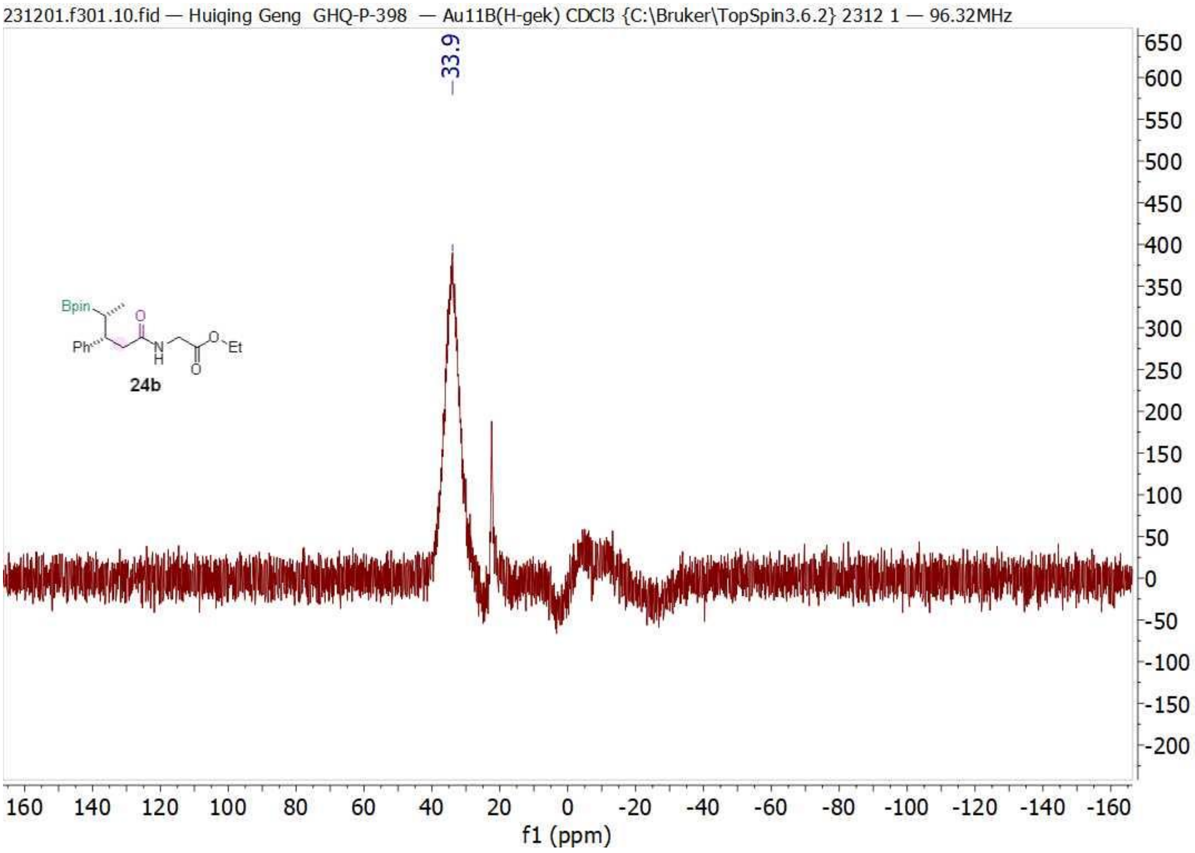
231201.f301.10.fid — Huiqing Geng GHQ-P-398 — Au11B(H-gek) CDCl3 {C:\Bruker\TopSpin3.6.2} 2312 1 — 96.32MHz
33.9
Bpin
O
Ph
N
H
O
Et
O
24b
f1 (ppm)

230616.315.10.fid — Huiqing Geng GHQ-P-110 — Au1H CDCl3 {C:\Bruker\TopSpin3.6.2} 2306 15 — 300.13MHz

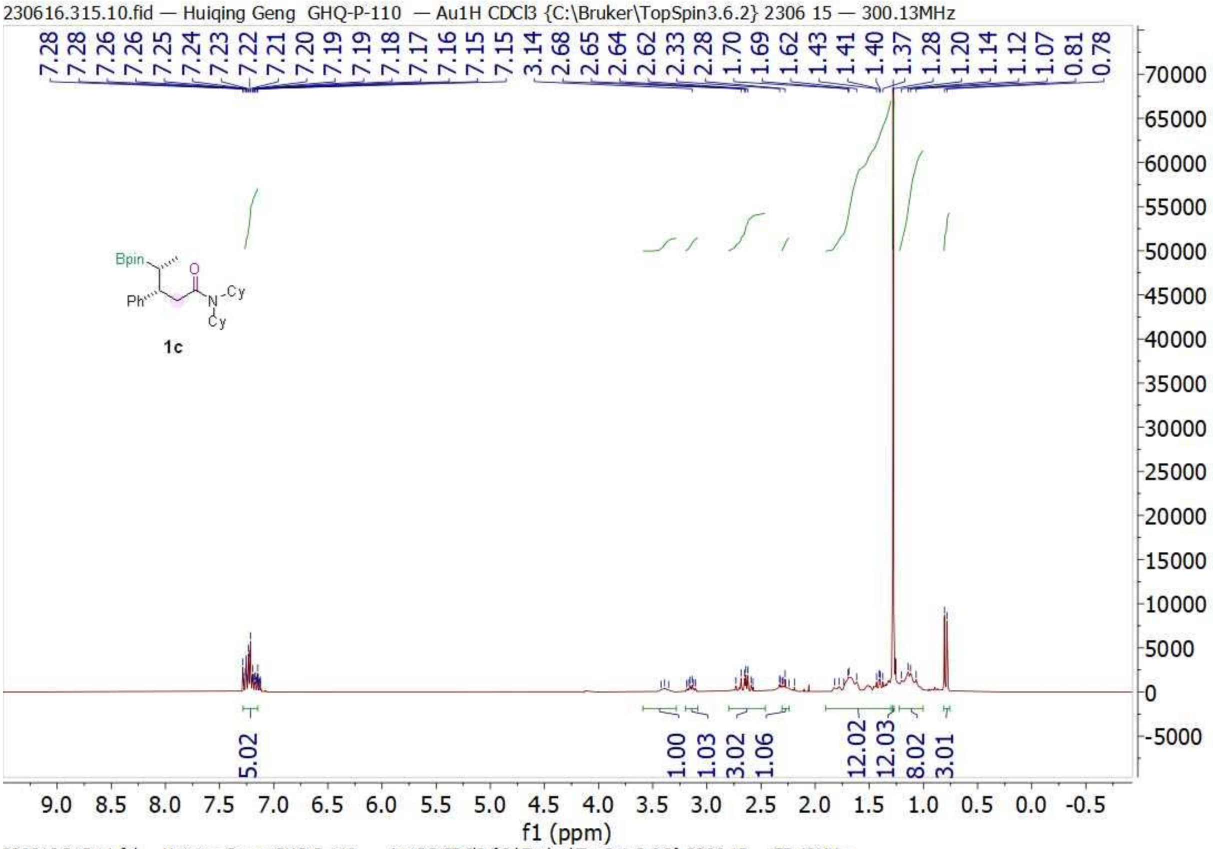

230616.315.11.fid — Huiqing Geng GHQ-P-110 — Au13C CDCl3 {C:\Bruker\TopSpin3.6.2} 2306 15 — 75.48MHz

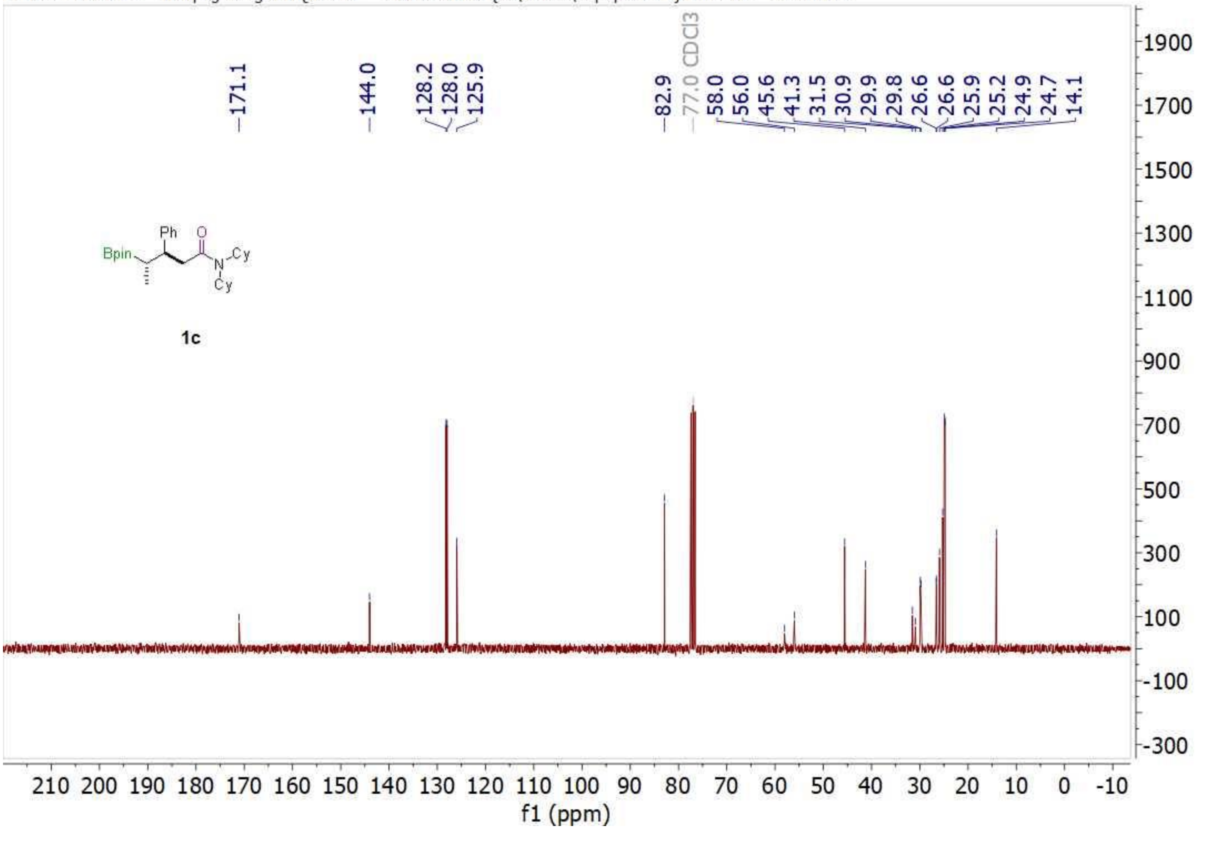

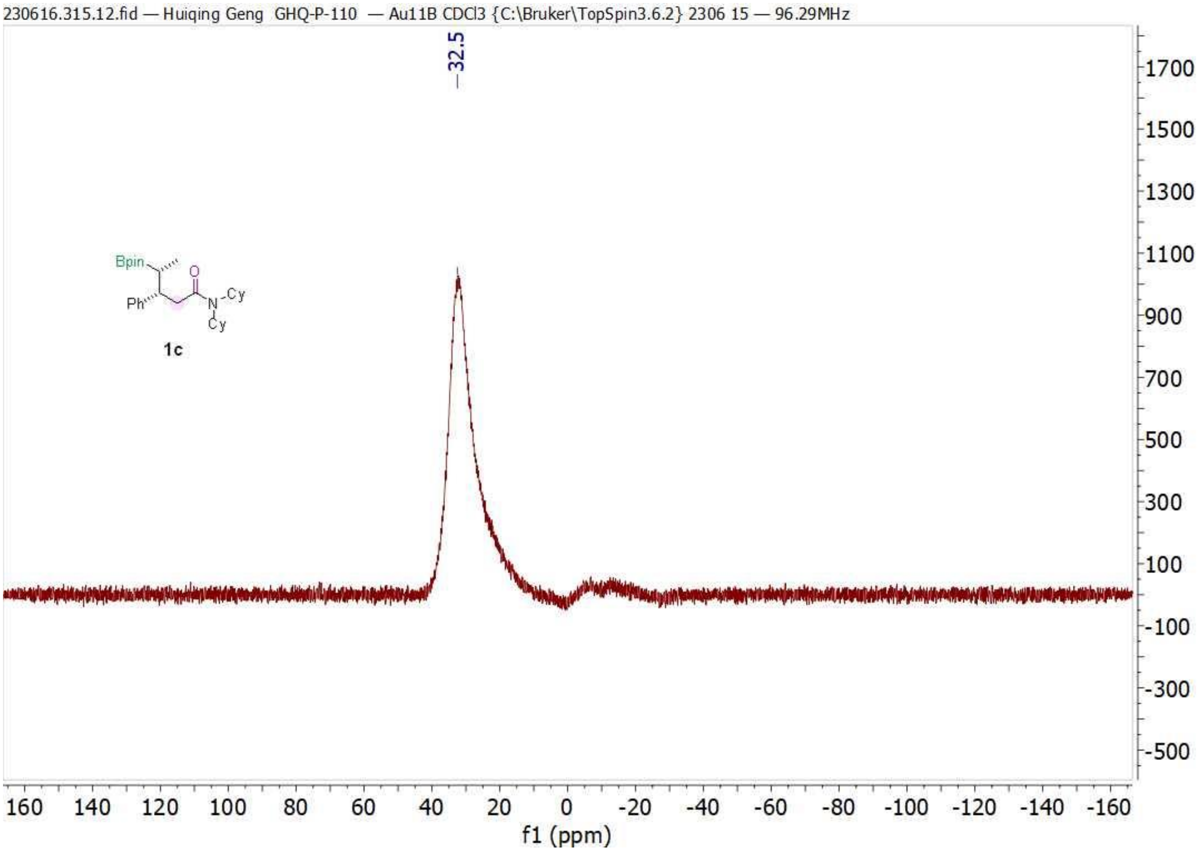
230616.315.12.fid — Huiqing Geng GHQ-P-110 — Au11B CDCl3 {C:\Bruker\TopSpin3.6.2} 2306 15 — 96.29MHz
32.5
Bpin
Ph
O
N
Cy
Cy
1c
f1 (ppm)

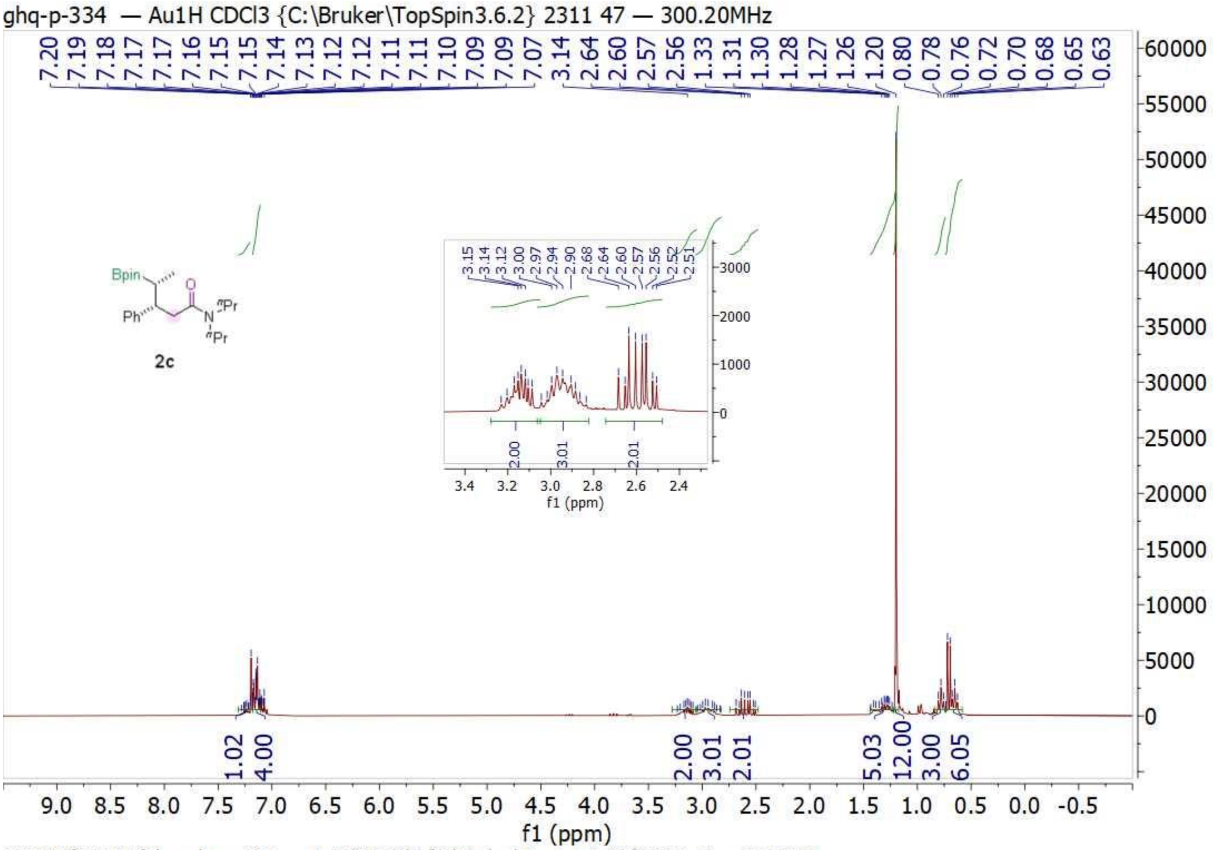
ghq-p-334 — Au1H CDCl3 {C:\Bruker\TopSpin3.6.2} 2311 47 — 300.20MHz
2c
f1 (ppm)

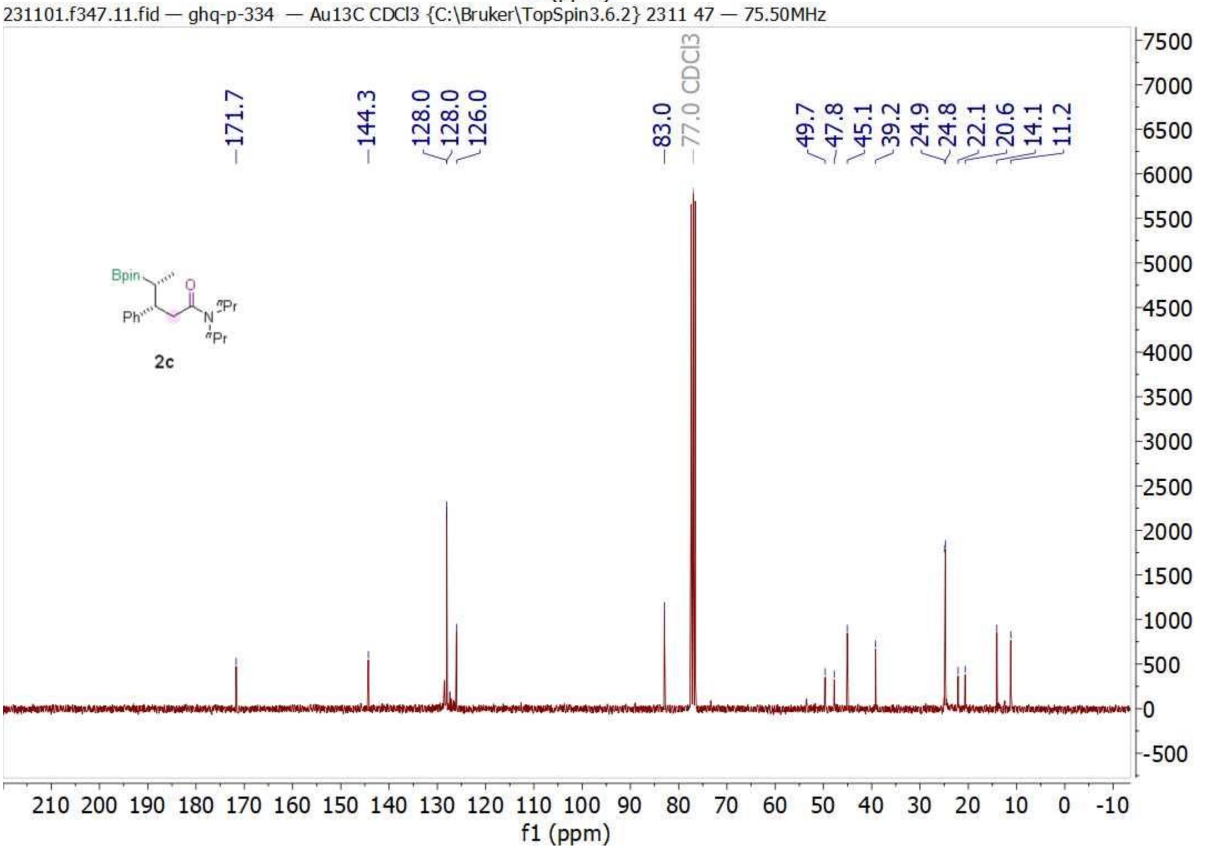
231101.f347.11.fid — ghq-p-334 — Au13C CDCl3 {C:\Bruker\TopSpin3.6.2} 2311 47 — 75.50MHz
171.7
144.3
128.0
128.0
126.0
83.0
77.0 CDCl3
49.7
47.8
45.1
39.2
24.9
24.8
22.1
20.6
14.1
11.2
2c
f1 (ppm)

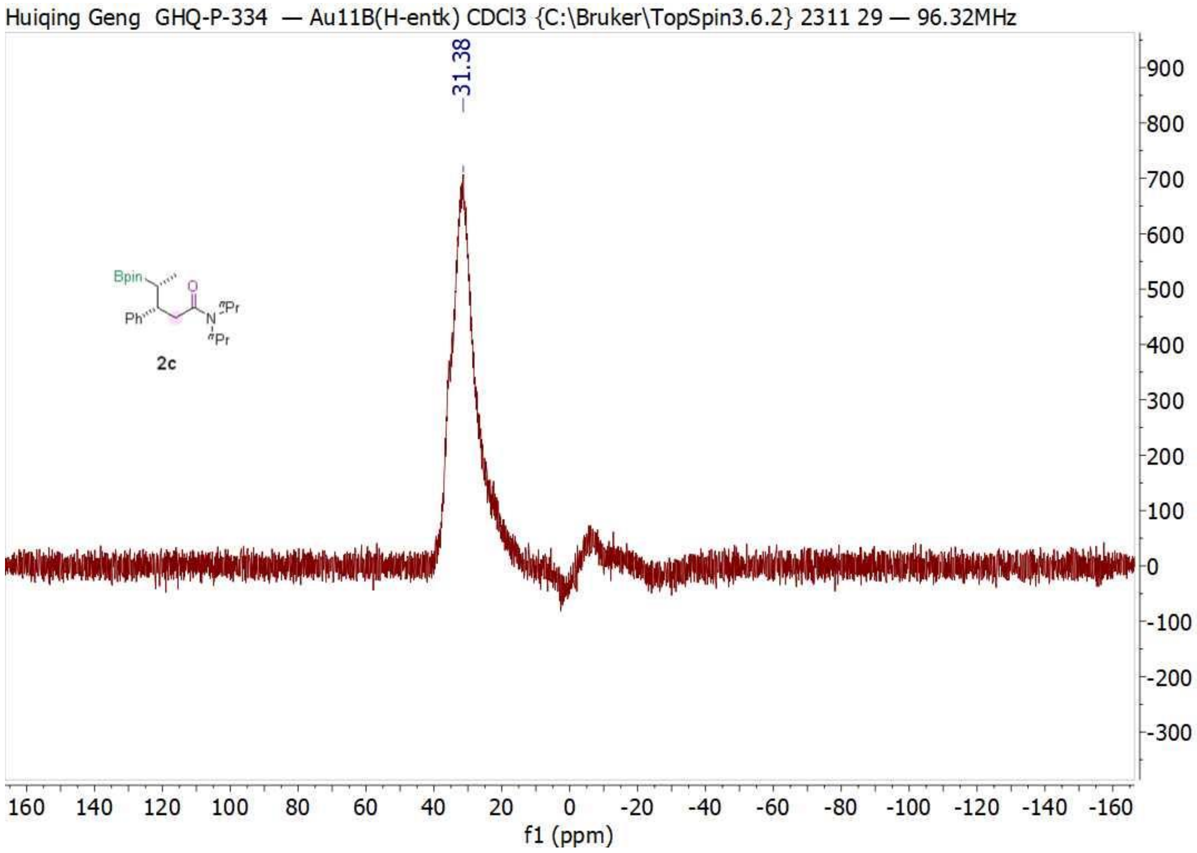
Huiqing Geng GHQ-P-334 — Au11B(H-entk) CDCl3 {C:\Bruker\TopSpin3.6.2} 2311 29 — 96.32MHz
31.38
Bpin
O
Ph
N
nPr
nPr
2c
f1 (ppm)

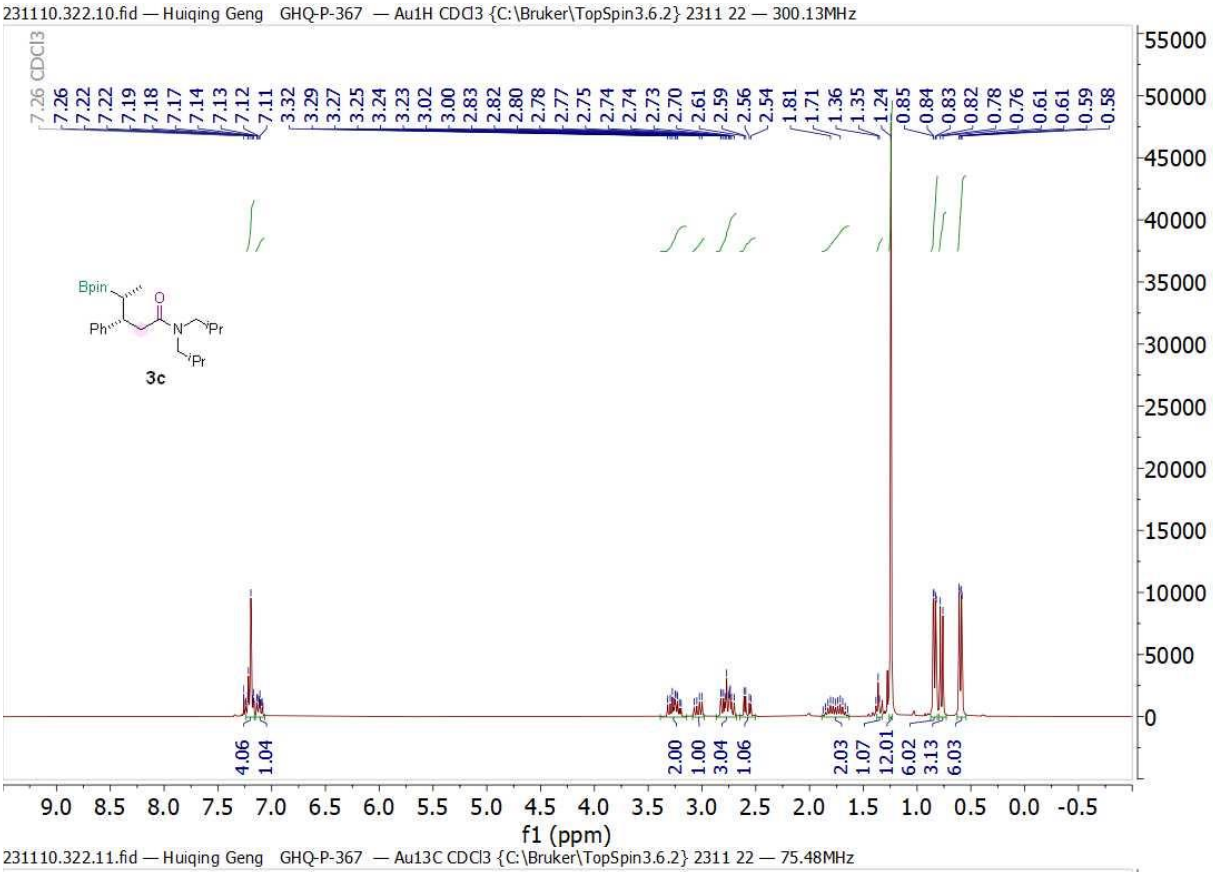
231110.322.10.fid — Huiqing Geng GHQ-P-367 — Au1H CDCl3 {C:\Bruker\TopSpin3.6.2} 2311 22 — 300.13MHz
3c
f1 (ppm)

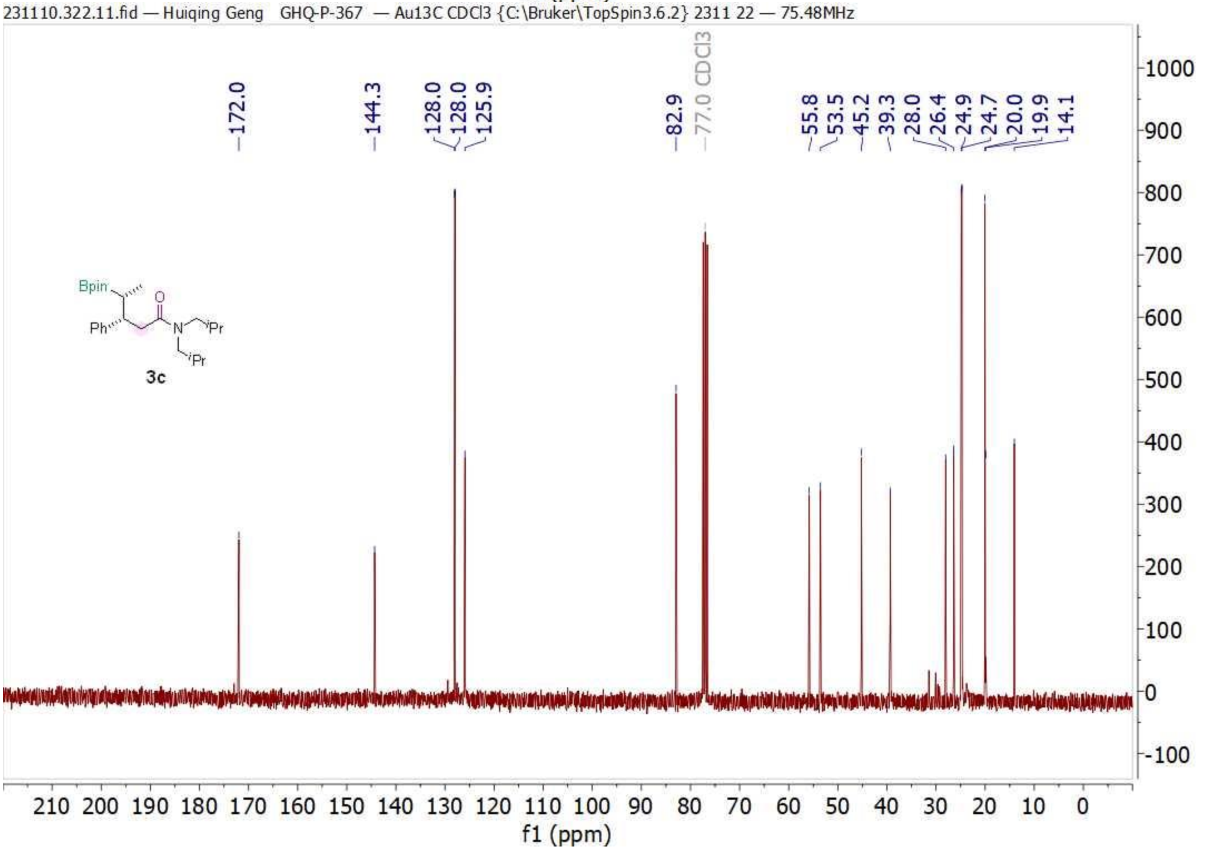
231110.322.11.fid — Huiqing Geng GHQ-P-367 — Au13C CDCl3 {C:\Bruker\TopSpin3.6.2} 2311 22 — 75.48MHz
3c
f1 (ppm)

231110.322.12.fid — Huiqing Geng GHQ-P-367 — Au11B CDCl3 {C:\Bruker\TopSpin3.6.2} 2311 22 — 96.29MHz

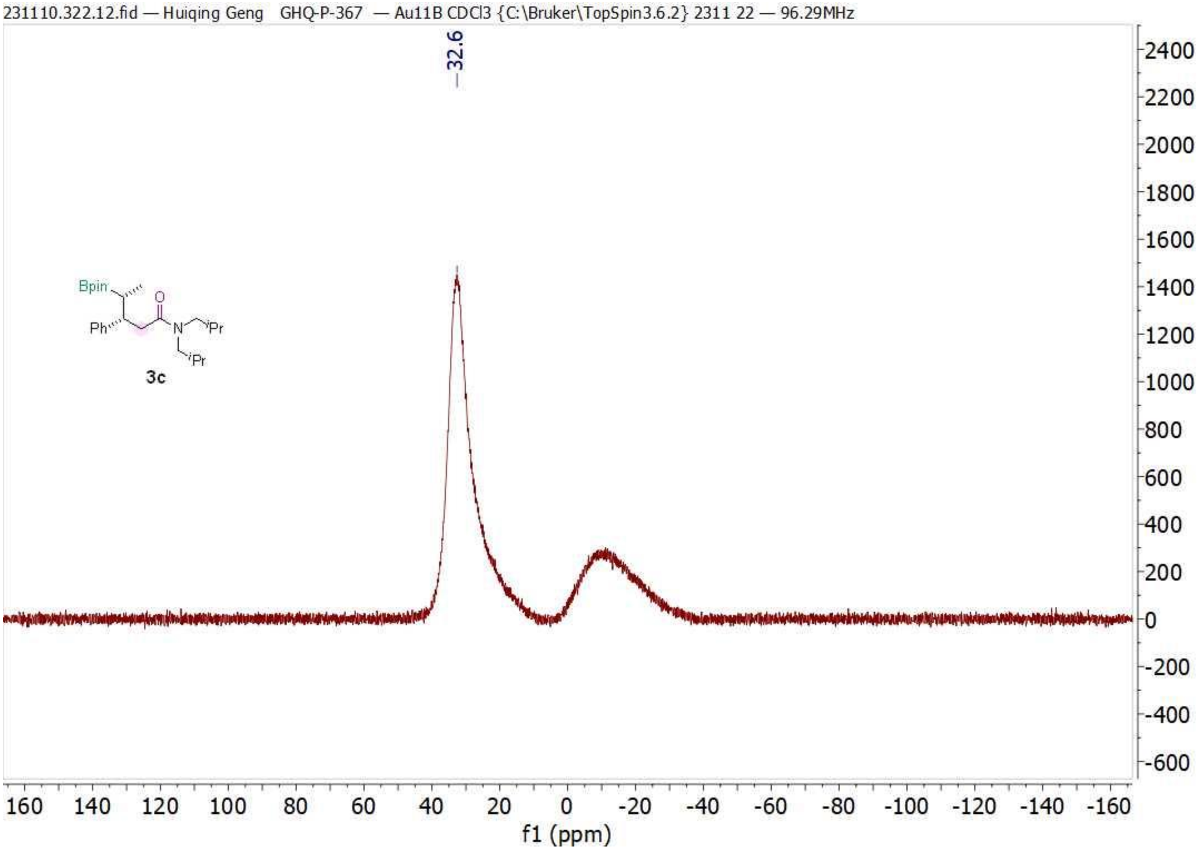

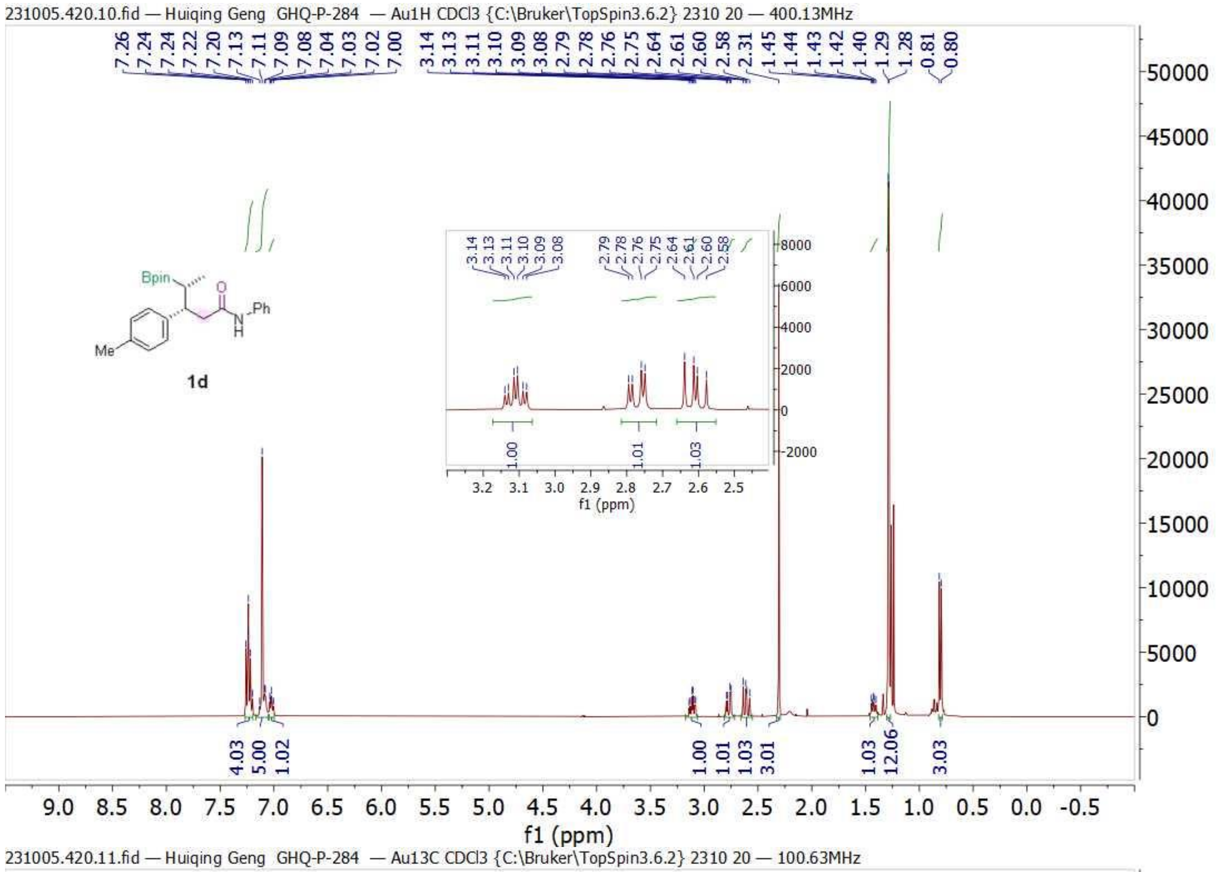
231005.420.10.fid — Huiqing Geng GHQ-P-284 — Au1H CDCl3 {C:\Bruker\TopSpin3.6.2} 2310 20 — 400.13MHz
7.26 7.24 7.24 7.22 7.20 7.13 7.11 7.09 7.08 7.04 7.03 7.02 7.00 3.14 3.13 3.11 3.10 3.09 3.08 2.79 2.78 2.76 2.75 2.64 2.61 2.60 2.58 2.31 1.45 1.44 1.43 1.42 1.40 1.29 1.28 0.81 0.80
Bpin
O
N
H
Ph
Me
1d
4.03 5.00 1.02 1.00 1.01 1.03 3.01 1.03 12.06 3.03
f1 (ppm)

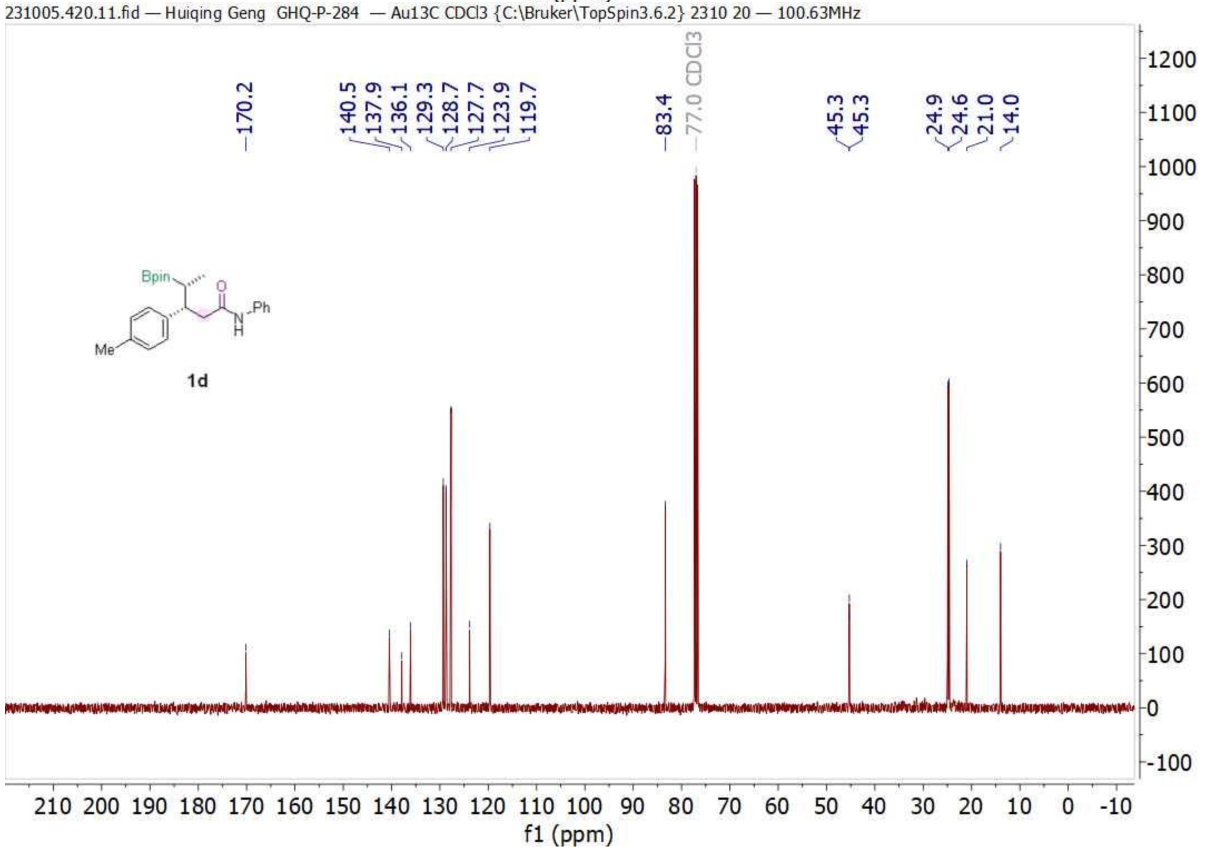
231005.420.11.fid — Huiqing Geng GHQ-P-284 — Au13C CDCl3 {C:\Bruker\TopSpin3.6.2} 2310 20 — 100.63MHz
170.2 140.5 137.9 136.1 129.3 128.7 127.7 123.9 119.7 83.4 77.0 CDCl3 45.3 45.3 24.9 24.6 21.0 14.0
Bpin
O
N
H
Ph
Me
1d
f1 (ppm)

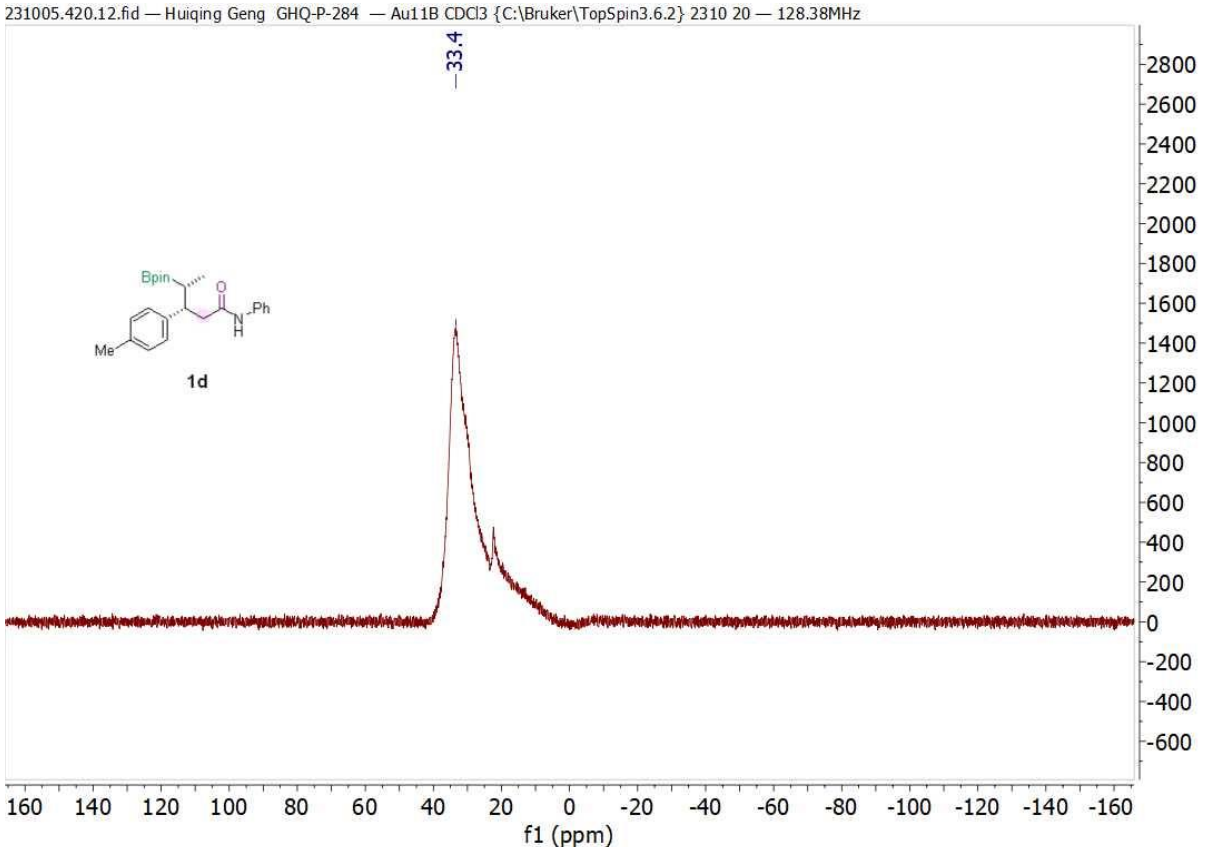
231005.420.12.fid — Huiqing Geng GHQ-P-284 — Au11B CDCl3 {C:\Bruker\TopSpin3.6.2} 2310 20 — 128.38MHz
33.4
Bpin
O
N
H
Ph
Me
1d
f1 (ppm)

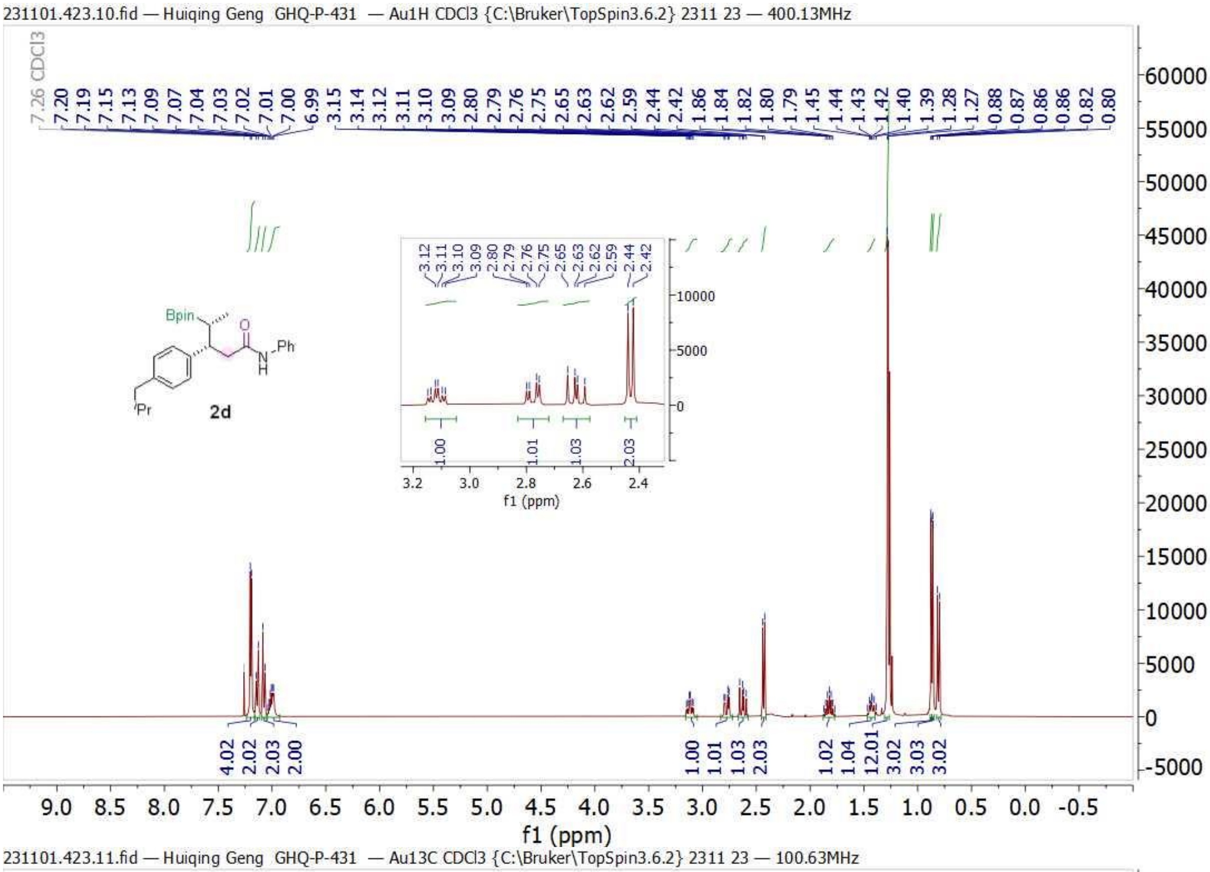
231101.423.10.fid — Huiqing Geng GHQ-P-431 — Au1H CDCl3 {C:\Bruker\TopSpin3.6.2} 2311 23 — 400.13MHz
7.26 CDCl3
7.20 7.19 7.15 7.13 7.09 7.07 7.04 7.03 7.02 7.01 7.00 6.99 3.15 3.14 3.12 3.11 3.10 3.09 2.80 2.79 2.76 2.75 2.65 2.63 2.62 2.59 2.44 2.42 1.86 1.84 1.82 1.80 1.79 1.45 1.44 1.43 1.42 1.40 1.39 1.28 1.27 0.88 0.87 0.86 0.82 0.80
Bpin
O
N
H
Ph
iPr
2d
4.02 2.02 2.03 2.00 1.00 1.01 1.03 2.03 1.02 1.04 12.01 3.02 3.03 3.02
f1 (ppm)

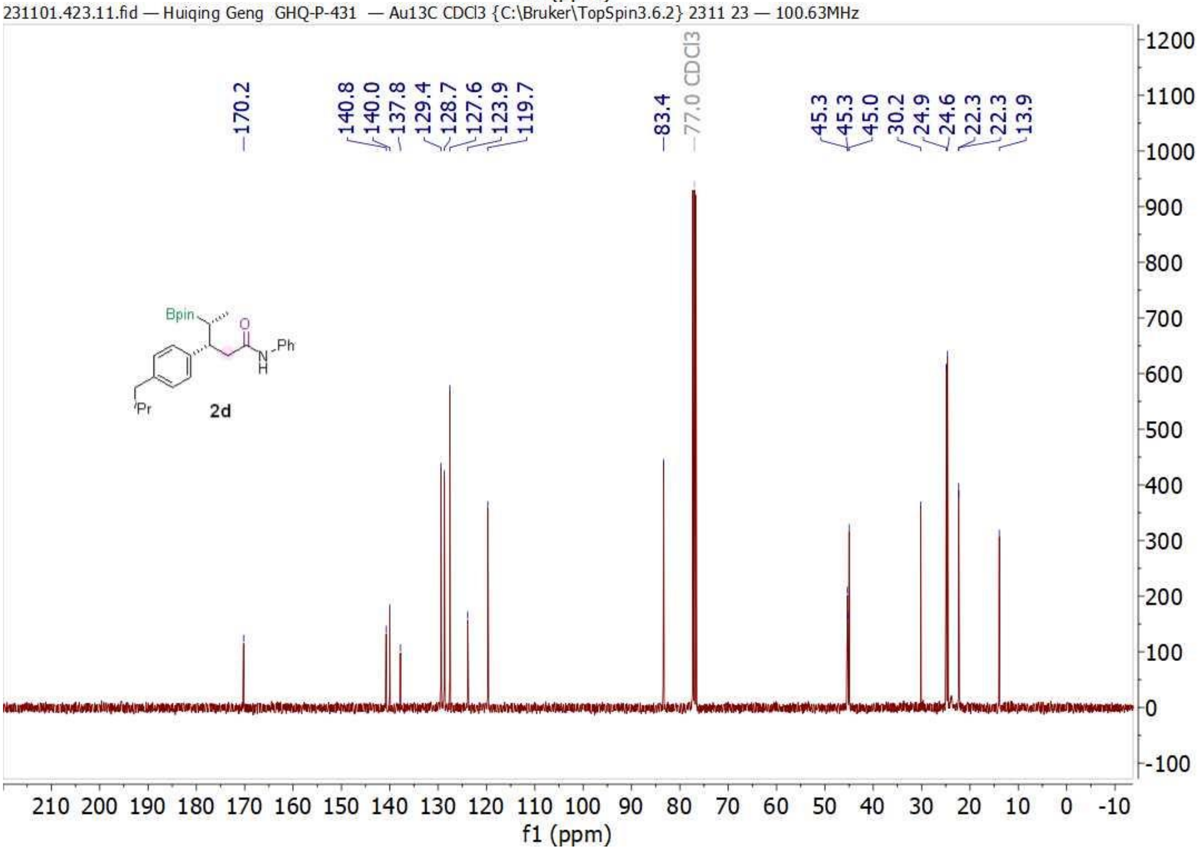
231101.423.11.fid — Huiqing Geng GHQ-P-431 — Au13C CDCl3 {C:\Bruker\TopSpin3.6.2} 2311 23 — 100.63MHz
170.2
140.8 140.0 137.8 129.4 128.7 127.6 123.9 119.7
83.4
77.0 CDCl3
45.3 45.3 45.0 30.2 24.9 24.6 22.3 22.3 13.9
Bpin
O
N
H
Ph
iPr
2d
f1 (ppm)

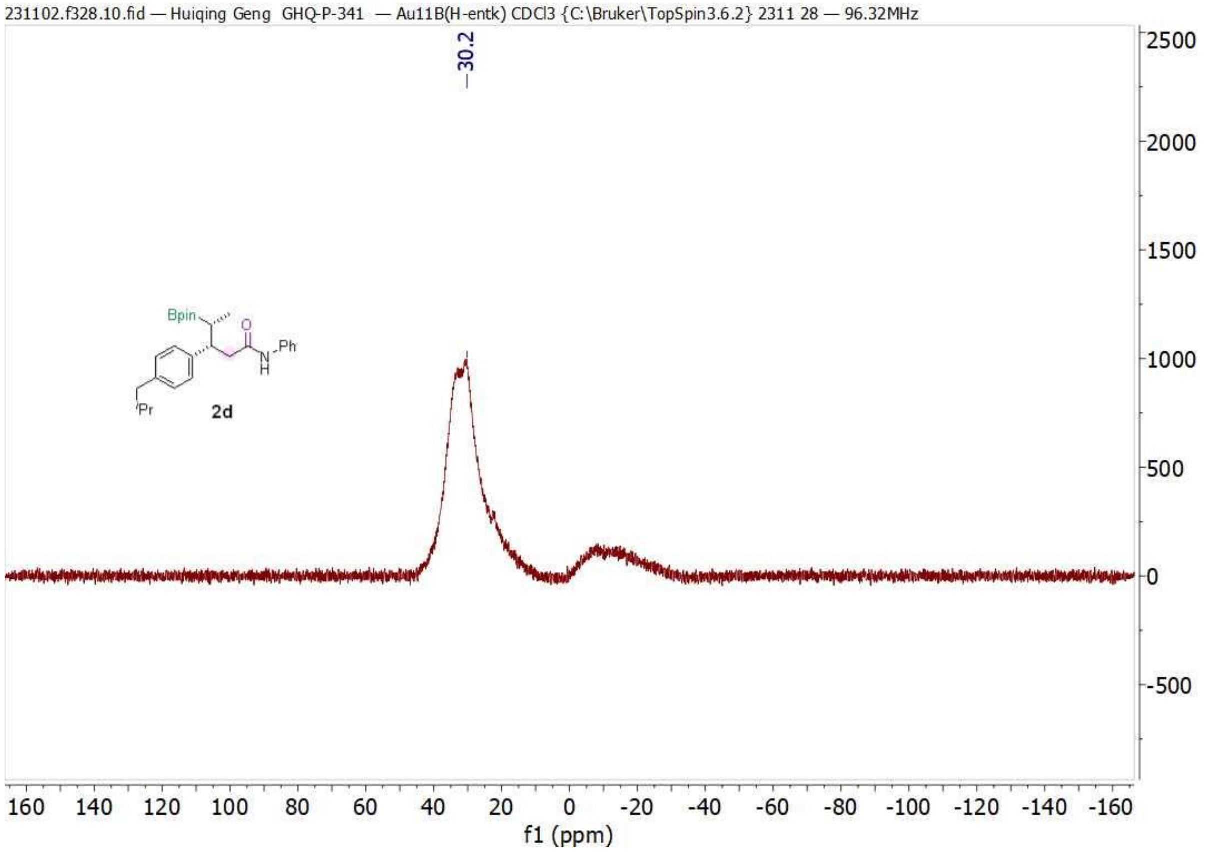

231102.f328.10.fid — Huiqing Geng GHQ-P-341 — Au11B(H-entk) CDCl3 {C:\Bruker\TopSpin3.6.2} 2311 28 — 96.32MHz
30.2
Bpin
O
N
H
Ph
iPr
2d
2500
2000
1500
1000
500
0
-500
160 140 120 100 80 60 40 20 0 -20 -40 -60 -80 -100 -120 -140 -160
f1 (ppm)

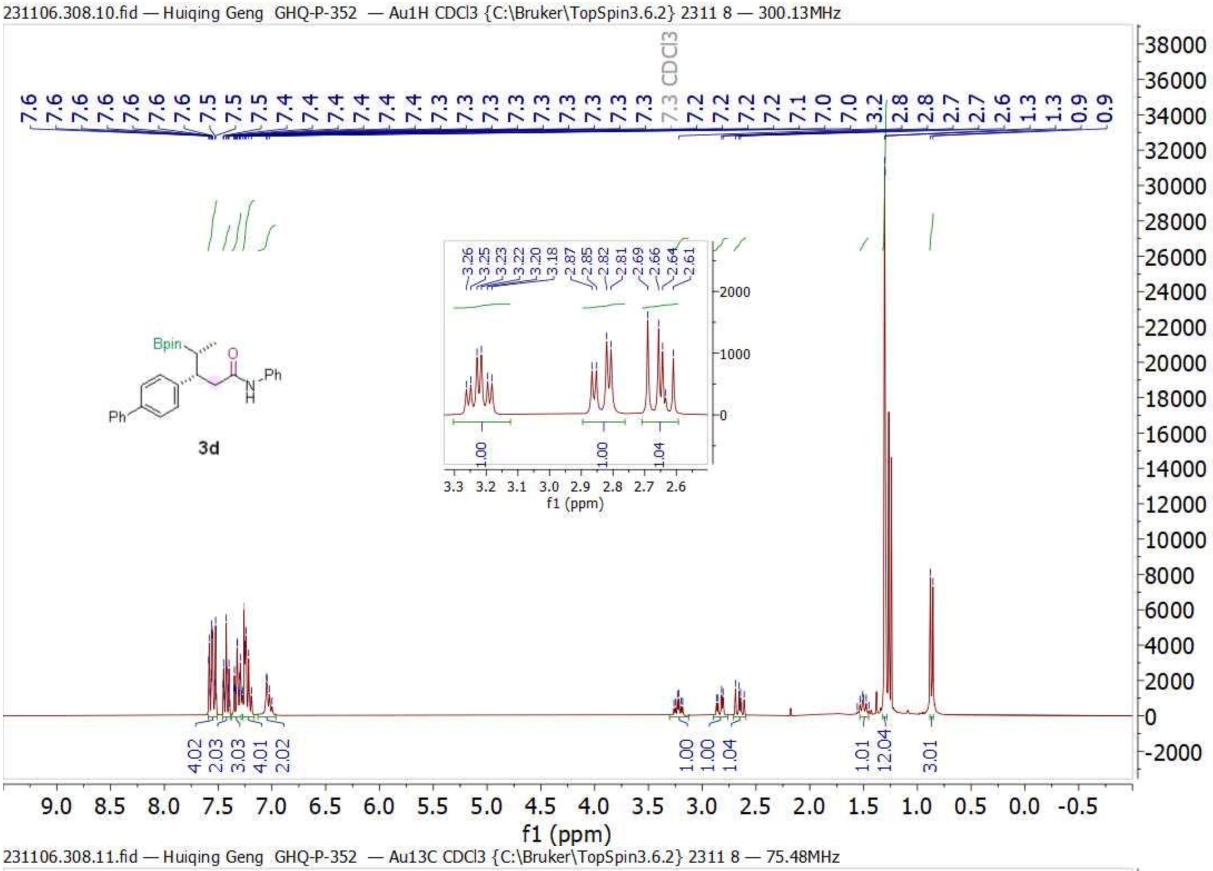
231106.308.10.fid — Huiqing Geng GHQ-P-352 — Au1H CDCl3 {C:\Bruker\TopSpin3.6.2} 2311 8 — 300.13MHz
CDCl3
3d
Bpin
Ph
f1 (ppm)

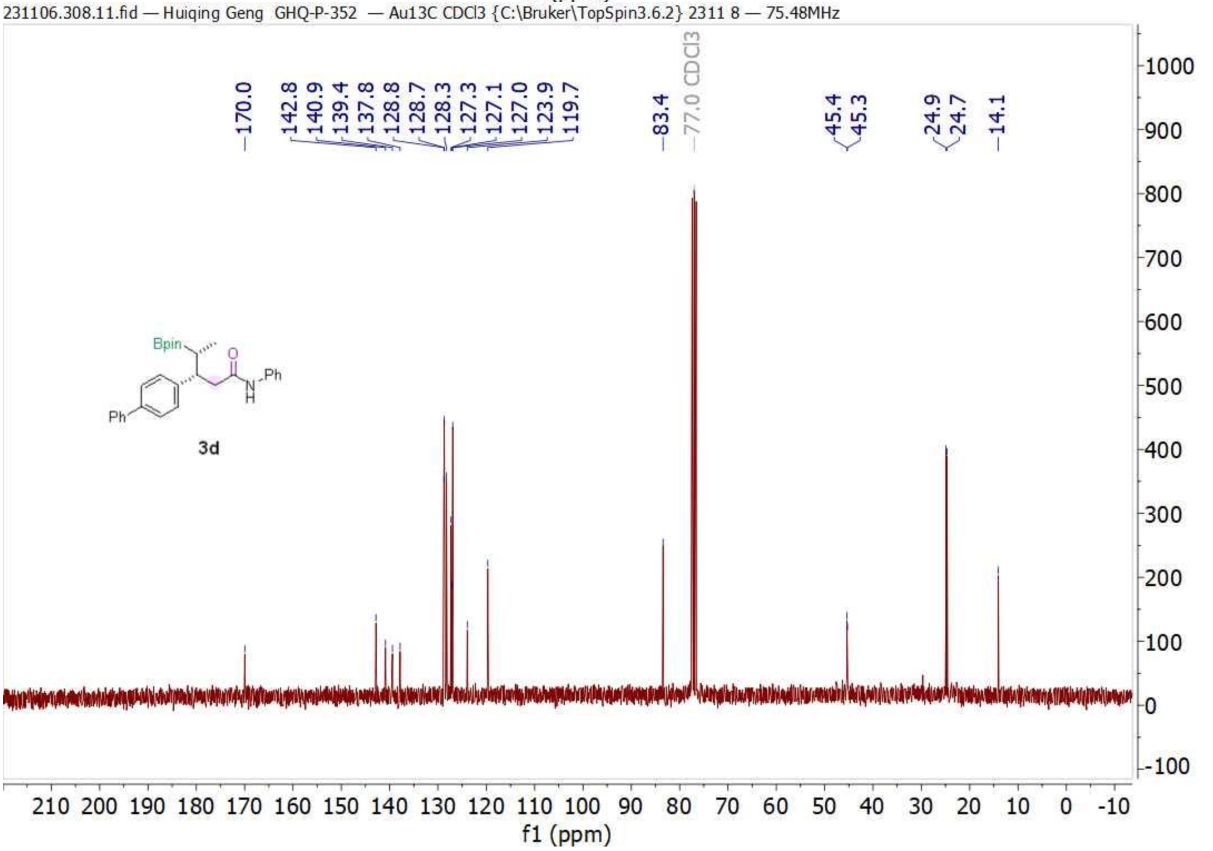
231106.308.11.fid — Huiqing Geng GHQ-P-352 — Au13C CDCl3 {C:\Bruker\TopSpin3.6.2} 2311 8 — 75.48MHz
170.0
142.8
140.9
139.4
137.8
128.8
128.7
128.3
127.3
127.1
127.0
123.9
119.7
83.4
77.0 CDCl3
45.4
45.3
24.9
24.7
14.1
3d
Bpin
Ph
f1 (ppm)

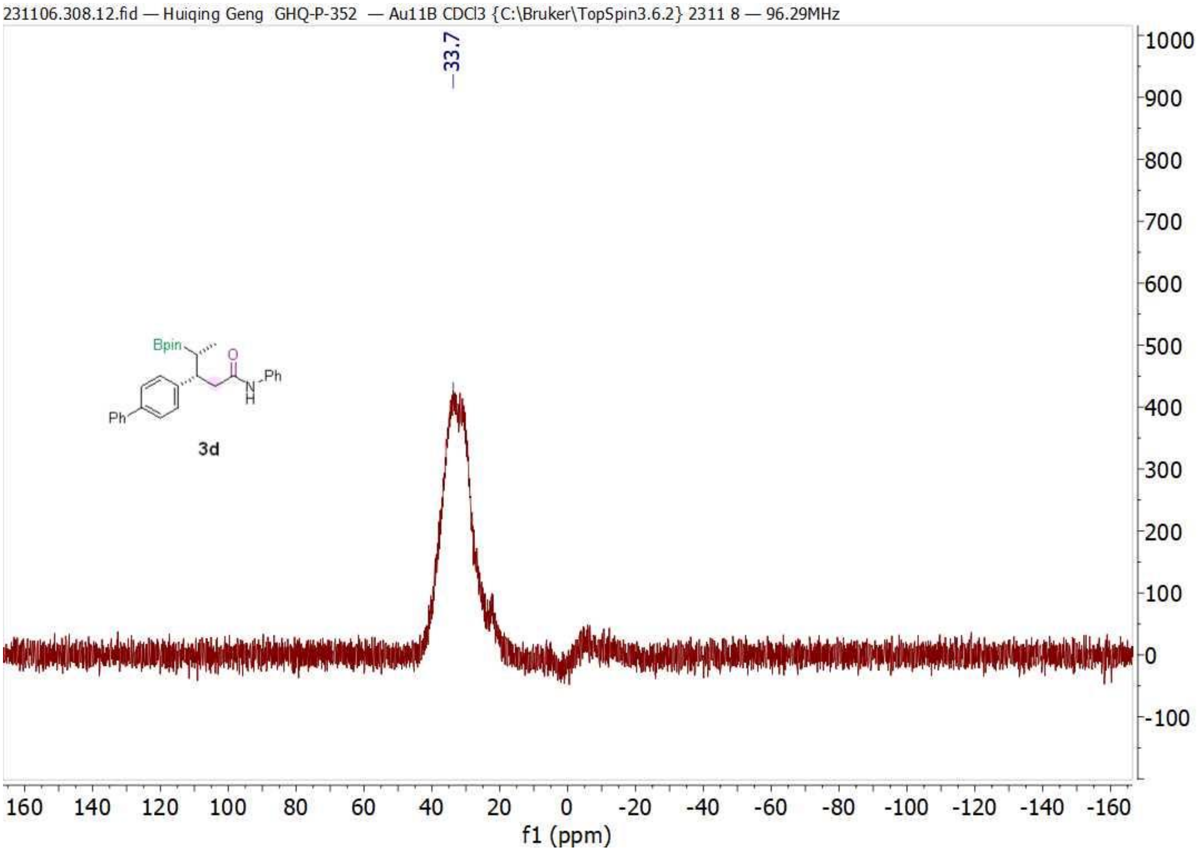
231106.308.12.fid — Huiqing Geng GHQ-P-352 — Au11B CDCl3 {C:\Bruker\TopSpin3.6.2} 2311 8 — 96.29MHz
—33.7
Bpin
O
N
H
Ph
Ph
f1 (ppm)

3d

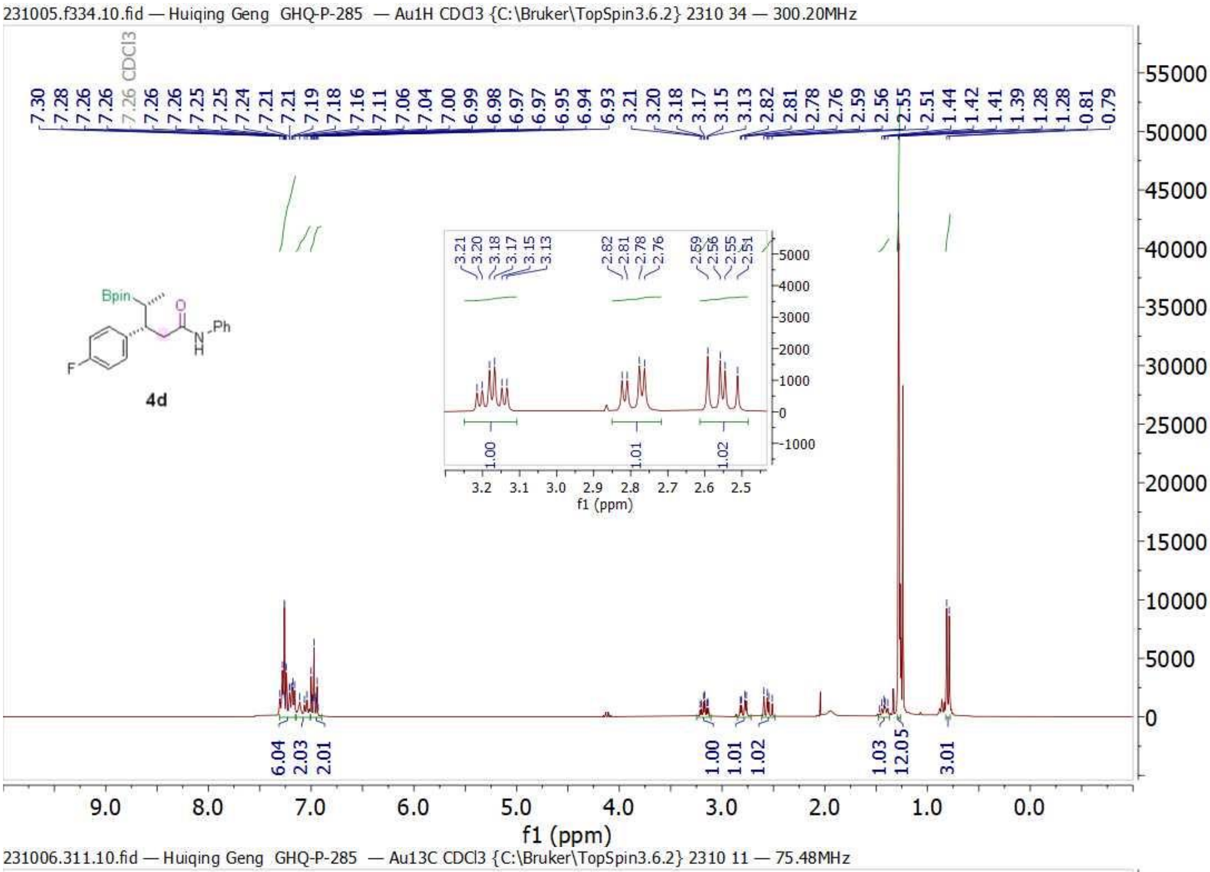
231005.f334.10.fid — Huiqing Geng GHQ-P-285 — Au1H CDCl3 {C:\Bruker\TopSpin3.6.2} 2310 34 — 300.20MHz
7.30 7.28 7.26 7.26 CDCl3 7.26 7.26 7.25 7.25 7.24 7.21 7.21 7.19 7.18 7.16 7.11 7.06 7.04 7.00 6.99 6.98 6.97 6.97 6.95 6.94 6.93 3.21 3.20 3.18 3.17 3.15 3.13 2.82 2.81 2.78 2.76 2.59 2.56 2.55 2.51 1.44 1.42 1.41 1.39 1.28 0.81 0.79
Bpin
O
N
H
Ph
F
4d
6.04 2.03 2.01 1.00 1.01 1.02 1.03 12.05 3.01
f1 (ppm)

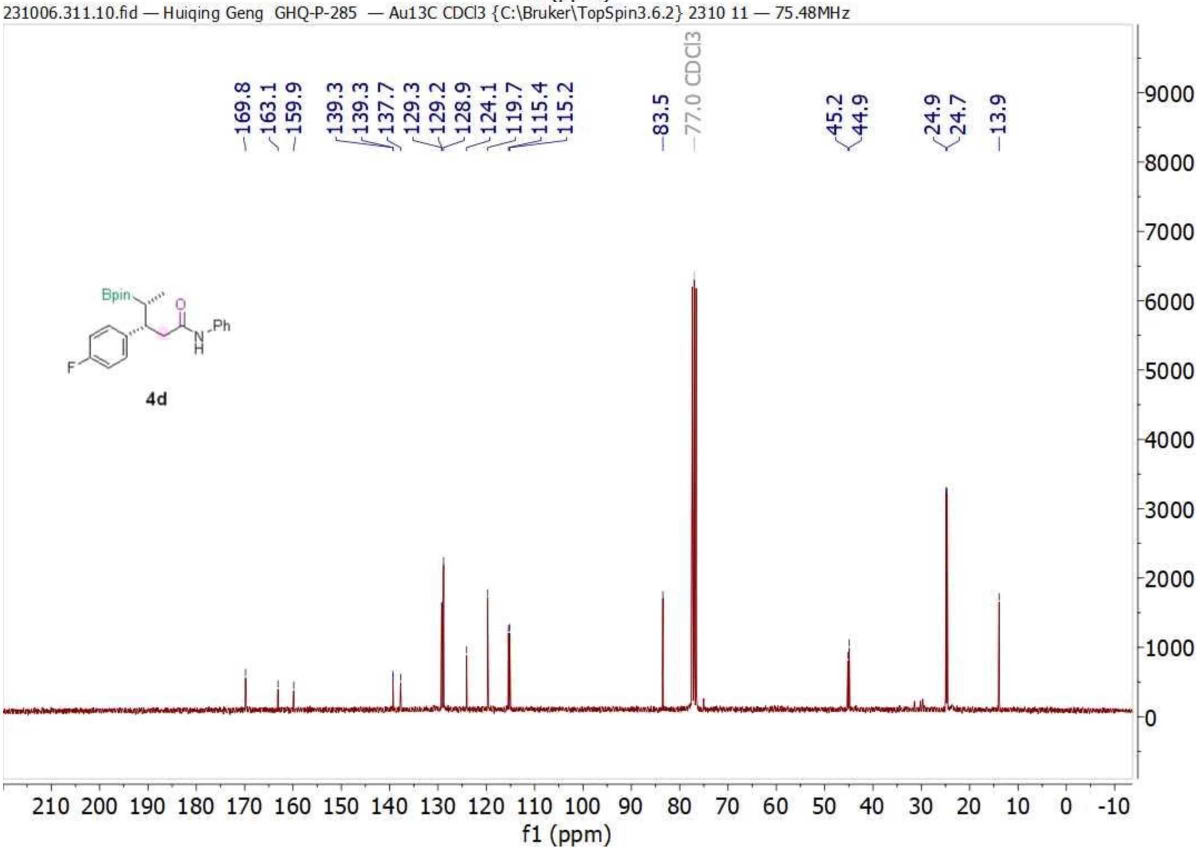
231006.311.10.fid — Huiqing Geng GHQ-P-285 — Au13C CDCl3 {C:\Bruker\TopSpin3.6.2} 2310 11 — 75.48MHz
169.8 163.1 159.9 139.3 139.3 137.7 129.3 129.2 128.9 124.1 119.7 115.4 115.2 83.5 77.0 CDCl3 45.2 44.9 24.9 24.7 13.9
Bpin
O
N
H
Ph
F
4d
f1 (ppm)

231005.f334.12.fid — Huiqing Geng GHQ-P-285 — Au11B(H-gek) CDCl3 {C:\Bruker\TopSpin3.6.2} 2310 34 — 96.32MHz

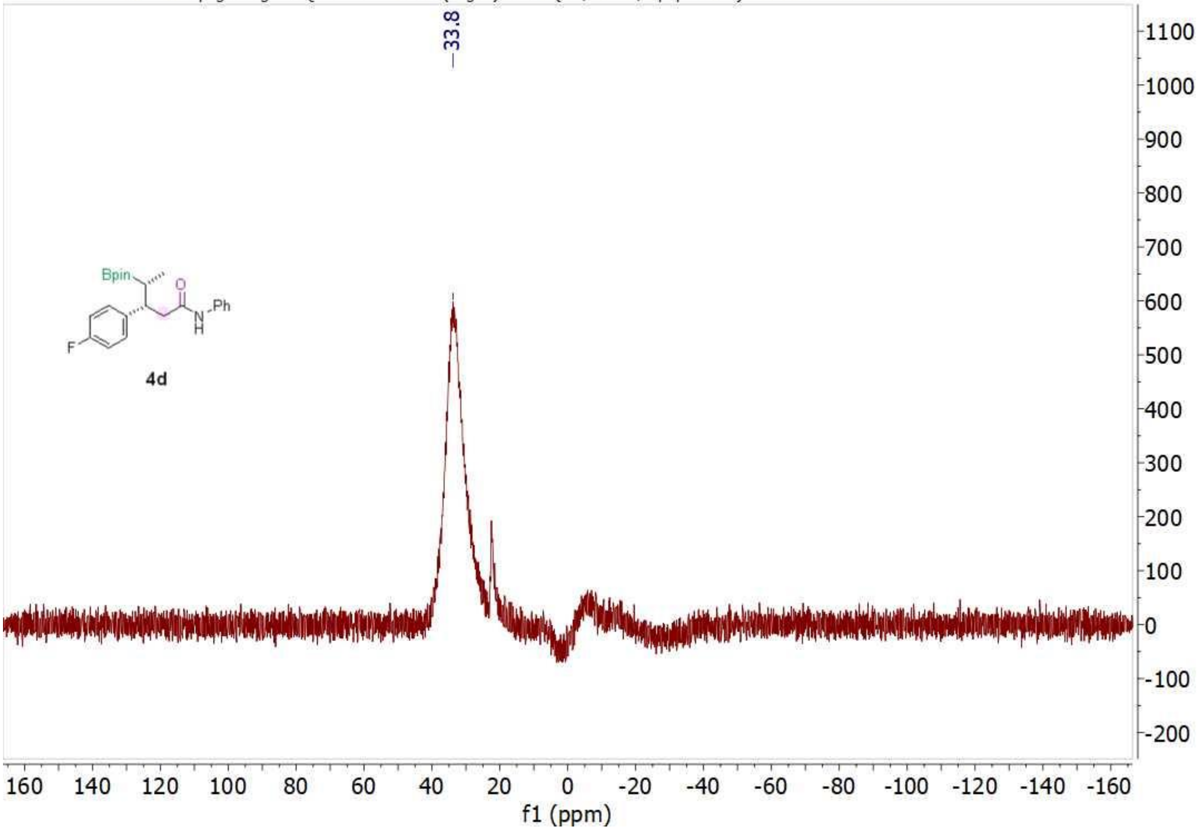

Huiqing Geng GHQ-P-285 — Au19F(H-gek) CDCl3 {C:\Bruker\TopSpin3.6.2} 2310 34 — 282.46MHz

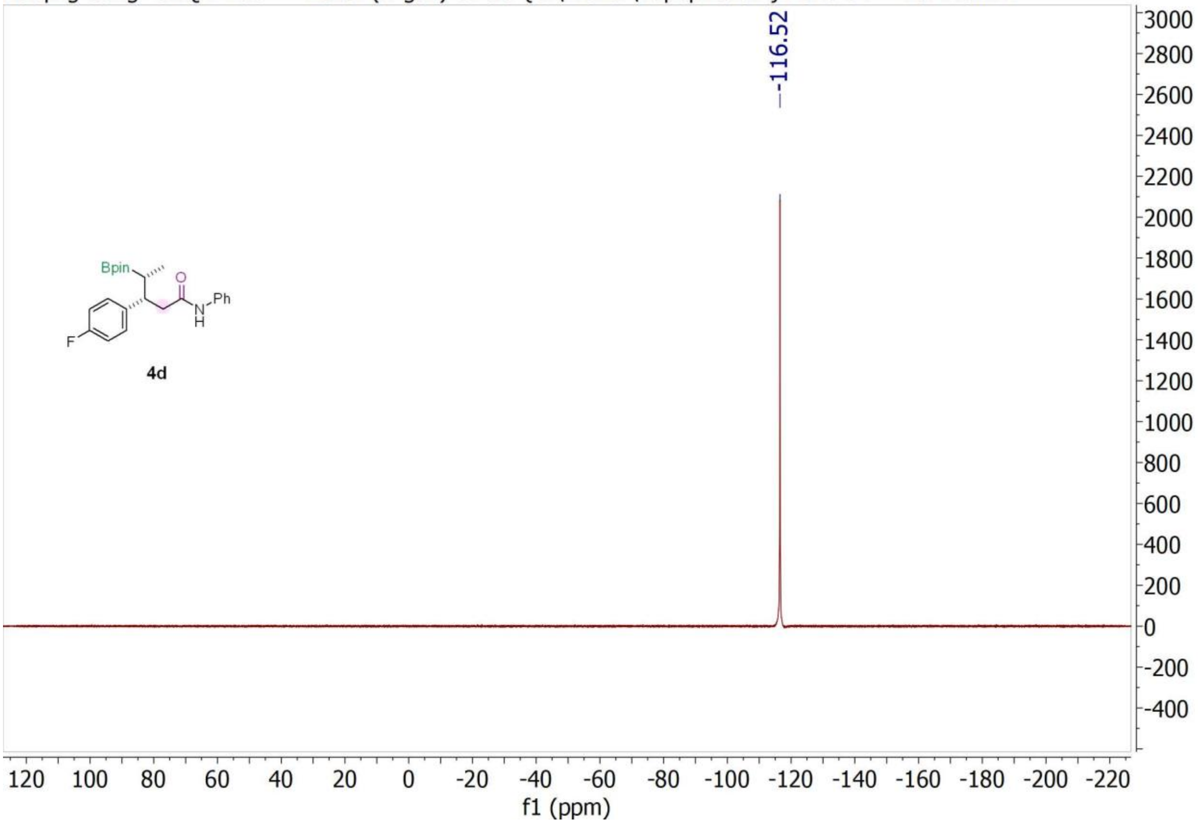

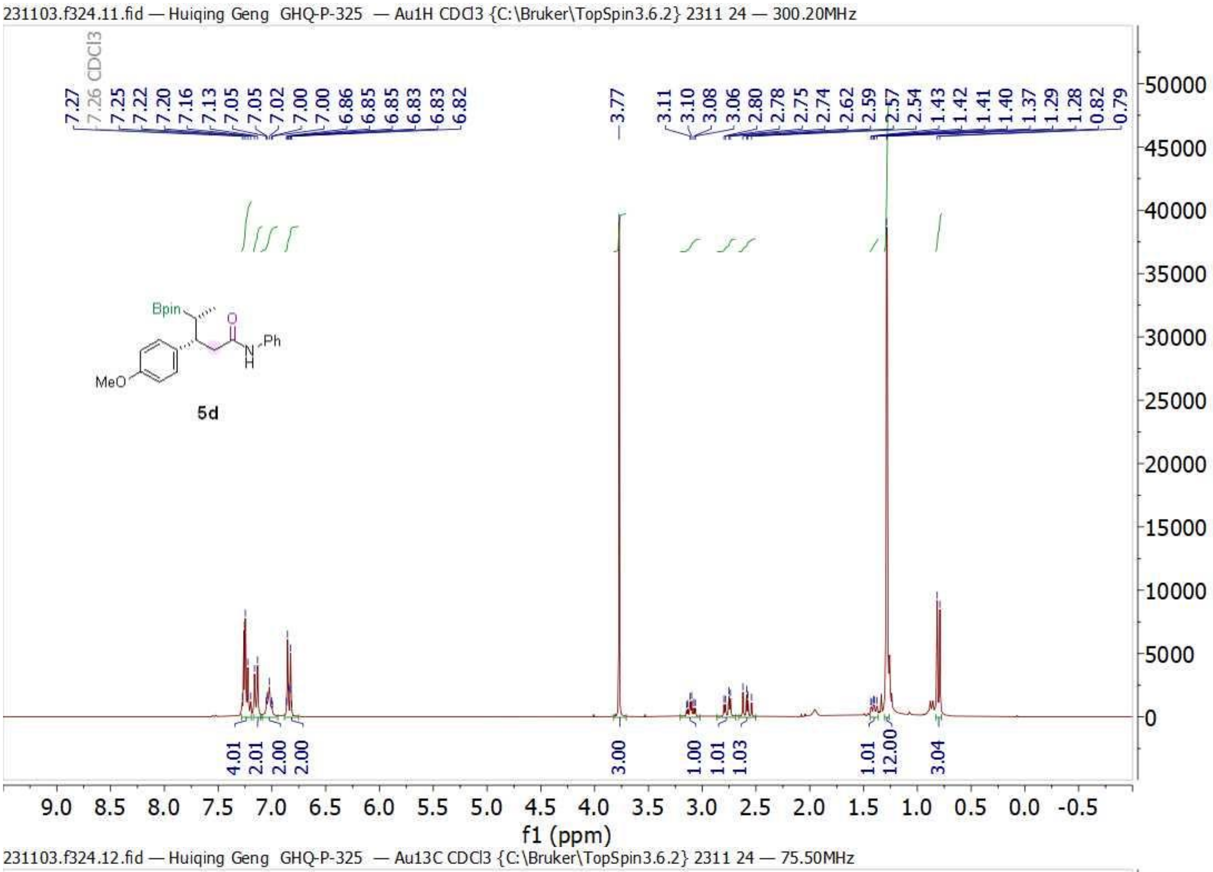
231103.f324.11.fid — Huiqing Geng GHQ-P-325 — Au1H CDCl3 {C:\Bruker\TopSpin3.6.2} 2311 24 — 300.20MHz
7.27 7.26 CDCl3 7.25 7.22 7.20 7.16 7.13 7.05 7.05 7.02 7.00 7.00 6.86 6.85 6.85 6.83 6.83 6.82
3.77
3.11 3.10 3.08 3.06 2.80 2.78 2.75 2.74 2.62 2.59 2.57 2.54 1.43 1.42 1.41 1.40 1.37 1.29 1.28 0.82 0.79
Bpin
O
N
H
Ph
MeO
5d
4.01 2.01 2.00 2.00 3.00 1.00 1.01 1.03 1.01 12.00 3.04
f1 (ppm)

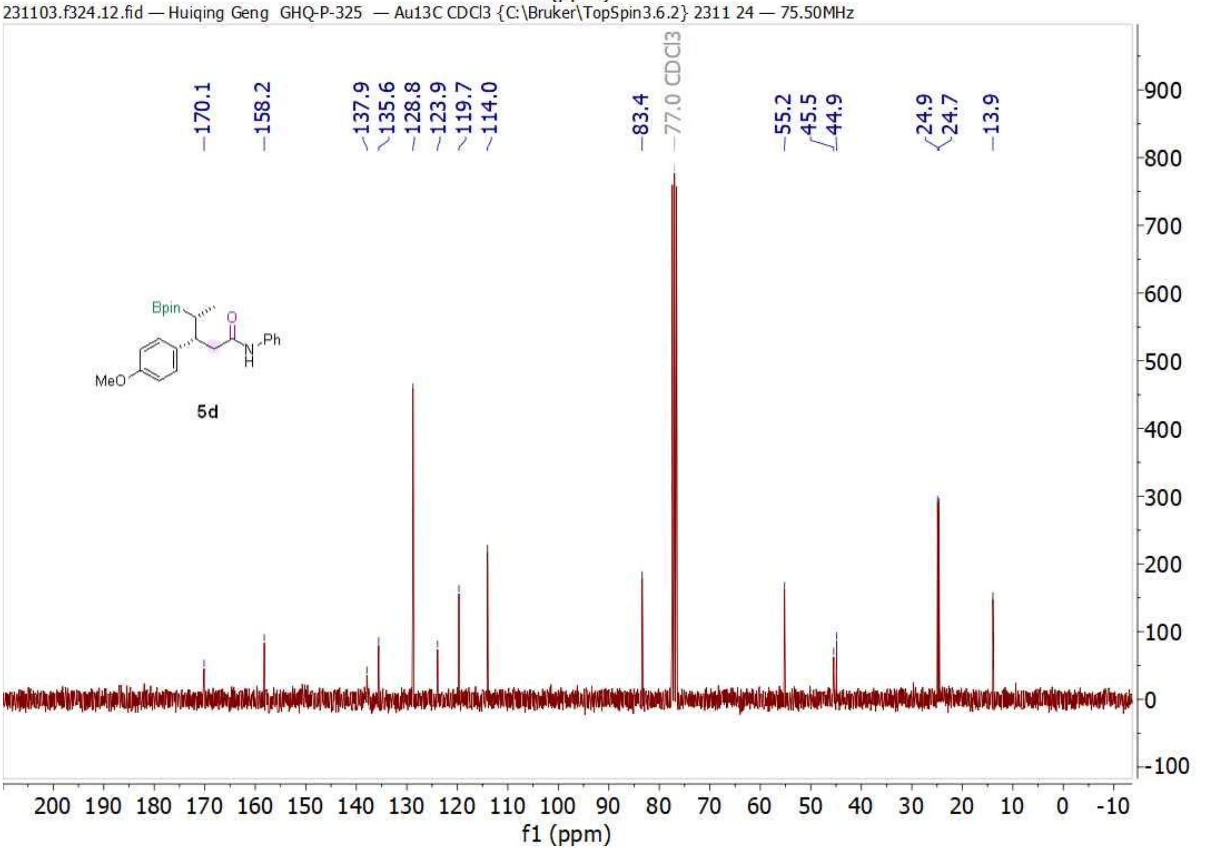
231103.f324.12.fid — Huiqing Geng GHQ-P-325 — Au13C CDCl3 {C:\Bruker\TopSpin3.6.2} 2311 24 — 75.50MHz
170.1
158.2
137.9 135.6 128.8 123.9 119.7 114.0
83.4
77.0 CDCl3
55.2 45.5 44.9
24.9 24.7
13.9
Bpin
O
N
H
Ph
MeO
5d
f1 (ppm)

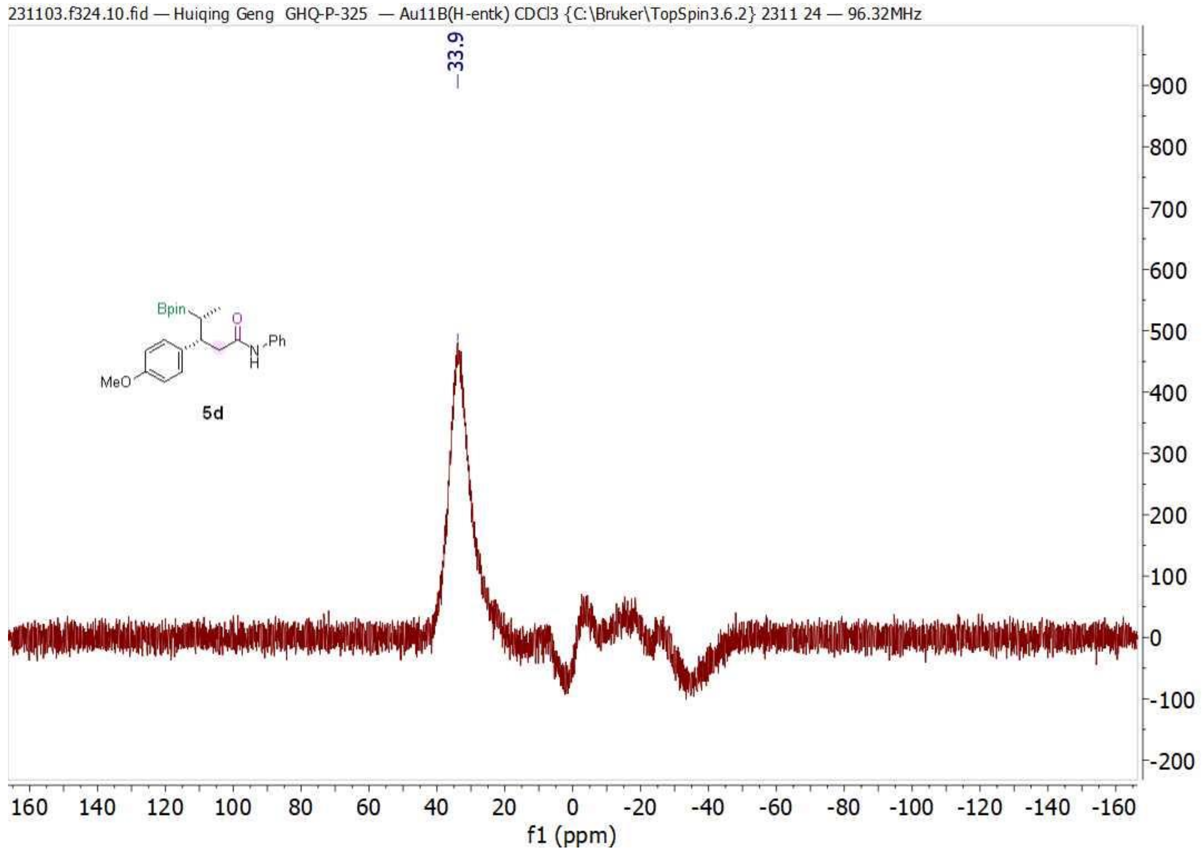

231103.f324.10.fid — Huiqing Geng GHQ-P-325 — Au11B(H-entk) CDCl3 {C:\Bruker\TopSpin3.6.2} 2311 24 — 96.32MHz
33.9
Bpin
O
N
Ph
H
MeO
5d
900
800
700
600
500
400
300
200
100
0
-100
-200
160 140 120 100 80 60 40 20 0 -20 -40 -60 -80 -100 -120 -140 -160
f1 (ppm)

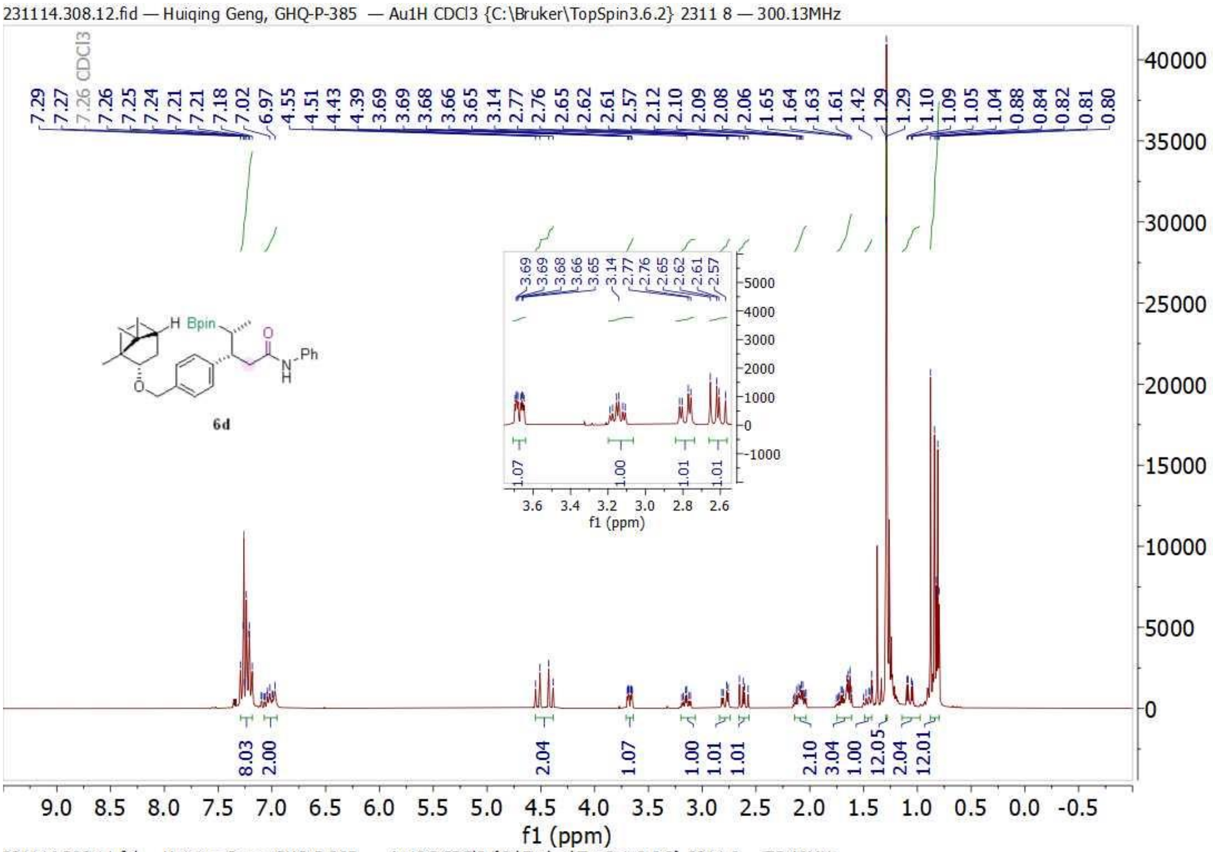

231114.308.12.fid — Huiqing Geng, GHQ-P-385 — Au1H CDCl3 {C:\Bruker\TopSpin3.6.2} 2311 8 — 300.13MHz
6d
f1 (ppm)

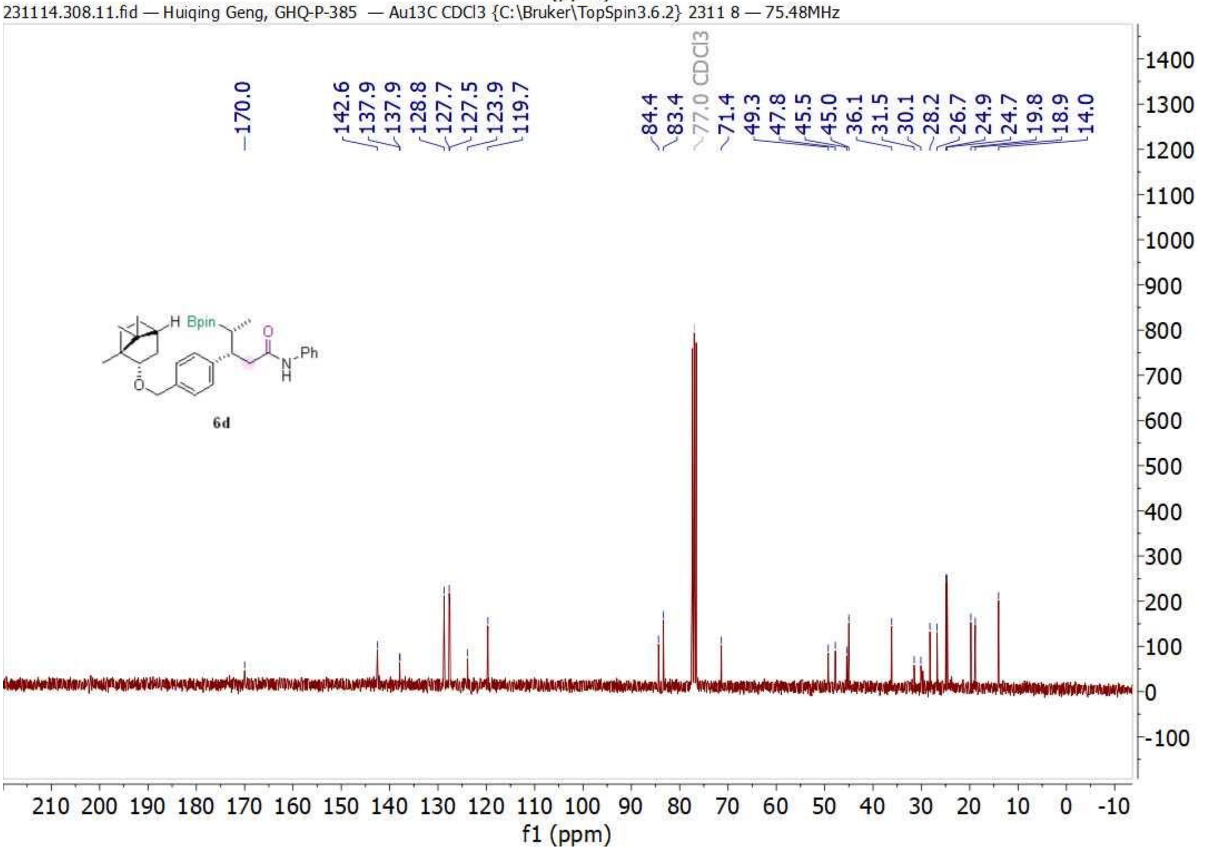

231114.308.11.fid — Huiqing Geng, GHQ-P-385 — Au13C CDCl3 {C:\Bruker\TopSpin3.6.2} 2311 8 — 75.48MHz
6d
f1 (ppm)

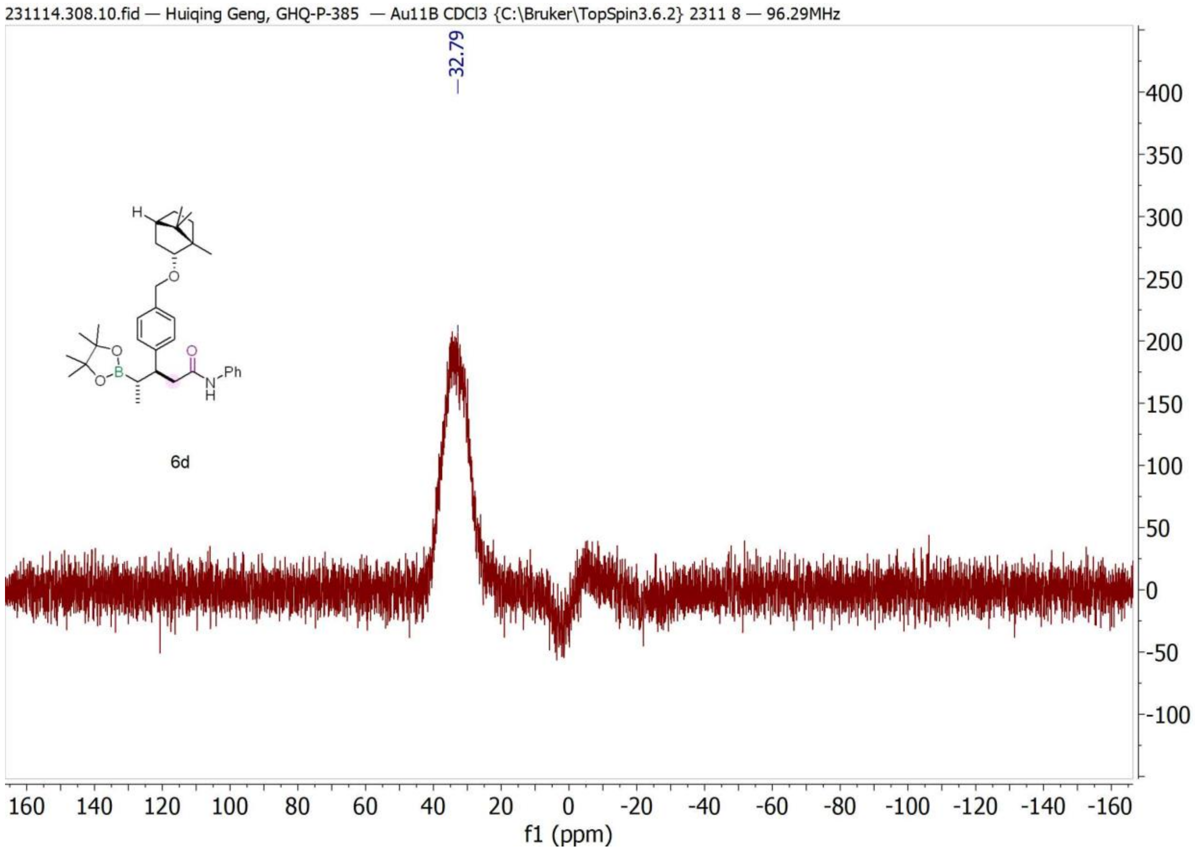
231114.308.10.fid — Huiqing Geng, GHQ-P-385 — Au11B CDCl3 {C:\Bruker\TopSpin3.6.2} 2311 8 — 96.29MHz
—32.79
6d
f1 (ppm)

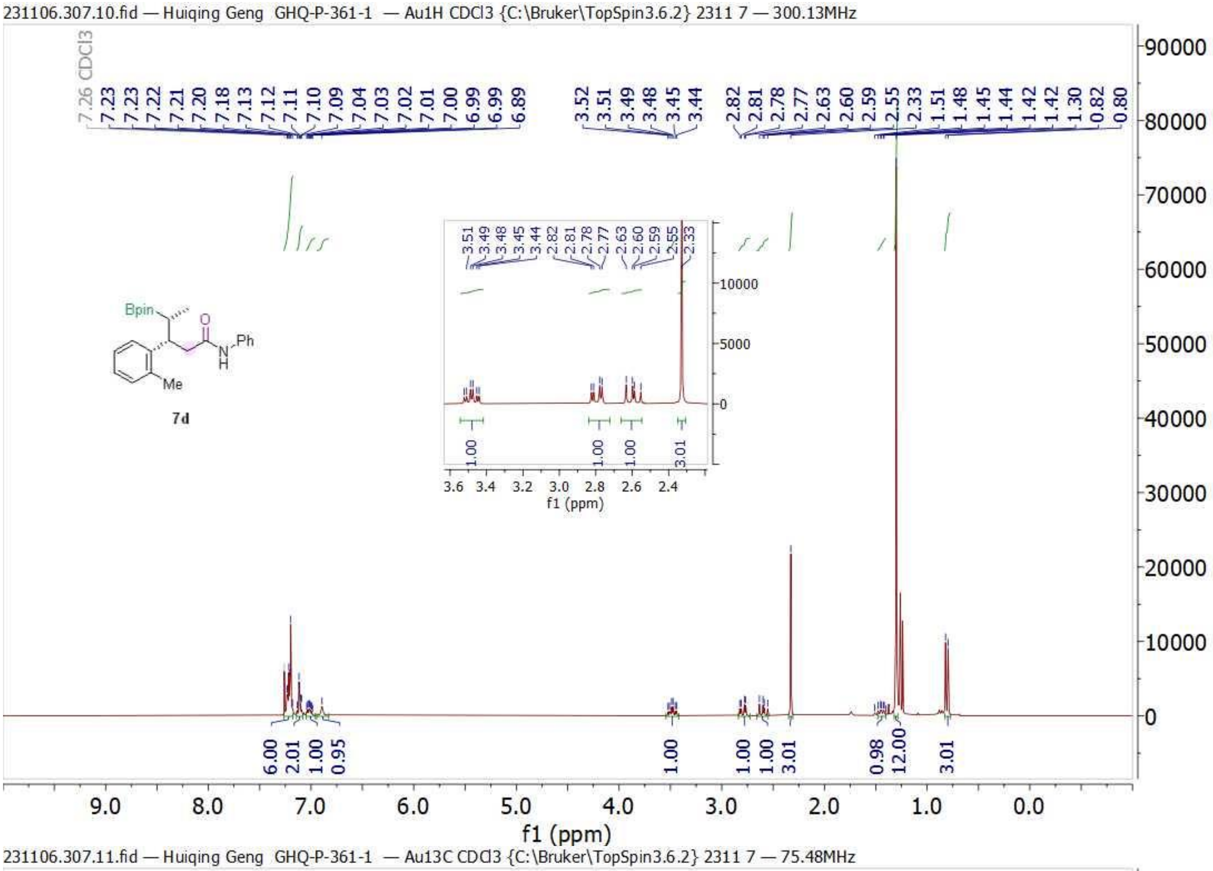
231106.307.10.fid — Huiqing Geng GHQ-P-361-1 — Au1H CDCl3 {C:\Bruker\TopSpin3.6.2} 2311 7 — 300.13MHz
7.26 CDCl3
7.23 7.23 7.22 7.21 7.20 7.18 7.13 7.12 7.11 7.10 7.09 7.04 7.03 7.02 7.01 7.00 6.99 6.99 6.89
3.52 3.51 3.49 3.48 3.45 3.44
2.82 2.81 2.78 2.77 2.63 2.60 2.59 2.55 2.33 1.51 1.48 1.45 1.44 1.42 1.42 1.30 0.82 0.80
Bpin
O
N
H
Ph
Me
7d
6.00 2.01 1.00 0.95
1.00
1.00 1.00 3.01
0.98 12.00
3.01
f1 (ppm)
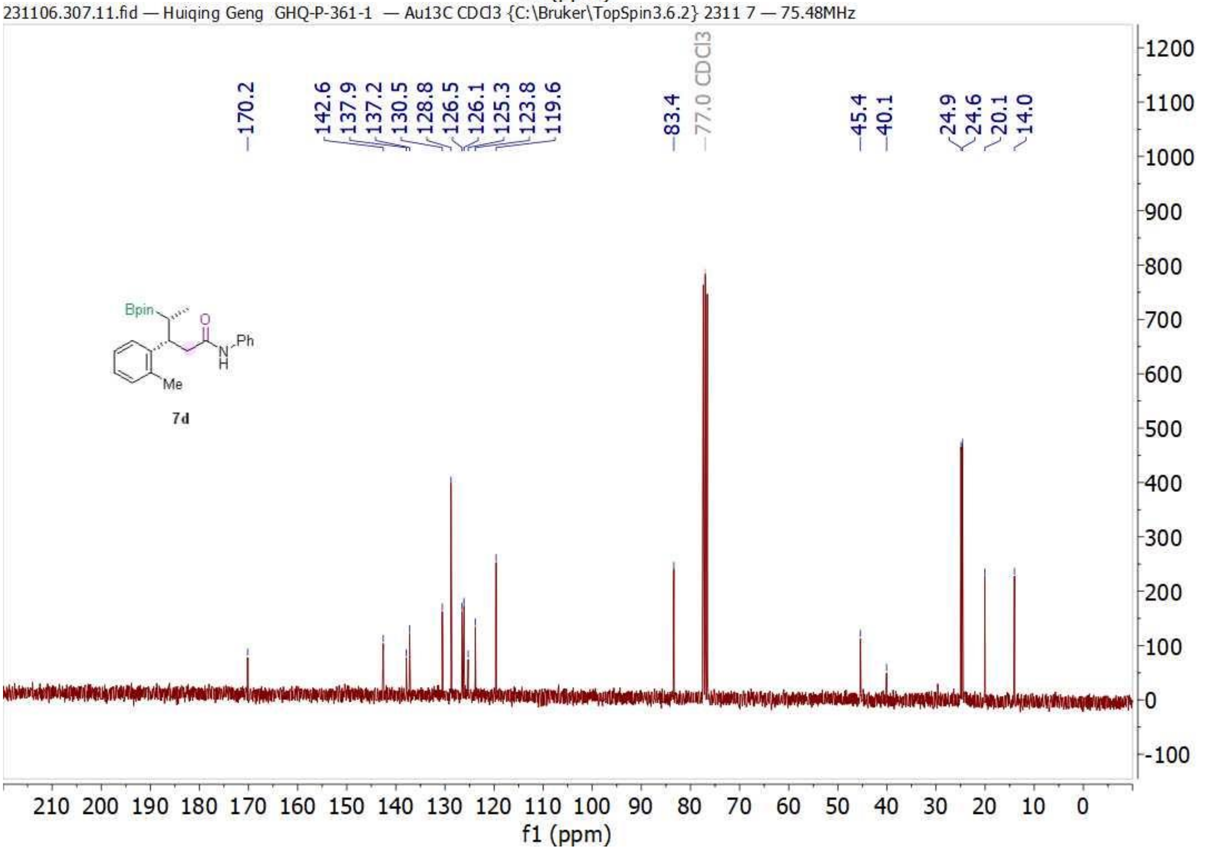
231106.307.11.fid — Huiqing Geng GHQ-P-361-1 — Au13C CDCl3 {C:\Bruker\TopSpin3.6.2} 2311 7 — 75.48MHz
170.2
142.6 137.9 137.2 130.5 128.8 126.5 126.1 125.3 123.8 119.6
83.4
77.0 CDCl3
45.4 40.1
24.9 24.6 20.1 14.0
Bpin
O
N
H
Ph
Me
7d
f1 (ppm)

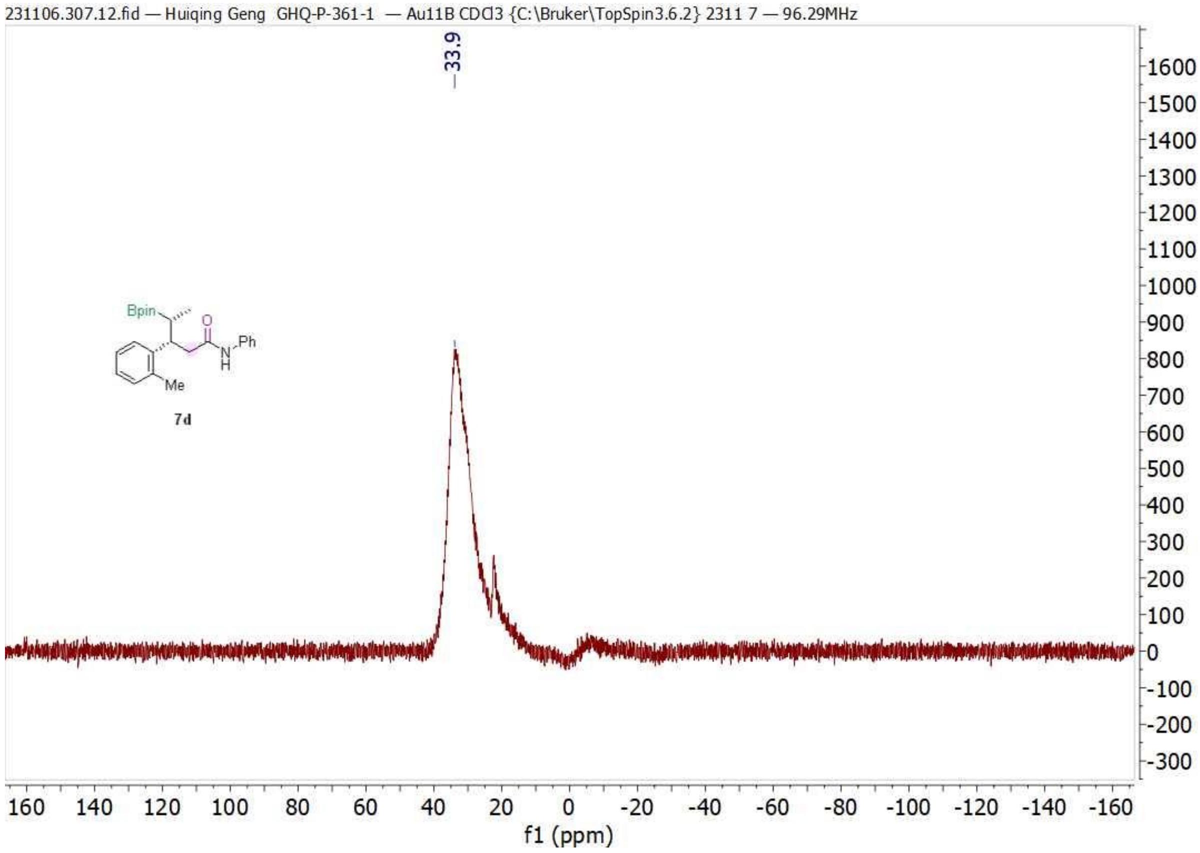

231106.307.12.fid — Huiqing Geng GHQ-P-361-1 — Au11B CDCl3 {C:\Bruker\TopSpin3.6.2} 2311 7 — 96.29MHz
33.9
Bpin
O
N
H
Ph
Me
7d
1600
1500
1400
1300
1200
1100
1000
900
800
700
600
500
400
300
200
100
0
-100
-200
-300
160 140 120 100 80 60 40 20 0 -20 -40 -60 -80 -100 -120 -140 -160
f1 (ppm)

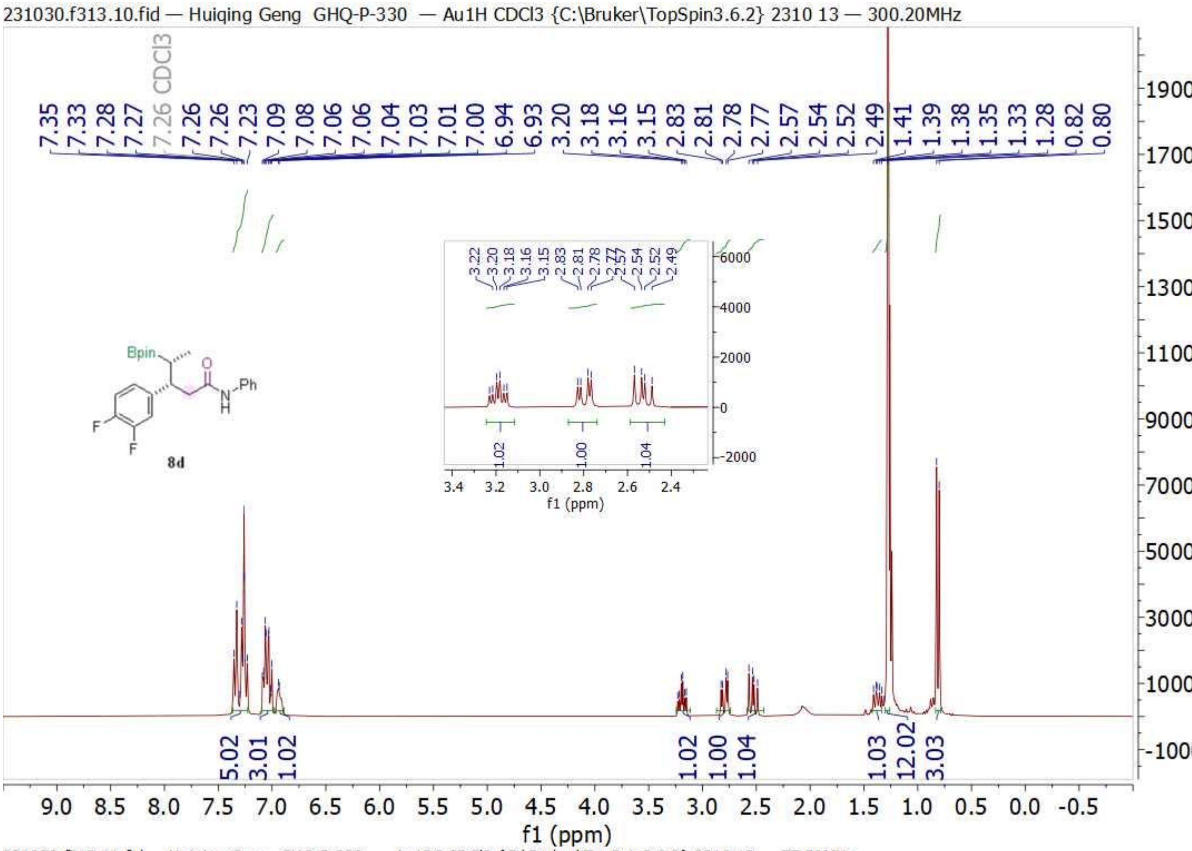
231030.f313.10.fid — Huiqing Geng GHQ-P-330 — Au1H CDCl3 {C:\Bruker\TopSpin3.6.2} 2310 13 — 300.20MHz
7.35 7.33 7.28 7.27 7.26 CDCl3 7.26 7.26 7.23 7.09 7.08 7.06 7.06 7.04 7.03 7.01 7.00 6.94 6.93 3.20 3.18 3.16 3.15 2.83 2.81 2.78 2.77 2.57 2.54 2.52 2.49 1.41 1.39 1.38 1.35 1.33 1.28 0.82 0.80
5.02 3.01 1.02 1.02 1.00 1.04 1.03 12.02 3.03
Bpin
N
H
Ph
O
F
F
8d
f1 (ppm)

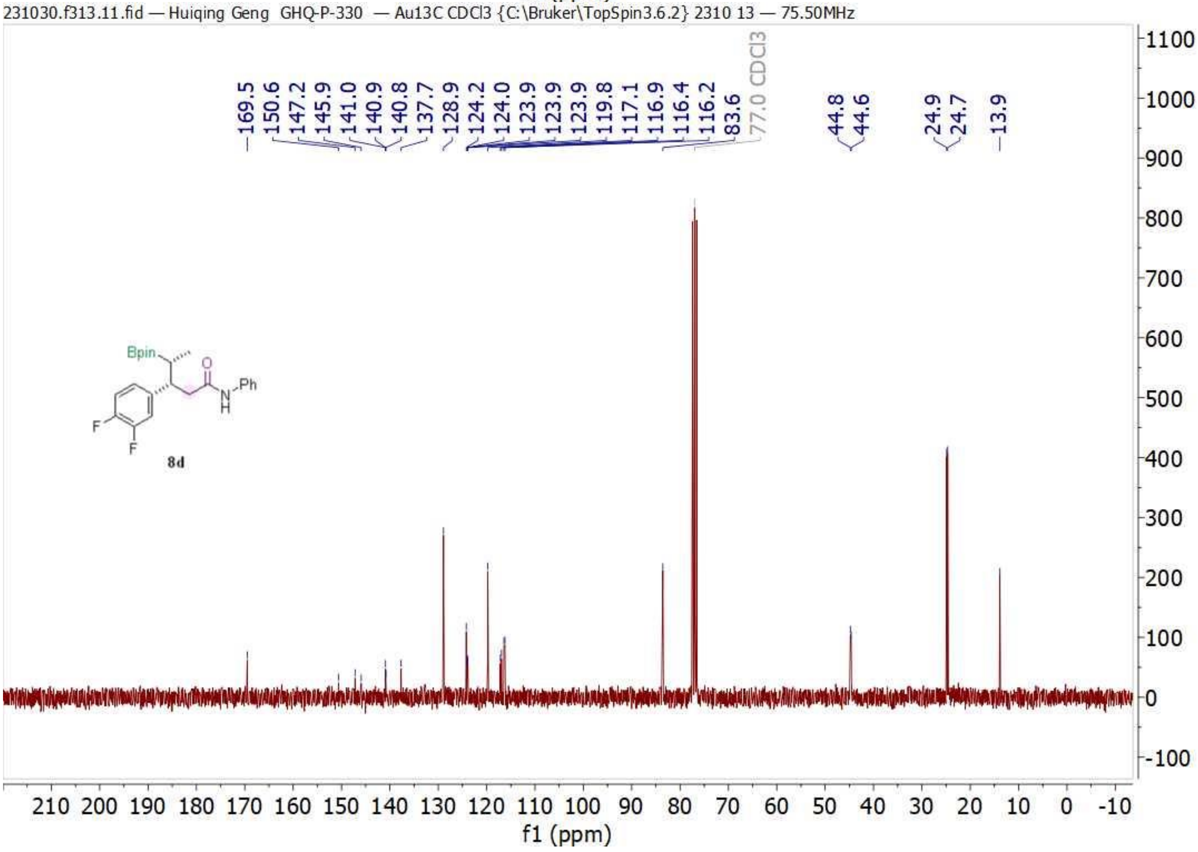
231030.f313.11.fid — Huiqing Geng GHQ-P-330 — Au13C CDCl3 {C:\Bruker\TopSpin3.6.2} 2310 13 — 75.50MHz
169.5 150.6 147.2 145.9 141.0 140.9 140.8 137.7 128.9 124.2 124.0 123.9 123.9 123.9 119.8 117.1 116.9 116.4 116.2 83.6 77.0 CDCl3 44.8 44.6 24.9 24.7 13.9
Bpin
N
H
Ph
O
F
F
8d
f1 (ppm)

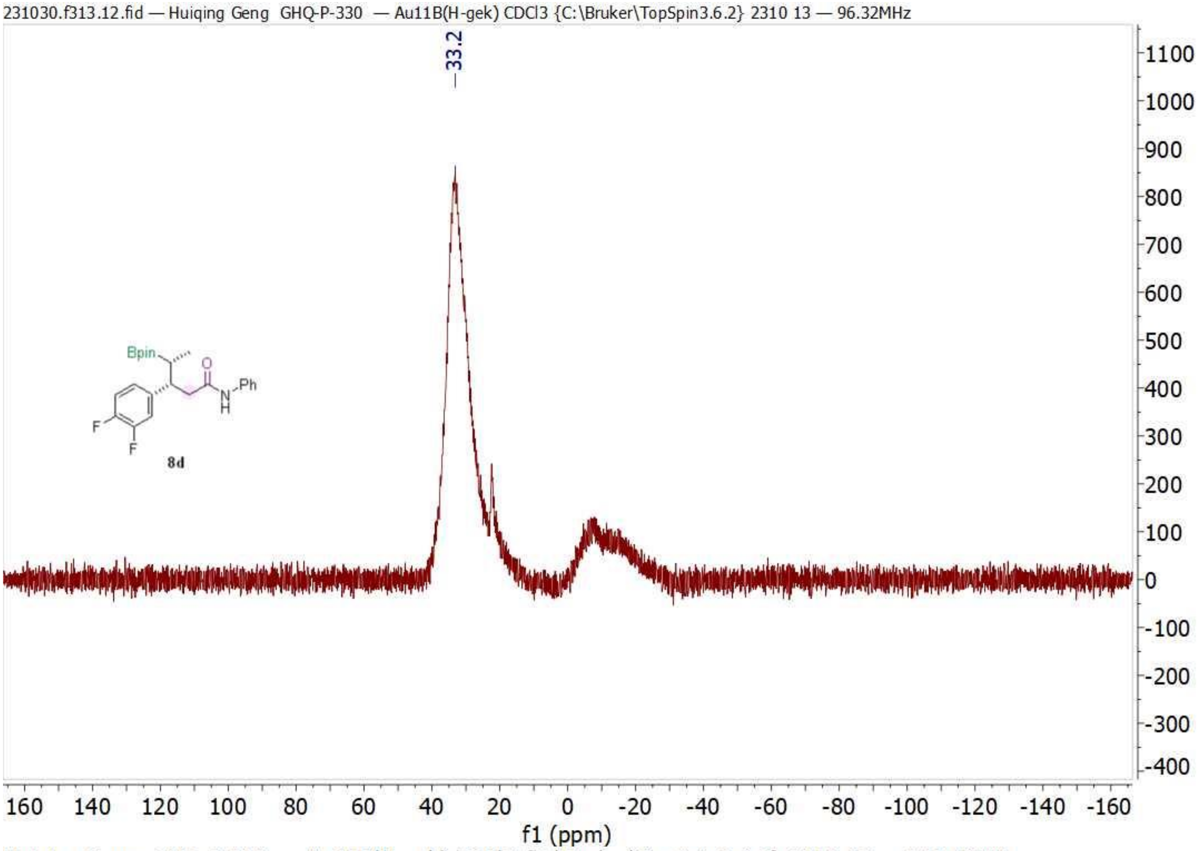
231030.f313.12.fid — Huiqing Geng GHQ-P-330 — Au11B(H-gek) CDCl3 {C:\Bruker\TopSpin3.6.2} 2310 13 — 96.32MHz
33.2
Bpin
O
N
H
Ph
F
F
8d
f1 (ppm)

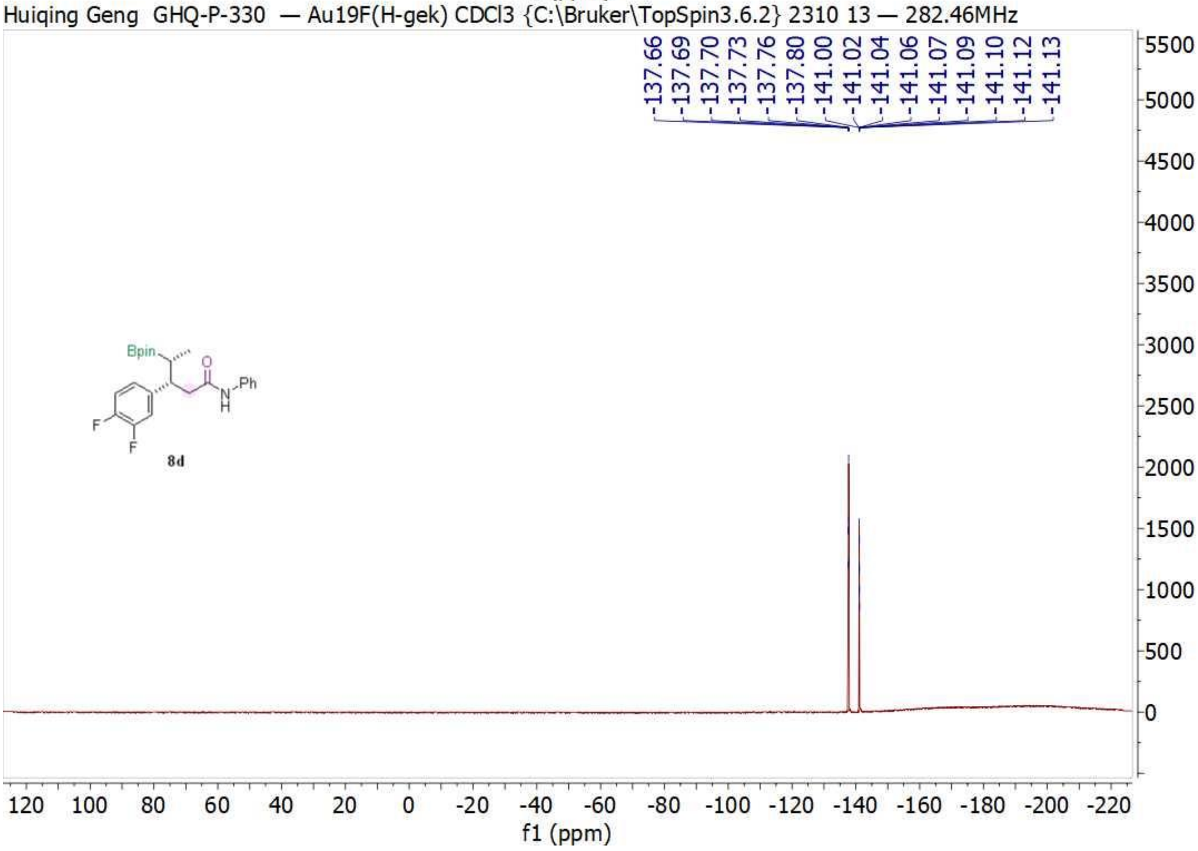
Huiqing Geng GHQ-P-330 — Au19F(H-gek) CDCl3 {C:\Bruker\TopSpin3.6.2} 2310 13 — 282.46MHz
-137.66
-137.69
-137.70
-137.73
-137.76
-137.80
-141.00
-141.02
-141.04
-141.06
-141.07
-141.09
-141.10
-141.12
-141.13
Bpin
O
N
H
Ph
F
F
8d
f1 (ppm)

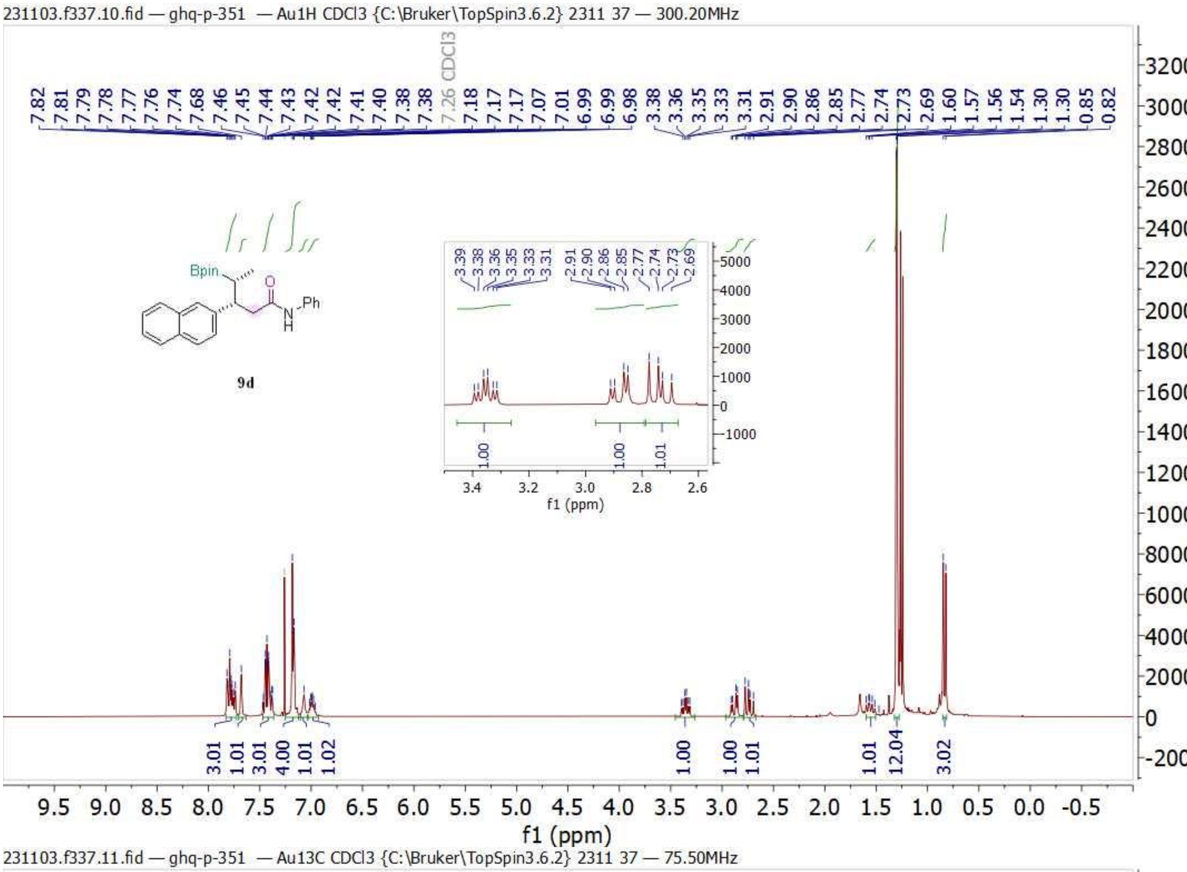
231103.f337.10.fid — ghq-p-351 — Au1H CDCl3 {C:\Bruker\TopSpin3.6.2} 2311 37 — 300.20MHz
7.82 7.81 7.79 7.78 7.77 7.76 7.74 7.68 7.46 7.45 7.44 7.43 7.42 7.42 7.41 7.40 7.38 7.38 7.26 CDCl3 7.18 7.17 7.17 7.07 7.01 6.99 6.99 6.98 3.38 3.36 3.35 3.33 3.31 2.91 2.90 2.86 2.85 2.77 2.74 2.73 2.69 1.60 1.57 1.56 1.54 1.30 0.85 0.82
Bpin
O
N
H
Ph
9d
3.39 3.38 3.36 3.35 3.33 3.31 2.91 2.90 2.86 2.85 2.77 2.74 2.73 2.69
1.00 1.00 1.01
f1 (ppm)
3.01 1.01 3.01 4.00 1.01 1.02 1.00 1.00 1.01 1.01 12.04 3.02
f1 (ppm)

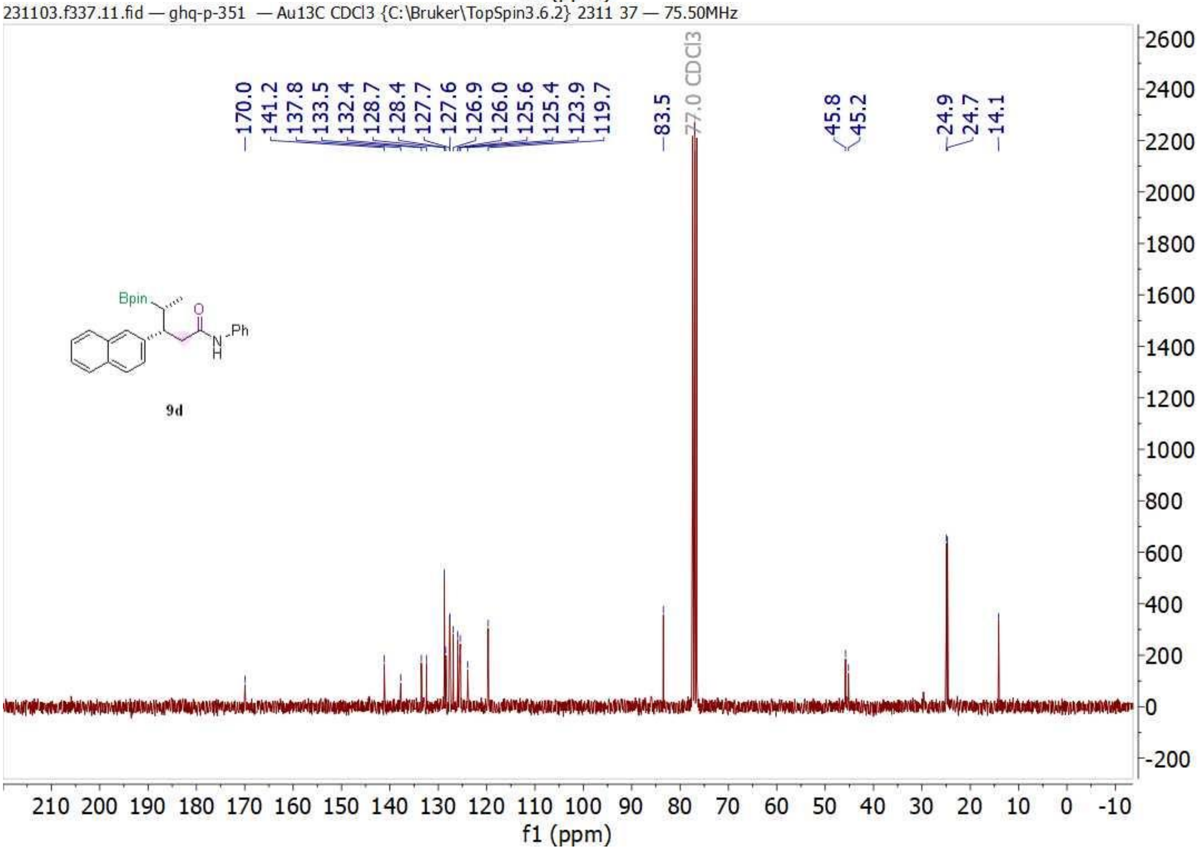
231103.f337.11.fid — ghq-p-351 — Au13C CDCl3 {C:\Bruker\TopSpin3.6.2} 2311 37 — 75.50MHz
170.0 141.2 137.8 133.5 132.4 128.7 128.4 127.7 127.6 126.9 126.0 125.6 125.4 123.9 119.7 83.5 77.0 CDCl3 45.8 45.2 24.9 24.7 14.1
Bpin
O
N
H
Ph
9d
f1 (ppm)

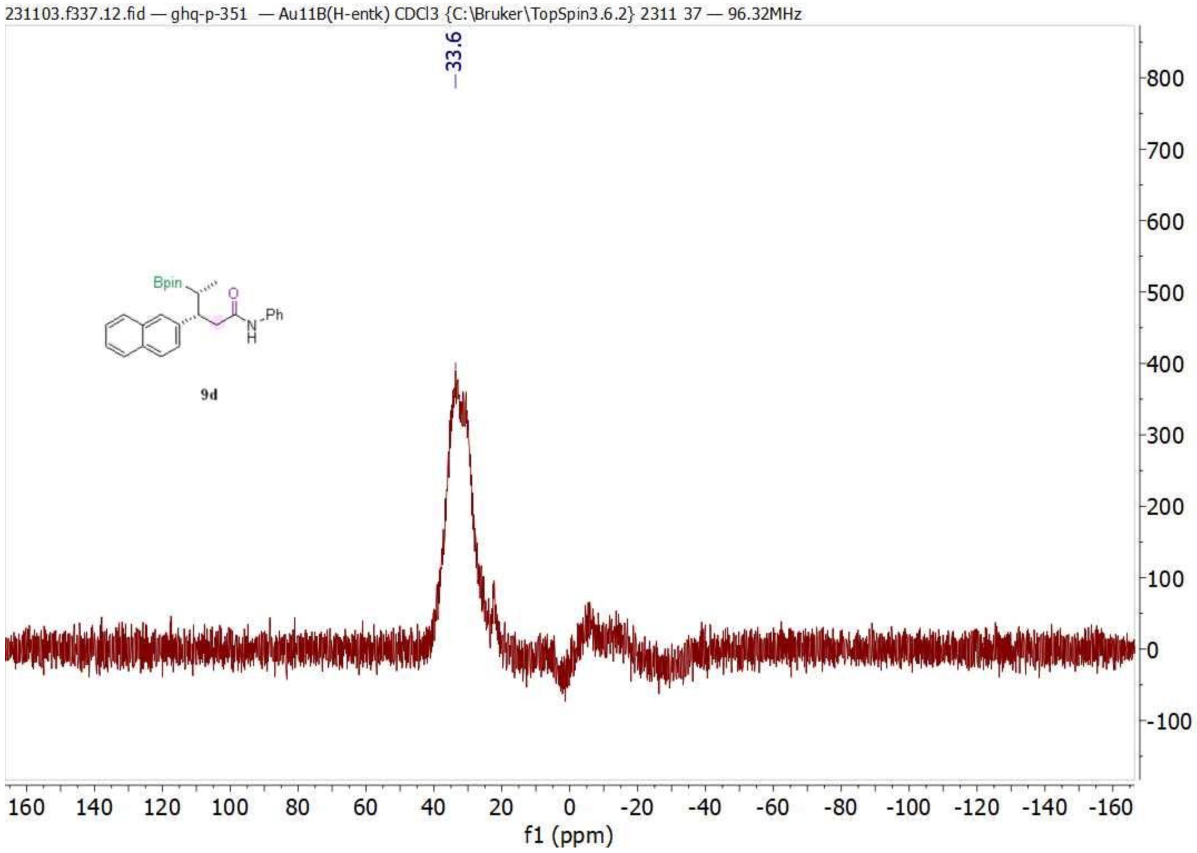

231103.f337.12.fid — ghq-p-351 — Au11B(H-entk) CDCl3 {C:\Bruker\TopSpin3.6.2} 2311 37 — 96.32MHz
33.6
Bpin
O
N
H
Ph
9d
800
700
600
500
400
300
200
100
0
-100
160 140 120 100 80 60 40 20 0 -20 -40 -60 -80 -100 -120 -140 -160
f1 (ppm)

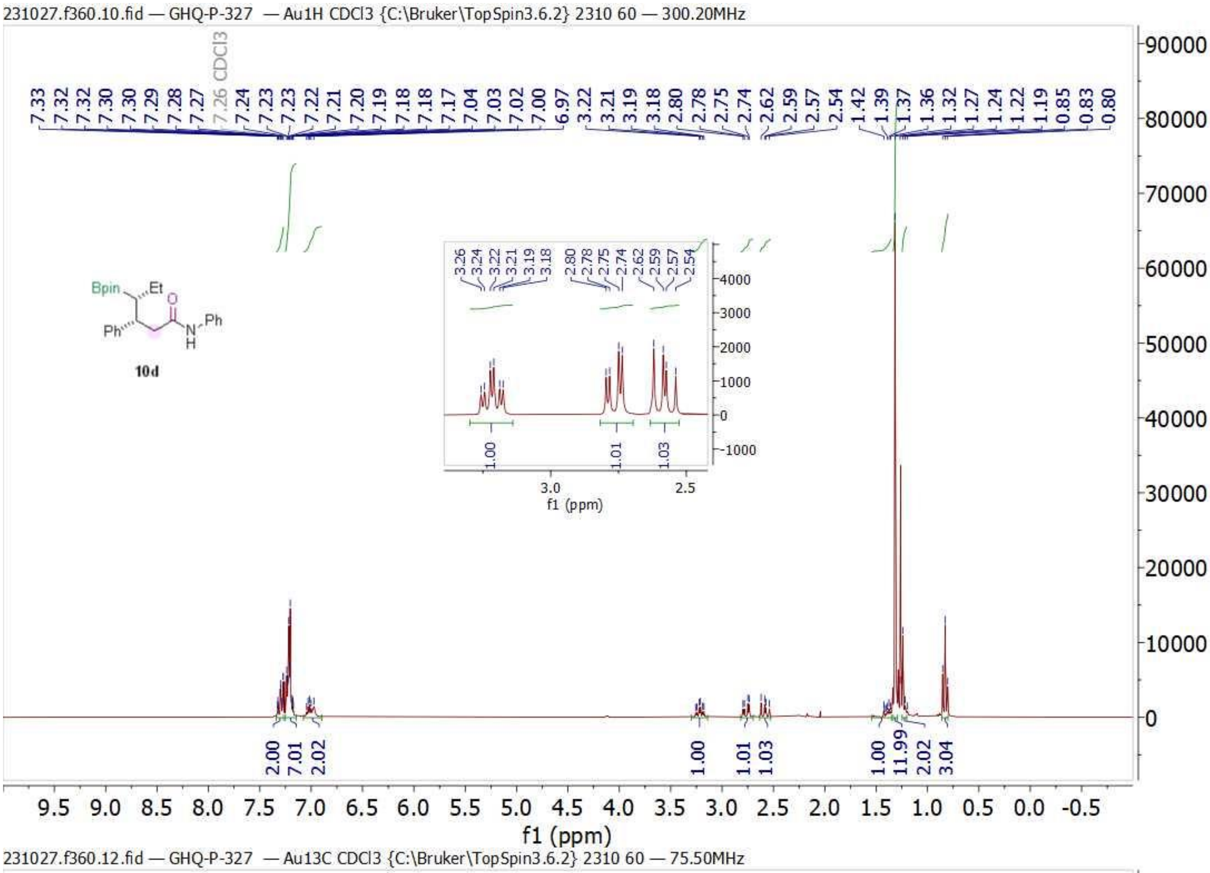
231027.f360.10.fid — GHQ-P-327 — Au1H CDCl3 {C:\Bruker\TopSpin3.6.2} 2310 60 — 300.20MHz
10d
f1 (ppm)

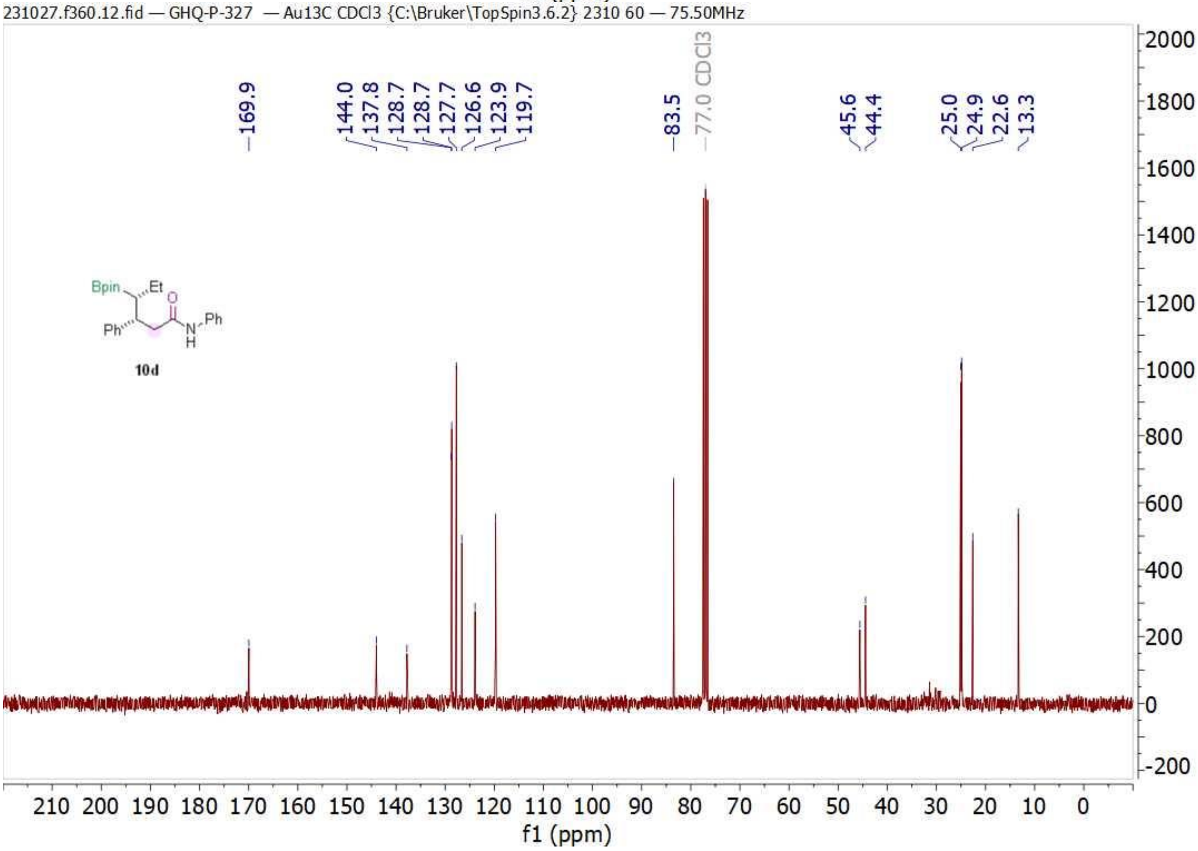
231027.f360.12.fid — GHQ-P-327 — Au13C CDCl3 {C:\Bruker\TopSpin3.6.2} 2310 60 — 75.50MHz
169.9
144.0
137.8
128.7
128.7
127.7
126.6
123.9
119.7
83.5
77.0 CDCl3
45.6
44.4
25.0
24.9
22.6
13.3
10d
f1 (ppm)

231027.f360.11.fid — GHQ-P-327 — Au11B(H-entk) CDCl3 {C:\Bruker\TopSpin3.6.2} 2310 60 — 96.32MHz

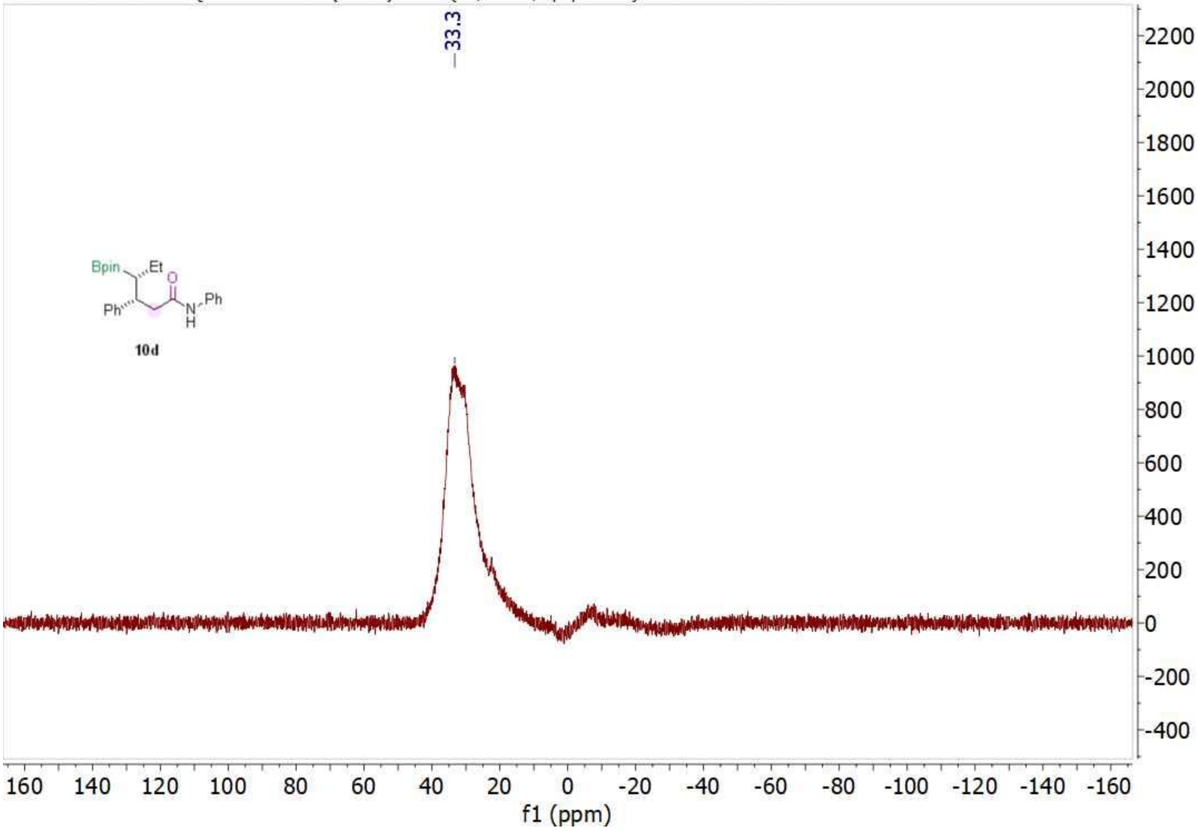

231027.307.10.fid — Huiqing Geng GHQ-P-328 — Au1H CDCl3 {C:\Bruker\TopSpin3.6.2} 2310 7 — 300.13MHz

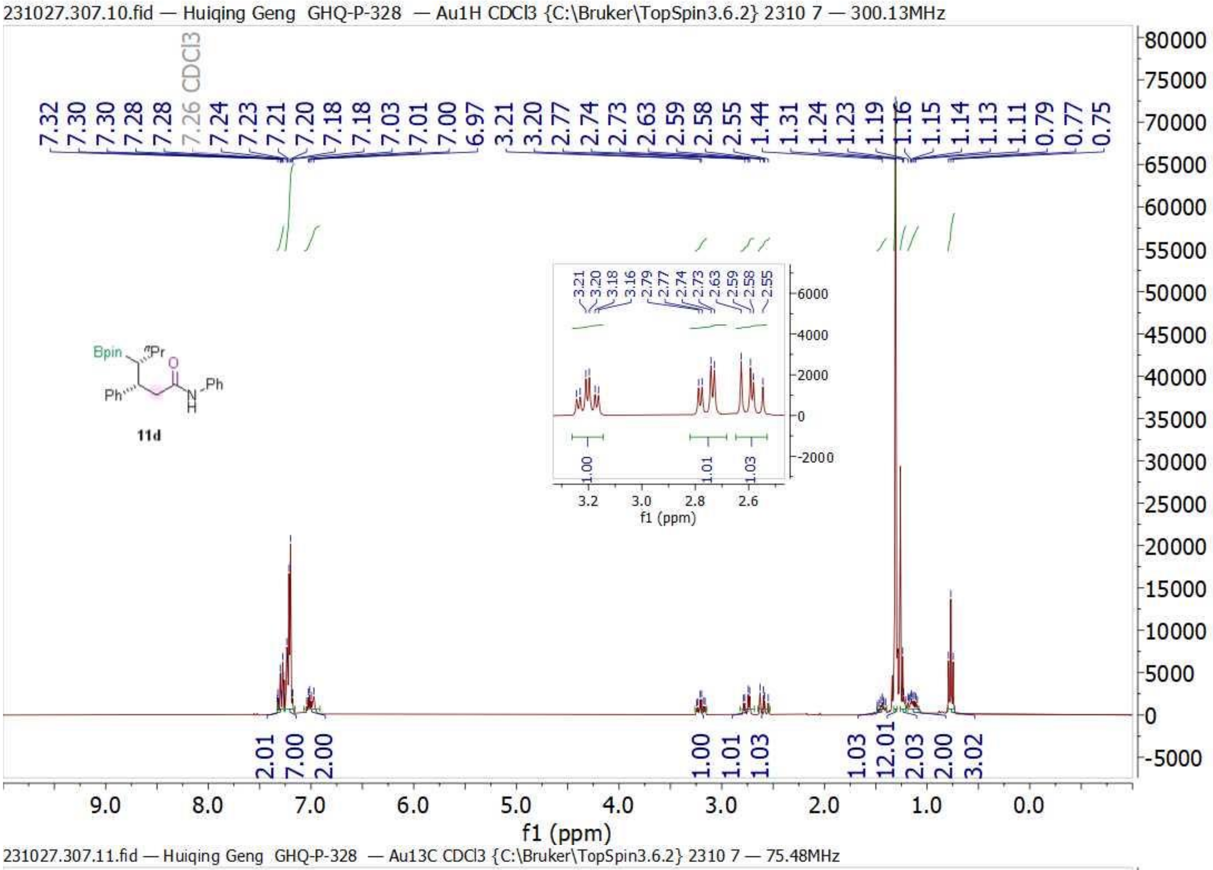

231027.307.11.fid — Huiqing Geng GHQ-P-328 — Au13C CDCl3 {C:\Bruker\TopSpin3.6.2} 2310 7 — 75.48MHz

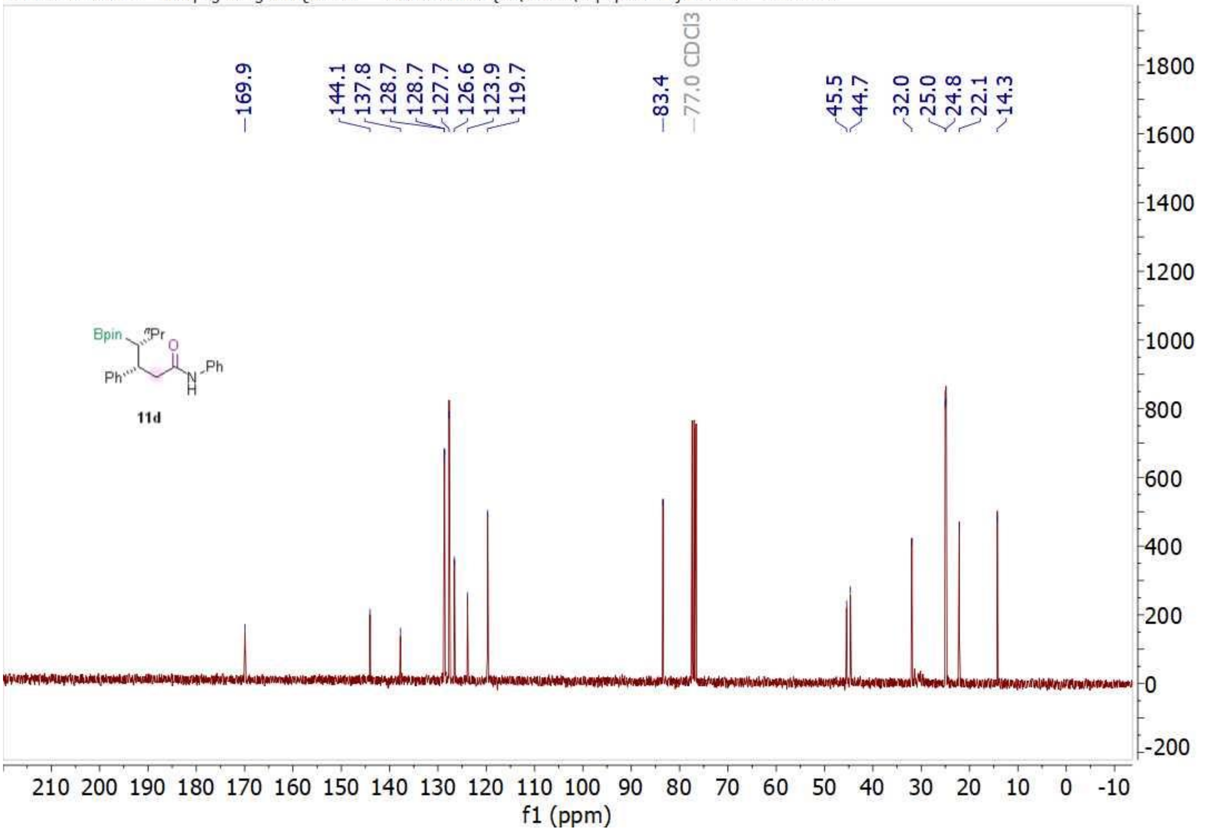

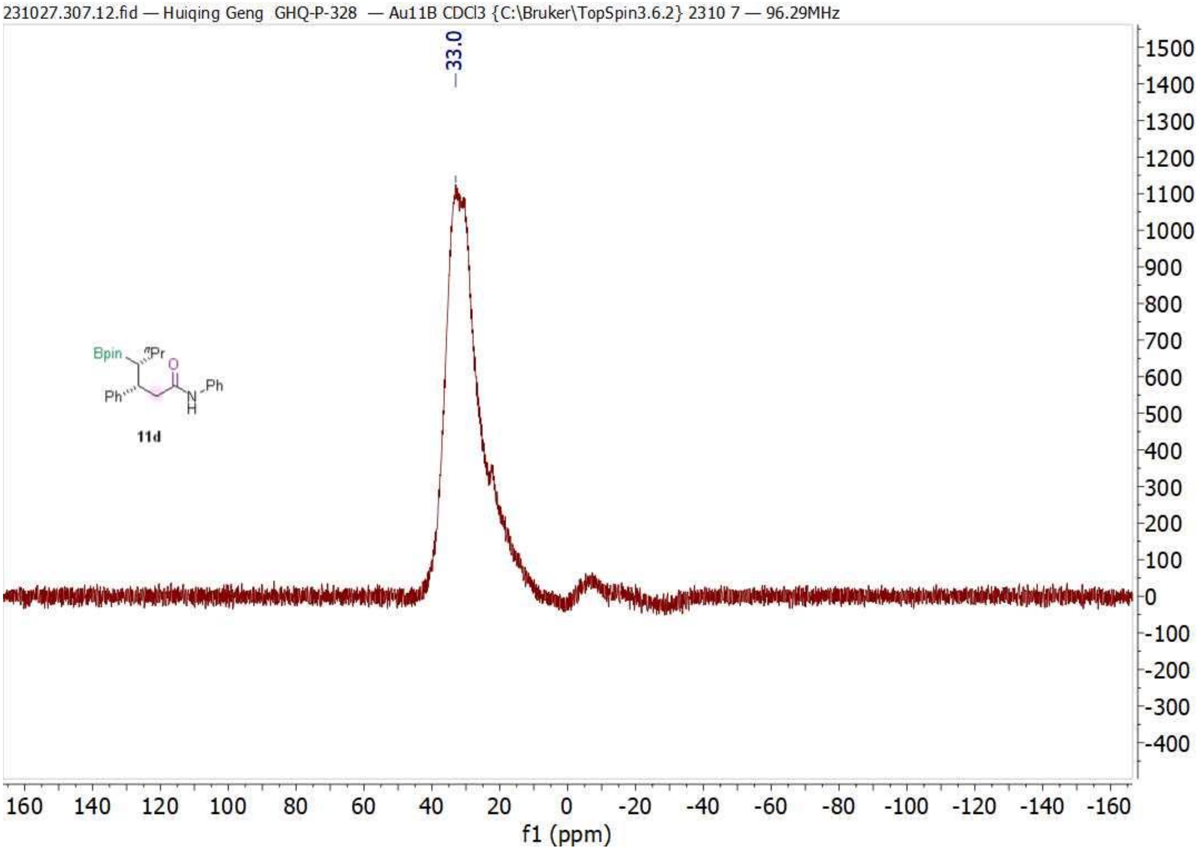
231027.307.12.fid — Huiqing Geng GHQ-P-328 — Au11B CDCl3 {C:\Bruker\TopSpin3.6.2} 2310 7 — 96.29MHz
—33.0
Bpin
iPr
O
Ph
N
H
Ph
11d
1500
1400
1300
1200
1100
1000
900
800
700
600
500
400
300
200
100
0
-100
-200
-300
-400
160 140 120 100 80 60 40 20 0 -20 -40 -60 -80 -100 -120 -140 -160
f1 (ppm)

231121.307.10.fid — Huiqing Geng GHQ-P-387 — Au1H CDCl3 {C:\Bruker\TopSpin3.6.2} 2311 7 — 300.13MHz

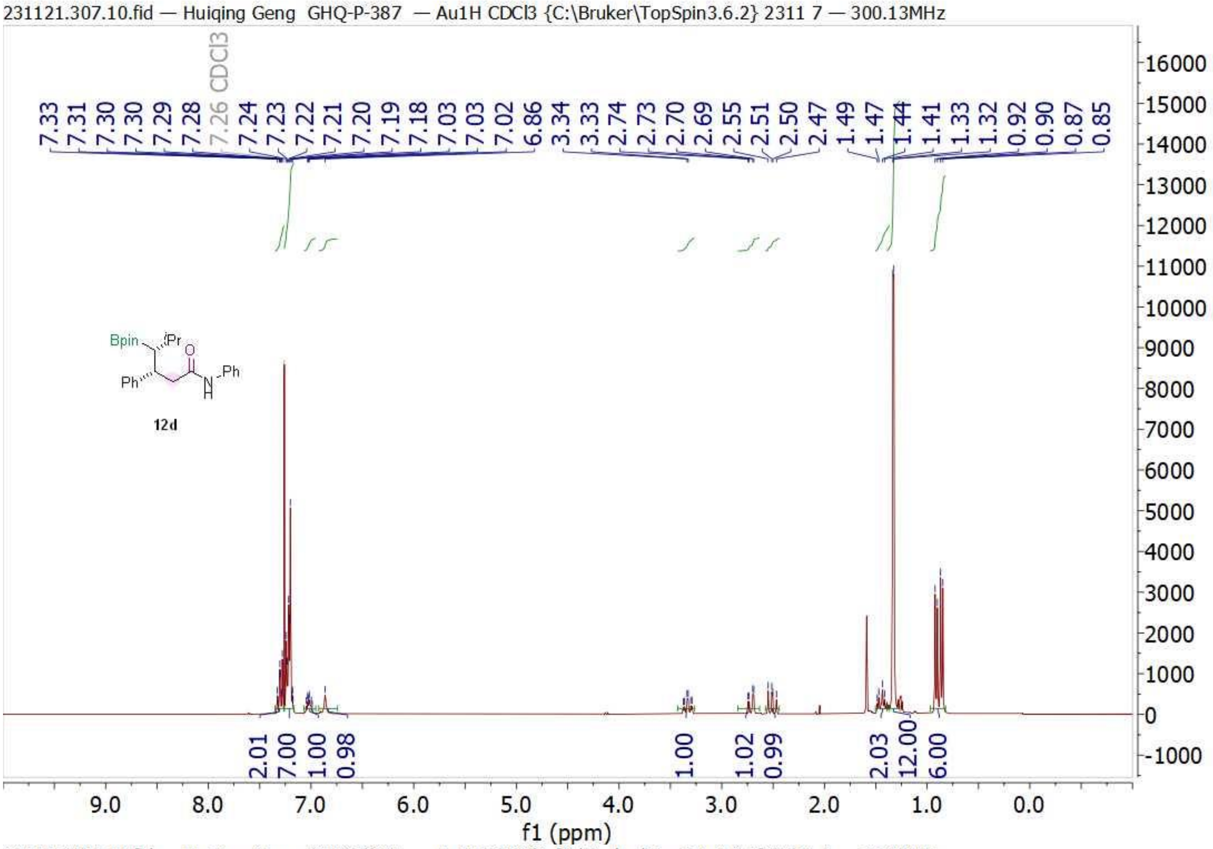

231121.307.11.fid — Huiqing Geng GHQ-P-387 — Au13C CDCl3 {C:\Bruker\TopSpin3.6.2} 2311 7 — 75.48MHz

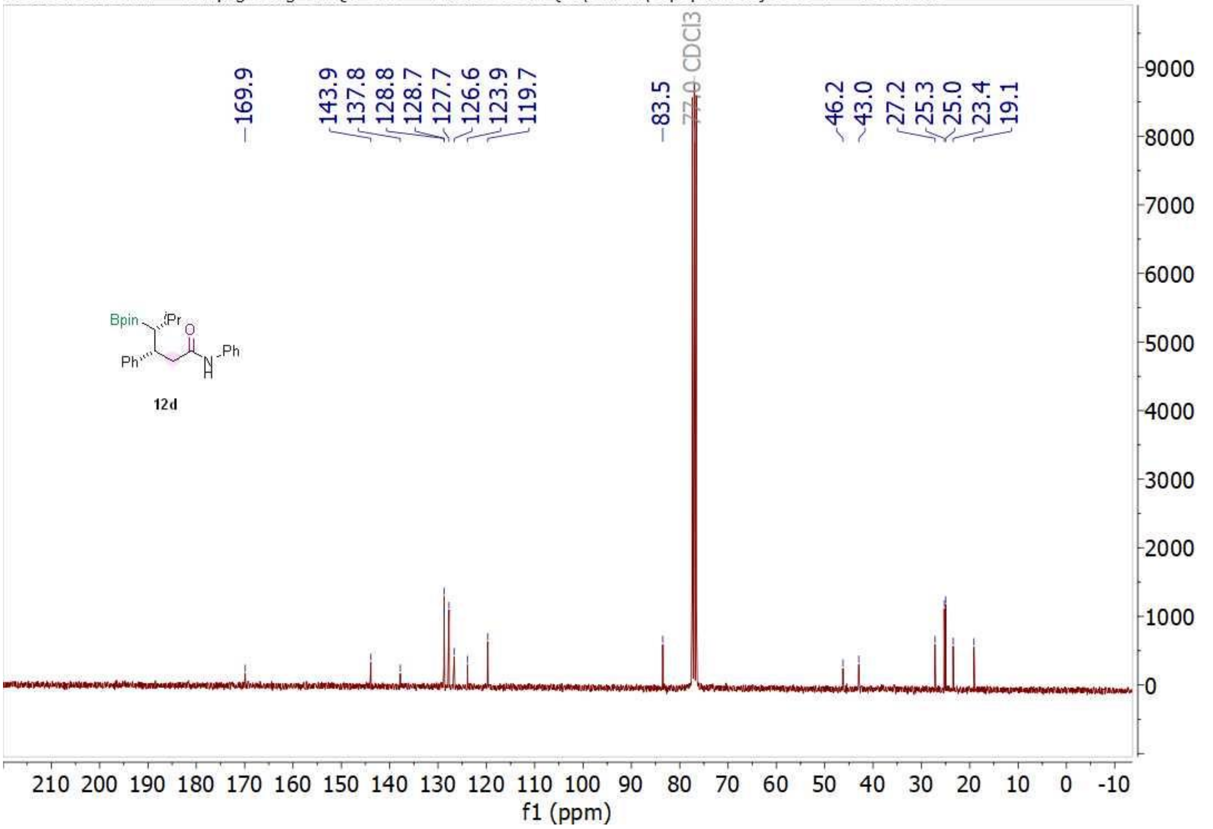

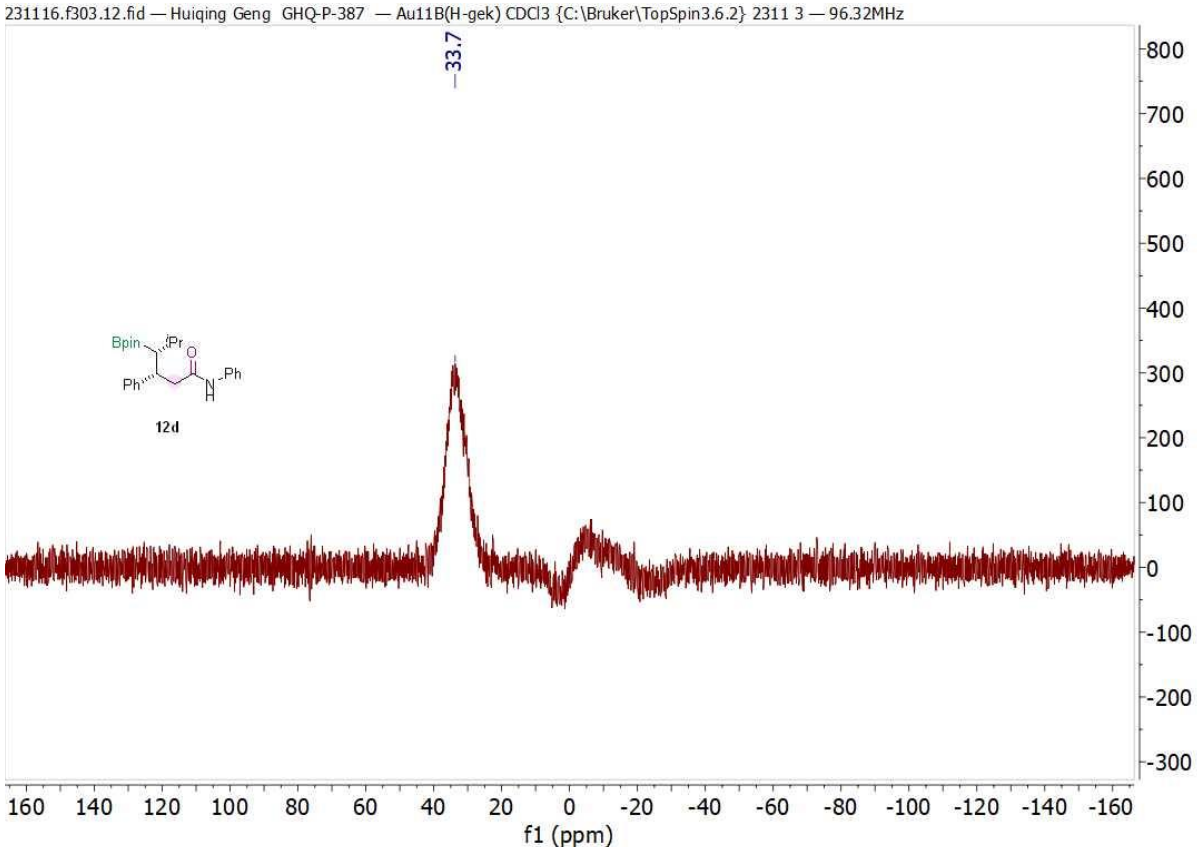
231116.f303.12.fid — Huiqing Geng GHQ-P-387 — Au11B(H-gek) CDCl3 {C:\Bruker\TopSpin3.6.2} 2311 3 — 96.32MHz
—33.7
Bpin
iPr
O
Ph
N
H
Ph
12d
800
700
600
500
400
300
200
100
0
-100
-200
-300
160 140 120 100 80 60 40 20 0 -20 -40 -60 -80 -100 -120 -140 -160
f1 (ppm)

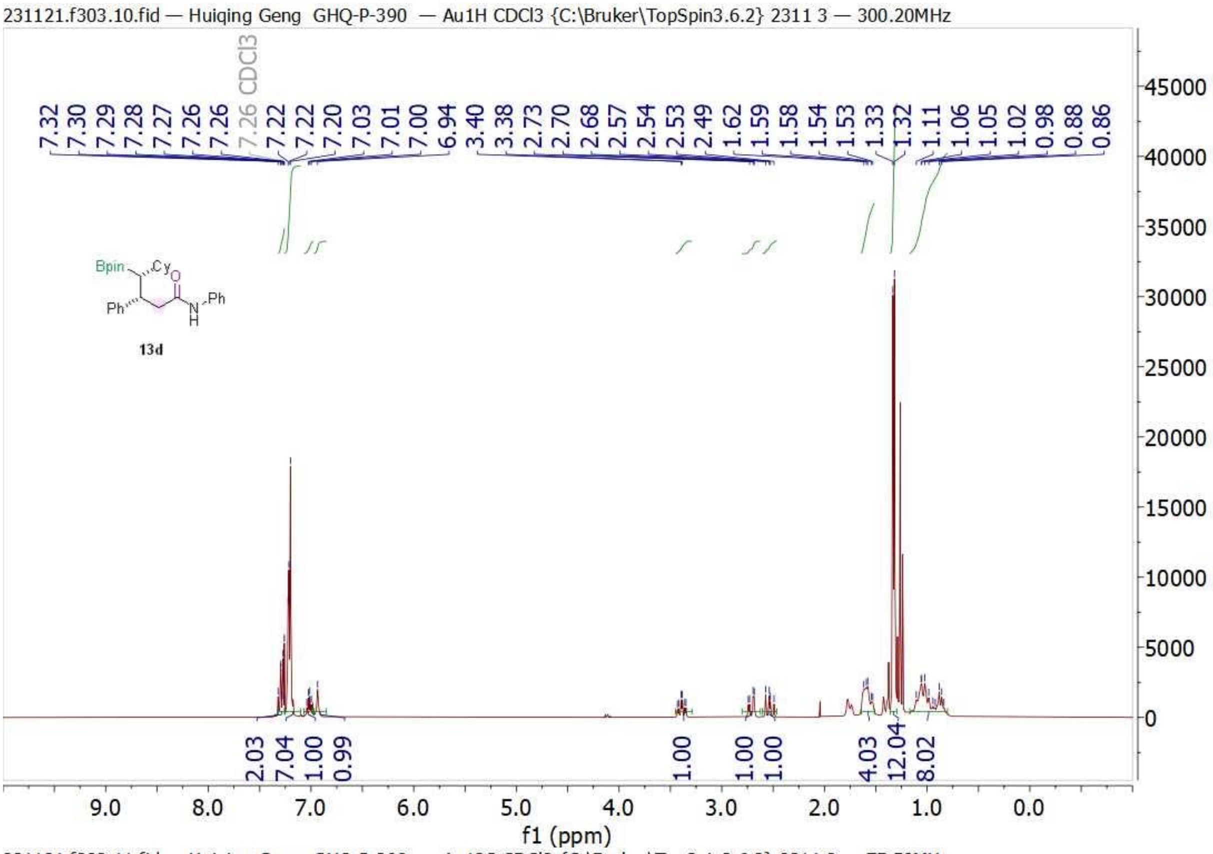
231121.f303.10.fid — Huiqing Geng GHQ-P-390 — Au1H CDCl3 {C:\Bruker\TopSpin3.6.2} 2311 3 — 300.20MHz
7.32 7.30 7.29 7.28 7.27 7.26 7.26 7.26 CDCl3 7.22 7.22 7.20 7.03 7.01 7.00 6.94 3.40 3.38 2.73 2.70 2.68 2.57 2.54 2.53 2.49 1.62 1.59 1.58 1.54 1.53 1.33 1.32 1.11 1.06 1.05 1.02 1.02 0.98 0.88 0.86
Bpin
Cy
O
Ph
N
H
Ph
13d
2.03 7.04 1.00 0.99 1.00 1.00 1.00 4.03 12.04 8.02
f1 (ppm)

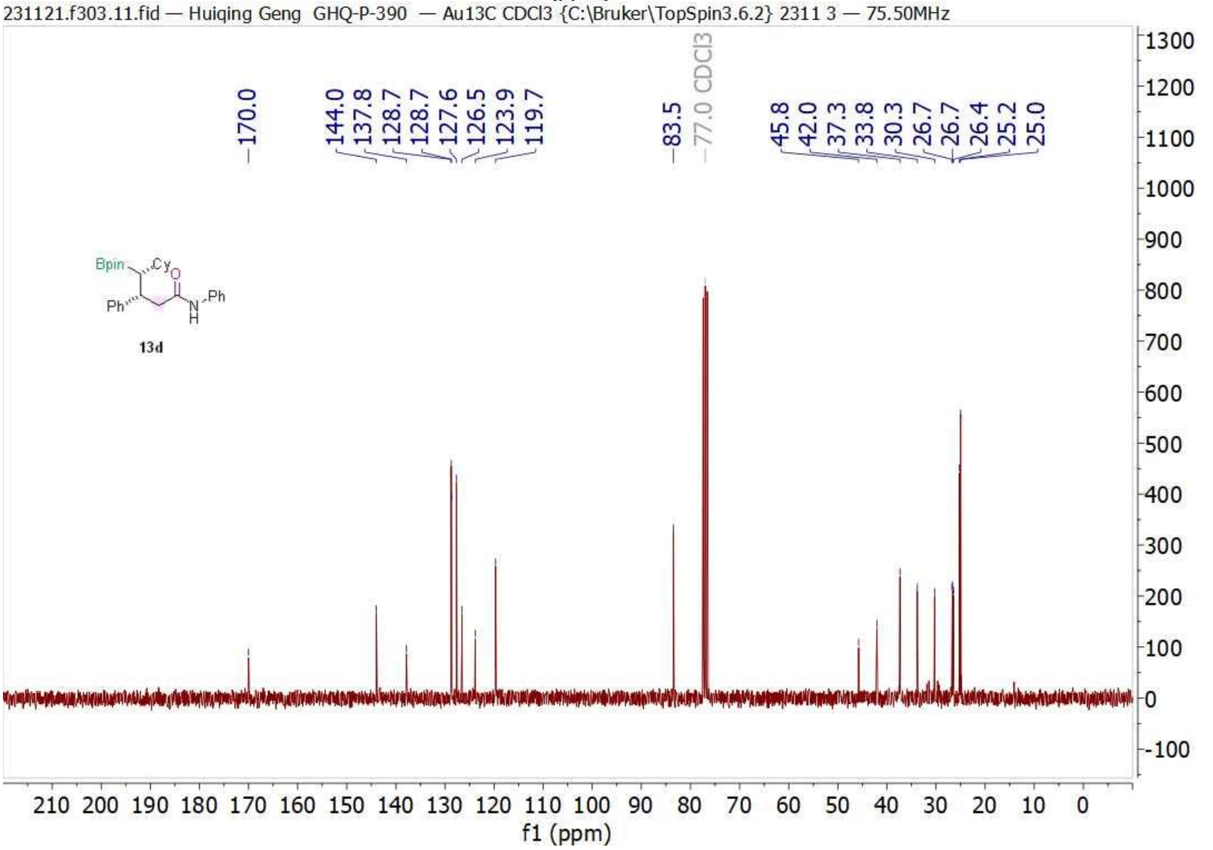
231121.f303.11.fid — Huiqing Geng GHQ-P-390 — Au13C CDCl3 {C:\Bruker\TopSpin3.6.2} 2311 3 — 75.50MHz
170.0
144.0 137.8 128.7 128.7 127.6 126.5 123.9 119.7
83.5
77.0 CDCl3
45.8 42.0 37.3 33.8 30.3 26.7 26.7 26.4 25.2 25.0
Bpin
Cy
O
Ph
N
H
Ph
13d
f1 (ppm)

Huiqing Geng GHQ-P-390 — Au11B(H-gek) CDCl3 {C:\Bruker\TopSpin3.6.2} 2311 3 — 96.32MHz

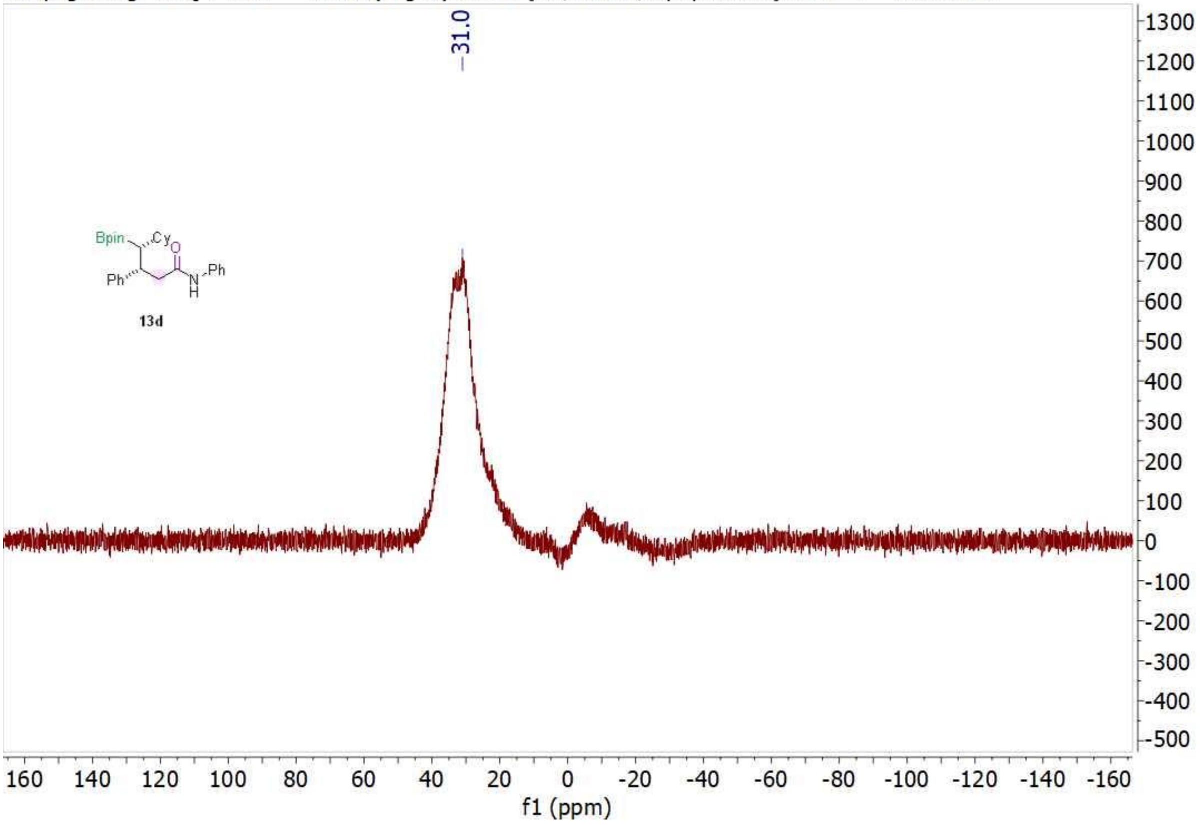

231110.f34.10.fid — ghq-pa-2 — Au1H CDCl3 {C:\Bruker\TopSpin3.6.2} 2311 4 — 300.20MHz

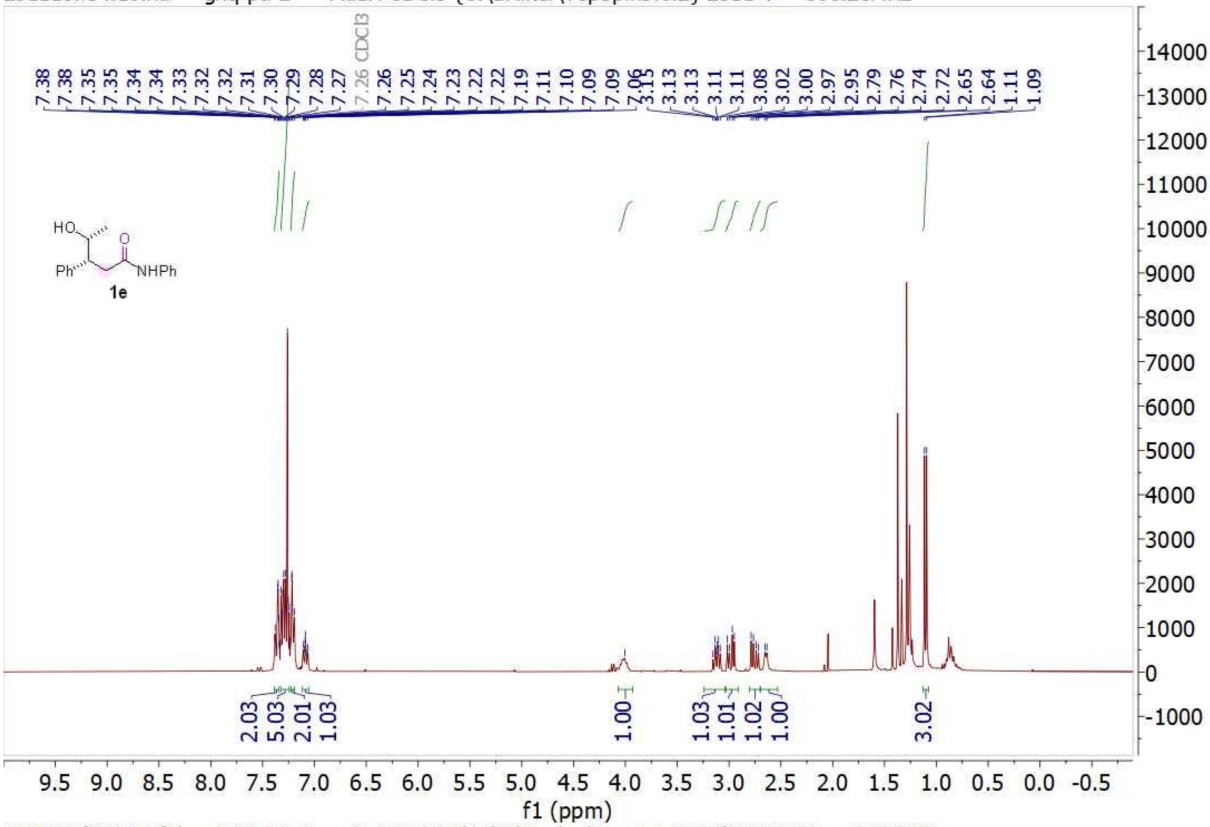

231030.f323.11.fid — GHQ-PA-2 — Au13C CDCl3 {C:\Bruker\TopSpin3.6.2} 2310 23 — 75.50MHz

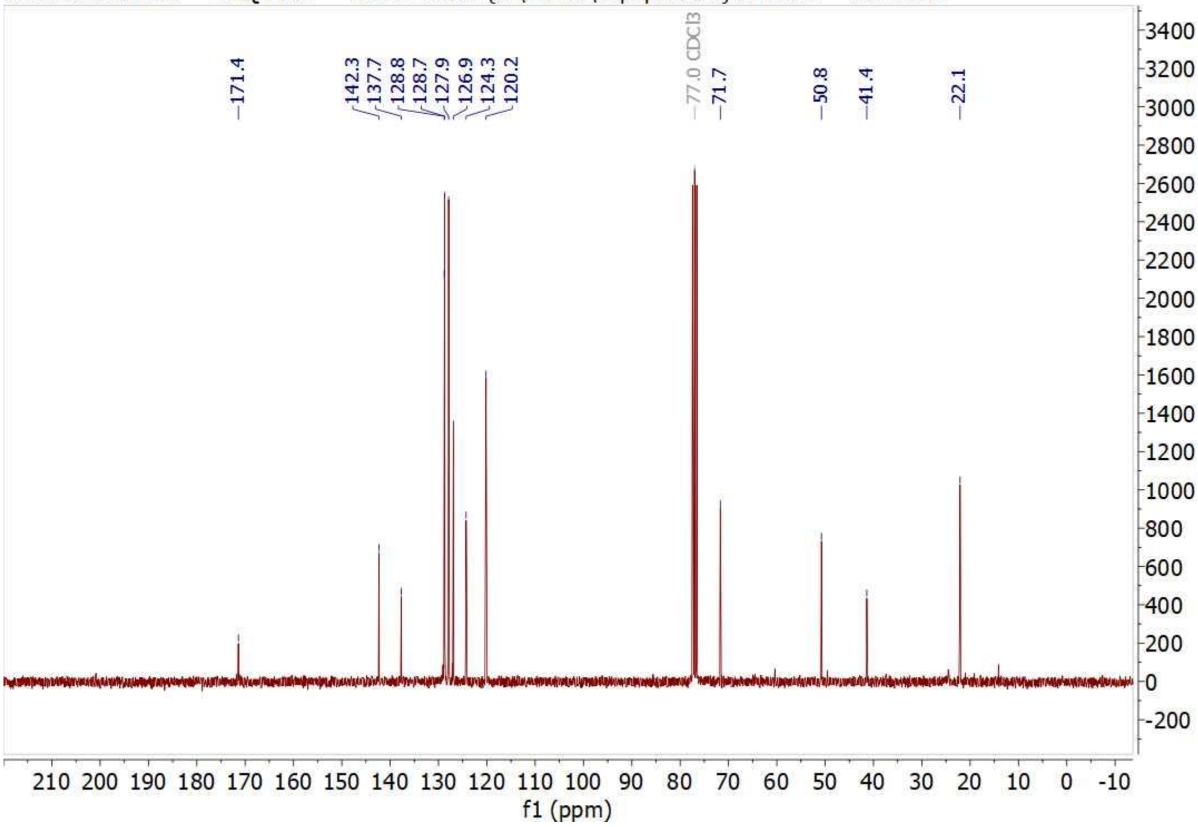

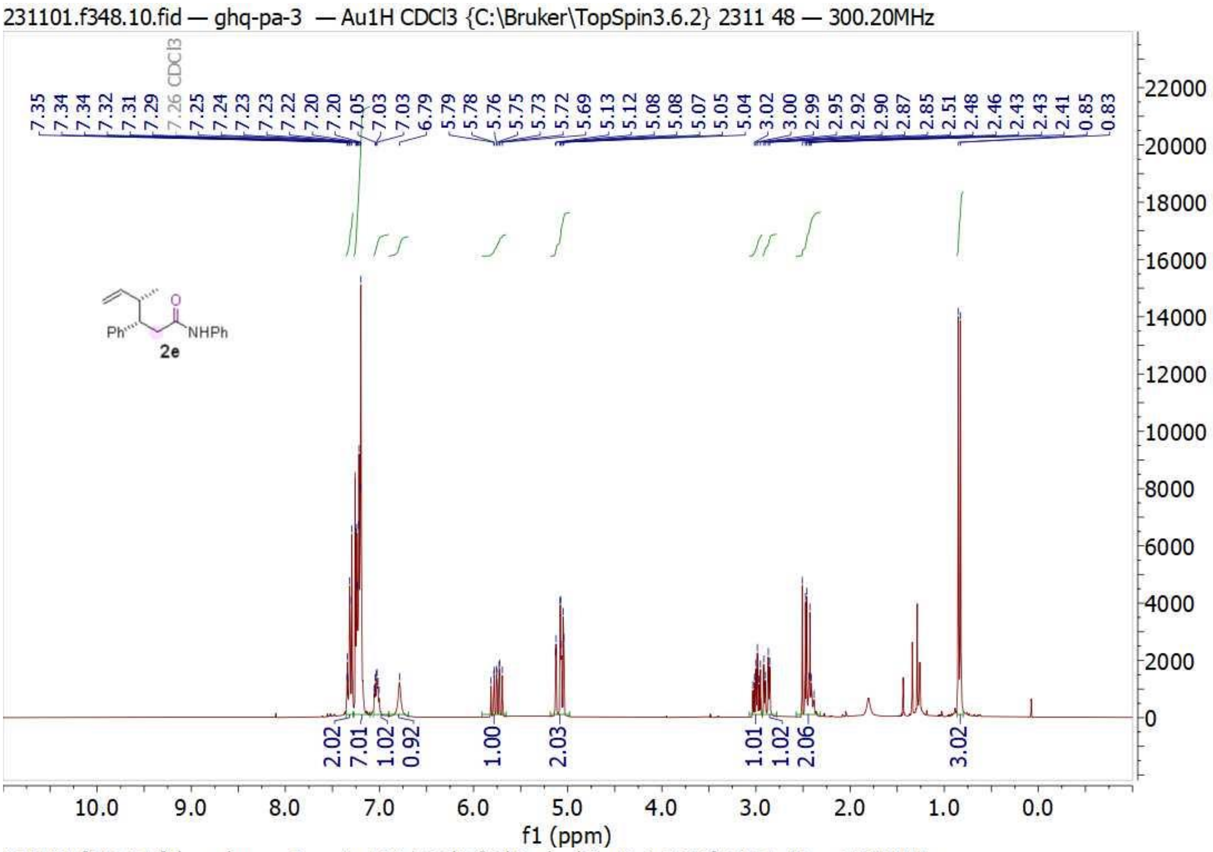
231101.f348.10.fid — ghq-pa-3 — Au1H CDCl3 {C:\Bruker\TopSpin3.6.2} 2311 48 — 300.20MHz
7.35 7.34 7.34 7.32 7.31 7.29 7.26 CDCl3 7.25 7.24 7.23 7.23 7.23 7.22 7.20 7.20 7.05 7.03 7.03 6.79 5.79 5.78 5.76 5.75 5.73 5.72 5.69 5.13 5.12 5.08 5.08 5.07 5.05 5.04 3.02 3.00 2.99 2.95 2.92 2.90 2.87 2.85 2.51 2.48 2.46 2.43 2.43 2.41 0.85 0.83
NHPh
Ph
2e
2.02 7.01 1.02 0.92 1.00 2.03 1.01 1.02 2.06 3.02
f1 (ppm)

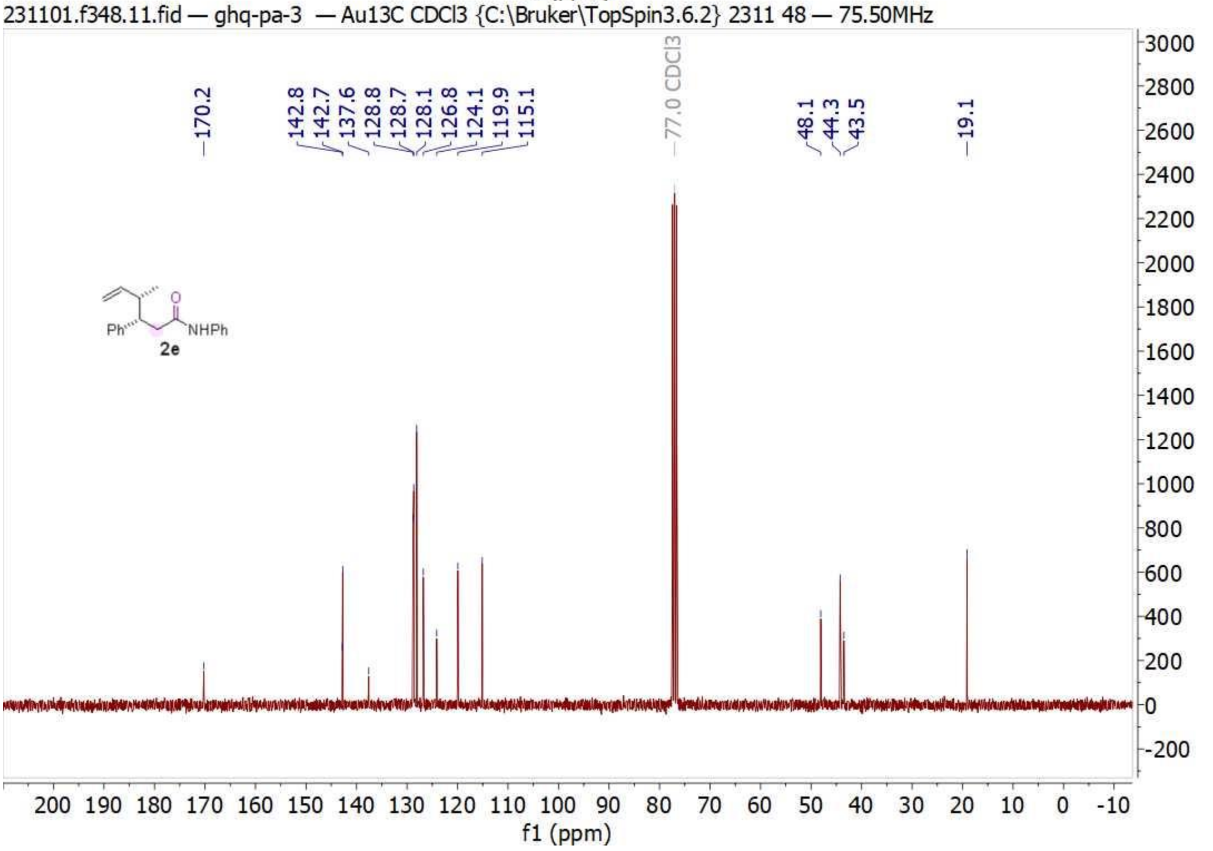
231101.f348.11.fid — ghq-pa-3 — Au13C CDCl3 {C:\Bruker\TopSpin3.6.2} 2311 48 — 75.50MHz
170.2
142.8 142.7 137.6 128.8 128.7 128.1 126.8 124.1 119.9 115.1
77.0 CDCl3
48.1 44.3 43.5
19.1
NHPh
Ph
2e
f1 (ppm)

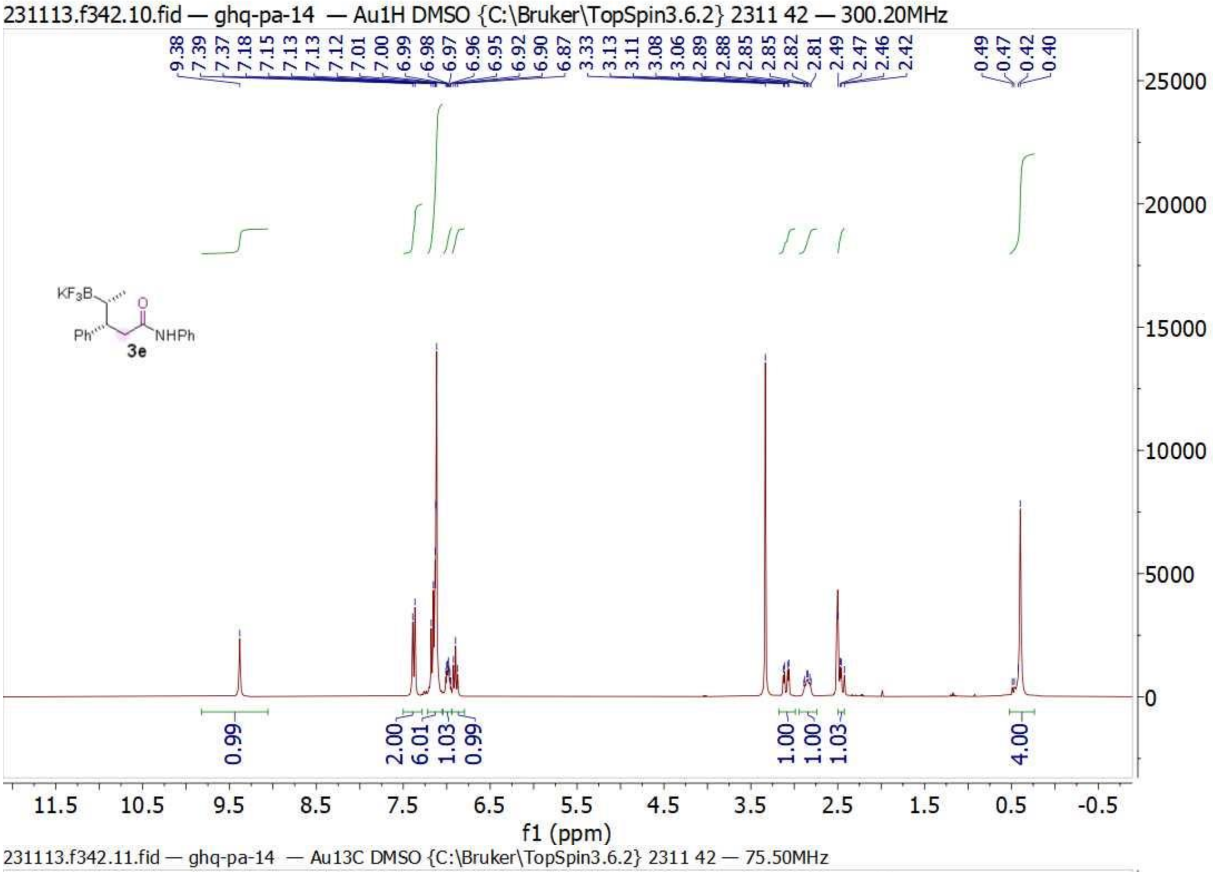
231113.f342.10.fid — ghq-pa-14 — Au1H DMSO {C:\Bruker\TopSpin3.6.2} 2311 42 — 300.20MHz
9.38 7.39 7.37 7.18 7.15 7.13 7.13 7.12 7.01 7.00 6.99 6.98 6.97 6.96 6.95 6.92 6.90 6.87 3.33 3.13 3.11 3.08 3.06 2.89 2.88 2.85 2.85 2.82 2.81 2.49 2.47 2.46 2.42 0.49 0.47 0.42 0.40
0.99 2.00 6.01 1.03 0.99 1.00 1.00 1.03 4.00
3e
f1 (ppm)

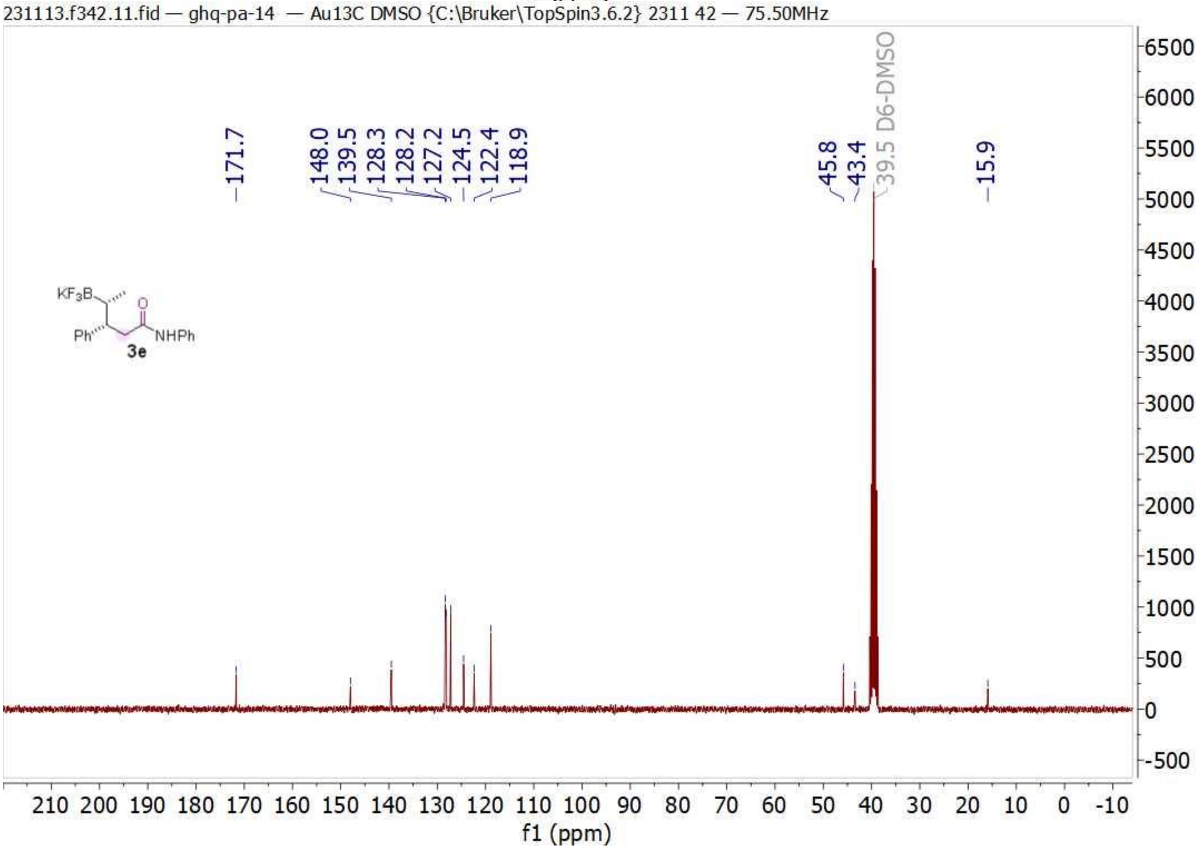
231113.f342.11.fid — ghq-pa-14 — Au13C DMSO {C:\Bruker\TopSpin3.6.2} 2311 42 — 75.50MHz
171.7 148.0 139.5 128.3 128.2 127.2 124.5 122.4 118.9 45.8 43.4 39.5 D6-DMSO 15.9
3e
f1 (ppm)

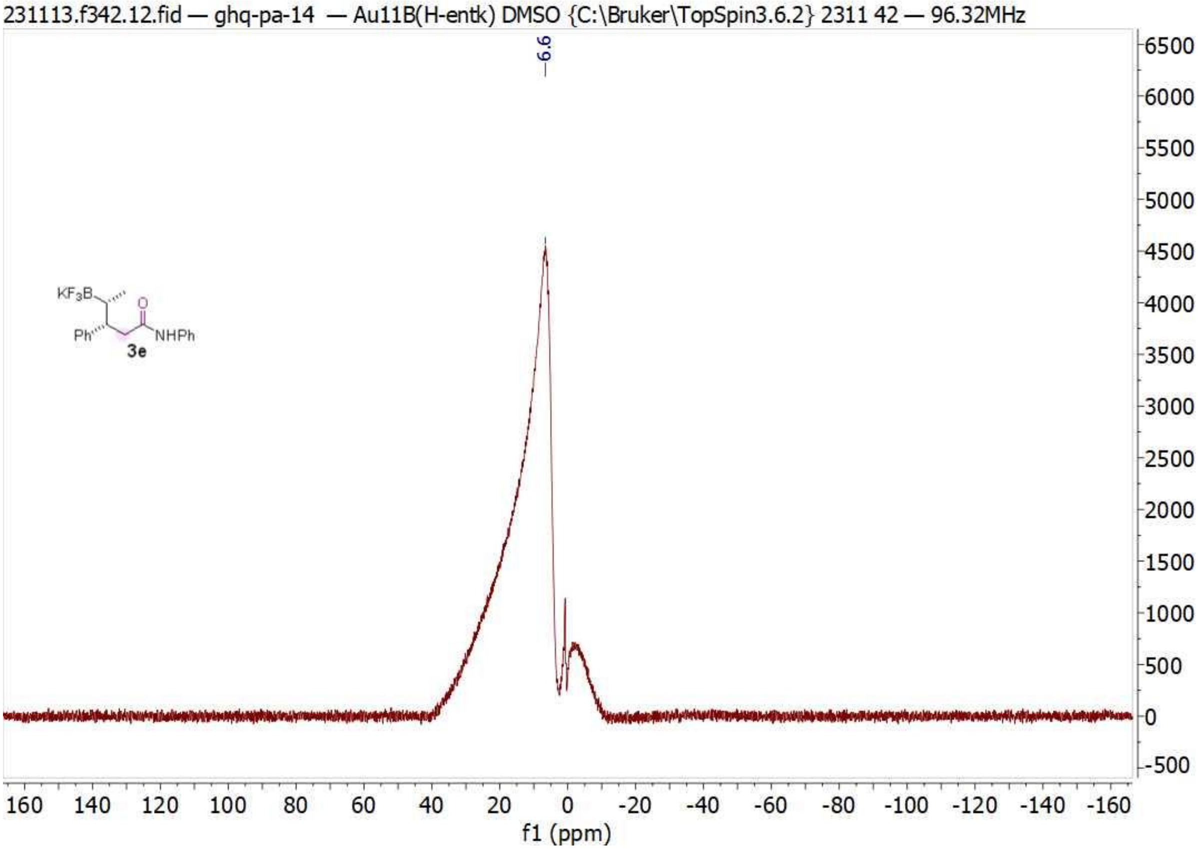
231113.f342.12.fid — ghq-pa-14 — Au11B(H-entk) DMSO {C:\Bruker\TopSpin3.6.2} 2311 42 — 96.32MHz
—6.6
KF3B
O
NHPh
Ph
3e
f1 (ppm)

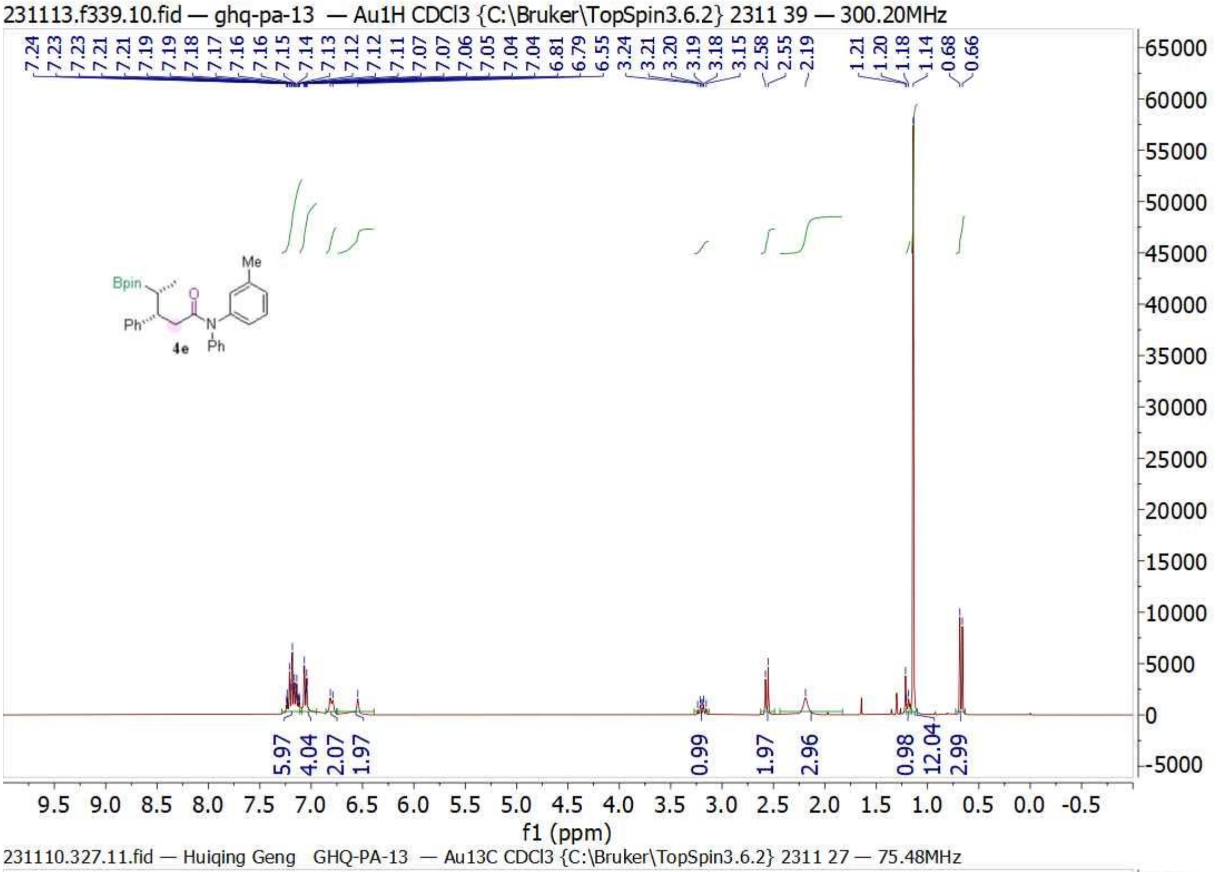
231113.f339.10.fid — ghq-pa-13 — Au1H CDCl3 {C:\Bruker\TopSpin3.6.2} 2311 39 — 300.20MHz
7.24 7.23 7.23 7.21 7.21 7.19 7.19 7.18 7.17 7.16 7.16 7.15 7.14 7.13 7.12 7.12 7.11 7.07 7.06 7.05 7.04 7.04 6.81 6.79 6.55 3.24 3.21 3.20 3.19 3.18 3.15 2.58 2.55 2.19 1.21 1.20 1.18 1.14 0.68 0.66
Bpin
Me
O
N
Ph
Ph
4e
5.97 4.04 2.07 1.97 0.99 1.97 2.96 0.98 12.04 2.99
f1 (ppm)

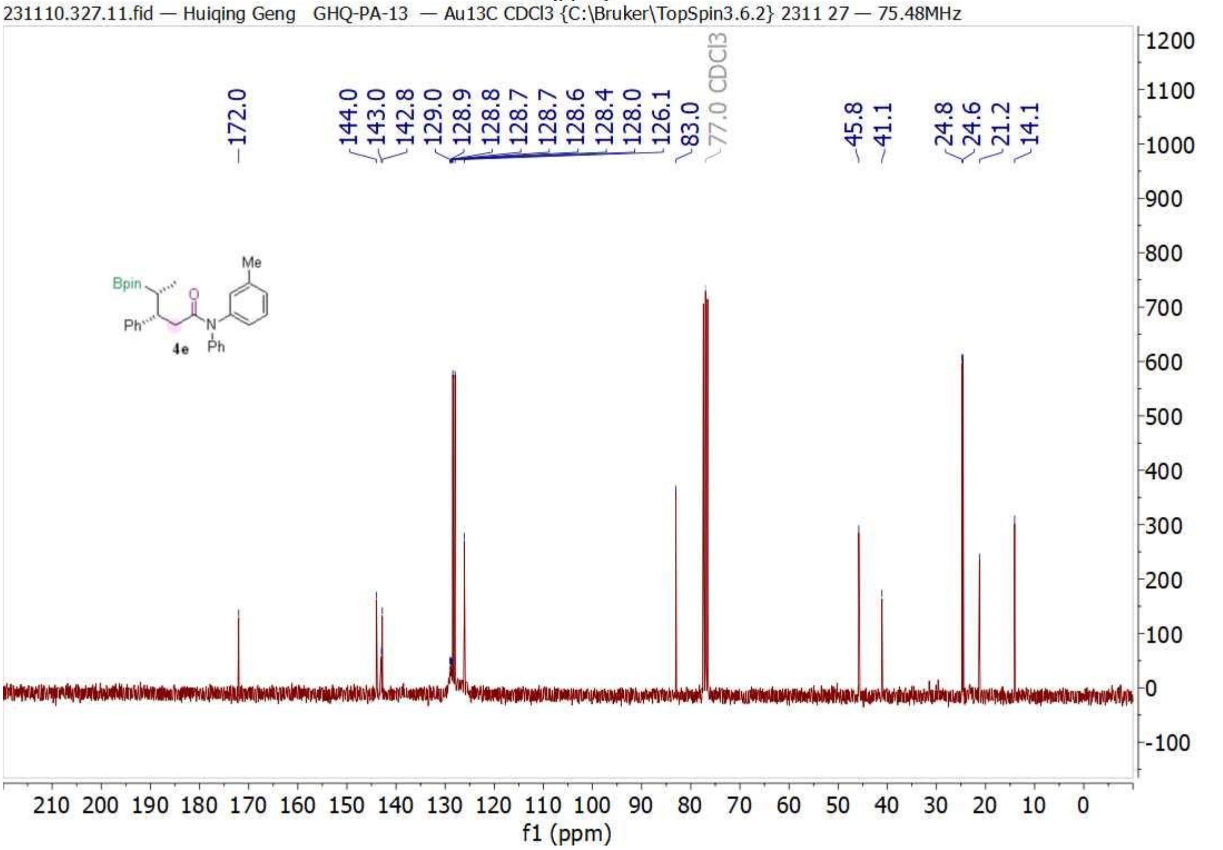
231110.327.11.fid — Huiqing Geng GHQ-PA-13 — Au13C CDCl3 {C:\Bruker\TopSpin3.6.2} 2311 27 — 75.48MHz
172.0 144.0 143.0 142.8 129.0 128.9 128.8 128.7 128.7 128.6 128.4 128.0 126.1 83.0 77.0 CDCl3 45.8 41.1 24.8 24.6 21.2 14.1
Bpin
Me
O
N
Ph
Ph
4e
f1 (ppm)

231110.327.12.fid — Huiqing Geng GHQ-PA-13 — Au11B CDCl3 {C:\Bruker\TopSpin3.6.2} 2311 27 — 96.29MHz

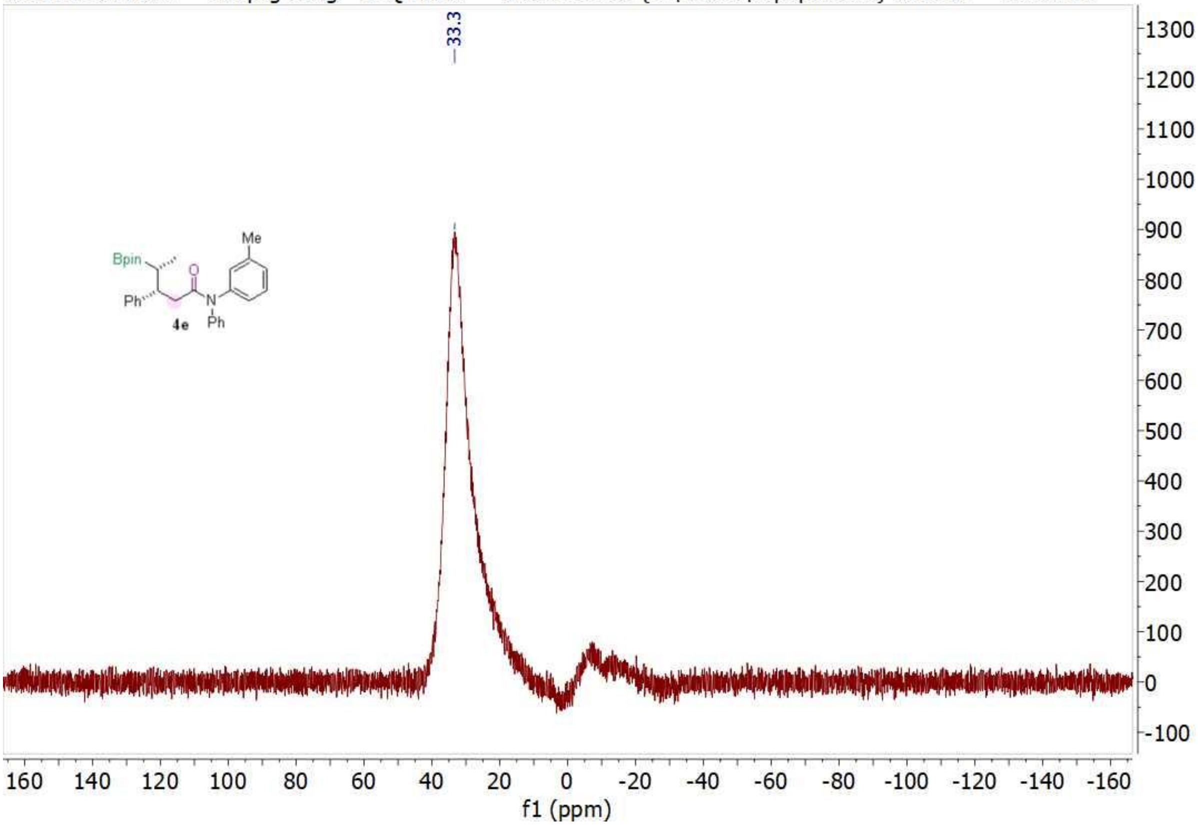

Supplement: SC-015-D4SC00156G-s001 [file SC-015-D4SC00156G-s001.pdf]
